# Supplementary material for: Structure and Fungicidal Activity of Secondary Metabolites Isolated from Trichoderma hamatum b-3
Source: J Fungi (Basel). 2024 Oct 31;10(11):755. doi: 10.3390/jof10110755 (PMC11595493; doi:10.3390/jof10110755)
Supplement: Supplementary file 1 [file jof-10-00755-s001.zip › jof-3251846-supplementary.pdf]

Supplementary materials

Structure and fungicidal activity of secondary metabolites isolated from *Trichoderma hamatum* b-3

Li Huang [1,#], Qiang Bian [2,#], Meng-Dan Liu [3], Yi-Wen Hu [1], Li-Juan Chen [1], Yu-Cheng Gu [3], Qi-Wei Zu [4], Guang-Zhi Wang [1,*] and Da-Le Guo [1,*]

1 State Key Laboratory of Southwestern Chinese Medicine Resource, School of Pharmacy, Chengdu University of Traditional Chinese Medicine, Chengdu, 611137, China (G.-Z. Wang; D.-L. Guo)
2 National Pesticide Engineering Research Center (Tianjin), College of Chemistry, Nankai University, Tianjin, 300071, China
3 School of Basic Medical Sciences, Chengdu University of Traditional Chinese Medicine, Chengdu, 611137, China
4 Syngenta Jealott's Hill International Research Centre, Syngenta, Berkshire, RG42 6EY, UK
5 Department of Biochemistry, College of Art & Science, Baylor University, Texas, 76706, USA

# These authors are co-first authors and contributed equally.
* Correspondence: wangguangzhi@cdutcm.edu.cn (G.-Z.Wang); guodale@cdutcm.edu.cn (D.-L. Guo)

Contents

Figure S 1. HRESIMS spectrum of compound 1 9
Figure S 2. UV spectrum of compound 1 9
Figure S 3. IR spectrum of compound 1 10
Figure S 4. The ^{1}H NMR spectrum of compound 1 in $CDCl_3$ (600MHz) 10
Figure S 5. The ^{13}C NMR spectrum of compound 1 in $CDCl_3$ (150MHz) 11
Figure S 6. The HSQC spectrum of compound 1 in $CDCl_3$ 11
Figure S 7. The ^{1}H-^{1}H COSY spectrum of compound 1 in $CDCl_3$ 12
Figure S 8. The HMBC spectrum of compound 1 in $CDCl_3$ 12
Figure S 9. The NOESY spectrum of compound 1 in $CDCl_3$ 13
Figure S 10. HRESIMS spectrum of compound 2 13
Figure S 11. UV spectrum of compound 2 14
Figure S 12. IR spectrum of compound 2 14
Figure S 13. The ^{1}H NMR spectrum of compound 2 in $CDCl_3$ (600MHz) 15
Figure S 14 The ^{13}C NMR spectrum of compound 2 in $CDCl_3$ (150MHz) 15
Figure S 15. The HSQC spectrum of compound 2 in $CDCl_3$ 16
Figure S 16. The ^{1}H-^{1}H COSY spectrum of compound 2 in $CDCl_3$ 16
Figure S 17. The HMBC spectrum of compound 2 in $CDCl_3$ 17
Figure S 18. The NOESY spectrum of compound 2 in $CDCl_3$ 17
Figure S 19. HRESIMS spectrum of compound 3 18
Figure S 20. UV spectrum of compound 3 18
Figure S 21. IR spectrum of compound 3 19
Figure S 22. The ^{1}H NMR spectrum of compound 3 in $CDCl_3$ (600MHz) 19
Figure S 23. The ^{13}C NMR spectrum of compound 3 in $CDCl_3$ (150MHz) 20
Figure S 24. The HSQC spectrum of compound 3 in $CDCl_3$ 20
Figure S 25. The ^{1}H-^{1}H COSY spectrum of compound 3 in $CDCl_3$ 21
Figure S 26. The HMBC spectrum of compound 3 in $CDCl_3$ 21
Figure S 27. The NOESY spectrum of compound 3 in $CDCl_3$ 22
Figure S 28. HRESIMS spectrum of compound 4 22
Figure S 29. UV spectrum of compound 4 23
Figure S 30. IR spectrum of compound 4 23
Figure S 31. The ^{1}H NMR spectrum of compound 4 in $CDCl_3$ (600MHz) 24
Figure S 32. The ^{13}C NMR spectrum of compound 4 in $CDCl_3$ (150MHz) 24
Figure S 33. The HSQC spectrum of compound 4 in $CDCl_3$ 25
Figure S 34. The ^{1}H-^{1}H COSY spectrum of compound 4 in $CDCl_3$ 25
Figure S 35. The HMBC spectrum of compound 4 in $CDCl_3$ 26
Figure S 36. The NOESY spectrum of compound 4 in $CDCl_3$ 26
Figure S 37. HRESIMS spectrum of compound 5 27
Figure S 38. UV spectrum of compound 5 27
Figure S 39. IR spectrum of compound 5 28
Figure S 40. The ^{1}H NMR spectrum of compound 5 in $CDCl_3$ (600MHz) 28
Figure S 41. The ^{13}C NMR spectrum of compound 5 in $CDCl_3$ (150MHz) 29
Figure S 42. The HSQC spectrum of compound 5 in $CDCl_3$ 29

Figure S 44. The HMBC spectrum of compound 5 in $CDCl_3$ 30
Figure S 45. The NOESY spectrum of compound 5 in $CDCl_3$ 31
Figure S 46. HRESIMS spectrum of compound 6 31
Figure S 47. UV spectrum of compound 6 32
Figure S 48. IR spectrum of compound 6 32
Figure S 49. The ^1H NMR spectrum of compound 6 in $CDCl_3$ (600MHz) 33
Figure S 50. The ^{13}C NMR spectrum of compound 6 in $CDCl_3$ (150MHz) 33
Figure S 51. The HSQC spectrum of compound 6 in $CDCl_3$ 34
Figure S 52. The ^1H-^1H COSY spectrum of compound 6 in $CDCl_3$ 34
Figure S 53. The HMBC spectrum of compound 6 in $CDCl_3$ 35
Figure S 54. The NOESY spectrum of compound 6 in $CDCl_3$ 35
Figure S 55. HRESIMS spectrum of compound 7 36
Figure S 56. UV spectrum of compound 7 36
Figure S 57. IR spectrum of compound 7 37
Figure S 58. The ^1H NMR spectrum of compound 7 in $CDCl_3$ (600MHz) 37
Figure S 59. The ^{13}C NMR spectrum of compound 7 in $CDCl_3$ (150MHz) 38
Figure S 60. The HSQC spectrum of compound 7 in $CDCl_3$ 38
Figure S 61. The ^1H-^1H COSY spectrum of compound 7 in $CDCl_3$ 39
Figure S 62. The HMBC spectrum of compound 7 in $CDCl_3$ 39
Figure S 63. The NOESY spectrum of compound 7 in $CDCl_3$ 40
Figure S 64. HRESIMS spectrum of compound 8 40
Figure S 65. UV spectrum of compound 8 41
Figure S 66. IR spectrum of compound 8 41
Figure S 67. The ^1H NMR spectrum of compound 8 in $CDCl_3$ (600MHz) 42
Figure S 68. The ^{13}C NMR spectrum of compound 8 in $CDCl_3$ (150MHz) 42
Figure S 69. The HSQC spectrum of compound 8 in $CDCl_3$ 43
Figure S 70. The ^1H-^1H COSY spectrum of compound 8 in $CDCl_3$ 43
Figure S 71. The HMBC spectrum of compound 8 in $CDCl_3$ 44
Figure S 72.The NOESY spectrum of compound 8 in $CDCl_3$ 44
Figure S 73. Most stable conformers of 2*S*, 5*R*, 6*R*, 13*S*, 14*S*-1 calculated at MPW1PW91/6-31G+d, p level 45
Figure S 74. Most stable conformers of 2*R*, 5*S*, 6*S*, 13*R*, 14*R*-1 calculated at MPW1PW91/6-31G+d, p level 48
Figure S 75. Most stable conformers of 2*S*, 3*S*, 5*R*, 6*R*, 13*S*, 14*S*-2 calculated at MPW1PW91/6-31G+d, p level 49
Figure S 76. Most stable conformers of 2*R*, 3*R*, 5*S*, 6*S*, 13*R*, 14*R*-2 calculated at MPW1PW91/6-31G+d, p level 52
Figure S 77. Most stable conformers of 2*S*, 3*R*, 6*R*, 7*R*-3 calculated at MPW1PW91/6-31G+d, p level 56
Figure S 78. Most stable conformers of 2*S*, 3*R*, 6*R*, 7*S*-3 calculated at MPW1PW91/6-31G+d, p level 65
Figure S 79. Correlation between the calculated ^{13}C NMR data for 2*S*, 3*R*, 6*R*, 7*R*-3 (a) and 2*S*, 3*R*, 6*R*, 7*S*-3 (b) and experimental ^{13}C NMR data of 3 73
Figure S 80. Differences between experimental NMR chemical shifts of 3 and theoretical NMR

chemical shifts for 2*S*, 3*R*, 6*R*, 7*R*-3 (yellow bar) and 2*S*, 3*R*, 6*R*, 7*S*-3 (purple bar) 73
Figure S 81. Most stable conformers of 2*S*, 3*R*, 6*R*, 7*R*-3 at the CAM-B3LYP/DGDZVP level 75
Figure S 82. Most stable conformers of 2*R*, 3*S*, 6*S*, 7*S*-3 at the CAM-B3LYP/DGDZVP level 84
Figure S 83. Most stable conformers of 2*S*, 3*R*, 6*R*, 7*R*, 10*R*-4 calculated at MPW1PW91/6-31G+d, p level 91
Figure S 84. Most stable conformers of 2*S*, 3*R*, 6*R*, 7*R*, 10*S*-4 calculated at MPW1PW91/6-31G+d, p level 100
Figure S 85. Most stable conformers of 2*S*, 3*R*, 6*R*, 7*S*, 10*R*-4 calculated at MPW1PW91/6-31G+d, p level 109
Figure S 86. Most stable conformers of 2*S*, 3*R*, 6*R*, 7*S*, 10*S*-4 calculated at MPW1PW91/6-31G+d, p level 118
Figure S 87. Correlation between the calculated ^{13}C NMR data for 2*S*, 3*R*, 6*R*, 7*R*, 10*R*-4 (a); 2*S*, 3*R*, 6*R*, 7*R*, 10*S*-4 (b); 2*S*, 3*R*, 6*R*, 7*S*, 10*R*-4 (c) and 2*S*, 3*R*, 6*R*, 7*S*, 10*S*-4 (d) and experimental ^{13}C NMR data of 4 127
Figure S 88. Differences between experimental NMR chemical shifts of 3 and theoretical NMR chemical shifts for 2*S*, 3*R*, 6*R*, 7*R*, 10*R*-4 (purple bar); 2*S*, 3*R*, 6*R*, 7*R*, 10*S*-4 (yellow bar); 2*S*, 3*R*, 6*R*, 7*S*, 10*R*-4 (green bar) and 2*S*, 3*R*, 6*R*, 7*S*, 10*R*-4 (pink bar). 127
Figure S 89. Most stable conformers of 2*S*, 3*R*, 6*R*, 7*R*, 10*R*-4 at the CAM-B3LYP/DGDZVP level 129
Figure S 90. Most stable conformers of 2*R*, 3*S*, 6*S*, 7*S*, 10*S*-4 at the CAM-B3LYP/DGDZVP level 139
Figure S 91. Most stable conformers of 2*S*, 3*R*, 6*R*, 7*R*-5 calculated at MPW1PW91/6-31G+d, p level 148
Figure S 92. Most stable conformers of 2*S*, 3*R*, 6*R*, 7*S*-5 calculated at MPW1PW91/6-31G+d, p level 156
Figure S 93. Correlation between the calculated ^{13}C NMR data for 2*S*, 3*R*, 6*R*, 7*R*-5 (a) and 2*S*, 3*R*, 6*R*, 7*S*-5 (b) and experimental ^{13}C NMR data of 5 162
Figure S 94. Differences between experimental NMR chemical shifts of 5 and theoretical NMR chemical shifts for 2*S*, 3*R*, 6*R*, 7*R*-5 (purple bar) and 2*S*, 3*R*, 6*R*, 7*S*-5 (yellow bar) 163
Figure S 95. Most stable conformers of 2*S*, 3*R*, 6*R*, 7*R*-5 at the CAM-B3LYP/DGDZVP level .. 164
Figure S 96. Most stable conformers of 2*R*, 3*S*, 6*S*, 7*S*-5 at the CAM-B3LYP/DGDZVP level ... 172
Figure S 97. Most stable conformers of 2*S*, 3*R*, 6*R*, 7*R*-6 calculated at MPW1PW91/6-31G+d, p level 179
Figure S 98. Most stable conformers of 2*S*, 3*R*, 6*R*, 7*S*-6 calculated at MPW1PW91/6-31G+d, p level 186
Figure S 99. Correlation between the calculated ^{13}C NMR data for 2*S*, 3*R*, 6*R*, 7*R*-6 (a) and 2*S*, 3*R*, 6*R*, 7*S*-6 (b) and experimental ^{13}C NMR data of 6 191
Figure S 100. Differences between experimental NMR chemical shifts of 6 and theoretical NMR chemical shifts for 2*S*, 3*R*, 6*R*, 7*R*-6 (purple bar) and 2*S*, 3*R*, 6*R*, 7*S*-6 (yellow bar) 191
Figure S 101. Most stable conformers of 2*S*, 3*R*, 6*R*, 7*R*-6 at the CAM-B3LYP/DGDZVP level 193
Figure S 102. Most stable conformers of 2*R*, 3*S*, 6*S*, 7*S*-6 at the CAM-B3LYP/DGDZVP level. 199
Figure S 103. Most stable conformers of 2*S*, 3*R*, 6*R*, 7*R*-7 calculated at MPW1PW91/6-31G+d, p level 202
Figure S 104. Most stable conformers of 2*S*, 3*R*, 6*R*, 7*S*-7 calculated at MPW1PW91/6-31G+d, p

level..208
Figure S 105. Correlation between the calculated ^{13}C NMR data for 2*S*, 3*R*, 6*R*, 7*R*-7 (a) and 2*S*, 3*R*, 6*R*, 7*S*-7 (b) and experimental ^{13}C NMR data of 7..212
Figure S 106. Differences between experimental NMR chemical shifts of 7 and theoretical NMR chemical shifts for 2*S*, 3*R*, 6*R*, 7*R*-7 (purple bar) and 2*S*, 3*R*, 6*R*, 7*S*-7 (yellow bar)..................212
Figure S 107. Most stable conformers of 2*S*, 3*R*, 6*R*, 7*R*-7 at the CAM-B3LYP/DGDZVP level 214
Figure S 108. Most stable conformers of 2*R*, 3*S*, 6*S*, 7*S*-7 at the CAM-B3LYP/DGDZVP level.220
Figure S 109. Most stable conformers of 2*S*, 3*R*, 6*R*, 7*R, 10S*-8 at the CAM-B3LYP/DGDZVP level..228
Figure S 110. Most stable conformers of 2*R*, 3*S*, 6*S*, 7*S, 10R*-8 at the CAM-B3LYP/DGDZVP level..237
Figure S 111. Morphological characteristics of b-3. **a** Characteristics of colony of strain b-3 on PDA. **b** Conidia of strain b-3. **c** Spores of strain b-3……………....……………….………242

Table S 1. Optimized Z-Matrixes of 2*S*, 5*R*, 6*R*, 13*S*, 14*S*-1 conformations in the methanol at CAM-B3LYP/DGDZVP level 45
Table S 2. Energy analysis for 2*S*, 5*R*, 6*R*, 13*S*, 14*S*-1..47
Table S 3. Optimized Z-Matrixes of 2*R*, 5*S*, 6*S*, 13*R*, 14*R*-1 conformations in the methanol at CAM-B3LYP/DGDZVP level. 48
Table S 4. Energy analysis for 2*R*, 5*S*, 6*S*, 13*R*, 14*R*-1 49
Table S 5. Optimized Z-Matrixes of 2*S*, 3*S*, 5*R*, 6*R*, 13*S*, 14*S*-2 conformations in the Chloroform at CAM-B3LYP/DGDZVP level...49
Table S 6. Energy analysis for 2*S*, 3*S*, 5*R*, 6*R*, 13*S*, 14*S*-2..52
Table S 7. Optimized Z-Matrixes of 2*R*, 3*R*, 5*S*, 6*S*, 13*R*, 14*R*-2 conformations in the Chloroform at CAM-B3LYP/DGDZVP level...52
Table S 8. Energy analysis for 2*R*, 3*R*, 5*S*, 6*S*, 13*R*, 14*R*-2...55
Table S 9. Optimized Z-Matrixes of 2*S*, 3*R*, 6*R*, 7*R*-3 conformations in the Chloroform at MPW1PW91/6-31G+d, p level...56
Table S 10. Energy analysis for 2*S*, 3*R*, 6*R*, 7*R*-3 in the Chloroform at MPW1PW91/6-31G+d, p level..64
Table S 11. Optimized Z-Matrixes of 2*S*, 3*R*, 6*R*, 7*S*-3 conformations in the Chloroform at MPW1PW91/6-31G+d, p level...65
Table S 12. Energy analysis for 2*S*, 3*R*, 6*R*, 7*S*-3 in the Chloroform at MPW1PW91/6-31G+d, p level..72
Table S 13. Experimental chemical shifts of 3, the calculated shielding tensors for 2*S*, 3*R*, 6*R*, 7*R*-3 (isomer 1) and 2*S*, 3*R*, 6*R*, 7*S*-3 (isomer 2), as well as their DP4+ probability......................73
Table S 14. Optimized Z-Matrixes of 2*S*, 3*R*, 6*R*, 7*R*-3 conformations in the methanol at CAM-B3LYP/DGDZVP level 75
Table S 15. Energy analysis for 2*S*, 3*R*, 6*R*, 7*R*-3 83
Table S 16. Optimized Z-Matrixes of 2*R*, 3*S*, 6*S*, 7*S*-3 conformations in the methanol at CAM-B3LYP/DGDZVP level 84
Table S 17. Energy analysis for 2*R*, 3*S*, 6*S*, 7*S*-3 90
Table S 18. Optimized Z-Matrixes of 2*S*, 3*R*, 6*R*, 7*R*, 10*R*-4 conformations in the Chloroform at MPW1PW91/6-31G+d, p level...91
Table S 19. Energy analysis for 2*S*, 3*R*, 6*R*, 7*R*, 10*R*-4 in the Chloroform at MPW1PW91/6-31G+d, p level..99
Table S 20. Optimized Z-Matrixes of 2*S*, 3*R*, 6*R*, 7*R*, 10*S*-4 conformations in the Chloroform at MPW1PW91/6-31G+d, p level...100
Table S 21. Energy analysis for 2*S*, 3*R*, 6*R*, 7*R*, 10*S*-4 in the Chloroform at MPW1PW91/6-31G+d, p level..108
Table S 22. Optimized Z-Matrixes of 2*S*, 3*R*, 6*R*, 7*S*, 10*R*-4 conformations in the Chloroform at MPW1PW91/6-31G+d, p level...109
Table S 23. Energy analysis for 2*S*, 3*R*, 6*R*, 7*S*, 10*R*-4 in the Chloroform at MPW1PW91/6-31G+d, p level..117
Table S 24. Optimized Z-Matrixes of 2*S*, 3*R*, 6*R*, 7*S*, 10*S*-4 conformations in the Chloroform at MPW1PW91/6-31G+d, p level...118
Table S 25. Energy analysis for 2*S*, 3*R*, 6*R*, 7*S*, 10*S*-4 in the Chloroform at MPW1PW91/6-31G+d,

p level..126
Table S 26. Experimental chemical shifts of 4, the calculated shielding tensors for 2*S*, 3*R*, 6*R*, 7*R*, 10*R*-4 (isomer 1); 2*S*, 3*R*, 6*R*, 7*R*, 10*S*-4 (isomer 2); 2*S*, 3*R*, 6*R*, 7*S*, 10*R*-4 (isomer 3) and 2*S*, 3*R*, 6*R*, 7*S*, 10*S*-4 (isomer 4), as well as their DP4+ probability..127
Table S 27. Optimized Z-Matrixes of 2*S*, 3*R*, 6*R*, 7*R*, 10*R*-4 conformations in the methanol at CAM-B3LYP/DGDZVP level 129
Table S 28. Energy analysis for 2*S*, 3*R*, 6*R*, 7*R*, 10*R*-4...138
Table S 29. Optimized Z-Matrixes of 2*R*, 3*S*, 6*S*, 7*S*, 10*S*-4 conformations in the methanol at CAM-B3LYP/DGDZVP level 139
Table S 30. Energy analysis for 2*R*, 3*S*, 6*S*, 7*S*, 10*S*-4 147
Table S 31. Optimized Z-Matrixes of 2*S*, 3*R*, 6*R*, 7*R*-5 conformations in the Chloroform at MPW1PW91/6-31G+d, p level..148
Table S 32. Energy analysis for 2*S*, 3*R*, 6*R*, 7*R*-5 in the Chloroform at MPW1PW91/6-31G+d, p level..155
Table S 33. Optimized Z-Matrixes of 2*S*, 3*R*, 6*R*, 7*S*-5 conformations in the Chloroform at MPW1PW91/6-31G+d, p level..156
Table S 34. Energy analysis for 2*S*, 3*R*, 6*R*, 7*S*-5 in the Chloroform at MPW1PW91/6-31G+d, p level..162
Table S 35. Experimental chemical shifts of 5, the calculated shielding tensors for 2*S*, 3*R*, 6*R*, 7*R*-5 (isomer 1) and 2*S*, 3*R*, 6*R*, 7*S*-5 (isomer 2), as well as their DP4+ probability...................163
Table S 36. Optimized Z-Matrixes of 2*S*, 3*R*, 6*R*, 7*R*-5 conformations in the methanol at CAM-B3LYP/DGDZVP level 164
Table S 37. Energy analysis for 2*S*, 3*R*, 6*R*, 7*R*-5 171
Table S 38. Optimized Z-Matrixes of 2*R*, 3*S*, 6*S*, 7*S*-5 conformations in the methanol at CAM-B3LYP/DGDZVP level 172
Table S 39. Energy analysis for 2*R*, 3*S*, 6*S*, 7*S*-5 179
Table S 40. Optimized Z-Matrixes of 2*S*, 3*R*, 6*R*, 7*R*-6 conformations in the Chloroform at MPW1PW91/6-31G+d, p level..179
Table S 41. Energy analysis for 2*S*, 3*R*, 6*R*, 7*R*-6 in the Chloroform at MPW1PW91/6-31G+d, p level..185
Table S 42. Optimized Z-Matrixes of 2*S*, 3*R*, 6*R*, 7*S*-6 conformations in the Chloroform at MPW1PW91/6-31G+d, p level..186
Table S 43. Energy analysis for 2*S*, 3*R*, 6*R*, 7*S*-6 in the Chloroform at MPW1PW91/6-31G+d, p level..190
Table S 44. Experimental chemical shifts of 6, the calculated shielding tensors for 2*S*, 3*R*, 6*R*, 7*R*-6 (isomer 1) and 2*S*, 3*R*, 6*R*, 7*S*-6 (isomer 2), as well as their DP4+ probability...................191
Table S 45. Optimized Z-Matrixes of 2*S*, 3*R*, 6*R*, 7*R*-6 conformations in the methanol at CAM-B3LYP/DGDZVP level 193
Table S 46. Energy analysis for 2*S*, 3*R*, 6*R*, 7*R*-6 199
Table S 47. Optimized Z-Matrixes of 2*R*, 3*S*, 6*S*, 7*S*-6 conformations in the methanol at CAM-B3LYP/DGDZVP level 199
Table S 48. Energy analysis for 2*R*, 3*S*, 6*S*, 7*S*-6 202
Table S 49. Optimized Z-Matrixes of 2*S*, 3*R*, 6*R*, 7*R*-7 conformations in the Chloroform at MPW1PW91/6-31G+d, p level..202

Table S 50. Energy analysis for 2*S*, 3*R*, 6*R*, 7*R*-7 in the Chloroform at MPW1PW91/6-31G+d, p level....208
Table S 51. Optimized Z-Matrixes of 2*S*, 3*R*, 6*R*, 7*S*-7 conformations in the Chloroform at MPW1PW91/6-31G+d, p level....208
Table S 52. Energy analysis for 2*S*, 3*R*, 6*R*, 7*S*-7 in the Chloroform at MPW1PW91/6-31G+d, p level....211
Table S 53. Experimental chemical shifts of 7, the calculated shielding tensors for 2*S*, 3*R*, 6*R*, 7*R*-7 (isomer 1) and 2*S*, 3*R*, 6*R*, 7*S*-7 (isomer 2), as well as their DP4+ probability....212
Table S 54. Optimized Z-Matrixes of 2*S*, 3*R*, 6*R*, 7*R*-7 conformations in the methanol at CAM-B3LYP/DGDZVP level....214
Table S 55. Energy analysis for 2*S*, 3*R*, 6*R*, 7*R*-7....220
Table S 56. Optimized Z-Matrixes of 2*R*, 3*S*, 6*S*, 7*S*-7 conformations in the methanol at CAM-B3LYP/DGDZVP level....220
Table S 57. Energy analysis for 2*R*, 3*S*, 6*S*, 7*S*-7....227
Table S 58. Optimized Z-Matrixes of 2*S*, 3*R*, 6*R*, 7*R, 10S*-8 conformations in the methanol at CAM-B3LYP/DGDZVP level....228
Table S 59. Energy analysis for 2*S*, 3*R*, 6*R*, 7*R, 10S*-8....236
Table S 60. Optimized Z-Matrixes of 2*R*, 3*S*, 6*S*, 7*S, 10R*-8 conformations in the methanol at CAM-B3LYP/DGDZVP level....237
Table S 61. Energy analysis for 2*R*, 3*S*, 6*S*, 7*S, 10R*-8....242

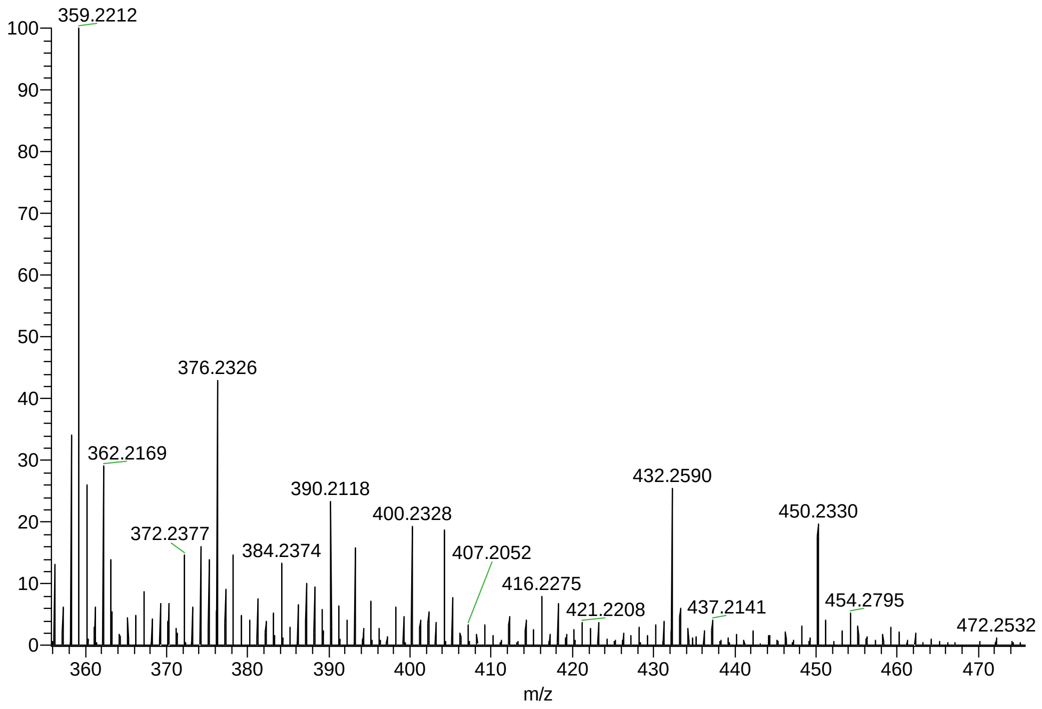

Figure S 1. HRESIMS spectrum of compound 1

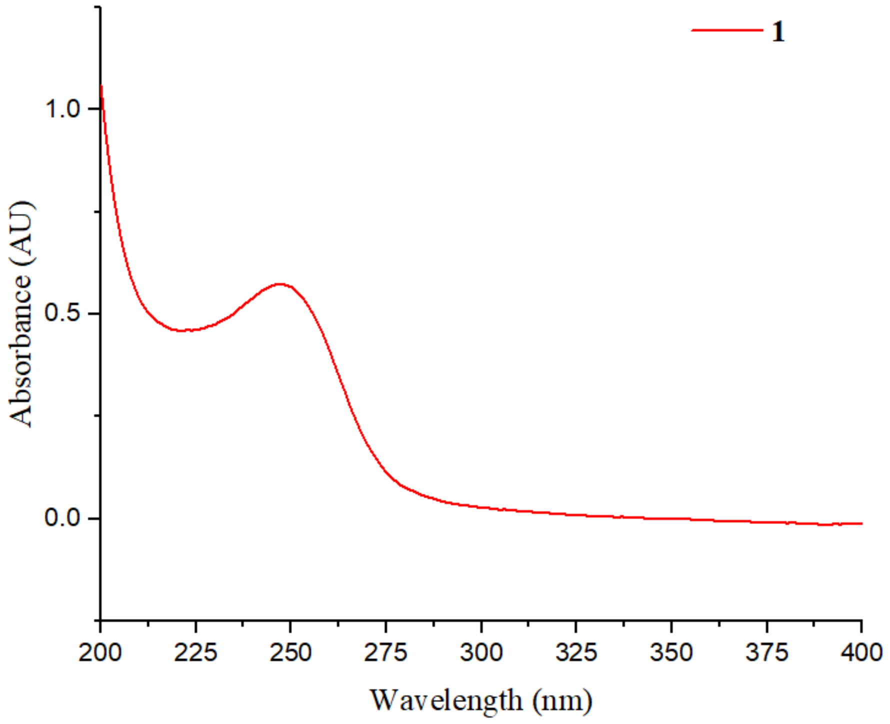

Figure S 2. UV spectrum of compound 1

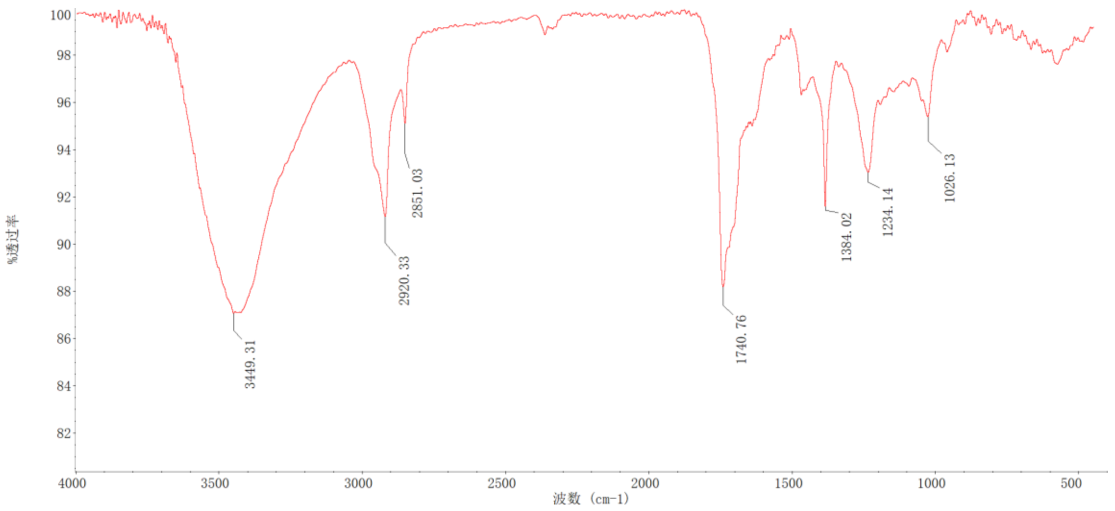

Figure S 3. IR spectrum of compound 1

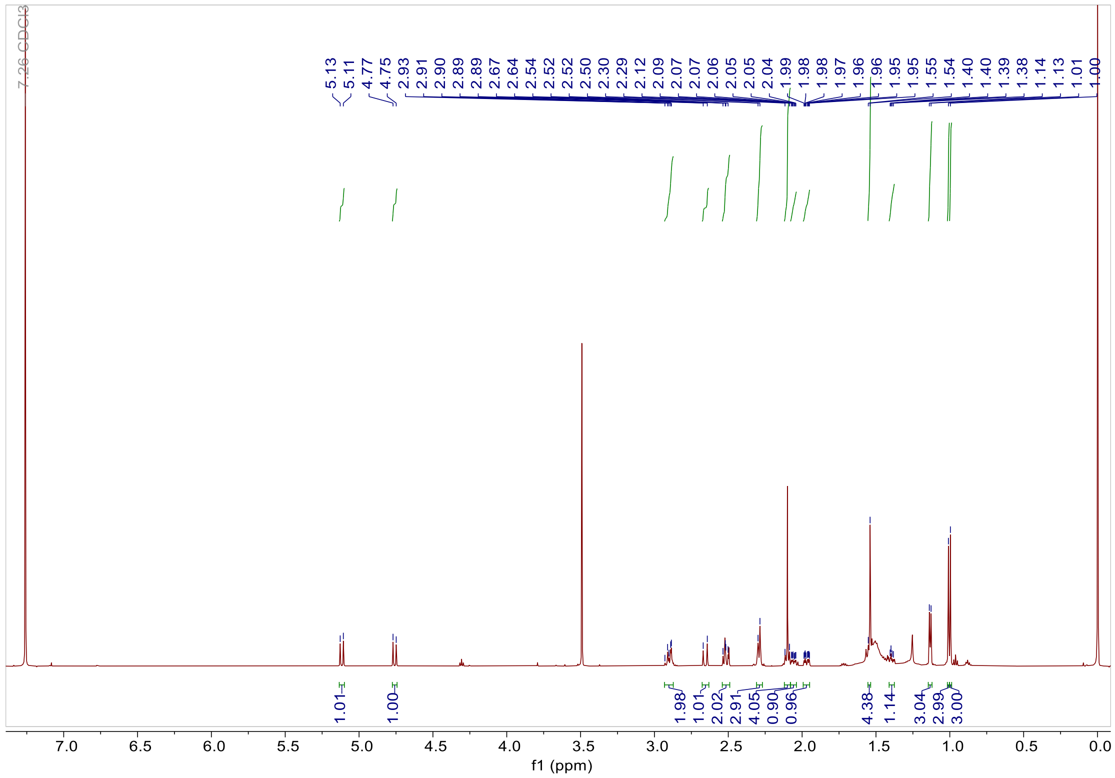

Figure S 4. The ^{1}H NMR spectrum of compound 1 in $CDCl_3$ (600MHz)

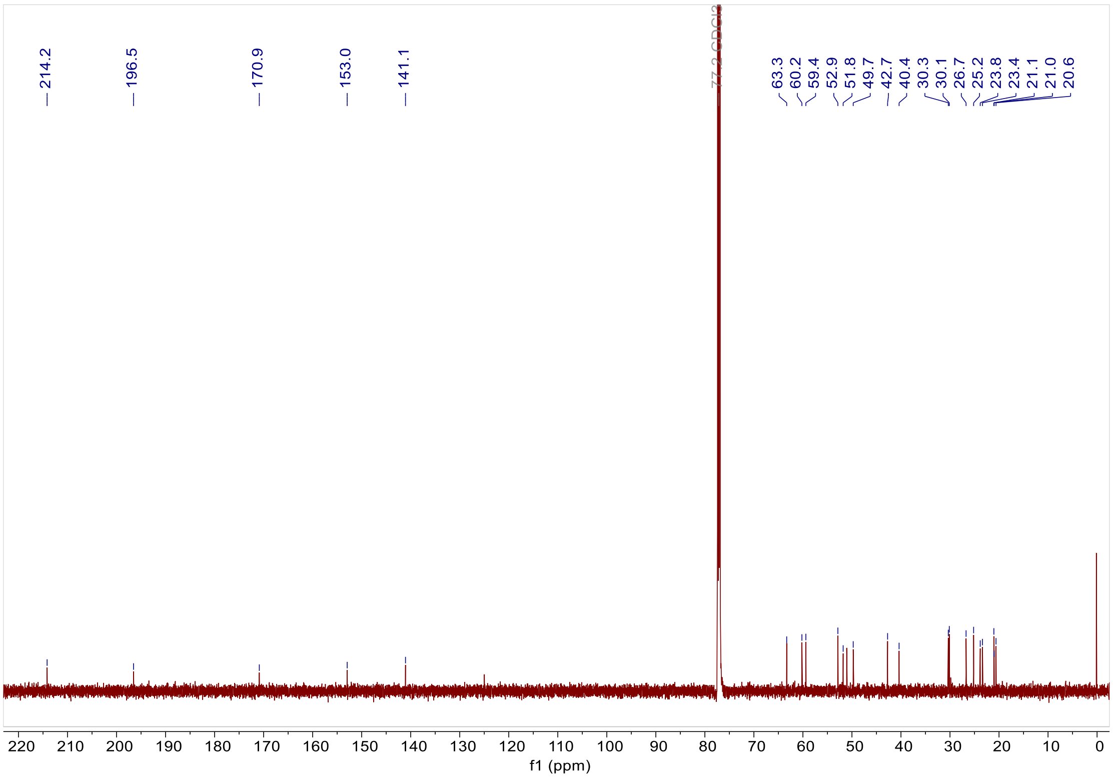

Figure S 5. The ^{13}C NMR spectrum of compound 1 in $CDCl_3$ (150MHz)

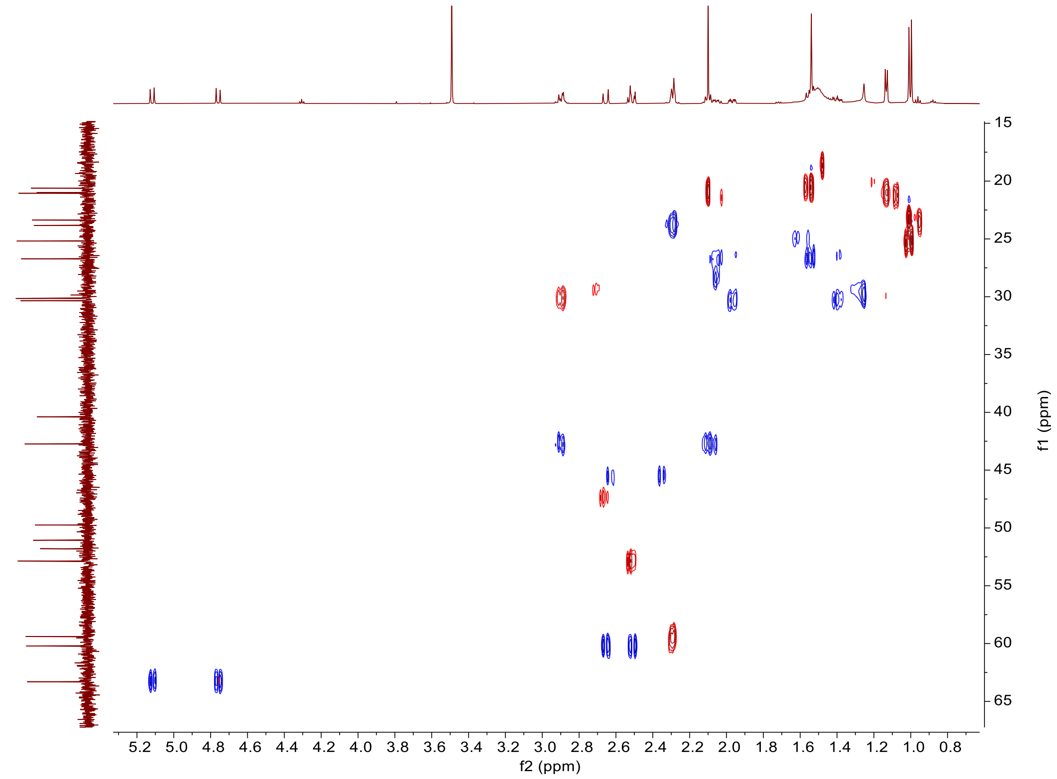

Figure S 6. The HSQC spectrum of compound 1 in $CDCl_3$

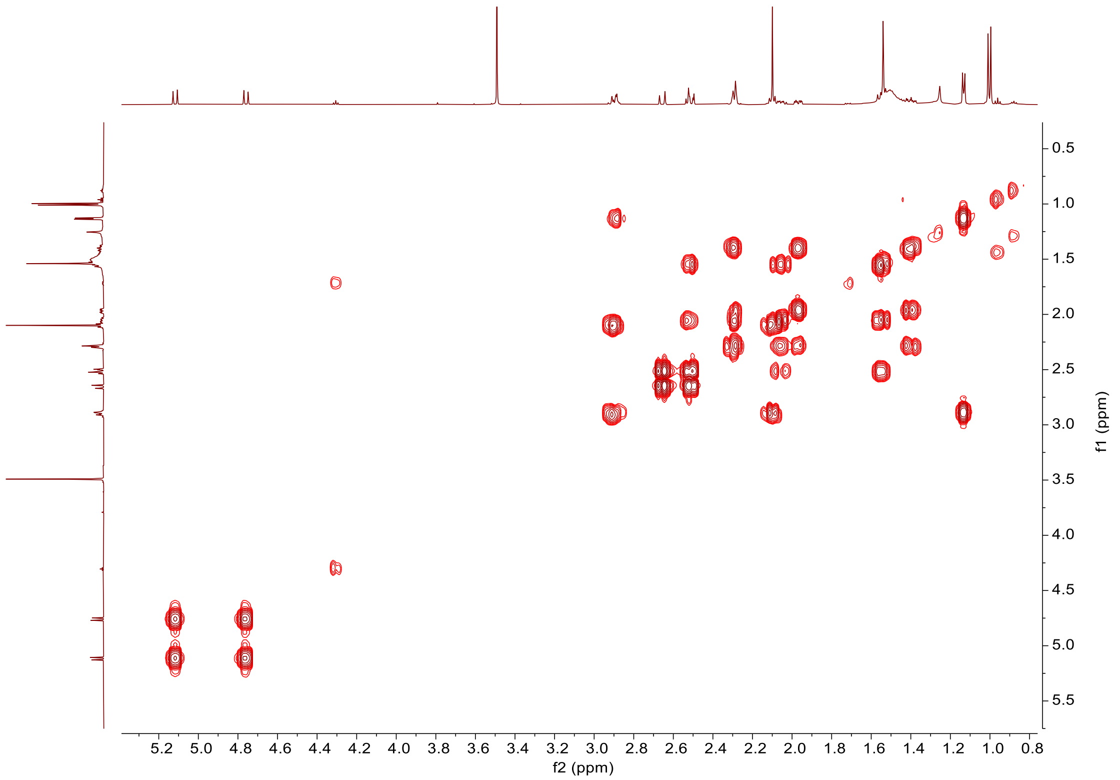

Figure S 7. The ^{1}H-^{1}H COSY spectrum of compound 1 in $CDCl_3$

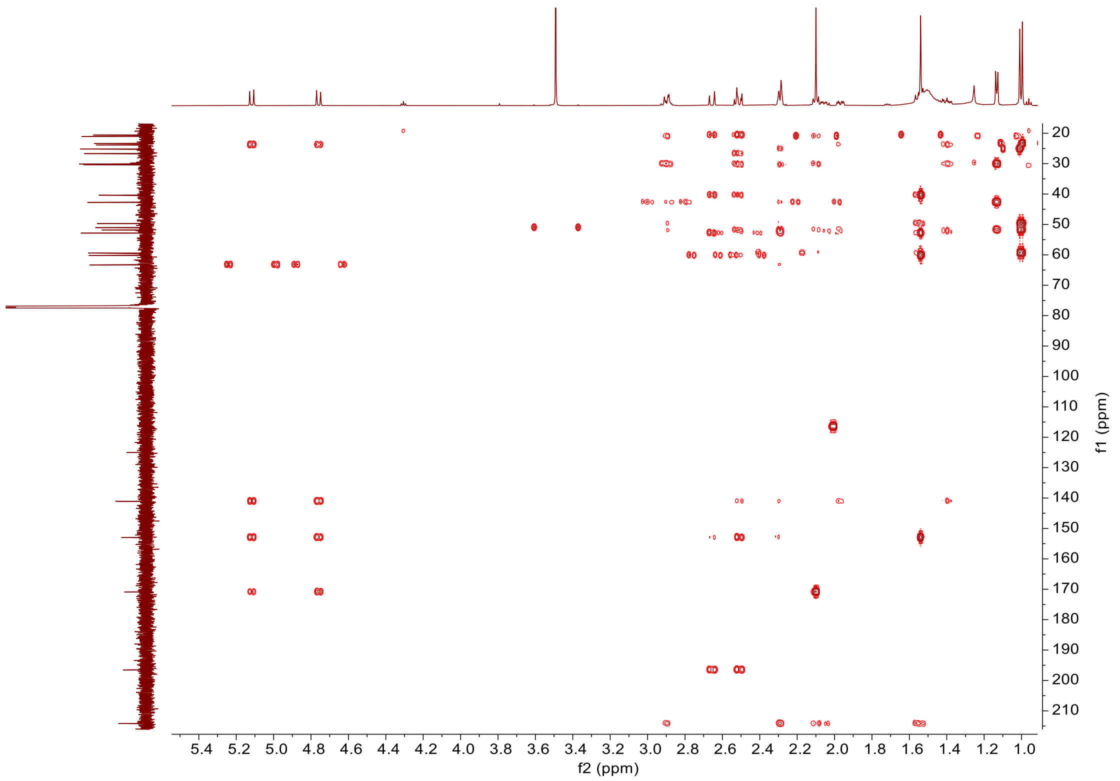

Figure S 8. The HMBC spectrum of compound 1 in $CDCl_3$

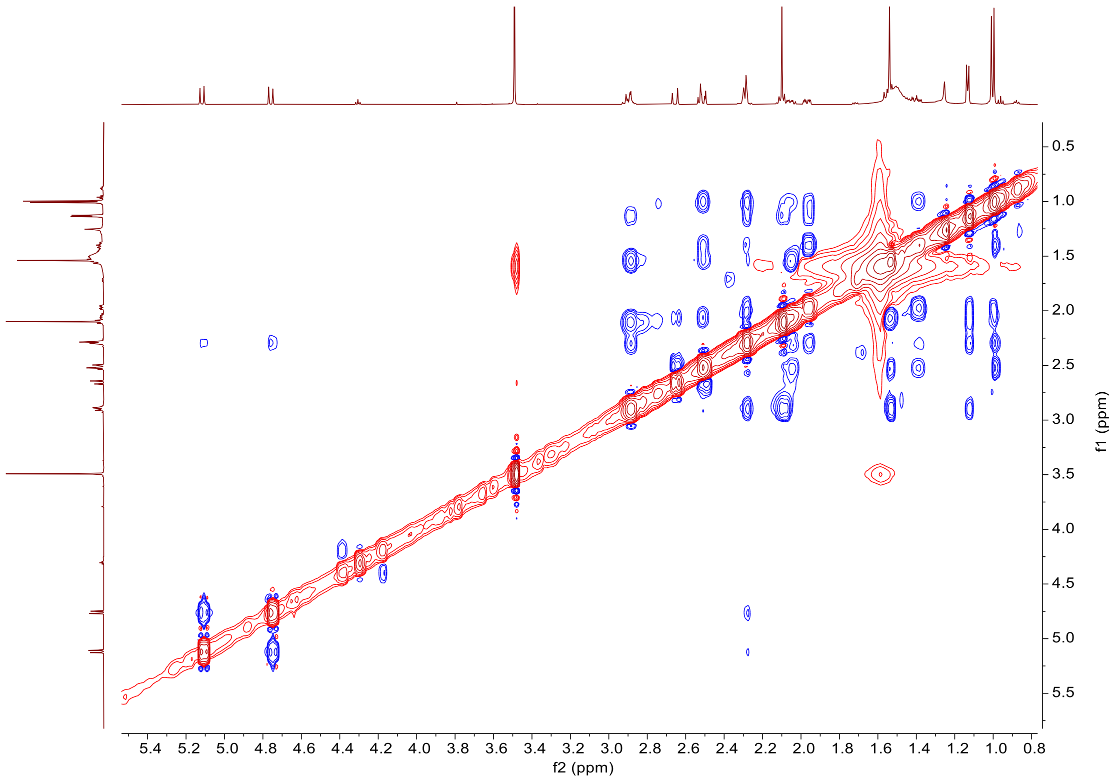

Figure S 9. The NOESY spectrum of compound 1 in $CDCl_3$

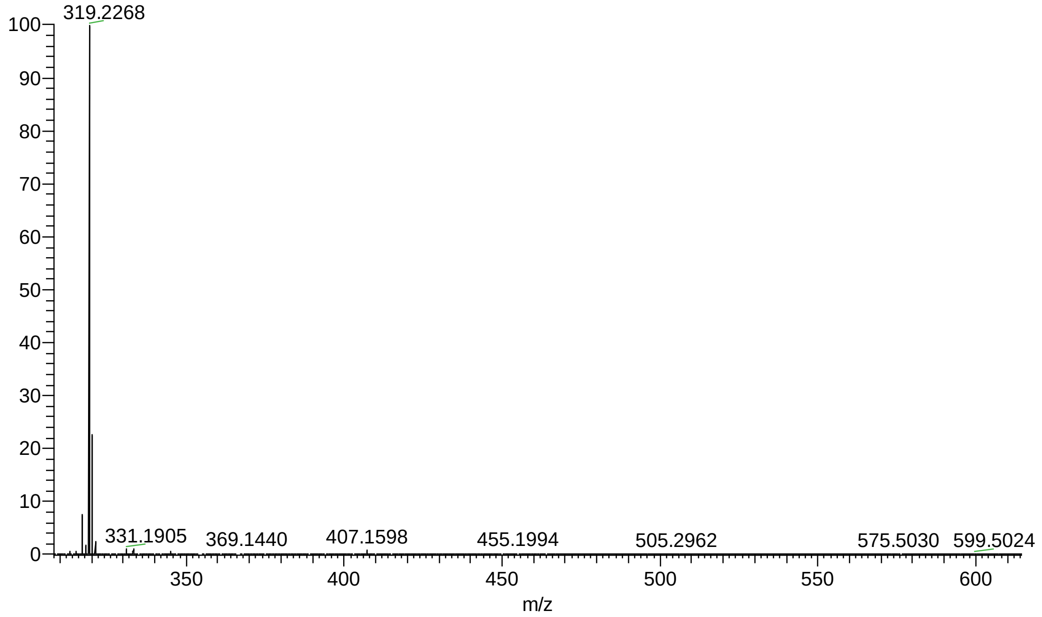

Figure S 10. HRESIMS spectrum of compound 2

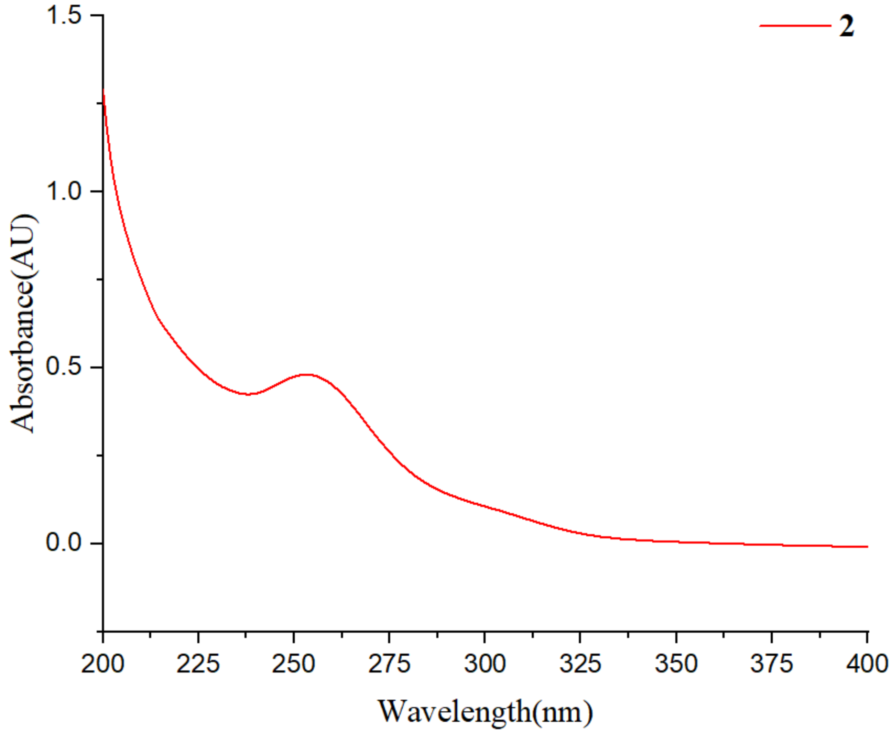

Figure S 11. UV spectrum of compound 2

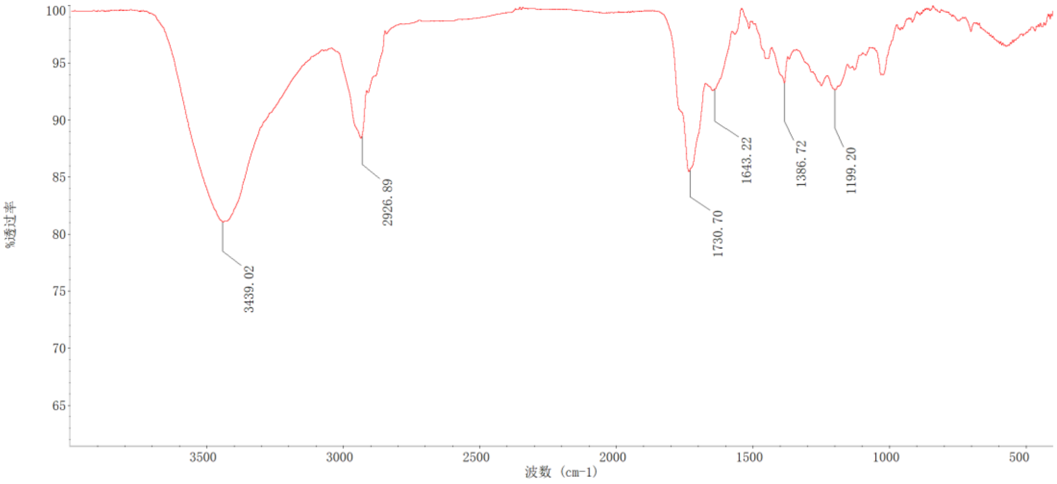

Figure S 12. IR spectrum of compound 2

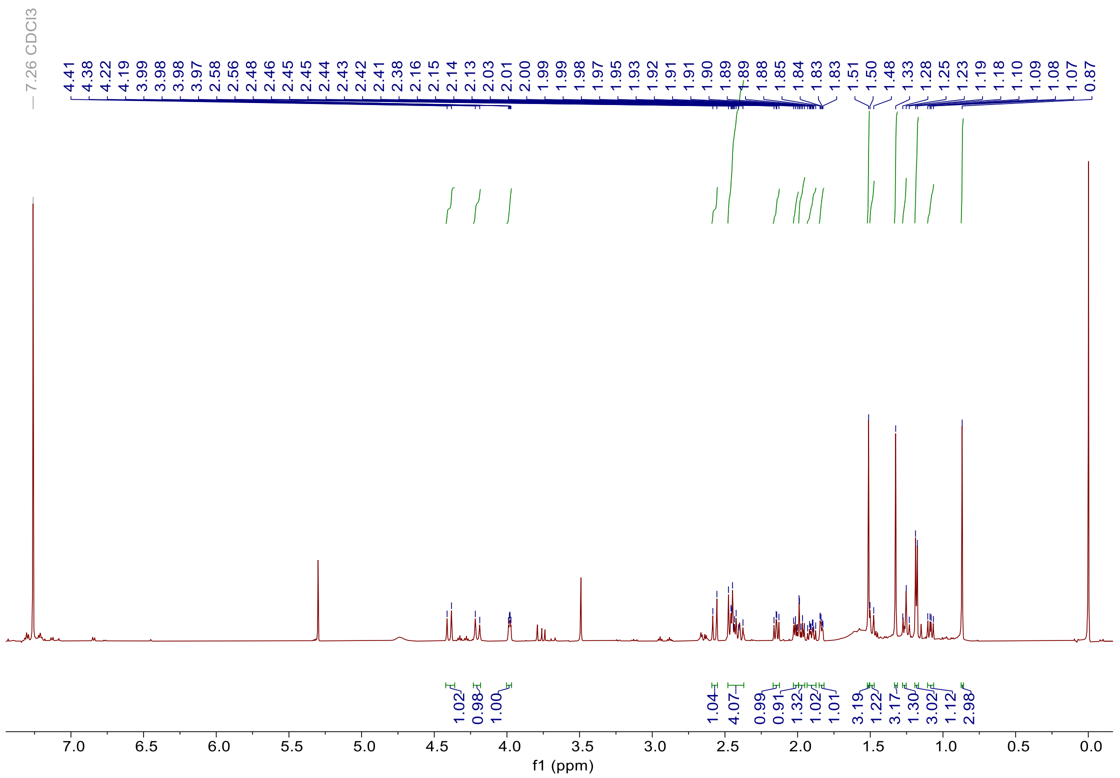

Figure S 13. The ^{1}H NMR spectrum of compound 2 in $CDCl_3$ (600MHz)

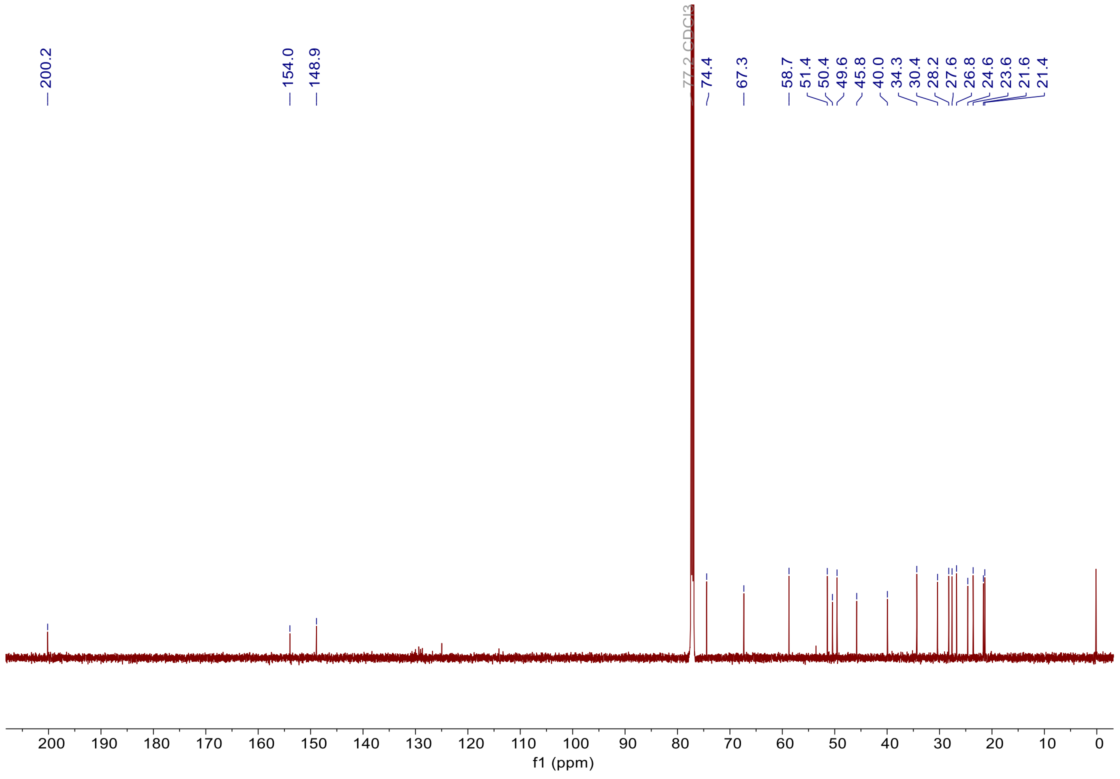

Figure S 14 The ^{13}C NMR spectrum of compound 2 in $CDCl_3$ (150MHz)

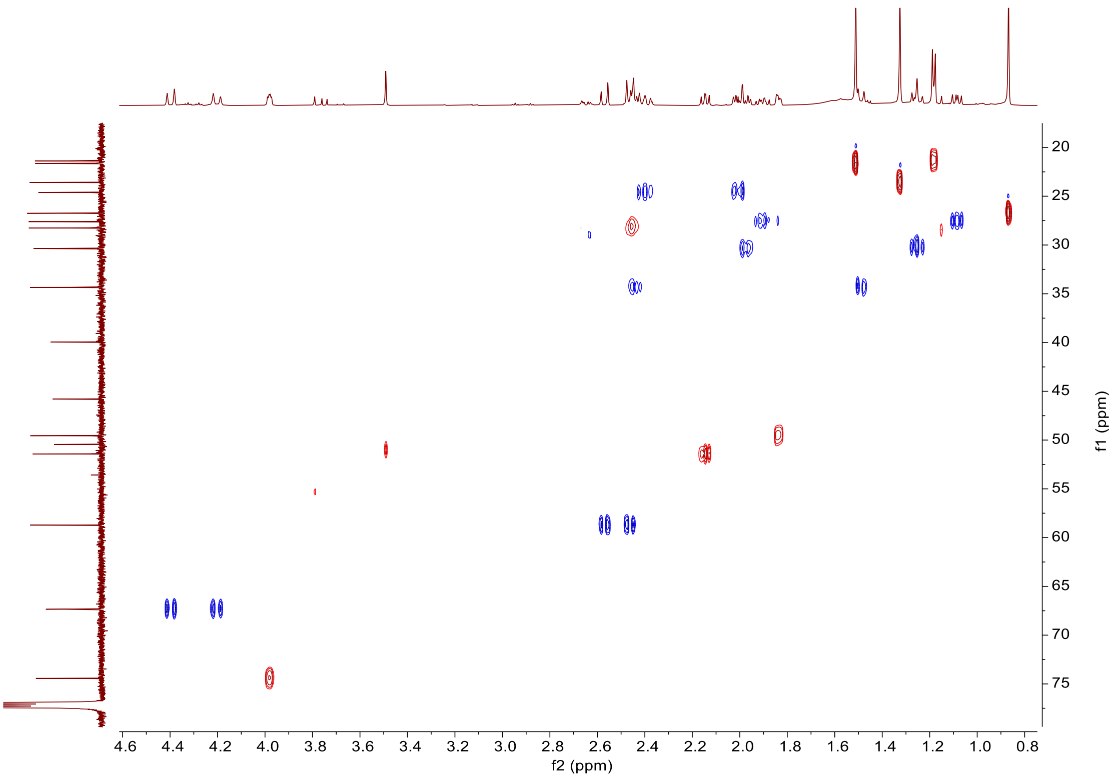

Figure S 15. The HSQC spectrum of compound 2 in $CDCl_3$

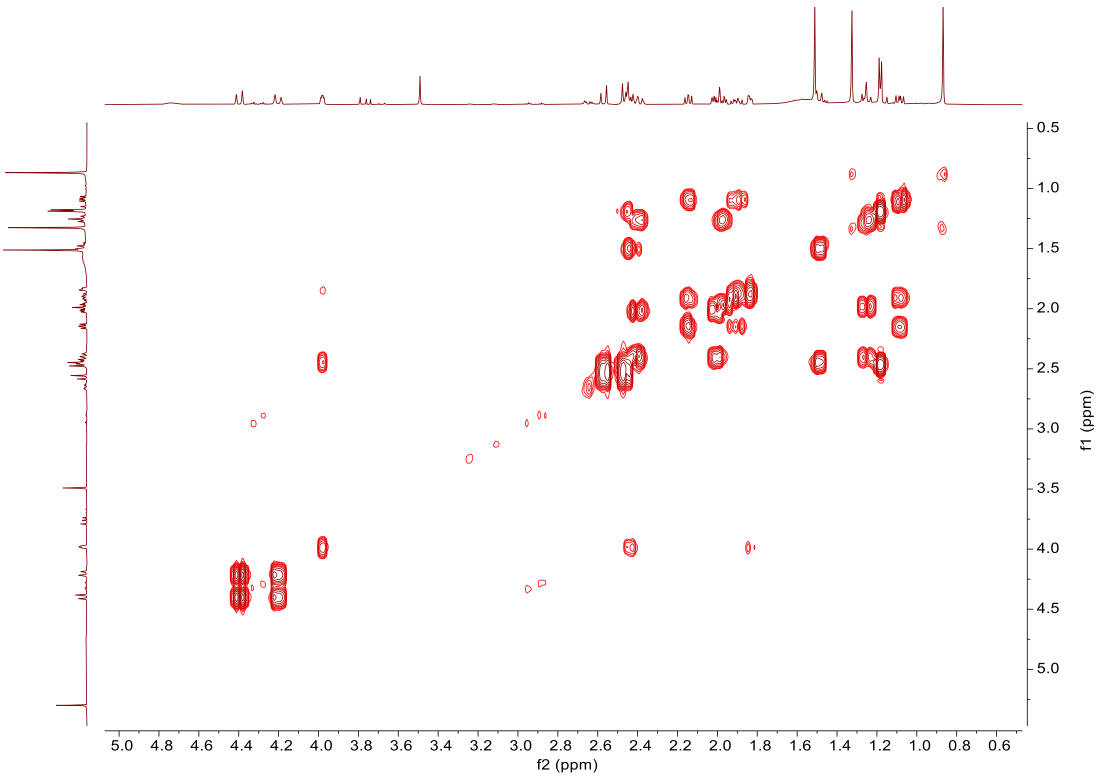

Figure S 16. The ^1H-^1H COSY spectrum of compound 2 in $CDCl_3$

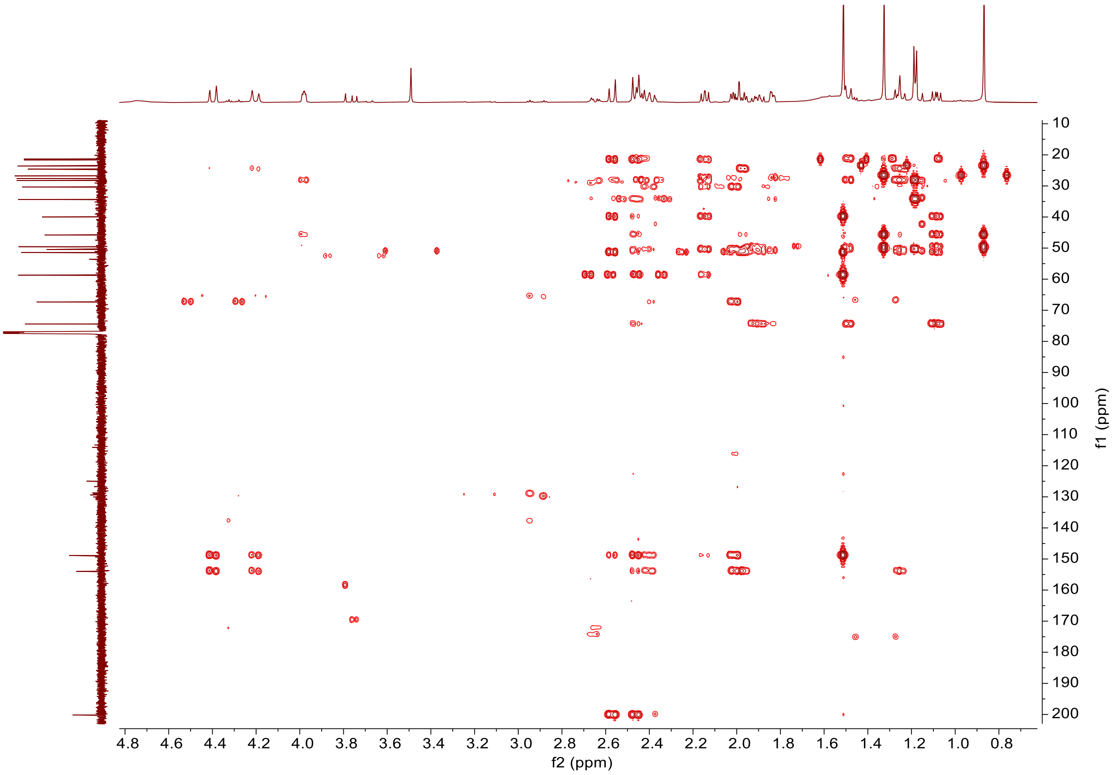

Figure S 17. The HMBC spectrum of compound 2 in $CDCl_3$

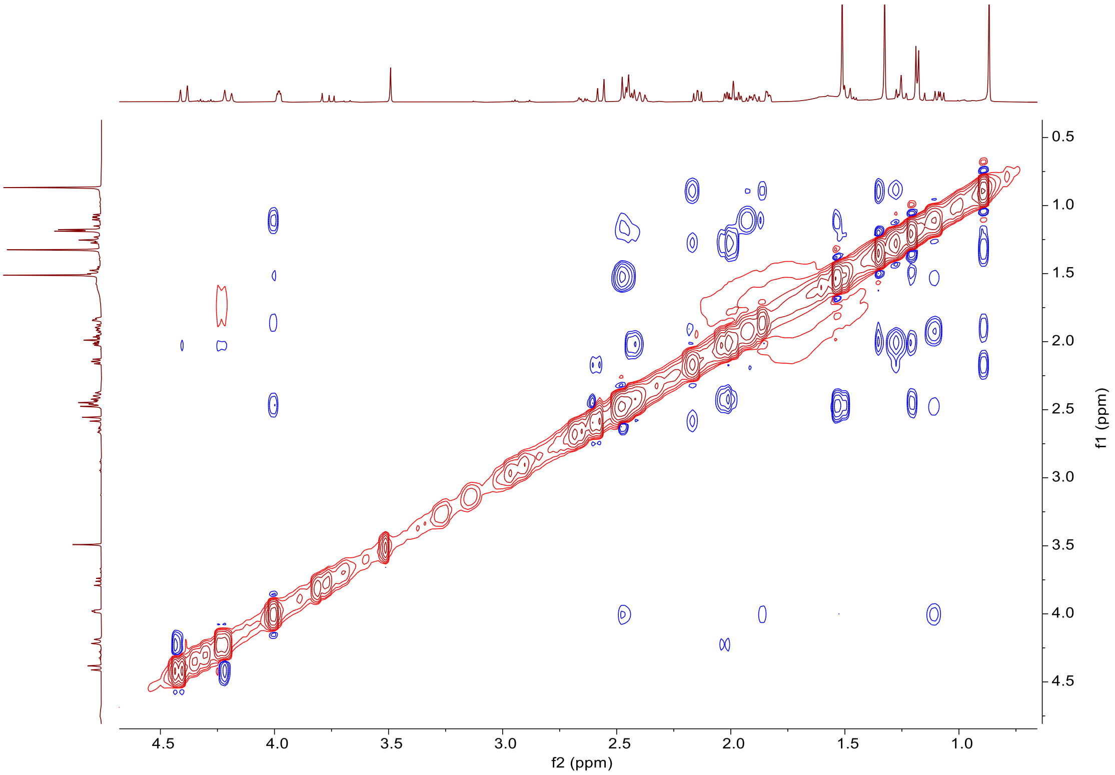

Figure S 18. The NOESY spectrum of compound 2 in $CDCl_3$

.	Component name	Formula	Adducts	Observed m/z	Observed neutral mass (Da)	Neutral mass (Da)	Mass error (ppm)
1	1-1	C14H24O3	+Na	263.1624	240.1732	240.1725	2.6

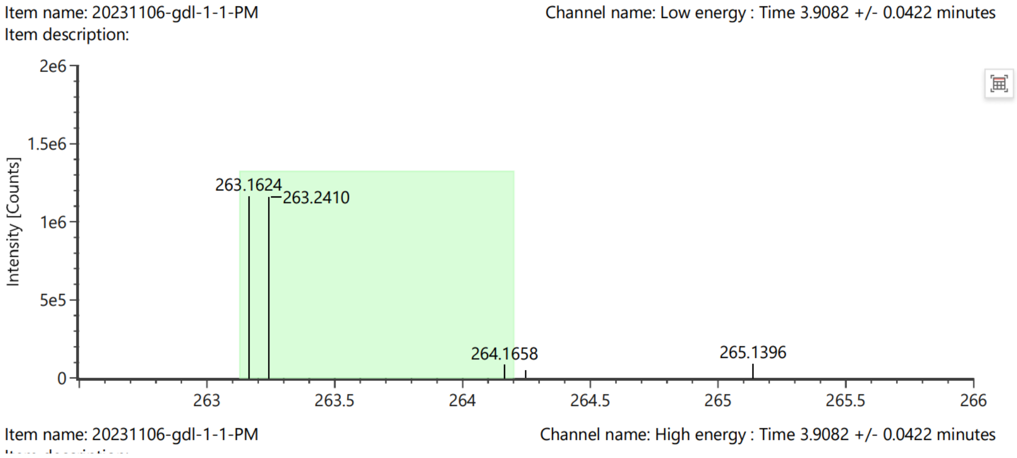

Figure S 19. HRESIMS spectrum of compound 3

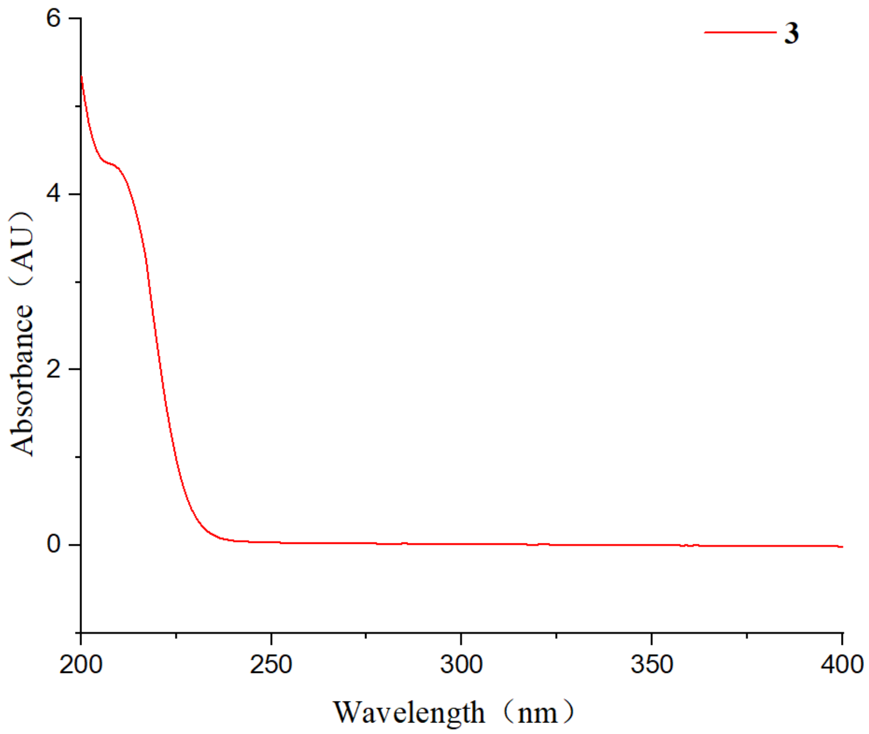

Figure S 20. UV spectrum of compound 3

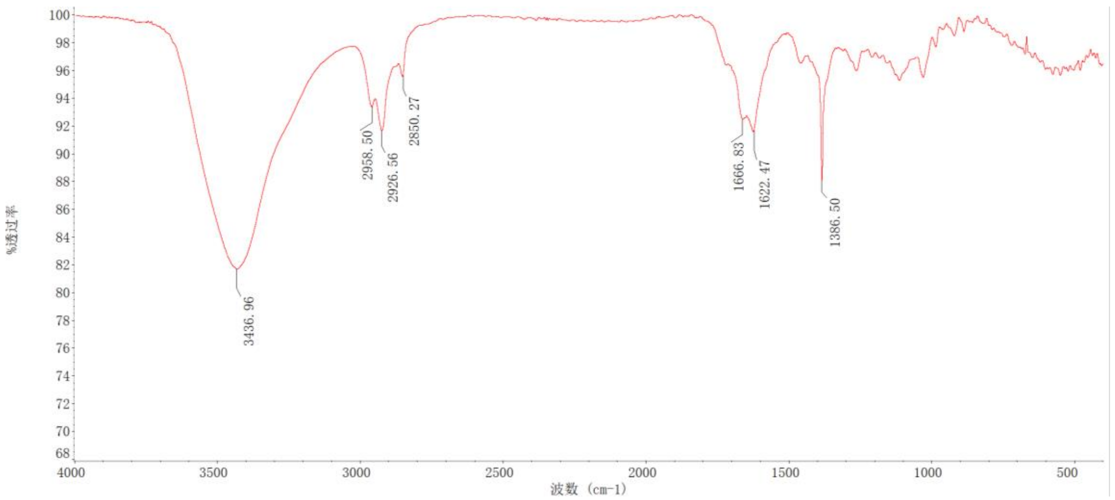

Figure S 21. IR spectrum of compound 3

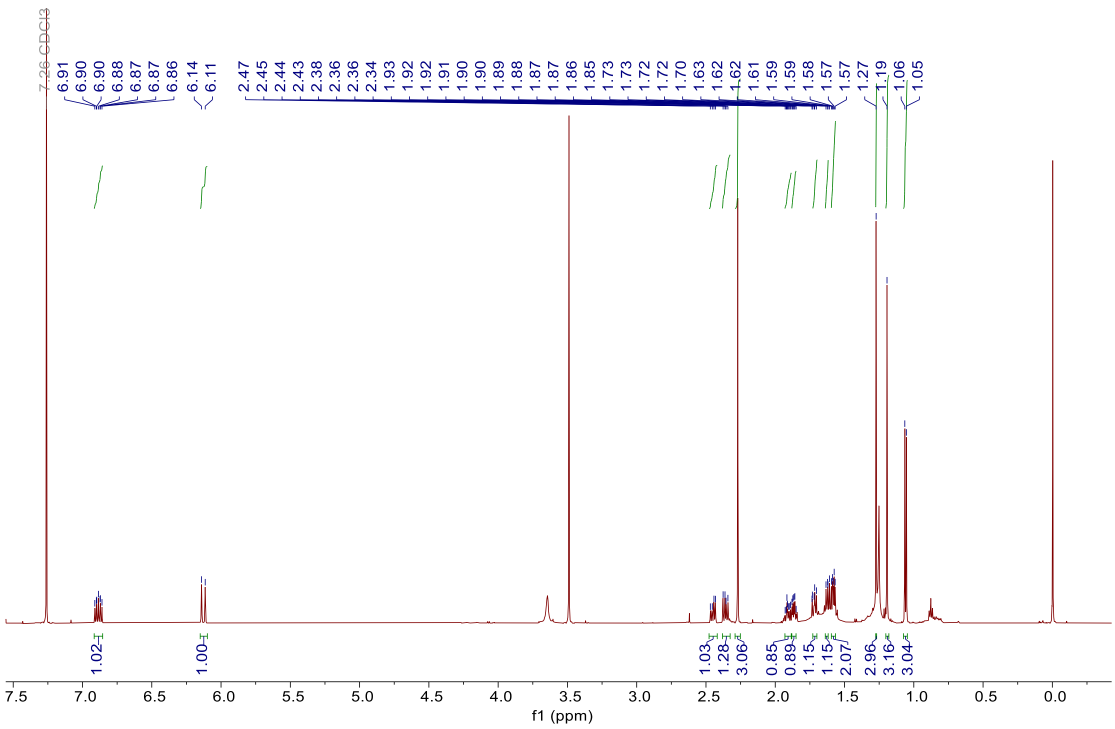

Figure S 22. The ^{1}H NMR spectrum of compound 3 in $CDCl_3$ (600MHz)

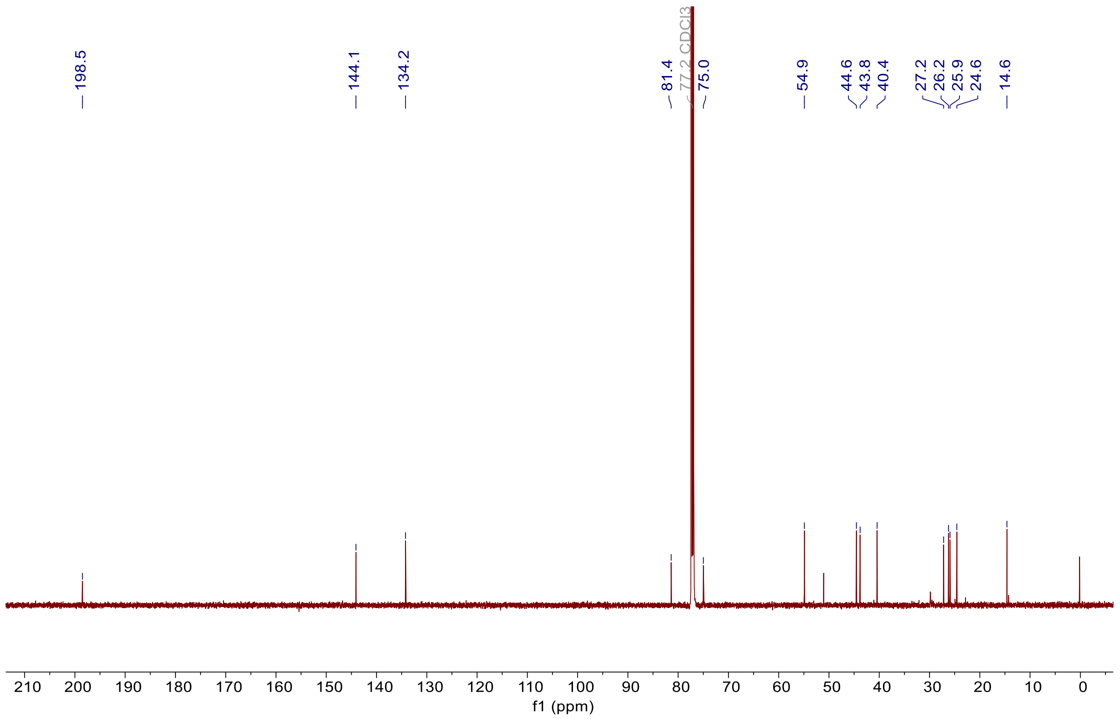

Figure S 23. The ^{13}C NMR spectrum of compound 3 in $CDCl_3$ (150MHz)

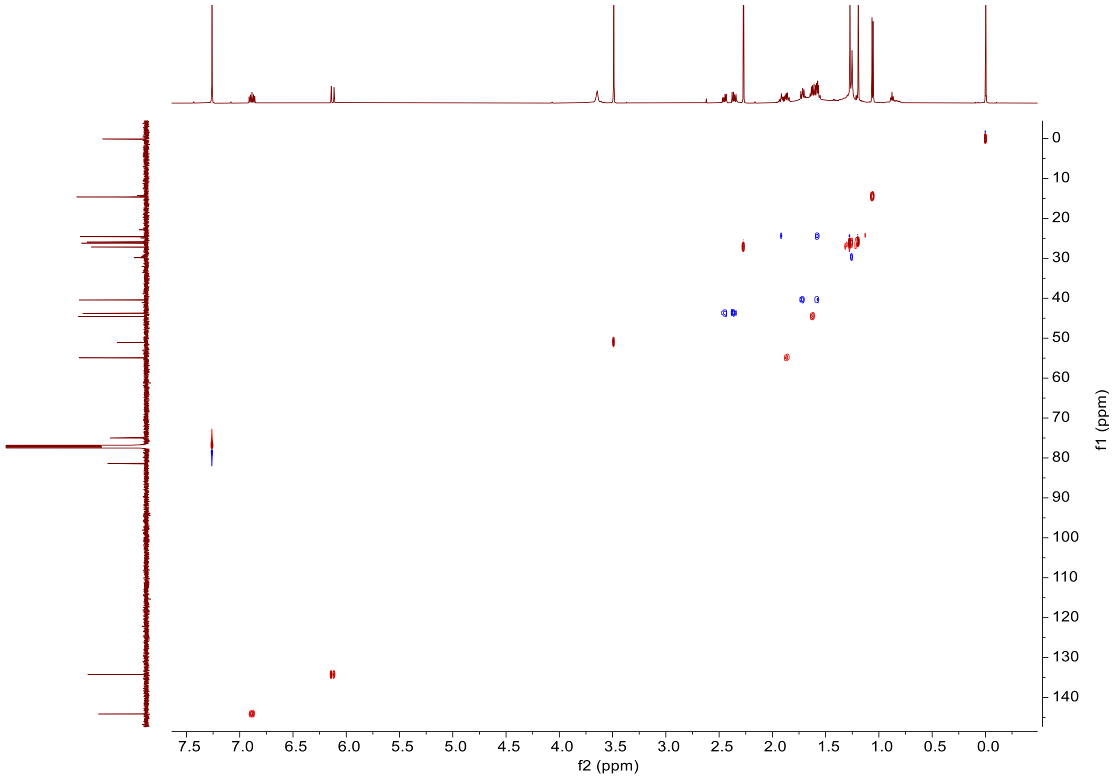

Figure S 24. The HSQC spectrum of compound 3 in $CDCl_3$

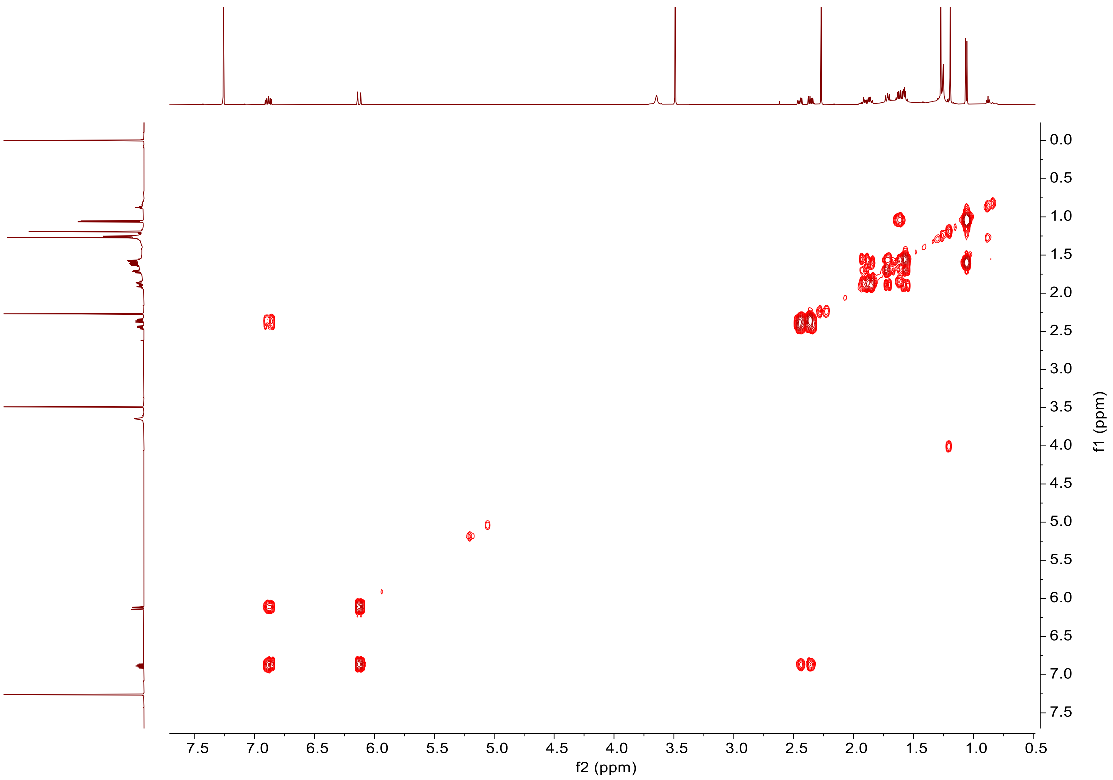

Figure S 25. The ^{1}H-^{1}H COSY spectrum of compound 3 in $CDCl_3$

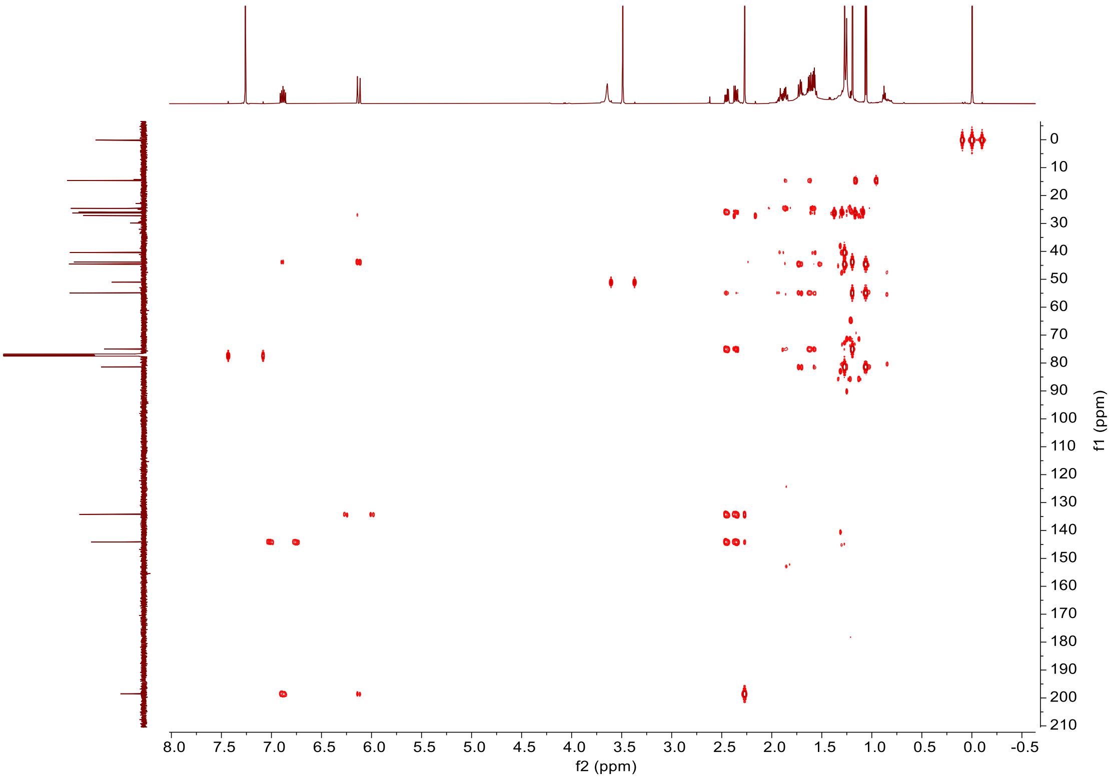

Figure S 26. The HMBC spectrum of compound 3 in $CDCl_3$

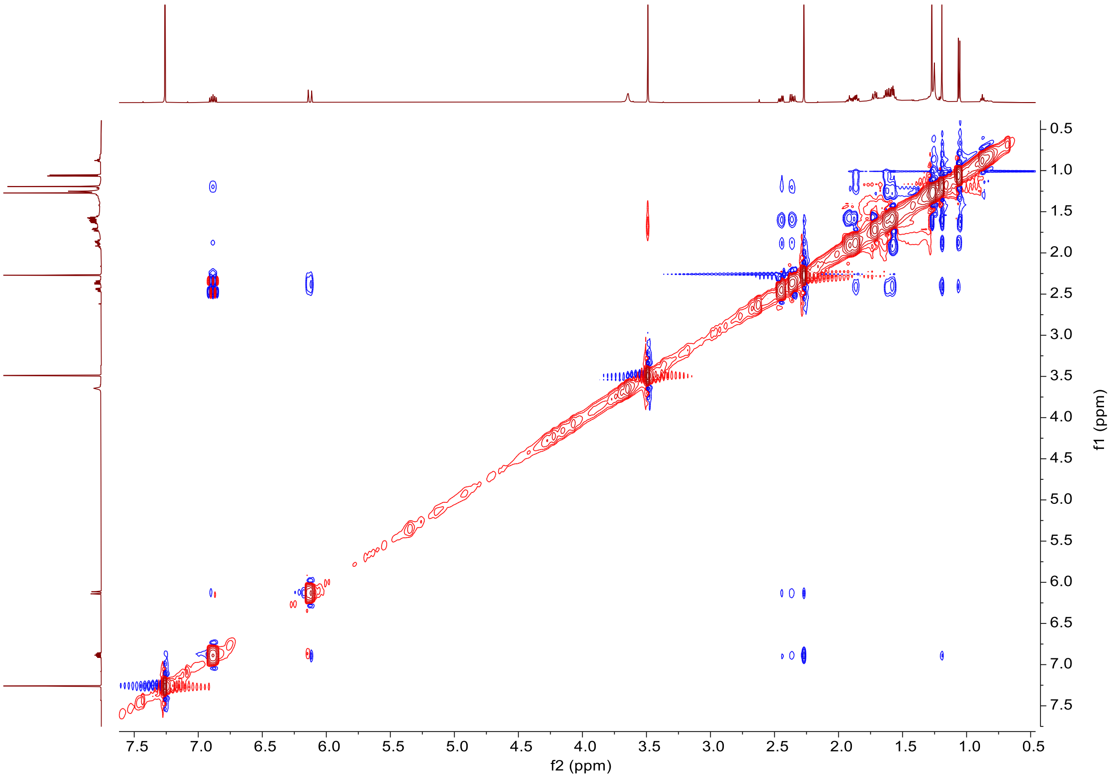

Figure S 27. The NOESY spectrum of compound 3 in $CDCl_3$

.	Component name	Formula	Adducts	Observed m/z	Observed neutral mass (Da)	Neutral mass (Da)	Mass error (ppm)
1	5-1	C15H28O3	+Na	279.1940	256.2048	256.2038	3.3

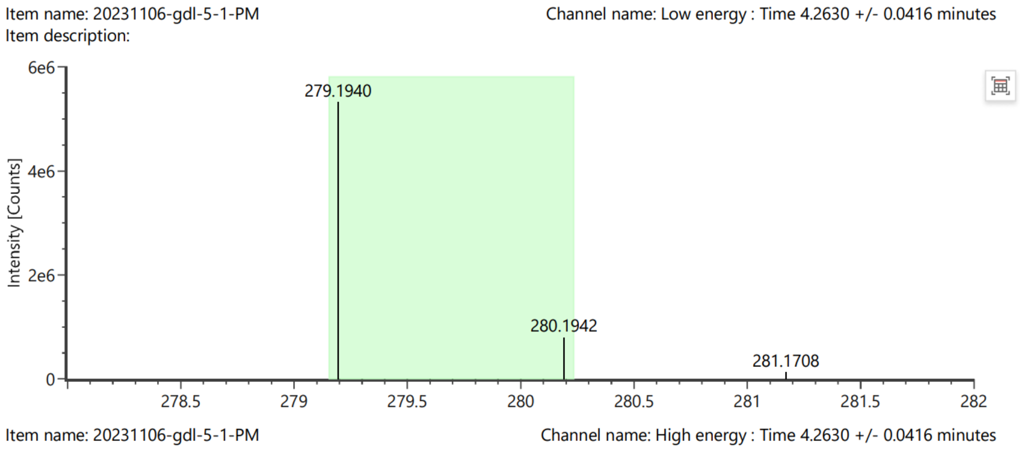

Figure S 28. HRESIMS spectrum of compound 4

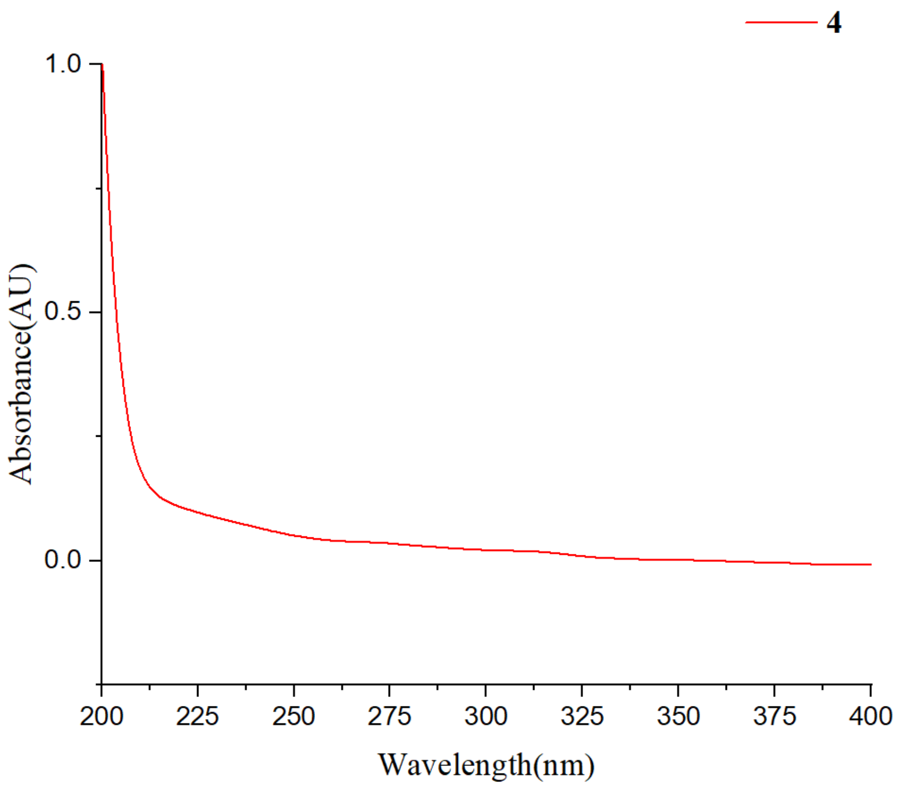

Figure S 29. UV spectrum of compound 4

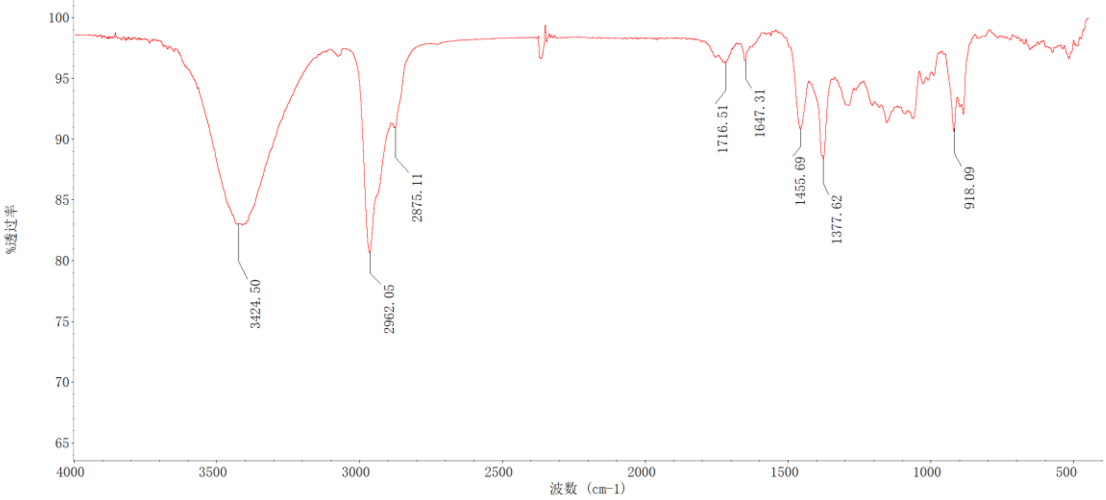

Figure S 30. IR spectrum of compound 4

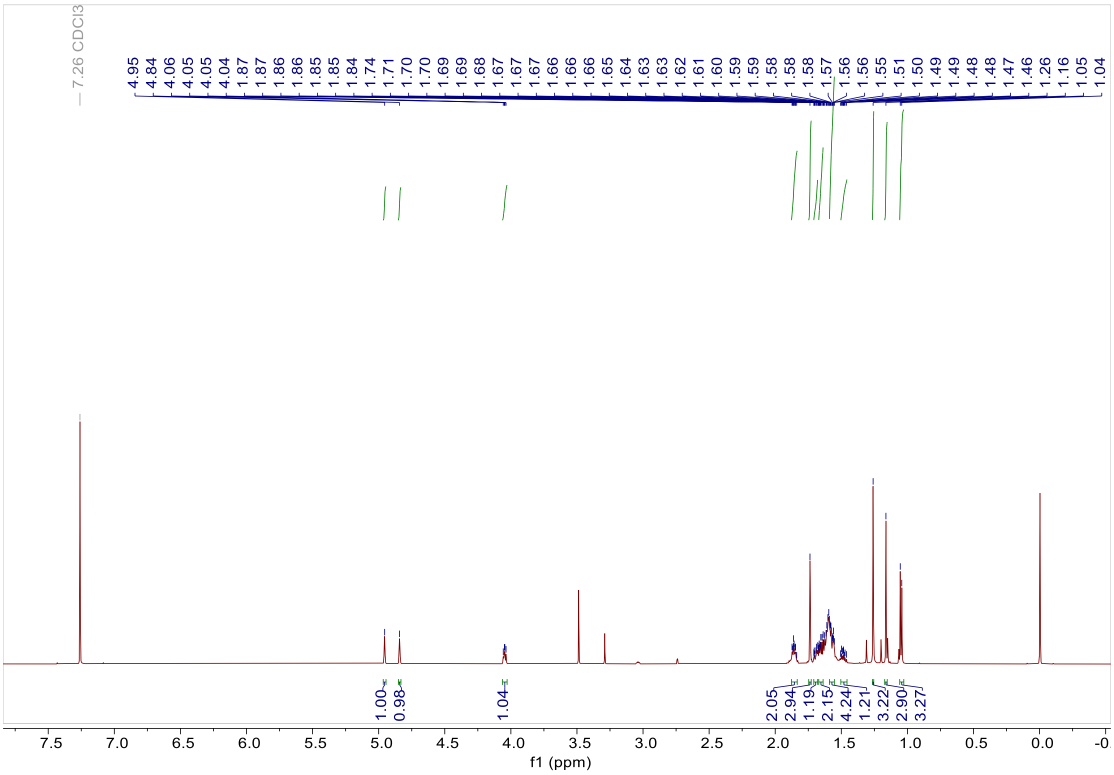

Figure S 31. The ^{1}H NMR spectrum of compound 4 in $CDCl_3$ (600MHz)

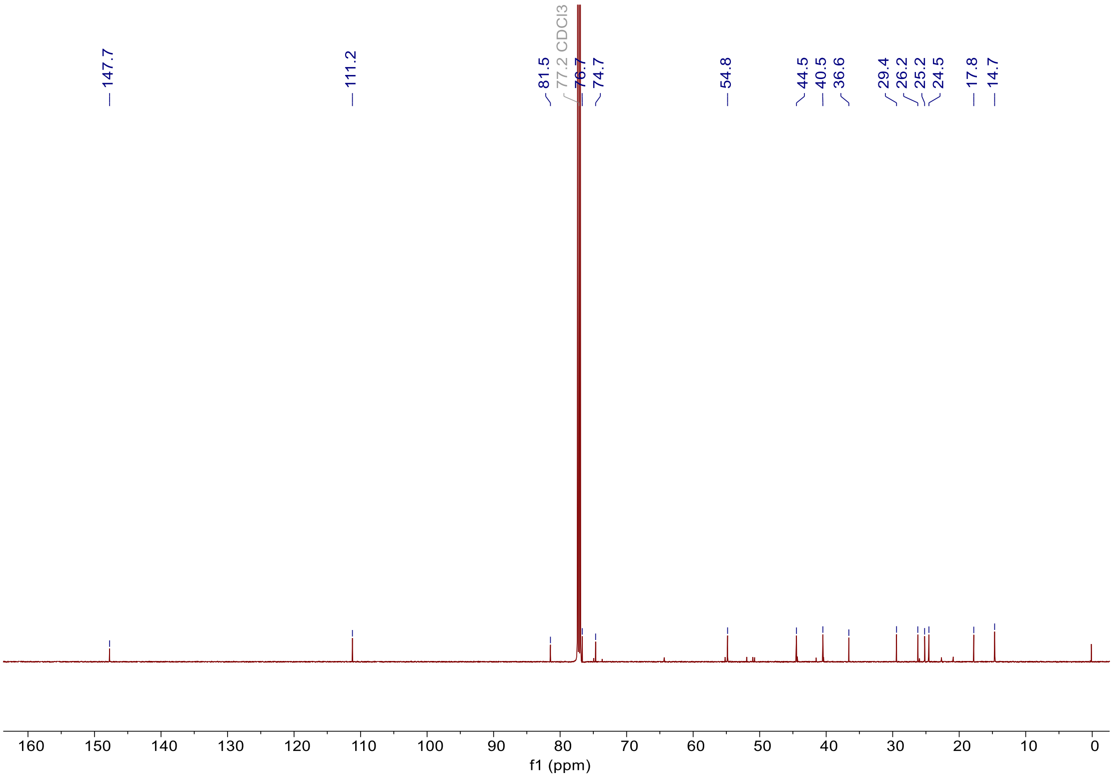

Figure S 32. The ^{13}C NMR spectrum of compound 4 in $CDCl_3$ (150MHz)

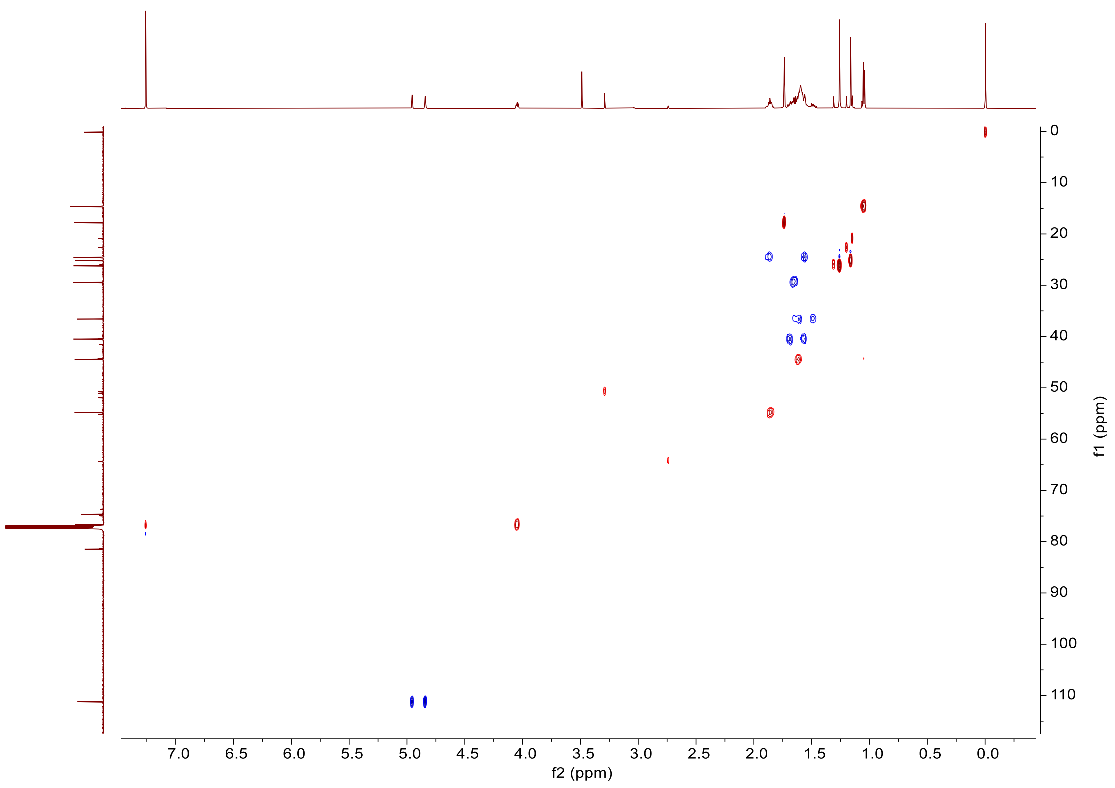

Figure S 33. The HSQC spectrum of compound 4 in $CDCl_3$

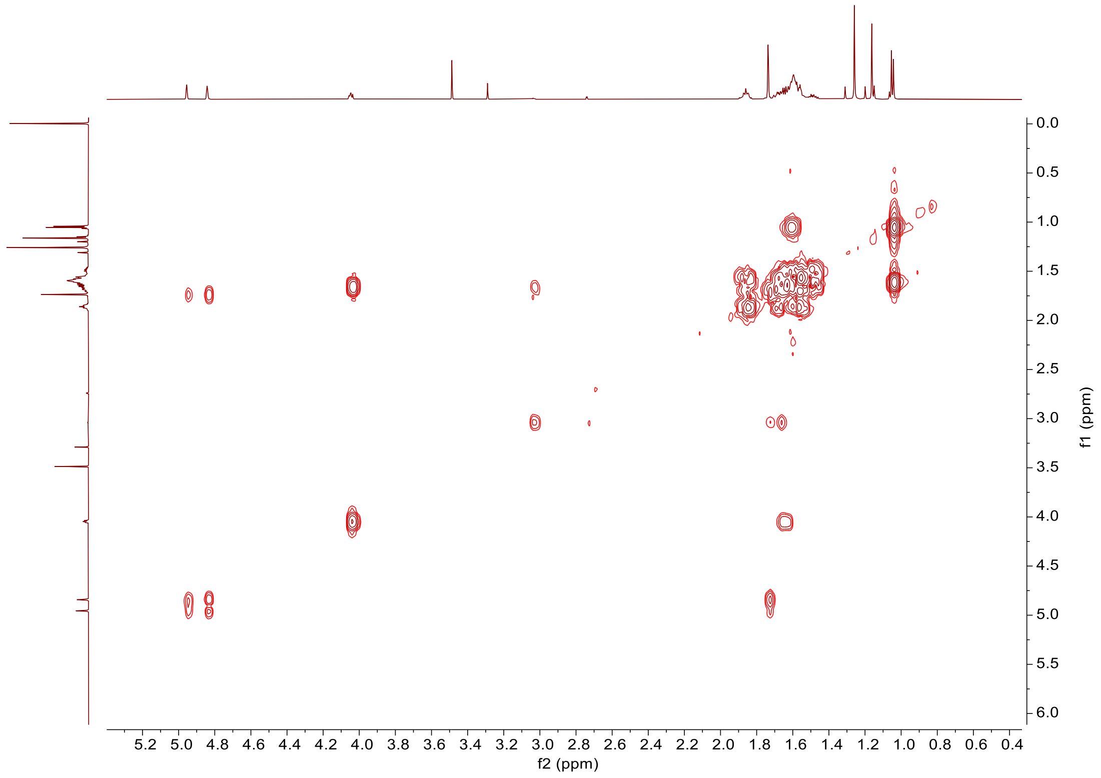

Figure S 34. The ^1H-^1H COSY spectrum of compound 4 in $CDCl_3$

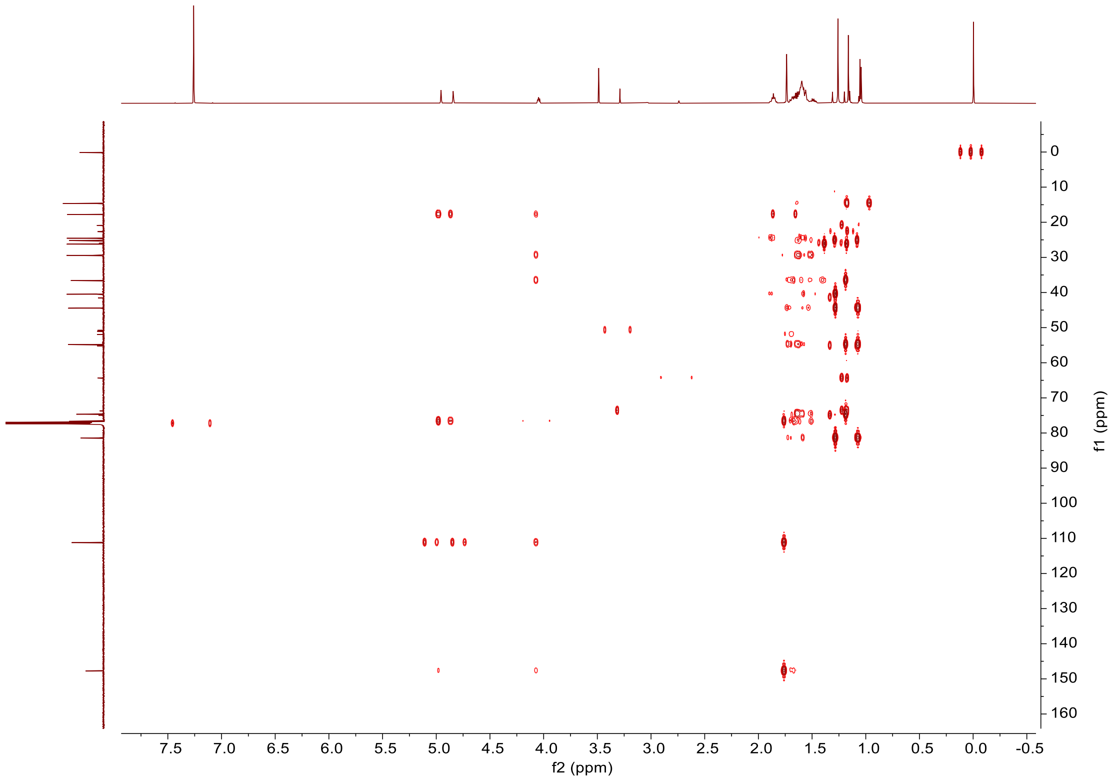

Figure S 35. The HMBC spectrum of compound 4 in $CDCl_3$

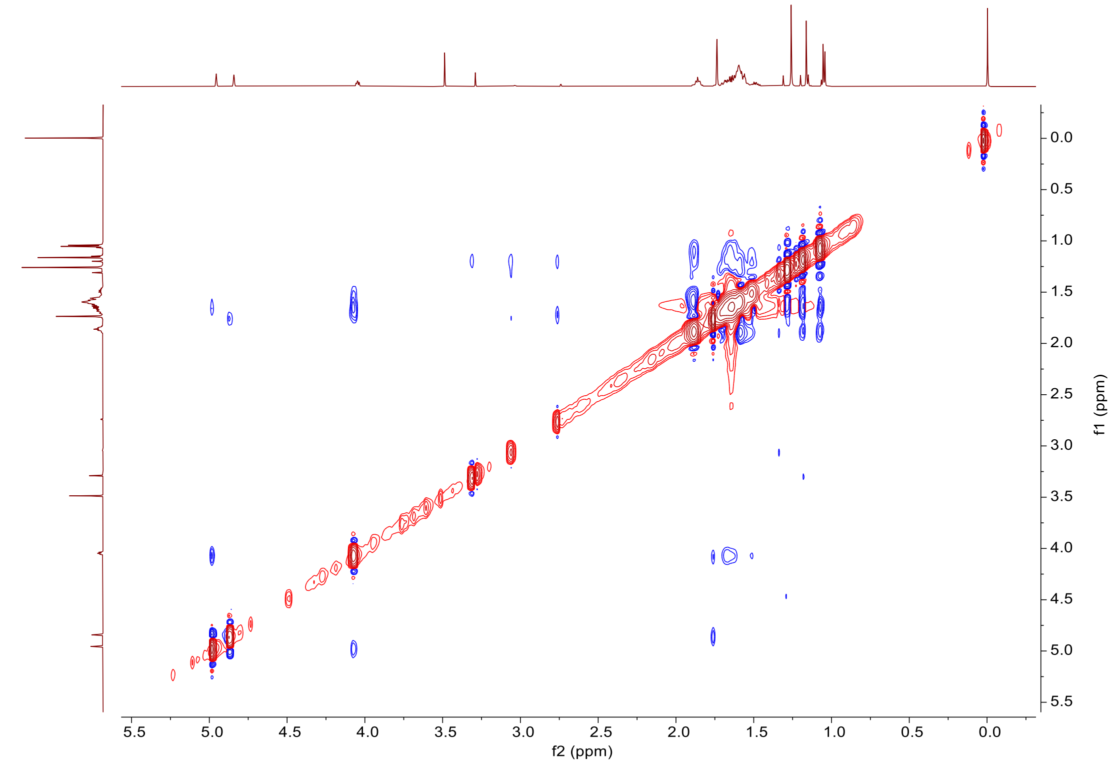

Figure S 36. The NOESY spectrum of compound 4 in $CDCl_3$

.	Component name	Formula	Adducts	Observed m/z	Observed neutral mass (Da)	Neutral mass (Da)	Mass error (ppm)
1	6-2	C15H28O4	-H	271.1910	272.1983	272.1988	-1.9

Item name: 20231016-GDL-6-2-NM   Channel name: Low energy : Time 6.2447 +/- 0.0500 minutes

Item description:

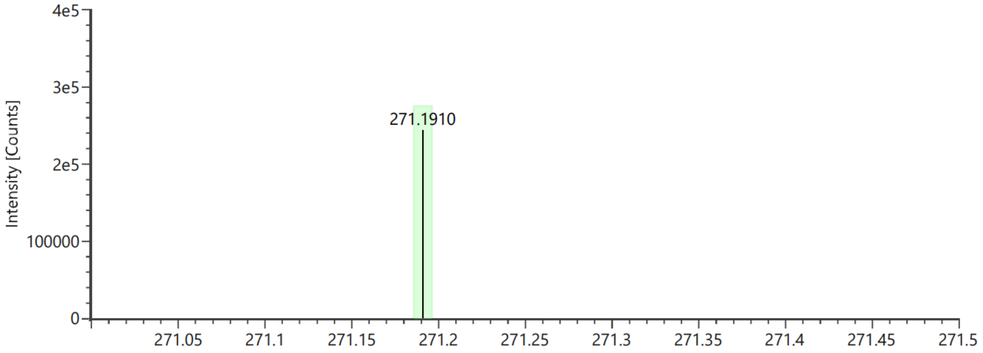

Item name: 20231016-GDL-6-2-NM   Channel name: High energy : Time 6.2447 +/- 0.0500 minutes

Figure S 37. HRESIMS spectrum of compound 5

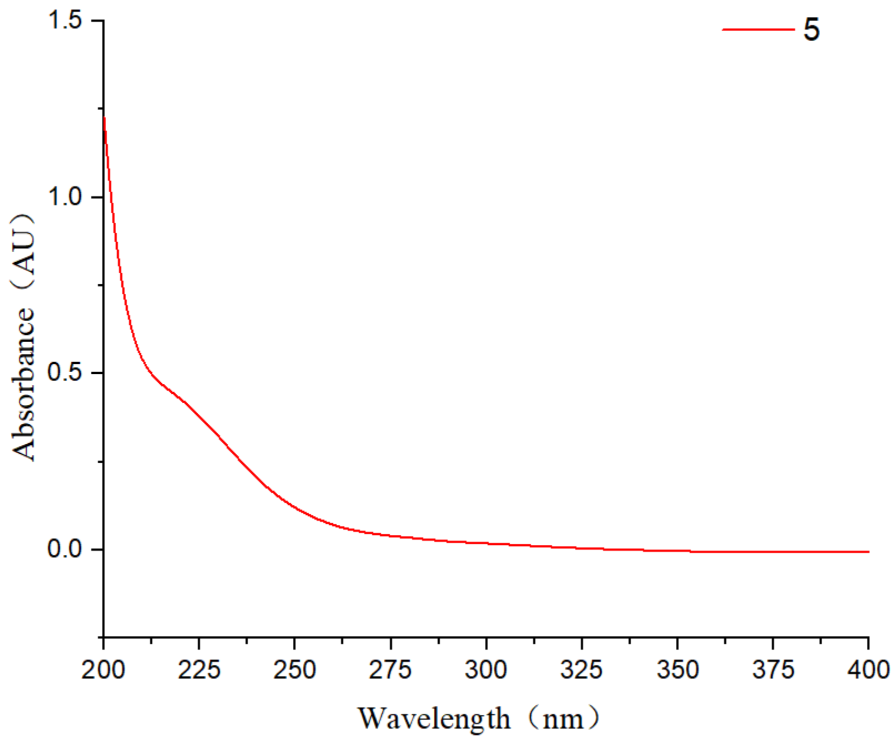

Figure S 38. UV spectrum of compound 5

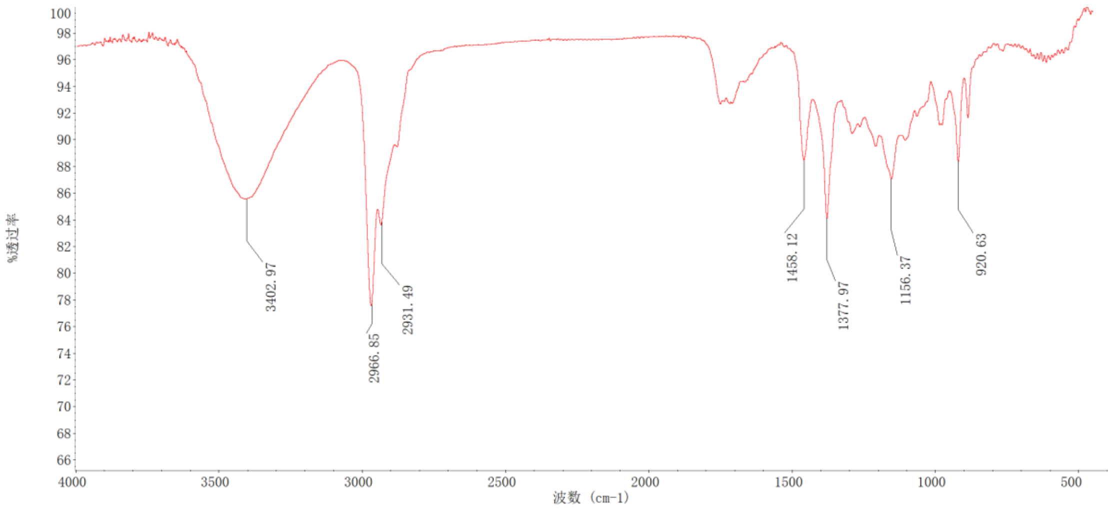

Figure S 39. IR spectrum of compound 5

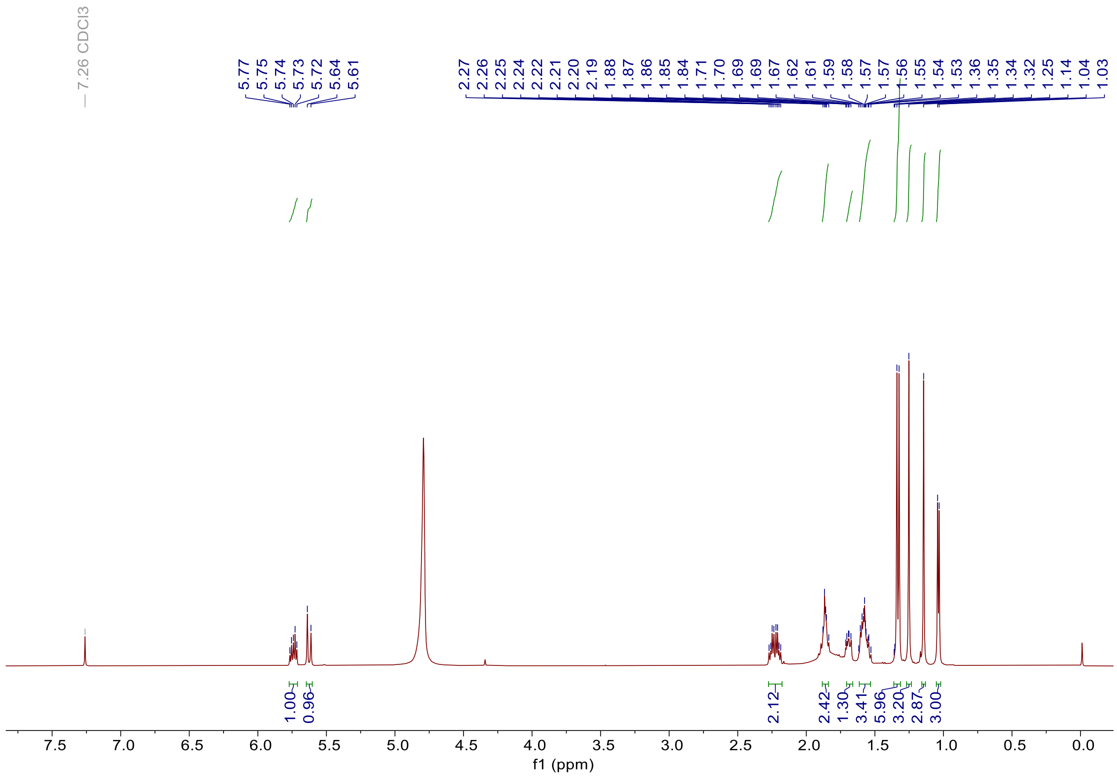

Figure S 40. The ^{1}H NMR spectrum of compound 5 in $CDCl_3$ (600MHz)

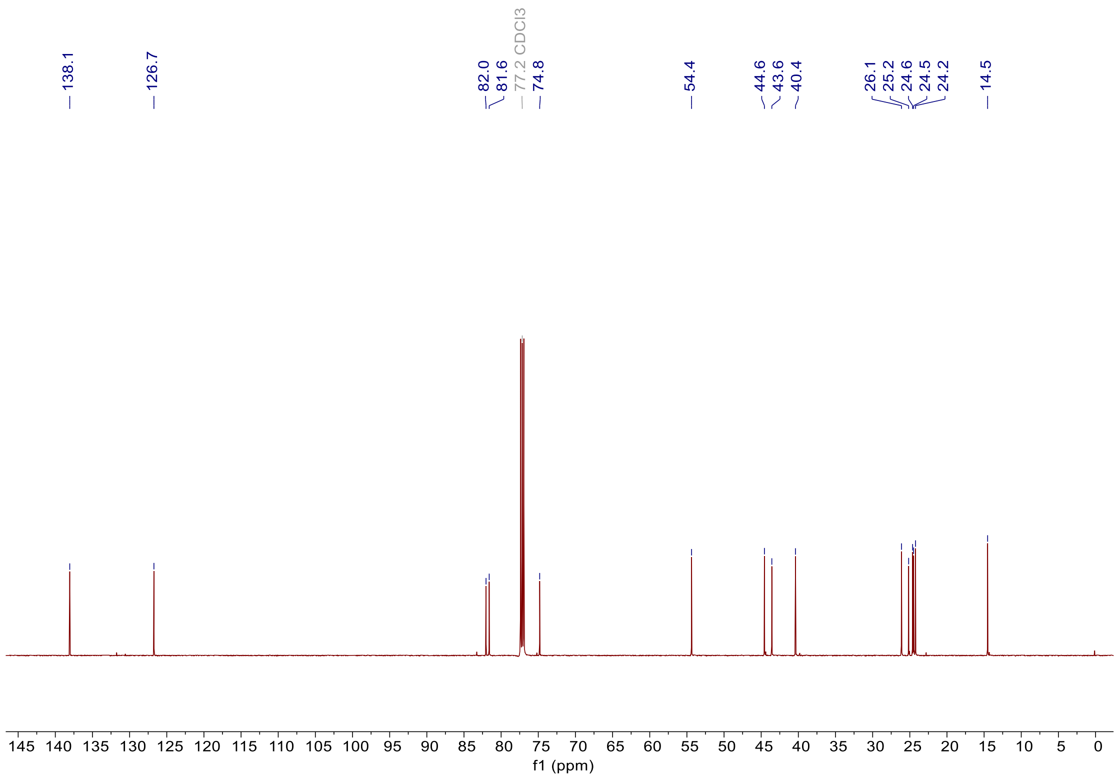

Figure S 41. The ^{13}C NMR spectrum of compound 5 in $CDCl_3$ (150MHz)

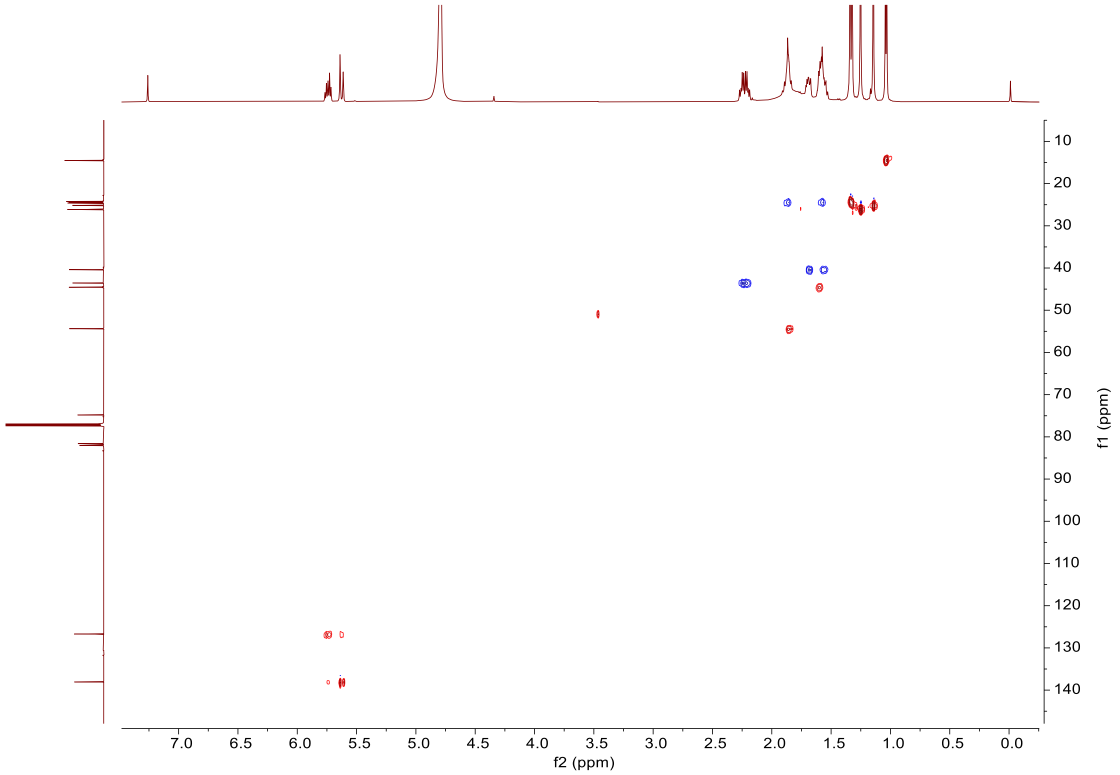

Figure S 42. The HSQC spectrum of compound 5 in $CDCl_3$

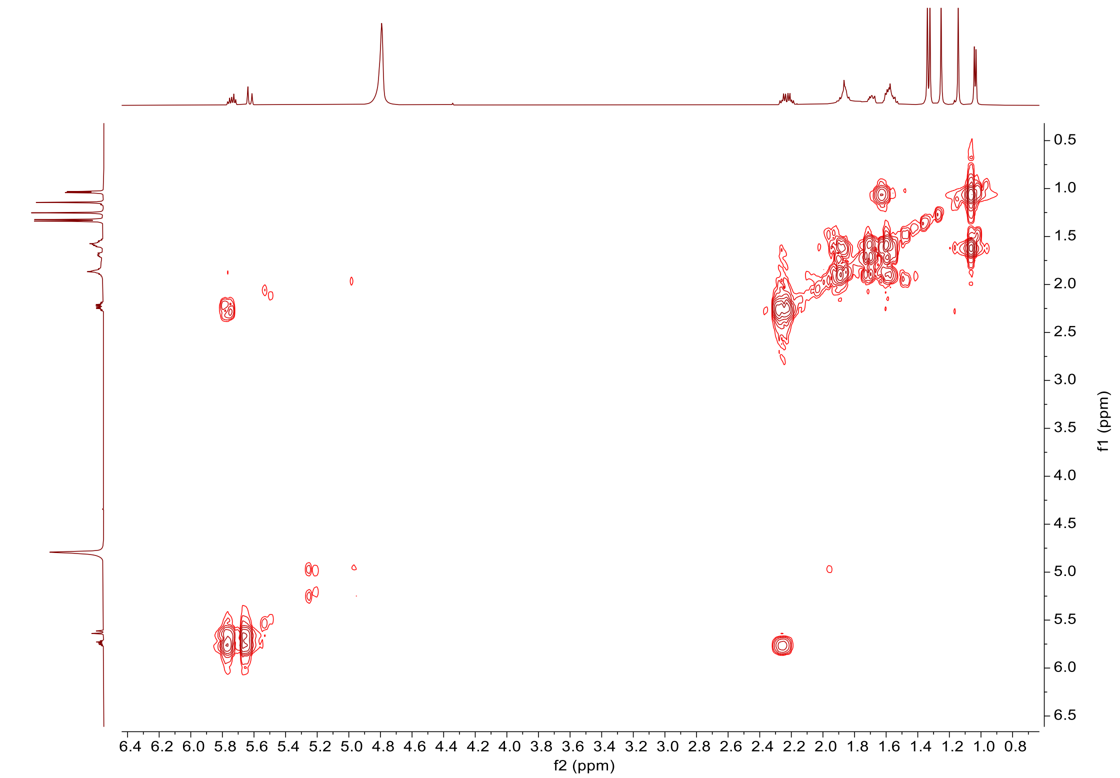

Figure S 43. The ^{1}H-^{1}H COSY spectrum of compound 5 in $CDCl_3$

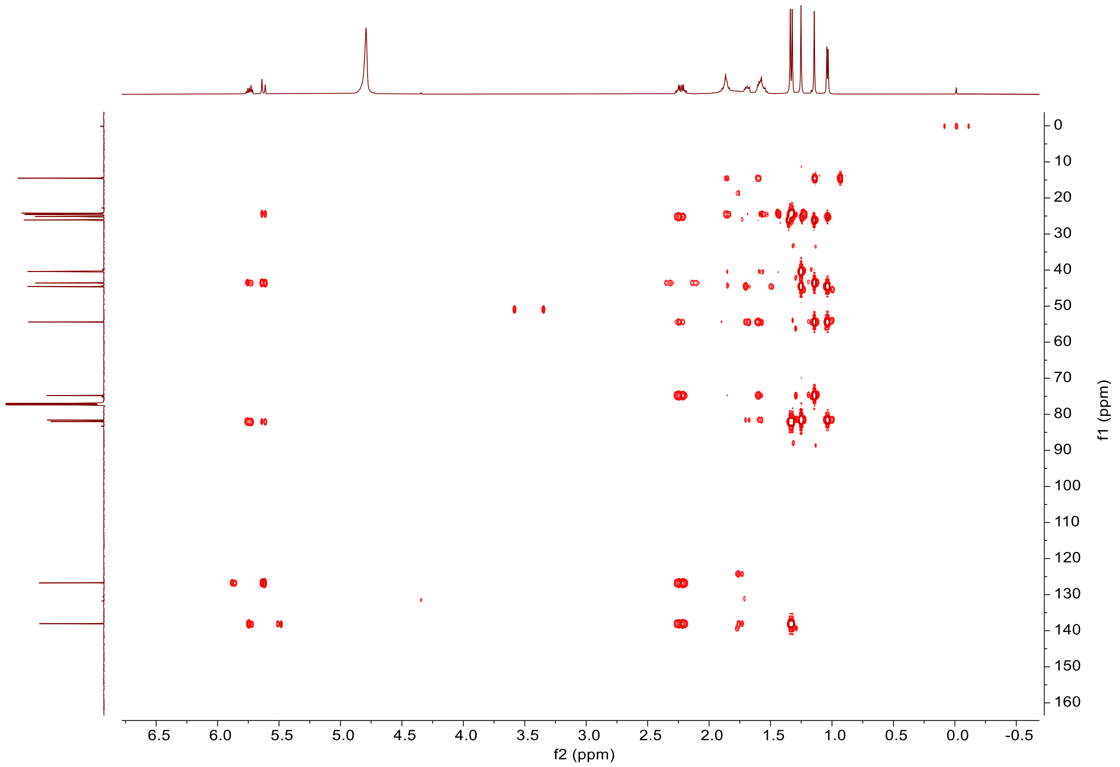

Figure S 44. The HMBC spectrum of compound 5 in $CDCl_3$

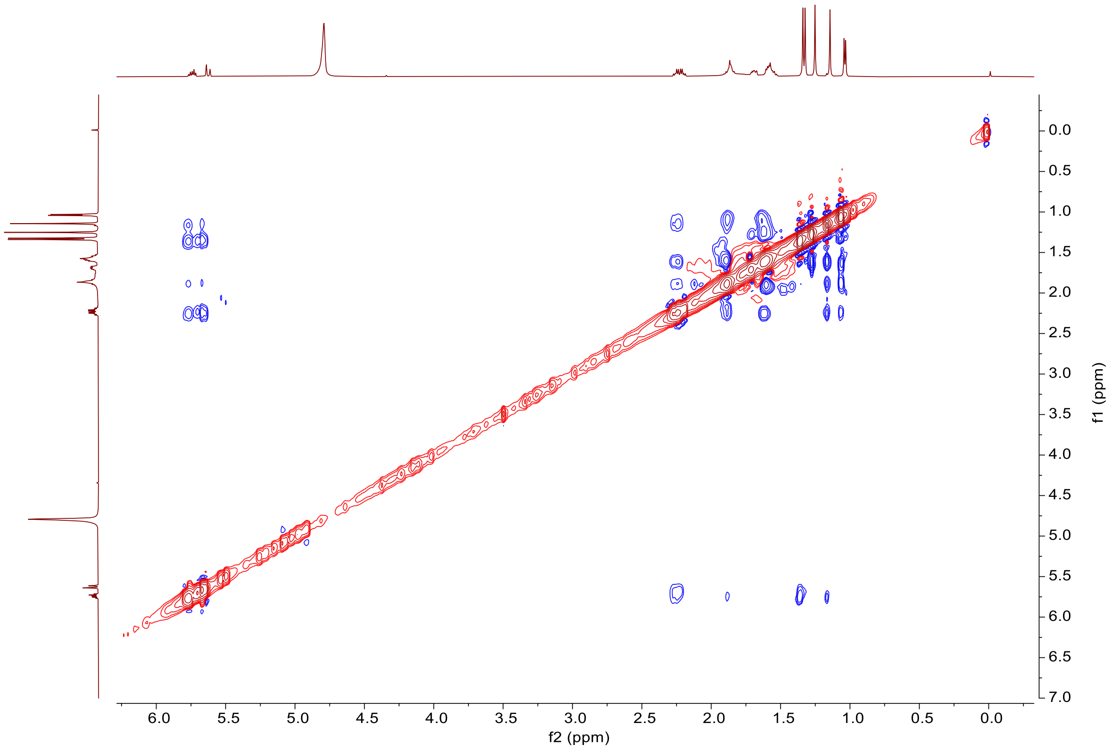

Figure S 45. The NOESY spectrum of compound 5 in $CDCl_3$

.	Component name	Formula	Adducts	Observed m/z	Observed neutral mass (Da)	Neutral mass (Da)	Mass error (ppm)
1	10-4	C20H34O6	+Na	393.2249	370.2357	370.2355	0.4

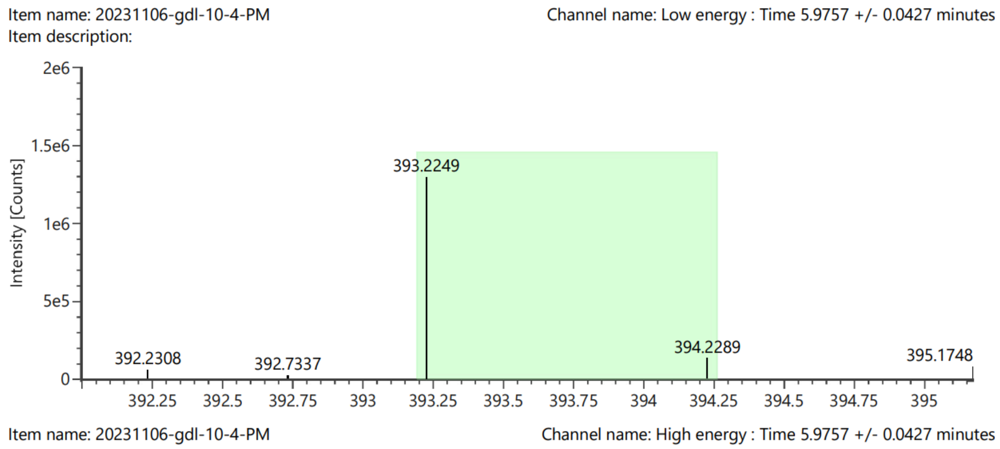

Figure S 46. HRESIMS spectrum of compound 6

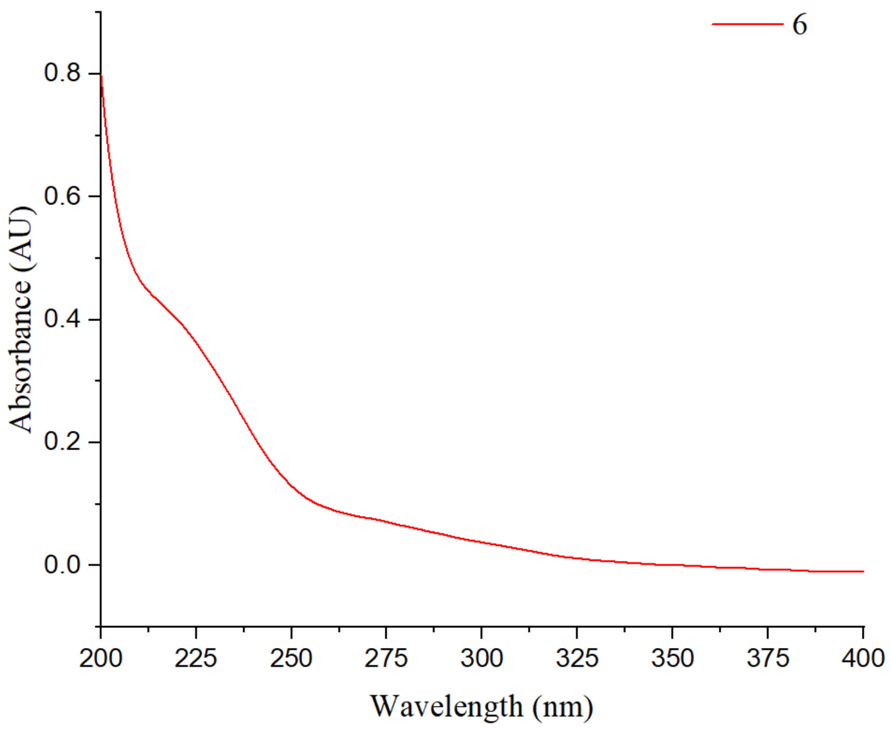

Figure S 47. UV spectrum of compound 6

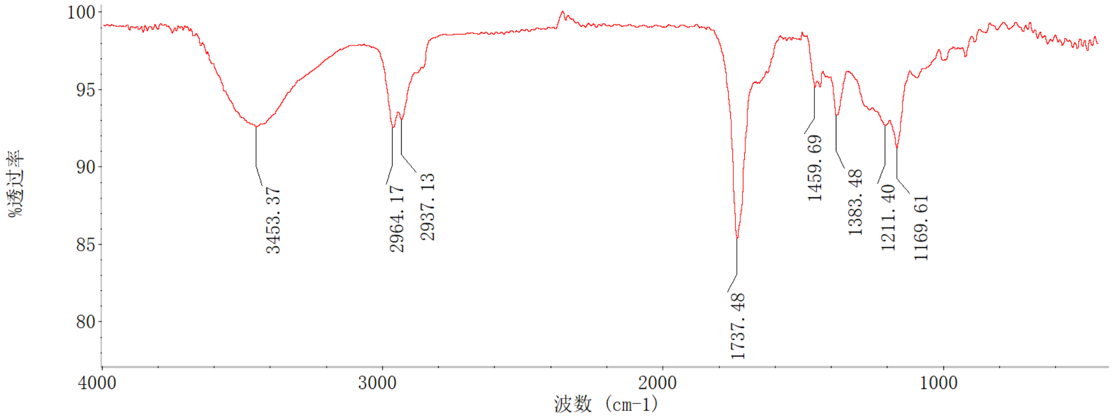

Figure S 48. IR spectrum of compound 6

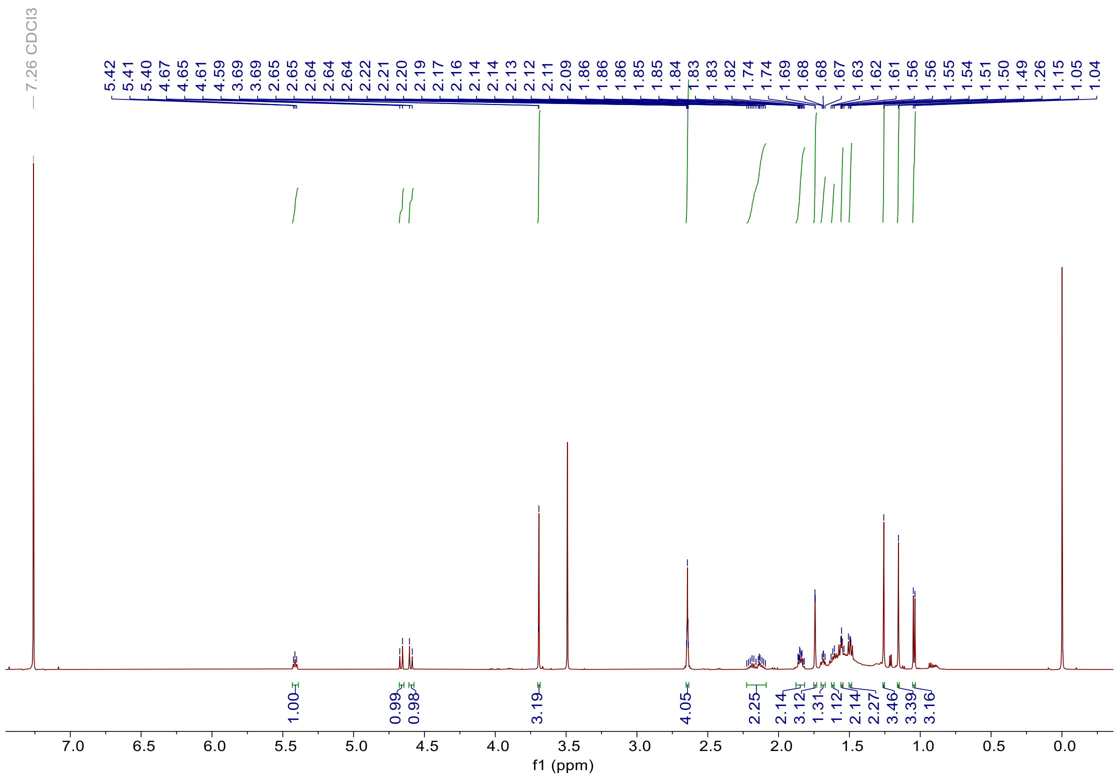

Figure S 49. The ^{1}H NMR spectrum of compound 6 in $CDCl_3$ (600MHz)

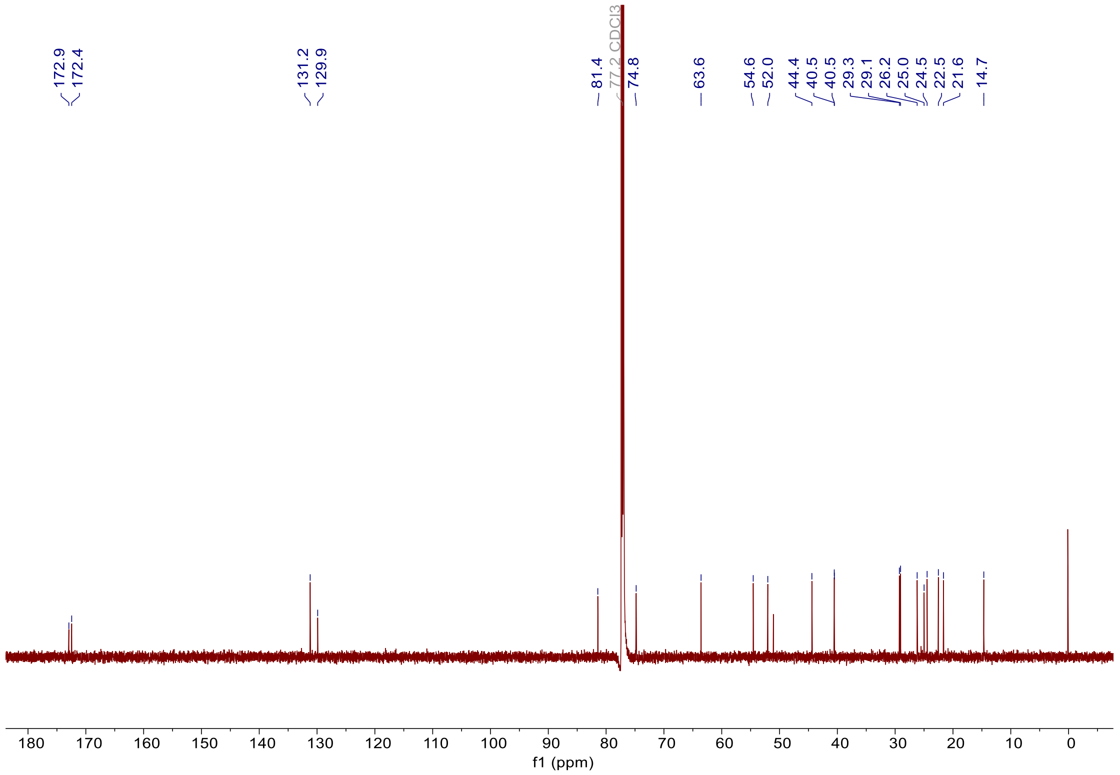

Figure S 50. The ^{13}C NMR spectrum of compound 6 in $CDCl_3$ (150MHz)

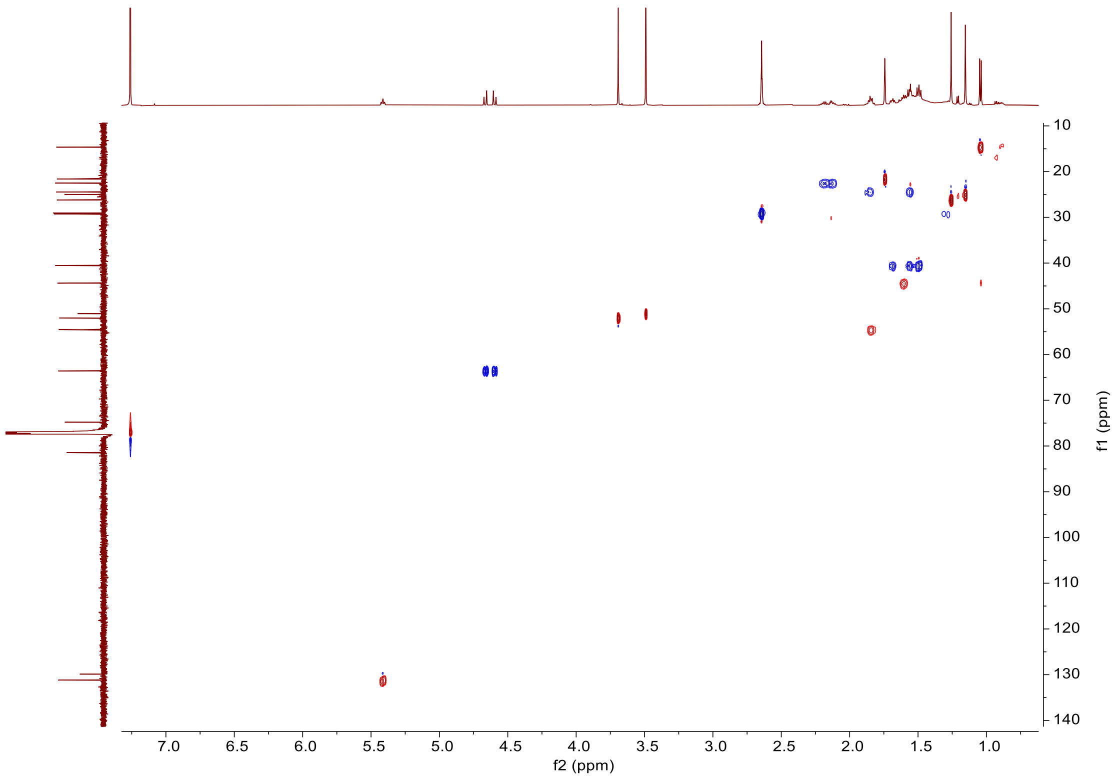

Figure S 51. The HSQC spectrum of compound 6 in $CDCl_3$

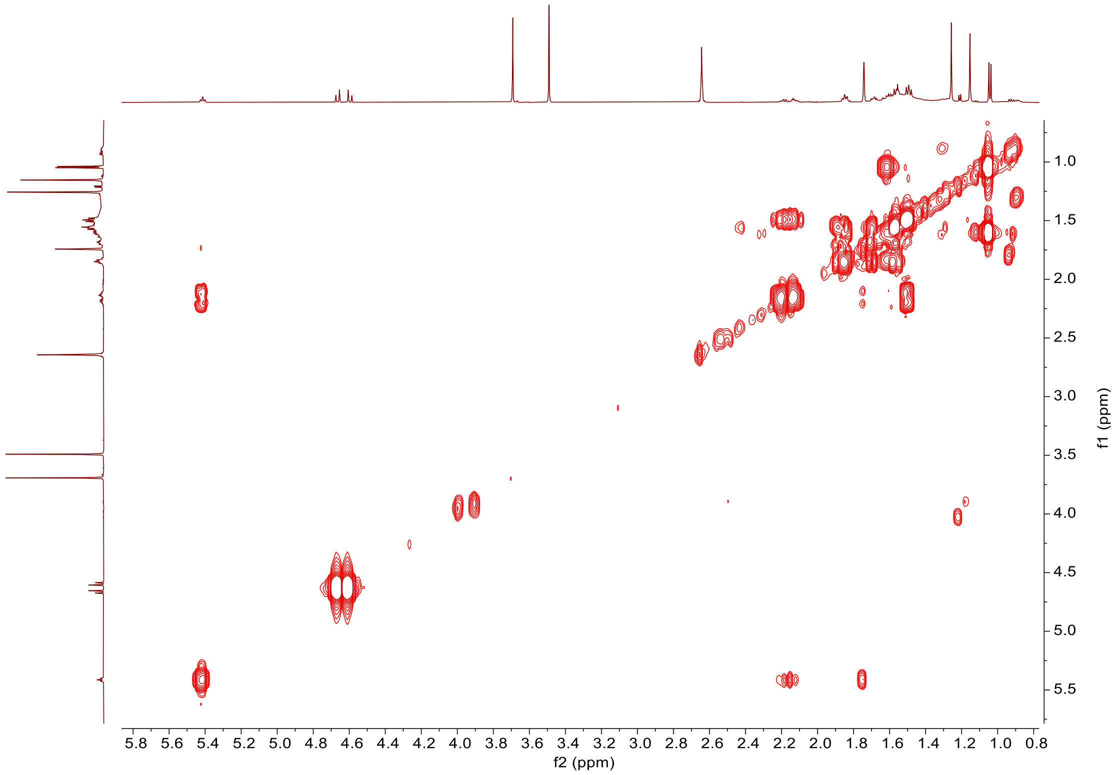

Figure S 52. The ^1H-^1H COSY spectrum of compound 6 in $CDCl_3$

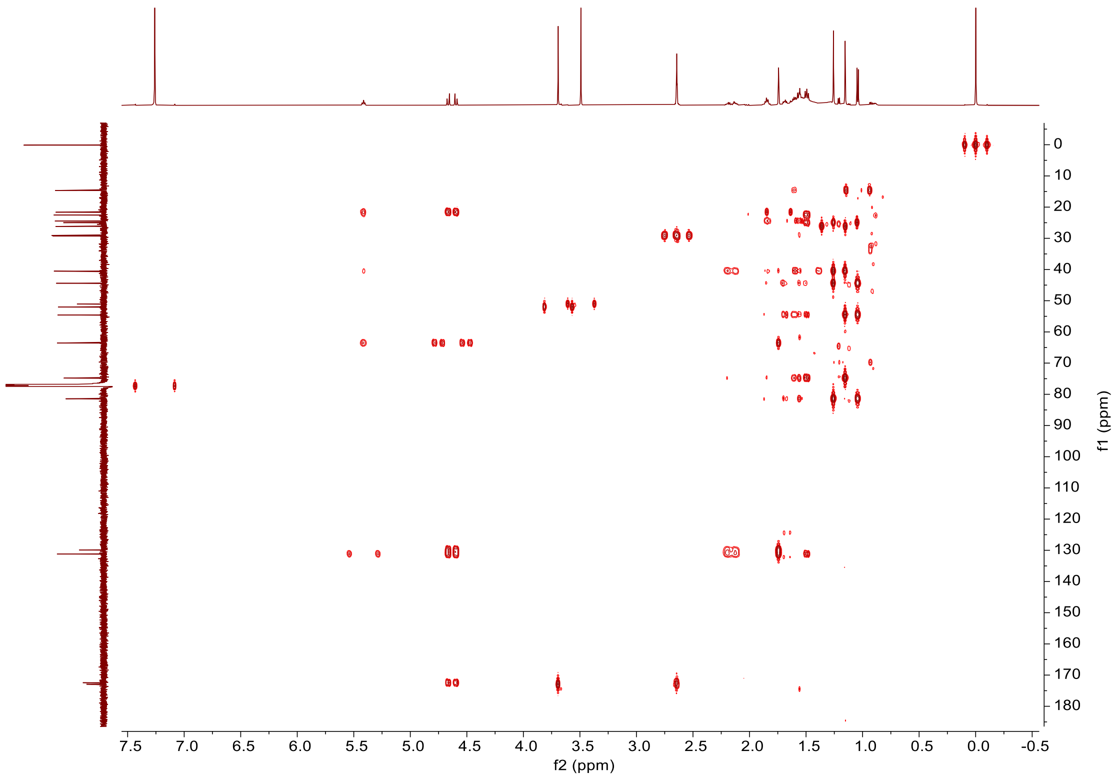

Figure S 53. The HMBC spectrum of compound 6 in $CDCl_3$

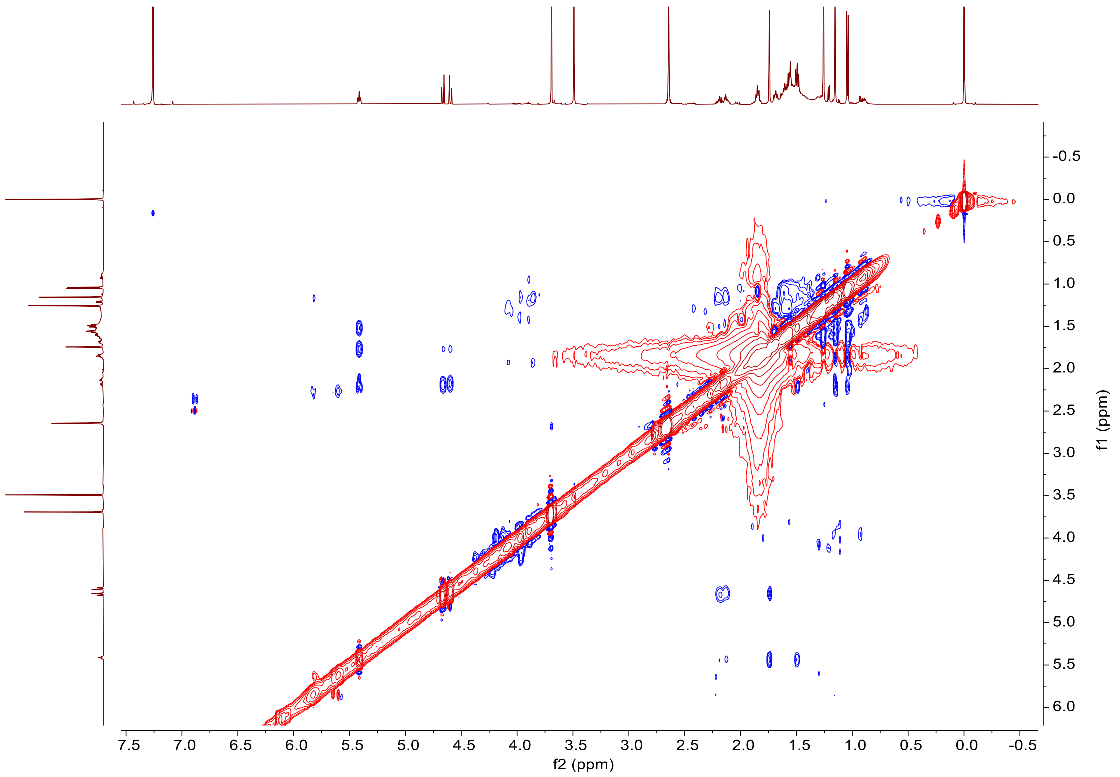

Figure S 54. The NOESY spectrum of compound 6 in $CDCl_3$

.	Component name	Formula	Adducts	Observed m/z	Observed neutral mass (Da)	Neutral mass (Da)	Mass error (ppm)
1	2-1	C20H34O6	+Na	393.2252	370.2360	370.2355	1.1

Item name: 20231106-gdl-2-1-PM
Channel name: Low energy : Time 5.7188 +/- 0.0516 minutes
Item description:

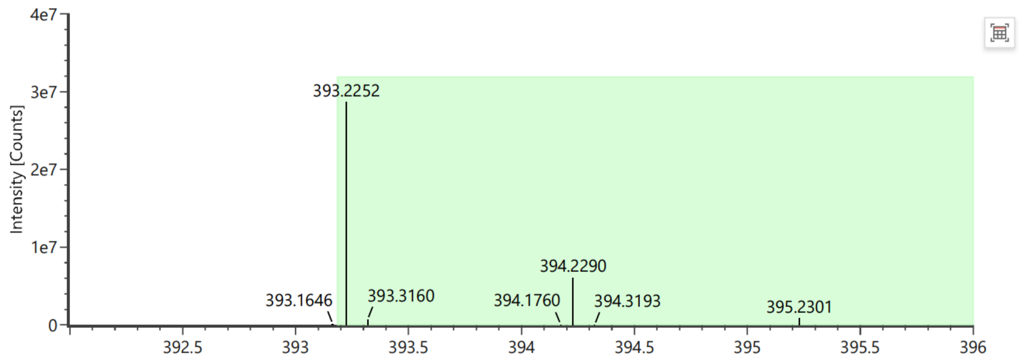

Item name: 20231106-gdl-2-1-PM
Channel name: High energy : Time 5.7188 +/- 0.0516 minutes

Figure S 55. HRESIMS spectrum of compound 7

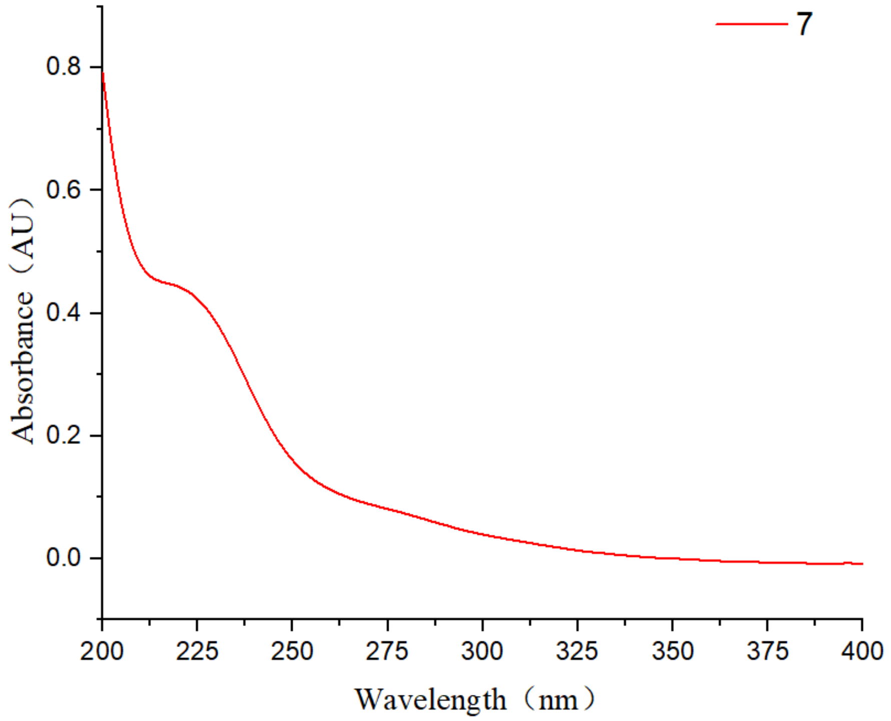

Figure S 56. UV spectrum of compound 7

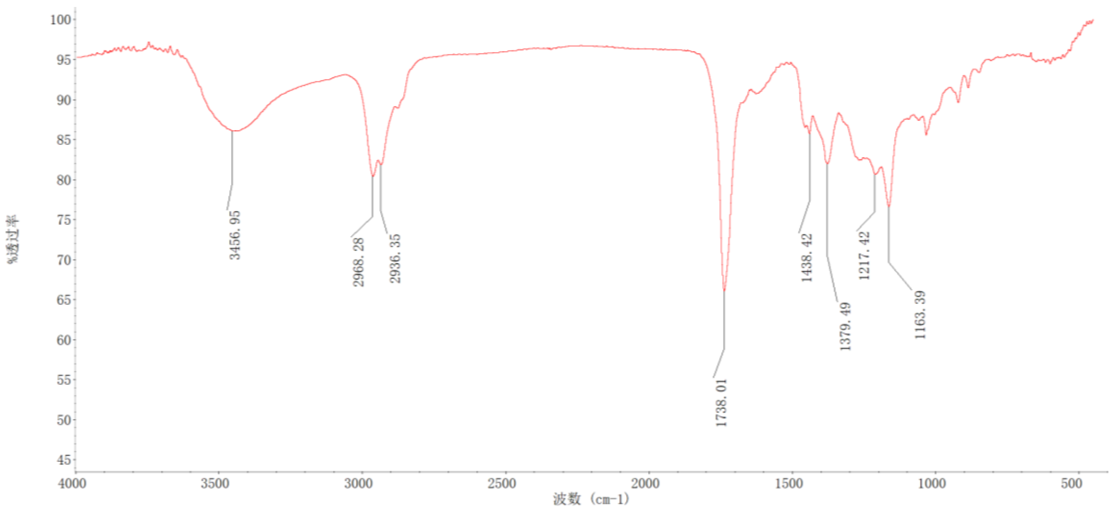

Figure S 57. IR spectrum of compound 7

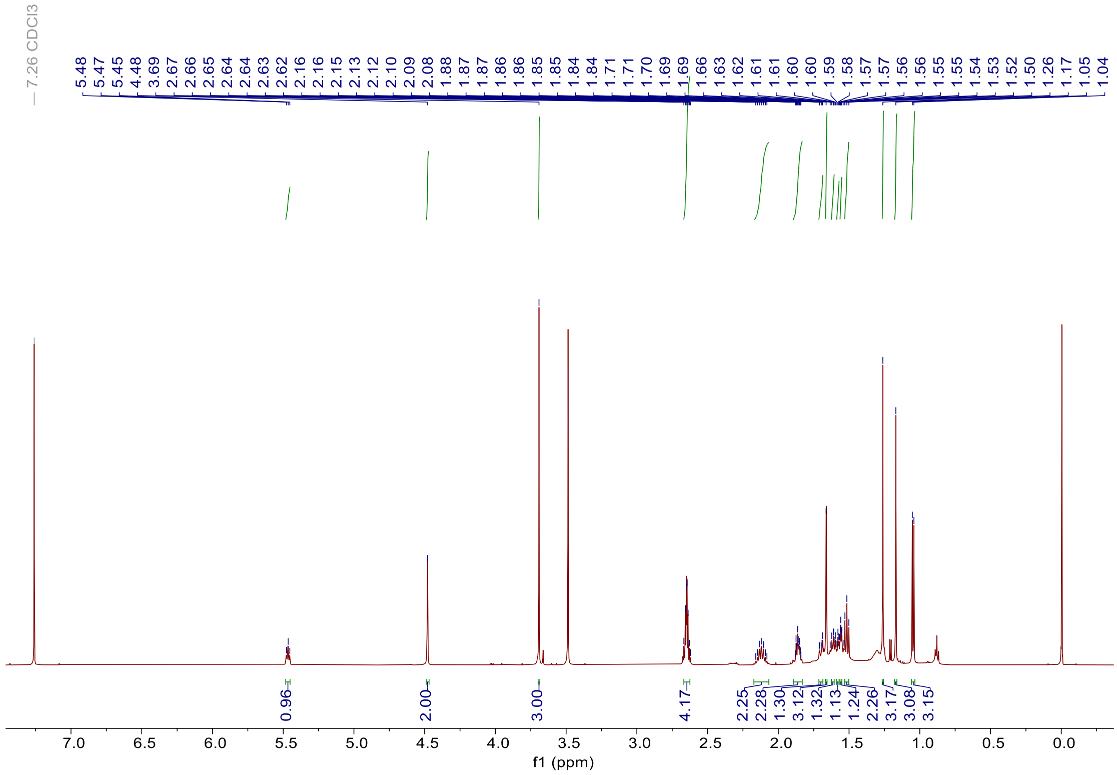

Figure S 58. The ^{1}H NMR spectrum of compound 7 in $CDCl_3$ (600MHz)

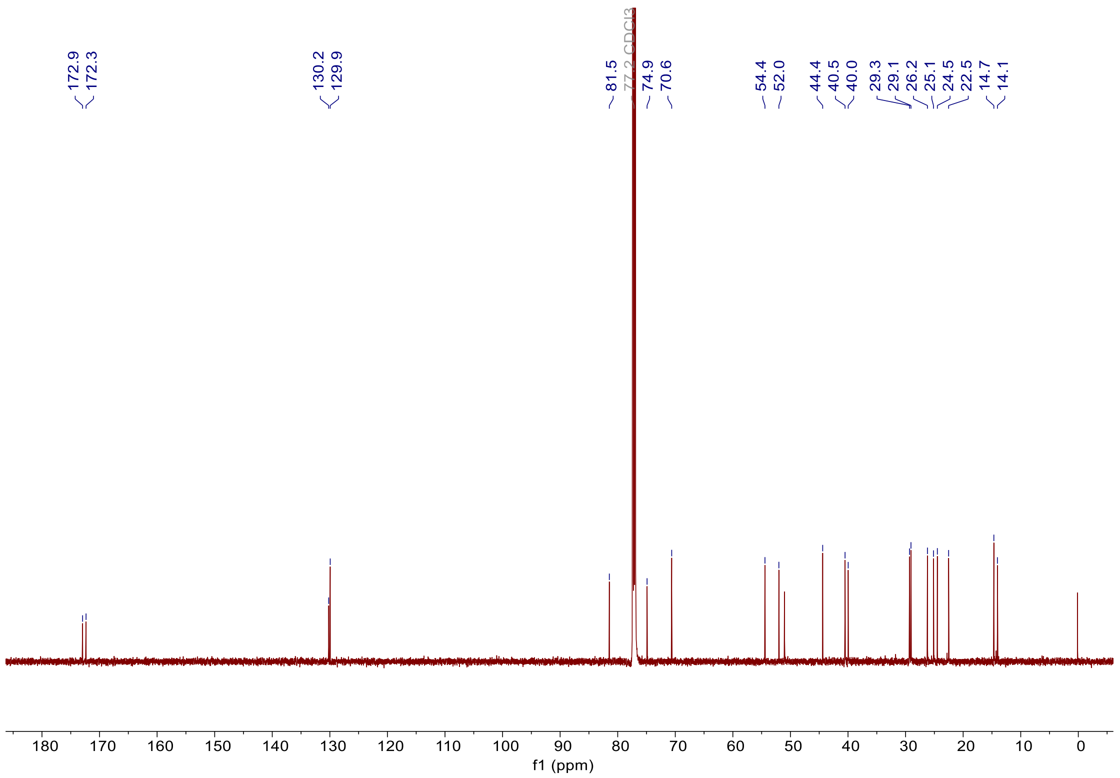

Figure S 59. The ^{13}C NMR spectrum of compound 7 in $CDCl_3$ (150MHz)

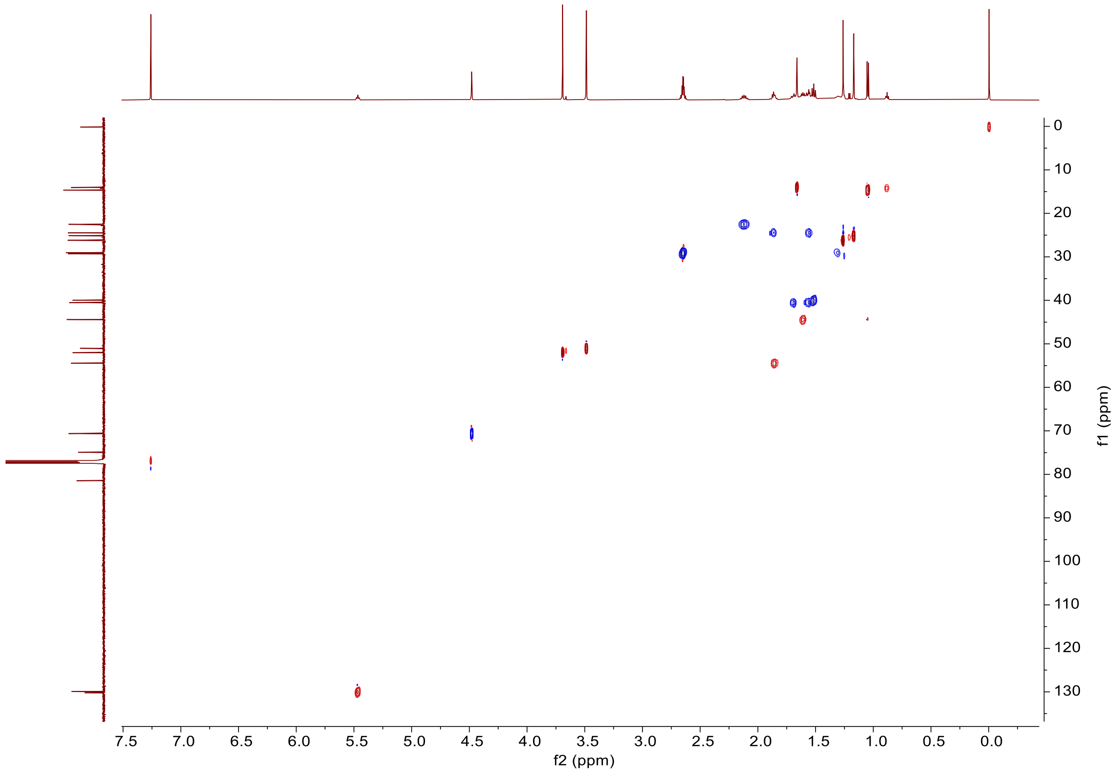

Figure S 60. The HSQC spectrum of compound 7 in $CDCl_3$

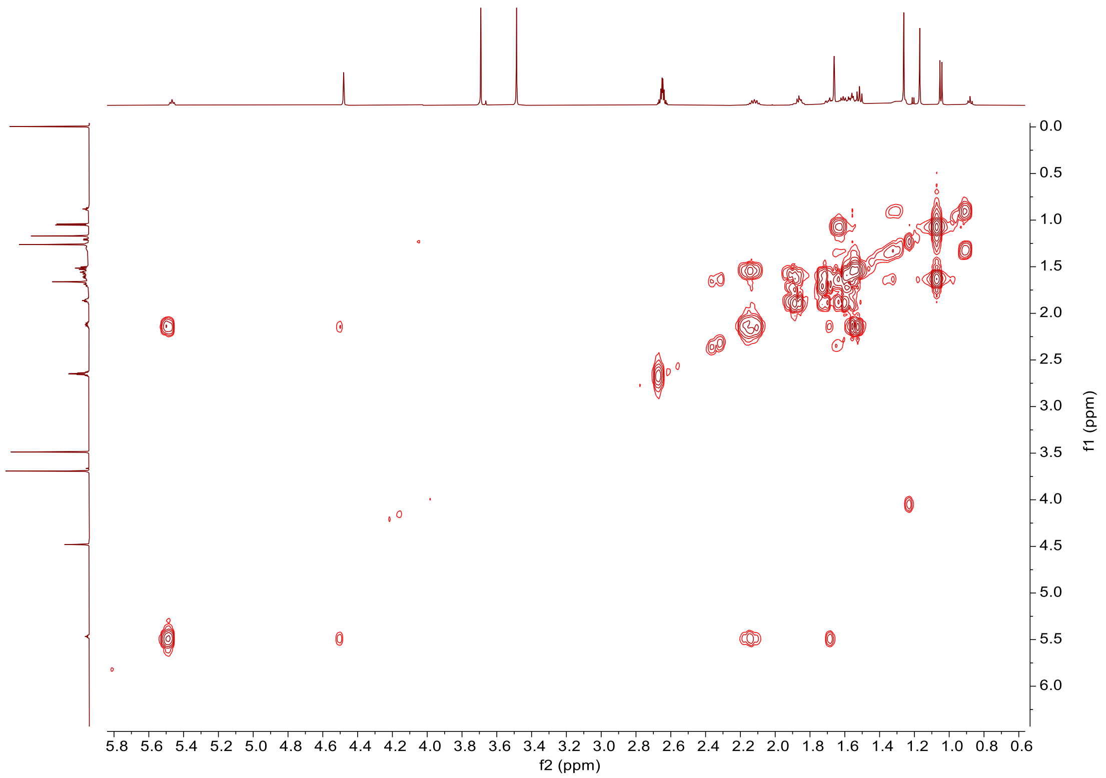

Figure S 61. The ^{1}H-^{1}H COSY spectrum of compound 7 in $CDCl_3$

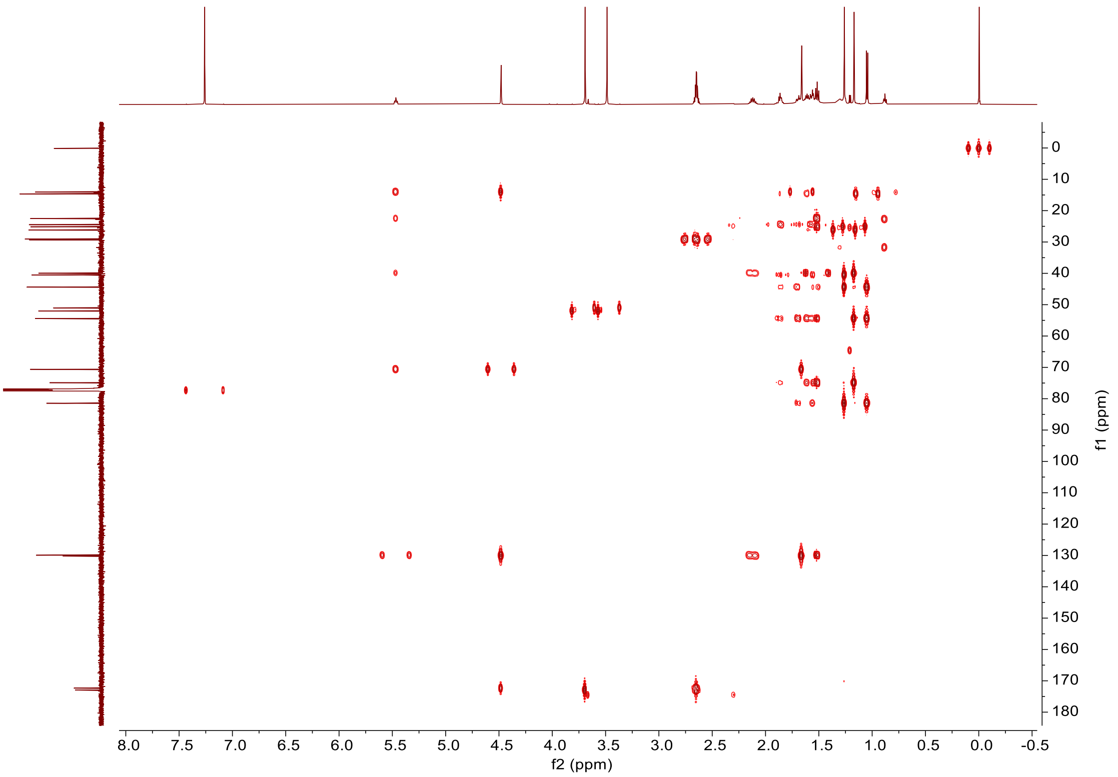

Figure S 62. The HMBC spectrum of compound 7 in $CDCl_3$

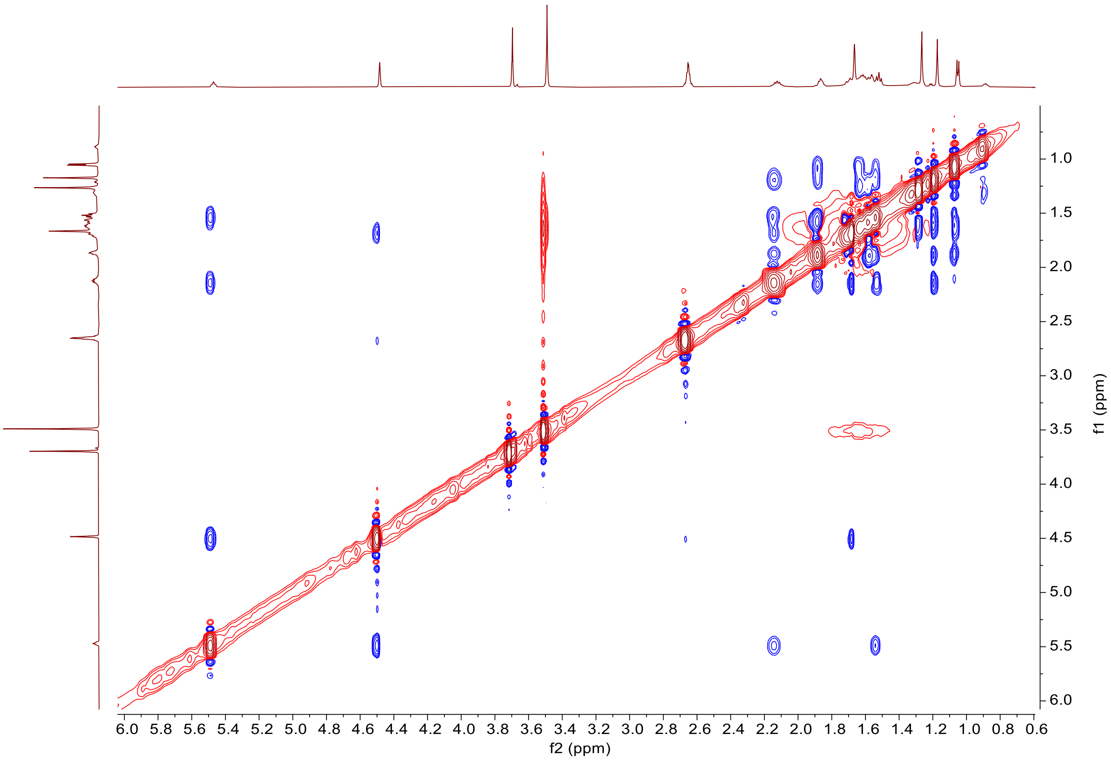

Figure S 63. The NOESY spectrum of compound 7 in $CDCl_3$

.	Component name	Formula	Adducts	Observed m/z	Observed neutral mass (Da)	Neutral mass (Da)	Mass error (ppm)
1	4-1	C15H28O3	+Na	279.1941	256.2048	256.2038	3.6

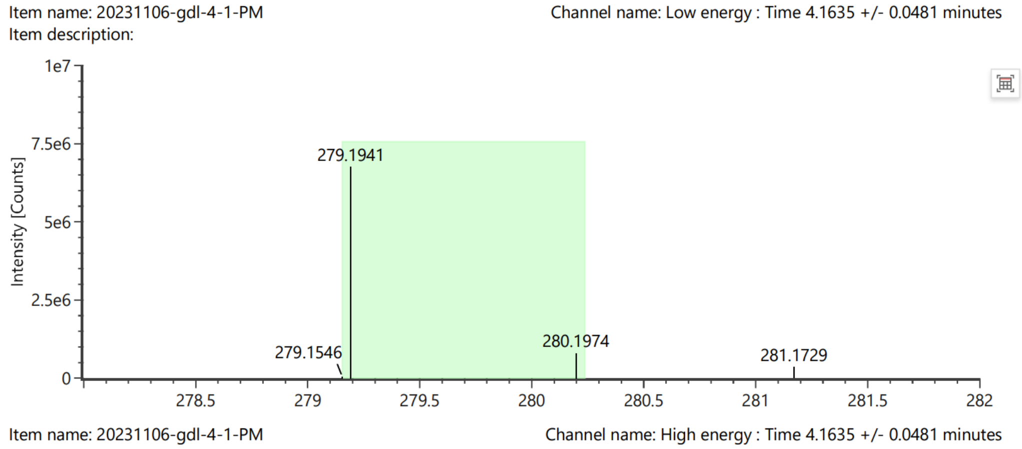

Figure S 64. HRESIMS spectrum of compound 8

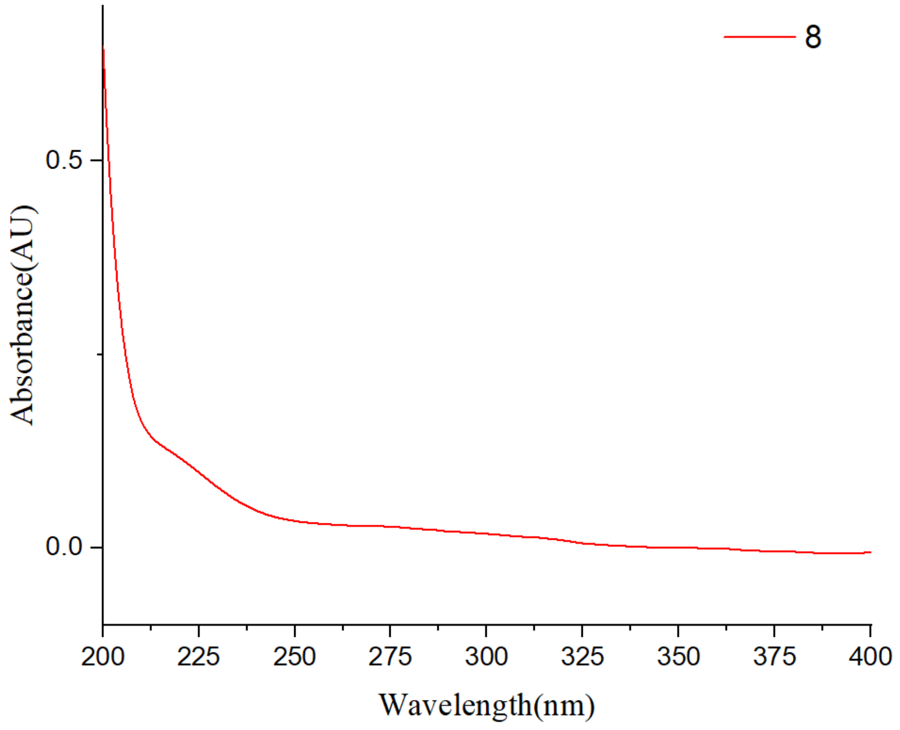

Figure S 65. UV spectrum of compound 8

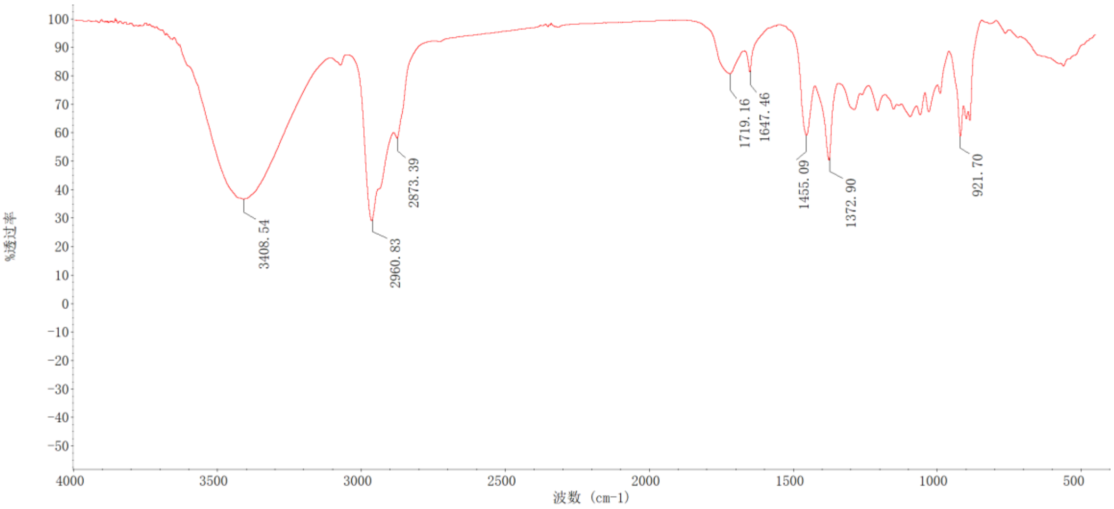

Figure S 66. IR spectrum of compound 8

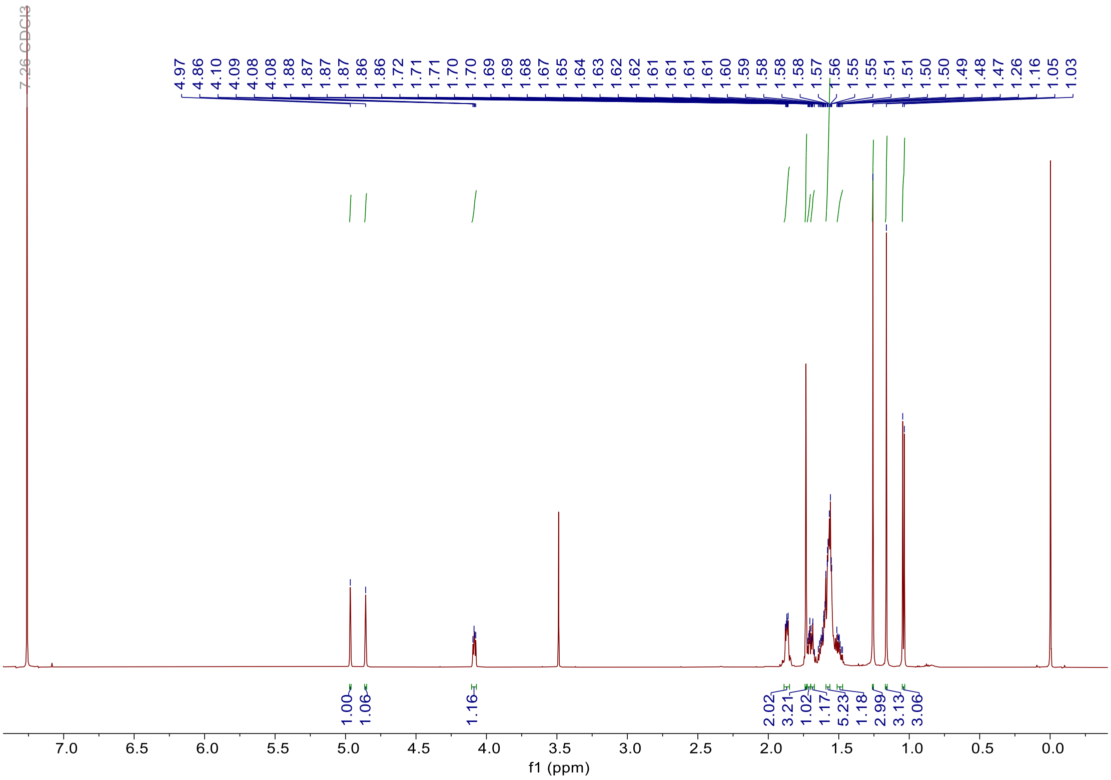

Figure S 67. The ^{1}H NMR spectrum of compound 8 in $CDCl_3$ (600MHz)

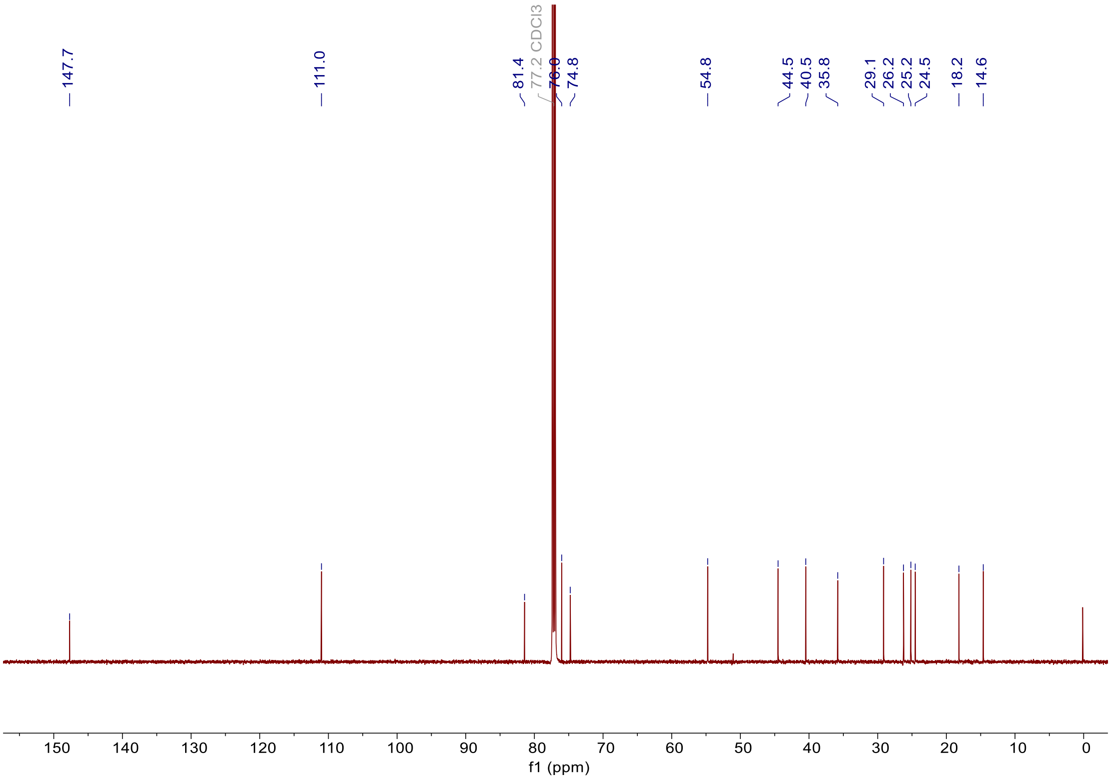

Figure S 68. The ^{13}C NMR spectrum of compound 8 in $CDCl_3$ (150MHz)

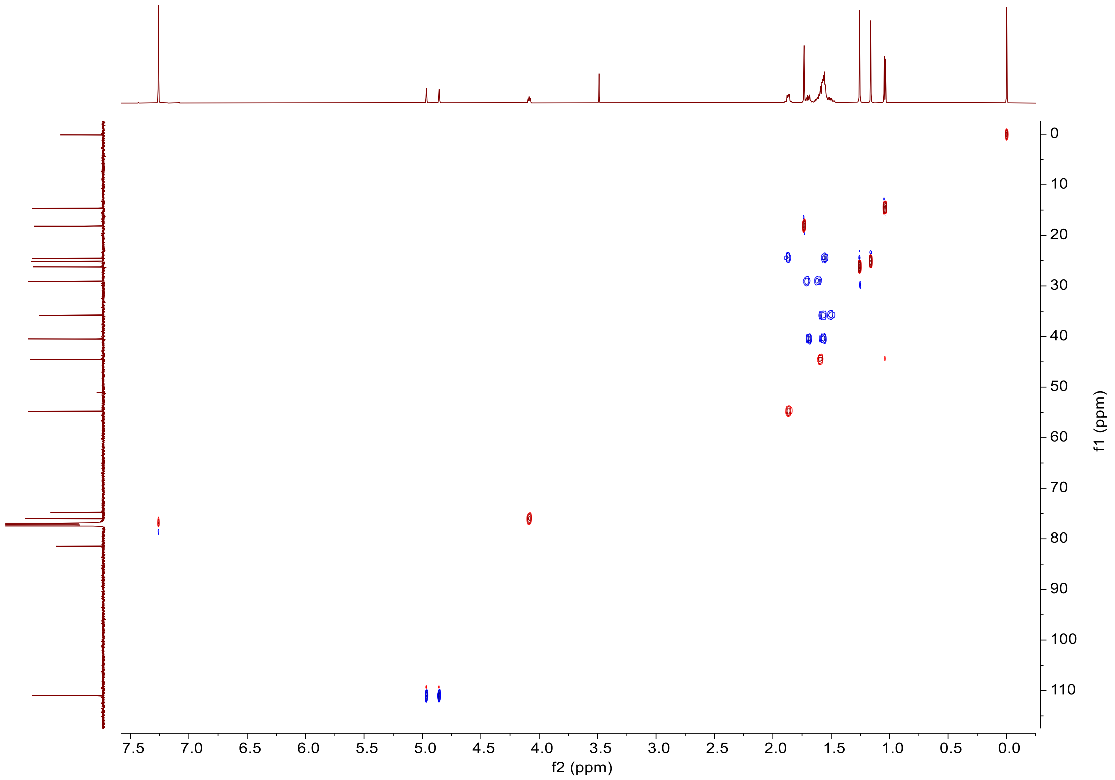

Figure S 69. The HSQC spectrum of compound 8 in $CDCl_3$

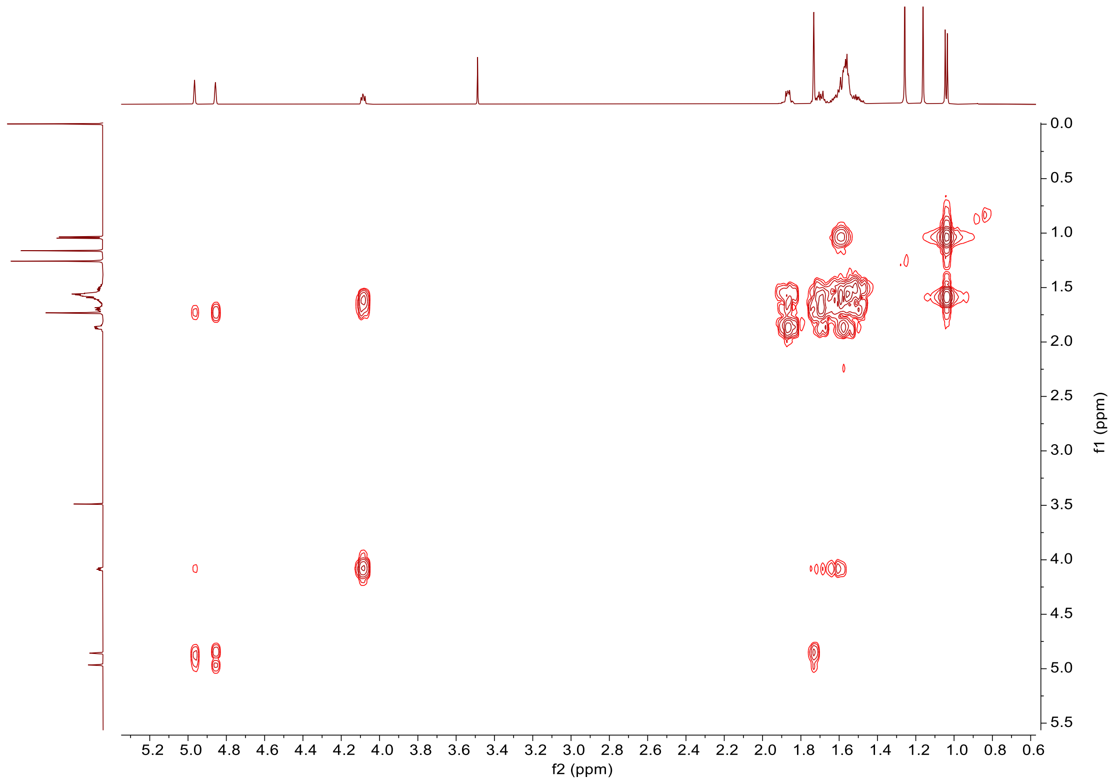

Figure S 70. The ^{1}H-^{1}H COSY spectrum of compound 8 in $CDCl_3$

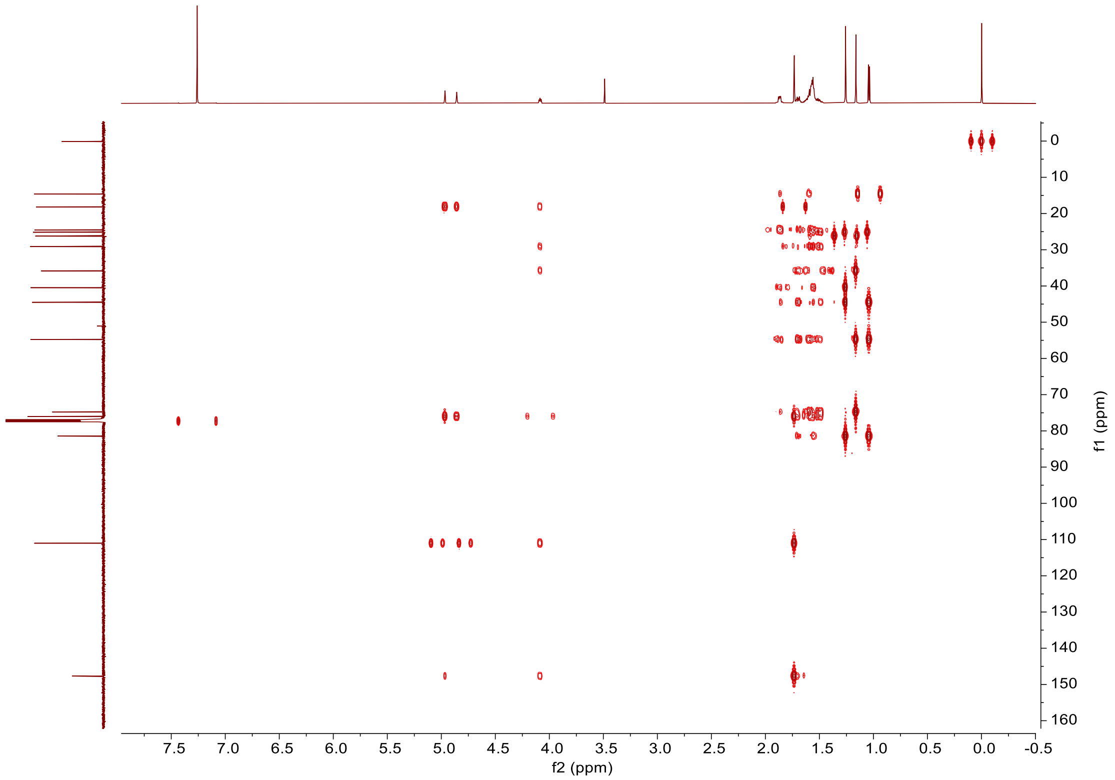

Figure S 71. The HMBC spectrum of compound 8 in $CDCl_3$

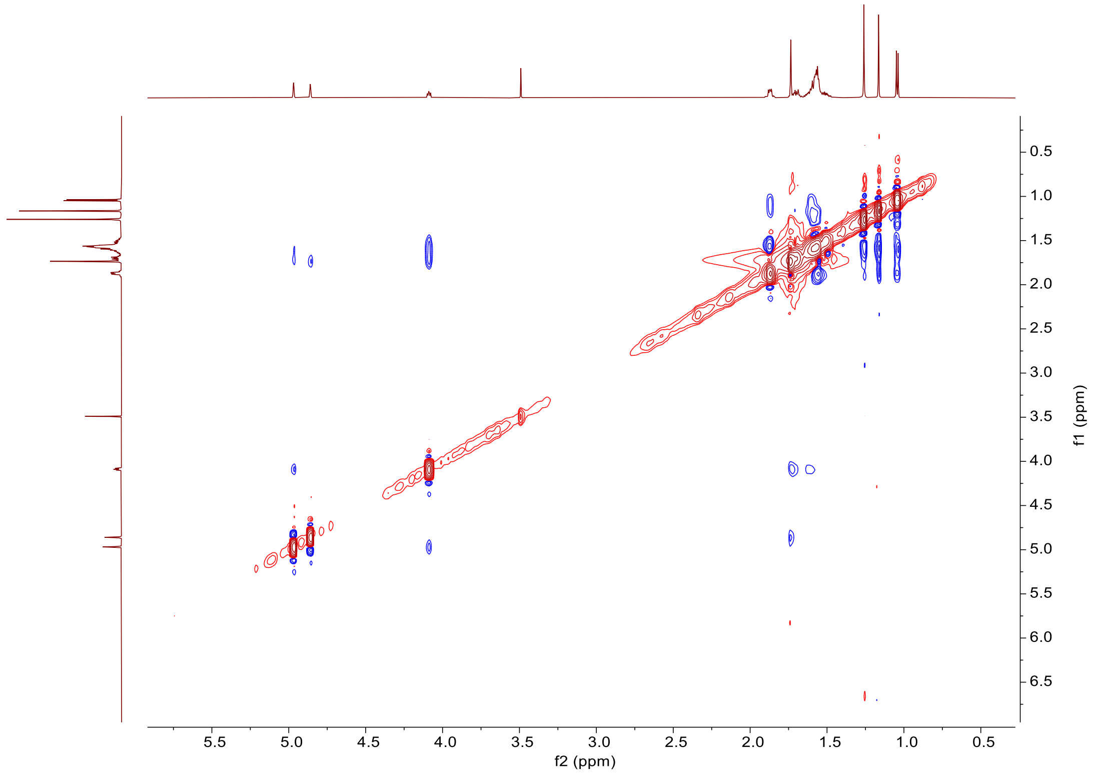

Figure S 72.The NOESY spectrum of compound 8 in $CDCl_3$

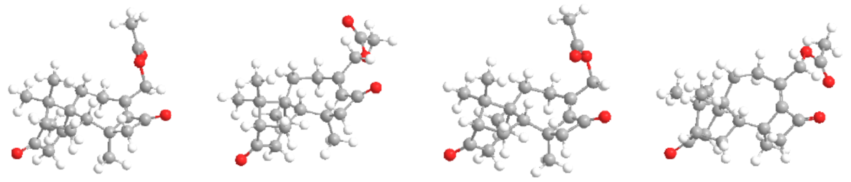

1a-1 1a-2 1a-3 1a-4

Figure S 73. Most stable conformers of 2*S*, 5*R*, 6*R*, 13*S*, 14*S*-1 calculated at MPW1PW91/6-31G+d, p level

Table S 1. Optimized Z-Matrixes of 2*S*, 5*R*, 6*R*, 13*S*, 14*S*-1 conformations in the methanol at CAM-B3LYP/DGDZVP level

	1a-1			1a-2			1a-3		
C	1.762045	-0.58812	-1.42332	1.533586	-1.01384	-1.20693	-1.93991	-0.13127	1.45462
C	3.206476	-0.02577	-1.42992	2.897056	-0.49742	-1.7322	-3.27374	0.570302	1.092559
C	3.990252	-0.16925	-0.14414	3.969555	-0.26026	-0.69215	-3.87299	0.22342	-0.25238
C	3.173373	-0.11259	1.121149	3.472023	0.193446	0.65648	-2.87955	-0.09584	-1.34001
C	2.367823	1.207233	1.148928	2.61894	1.471567	0.480967	-1.8974	1.088028	-1.49772
C	0.97138	0.840451	0.599773	1.156988	0.976387	0.423071	-0.66461	0.708152	-0.64749
C	1.112406	-0.59891	-0.00116	1.237776	-0.5796	0.265309	-1.09764	-0.53479	0.201024
C	0.225843	1.962202	-0.14194	0.190947	1.824802	-0.41778	0.114203	1.884777	-0.03449
C	-1.26438	1.650571	-0.31943	-1.27851	1.492281	-0.13794	1.505267	1.460246	0.453117
C	-1.94933	0.538586	-0.61986	-1.94815	0.352233	0.076983	1.973805	0.367477	1.069761
C	-1.2872	-0.75158	-1.04206	-1.32844	-1.01754	-0.07248	1.078791	-0.70409	1.646454
C	-0.25399	-1.30528	-0.03926	-0.05496	-1.24192	0.771249	0.130923	-1.36009	0.620598
C	-0.20124	3.12455	0.830433	-0.07312	3.238108	0.222389	0.839047	2.735993	-1.14383
C	-1.64724	2.894703	0.40313	-1.56305	2.911837	0.222595	2.162539	2.444935	-0.44387
O	5.208048	-0.28387	-0.14239	5.15775	-0.39287	-0.95047	-5.08122	0.241664	-0.44238
C	-3.44986	0.532383	-0.51351	-3.37008	0.399279	0.568674	3.461307	0.152028	1.184284
O	-2.68328	3.502033	0.592545	-2.56303	3.570459	0.431128	3.293262	2.871276	-0.58007
C	0.852679	2.586064	-1.39577	0.466667	2.024682	-1.91462	-0.59588	2.842441	0.930039
H	0.332606	0.686432	1.473437	2.480164	-0.87227	1.193881	-1.99669	-1.27853	-0.86152
C	2.098229	-1.22956	1.057845	1.358869	-2.50714	-1.54411	-2.17446	-1.22108	2.51794
C	1.690652	-1.89658	-2.23376	2.210068	-0.61597	2.69038	-1.20303	-1.84416	-2.05671
C	1.470751	-1.41824	2.453787	3.074579	-2.28762	1.120933	-2.84855	-2.44742	-0.34256
C	2.715158	-2.59005	0.697091	-4.21511	-0.29566	-0.37329	3.826073	-1.19629	0.824533
O	-3.84382	-0.47075	0.447608	-4.84037	-1.41961	0.024194	3.785899	-1.50902	-0.4837
C	-4.53284	-1.54475	0.019209	-5.68416	-1.98142	-1.08472	4.110419	-2.95896	-0.7052
C	-4.84539	-2.46879	1.16183	-4.7251	-1.90423	1.132757	3.504595	-0.70938	-1.35421
O	-4.8449	-1.73156	-1.1404	0.774102	-0.52655	-1.81494	-1.34529	0.612371	1.981597
H	1.168228	0.107401	-2.01299	3.304932	-1.15953	-2.49887	-4.03373	0.400575	1.858163
H	3.798152	-0.45818	-2.23957	2.75837	0.475038	-2.21859	-3.11957	1.655116	1.059265
H	3.179221	1.052968	-1.62256	4.329786	0.335886	1.317546	-3.42575	-0.32702	-2.25724
H	3.84318	-0.22603	1.976447	2.767417	2.142696	1.32958	-1.62104	1.209445	-2.54726
H	2.295402	1.581886	2.172163	2.912481	2.03401	-0.40924	-2.35106	2.033754	-1.18963
H	2.859498	1.993111	0.569647	0.751974	1.107216	1.429932	0.068932	0.293895	-1.34406

H	-0.83753	-0.60089	-2.02788	-1.13906	-1.2081	-1.13242	0.516374	-0.27459	2.480284
H	-2.05739	-1.51247	-1.18776	-2.06197	-1.76672	0.235698	1.704019	-1.48845	2.078194
H	-0.70734	-1.26963	0.95582	-0.2648	-0.89326	1.786947	0.719082	-1.60438	-0.26923
H	-0.10566	-2.36569	-0.2639	0.10148	-2.32175	0.852209	-0.20023	-2.31761	1.033514
H	0.20857	4.116106	0.62652	0.232955	4.112299	-0.35612	0.558769	3.788949	-1.21936
H	-0.06482	2.890575	1.890141	0.299684	3.335529	1.246307	0.812882	2.284656	-2.13967
H	-3.83531	1.482078	-0.14649	-3.44628	-0.08388	1.544791	4.021057	0.864638	0.579193
H	-3.89979	0.303266	-1.48124	-3.74763	1.417789	0.630121	3.772055	0.239823	2.226784
H	1.864888	2.945483	-1.19004	1.472926	2.421311	-2.07665	-1.50425	3.251848	0.478956
H	0.888154	1.908348	-2.24777	0.347919	1.117166	-2.50496	-0.85481	2.385911	1.884674
H	0.263421	3.453149	-1.70929	-0.23483	2.758381	-2.32328	0.059368	3.689556	1.15455
H	2.045155	-1.69355	-3.2485	1.435042	-2.62656	-2.6288	-2.64555	-0.75766	3.389661
H	2.300823	-2.70399	-1.83251	2.107411	-3.1567	-1.09358	-2.82335	-2.03191	2.19099
H	0.668922	-2.26972	-2.32512	0.376766	-2.88391	-1.25247	-1.23917	-1.66565	2.863366
H	0.736003	-2.22802	2.443924	1.543573	-1.37924	3.101311	-0.60498	-2.70776	-1.75294
H	2.255312	-1.70091	3.16159	3.154201	-0.68077	3.238806	-1.9063	-2.1893	-2.82007
H	0.984533	-0.52739	2.85363	1.778502	0.361331	2.910448	-0.53661	-1.12555	-2.53568
H	3.30772	-2.95221	1.542696	3.866678	-2.38445	1.869612	-3.35877	-2.9232	-1.18563
H	1.940767	-3.3352	0.494211	2.318722	-3.04683	1.340767	-2.22715	-3.20805	0.138492
H	3.386525	-2.55897	-0.16158	3.526152	-2.5299	0.158542	-3.62487	-2.1506	0.363258
H	-3.91374	-2.82245	1.607108	-6.4274	-1.24403	-1.39177	5.027144	-3.22837	-0.17987
H	-5.39944	-1.93177	1.932947	-5.05475	-2.1989	-1.94916	3.301014	-3.56925	-0.29891
H	-5.42807	-3.31417	0.803703	-6.17878	-2.88937	-0.74794	4.213639	-3.15468	-1.76998
	1a-4								
C	1.533271	-1.16385	-1.01779						
C	2.721896	-0.57737	-1.82101						
C	3.868808	-0.01863	-1.00825						
C	3.485085	0.59968	0.311924						
C	2.430454	1.704512	0.069202						
C	1.066938	1.02123	0.312333						
C	1.36498	-0.51402	0.393892						
C	-0.1176	1.570644	-0.49614						
C	-1.46993	1.105099	0.05362						
C	-1.92629	-0.05587	0.540136						
C	-1.12594	-1.3375	0.525148						
C	0.264024	-1.23575	1.189804						
C	-0.51369	3.029502	-0.05893						
C	-1.92146	2.515438	0.22619						
O	5.019912	-0.03753	-1.42153						
C	-3.26406	-0.1039	1.235681						
O	-2.97285	3.064402	0.492283						
C	-0.07034	1.530106	-2.03						
H	2.746058	-0.47189	1.156898						

C	1.550149	-2.70326	-1.0822						
C	2.632844	0.011645	2.616887						
C	3.536975	-1.78807	1.220829						
C	-4.05477	-1.21682	0.772318						
O	-4.70585	-1.06022	-0.39523						
C	-5.44675	-2.31188	-0.77273						
C	-4.68261	-0.03262	-1.04256						
O	0.635701	-0.89736	-1.57209						
H	3.126352	-1.30819	-2.52448						
H	2.373681	0.264423	-2.43073						
H	4.388898	0.967511	0.802774						
H	2.580871	2.528493	0.770057						
H	2.517599	2.13462	-0.93195						
H	0.777954	1.276995	1.335081						
H	-1.04584	-1.69279	-0.50622						
H	-1.69474	-2.10502	1.055283						
H	0.133562	-0.73785	2.155445						
H	0.59453	-2.25296	1.420571						
H	-0.4254	3.816198	-0.81124						
H	-0.03047	3.360051	0.865282						
H	-3.11431	-0.31023	2.297511						
H	-3.82057	0.823912	1.117257						
H	0.833745	2.01737	-2.40607						
H	-0.12328	0.523149	-2.44155						
H	-0.92319	2.080451	-2.43883						
H	1.500813	-3.00458	-2.13263						
H	2.443432	-3.15758	-0.65694						
H	0.685617	-3.14681	-0.58489						
H	2.150108	-0.74588	3.240444						
H	3.637684	0.170357	3.018791						
H	2.086476	0.94754	2.741014						
H	4.423203	-1.64559	1.846631						
H	2.941162	-2.58745	1.670432						
H	3.894281	-2.1383	0.252111						
H	-4.73208	-3.12109	-0.93367						
H	-6.1116	-2.61177	0.038365						
H	-6.01912	-2.14099	-1.68148						

Table S 2. Energy analysis for 2*S*, 5*R*, 6*R*, 13*S*, 14*S*-1

Conf.	Steric Energy	Relative Energy	Distribution
	(kJ/mol)	(kJ/mol)	(%)
1a-1	-1156.634474	0.001156	20.15
1a-2	-1156.633159	0.002471	5.01
1a-3	-1156.63563	0	68.51

1a-4	-1156.63338	0.00225	6.33

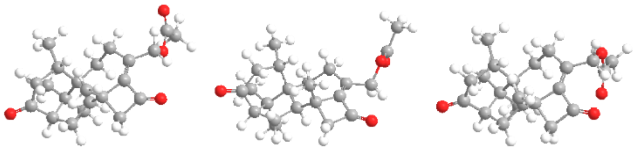

Figure S 74. Most stable conformers of 2*R*, 5*S*, 6*S*, 13*R*, 14*R*-1 calculated at MPW1PW91/6-31G+d, p level

Table S 3. Optimized Z-Matrixes of 2*R*, 5*S*, 6*S*, 13*R*, 14*R*-1 conformations in the methanol at CAM-B3LYP/DGDZVP level.

	1b-1			1b-2			1b-3		
C	-3.20648	-0.02577	-1.42992	-2.89706	-0.49742	-1.7322	3.273739	0.570302	1.092559
C	-3.99025	-0.16925	-0.14414	-3.96956	-0.26026	-0.69215	3.872994	0.22342	-0.25238
C	-3.17337	-0.11259	1.121149	-3.47202	0.193446	0.65648	2.879552	-0.09584	-1.34001
C	-2.36782	1.207233	1.148928	-2.61894	1.471567	0.480967	1.897401	1.088028	-1.49772
C	-0.97138	0.840451	0.599773	-1.15699	0.976387	0.423071	0.664609	0.708152	-0.64749
C	-1.11241	-0.59891	-0.00116	-1.23778	-0.5796	0.265309	1.097635	-0.53479	0.201024
C	-1.76205	-0.58812	-1.42332	-1.53359	-1.01384	-1.20693	1.939908	-0.13127	1.45462
C	-2.09823	-1.22956	1.057845	-2.48016	-0.87227	1.193881	1.996693	-1.27853	-0.86152
C	-1.69065	-1.89658	-2.23376	-1.35887	-2.50714	-1.54411	2.174461	-1.22108	2.51794
C	-0.22584	1.962202	-0.14194	-0.19095	1.824802	-0.41778	-0.1142	1.884777	-0.03449
C	1.264383	1.650571	-0.31943	1.27851	1.492281	-0.13794	-1.50527	1.460246	0.453117
C	1.949333	0.538586	-0.61986	1.948149	0.352233	0.076983	-1.97381	0.367477	1.069761
C	1.287198	-0.75158	-1.04206	1.328436	-1.01754	-0.07248	-1.07879	-0.70409	1.646454
C	0.25399	-1.30528	-0.03926	0.054963	-1.24192	0.771249	-0.13092	-1.36009	0.620598
C	-2.71516	-2.59005	0.697091	-3.07458	-2.28762	1.120933	2.84855	-2.44742	-0.34256
C	-1.47075	-1.41824	2.453787	-2.21007	-0.61597	2.69038	1.203033	-1.84416	-2.05671
H	-0.33261	0.686432	1.473437	-0.75197	1.107216	1.429932	-0.06893	0.293895	-1.34406
C	3.449858	0.532383	-0.51351	3.370084	0.399279	0.568674	-3.46131	0.152028	1.184284
C	0.201241	3.12455	0.830433	0.073122	3.238108	0.222389	-0.83905	2.735993	-1.14383
C	1.647244	2.894703	0.40313	1.563047	2.911837	0.222595	-2.16254	2.444935	-0.44387
O	2.683276	3.502033	0.592545	2.563025	3.570459	0.431128	-3.29326	2.871276	-0.58007
C	-0.85268	2.586064	-1.39577	-0.46667	2.024682	-1.91462	0.595883	2.842441	0.930039
O	3.843815	-0.47075	0.447608	4.215108	-0.29566	-0.37329	-3.82607	-1.19629	0.824533
O	-5.20805	-0.28387	-0.14239	-5.15775	-0.39287	-0.95047	5.081222	0.241664	-0.44238
C	4.532841	-1.54475	0.019209	4.840369	-1.41961	0.024194	-3.7859	-1.50902	-0.4837
C	4.845385	-2.46879	1.16183	5.684161	-1.98142	-1.08472	-4.11042	-2.95896	-0.7052
O	4.844902	-1.73156	-1.1404	4.725095	-1.90423	1.132757	-3.5046	-0.70938	-1.35421
H	-3.17922	1.052968	-1.62256	-3.30493	-1.15953	-2.49887	4.033728	0.400575	1.858163
H	-3.79815	-0.45818	-2.23957	-2.75837	0.475038	-2.21859	3.119565	1.655116	1.059265
H	-3.84318	-0.22603	1.976447	-4.32979	0.335886	1.317546	3.425754	-0.32702	-2.25724
H	-2.2954	1.581886	2.172163	-2.76742	2.142696	1.32958	1.621044	1.209445	-2.54726
H	-2.8595	1.993111	0.569647	-2.91248	2.03401	-0.40924	2.351057	2.033754	-1.18963

H	-1.16823	0.107401	-2.01299	-0.7741	-0.52655	-1.81494	1.345287	0.612371	1.981597
H	-2.04516	-1.69355	-3.2485	-1.43504	-2.62656	-2.6288	2.645552	-0.75766	3.389661
H	-0.66892	-2.26972	-2.32512	-0.37677	-2.88391	-1.25247	1.239171	-1.66565	2.863366
H	-2.30082	-2.70399	-1.83251	-2.10741	-3.1567	-1.09358	2.823345	-2.03191	2.19099
H	0.837529	-0.60089	-2.02788	1.139062	-1.2081	-1.13242	-0.51637	-0.27459	2.480284
H	2.057394	-1.51247	-1.18776	2.061965	-1.76672	0.235698	-1.70402	-1.48845	2.078194
H	0.105663	-2.36569	-0.2639	-0.10148	-2.32175	0.852209	0.200231	-2.31761	1.033514
H	0.707336	-1.26963	0.95582	0.2648	-0.89326	1.786947	-0.71908	-1.60438	-0.26923
H	-3.30772	-2.95221	1.542696	-3.86668	-2.38445	1.869612	3.358767	-2.9232	-1.18563
H	-1.94077	-3.3352	0.494211	-2.31872	-3.04683	1.340767	2.227148	-3.20805	0.138492
H	-3.38653	-2.55897	-0.16158	-3.52615	-2.5299	0.158542	3.624874	-2.1506	0.363258
H	-0.98453	-0.52739	2.85363	-1.7785	0.361331	2.910448	0.536608	-1.12555	-2.53568
H	-2.25531	-1.70091	3.16159	-3.1542	-0.68077	3.238806	1.906298	-2.1893	-2.82007
H	-0.736	-2.22802	2.443924	-1.54357	-1.37924	3.101311	0.604975	-2.70776	-1.75294
H	3.899787	0.303266	-1.48124	3.747634	1.417789	0.630121	-3.77206	0.239823	2.226784
H	3.835311	1.482078	-0.14649	3.446283	-0.08388	1.544791	-4.02106	0.864638	0.579193
H	-0.20857	4.116106	0.62652	-0.23296	4.112299	-0.35612	-0.55877	3.788949	-1.21936
H	0.06482	2.890575	1.890141	-0.29968	3.335529	1.246307	-0.81288	2.284656	-2.13967
H	-0.88815	1.908348	-2.24777	-0.34792	1.117166	-2.50496	0.854809	2.385911	1.884674
H	-1.86489	2.945483	-1.19004	-1.47293	2.421311	-2.07665	1.504254	3.251848	0.478956
H	-0.26342	3.453149	-1.70929	0.234833	2.758381	-2.32328	-0.05937	3.689556	1.15455
H	5.39944	-1.93177	1.932947	5.054754	-2.1989	-1.94916	-3.30101	-3.56925	-0.29891
H	3.913735	-2.82245	1.607108	6.4274	-1.24403	-1.39177	-5.02714	-3.22837	-0.17987
H	5.428067	-3.31417	0.803703	6.178782	-2.88937	-0.74794	-4.21364	-3.15468	-1.76998

Table S 4. Energy analysis for 2*R*, 5*S*, 6*S*, 13*R*, 14*R*-1

Conf.	Steric Energy (kJ/mol)	Relative Energy (kJ/mol)	Distribution (%)
1b-1	-1156.634474	0.001156	21.51
1b-2	-1156.633159	0.002471	5.35
1b-3	-1156.63563	0	73.14

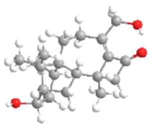

2a-1

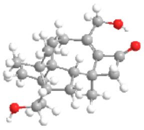

2a-2

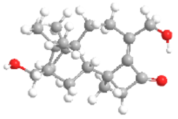

2a-3

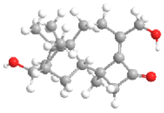

2a-4

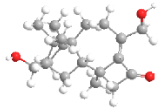

2a-5

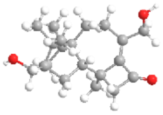

2a-6

Figure S 75. Most stable conformers of 2*S*, 3*S*, 5*R*, 6*R*, 13*S*, 14*S*-2 calculated at MPW1PW91/6-31G+d, p level

Table S 5. Optimized Z-Matrixes of 2*S*, 3*S*, 5*R*, 6*R*, 13*S*, 14*S*-2 conformations in the Chloroform at CAM-B3LYP/DGDZVP level

	2a-1			2a-2			2a-3		
C	-2.25964	-0.44812	-1.71081	-2.26025	-0.45609	-1.69773	-2.38792	-0.71561	-1.54332
C	-3.1908	-1.06339	-0.66422	-3.19555	-1.06662	-0.64274	-3.21809	-1.17267	-0.34222
C	-2.53569	-1.07632	0.723738	-2.53694	-1.07311	0.737725	-2.45489	-0.95773	0.971086
C	-1.21614	-1.87972	0.615727	-1.21546	-1.87468	0.631269	-1.13182	-1.75868	0.883297
C	-0.10534	-0.8134	0.4597	-0.10405	-0.81019	0.466244	-0.05386	-0.71954	0.495998
C	-0.85421	0.541129	0.237126	-0.85307	0.543261	0.239411	-0.84306	0.575357	0.109967
C	-1.34464	0.705908	-1.23936	-1.34726	0.702181	-1.23642	-1.44568	0.494052	-1.33405
C	-2.07083	0.318786	1.213867	-2.06804	0.325607	1.219292	-1.98453	0.503014	1.194979
C	-1.92793	2.072867	-1.64777	-1.93681	2.065497	-1.64774	-2.04458	1.784058	-1.92565
O	-4.45216	-0.3897	-0.71057	-4.4728	-0.42564	-0.56736	-4.49697	-0.53318	-0.39036
C	1.119959	-1.20125	-0.38063	1.118909	-1.20273	-0.37534	1.134142	-1.22651	-0.33737
C	2.299121	-0.22928	-0.21451	2.298939	-0.23056	-0.21712	2.329362	-0.25731	-0.28929
C	2.418853	1.110442	-0.15949	2.419428	1.109349	-0.16854	2.421022	1.079898	-0.38437
C	1.241132	2.05253	-0.25778	1.241923	2.051724	-0.26728	1.223061	1.925845	-0.75677
C	0.040318	1.721381	0.648885	0.043068	1.724988	0.643565	0.055176	1.81701	0.249627
C	-3.14906	1.413251	1.20503	-3.14139	1.42471	1.209001	-3.08303	1.571521	1.084093
C	-1.65916	0.20812	2.701823	-1.65175	0.220468	2.70655	-1.47158	0.637843	2.648942
H	0.34058	-0.69014	1.449998	0.345203	-0.68181	1.45437	0.444228	-0.43787	1.427022
C	3.742829	1.800075	0.08566	3.744475	1.799568	0.069106	3.67167	1.878192	-0.11609
C	2.001119	-2.29217	0.330197	2.001532	-2.2906	0.338336	2.0229	-2.2519	0.458094
C	3.166826	-1.3201	0.2829	3.167642	-1.3195	0.282727	3.210289	-1.33814	0.208441
O	4.338298	-1.41396	0.627242	4.340243	-1.41253	0.623432	4.419093	-1.49142	0.310577
C	0.940239	-1.61763	-1.84626	0.936069	-1.62591	-1.83866	0.896996	-1.79754	-1.74082
O	4.907823	1.111743	-0.30368	4.908025	1.108937	-0.32048	4.725251	1.253468	0.57637
H	-2.86786	-0.11673	-2.55777	-2.85577	-0.13323	-2.55884	-3.07165	-0.51385	-2.37311
H	-1.63691	-1.25644	-2.09701	-1.63584	-1.26918	-2.07274	-1.78791	-1.57048	-1.86125
H	-3.36507	-2.11188	-0.94572	-3.36796	-2.11533	-0.92234	-3.38605	-2.25462	-0.44198
H	-3.22952	-1.53575	1.438534	-3.23351	-1.52483	1.451605	-3.07862	-1.30326	1.804551
H	-1.04369	-2.48184	1.511296	-1.04163	-2.46928	1.531627	-0.88559	-2.22466	1.840652
H	-1.24683	-2.58166	-0.22244	-1.24491	-2.58375	-0.20068	-1.20831	-2.5738	0.157676
H	-0.44058	0.62441	-1.84418	-0.44486	0.621012	-1.84399	-0.59492	0.299755	-1.98548
H	-1.89223	2.148707	-2.73911	-1.90385	2.138401	-2.73942	-2.19511	1.629492	-2.99871
H	-1.35928	2.916214	-1.24944	-1.37054	2.912119	-1.25307	-1.37563	2.641494	-1.8205
H	-2.96872	2.204387	-1.35584	-2.97728	2.193764	-1.35301	-3.00957	2.05998	-1.50423
H	-4.97736	-0.6948	0.041061	-4.88975	-0.51361	-1.43397	-4.95556	-0.72659	0.438015
H	0.940373	2.128446	-1.30805	0.938449	2.124151	-1.31703	0.894974	1.662246	-1.76534
H	1.588124	3.053945	0.014348	1.590352	3.0539	0.000207	1.534643	2.972771	-0.80925
H	-0.56716	2.62829	0.722069	-0.5634	2.632743	0.7141	-0.55411	2.719899	0.154621
H	0.4287	1.54234	1.656541	0.43348	1.550216	1.651196	0.478576	1.847645	1.258538
H	-3.91127	1.177553	1.955734	-3.89141	1.206549	1.976249	-3.78241	1.46216	1.920122
H	-2.71295	2.379713	1.479145	-2.69559	2.394244	1.455845	-2.64711	2.573747	1.15464
H	-3.6647	1.516879	0.25839	-3.67499	1.509852	0.270676	-3.67057	1.505908	0.176358

H	-1.2959	1.167373	3.081811	-1.29096	1.182409	3.082298	-1.09864	1.64754	2.84375
H	-2.54327	-0.05603	3.290444	-2.53346	-0.04492	3.298055	-2.31	0.468844	3.331768
H	-0.90081	-0.54542	2.917241	-0.88994	-0.52933	2.922948	-0.68956	-0.06803	2.930973
H	3.740525	2.735177	-0.48044	3.740764	2.731851	-0.50162	4.006336	2.27594	-1.08781
H	3.772324	2.082963	1.15043	3.777461	2.087702	1.132354	3.38744	2.746439	0.486115
H	2.145535	-3.23521	-0.2016	2.143756	-3.23656	-0.18883	2.096651	-3.26789	0.064892
H	1.709962	-2.50439	1.362914	1.713262	-2.49709	1.373027	1.790644	-2.29282	1.526498
H	0.242358	-2.45537	-1.93152	0.239571	-2.46537	-1.91835	0.164983	-2.60942	-1.71252
H	0.597599	-0.80647	-2.48762	0.59033	-0.81809	-2.48263	0.565475	-1.05172	-2.46217
H	1.899764	-1.95605	-2.24992	1.895111	-1.96445	-2.24342	1.829543	-2.21947	-2.12879
H	4.924824	0.238448	0.133822	4.925691	0.23751	0.120673	4.855263	0.342677	0.254916
	2a-4			2a-5			2a-6		
C	-2.38524	-0.72001	-1.5303	-2.3311	-0.75462	-1.52461	-2.33392	-0.75255	-1.51204
C	-3.22106	-1.17078	-0.32253	-3.22033	-1.08458	-0.32415	-3.22508	-1.0801	-0.30396
C	-2.45683	-0.95163	0.983126	-2.48269	-0.83556	0.998432	-2.4833	-0.83024	1.009788
C	-1.13203	-1.75119	0.898316	-1.20578	-1.71144	1.000607	-1.20496	-1.70501	1.010631
C	-0.05314	-0.71579	0.503005	-0.063	-0.76479	0.560614	-0.06164	-0.76181	0.564578
C	-0.84164	0.57799	0.112805	-0.76793	0.551198	0.090151	-0.76602	0.55385	0.093182
C	-1.44618	0.492201	-1.32996	-1.33848	0.420429	-1.36108	-1.34064	0.421847	-1.35626
C	-1.98271	0.510969	1.198439	-1.93268	0.605297	1.150742	-1.92904	0.611457	1.155332
C	-2.05121	1.778424	-1.92342	-1.87777	1.69849	-2.03162	-1.88303	1.698815	-2.02629
O	-4.50566	-0.54441	-0.24502	-4.45509	-0.37265	-0.45054	-4.47054	-0.37461	-0.29963
C	1.132423	-1.22799	-0.33066	1.090469	-1.39777	-0.23458	1.088602	-1.39854	-0.23231
C	2.328663	-0.25957	-0.2913	2.320102	-0.4847	-0.32418	2.319486	-0.48771	-0.32726
C	2.421708	1.077106	-0.39226	2.495614	0.833222	-0.50316	2.49661	0.829563	-0.50946
C	1.2238	1.923795	-0.7633	1.366763	1.790126	-0.80684	1.368422	1.787698	-0.81181
C	0.058252	1.819171	0.246179	0.193979	1.747777	0.192499	0.1981	1.749124	0.190617
C	-3.07641	1.583508	1.082523	-2.96527	1.726989	0.959364	-2.95718	1.736311	0.961548
C	-1.46755	0.651111	2.651195	-1.43794	0.792699	2.605297	-1.43057	0.801706	2.608264
H	0.447865	-0.42983	1.431165	0.449669	-0.45762	1.475606	0.454609	-0.45281	1.476961
C	3.674671	1.874622	-0.13225	3.870011	1.437068	-0.38711	3.872363	1.431322	-0.39856
C	2.022882	-2.24984	0.467529	1.958104	-2.36829	0.649024	1.957061	-2.36855	0.651021
C	3.210365	-1.33916	0.207867	3.182564	-1.53431	0.28499	3.181906	-1.53744	0.281812
O	4.419406	-1.49388	0.304724	4.37816	-1.69721	0.442412	4.377686	-1.70206	0.436175
C	0.892131	-1.8068	-1.73042	0.805454	-2.10229	-1.5675	0.799221	-2.10547	-1.563
O	4.730305	1.251128	0.558238	3.830743	2.458473	0.609434	3.837779	2.454968	0.595856
H	-3.05528	-0.52872	-2.37538	-2.97665	-0.5772	-2.38997	-2.96944	-0.58117	-2.38786
H	-1.78171	-1.57811	-1.83468	-1.76849	-1.65908	-1.76112	-1.7713	-1.65949	-1.74202
H	-3.38764	-2.2524	-0.42104	-3.45272	-2.15854	-0.36393	-3.45485	-2.15394	-0.34513
H	-3.08569	-1.28929	1.813359	-3.14938	-1.09476	1.830234	-3.15282	-1.08293	1.83858
H	-0.88552	-2.20858	1.859788	-1.00419	-2.11604	1.995616	-1.00155	-2.10405	2.007571
H	-1.20616	-2.57288	0.180192	-1.31551	-2.5742	0.337244	-1.31395	-2.57166	0.352514
H	-0.59639	0.298495	-1.98309	-0.47748	0.157223	-1.97666	-0.48198	0.157736	-1.97488

H	-2.19796	1.622893	-2.99692	-1.93772	1.520523	-3.11004	-1.94547	1.520298	-3.10453
H	-1.38781	2.639893	-1.81647	-1.2239	2.561372	-1.88613	-1.22979	2.562467	-1.88278
H	-3.01877	2.048212	-1.5039	-2.877	1.976939	-1.7005	-2.88147	1.976456	-1.69196
H	-4.99388	-0.79145	-1.04083	-4.94508	-0.48211	0.375158	-4.94939	-0.63347	-1.0974
H	0.892672	1.658698	-1.7705	1.016781	1.609669	-1.82837	1.015404	1.606107	-1.83212
H	1.536759	2.970138	-0.81891	1.774518	2.803416	-0.80211	1.777875	2.800322	-0.81005
H	-0.54985	2.72267	0.149607	-0.37124	2.677229	0.076236	-0.36553	2.679463	0.073991
H	0.483937	1.852673	1.25404	0.619228	1.781785	1.200326	0.625829	1.784341	1.197362
H	-3.7639	1.495059	1.930011	-3.68808	1.698874	1.782212	-3.66865	1.724564	1.793814
H	-2.6336	2.584444	1.124501	-2.4749	2.705638	0.99263	-2.4604	2.712404	0.971011
H	-3.67937	1.499638	0.186529	-3.53472	1.650596	0.041151	-3.54184	1.645794	0.054378
H	-1.09825	1.662885	2.842452	-1.02067	1.792871	2.753433	-1.01507	1.803115	2.753454
H	-2.30423	0.480607	3.335678	-2.29407	0.698761	3.280789	-2.28477	0.70697	3.285978
H	-0.68205	-0.05064	2.934038	-0.69361	0.067724	2.93664	-0.68354	0.079148	2.939013
H	4.005827	2.267973	-1.10695	4.611603	0.674329	-0.13581	4.613322	0.667812	-0.1477
H	3.394286	2.745638	0.467716	4.133426	1.867153	-1.36296	4.133804	1.858848	-1.37606
H	2.093634	-3.26848	0.08067	1.985078	-3.42025	0.356012	1.981533	-3.42122	0.360305
H	1.794505	-2.28326	1.537033	1.744385	-2.2977	1.71967	1.746237	-2.29522	1.72205
H	0.163595	-2.62154	-1.69509	0.060617	-2.8928	-1.44014	0.054749	-2.89569	-1.43171
H	0.554798	-1.06597	-2.45421	0.469214	-1.42475	-2.35165	0.460434	-1.42925	-2.34726
H	1.824816	-2.22712	-2.11987	1.720203	-2.58017	-1.93278	1.712707	-2.58415	-1.93044
H	4.857648	0.338928	0.239898	4.629634	2.992517	0.51743	4.637554	2.987118	0.50065

Table S 6. Energy analysis for 2*S*, 3*S*, 5*R*, 6*R*, 13*S*, 14*S*-2

Conf.	Steric Energy	Relative Energy	Distribution
	(kJ/mol)	(kJ/mol)	(%)
2a-1	-1005.203021	0	45.05%
2a-2	-1005.202593	0.000428	28.64%
2a-3	-1005.202082	0.000939	16.67%
2a-4	-1005.201077	0.001944	5.75%
2a-5	-1005.200349	0.002672	2.66%
2a-6	-1005.199614	0.003407	1.22%

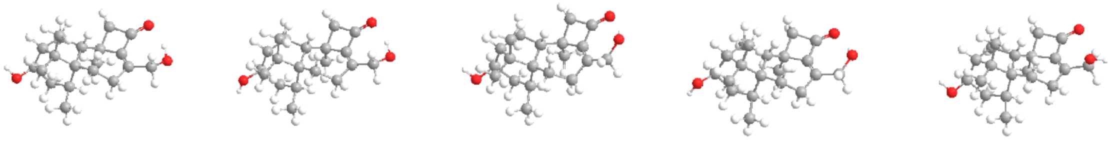

2b-1 2b-2 2b-3 2b-4 2b-5

Figure S 76. Most stable conformers of 2*R*, 3*R*, 5*S*, 6*S*, 13*R*, 14*R*-2 calculated at MPW1PW91/6-31G+d, p level

Table S 7. Optimized Z-Matrixes of 2*R*, 3*R*, 5*S*, 6*S*, 13*R*, 14*R*-2 conformations in the Chloroform at CAM-B3LYP/DGDZVP level

	2b-1			2b-2			2b-3		
C	2.259635	-0.448124	-1.71081	2.260252	-0.456093	-1.697725	2.387916	-0.71561	-1.543321
C	3.190803	-1.063393	-0.664223	3.195551	-1.066618	-0.642741	3.218086	-1.172673	-0.342224
C	2.535692	-1.076324	0.723738	2.53694	-1.073106	0.737725	2.45489	-0.957726	0.971086

C	1.216138	-1.879723	0.615727	1.215462	-1.874684	0.631269	1.13182	-1.758676	0.883297
C	0.105338	-0.813396	0.4597	0.104048	-0.810193	0.466244	0.053856	-0.71954	0.495998
C	0.854209	0.541129	0.237126	0.853069	0.543261	0.239411	0.843056	0.575357	0.109967
C	1.34464	0.705908	-1.239362	1.347264	0.702181	-1.236419	1.445683	0.494052	-1.334049
C	2.070828	0.318786	1.213867	2.068042	0.325607	1.219292	1.984526	0.503014	1.194979
C	1.927931	2.072867	-1.647766	1.936814	2.065497	-1.647743	2.044577	1.784058	-1.925654
O	4.452158	-0.389698	-0.710565	4.472798	-0.425644	-0.567357	4.496974	-0.533183	-0.390362
C	-1.119959	-1.201248	-0.380629	-1.118909	-1.202727	-0.375337	-1.134142	-1.226512	-0.337374
C	-2.299121	-0.229281	-0.214512	-2.298939	-0.230556	-0.217124	-2.329362	-0.257305	-0.289289
C	-2.418853	1.110442	-0.159491	-2.419428	1.109349	-0.168536	-2.421022	1.079898	-0.384373
C	-1.241132	2.05253	-0.257783	-1.241923	2.051724	-0.267283	-1.223061	1.925845	-0.756773
C	-0.040318	1.721381	0.648885	-0.043068	1.724988	0.643565	-0.055176	1.81701	0.249627
C	3.149064	1.413251	1.20503	3.141389	1.42471	1.209001	3.083032	1.571521	1.084093
C	1.659162	0.20812	2.701823	1.651745	0.220468	2.70655	1.471575	0.637843	2.648942
H	-0.34058	-0.690135	1.449998	-0.345203	-0.681805	1.45437	-0.444228	-0.43787	1.427022
C	-3.742829	1.800075	0.08566	-3.744475	1.799568	0.069106	-3.67167	1.878192	-0.116087
C	-2.001119	-2.292169	0.330197	-2.001532	-2.290596	0.338336	-2.0229	-2.251896	0.458094
C	-3.166826	-1.320101	0.2829	-3.167642	-1.319498	0.282727	-3.210289	-1.338144	0.208441
O	-4.338298	-1.413959	0.627242	-4.340243	-1.412525	0.623432	-4.419093	-1.491419	0.310577
C	-0.940239	-1.617626	-1.846257	-0.936069	-1.625909	-1.83866	-0.896996	-1.797537	-1.740819
O	-4.907823	1.111743	-0.303683	-4.908025	1.108937	-0.320483	-4.725251	1.253468	0.57637
H	2.86786	-0.116733	-2.557767	2.855765	-0.133228	-2.558838	3.071645	-0.513851	-2.37311
H	1.636913	-1.256438	-2.097011	1.635844	-1.26918	-2.072739	1.787908	-1.570479	-1.861248
H	3.365074	-2.111878	-0.945716	3.367964	-2.115326	-0.922335	3.386054	-2.25462	-0.441982
H	3.22952	-1.535745	1.438534	3.233505	-1.524832	1.451605	3.078622	-1.303263	1.804551
H	1.043693	-2.481837	1.511296	1.041627	-2.469275	1.531627	0.885587	-2.224664	1.840652
H	1.246826	-2.581663	-0.222442	1.244908	-2.583754	-0.200676	1.208311	-2.573797	0.157676
H	0.440579	0.62441	-1.844177	0.444856	0.621012	-1.843991	0.594924	0.299755	-1.985478
H	1.892233	2.148707	-2.739112	1.903848	2.138401	-2.739418	2.195105	1.629492	-2.998711
H	1.359282	2.916214	-1.249439	1.370542	2.912119	-1.253065	1.375634	2.641494	-1.820496
H	2.968715	2.204387	-1.355842	2.977282	2.193764	-1.353006	3.009565	2.05998	-1.504233
H	4.977362	-0.694802	0.041061	4.889746	-0.513613	-1.433965	4.955561	-0.726594	0.438015
H	-0.940373	2.128446	-1.308049	-0.938449	2.124151	-1.317033	-0.894974	1.662246	-1.765337
H	-1.588124	3.053945	0.014348	-1.590352	3.0539	0.000207	-1.534643	2.972771	-0.809245
H	-0.4287	1.54234	1.656541	-0.43348	1.550216	1.651196	-0.478576	1.847645	1.258538
H	0.567155	2.62829	0.722069	0.563402	2.632743	0.7141	0.554114	2.719899	0.154621
H	3.911271	1.177553	1.955734	3.891414	1.206549	1.976249	3.782411	1.46216	1.920122
H	2.712953	2.379713	1.479145	2.695587	2.394244	1.455845	2.647112	2.573747	1.15464
H	3.664697	1.516879	0.25839	3.674992	1.509852	0.270676	3.670566	1.505908	0.176358
H	0.900814	-0.545417	2.917241	0.889942	-0.529332	2.922948	0.689555	-0.06803	2.930973
H	2.543269	-0.056033	3.290444	2.533459	-0.044915	3.298055	2.310002	0.468844	3.331768
H	1.295896	1.167373	3.081811	1.290957	1.182409	3.082298	1.098636	1.64754	2.84375
H	-3.772324	2.082963	1.15043	-3.777461	2.087702	1.132354	-3.38744	2.746439	0.486115

H	-3.740525	2.735177	-0.480436	-3.740764	2.731851	-0.501618	-4.006336	2.27594	-1.087813
H	-2.145535	-3.23521	-0.201596	-2.143756	-3.236562	-0.188834	-2.096651	-3.267892	0.064892
H	-1.709962	-2.504394	1.362914	-1.713262	-2.497086	1.373027	-1.790644	-2.292819	1.526498
H	-0.597599	-0.806467	-2.487617	-0.59033	-0.818094	-2.482634	-0.565475	-1.051723	-2.462169
H	-0.242358	-2.455371	-1.931515	-0.239571	-2.465367	-1.918347	-0.164983	-2.609418	-1.712518
H	-1.899764	-1.95605	-2.249924	-1.895111	-1.964446	-2.243415	-1.829543	-2.219474	-2.128794
H	-4.924824	0.238448	0.133822	-4.925691	0.23751	0.120673	-4.855263	0.342677	0.254916
	2b-4			2b-5					
C	2.385241	-0.72001	-1.5303	2.331097	-0.75462	-1.52461			
C	3.221062	-1.17078	-0.32253	3.220333	-1.08458	-0.32415			
C	2.456829	-0.95163	0.983126	2.482692	-0.83556	0.998432			
C	1.132028	-1.75119	0.898316	1.205777	-1.71144	1.000607			
C	0.053138	-0.71579	0.503005	0.063	-0.76479	0.560614			
C	0.841643	0.57799	0.112805	0.767928	0.551198	0.090151			
C	1.446182	0.492201	-1.32996	1.338482	0.420429	-1.36108			
C	1.982714	0.510969	1.198439	1.932682	0.605297	1.150742			
C	2.051209	1.778424	-1.92342	1.877766	1.69849	-2.03162			
O	4.505661	-0.54441	-0.24502	4.455094	-0.37265	-0.45054			
C	-1.13242	-1.22799	-0.33066	-1.09047	-1.39777	-0.23458			
C	-2.32866	-0.25957	-0.2913	-2.3201	-0.4847	-0.32418			
C	-2.42171	1.077106	-0.39226	-2.49561	0.833222	-0.50316			
C	-1.2238	1.923795	-0.7633	-1.36676	1.790126	-0.80684			
C	-0.05825	1.819171	0.246179	-0.19398	1.747777	0.192499			
C	3.076414	1.583508	1.082523	2.965265	1.726989	0.959364			
C	1.467551	0.651111	2.651195	1.437937	0.792699	2.605297			
H	-0.44787	-0.42983	1.431165	-0.44967	-0.45762	1.475606			
C	-3.67467	1.874622	-0.13225	-3.87001	1.437068	-0.38711			
C	-2.02288	-2.24984	0.467529	-1.9581	-2.36829	0.649024			
C	-3.21037	-1.33916	0.207867	-3.18256	-1.53431	0.28499			
O	-4.41941	-1.49388	0.304724	-4.37816	-1.69721	0.442412			
C	-0.89213	-1.8068	-1.73042	-0.80545	-2.10229	-1.5675			
O	-4.73031	1.251128	0.558238	-3.83074	2.458473	0.609434			
H	3.055281	-0.52872	-2.37538	2.97665	-0.5772	-2.38997			
H	1.781706	-1.57811	-1.83468	1.768486	-1.65908	-1.76112			
H	3.387637	-2.2524	-0.42104	3.452722	-2.15854	-0.36393			
H	3.085689	-1.28929	1.813359	3.149379	-1.09476	1.830234			
H	0.88552	-2.20858	1.859788	1.004187	-2.11604	1.995616			
H	1.206157	-2.57288	0.180192	1.315514	-2.5742	0.337244			
H	0.596391	0.298495	-1.98309	0.477483	0.157223	-1.97666			
H	2.197956	1.622893	-2.99692	1.937723	1.520523	-3.11004			
H	1.387806	2.639893	-1.81647	1.223902	2.561372	-1.88613			
H	3.018765	2.048212	-1.5039	2.876999	1.976939	-1.7005			
H	4.99388	-0.79145	-1.04083	4.94508	-0.48211	0.375158			

H	-0.89267	1.658698	-1.7705	-1.01678	1.609669	-1.82837			
H	-1.53676	2.970138	-0.81891	-1.77452	2.803416	-0.80211			
H	-0.48394	1.852673	1.25404	-0.61923	1.781785	1.200326			
H	0.549854	2.72267	0.149607	0.371243	2.677229	0.076236			
H	3.763901	1.495059	1.930011	3.688079	1.698874	1.782212			
H	2.633602	2.584444	1.124501	2.4749	2.705638	0.99263			
H	3.679365	1.499638	0.186529	3.534724	1.650596	0.041151			
H	0.682053	-0.05064	2.934038	0.693607	0.067724	2.93664			
H	2.304228	0.480607	3.335678	2.294074	0.698761	3.280789			
H	1.09825	1.662885	2.842452	1.020671	1.792871	2.753433			
H	-3.39429	2.745638	0.467716	-4.13343	1.867153	-1.36296			
H	-4.00583	2.267973	-1.10695	-4.6116	0.674329	-0.13581			
H	-2.09363	-3.26848	0.08067	-1.98508	-3.42025	0.356012			
H	-1.79451	-2.28326	1.537033	-1.74439	-2.2977	1.71967			
H	-0.5548	-1.06597	-2.45421	-0.46921	-1.42475	-2.35165			
H	-0.1636	-2.62154	-1.69509	-0.06062	-2.8928	-1.44014			
H	-1.82482	-2.22712	-2.11987	-1.7202	-2.58017	-1.93278			
H	-4.85765	0.338928	0.239898	-4.62963	2.992517	0.51743			

Table S 8. Energy analysis for 2*R*, 3*R*, 5*S*, 6*S*, 13*R*, 14*R*-2

Conf.	Steric Energy	Relative Energy	Distribution
	(kJ/mol)	(kJ/mol)	(%)
2b-1	-1005.203021	0	45.61%
2b-2	-1005.202593	0.000428	28.99%
2b-3	-1005.202082	0.000939	16.88%
2b-4	-1005.201077	0.001944	5.82%
2b-5	-1005.200349	0.002672	2.69%

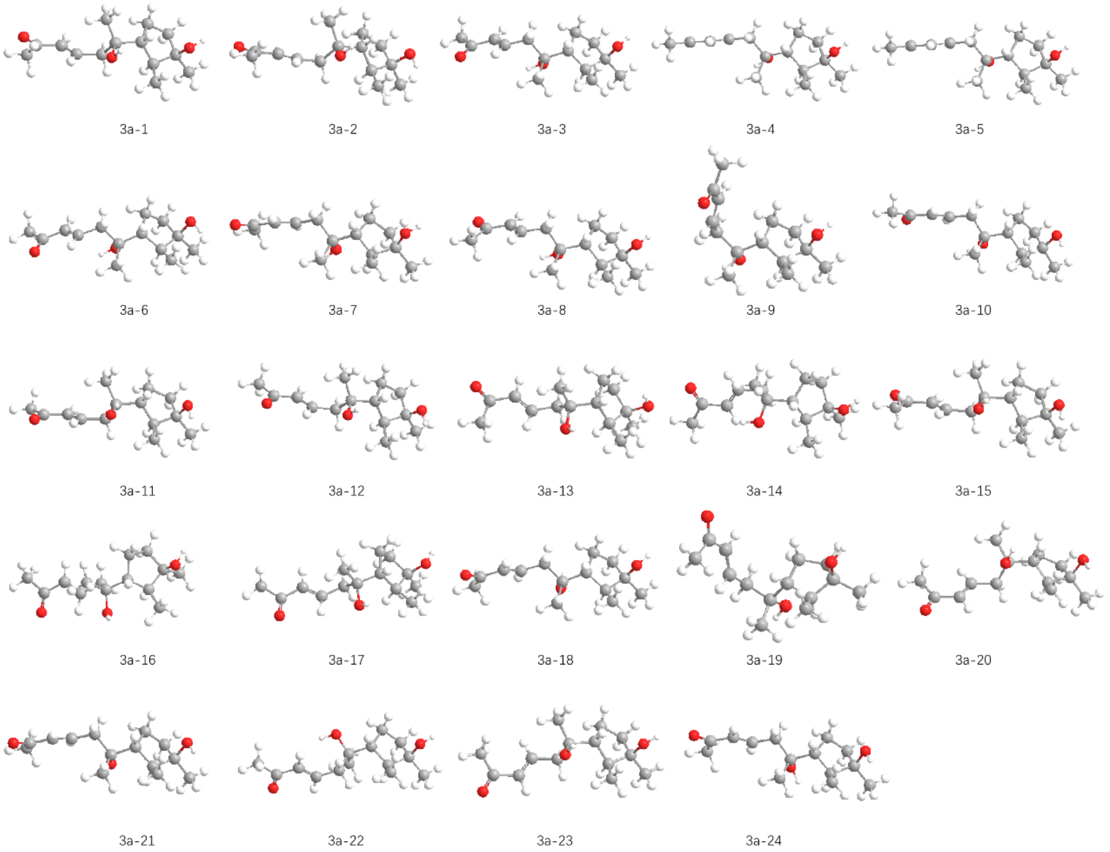

Figure S 77. Most stable conformers of 2*S*, 3*R*, 6*R*, 7*R*-3 calculated at MPW1PW91/6-31G+d, p level

Table S 9. Optimized Z-Matrixes of 2*S*, 3*R*, 6*R*, 7*R*-3 conformations in the Chloroform at MPW1PW91/6-31G+d, p level

	3a-1			3a-2			3a-3		
C	-1.3394	0.204209	0.695126	-1.33723	0.159075	0.695822	1.237076	0.251515	-0.63225
C	-2.12583	0.345748	-0.62383	-2.11223	0.325212	-0.62372	2.408456	0.606817	0.32706
C	-3.40475	-0.46413	-0.34274	-3.42567	-0.44015	-0.34118	3.403718	-0.56492	0.214486
C	-2.85664	-1.71612	0.355709	-2.94242	-1.69129	0.400258	2.480303	-1.76369	-0.0005
C	-1.6325	-1.24412	1.165156	-1.63711	-1.29645	1.123251	1.432628	-1.24879	-0.98736
C	0.156944	0.586349	0.686233	0.157774	0.544315	0.708651	-0.16734	0.555997	-0.06487
C	0.918454	-0.08302	-0.47101	0.924262	-0.07106	-0.47497	-1.21955	0.33945	-1.19149
C	2.392854	0.171487	-0.43286	2.397622	0.187608	-0.42204	-2.62901	0.533119	-0.72487
C	3.330103	-0.78575	-0.43299	3.338365	-0.76505	-0.4673	-3.43437	-0.46081	-0.32506
C	4.788569	-0.53289	-0.39938	4.796002	-0.50911	-0.42026	-4.81808	-0.20598	0.163431
O	5.567705	-1.48057	-0.40262	5.578305	-1.45243	-0.4764	-5.27736	0.924756	0.261294
C	5.299026	0.890161	-0.35619	5.301543	0.911121	-0.29831	-5.62471	-1.42692	0.532059
C	0.790044	0.246776	2.041138	0.789079	0.145279	2.047543	-0.28167	1.98144	0.485291
H	-1.81283	0.884457	1.415667	-1.82033	0.819841	1.429015	1.33944	0.855841	-1.53988
O	-4.18469	0.31315	0.579803	-4.2413	0.282914	0.595471	4.184645	-0.3384	-0.97077
C	-2.36464	1.772657	-1.10501	-2.29752	1.759828	-1.10769	3.045218	1.9711	0.069303
O	0.308081	1.994911	0.450428	0.303212	1.962815	0.533993	-0.38495	-0.38071	0.993023
C	-4.23441	-0.7811	-1.5818	-4.2411	-0.76112	-1.58913	4.326123	-0.70802	1.420143
H	-1.60002	-0.20896	-1.41175	-1.60095	-0.24594	-1.40987	2.030951	0.576364	1.355437

H	-2.55721	-2.4471	-0.40132	-3.72004	-2.04266	1.081446	2.005629	-2.02697	0.949877
H	-3.62235	-2.19274	0.97497	-2.75606	-2.48913	-0.32318	3.023059	-2.64467	-0.35976
H	-1.81794	-1.27071	2.240663	-1.7261	-1.3776	2.208246	1.801746	-1.34163	-2.01118
H	-0.7834	-1.90694	0.979244	-0.82703	-1.96794	0.824544	0.509707	-1.82538	-0.91456
H	0.529562	0.331899	-1.40711	0.535514	0.38122	-1.39373	-1.10301	-0.67082	-1.58789
H	0.721596	-1.15804	-0.47689	0.732405	-1.14562	-0.52731	-1.00317	1.0446	-1.99988
H	2.685592	1.219127	-0.40543	2.686104	1.233844	-0.34359	-3.02118	1.54734	-0.67514
H	3.0515	-1.83736	-0.45471	3.063672	-1.8153	-0.5411	-3.09781	-1.49493	-0.36557
H	4.916988	1.409876	0.526022	4.913004	1.382111	0.608053	-5.10749	-1.99123	1.313418
H	6.387682	0.88064	-0.32648	6.390073	0.90326	-0.26328	-6.61666	-1.14056	0.878523
H	4.965893	1.44619	-1.23596	4.971312	1.51202	-1.14923	-5.71123	-2.08868	-0.33454
H	1.794812	0.671731	2.10275	1.792579	0.569576	2.131223	-1.28874	2.169403	0.869931
H	0.189789	0.666168	2.855277	0.185962	0.522857	2.879877	0.413301	2.133813	1.312512
H	0.867437	-0.83153	2.198973	0.868643	-0.93931	2.152857	-0.07405	2.72397	-0.29007
H	-4.94859	-0.21873	0.838144	-4.54101	1.094969	0.16545	4.706517	-1.13588	-1.1299
H	-2.99364	1.785723	-2.00014	-2.95979	1.804787	-1.97827	3.882668	2.15215	0.749886
H	-1.41843	2.259944	-1.34563	-1.34084	2.202089	-1.3886	2.325006	2.779648	0.212286
H	-2.86429	2.362633	-0.33225	-2.71975	2.391852	-0.31904	3.424838	2.031386	-0.95315
H	-0.17889	2.465578	1.140123	-0.16109	2.400616	1.259951	-1.26152	-0.21522	1.367386
H	-5.07264	-1.43738	-1.32376	-5.10263	-1.37935	-1.32303	4.987985	-1.57165	1.295337
H	-4.64432	0.133563	-2.01702	-4.61535	0.150235	-2.06706	4.951401	0.180958	1.537906
H	-3.63214	-1.28955	-2.3396	-3.63835	-1.30217	-2.32349	3.751113	-0.85494	2.338334
	3a-4			3a-5			3a-6		
C	1.224478	0.086961	-0.56136	1.210914	0.102755	-0.54123	1.235598	0.249759	-0.6304
C	2.381824	0.709288	0.268607	2.37907	0.686041	0.304375	2.404191	0.595852	0.334399
C	3.58213	-0.23809	0.075305	3.57613	-0.25336	0.05326	3.406578	-0.5776	0.20697
C	2.909668	-1.60758	-0.01564	2.899421	-1.6164	-0.0912	2.490385	-1.77148	-0.02852
C	1.685376	-1.35939	-0.89682	1.663047	-1.32768	-0.94312	1.43279	-1.24695	-1.00032
C	-0.15229	0.125288	0.137449	-0.17065	0.114031	0.162466	-0.16976	0.551156	-0.06417
C	-1.24141	-0.32851	-0.87287	-1.25666	-0.30569	-0.84958	-1.22063	0.337356	-1.19251
C	-2.58711	-0.53329	-0.2556	-2.62344	-0.41422	-0.24903	-2.63024	0.532992	-0.72703
C	-3.69624	0.146964	-0.5721	-3.71382	0.205306	-0.72053	-3.43643	-0.45911	-0.32441
C	-5.00009	-0.1243	0.091446	-5.06755	0.082308	-0.13357	-4.81973	-0.20093	0.163729
O	-5.12213	-0.96167	0.977111	-6.00651	0.683641	-0.64538	-5.2774	0.93079	0.257823
C	-6.17124	0.698206	-0.38915	-5.27512	-0.77974	1.091964	-5.62772	-1.41942	0.537178
C	-0.50445	1.516803	0.667575	-0.52036	1.484766	0.747117	-0.2847	1.975106	0.490022
H	1.116486	0.657196	-1.49042	1.107706	0.718014	-1.44158	1.339598	0.861927	-1.53281
O	4.176175	0.094631	-1.19068	4.150253	0.137966	-1.20314	4.196488	-0.45422	-0.98696
C	2.696604	2.165303	-0.06901	2.689549	2.15635	0.030776	3.037623	1.96455	0.090382
O	-0.0782	-0.80114	1.228829	-0.21705	-0.87386	1.202347	-0.38742	-0.38845	0.990717
C	4.629927	-0.1404	1.178734	4.639162	-0.20591	1.145321	4.321909	-0.73497	1.416219
H	2.116564	0.636794	1.329695	2.144216	0.57728	1.372369	2.026507	0.558922	1.362561
H	2.595129	-1.92047	0.984803	2.601863	-1.97738	0.899253	2.022942	-2.05087	0.920318

H	3.58391	-2.37508	-0.41085	3.566905	-2.36623	-0.52867	3.044385	-2.63396	-0.40813
H	1.960126	-1.4247	-1.95198	1.921394	-1.35241	-2.00381	1.792414	-1.33161	-2.02807
H	0.911621	-2.10544	-0.71049	0.888516	-2.07661	-0.77242	0.511362	-1.82563	-0.92377
H	-0.92882	-1.2797	-1.31474	-0.99255	-1.28669	-1.25808	-1.10502	-0.67279	-1.58935
H	-1.30172	0.406218	-1.68115	-1.26107	0.40331	-1.6816	-1.00223	1.042774	-2.00012
H	-2.67168	-1.30567	0.50784	-2.69835	-1.05509	0.626588	-3.0219	1.547545	-0.68019
H	-3.67307	0.922883	-1.33476	-3.65336	0.854359	-1.59166	-3.10108	-1.49373	-0.36207
H	-7.07606	0.43017	0.154422	-4.64871	-0.43373	1.917855	-5.11114	-1.9811	1.320859
H	-5.95838	1.762359	-0.2521	-6.32201	-0.73577	1.389204	-6.61934	-1.13051	0.882476
H	-6.32179	0.538325	-1.46071	-5.00141	-1.817	0.883599	-5.71492	-2.08468	-0.32665
H	-1.50879	1.519044	1.104122	-1.51478	1.458352	1.19957	-1.29363	2.164581	0.868766
H	0.198511	1.830584	1.441614	0.190459	1.782312	1.522915	0.4047	2.122133	1.322929
H	-0.49347	2.257287	-0.1364	-0.51715	2.255021	-0.02877	-0.06992	2.719827	-0.28143
H	4.832158	-0.58596	-1.39059	4.80132	-0.53292	-1.44719	4.798284	0.292689	-0.87226
H	3.536939	2.536428	0.525443	3.537502	2.499804	0.630579	3.900098	2.13023	0.745113
H	1.841506	2.814493	0.131619	1.836559	2.794616	0.270047	2.331515	2.775119	0.279824
H	2.961301	2.267892	-1.12367	2.940327	2.303981	-1.02166	3.369283	2.063452	-0.94806
H	-0.76923	-0.58314	1.867274	0.341385	-0.57407	1.931075	-1.26605	-0.22743	1.362256
H	5.439132	-0.8574	1.003511	5.446985	-0.91112	0.923798	4.973099	-1.6028	1.282023
H	5.068187	0.860574	1.210248	5.075814	0.793483	1.218412	4.957577	0.146951	1.552302
H	4.191579	-0.36085	2.155767	4.216764	-0.47484	2.117428	3.740889	-0.87382	2.331619
	3a-7			3a-8			3a-9		
C	1.219606	0.135363	-0.5739	1.224332	0.218495	-0.63801	-0.6192	0.200182	-0.04335
C	2.374853	0.677192	0.31305	2.381039	0.646073	0.309632	-1.96258	0.045138	0.724409
C	3.546577	-0.30213	0.104952	3.423863	-0.48563	0.222148	-2.78841	-0.96992	-0.0908
C	2.830248	-1.64161	-0.06507	2.550048	-1.72652	0.043101	-2.36284	-0.66744	-1.52776
C	1.63744	-1.31103	-0.96234	1.480577	-1.28155	-0.9542	-0.85089	-0.46444	-1.42789
C	-0.1733	0.19192	0.08958	-0.19035	0.479423	-0.07431	-0.11582	1.654423	-0.14607
C	-1.24999	-0.17177	-0.96959	-1.23537	0.183912	-1.18905	1.270922	1.715257	-0.84312
C	-2.60444	-0.4232	-0.3862	-2.65226	0.361254	-0.73384	2.355127	0.973223	-0.1276
C	-3.68968	0.328064	-0.61746	-3.42059	-0.63806	-0.2758	2.940985	-0.14123	-0.58294
C	-5.03046	0.074172	-0.03993	-4.81083	-0.47842	0.215254	3.995722	-0.84804	0.192127
O	-5.95479	0.835354	-0.30437	-5.4184	-1.4588	0.62978	4.418776	-0.42287	1.260114
C	-5.24148	-1.11674	0.86824	-5.45656	0.88861	0.199508	4.514414	-2.1298	-0.41364
C	-0.49145	1.568032	0.677132	-0.36395	1.914654	0.433563	-0.04039	2.341787	1.220579
H	1.156799	0.749656	-1.47881	1.300682	0.802517	-1.56137	0.153093	-0.35608	0.496771
O	4.183507	0.066047	-1.12978	4.18847	-0.25753	-0.9735	-2.32934	-2.27965	0.283188
C	2.748374	2.135213	0.053192	2.96045	2.029114	0.01863	-1.81054	-0.33848	2.195142
O	-0.16601	-0.7881	1.137691	-0.36313	-0.43303	1.011664	-1.05413	2.349406	-0.97622
C	4.568886	-0.29128	1.236261	4.357288	-0.55949	1.425604	-4.29213	-0.86635	0.139622
H	2.079377	0.564387	1.362682	2.006562	0.624119	1.339289	-2.50741	0.993542	0.657039
H	2.48068	-1.98857	0.912286	2.087794	-1.98326	1.001363	-2.8445	0.258107	-1.85814
H	3.487867	-2.41324	-0.47976	3.127435	-2.59408	-0.29406	-2.65258	-1.46339	-2.2224

H	1.93513	-1.34131	-2.01278	1.851979	-1.38569	-1.97603	-0.33754	-1.42848	-1.4634
H	0.833168	-2.03572	-0.82704	0.582671	-1.89394	-0.86416	-0.47894	0.1363	-2.25866
H	-0.93623	-1.0815	-1.49047	-1.0931	-0.84048	-1.53745	1.557773	2.772567	-0.9098
H	-1.29941	0.630657	-1.71111	-1.03722	0.85512	-2.03024	1.171277	1.344637	-1.86488
H	-2.683	-1.29081	0.266703	-3.05421	1.372221	-0.75633	2.683695	1.357157	0.837017
H	-3.63397	1.198249	-1.26813	-3.04311	-1.65822	-0.2494	2.64314	-0.57248	-1.5367
H	-4.59469	-1.05129	1.746824	-5.49278	1.285383	-0.81808	4.932759	-1.92918	-1.40421
H	-6.28179	-1.14375	1.189299	-6.46906	0.812752	0.593364	5.275988	-2.57498	0.225087
H	-4.99853	-2.0467	0.348361	-4.88384	1.592687	0.80855	3.689154	-2.83431	-0.55215
H	-1.51178	1.589792	1.074476	-1.37591	2.068124	0.82154	0.407753	3.33685	1.12372
H	0.192714	1.814064	1.491702	0.328567	2.122789	1.250522	-1.03908	2.464281	1.643858
H	-0.41982	2.346662	-0.0863	-0.19507	2.642282	-0.36514	0.565184	1.770347	1.928256
H	4.817431	-0.62989	-1.34742	4.736706	-1.03966	-1.11946	-2.74528	-2.91364	-0.31562
H	3.589132	2.445759	0.680796	3.787117	2.262298	0.696493	-2.78618	-0.47565	2.671245
H	1.913586	2.805346	0.269345	2.206625	2.810253	0.139292	-1.27871	0.434226	2.754513
H	3.038152	2.278739	-0.99006	3.341121	2.079735	-1.00388	-1.25418	-1.27306	2.294691
H	-0.83424	-0.54377	1.79059	-1.24748	-0.30353	1.381559	-0.81858	3.286572	-0.9805
H	5.35754	-1.02726	1.047154	5.053853	-1.3979	1.31861	-4.82599	-1.59276	-0.48241
H	5.040113	0.691226	1.324176	4.945803	0.357236	1.517658	-4.53935	-1.07224	1.184498
H	4.098984	-0.54027	2.191557	3.793127	-0.70705	2.350406	-4.66012	0.130704	-0.11676
	3a-10			3a-11			3a-12		
C	1.223641	0.094096	-0.56685	-1.35453	0.236113	0.681358	-1.35451	0.23612	0.681347
C	2.380191	0.699644	0.275217	-2.13542	0.257016	-0.6454	-2.13545	0.257044	-0.64539
C	3.581354	-0.25735	0.078142	-3.40947	-0.54886	-0.30198	-3.40944	-0.5489	-0.30195
C	2.904677	-1.61632	-0.04701	-2.86677	-1.70993	0.537279	-2.86666	-1.70999	0.537223
C	1.682141	-1.34749	-0.92506	-1.59466	-1.18727	1.237455	-1.59456	-1.1873	1.237387
C	-0.15463	0.12764	0.128806	0.124254	0.680428	0.653257	0.124255	0.680478	0.653225
C	-1.243	-0.30865	-0.89034	0.914982	-0.01512	-0.46899	0.91497	-0.01503	-0.46905
C	-2.58722	-0.53103	-0.27581	2.379904	0.285621	-0.43794	2.379898	0.285676	-0.43798
C	-3.69548	0.163079	-0.56395	3.350007	-0.6365	-0.39882	3.349974	-0.63647	-0.39883
C	-4.99752	-0.12691	0.095557	4.789382	-0.26447	-0.37738	4.789359	-0.26449	-0.37736
O	-5.11878	-0.99514	0.951026	5.166772	0.900859	-0.3927	5.16679	0.900822	-0.39273
C	-6.16737	0.71742	-0.34893	5.768942	-1.41332	-0.33248	5.768881	-1.41337	-0.3324
C	-0.50277	1.514222	0.674982	0.769042	0.436951	2.022732	0.769069	0.436973	2.022678
H	1.11815	0.678827	-1.48736	-1.86219	0.939963	1.355326	-1.86218	0.939919	1.355363
O	4.223916	-0.02519	-1.18601	-4.26501	0.204374	0.572832	-4.265	0.204256	0.572928
C	2.703154	2.156772	-0.05104	-2.39219	1.638507	-1.23938	-2.39229	1.638543	-1.23932
O	-0.08514	-0.81201	1.208545	0.212097	2.079276	0.343135	0.212063	2.079344	0.343149
C	4.610771	-0.18568	1.200573	-4.2022	-1.00964	-1.52034	-4.2022	-1.00965	-1.52031
H	2.109126	0.625637	1.334693	-1.59493	-0.34683	-1.38616	-1.59496	-0.34675	-1.38618
H	2.586857	-1.9459	0.946462	-2.62642	-2.5478	-0.12235	-2.62628	-2.54781	-0.12246
H	3.58394	-2.36643	-0.46015	-3.63136	-2.05338	1.23716	-3.63121	-2.05352	1.237109
H	1.95984	-1.39757	-1.98006	-1.70001	-1.17102	2.323943	-1.69988	-1.1711	2.323879

H	0.90508	-2.09375	-0.75307	-0.74952	-1.84504	1.015251	-0.74939	-1.84501	1.015128
H	-0.92774	-1.24877	-1.35326	0.518072	0.345948	-1.42413	0.518065	0.346098	-1.42418
H	-1.30651	0.443624	-1.68207	0.749885	-1.09494	-0.42867	0.749854	-1.09485	-0.42879
H	-2.67098	-1.32899	0.460888	2.66692	1.335444	-0.45192	2.666943	1.33549	-0.45199
H	-3.67311	0.964672	-1.29962	3.107113	-1.69723	-0.37805	3.107041	-1.69719	-0.37804
H	-7.07189	0.429614	0.184912	5.620019	-2.06243	-1.20022	5.619988	-2.06248	-1.20015
H	-5.95126	1.774341	-0.16781	6.793148	-1.04296	-0.32063	6.793096	-1.04304	-0.3205
H	-6.32028	0.60291	-1.42591	5.585712	-2.02261	0.557359	5.585586	-2.02266	0.557425
H	-1.51117	1.517318	1.101906	1.754858	0.906332	2.062923	1.75489	0.906339	2.062853
H	0.194261	1.81176	1.460802	0.150079	0.868881	2.816289	0.150128	0.8689	2.816254
H	-0.47873	2.265904	-0.11838	0.889844	-0.62862	2.232175	0.889859	-0.6286	2.232106
H	4.673113	0.828857	-1.141	-4.60309	0.962589	0.077972	-4.60313	0.962483	0.078125
H	3.574151	2.511143	0.51111	-3.05642	1.582756	-2.10805	-3.0565	1.582787	-2.108
H	1.871393	2.818705	0.197559	-1.45841	2.103901	-1.55719	-1.45852	2.103996	-1.55709
H	2.9084	2.283901	-1.11871	-2.84511	2.308463	-0.50055	-2.84525	2.308453	-0.50046
H	-0.78502	-0.60804	1.84212	-0.24446	2.567575	1.041183	-0.24453	2.567609	1.041197
H	5.411246	-0.90821	1.020887	-5.03372	-1.64595	-1.20582	-5.03369	-1.646	-1.20578
H	5.061863	0.810679	1.264527	-4.61819	-0.15876	-2.06977	-4.61823	-0.15876	-2.06969
H	4.151606	-0.40812	2.167265	-3.57035	-1.5776	-2.2085	-3.57036	-1.57756	-2.20851
	3a-13			3a-14			3a-15		
C	-1.39138	0.586369	0.750787	1.413726	0.898222	0.419081	-1.3372	0.159248	0.695815
C	-2.21897	0.457739	-0.55965	1.976421	-0.54105	0.587534	-2.1123	0.325305	-0.62368
C	-3.16723	-0.74909	-0.34316	3.107811	-0.66952	-0.45298	-3.42561	-0.44031	-0.34114
C	-2.34643	-1.66086	0.574804	2.615945	0.221321	-1.59783	-2.94208	-1.6914	0.400175
C	-1.69932	-0.69601	1.571746	2.05384	1.449732	-0.88096	-1.637	-1.29625	1.123411
C	0.118578	0.853972	0.527634	-0.12768	0.990769	0.456016	0.157794	0.544518	0.708508
C	0.835402	-0.34089	-0.12972	-0.78203	0.289653	-0.76701	0.924245	-0.07109	-0.47502
C	2.284313	-0.07819	-0.40194	-2.27767	0.346658	-0.7327	2.39761	0.187561	-0.42214
C	3.289421	-0.84525	0.040599	-3.06271	-0.64247	-0.2843	3.338333	-0.76513	-0.46716
C	4.722457	-0.59247	-0.23454	-4.54592	-0.51217	-0.24202	4.795978	-0.50922	-0.42015
O	5.569156	-1.35363	0.222082	-5.12029	0.51155	-0.58991	5.578259	-1.45257	-0.47603
C	5.123389	0.601677	-1.07148	-5.30248	-1.7189	0.256347	5.301545	0.911041	-0.29856
C	0.791467	1.225618	1.85302	-0.56576	2.457561	0.546293	0.789141	0.145746	2.047462
H	-1.75823	1.459162	1.306862	1.755575	1.497381	1.269227	-1.82026	0.820058	1.429002
O	-4.38574	-0.35704	0.307828	4.284092	-0.09127	0.134359	-4.24132	0.2825	0.595629
C	-2.93049	1.741202	-0.97783	2.39519	-0.87724	2.015685	-2.29788	1.759914	-1.10755
O	0.280588	1.935691	-0.40052	-0.5178	0.310658	1.652436	0.303225	1.962982	0.533578
C	-3.60236	-1.41976	-1.63573	3.393833	-2.10705	-0.87317	-4.24105	-0.76135	-1.58906
H	-1.54461	0.178163	-1.37519	1.208121	-1.26245	0.291521	-1.60096	-0.2457	-1.40989
H	-1.57517	-2.16663	-0.01623	1.826777	-0.30239	-2.14905	-2.75534	-2.48903	-0.3234
H	-2.96846	-2.42676	1.044639	3.412275	0.458523	-2.31114	-3.71968	-2.04318	1.081179
H	-2.39463	-0.46614	2.385609	2.869697	2.128622	-0.62469	-1.72628	-1.3772	2.208399
H	-0.8194	-1.1277	2.05301	1.360394	2.014718	-1.50824	-0.82677	-1.96771	0.825064

H	0.348814	-0.55359	-1.08784	-0.45836	-0.75327	-0.78454	0.535485	0.381045	-1.39385
H	0.730755	-1.2293	0.496632	-0.43091	0.768484	-1.68541	0.732366	-1.14566	-0.52717
H	2.49522	0.802274	-1.00443	-2.76295	1.263049	-1.06401	2.686117	1.233809	-0.34395
H	3.09284	-1.72401	0.651212	-2.63014	-1.58313	0.050926	3.063618	-1.81539	-0.54069
H	4.769091	1.529373	-0.61534	-4.96933	-1.9753	1.266148	4.913054	1.382245	0.607708
H	6.208735	0.6324	-1.15666	-6.37405	-1.52486	0.259084	6.390077	0.903173	-0.26358
H	4.683524	0.536763	-2.06972	-5.0838	-2.58127	-0.38005	4.971284	1.511748	-1.14961
H	1.834448	1.504702	1.685801	-1.64572	2.537124	0.703454	1.792652	0.570042	2.131005
H	0.277969	2.075551	2.314185	-0.06903	2.939138	1.391942	0.186062	0.523541	2.879727
H	0.772564	0.397024	2.565041	-0.32081	3.013039	-0.36243	0.86868	-0.93883	2.153022
H	-4.169	0.095521	1.133471	4.966001	-0.06119	-0.54952	-4.54143	1.094405	0.165605
H	-3.55399	1.577849	-1.86146	2.807666	-1.88898	2.081113	-2.96013	1.804788	-1.97815
H	-2.20095	2.516997	-1.22151	1.529079	-0.81629	2.677412	-1.34128	2.202386	-1.38842
H	-3.57846	2.118483	-0.18158	3.156933	-0.18015	2.372084	-2.72028	2.391791	-0.31887
H	-0.07243	2.736294	0.010115	-1.48153	0.362201	1.727087	-0.16129	2.400931	1.259312
H	-4.2497	-2.27325	-1.41779	4.190786	-2.13808	-1.62398	-5.10241	-1.37982	-1.32297
H	-4.16003	-0.72195	-2.266	3.714933	-2.70236	-0.01428	-4.61556	0.149957	-2.06686
H	-2.73397	-1.77576	-2.19516	2.505109	-2.57314	-1.30763	-3.63823	-1.30217	-2.32352
	3a-16			3a-17			3a-18		
C	-1.41245	0.621429	0.724183	-1.41259	0.621776	0.724147	1.209484	0.105218	-0.56203
C	-2.25768	0.413894	-0.56308	-2.25767	0.413825	-0.56316	2.36661	0.698088	0.290276
C	-3.14982	-0.81286	-0.28386	-3.14963	-0.81302	-0.28386	3.56985	-0.24123	0.073372
C	-2.27563	-1.66192	0.644082	-2.2756	-1.66164	0.644659	2.899523	-1.60725	-0.0809
C	-1.66197	-0.63601	1.597582	-1.6623	-0.63532	1.597948	1.693518	-1.31394	-0.97426
C	0.081666	0.932972	0.458639	0.081571	0.933114	0.458601	-0.16562	0.103518	0.154386
C	0.829497	-0.25907	-0.16903	0.829486	-0.25921	-0.1683	-1.2635	-0.34648	-0.84249
C	2.268779	0.03133	-0.45724	2.268698	0.031191	-0.45688	-2.6273	-0.41985	-0.23047
C	3.298698	-0.70776	-0.02568	3.298727	-0.70782	-0.02545	-3.72252	0.162703	-0.73626
C	4.706315	-0.36824	-0.36345	4.706278	-0.36835	-0.36358	-5.07153	0.081104	-0.13073
O	5.001798	0.606921	-1.04341	5.001579	0.606289	-1.04436	-6.01363	0.648843	-0.67378
C	5.761669	-1.29996	0.183926	5.761792	-1.29949	0.184482	-5.26963	-0.69766	1.15071
C	0.767638	1.377602	1.754895	0.76739	1.378261	1.754771	-0.52623	1.475269	0.718062
H	-1.80424	1.499304	1.252716	-1.80432	1.499895	1.252328	1.089	0.716334	-1.46284
O	-4.29775	-0.33921	0.438012	-4.2979	-0.33938	0.437454	4.177721	0.137928	-1.17136
C	-3.02551	1.654251	-1.01157	-3.02555	1.654018	-1.01203	2.680682	2.164146	-0.00036
O	0.185949	1.98339	-0.51247	0.185975	1.983066	-0.51299	-0.14999	-0.77134	1.290438
C	-3.59747	-1.54452	-1.54422	-3.5967	-1.54515	-1.54415	4.602932	-0.18757	1.193196
H	-1.59073	0.127151	-1.38244	-1.59055	0.127068	-1.38237	2.096743	0.592872	1.347542
H	-1.4976	-2.15781	0.05297	-1.49738	-2.15769	0.053932	2.578732	-1.96135	0.904934
H	-2.84871	-2.44311	1.154192	-2.84875	-2.4427	1.154898	3.577658	-2.35865	-0.4982
H	-2.37756	-0.39711	2.386893	-2.37824	-0.39617	2.386864	2.00262	-1.3052	-2.02095
H	-0.76217	-1.01223	2.090031	-0.76264	-1.01122	2.090902	0.928401	-2.09198	-0.89202
H	0.34436	-0.50812	-1.11939	0.344352	-0.50909	-1.11846	-1.01364	-1.34365	-1.22449

H	0.747934	-1.13326	0.480802	0.748149	-1.13296	0.482161	-1.26942	0.32841	-1.70297
H	2.481142	0.906279	-1.06803	2.480898	0.906021	-1.06791	-2.69703	-0.99638	0.689985
H	3.130579	-1.58742	0.592916	3.130775	-1.58731	0.59343	-3.66945	0.750611	-1.65016
H	5.586039	-2.31544	-0.18284	5.586827	-2.31504	-0.18243	-4.63836	-0.29694	1.947652
H	6.755756	-0.9682	-0.11275	6.755875	-0.96729	-0.11172	-6.31453	-0.63526	1.451529
H	5.6944	-1.33821	1.275059	5.693956	-1.33782	1.275571	-4.99623	-1.7463	1.009615
H	1.79403	1.694016	1.554306	1.79394	1.694229	1.554267	-1.51462	1.444286	1.183113
H	0.228914	2.22053	2.200047	0.228841	2.221624	2.199331	0.18988	1.785527	1.480486
H	0.797603	0.57297	2.493821	0.796875	0.574025	2.494147	-0.54535	2.225991	-0.07576
H	-4.81426	-1.11189	0.70243	-4.81416	-1.11211	0.702217	4.859702	-0.5159	-1.3739
H	-3.649	1.441247	-1.88511	-3.64902	1.440742	-1.88552	3.51688	2.51747	0.610303
H	-2.32847	2.449284	-1.28724	-2.32843	2.448941	-1.28787	1.822502	2.80399	0.215552
H	-3.67774	2.020451	-0.21542	-3.67781	2.020452	-0.216	2.95086	2.299683	-1.04979
H	-0.18984	2.784865	-0.12471	-0.1898	2.78475	-0.12567	0.068771	-1.66305	0.987932
H	-4.21088	-2.4146	-1.28676	-4.21014	-2.41519	-1.28666	5.415875	-0.89562	1.000299
H	-4.19461	-0.88614	-2.18065	-4.19363	-0.88702	-2.18104	5.038174	0.812136	1.269831
H	-2.73732	-1.89826	-2.11909	-2.73626	-1.899	-2.11854	4.15244	-0.44677	2.154971
	3a-19			3a-20			3a-21		
C	-0.61534	0.19206	-0.03736	1.360371	0.602084	0.435311	1.218794	0.142992	-0.58072
C	-1.95966	0.052964	0.731723	2.000811	-0.7176	-0.03183	2.37234	0.667546	0.317657
C	-2.8073	-0.93398	-0.09571	3.434479	-0.28008	-0.41132	3.544159	-0.32236	0.106823
C	-2.37777	-0.62095	-1.52925	3.20876	1.052198	-1.13523	2.823328	-1.64905	-0.09514
C	-0.86187	-0.44985	-1.42976	1.909839	1.652748	-0.55632	1.635974	-1.29713	-0.9918
C	-0.08384	1.637312	-0.12142	-0.1718	0.631989	0.631376	-0.17513	0.1956	0.080569
C	1.296916	1.681824	-0.82979	-0.91206	0.016779	-0.57168	-1.25197	-0.15581	-0.98292
C	2.360173	0.864123	-0.16471	-2.39686	0.213183	-0.54202	-2.6045	-0.41872	-0.4
C	2.9407	-0.20886	-0.71844	-3.28471	-0.7678	-0.33584	-3.68938	0.339795	-0.60799
C	3.979905	-1.03712	-0.06354	-4.75335	-0.57973	-0.3083	-5.02727	0.075929	-0.02811
O	4.450536	-1.99854	-0.66159	-5.48423	-1.5475	-0.12506	-5.95096	0.845747	-0.26872
C	4.441921	-0.69014	1.334098	-5.33416	0.803571	-0.50245	-5.2358	-1.135	0.853706
C	0.016322	2.300062	1.256187	-0.62926	2.068437	0.916513	-0.49014	1.568054	0.679061
H	0.147466	-0.3868	0.492998	1.790192	0.828634	1.421181	1.158449	0.771247	-1.47638
O	-2.37449	-2.25716	0.260823	4.196508	0.033918	0.765871	4.225886	-0.05933	-1.13017
C	-1.81171	-0.35484	2.196446	1.924133	-1.87887	0.954228	2.754415	2.125598	0.069539
O	-1.01515	2.3656	-0.93002	-0.54009	-0.19645	1.742425	-0.17039	-0.79298	1.120026
C	-4.30824	-0.80284	0.13897	4.193878	-1.29823	-1.25514	4.547673	-0.33734	1.254611
H	-2.48549	1.012975	0.679588	1.528008	-1.02686	-0.9733	2.069989	0.554121	1.365177
H	-2.84151	0.318251	-1.84656	3.103461	0.866679	-2.2072	2.467369	-2.00941	0.874477
H	-2.68438	-1.40153	-2.23394	4.077284	1.6999	-0.99943	3.486425	-2.40493	-0.52374
H	-0.36717	-1.42278	-1.47856	2.082992	2.61019	-0.06124	1.941613	-1.30959	-2.04019
H	-0.4791	0.153951	-2.25325	1.194693	1.842059	-1.36246	0.828669	-2.02187	-0.8753
H	1.623708	2.729604	-0.84987	-0.68652	-1.05148	-0.60042	-0.93697	-1.05704	-1.51732
H	1.175752	1.364878	-1.86698	-0.51972	0.464963	-1.49087	-1.30458	0.657404	-1.71238

H	2.658774	1.175335	0.834888	-2.75364	1.22749	-0.70995	-2.68162	-1.30104	0.23302
H	2.656677	-0.53979	-1.71517	-2.95255	-1.78965	-0.16755	-3.63546	1.224585	-1.23881
H	3.602753	-0.71349	2.033895	-5.0444	1.211225	-1.47415	-4.58405	-1.09077	1.72996
H	5.196226	-1.40867	1.651688	-6.42032	0.748245	-0.44486	-6.27435	-1.16724	1.179871
H	4.867497	0.316106	1.360985	-4.96524	1.48705	0.266435	-4.99756	-2.0534	0.311615
H	0.479219	3.28948	1.171991	-1.66384	2.071414	1.267522	-1.51257	1.591664	1.070599
H	-0.97596	2.431287	1.69152	-0.00656	2.513535	1.699346	0.190006	1.802717	1.500382
H	0.618272	1.706908	1.949358	-0.55964	2.705986	0.03145	-0.40966	2.35327	-0.07687
H	-2.78986	-2.87403	-0.356	4.334043	-0.78589	1.259011	4.699723	0.77759	-1.03898
H	-2.78861	-0.47882	2.673528	2.50601	-2.73771	0.6042	3.619661	2.422058	0.672347
H	-1.26293	0.398978	2.765482	0.892531	-2.20527	1.092051	1.93901	2.804752	0.325409
H	-1.27483	-1.30231	2.280876	2.298798	-1.58621	1.940965	2.995482	2.294544	-0.98473
H	-0.75323	3.295758	-0.93159	-0.08653	0.140268	2.52676	-0.84754	-0.55987	1.767929
H	-4.85743	-1.51077	-0.49082	5.156668	-0.88275	-1.56441	5.322719	-1.08423	1.063663
H	-4.55765	-1.01691	1.18171	4.389144	-2.21673	-0.69199	5.037053	0.636192	1.36841
H	-4.65663	0.20456	-0.10418	3.627205	-1.56696	-2.15068	4.056946	-0.57885	2.20103
	3a-22			3a-23			1a-24		
C	1.214154	-0.29144	-0.67587	1.360344	0.588356	0.478412	1.2092	0.101924	-0.53562
C	2.075967	0.771426	0.06386	2.02139	-0.73104	0.028264	2.378518	0.673256	0.314921
C	3.482793	0.14781	0.207383	3.407836	-0.27042	-0.45715	3.580821	-0.26534	0.04282
C	3.184225	-1.35126	0.300554	3.073591	0.996472	-1.25539	2.90804	-1.62182	-0.12481
C	2.092821	-1.56507	-0.74745	1.887095	1.650849	-0.52174	1.659757	-1.32374	-0.95662
C	-0.17637	-0.55093	-0.0547	-0.1732	0.592387	0.670323	-0.17281	0.108536	0.167214
C	-0.99149	0.770727	-0.02595	-0.90206	0.001494	-0.5522	-1.25805	-0.29999	-0.85029
C	-2.39861	0.580421	0.448692	-2.38561	0.208722	-0.53919	-2.62575	-0.41272	-0.25263
C	-3.4602	0.448784	-0.35816	-3.28375	-0.76507	-0.34347	-3.7138	0.215743	-0.7176
C	-4.8322	0.226251	0.177347	-4.75083	-0.56369	-0.33263	-5.06867	0.089595	-0.13389
O	-5.05606	0.130869	1.377333	-5.49297	-1.52452	-0.15839	-6.00469	0.701679	-0.63829
C	-5.93404	0.122024	-0.84834	-5.31661	0.824955	-0.53364	-5.28083	-0.78865	1.07924
C	-0.11475	-1.18493	1.339681	-0.64917	2.01462	0.996917	-0.52026	1.474748	0.763934
H	1.011653	0.059399	-1.693	1.780985	0.829113	1.464009	1.105776	0.727672	-1.42886
O	4.187802	0.4236	-1.01266	4.159394	0.073396	0.717537	4.180971	0.029392	-1.22705
C	2.083597	2.147623	-0.59982	2.045061	-1.84681	1.067337	2.685573	2.147725	0.058793
O	-0.80293	-1.46284	-0.96316	-0.53799	-0.27047	1.755573	-0.22246	-0.88863	1.197481
C	4.272556	0.693124	1.392437	4.167803	-1.31179	-1.27066	4.642924	-0.23713	1.136191
H	1.700666	0.890913	1.086981	1.508346	-1.09758	-0.86998	2.147051	0.555099	1.382736
H	2.812721	-1.58348	1.3048	2.786457	0.713377	-2.27269	2.624172	-2.00096	0.862304
H	4.078711	-1.96071	0.1335	3.942536	1.655993	-1.3393	3.580328	-2.34831	-0.5879
H	2.547017	-1.63434	-1.73823	2.185248	2.559341	0.005179	1.905086	-1.33954	-2.02032
H	1.520179	-2.48138	-0.59473	1.119164	1.947144	-1.24125	0.887768	-2.07473	-0.78284
H	-0.99025	1.183906	-1.03828	-0.68374	-1.06778	-0.59153	-0.99415	-1.2772	-1.26779
H	-0.4916	1.484166	0.63536	-0.49407	0.458869	-1.46	-1.26071	0.417111	-1.67536
H	-2.57257	0.51487	1.521378	-2.7325	1.226206	-0.70888	-2.70346	-1.06448	0.614657

H	-3.34557	0.514795	-1.43818	-2.96198	-1.78981	-0.17277	-3.65034	0.875492	-1.58045
H	-5.72395	-0.70308	-1.53514	-5.01307	1.228933	-1.50268	-4.65498	-0.45562	1.910898
H	-6.89564	-0.03785	-0.36282	-6.40377	0.779777	-0.48738	-6.32812	-0.74587	1.375213
H	-5.96896	1.035369	-1.44906	-4.94935	1.505808	0.238362	-5.00947	-1.82368	0.857234
H	-1.11948	-1.4294	1.697017	-1.68462	1.993003	1.344781	-1.51732	1.448351	1.210463
H	0.456412	-2.11483	1.316288	-0.03438	2.44252	1.795426	0.18702	1.760561	1.547436
H	0.340455	-0.51465	2.073247	-0.58678	2.681971	0.133622	-0.50925	2.253087	-0.00393
H	5.016939	-0.07182	-0.98748	5.000708	0.448701	0.42663	4.633616	0.879919	-1.15582
H	2.73923	2.841304	-0.06518	2.595523	-2.71714	0.697747	3.561159	2.479328	0.626947
H	1.081483	2.583371	-0.6134	1.030017	-2.16376	1.311862	1.852081	2.789593	0.349044
H	2.437247	2.077143	-1.63033	2.527789	-1.50782	1.987804	2.875287	2.32705	-1.00389
H	-1.68871	-1.66215	-0.62833	-0.0695	0.030331	2.545775	0.341895	-0.6012	1.926649
H	5.25381	0.210757	1.454261	5.100749	-0.88836	-1.65824	5.438309	-0.94938	0.902169
H	4.431546	1.76977	1.287883	4.421833	-2.17586	-0.65197	5.094836	0.756446	1.227502
H	3.747345	0.507597	2.333428	3.575579	-1.65443	-2.12347	4.214843	-0.50116	2.106862

Table S 10. Energy analysis for 2*S*, 3*R*, 6*R*, 7*R*-3 in the Chloroform at MPW1PW91/6-31G+d, p level

Conf.	Steric Energy (kJ/mol)	Relative Energy (kJ/mol)	Distribution (%)
3a-1	-773.144423	0.000642	8.24%
3a-2	-773.145065	0	16.26%
3a-3	-773.142525	0.00254	1.10%
3a-4	-773.142825	0.00224	1.52%
3a-5	-773.143102	0.001963	2.03%
3a-6	-773.142275	0.00279	0.85%
3a-7	-773.141971	0.003094	0.61%
3a-8	-773.142257	0.002808	0.83%
3a-9	-773.143804	0.001261	4.28%
3a-10	-773.142781	0.002284	1.45%
3a-11	-773.14458	0.000485	9.73%
3a-12	-773.144582	0.000483	9.75%
3a-13	-773.142912	0.002153	1.66%
3a-14	-773.142437	0.002628	1.01%
3a-15	-773.145064	0.000001	16.24%
3a-16	-773.143499	0.001566	3.10%
3a-17	-773.143501	0.001564	3.10%
3a-18	-773.143203	0.001862	2.26%
3a-19	-773.143334	0.001731	2.60%
3a-20	-773.143279	0.001786	2.45%
3a-21	-773.142308	0.002757	0.88%
3a-22	-773.142656	0.002409	1.27%
3a-23	-773.142219	0.002846	0.80%
3a-24	-773.144391	0.000674	7.96%

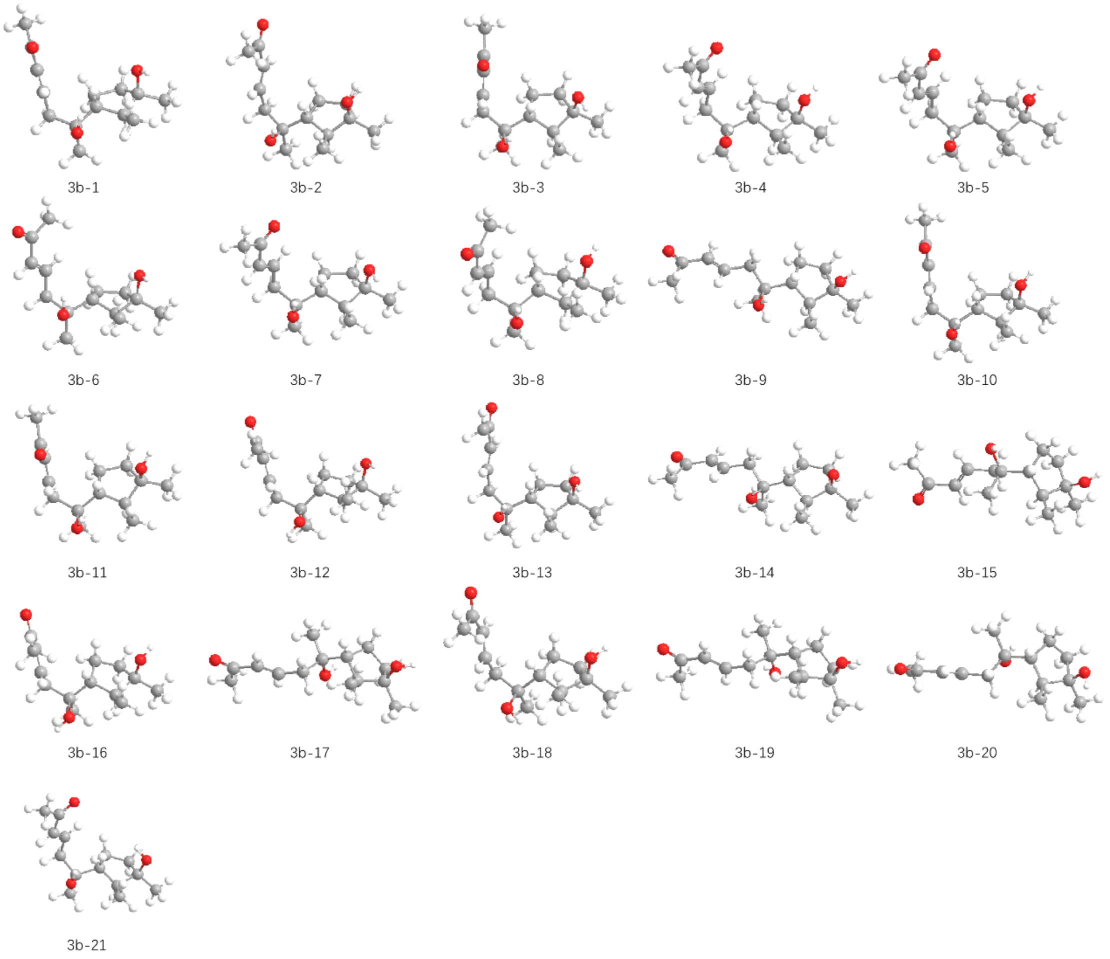

Figure S 78. Most stable conformers of 2*S*, 3*R*, 6*R*, 7*S*-3 calculated at MPW1PW91/6-31G+d, p level

Table S 11. Optimized Z-Matrixes of 2*S*, 3*R*, 6*R*, 7*S*-3 conformations in the Chloroform at MPW1PW91/6-31G+d, p level

	3b-1			3b-2			3b-3		
C	-0.73603	0.345784	-0.1064	-0.72423	0.34669	-0.10106	-0.736	0.345837	-0.1064
C	-2.19835	0.160193	0.343382	-2.1871	0.190853	0.36299	-2.1983	0.160138	0.343427
C	-2.49761	-1.30028	-0.06555	-2.52139	-1.25056	-0.0614	-2.49748	-1.30032	-0.06559
C	-1.85196	-1.40203	-1.44879	-1.9116	-1.33245	-1.468	-1.85194	-1.4019	-1.44888
C	-0.63939	-0.44929	-1.43125	-0.66648	-0.42343	-1.44353	-0.63931	-0.44923	-1.43125
C	-0.19639	1.790365	-0.18104	-0.14583	1.776546	-0.15453	-0.19643	1.790451	-0.18102
C	1.235706	1.805805	-0.77805	1.27001	1.769609	-0.78729	1.235675	1.80594	-0.77799
C	2.214325	0.969712	-0.01704	2.23925	0.873441	-0.08295	2.214254	0.969781	-0.01701
C	2.921312	-0.04866	-0.52237	2.918294	-0.12516	-0.66334	2.921186	-0.04861	-0.52237
C	3.867707	-0.83592	0.312854	3.871232	-1.01593	0.037995	3.867533	-0.83596	0.312831
O	4.056755	-0.58809	1.49745	4.440574	-1.90348	-0.58835	4.056612	-0.58817	1.49743
C	4.581477	-1.96839	-0.38473	4.12335	-0.82537	1.516972	4.581202	-1.96848	-0.38477
C	-1.08429	2.725949	-0.99675	-1.02833	2.756458	-0.92282	-1.08432	2.726044	-0.99673
H	-0.11142	-0.18173	0.628778	-0.10558	-0.21211	0.615598	-0.11134	-0.18164	0.628761
O	-1.78085	-2.22001	0.773301	-1.80892	-2.11348	0.838967	-1.78059	-2.22006	0.773137
C	-2.48834	0.486719	1.805493	-2.46245	0.504495	1.829842	-2.48824	0.486572	1.80557

O	-0.14848	2.375924	1.128311	-0.03793	2.32392	1.169393	-0.1485	2.375967	1.128349
C	-3.98011	-1.65659	-0.06866	-4.00812	-1.58685	-0.03225	-3.97995	-1.65675	-0.0686
H	-2.84548	0.783151	-0.28689	-2.82248	0.833911	-0.25906	-2.8455	0.783085	-0.28678
H	-2.57741	-1.08667	-2.20346	-2.64004	-0.96641	-2.1974	-2.57741	-1.08636	-2.20344
H	-1.58187	-2.43783	-1.66442	-1.67992	-2.36665	-1.73914	-1.58192	-2.43768	-1.66471
H	0.306036	-0.99282	-1.48523	0.259622	-0.99657	-1.51879	0.306085	-0.99282	-1.48515
H	-0.67168	0.214433	-2.29932	-0.68535	0.25909	-2.29706	-0.67148	0.214474	-2.29934
H	1.210672	1.500455	-1.82715	1.213187	1.506328	-1.84634	1.21067	1.500672	-1.82711
H	1.575867	2.847118	-0.74477	1.64454	2.797628	-0.72312	1.575833	2.847251	-0.74461
H	2.371434	1.212666	1.033392	2.39114	1.075587	0.976268	2.37135	1.212682	1.033442
H	2.816915	-0.33334	-1.56744	2.79163	-0.34056	-1.72226	2.816766	-0.33325	-1.56745
H	5.151476	-1.58072	-1.234	4.510173	0.176787	1.71735	3.849168	-2.67502	-0.7866
H	5.249762	-2.48239	0.304708	3.195339	-0.94145	2.082569	5.151121	-1.58088	-1.23413
H	3.849512	-2.67493	-0.78667	4.846633	-1.56546	1.856516	5.249536	-2.48247	0.30462
H	-0.62501	3.715816	-1.05806	-0.54357	3.734773	-0.97348	-0.62499	3.715883	-1.05813
H	-2.05993	2.838701	-0.52022	-1.98729	2.883745	-0.4171	-2.05993	2.838882	-0.52015
H	-1.23159	2.354401	-2.01334	-1.21259	2.413818	-1.94381	-1.23171	2.354456	-2.01329
H	-2.14531	-2.15651	1.666158	-1.91602	-3.02042	0.523961	-2.14496	-2.15662	1.666032
H	-3.50611	0.1967	2.085274	-3.49573	0.263979	2.095823	-3.50598	0.196492	2.085384
H	-2.38418	1.556616	1.992189	-2.30349	1.564922	2.035199	-2.38412	1.556467	1.992312
H	-1.79031	-0.02938	2.474288	-1.80627	-0.0776	2.483182	-1.79015	-0.02952	2.474312
H	0.160333	1.708672	1.755067	0.151301	1.606037	1.78711	0.160379	1.708709	1.755066
H	-4.11957	-2.66824	-0.45895	-4.18138	-2.58957	-0.4375	-4.11937	-2.66838	-0.45896
H	-4.39985	-1.62524	0.942071	-4.38966	-1.56732	0.991452	-4.39961	-1.62553	0.94217
H	-4.55009	-0.96173	-0.69122	-4.58375	-0.87821	-0.63389	-4.55005	-0.96189	-0.69105
	3b-4			3b-5			3b-6		
C	-0.8085	0.269448	-0.17131	-0.8085	0.269447	-0.17131	-0.78346	0.29854	0.29854
C	-2.11227	0.191749	0.652042	-2.11228	0.191758	0.652033	-2.12546	0.260442	0.260442
C	-2.89386	-0.93273	-0.05093	-2.89385	-0.93273	-0.05092	-2.81591	-0.98387	-0.98387
C	-2.67602	-0.60564	-1.53296	-2.67601	-0.60567	-1.53296	-2.50461	-0.85536	-0.85536
C	-1.2429	-0.04911	-1.6267	-1.2429	-0.04912	-1.6267	-1.15471	-0.11447	-0.11447
C	0.037569	1.553403	-0.02021	0.037566	1.553404	-0.02022	0.051968	1.593252	1.593252
C	1.250034	1.540667	-0.99522	1.250035	1.540665	-0.99522	1.287858	1.538058	1.538058
C	2.104518	0.320301	-0.85242	2.104517	0.320299	-0.85241	2.144525	0.331616	0.331616
C	3.231757	0.265275	-0.12998	3.231757	0.265274	-0.12998	3.253174	0.329478	0.329478
C	4.016278	-0.99261	0.00876	4.016278	-0.99261	0.008767	4.078708	-0.87077	-0.87077
O	3.686219	-2.03251	-0.54701	3.686209	-2.03252	-0.54699	5.072408	-0.7668	-0.7668
C	5.247044	-0.91229	0.878738	5.247053	-0.91229	0.878731	3.679377	-2.20469	-2.20469
C	-0.76329	2.83246	-0.24489	-0.76329	2.832457	-0.24491	-0.74976	2.854506	2.854506
H	-0.1792	-0.56098	0.174057	-0.1792	-0.56098	0.174068	-0.17009	-0.50727	-0.50727
O	-2.23003	-2.16075	0.284764	-2.23002	-2.16075	0.284792	-2.1634	-2.18756	-2.18756
C	-1.93272	0.000284	2.154392	-1.93274	0.000316	2.154387	-2.02886	0.26287	0.26287
O	0.540878	1.66324	1.315226	0.540871	1.663253	1.315221	0.517009	1.772185	1.772185

C	-4.36567	-1.0156	0.337734	-4.36567	-1.01561	0.337727	-4.30879	-1.07516	-1.07516
H	-2.69644	1.104453	0.480695	-2.69644	1.104458	0.480667	-2.73246	1.119651	1.119651
H	-3.40221	0.152624	-1.84154	-3.4022	0.152582	-1.84156	-3.29701	-0.27777	-0.27777
H	-2.83846	-1.48262	-2.16701	-2.83843	-1.48266	-2.167	-2.48828	-1.84281	-1.84281
H	-0.56192	-0.7712	-2.08269	-0.5619	-0.77121	-2.08269	-0.38044	-0.74312	-0.74312
H	-1.22678	0.840369	-2.26129	-1.22678	0.840353	-2.26129	-1.25671	0.761349	0.761349
H	0.88952	1.61539	-2.02513	0.889524	1.615386	-2.02513	0.950951	1.552713	1.552713
H	1.839611	2.435945	-0.78052	1.839612	2.435943	-0.78052	1.868664	2.446673	2.446673
H	1.774653	-0.59693	-1.3378	1.774653	-0.59694	-1.33779	1.809773	-0.60071	-0.60071
H	3.608734	1.152293	0.375161	3.608735	1.152294	0.375159	3.614162	1.247723	1.247723
H	5.937163	-0.16585	0.474843	4.971266	-0.58045	1.883853	2.679129	-2.49049	-2.49049
H	4.971256	-0.58042	1.883849	5.742142	-1.88078	0.932247	3.65901	-2.15373	-2.15373
H	5.742115	-1.88079	0.932282	5.937154	-0.16582	0.474851	4.394885	-2.96412	-2.96412
H	-0.09976	3.700525	-0.2218	-0.09977	3.700525	-0.22183	-0.09265	3.727522	3.727522
H	-1.50787	2.959044	0.543341	-1.50788	2.959043	0.543325	-1.52261	3.006704	3.006704
H	-1.27544	2.817936	-1.20972	-1.27545	2.817921	-1.20973	-1.22728	2.797227	2.797227
H	-2.63382	-2.86586	-0.23756	-2.63379	-2.86586	-0.23754	-2.32629	-2.28652	-2.28652
H	-2.89778	-0.13477	2.651875	-2.8978	-0.13474	2.651865	-3.00877	0.102898	0.102898
H	-1.44183	0.869487	2.594916	-1.44186	0.86953	2.594903	-1.63699	1.213879	1.213879
H	-1.32042	-0.88139	2.361899	-1.32043	-0.88135	2.361912	-1.34864	-0.51905	-0.51905
H	1.012433	0.844925	1.526282	1.012428	0.844941	1.526283	1.025076	0.987956	0.987956
H	-4.87819	-1.77475	-0.26298	-4.87817	-1.77477	-0.26297	-4.74538	-1.92045	-1.92045
H	-4.47274	-1.29095	1.389713	-4.47275	-1.29094	1.389711	-4.49409	-1.22528	-1.22528
H	-4.87098	-0.06	0.173007	-4.87098	-0.06002	0.172981	-4.82652	-0.16228	-0.16228
	3b-7			3b-8			3b-9		
C	-0.82632	0.276576	-0.19038	-0.73891	0.255833	-0.14967	1.240043	-0.07316	-0.35619
C	-2.15821	0.24753	0.582382	-2.02225	0.125634	0.705805	2.429381	0.577063	0.380486
C	-2.88672	-0.96312	-0.04494	-2.87402	-0.89396	-0.07199	3.598597	-0.35917	0.025243
C	-2.58573	-0.80928	-1.5392	-2.66311	-0.45031	-1.52179	2.971395	-1.7504	0.188517
C	-1.23067	-0.07735	-1.64024	-1.17522	-0.0812	-1.60378	1.492212	-1.59662	-0.21836
C	0.040792	1.547315	-0.05031	0.043476	1.582015	-0.01055	-0.17858	0.3875	0.045454
C	1.254175	1.504192	-1.02204	1.289819	1.597569	-0.94222	-1.21988	-0.45963	-0.7303
C	2.106589	0.287379	-0.84036	2.156948	0.387176	-0.78301	-2.6283	-0.00181	-0.51574
C	3.243651	0.258549	-0.13192	3.26277	0.346155	-0.02584	-3.63534	-0.79784	-0.13154
C	4.028125	-0.99506	0.044449	4.098752	-0.86163	0.176747	-5.02942	-0.34572	0.080393
O	3.674996	-2.0598	-0.44594	5.092426	-0.79365	0.891236	-5.88097	-1.15602	0.431736
C	5.288172	-0.87768	0.86689	3.709693	-2.15995	-0.49384	-5.38479	1.108641	-0.13443
C	-0.73928	2.836542	-0.28918	-0.80262	2.817253	-0.30608	-0.43394	0.320046	1.547712
H	-0.22634	-0.55808	0.197211	-0.0623	-0.53887	0.189595	1.35362	0.181196	-1.41919
O	-2.26516	-2.19391	0.358091	-2.26683	-2.17904	0.136457	3.908299	-0.11649	-1.35648
C	-2.04114	0.206741	2.102834	-1.78926	-0.23013	2.170992	2.678063	2.050478	0.076439
O	0.539887	1.663244	1.286534	0.49107	1.75267	1.337531	-0.36778	1.766612	-0.30334
C	-4.37925	-1.01986	0.262191	-4.33967	-0.93585	0.344301	4.846669	-0.15978	0.8777

H	-2.74917	1.128645	0.300585	-2.58117	1.067372	0.661572	2.279811	0.460422	1.461319
H	-3.37783	-0.21923	-2.0072	-3.28692	0.427563	-1.71974	3.050687	-2.06	1.234747
H	-2.57933	-1.78949	-2.01995	-2.95562	-1.22535	-2.23713	3.503671	-2.49784	-0.40717
H	-0.47139	-0.69384	-2.12635	-0.58875	-0.91939	-1.98732	1.27033	-2.11281	-1.1545
H	-1.33902	0.825153	-2.24829	-1.02228	0.7504	-2.29518	0.848514	-2.04063	0.54536
H	0.892136	1.547167	-2.05343	0.967324	1.679281	-1.98396	-0.9872	-0.37413	-1.80047
H	1.846142	2.404227	-0.83549	1.857367	2.499009	-0.69719	-1.12792	-1.51556	-0.46624
H	1.768384	-0.64858	-1.28237	1.832156	-0.51517	-1.29777	-2.80935	1.056865	-0.68916
H	3.631704	1.164715	0.328981	3.613396	1.236215	0.492495	-3.46909	-1.85684	0.054648
H	5.963711	-0.15007	0.407869	2.710951	-2.47239	-0.17819	-4.7894	1.751753	0.518336
H	5.046506	-0.50227	1.865481	3.691273	-2.04266	-1.58023	-5.17992	1.406551	-1.16571
H	5.785116	-1.8435	0.94583	4.429994	-2.932	-0.22708	-6.4425	1.255832	0.07988
H	-0.06345	3.694723	-0.25822	-0.17302	3.710457	-0.31467	-1.46198	0.615004	1.771625
H	-1.49312	2.975222	0.488403	-1.56363	2.9535	0.464201	0.233168	1.000772	2.079805
H	-1.23803	2.827577	-1.26085	-1.29827	2.73752	-1.27637	-0.28231	-0.69171	1.931075
H	-2.41344	-2.30642	1.306521	-2.73471	-2.81777	-0.41714	4.577785	-0.75945	-1.62406
H	-3.01762	0.053599	2.573857	-2.73722	-0.38182	2.695686	3.593194	2.402696	0.561468
H	-1.6255	1.13979	2.485206	-1.24945	0.572605	2.675836	1.848218	2.662388	0.43362
H	-1.3716	-0.59743	2.426922	-1.20605	-1.15049	2.259038	2.785117	2.211096	-0.99972
H	1.026518	0.853439	1.496165	1.026379	0.982813	1.577652	-0.04873	1.894385	-1.20715
H	-4.84261	-1.84283	-0.28855	-4.90073	-1.62572	-0.29526	5.59941	-0.91848	0.637457
H	-4.55957	-1.18571	1.329322	-4.43934	-1.27816	1.3772	5.288329	0.822574	0.69384
H	-4.87508	-0.08788	-0.02227	-4.80113	0.051555	0.257686	4.613508	-0.24461	1.94252
	3b-10			3b-11			3b-12		
C	-0.73558	0.342739	-0.09201	-0.73517	0.342765	-0.09173	-0.61361	0.205176	-0.0328
C	-2.20273	0.160541	0.350228	-2.20201	0.160421	0.351573	-1.93997	-0.0595	0.729197
C	-2.50128	-1.29353	-0.05714	-2.50164	-1.29298	-0.05745	-2.78024	-0.96209	-0.20766
C	-1.86738	-1.38208	-1.45167	-1.86875	-1.38007	-1.45248	-2.36341	-0.48526	-1.59566
C	-0.62382	-0.47288	-1.40546	-0.62398	-0.47267	-1.4054	-0.84933	-0.29598	-1.48086
C	-0.20127	1.788345	-0.18545	-0.20112	1.788469	-0.18536	-0.11548	1.661859	0.050985
C	1.233184	1.802899	-0.77667	1.233356	1.803162	-0.77658	1.29505	1.795504	-0.57558
C	2.209311	0.967857	-0.01178	2.209493	0.967995	-0.01184	2.290852	0.837642	-0.00233
C	2.926645	-0.04255	-0.51892	2.926363	-0.0427	-0.51904	3.04244	-0.00114	-0.72785
C	3.873894	-0.82883	0.315654	3.873569	-0.82919	0.315384	4.019467	-0.95727	-0.15885
O	4.055019	-0.58916	1.5031	4.055149	-0.58937	1.50273	4.672309	-1.67448	-0.90987
C	4.600963	-1.95004	-0.38704	4.599977	-1.95082	-0.38732	4.193748	-1.04204	1.341123
C	-1.08994	2.707873	-1.01855	-1.08988	2.707713	-1.01868	-1.05111	2.667889	-0.6256
H	-0.11545	-0.16858	0.657365	-0.11448	-0.16851	0.657205	0.166765	-0.40719	0.430065
O	-1.78519	-2.12981	0.864749	-1.78537	-2.13086	0.862834	-2.353	-2.33069	-0.128
C	-2.50612	0.490532	1.808022	-2.50378	0.48846	1.810149	-1.73772	-0.62185	2.133624
O	-0.15744	2.391383	1.115935	-0.15748	2.391746	1.115906	-0.02883	1.95558	1.452462
C	-3.98133	-1.65904	-0.04658	-3.98188	-1.65768	-0.04632	-4.28116	-0.87566	0.04642
H	-2.84308	0.77987	-0.29038	-2.84278	0.780893	-0.28749	-2.49277	0.880202	0.829176

H	-2.58272	-1.01601	-2.19424	-2.58407	-1.01167	-2.19393	-2.85696	0.469956	-1.80471
H	-1.63219	-2.41731	-1.71669	-1.63513	-2.41515	-1.71947	-2.65746	-1.19655	-2.37158
H	0.303332	-1.04963	-1.41811	0.302329	-1.05081	-1.41756	-0.34638	-1.25449	-1.62283
H	-0.60228	0.176668	-2.28413	-0.60091	0.17693	-2.28398	-0.46239	0.379399	-2.24748
H	1.21426	1.498626	-1.82623	1.21443	1.499018	-1.82616	1.242481	1.681387	-1.66102
H	1.572311	2.844514	-0.7413	1.572448	2.844781	-0.74107	1.644613	2.818614	-0.38347
H	2.354966	1.205371	1.04145	2.355505	1.205581	1.041331	2.377495	0.844121	1.082549
H	2.830679	-0.32019	-1.56676	2.829997	-0.32047	-1.56681	2.961051	-0.02516	-1.81253
H	5.176335	-1.54992	-1.22687	3.876145	-2.65768	-0.80325	4.524047	-0.08189	1.745281
H	5.266392	-2.46678	0.303129	5.175337	-1.55105	-1.22733	3.247225	-1.29549	1.825102
H	3.877534	-2.65717	-0.80322	5.265341	-2.46777	0.302756	4.93512	-1.80503	1.574338
H	-0.63107	3.696425	-1.10152	-0.63102	3.696234	-1.10206	-0.64378	3.680472	-0.5362
H	-2.06457	2.83029	-0.54251	-2.06444	2.830307	-0.54257	-2.03842	2.663111	-0.15974
H	-1.23909	2.314927	-2.02705	-1.23916	2.314396	-2.02701	-1.16966	2.456374	-1.69082
H	-1.88456	-3.04442	0.570206	-1.8855	-3.04503	0.567168	-2.56734	-2.65959	0.754822
H	-3.53847	0.233691	2.062612	-3.53593	0.231579	2.065486	-2.69301	-0.84581	2.621316
H	-2.36952	1.556759	1.997921	-2.36663	1.554369	2.001411	-1.20716	0.103949	2.752236
H	-1.84737	-0.06737	2.4792	-1.8445	-0.07061	2.479822	-1.13772	-1.53753	2.111813
H	0.117534	1.723709	1.757879	0.118423	1.724442	1.757842	0.182017	2.894201	1.5492
H	-4.12828	-2.67166	-0.43755	-4.12967	-2.66974	-0.43842	-4.81946	-1.50734	-0.66512
H	-4.3803	-1.63	0.970186	-4.38013	-1.62965	0.970753	-4.5306	-1.21486	1.057663
H	-4.56077	-0.97217	-0.66944	-4.56136	-0.96973	-0.66794	-4.63876	0.152074	-0.05989
	3b-13			3b-14			3b-15		
C	-0.73245	0.345609	-0.1141	1.239898	-0.08901	-0.34482	-1.43182	-1.00532	0.043437
C	-2.20048	0.196237	0.32709	2.423078	0.544419	0.409946	-2.08881	0.219007	0.744905
C	-2.52429	-1.26346	-0.0667	3.606926	-0.36405	0.005865	-3.08936	0.800626	-0.27861
C	-1.8509	-1.41057	-1.43584	2.999361	-1.77065	0.057506	-2.43882	0.462447	-1.62378
C	-0.67074	-0.41641	-1.45658	1.486377	-1.60802	-0.20183	-1.92345	-0.96237	-1.42342
C	-0.14331	1.771438	-0.15306	-0.18003	0.382278	0.038719	0.108591	-1.0909	0.179253
C	1.274017	1.760281	-0.7813	-1.22051	-0.49051	-0.70798	0.830857	0.053599	-0.55728
C	2.24153	0.862607	-0.07602	-2.62877	-0.02144	-0.517	2.322742	-0.04735	-0.48028
C	2.934062	-0.12501	-0.65906	-3.63732	-0.79934	-0.10111	3.127607	0.923363	-0.02801
C	3.892734	-1.01024	0.042056	-5.03175	-0.33794	0.087937	4.603709	0.826322	0.037644
O	4.484472	-1.87915	-0.58915	-5.88455	-1.13251	0.470539	5.253603	1.771773	0.472146
C	4.124328	-0.83531	1.526382	-5.38571	1.106276	-0.18867	5.290873	-0.43815	-0.4277
C	-1.01547	2.764808	-0.91601	-0.43707	0.362107	1.542005	0.530404	-1.13603	1.650583
H	-0.12578	-0.22483	0.605049	1.371551	0.163046	-1.40711	-1.81284	-1.91718	0.520726
O	-1.85061	-2.18377	0.807449	3.970722	-0.13983	-1.36653	-4.30994	0.058425	-0.13551
C	-2.50023	0.546619	1.781483	2.648696	2.033475	0.167648	-2.71007	-0.09348	2.104614
O	-0.04074	2.308141	1.175867	-0.36641	1.750618	-0.35286	0.562092	-2.28521	-0.47682
C	-4.01479	-1.58381	-0.09677	4.838505	-0.21222	0.892013	-3.36443	2.287642	-0.08388
H	-2.82592	0.825668	-0.31891	2.278028	0.383341	1.486036	-1.3313	0.996625	0.894712
H	-2.57192	-1.16961	-2.22093	3.17665	-2.20618	1.043939	-1.61065	1.15529	-1.80992

H	-1.53839	-2.44596	-1.58463	3.491322	-2.41226	-0.67621	-3.14152	0.561151	-2.4578
H	0.289156	-0.92199	-1.57705	1.161934	-2.14921	-1.09277	-2.74741	-1.66767	-1.55187
H	-0.77585	0.270832	-2.30069	0.918739	-2.01495	0.640022	-1.14703	-1.24742	-2.13608
H	1.217602	1.497282	-1.84053	-0.9835	-0.45098	-1.78002	0.547471	0.012119	-1.61447
H	1.651756	2.787182	-0.71672	-1.13152	-1.53442	-0.39894	0.503479	1.018741	-0.16444
H	2.38483	1.05699	0.985914	-2.80867	1.028927	-0.73612	2.74584	-0.98718	-0.82755
H	2.819256	-0.33227	-1.72094	-3.47223	-1.84945	0.13079	2.718094	1.865772	0.32982
H	4.499719	0.16785	1.743076	-4.79099	1.776028	0.437372	4.945537	-1.29912	0.150094
H	3.190576	-0.96653	2.079014	-5.17887	1.360004	-1.23133	5.065565	-0.63538	-1.47863
H	4.85038	-1.5726	1.866036	-6.4437	1.263057	0.017247	6.36733	-0.32843	-0.30384
H	-0.52691	3.74203	-0.94578	-1.46595	0.662294	1.754806	1.600661	-1.34186	1.729594
H	-1.9799	2.887626	-0.41958	0.2284	1.059787	2.053887	-0.01096	-1.92558	2.181767
H	-1.18882	2.439033	-1.94418	-0.28417	-0.63716	1.95626	0.331043	-0.18829	2.157473
H	-2.25932	-2.11921	1.680844	4.351055	0.746336	-1.43224	-4.90166	0.332783	-0.84846
H	-3.52893	0.287094	2.050666	3.57952	2.375815	0.631325	-3.19565	0.789664	2.530028
H	-2.37097	1.615351	1.959849	1.830386	2.623485	0.582385	-1.94786	-0.4265	2.813425
H	-1.82613	0.017901	2.464977	2.695892	2.258205	-0.90352	-3.46204	-0.88014	2.015219
H	0.166588	1.588638	1.785725	-0.07506	1.842386	-1.27024	0.144751	-3.03991	-0.0403
H	-4.17014	-2.59973	-0.46952	5.59735	-0.9451	0.605047	-4.07214	2.648804	-0.83743
H	-4.4576	-1.5204	0.902757	5.279492	0.785212	0.794321	-3.79715	2.474171	0.902651
H	-4.55247	-0.88882	-0.74718	4.586133	-0.36664	1.944464	-2.44578	2.873091	-0.17819
	3b-16			3b-17			3b-18		
C	-0.61493	0.211877	-0.02638	-1.28895	0.351522	-0.72187	-0.61161	0.191693	-0.04284
C	-1.9496	-0.03689	0.728143	-1.99493	-0.76662	0.096054	-1.92144	-0.08266	0.749955
C	-2.76594	-0.97484	-0.18366	-3.43989	-0.27595	0.318634	-2.80508	-0.93561	-0.18609
C	-2.34499	-0.52734	-1.58633	-3.26087	1.238334	0.435409	-2.41015	-0.42792	-1.57563
C	-0.83344	-0.32551	-1.46445	-2.2575	1.566503	-0.66986	-0.89007	-0.28759	-1.49062
C	-0.11792	1.670745	0.024086	0.140065	0.698634	-0.22997	-0.10382	1.650711	0.050362
C	1.294769	1.790852	-0.60116	1.013153	-0.56613	-0.12797	1.296263	1.784439	-0.58173
C	2.28623	0.838423	-0.01173	2.42382	-0.28122	0.284403	2.304417	0.838792	-0.00769
C	3.0377	-0.0116	-0.72425	3.509459	-0.68554	-0.38844	3.041234	-0.01365	-0.7325
C	4.009351	-0.96438	-0.14087	4.904162	-0.41139	0.025987	4.038202	-0.95143	-0.16799
O	4.659804	-1.69475	-0.88142	5.832366	-0.82145	-0.66352	4.668072	-1.68936	-0.91888
C	4.180278	-1.03026	1.36029	5.166784	0.375103	1.291097	4.261542	-0.99195	1.327218
C	-1.0516	2.660207	-0.67929	0.80561	1.704487	-1.17621	-1.04066	2.668035	-0.60845
H	0.159728	-0.38824	0.461161	-1.18754	0.019961	-1.76097	0.177107	-0.42906	0.392607
O	-2.29203	-2.30875	0.05873	-4.17499	-0.58376	-0.87621	-2.37971	-2.29722	-0.03122
C	-1.76724	-0.54898	2.153909	-1.91817	-2.15844	-0.52881	-1.69411	-0.70534	2.12633
O	-0.03518	1.998518	1.417799	0.101708	1.22604	1.104278	0.090846	2.010432	1.427021
C	-4.27018	-0.90941	0.056089	-4.12561	-0.9068	1.525404	-4.29672	-0.82289	0.109169
H	-2.51251	0.900697	0.779813	-1.53971	-0.79538	1.092978	-2.4711	0.85711	0.887832
H	-2.84275	0.419104	-1.82595	-2.84349	1.474627	1.420027	-2.87981	0.54643	-1.751
H	-2.62702	-1.2523	-2.35721	-4.20875	1.777521	0.337254	-2.74198	-1.10142	-2.37239

H	-0.32039	-1.28281	-1.57813	-2.77703	1.66111	-1.62526	-0.41925	-1.26017	-1.6488
H	-0.44577	0.33408	-2.24417	-1.76149	2.525295	-0.49551	-0.49586	0.383019	-2.25736
H	1.245495	1.66167	-1.68505	0.574643	-1.2248	0.628895	1.232068	1.656776	-1.66442
H	1.646831	2.815539	-0.42265	0.996032	-1.09704	-1.08329	1.635307	2.809627	-0.39334
H	2.369196	0.858739	1.073258	2.534813	0.291634	1.202695	2.417353	0.872096	1.073879
H	2.959418	-0.04883	-1.80883	3.413045	-1.25338	-1.3115	2.931989	-0.06267	-1.81399
H	4.507581	-0.06479	1.753986	4.704374	1.36364	1.23345	4.595698	-0.01811	1.693507
H	3.232745	-1.27963	1.844445	4.742236	-0.13692	2.158151	3.333156	-1.23894	1.848248
H	4.922113	-1.78928	1.604716	6.241414	0.486765	1.428686	5.016316	-1.74253	1.557836
H	-0.64221	3.674116	-0.61739	1.785113	1.999493	-0.79221	-0.62984	3.673816	-0.49053
H	-2.03854	2.670224	-0.21283	0.203939	2.608988	-1.295	-2.03358	2.65703	-0.14953
H	-1.17125	2.420114	-1.73839	0.946409	1.264611	-2.16728	-1.16531	2.472632	-1.67609
H	-2.70866	-2.88844	-0.59234	-5.05261	-0.18941	-0.78767	-2.83993	-2.82404	-0.69786
H	-2.73218	-0.7263	2.639133	-2.47029	-2.89031	0.068194	-2.6411	-0.88724	2.64303
H	-1.21718	0.18701	2.743522	-0.88594	-2.50803	-0.60132	-1.08732	-0.04857	2.75546
H	-1.21043	-1.48888	2.159619	-2.34631	-2.15418	-1.53342	-1.17076	-1.65898	2.034123
H	0.169749	2.94044	1.4922	-0.29002	2.108426	1.071255	-0.7747	2.059542	1.853045
H	-4.79932	-1.57891	-0.6305	-5.13514	-0.50024	1.648576	-4.87464	-1.43236	-0.59349
H	-4.5108	-1.21454	1.077838	-4.21159	-1.98925	1.400194	-4.51664	-1.17407	1.12075
H	-4.64952	0.103648	-0.10363	-3.56762	-0.70476	2.443538	-4.63886	0.211499	0.016027
	3b-19			3b-20			3b-21		
C	-1.29382	0.374885	-0.7029	-1.28747	0.35097	-0.72256	-0.81126	0.276421	-0.17409
C	-2.00011	-0.76832	0.081888	-1.99485	-0.75872	0.104094	-2.12984	0.212419	0.623767
C	-3.44337	-0.27829	0.315905	-3.44699	-0.26376	0.318934	-2.88923	-0.95541	-0.04915
C	-3.25664	1.228687	0.488787	-3.27187	1.246966	0.412189	-2.62486	-0.70888	-1.54209
C	-2.24299	1.59923	-0.59311	-2.25749	1.565503	-0.68648	-1.22541	-0.062	-1.63034
C	0.140679	0.697812	-0.22378	0.140554	0.701983	-0.23092	0.040025	1.560034	-0.03024
C	1.01146	-0.58089	-0.18077	1.013282	-0.56241	-0.12046	1.253169	1.535989	-1.00399
C	2.415972	-0.33278	0.270653	2.425123	-0.27617	0.287009	2.104667	0.315602	-0.84463
C	3.512182	-0.63108	-0.43965	3.508771	-0.69086	-0.38264	3.231392	0.263578	-0.12147
C	4.901253	-0.39324	0.015825	4.904945	-0.41719	0.027396	4.006109	-0.99959	0.031584
O	5.837485	-0.69205	-0.71795	5.830719	-0.8393	-0.65803	3.634791	-2.05498	-0.466
C	5.148257	0.217592	1.377384	5.171719	0.383062	1.282879	5.276787	-0.90455	0.839483
C	0.792635	1.728489	-1.14973	0.80704	1.70242	-1.18221	-0.75981	2.837608	-0.26534
H	-1.20854	0.073697	-1.75266	-1.18253	0.010033	-1.75843	-0.17339	-0.53964	0.199766
O	-4.17404	-0.53976	-0.89355	-4.24861	-0.4868	-0.85153	-2.36128	-2.22712	0.359484
C	-1.92998	-2.13874	-0.59012	-1.91649	-2.15447	-0.51209	-1.98474	0.085858	2.135421
O	0.034361	1.219187	1.11042	0.099841	1.236747	1.099962	0.538687	1.679084	1.304812
C	-4.13901	-0.95063	1.494545	-4.12883	-0.87824	1.536304	-4.36903	-1.00915	0.294712
H	-1.54775	-0.83521	1.078247	-1.53954	-0.78511	1.101063	-2.71418	1.113356	0.397547
H	-2.83693	1.427151	1.479963	-2.86445	1.494087	1.397734	-3.38207	-0.02308	-1.93158
H	-4.20225	1.775649	0.408073	-4.22353	1.770723	0.29336	-2.70863	-1.64012	-2.10736
H	-2.75042	1.761477	-1.54667	-2.76912	1.655389	-1.6464	-0.4973	-0.72509	-2.10464

H	-1.71833	2.520992	-0.33845	-1.76219	2.525073	-0.51372	-1.26911	0.837638	-2.24862
H	0.556647	-1.27438	0.533288	0.575748	-1.21408	0.643065	0.892997	1.599546	-2.03473
H	1.008792	-1.06052	-1.16384	0.993874	-1.10111	-1.07149	1.84333	2.432875	-0.79852
H	2.522149	0.113065	1.258487	2.53857	0.306185	1.199015	1.77285	-0.60705	-1.31852
H	3.428771	-1.07889	-1.42761	3.409627	-1.26833	-1.29943	3.619103	1.155937	0.365678
H	4.678994	1.202062	1.449298	4.712213	1.372259	1.214535	5.95247	-0.17553	0.383042
H	4.722789	-0.40941	2.164756	4.746781	-0.11772	2.156301	5.050117	-0.54189	1.846255
H	6.220951	0.318195	1.536333	6.246861	0.492949	1.417813	5.766589	-1.87537	0.898434
H	1.788649	2.005014	-0.78858	1.78604	1.999566	-0.79864	-0.09411	3.703854	-0.2454
H	0.193545	2.637878	-1.2231	0.205498	2.606163	-1.30661	-1.50552	2.969359	0.520854
H	0.914262	1.315795	-2.15446	0.949043	1.257011	-2.17065	-1.26968	2.819233	-1.23106
H	-5.03622	-0.11194	-0.80906	-4.38681	-1.43851	-0.944	-1.45916	-2.31655	0.025967
H	-2.48075	-2.88949	-0.01563	-2.49712	-2.8809	0.066625	-2.96167	-0.03168	2.613471
H	-0.8994	-2.4887	-0.68196	-0.89006	-2.52431	-0.5482	-1.50296	0.970865	2.553446
H	-2.36416	-2.09804	-1.59134	-2.29395	-2.15165	-1.5393	-1.37887	-0.78714	2.394767
H	0.690609	1.916458	1.225408	-0.29921	2.115743	1.062568	1.039898	0.877946	1.513603
H	-5.14807	-0.54529	1.625093	-5.13121	-0.45861	1.655207	-4.86548	-1.77988	-0.30084
H	-4.22822	-2.02774	1.330361	-4.22713	-1.96411	1.430597	-4.51281	-1.25262	1.350245
H	-3.58674	-0.78326	2.423135	-3.55767	-0.68058	2.447071	-4.8479	-0.04849	0.090014

Table S 12. Energy analysis for 2*S*, 3*R*, 6*R*, 7*S*-3 in the Chloroform at MPW1PW91/6-31G+d, p level

Conf.	Steric Energy	Relative Energy	Distribution
	(kJ/mol)	(kJ/mol)	(%)
3b-1	-773.144879	0.000231	7.30%
3b-2	-773.143628	0.001482	1.94%
3b-3	-773.144875	0.000235	7.27%
3b-4	-773.144714	0.000396	6.13%
3b-5	-773.144715	0.000395	6.13%
3b-6	-773.14511	0.000000	9.32%
3b-7	-773.145017	0.000093	8.44%
3b-8	-773.145014	0.000096	8.42%
3b-9	-773.144041	0.001069	3.00%
3b-10	-773.143847	0.001263	2.45%
3b-11	-773.143931	0.001179	2.67%
3b-12	-773.144537	0.000573	5.08%
3b-13	-773.144411	0.000699	4.45%
3b-14	-773.144925	0.000185	7.66%
3b-15	-773.144027	0.001083	2.96%
3b-16	-773.143487	0.001623	1.67%
3b-17	-773.143671	0.001439	2.03%
3b-18	-773.144347	0.000763	4.15%
3b-19	-773.143335	0.001775	1.42%
3b-20	-773.143534	0.001576	1.76%
3b-21	-773.144654	0.000456	5.75%

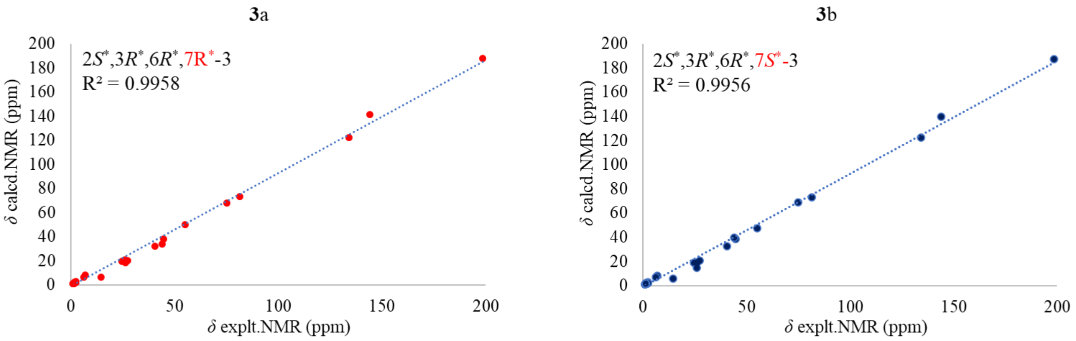

Figure S 79. Correlation between the calculated ^{13}C NMR data for 2*S*, 3*R*, 6*R*, 7*R*-3 (a) and 2*S*, 3*R*, 6*R*, 7*S*-3 (b) and experimental ^{13}C NMR data of 3

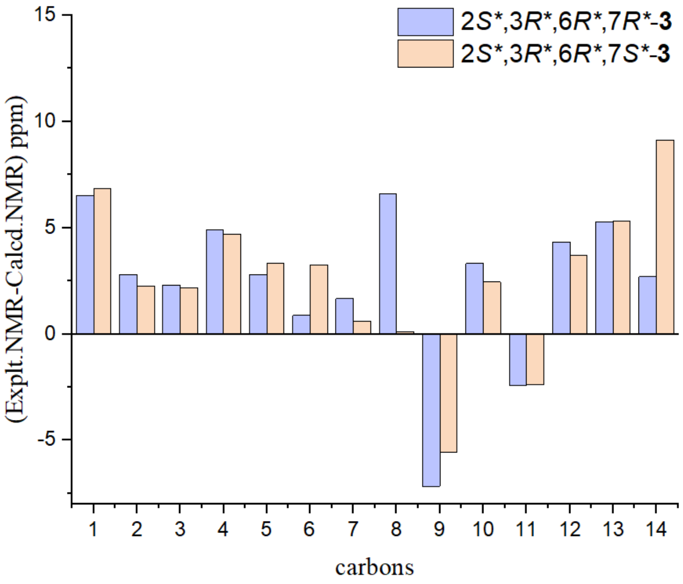

Figure S 80. Differences between experimental NMR chemical shifts of 3 and theoretical NMR chemical shifts for 2*S*, 3*R*, 6*R*, 7*R*-3 (yellow bar) and 2*S*, 3*R*, 6*R*, 7*S*-3 (purple bar)

Table S 13. Experimental chemical shifts of 3, the calculated shielding tensors for 2*S*, 3*R*, 6*R*, 7*R*-3 (isomer 1) and 2*S*, 3*R*, 6*R*, 7*S*-3 (isomer 2), as well as their DP4+ probability

	A	B	C	D	E	F	G	H
1	Functional		Solvent?		Basis Set		Type of Data	
2	mPW1PW91		PCM		6-31+G(d,p)		Shielding Tensors	
3								
12			DP4+	96.49%	3.51%	-	-	-
14	Nuclei	sp2?	xperimenta	Isomer 1	Isomer 2	Isomer 3	Isomer 4	Isomer 5
15	C		54.9	138.9	141.2			
16	C		44.6	150.5	150			
17	C		81.4	115.4	115.4			
18	C		40.4	156.4	156.3			
19	C		24.6	169.3	169.8			
20	C		75	120.8	119.9			
21	C		43.8	154.8	148.8			
22	C	x	144.1	47.5	49.2			
23	C	x	134.2	66.7	66.1			
24	C	x	198.5	0.8	1.1			
25	C		27.2	168.3	167.8			
26	C		25.9	168	174.1			
27	C		14.6	182.2	182.6			
28	C		26.2	170.1	170.2			
29	H		1.86	29.8	29.8			
30	H		1.62	30	29.9			
31	H		1.58	30	30			
32	H		1.72	30.1	30.1			
33	H		1.58	29.8	29.7			
34	H		1.91	30.3	30.4			
35	H		2.45	29.1	29.1			
36	H		2.36	29.4	29.5			
37	H	x	6.88	23.9	24			
38	H	x	6.13	25.3	25.2			
39	H		2.27	29.2	29.2			
40	H		2.27	29.8	29.3			
41	H		2.27	29.2	29.7			
42	H		1.19	30.6	30.7			
43	H		1.19	30.7	30.4			
44	H		1.19	30.5	30.6			
45	H		1.06	30.7	30.7			
46	H		1.06	30.1	30.1			
47	H		1.06	30.9	31			
48	H		1.27	30.4	30.4			
49	H		1.27	30.4	30.4			
50	H		1.27	30.8	30.9			

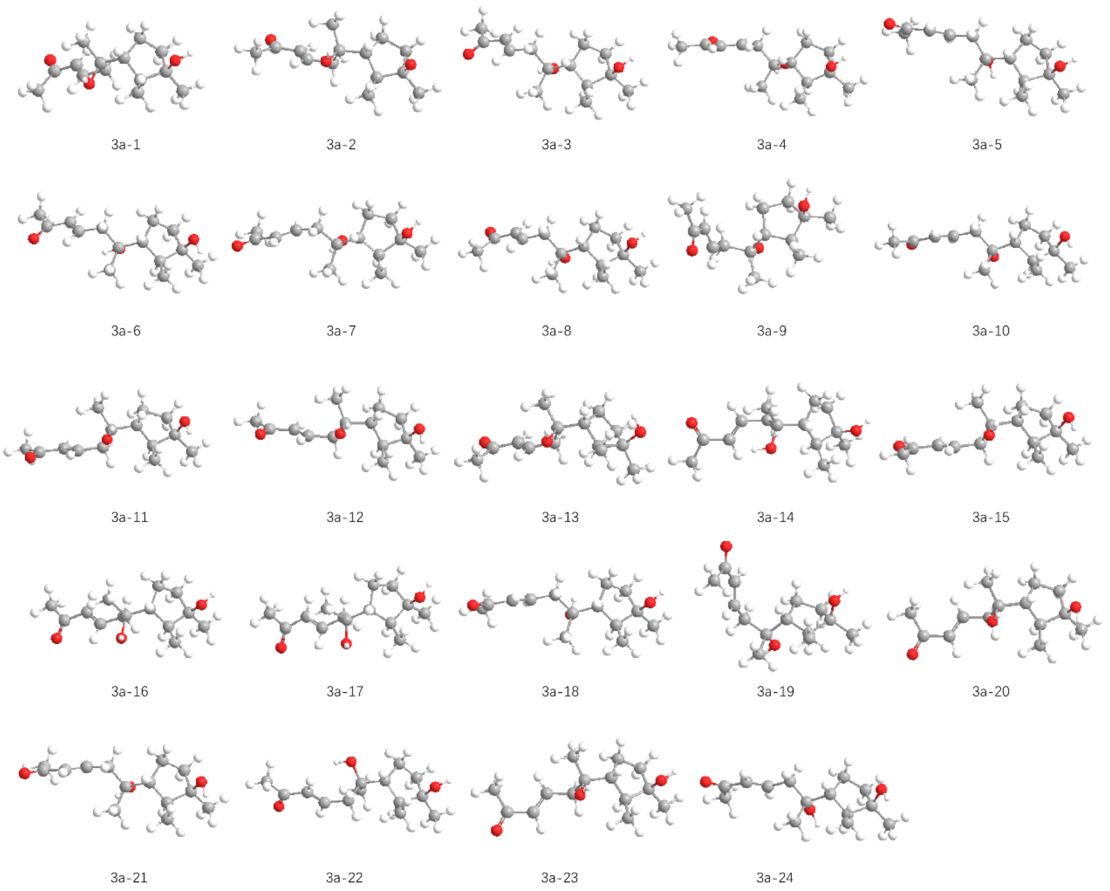

Figure S 81. Most stable conformers of 2*S*, 3*R*, 6*R*, 7*R*-3 at the CAM-B3LYP/DGDZVP level

Table S 14. Optimized Z-Matrixes of 2*S*, 3*R*, 6*R*, 7*R*-3 conformations in the methanol at CAM-B3LYP/DGDZVP level

	3a-1			3a-2			3a-3		
C	-1.39494	0.608427	0.74754	-1.33699	0.177046	0.695123	1.236444	0.249049	-0.6288
C	-2.22109	0.459049	-0.56061	-2.11094	0.312271	-0.62864	2.406377	0.606707	0.331429
C	-3.14637	-0.75533	-0.33745	-3.42169	-0.45263	-0.33385	3.409857	-0.55743	0.211865
C	-2.31731	-1.64737	0.590999	-2.93606	-1.68419	0.438351	2.495673	-1.76185	-0.0105
C	-1.70208	-0.66105	1.584238	-1.63163	-1.27035	1.152385	1.440544	-1.24868	-0.99054
C	0.114028	0.87412	0.518161	0.157063	0.566899	0.700639	-0.17019	0.545725	-0.06212
C	0.832287	-0.33003	-0.12034	0.922924	-0.06715	-0.47364	-1.21923	0.326155	-1.19096
C	2.281913	-0.07487	-0.39279	2.396895	0.186244	-0.42314	-2.62969	0.526917	-0.73061
C	3.283098	-0.84805	0.049896	3.33314	-0.77202	-0.45735	-3.43924	-0.46244	-0.32744
C	4.71498	-0.6046	-0.22942	4.790141	-0.52154	-0.41356	-4.82165	-0.20309	0.158291
O	5.559038	-1.3758	0.221414	5.569092	-1.47115	-0.45686	-5.28762	0.928981	0.230679
C	5.123825	0.588684	-1.06187	5.303811	0.896076	-0.31094	-5.62102	-1.41773	0.557347
C	0.789296	1.267875	1.83605	0.791126	0.191329	2.045058	-0.29133	1.969315	0.490786
H	-1.7659	1.486695	1.289975	-1.82088	0.852377	1.414157	1.334919	0.85762	-1.53401
O	-4.30664	-0.27758	0.3645	-4.24711	0.289345	0.58151	4.190988	-0.31959	-0.97249
C	-2.95083	1.730763	-0.98544	-2.30046	1.735604	-1.14359	3.033395	1.97714	0.082146
O	0.274527	1.943635	-0.42682	0.297848	1.983519	0.50382	-0.38657	-0.39372	0.994924
C	-3.57342	-1.44377	-1.62896	-4.22996	-0.80631	-1.57756	4.333555	-0.70148	1.416398

H	-1.54652	0.181118	-1.37671	-1.59661	-0.27368	-1.40145	2.030699	0.568585	1.360209
H	-1.53729	-2.14847	0.00738	-3.71313	-2.02226	1.127334	2.027557	-2.03627	0.940088
H	-2.92227	-2.4257	1.067091	-2.74743	-2.4984	-0.26574	3.045012	-2.63525	-0.37738
H	-2.43132	-0.42011	2.360542	-1.71834	-1.32994	2.239072	1.8054	-1.33606	-2.01645
H	-0.82708	-1.07367	2.091484	-0.82019	-1.94504	0.865501	0.521431	-1.83137	-0.91846
H	0.348854	-0.55659	-1.07709	0.537432	0.373667	-1.39936	-1.10459	-0.68652	-1.58166
H	0.724263	-1.20879	0.518592	0.727226	-1.14142	-0.5117	-0.99831	1.0271	-2.00153
H	2.499336	0.80269	-0.99723	2.691923	1.231481	-0.35742	-3.01636	1.543515	-0.68917
H	3.079623	-1.72447	0.661734	3.051243	-1.82118	-0.51807	-3.10528	-1.49745	-0.35757
H	4.776046	1.51678	-0.602	4.921151	1.379076	0.591398	-5.09526	-1.96461	1.345058
H	6.209242	0.61346	-1.1493	6.39253	0.884767	-0.28029	-6.61206	-1.12966	0.905262
H	4.681994	0.530326	-2.05951	4.972424	1.488555	-1.16709	-5.71016	-2.09657	-0.29554
H	1.831217	1.547777	1.661733	1.793368	0.620513	2.120726	-1.2989	2.150417	0.877072
H	0.274598	2.123196	2.285133	0.187929	0.580335	2.871748	0.403722	2.124058	1.317581
H	0.774321	0.449034	2.559376	0.874399	-0.89135	2.166299	-0.0889	2.713741	-0.28395
H	-4.83268	-1.04946	0.613245	-4.53628	1.09667	0.134899	4.707936	-1.11862	-1.14175
H	-3.56673	1.557851	-1.87331	-2.96847	1.760575	-2.01046	3.872257	2.158102	0.761162
H	-2.23133	2.516459	-1.22839	-1.34513	2.169674	-1.44174	2.308894	2.780002	0.23387
H	-3.60329	2.094526	-0.18821	-2.71689	2.386098	-0.36715	3.407426	2.048983	-0.94179
H	-0.09729	2.744499	-0.03297	-0.17272	2.431065	1.22018	-1.26308	-0.22952	1.370698
H	-4.2156	-2.30359	-1.41171	-5.08783	-1.42574	-1.30178	5.003677	-1.55723	1.283767
H	-4.1337	-0.75466	-2.2668	-4.60879	0.091834	-2.07588	4.949267	0.193132	1.543029
H	-2.7043	-1.80415	-2.18586	-3.61976	-1.35906	-2.29673	3.75933	-0.86139	2.332882
	3a-4			3a-5			3a-6		
C	1.223216	0.081929	-0.55394	1.208888	0.102094	-0.54118	1.234735	0.247973	-0.62703
C	2.383809	0.70914	0.267515	2.377655	0.688832	0.301466	2.404403	0.59594	0.335798
C	3.586275	-0.23435	0.068034	3.576398	-0.24871	0.052037	3.411387	-0.573	0.204149
C	2.918767	-1.60668	-0.01599	2.90239	-1.61281	-0.09469	2.500403	-1.77013	-0.03583
C	1.685114	-1.36502	-0.88622	1.666277	-1.32538	-0.94742	1.436735	-1.24687	-1.00198
C	-0.15168	0.121698	0.149021	-0.17023	0.106255	0.167886	-0.17144	0.543867	-0.05929
C	-1.24196	-0.33478	-0.85803	-1.25727	-0.31409	-0.84292	-1.22065	0.331172	-1.18923
C	-2.58914	-0.53318	-0.24192	-2.62476	-0.41654	-0.24392	-2.63084	0.531665	-0.72791
C	-3.69845	0.138603	-0.57689	-3.71314	0.202159	-0.72246	-3.44173	-0.45838	-0.32919
C	-5.00595	-0.1242	0.079748	-5.06637	0.084879	-0.13725	-4.82368	-0.19903	0.157987
O	-5.14132	-0.9719	0.956439	-6.0043	0.687357	-0.65485	-5.28796	0.933416	0.234961
C	-6.16537	0.716567	-0.39396	-5.27978	-0.76949	1.091179	-5.62465	-1.41404	0.552542
C	-0.5029	1.514033	0.676572	-0.52239	1.474225	0.757384	-0.28958	1.965174	0.500184
H	1.109653	0.648398	-1.4846	1.097674	0.718741	-1.43971	1.334436	0.863166	-1.52772
O	4.172499	0.09644	-1.20352	4.155952	0.142454	-1.20391	4.202647	-0.44142	-0.98962
C	2.690537	2.1664	-0.07256	2.682954	2.159732	0.025288	3.031509	1.968099	0.094354
O	-0.07778	-0.80306	1.243535	-0.20948	-0.88465	1.205673	-0.38883	-0.40011	0.993281
C	4.641438	-0.13094	1.163829	4.636592	-0.2008	1.146675	4.327589	-0.73089	1.412589
H	2.126165	0.637973	1.330582	2.144967	0.582535	1.369475	2.030057	0.555241	1.365

H	2.616696	-1.91938	0.988475	2.604516	-1.97364	0.895645	2.038608	-2.05692	0.91364
H	3.593025	-2.37125	-0.41601	3.572128	-2.36054	-0.53175	3.056862	-2.6294	-0.41964
H	1.949442	-1.43656	-1.94369	1.925883	-1.34682	-2.00801	1.791478	-1.32749	-2.03193
H	0.914422	-2.11206	-0.69067	0.893643	-2.07762	-0.78219	0.517916	-1.82969	-0.92576
H	-0.93245	-1.28984	-1.29421	-0.99653	-1.29782	-1.2472	-1.10735	-0.67981	-1.58464
H	-1.30011	0.394735	-1.67082	-1.25929	0.39143	-1.67763	-0.99878	1.035642	-1.99648
H	-2.67293	-1.29253	0.53454	-2.70459	-1.0516	0.635451	-3.01623	1.548546	-0.68187
H	-3.67123	0.905722	-1.34779	-3.64744	0.845731	-1.5973	-3.1093	-1.49374	-0.36396
H	-7.07522	0.45674	0.145415	-4.65787	-0.41727	1.917635	-5.09956	-1.9645	1.3382
H	-5.93784	1.776354	-0.24859	-6.32794	-0.7263	1.384145	-6.61527	-1.1259	0.901598
H	-6.31558	0.567943	-1.46708	-5.00294	-1.80727	0.89064	-5.71474	-2.08963	-0.30281
H	-1.50615	1.516882	1.11529	-1.51447	1.442662	1.214951	-1.29792	2.14858	0.883191
H	0.201289	1.829927	1.448699	0.190454	1.772211	1.530841	0.40222	2.112472	1.331094
H	-0.49408	2.251688	-0.12979	-0.52613	2.245566	-0.01741	-0.08069	2.712663	-0.26993
H	4.821531	-0.5895	-1.40953	4.802771	-0.53333	-1.44715	4.797346	0.311388	-0.87322
H	3.538202	2.538551	0.5109	3.535114	2.504703	0.61838	3.895067	2.135195	0.746911
H	1.837032	2.813314	0.140761	1.830606	2.79575	0.272664	2.321561	2.774218	0.287814
H	2.938461	2.272087	-1.13122	2.922525	2.309928	-1.02968	3.360228	2.071927	-0.94449
H	-0.74718	-0.56232	1.896866	0.349019	-0.58458	1.934721	-1.26594	-0.23806	1.368602
H	5.452733	-0.84366	0.982438	5.44618	-0.90373	0.924996	4.981103	-1.5969	1.276823
H	5.074164	0.872658	1.193447	5.069969	0.799933	1.224134	4.960238	0.152539	1.550351
H	4.210456	-0.35308	2.143729	4.211104	-0.47212	2.116576	3.746583	-0.87393	2.327303
	3a-7			3a-8			3a-9		
C	1.216528	0.12721	-0.5645	1.222933	0.211517	-0.63782	-0.623	0.206643	-0.03506
C	2.373605	0.677488	0.314774	2.382213	0.648127	0.302528	-1.97981	0.060682	0.710491
C	3.554083	-0.28832	0.092756	3.424748	-0.48487	0.225933	-2.7795	-0.98633	-0.09039
C	2.851507	-1.63505	-0.07693	2.551184	-1.7276	0.058177	-2.32994	-0.72125	-1.52734
C	1.644501	-1.31468	-0.95913	1.478718	-1.29153	-0.94013	-0.82277	-0.4947	-1.40643
C	-0.17338	0.17137	0.107164	-0.19117	0.480547	-0.07614	-0.12331	1.660394	-0.16224
C	-1.25077	-0.1987	-0.94873	-1.23612	0.174859	-1.1882	1.271743	1.710661	-0.84272
C	-2.60888	-0.42799	-0.36601	-2.65183	0.356212	-0.73283	2.342826	0.965048	-0.11202
C	-3.69262	0.312824	-0.63686	-3.42475	-0.64275	-0.2812	2.952527	-0.13403	-0.57481
C	-5.03514	0.07968	-0.05908	-4.81171	-0.47663	0.211792	3.996081	-0.84554	0.208285
O	-5.96257	0.823947	-0.368	-5.43049	-1.45943	0.610664	4.384247	-0.44426	1.300891
C	-5.24897	-1.06538	0.9036	-5.44352	0.895513	0.220476	4.551871	-2.10019	-0.41805
C	-0.4989	1.543665	0.699258	-0.3619	1.920871	0.417093	-0.06916	2.375999	1.190873
H	1.141733	0.742844	-1.4675	1.296736	0.786376	-1.5671	0.142095	-0.33123	0.533722
O	4.178737	0.090731	-1.14656	4.191941	-0.26999	-0.97191	-2.31524	-2.28197	0.328847
C	2.728434	2.141099	0.059225	2.960786	2.028356	-0.00316	-1.85515	-0.27533	2.19551
O	-0.15446	-0.81147	1.152871	-0.36819	-0.42067	1.020545	-1.05157	2.337338	-1.0191
C	4.585964	-0.27176	1.215166	4.358029	-0.54734	1.430099	-4.28836	-0.8932	0.108165
H	2.088685	0.558098	1.366563	2.01079	0.638309	1.333499	-2.5323	1.001126	0.604723
H	2.518548	-1.99024	0.903358	2.092767	-1.97623	1.020551	-2.81928	0.186787	-1.89355

H	3.513726	-2.39677	-0.50176	3.128535	-2.59742	-0.27242	-2.5966	-1.54104	-2.20275
H	1.929034	-1.34267	-2.01336	1.846665	-1.4065	-1.96217	-0.29531	-1.45176	-1.41138
H	0.848562	-2.04715	-0.81622	0.58057	-1.9027	-0.8441	-0.44307	0.089737	-2.24517
H	-0.94425	-1.12069	-1.45241	-1.09379	-0.85214	-1.52894	1.565681	2.765755	-0.90898
H	-1.29048	0.591441	-1.70372	-1.03921	0.840657	-2.03374	1.183662	1.336676	-1.86425
H	-2.69309	-1.26822	0.320867	-3.04816	1.36912	-0.7487	2.640667	1.33563	0.867672
H	-3.63065	1.156041	-1.32166	-3.05186	-1.66479	-0.26206	2.684555	-0.54787	-1.54468
H	-4.60597	-0.95586	1.780306	-5.47746	1.309464	-0.79015	4.991874	-1.86404	-1.39106
H	-6.29074	-1.08095	1.220992	-6.456	0.825127	0.615566	5.303596	-2.55289	0.227114
H	-5.00077	-2.01831	0.430164	-4.86087	1.582877	0.838637	3.741924	-2.81232	-0.59979
H	-1.51557	1.555209	1.1058	-1.37288	2.080241	0.804576	0.378286	3.369412	1.080114
H	0.189258	1.7944	1.508946	0.332485	2.136513	1.230672	-1.07458	2.505015	1.595995
H	-0.44086	2.323137	-0.06444	-0.19321	2.638918	-0.38999	0.526095	1.820047	1.919434
H	4.813265	-0.60203	-1.37387	4.730721	-1.05968	-1.11474	-2.70303	-2.93426	-0.26982
H	3.574355	2.456362	0.677641	3.792352	2.266039	0.667269	-2.83922	-0.4152	2.653296
H	1.889773	2.800872	0.291027	2.208503	2.810875	0.116548	-1.35005	0.52327	2.742857
H	3.000839	2.295404	-0.98745	3.332708	2.07198	-1.02941	-1.28389	-1.19556	2.337411
H	-0.81815	-0.57252	1.812766	-1.24453	-0.26712	1.400838	-0.80804	3.272461	-1.04902
H	5.381309	-0.99702	1.01409	5.056023	-1.38509	1.329758	-4.80116	-1.64504	-0.50076
H	5.04542	0.716168	1.306524	4.943945	0.371847	1.515784	-4.55397	-1.06791	1.154316
H	4.126663	-0.53191	2.172593	3.793324	-0.68772	2.35568	-4.66178	0.091267	-0.18616
	3a-10			3a-11			3a-12		
C	1.222231	0.084614	-0.55673	-1.3539	0.211395	0.687561	-1.35391	0.211406	0.68756
C	2.381512	0.698292	0.275171	-2.1321	0.271115	-0.63953	-2.13209	0.271109	-0.63954
C	3.587147	-0.25054	0.068095	-3.41426	-0.53025	-0.31701	-3.41426	-0.53026	-0.31701
C	2.918983	-1.61479	-0.04621	-2.88448	-1.71493	0.497687	-2.88448	-1.71493	0.497704
C	1.681678	-1.35866	-0.90797	-1.60078	-1.22608	1.201482	-1.60078	-1.22607	1.201499
C	-0.15428	0.119831	0.142813	0.126816	0.649858	0.676162	0.126815	0.649867	0.676159
C	-1.24331	-0.32177	-0.87249	0.914753	-0.00935	-0.46974	0.914751	-0.00935	-0.46974
C	-2.58998	-0.53344	-0.25956	2.378663	0.294847	-0.43533	2.378662	0.294844	-0.43533
C	-3.69736	0.152572	-0.57122	3.352308	-0.62463	-0.41699	3.352306	-0.62463	-0.41699
C	-5.00395	-0.12365	0.081894	4.789316	-0.2488	-0.38881	4.789315	-0.24881	-0.38882
O	-5.14115	-0.99914	0.930493	5.163596	0.91997	-0.39303	5.163596	0.919965	-0.39303
C	-6.1598	0.739984	-0.35821	5.772074	-1.39307	-0.35146	5.772071	-1.39308	-0.35145
C	-0.50254	1.507465	0.684765	0.770117	0.353548	2.035887	0.770115	0.353567	2.035886
H	1.111811	0.663005	-1.48049	-1.85934	0.89812	1.380545	-1.85934	0.898139	1.380535
O	4.215433	-0.01641	-1.20448	-4.26703	0.212783	0.571891	-4.26703	0.212785	0.571884
C	2.692137	2.157872	-0.05262	-2.37362	1.667677	-1.2039	-2.37362	1.667664	-1.20391
O	-0.08481	-0.81724	1.22686	0.220939	2.060303	0.418978	0.22094	2.06031	0.418964
C	4.629204	-0.1687	1.17796	-4.20842	-0.95664	-1.54686	-4.20841	-0.95665	-1.54685
H	2.120179	0.622907	1.337063	-1.59668	-0.32219	-1.39209	-1.59667	-0.3222	-1.39209
H	2.619045	-1.94417	0.952999	-2.66026	-2.54371	-0.17848	-2.66025	-2.54371	-0.17845
H	3.598036	-2.36243	-0.46473	-3.65068	-2.06087	1.194914	-3.65068	-2.06086	1.19493

H	1.940661	-1.41966	-1.96732	-1.69338	-1.24409	2.289186	-1.69338	-1.24406	2.289203
H	0.910131	-2.10635	-0.71763	-0.76432	-1.88313	0.947655	-0.76432	-1.88311	0.947686
H	-0.93176	-1.26824	-1.32548	0.513743	0.377369	-1.41305	0.513743	0.37736	-1.41305
H	-1.30276	0.422181	-1.67199	0.753755	-1.09014	-0.45993	0.753752	-1.09014	-0.45991
H	-2.67456	-1.31479	0.494601	2.662105	1.345745	-0.42796	2.662105	1.345742	-0.42796
H	-3.669	0.942285	-1.31893	3.113371	-1.68605	-0.41556	3.113368	-1.68605	-0.41555
H	-7.06946	0.466578	0.174709	5.62766	-2.03354	-1.22611	5.62767	-2.03354	-1.22612
H	-5.92547	1.792569	-0.17594	6.795634	-1.02119	-0.33117	6.795632	-1.0212	-0.33115
H	-6.31425	0.630615	-1.43541	5.585347	-2.01243	0.530418	5.585331	-2.01245	0.530416
H	-1.50908	1.510299	1.115718	1.758208	0.816725	2.092714	1.758206	0.816744	2.092711
H	0.197065	1.809877	1.466463	0.153657	0.759536	2.844513	0.153654	0.759561	2.844509
H	-0.48342	2.254913	-0.11241	0.886565	-0.71965	2.204879	0.886562	-0.71963	2.204887
H	4.655301	0.843243	-1.16695	-4.58699	0.99115	0.095903	-4.58699	0.991145	0.095886
H	3.564422	2.517012	0.503914	-3.04018	1.637447	-2.07178	-3.04018	1.637423	-2.07181
H	1.857132	2.813131	0.201883	-1.43444	2.126747	-1.51563	-1.43444	2.126732	-1.51565
H	2.889645	2.287749	-1.12141	-2.81623	2.328556	-0.45096	-2.81624	2.32855	-0.45099
H	-0.75838	-0.58533	1.879182	-0.26544	2.52187	1.11556	-0.26544	2.521883	1.115542
H	5.429004	-0.89151	0.995345	-5.04336	-1.59676	-1.24882	-5.04336	-1.59678	-1.24881
H	5.078868	0.828721	1.228532	-4.6194	-0.0905	-2.07541	-4.61939	-0.09053	-2.07542
H	4.181044	-0.38476	2.151205	-3.5783	-1.51158	-2.24693	-3.57829	-1.5116	-2.24691
	3a-13			3a-14			3a-15		
C	-1.3964	0.620119	0.738692	1.412386	0.892777	0.431604	-1.33699	0.177098	0.695111
C	-2.22164	0.447007	-0.56833	1.98021	-0.54664	0.583474	-2.11094	0.312228	-0.62866
C	-3.1493	-0.77183	-0.32831	3.109617	-0.66184	-0.46098	-3.42169	-0.45266	-0.33382
C	-2.32627	-1.64081	0.628168	2.614982	0.241755	-1.59391	-2.93605	-1.68417	0.438469
C	-1.70327	-0.63519	1.599606	2.050062	1.460424	-0.86292	-1.63162	-1.27027	1.152471
C	0.113342	0.88137	0.506044	-0.12938	0.982225	0.468162	0.15706	0.566961	0.700603
C	0.830987	-0.33452	-0.1105	-0.78078	0.287408	-0.75934	0.922924	-0.06716	-0.47364
C	2.279828	-0.082	-0.39018	-2.27645	0.349061	-0.73184	2.396897	0.186227	-0.42314
C	3.283	-0.84575	0.063908	-3.06581	-0.642	-0.29485	3.333133	-0.77205	-0.45729
C	4.714034	-0.60408	-0.22212	-4.54757	-0.51056	-0.25348	4.790137	-0.52158	-0.41353
O	5.560298	-1.36583	0.240293	-5.12375	0.506629	-0.62349	5.569081	-1.47119	-0.45685
C	5.11891	0.57618	-1.07479	-5.3031	-1.70447	0.272827	5.303818	0.896037	-0.31093
C	0.787259	1.298476	1.81731	-0.5698	2.447823	0.567421	0.791126	0.191481	2.045046
H	-1.7641	1.509749	1.265593	1.752371	1.484682	1.287973	-1.82089	0.852474	1.414099
O	-4.38661	-0.38561	0.293723	4.28965	-0.09031	0.129681	-4.24712	0.289372	0.581481
C	-2.95472	1.709122	-1.01421	2.403399	-0.89879	2.006805	-2.30047	1.735524	-1.14371
O	0.272664	1.932794	-0.45793	-0.52073	0.294885	1.661762	0.297835	1.983569	0.503695
C	-3.55106	-1.48761	-1.60756	3.395562	-2.09411	-0.89856	-4.22995	-0.80643	-1.57751
H	-1.54262	0.161567	-1.37776	1.214193	-1.26734	0.28045	-1.59661	-0.27377	-1.40143
H	-1.54259	-2.15536	0.061967	1.826431	-0.27762	-2.1496	-2.74742	-2.49843	-0.26556
H	-2.94291	-2.39964	1.116475	3.409914	0.488684	-2.30509	-3.71312	-2.02218	1.127479
H	-2.41303	-0.38223	2.393715	2.863217	2.140575	-0.60098	-1.71832	-1.32979	2.239162

H	-0.82652	-1.04101	2.10832	1.353929	2.029087	-1.4835	-0.82018	-1.94498	0.865627
H	0.346346	-0.57999	-1.06194	-0.46046	-0.75634	-0.78099	0.53744	0.373611	-1.39939
H	0.725854	-1.20128	0.545047	-0.42528	0.769767	-1.67398	0.727218	-1.14143	-0.51164
H	2.494409	0.785301	-1.01016	-2.75488	1.270468	-1.05893	2.691934	1.231465	-0.35748
H	3.082418	-1.71186	0.691155	-2.63701	-1.58482	0.038238	3.051227	-1.82121	-0.51795
H	4.771228	1.510929	-0.62847	-4.96798	-1.93604	1.287784	4.921053	1.379123	0.591314
H	6.20403	0.601001	-1.16569	-6.37516	-1.51263	0.272006	6.392533	0.884711	-0.28016
H	4.674292	0.501681	-2.07008	-5.0826	-2.58134	-0.34245	4.97255	1.488446	-1.16718
H	1.82973	1.573249	1.638698	-1.64859	2.52341	0.733246	1.793363	0.620678	2.120688
H	0.273692	2.162897	2.24992	-0.06764	2.928159	1.410808	0.187924	0.580531	2.871712
H	0.770524	0.493351	2.555797	-0.33362	3.006904	-0.34121	0.874409	-0.89119	2.166355
H	-4.19128	0.0994	1.106525	4.961882	-0.03457	-0.56261	-4.53629	1.096666	0.134813
H	-3.57216	1.517389	-1.89675	2.827253	-1.9067	2.057531	-2.96848	1.760432	-2.01058
H	-2.23853	2.493291	-1.27061	1.538185	-0.8588	2.671396	-1.34514	2.169578	-1.44189
H	-3.60904	2.092593	-0.22622	3.156038	-0.19769	2.375425	-2.7169	2.386071	-0.36732
H	-0.08717	2.744211	-0.0747	-1.48588	0.334014	1.728899	-0.17271	2.431158	1.220041
H	-4.18808	-2.34578	-1.3766	4.194198	-2.11542	-1.64752	-5.08781	-1.42585	-1.30169
H	-4.10668	-0.81756	-2.26949	3.713358	-2.70154	-0.04668	-4.60879	0.091671	-2.0759
H	-2.66681	-1.84575	-2.13986	2.506833	-2.55295	-1.3403	-3.61974	-1.35924	-2.29664
	3a-16			3a-17			3a-18		
C	-1.41259	0.625244	0.726125	-1.41247	0.62493	0.726303	1.208805	0.110135	-0.56266
C	-2.25708	0.417325	-0.56188	-2.25704	0.417539	-0.56176	2.366709	0.696951	0.292924
C	-3.14673	-0.81248	-0.28658	-3.14689	-0.81217	-0.28676	3.568841	-0.24341	0.073671
C	-2.27531	-1.65833	0.646306	-2.27554	-1.65852	0.645747	2.89704	-1.60689	-0.09296
C	-1.66577	-0.6308	1.600535	-1.6658	-0.63138	1.600287	1.691344	-1.30663	-0.98409
C	0.083041	0.931387	0.462192	0.083205	0.930926	0.462522	-0.16615	0.10536	0.154382
C	0.826726	-0.26482	-0.16257	0.826662	-0.26509	-0.16292	-1.26321	-0.34028	-0.84524
C	2.264159	0.022772	-0.46143	2.264058	0.022515	-0.46195	-2.62671	-0.42212	-0.23503
C	3.29761	-0.70696	-0.02155	3.297606	-0.70675	-0.02151	-3.71984	0.17502	-0.72963
C	4.702426	-0.37004	-0.36813	4.702379	-0.36982	-0.36825	-5.06775	0.082566	-0.12753
O	4.993425	0.590864	-1.07384	4.993263	0.590941	-1.07421	-6.0094	0.666327	-0.65941
C	5.761716	-1.28121	0.201466	5.761799	-1.28074	0.201478	-5.27086	-0.72401	1.134419
C	0.768891	1.374778	1.758797	0.769143	1.373609	1.759323	-0.52629	1.47538	0.722921
H	-1.80119	1.5055	1.252819	-1.80097	1.50503	1.253349	1.086924	0.727608	-1.45904
O	-4.30294	-0.34432	0.428581	-4.30289	-0.34406	0.428765	4.185483	0.143507	-1.16621
C	-3.02584	1.657342	-1.0104	-3.02555	1.657842	-1.00996	2.682034	2.164503	0.010969
O	0.192112	1.983493	-0.50882	0.192496	1.983547	-0.50792	-0.1498	-0.77384	1.287606
C	-3.58465	-1.54578	-1.54941	-3.58507	-1.54491	-1.54983	4.597997	-0.20008	1.197622
H	-1.58983	0.131842	-1.38137	-1.58984	0.132155	-1.38133	2.09679	0.587736	1.349725
H	-1.49457	-2.15373	0.05875	-1.49493	-2.15389	0.058016	2.574303	-1.9687	0.889225
H	-2.8495	-2.4394	1.154857	-2.84994	-2.43954	1.154121	3.574975	-2.35536	-0.51516
H	-2.38384	-0.39053	2.387374	-2.38379	-0.39126	2.387237	1.999033	-1.29243	-2.03127
H	-0.76834	-1.00652	2.097239	-0.7684	-1.00742	2.096788	0.925976	-2.08411	-0.90511

H	0.336304	-0.52274	-1.10788	0.335989	-0.52264	-1.10817	-1.01184	-1.33475	-1.23231
H	0.749946	-1.13352	0.49453	0.749958	-1.13398	0.493944	-1.26973	0.340746	-1.70059
H	2.472351	0.887666	-1.08783	2.472226	0.887009	-1.0889	-2.70043	-1.01987	0.671238
H	3.13417	-1.5753	0.613291	3.13427	-1.57476	0.613818	-3.66208	0.782458	-1.63042
H	5.590121	-2.30605	-0.13977	5.590646	-2.30555	-0.14011	-4.6415	-0.3414	1.941492
H	6.755168	-0.95313	-0.10158	6.755222	-0.95225	-0.10122	-6.31633	-0.6686	1.434792
H	5.692216	-1.2935	1.29286	5.692013	-1.29339	1.292846	-4.99672	-1.76896	0.970838
H	1.797545	1.685004	1.559339	1.79778	1.683907	1.559951	-1.51562	1.443775	1.186446
H	0.234391	2.221782	2.200572	0.234695	2.220412	2.201533	0.189377	1.782906	1.487161
H	0.793917	0.570906	2.498525	0.794176	0.569351	2.498628	-0.54321	2.229037	-0.06808
H	-4.80264	-1.12208	0.711158	-4.80336	-1.12177	0.710065	4.850162	-0.52477	-1.38046
H	-3.64974	1.4438	-1.88374	-3.64997	1.444532	-1.88297	3.523052	2.511736	0.618746
H	-2.33029	2.453974	-1.28548	-2.32986	2.454211	-1.28544	1.826673	2.804595	0.236983
H	-3.67621	2.025944	-0.21365	-3.67541	2.026739	-0.21292	2.943745	2.308243	-1.03977
H	-0.19497	2.781797	-0.12467	-0.1955	2.781383	-0.1237	0.037294	-1.67115	0.979559
H	-4.20124	-2.41409	-1.29493	-4.20198	-2.41311	-1.2957	5.411691	-0.90536	0.999193
H	-4.17491	-0.88777	-2.19303	-4.17509	-0.88651	-2.19326	5.031576	0.799514	1.287656
H	-2.71927	-1.90081	-2.11551	-2.7198	-1.90007	-2.116	4.143744	-0.46987	2.154692
	3a-19			3a-20			3a-21		
C	-0.61795	0.193557	-0.04599	1.361803	0.606472	0.434803	1.215556	0.1361	-0.57178
C	-1.95481	0.049748	0.735116	2.001953	-0.7193	-0.01599	2.37097	0.666856	0.320489
C	-2.81231	-0.92917	-0.09174	3.429654	-0.28499	-0.41958	3.552781	-0.30699	0.092538
C	-2.39834	-0.60408	-1.527	3.188653	1.029126	-1.1695	2.847107	-1.64073	-0.11734
C	-0.88153	-0.4313	-1.44321	1.905605	1.645925	-0.57294	1.643967	-1.29681	-0.99651
C	-0.07788	1.636538	-0.11766	-0.16974	0.638942	0.63527	-0.17441	0.170414	0.100122
C	1.298842	1.678721	-0.83453	-0.91233	0.010732	-0.55959	-1.25368	-0.1844	-0.95911
C	2.364754	0.863665	-0.17168	-2.39628	0.208883	-0.53101	-2.61003	-0.4215	-0.37531
C	2.934341	-0.21842	-0.72041	-3.28627	-0.77392	-0.33803	-3.6947	0.323104	-0.63149
C	3.973925	-1.04334	-0.066	-4.75308	-0.5832	-0.3124	-5.03447	0.08267	-0.05029
O	4.429411	-2.01993	-0.65609	-5.48601	-1.55283	-0.13177	-5.96346	0.830426	-0.34584
C	4.457298	-0.68013	1.318874	-5.33282	0.799375	-0.50387	-5.24339	-1.07346	0.900182
C	0.034787	2.284171	1.265866	-0.62524	2.078425	0.907471	-0.49786	1.535387	0.710461
H	0.147556	-0.39428	0.470558	1.792665	0.846667	1.416763	1.141667	0.769882	-1.46232
O	-2.37789	-2.2583	0.24661	4.204298	0.057866	0.743132	4.224917	-0.02294	-1.14679
C	-1.79356	-0.36765	2.195763	1.93878	-1.86297	0.99151	2.73329	2.131592	0.081253
O	-1.00746	2.381537	-0.91414	-0.53468	-0.17691	1.758984	-0.15491	-0.82689	1.131859
C	-4.31053	-0.79937	0.160019	4.184632	-1.31808	-1.24896	4.56443	-0.32082	1.233176
H	-2.48042	1.010511	0.69655	1.521768	-1.04707	-0.947	2.079548	0.542275	1.369814
H	-2.86843	0.336595	-1.83062	3.053721	0.815071	-2.23266	2.508601	-2.01615	0.852872
H	-2.71239	-1.37891	-2.23427	4.061247	1.679206	-1.07552	3.51505	-2.38473	-0.5596
H	-0.3863	-1.40292	-1.51207	2.096174	2.603803	-0.08501	1.93347	-1.30119	-2.04976
H	-0.50827	0.182632	-2.26358	1.180601	1.839237	-1.3687	0.846262	-2.03133	-0.8746
H	1.625222	2.726044	-0.85712	-0.68655	-1.05769	-0.57995	-0.94876	-1.09942	-1.47619

H	1.172685	1.358737	-1.87024	-0.52306	0.451385	-1.48354	-1.2951	0.616256	-1.70284
H	2.674928	1.184704	0.820894	-2.75109	1.224854	-0.69097	-2.69171	-1.27099	0.300386
H	2.637274	-0.55762	-1.71064	-2.95473	-1.79811	-0.18149	-3.63563	1.175442	-1.30515
H	3.627286	-0.68765	2.029577	-5.04129	1.208282	-1.47427	-4.59643	-0.9735	1.775122
H	5.211789	-1.3979	1.638005	-6.41923	0.746759	-0.44694	-6.28366	-1.09318	1.222203
H	4.888	0.324031	1.325657	-4.96276	1.481401	0.265483	-4.99693	-2.02087	0.414917
H	0.506852	3.269366	1.188921	-1.65888	2.087178	1.261226	-1.51539	1.544384	1.114694
H	-0.95461	2.420511	1.706371	0.000324	2.529436	1.684312	0.188766	1.773384	1.525296
H	0.63392	1.677983	1.949959	-0.55877	2.70633	0.015593	-0.43566	2.324903	-0.04262
H	-2.79793	-2.86651	-0.3762	4.339881	-0.74848	1.259027	4.685395	0.821236	-1.0505
H	-2.76634	-0.50064	2.679026	2.528594	-2.72216	0.656374	3.600358	2.432845	0.678639
H	-1.24513	0.385028	2.766118	0.910155	-2.19679	1.13458	1.911316	2.797507	0.350167
H	-1.24865	-1.31165	2.270398	2.311592	-1.54924	1.972368	2.963133	2.31296	-0.97348
H	-0.7358	3.309305	-0.90616	-0.06222	0.15813	2.533253	-0.81608	-0.59582	1.797066
H	-4.86657	-1.5016	-0.46979	5.140271	-0.90393	-1.58202	5.345029	-1.05965	1.032608
H	-4.54936	-1.02027	1.203929	4.393502	-2.2214	-0.66675	5.045211	0.656289	1.350369
H	-4.65995	0.210507	-0.07086	3.607258	-1.61037	-2.13004	4.081759	-0.57372	2.180698
	3a-22			3a-23			3a-24		
C	1.21505	-0.30787	-0.66902	1.363098	0.602772	0.460384	1.226571	0.156717	-0.56522
C	2.075278	0.770883	0.049733	2.014073	-0.72987	0.036563	2.408616	0.680262	0.32699
C	3.483575	0.153777	0.203558	3.408019	-0.29074	-0.44717	3.586415	-0.31609	0.077873
C	3.188722	-1.34333	0.32886	3.092279	0.967415	-1.26688	2.870761	-1.65051	-0.15885
C	2.095069	-1.58239	-0.71133	1.89479	1.637524	-0.56499	1.648744	-1.28167	-1.02117
C	-0.17657	-0.55463	-0.04464	-0.16957	0.623359	0.657222	-0.16904	0.183881	0.13153
C	-0.99145	0.766976	-0.04419	-0.90515	-0.00228	-0.5434	-1.28028	-0.21132	-0.8781
C	-2.39801	0.58919	0.436831	-2.38837	0.202498	-0.52801	-2.63796	-0.37345	-0.25631
C	-3.46165	0.448519	-0.36604	-3.28459	-0.77527	-0.33811	-3.75979	0.227508	-0.69593
C	-4.83299	0.237104	0.172057	-4.7506	-0.57729	-0.32583	-5.10462	0.055491	-0.11207
O	-5.06324	0.195468	1.375791	-5.48989	-1.54237	-0.14675	-6.07506	0.673615	-0.60189
C	-5.92755	0.074242	-0.85202	-5.32186	0.80711	-0.52947	-5.30043	-0.86907	1.07561
C	-0.11678	-1.16032	1.362005	-0.63503	2.058115	0.939602	-0.49765	1.542069	0.773014
H	1.014448	0.022488	-1.69369	1.787096	0.862624	1.439588	1.147908	0.81044	-1.44461
O	4.188979	0.404342	-1.02372	4.159683	0.061285	0.72697	4.2386	-0.01648	-1.2097
C	2.077352	2.13558	-0.6373	2.01797	-1.82973	1.092578	2.7859	2.148125	0.076162
O	-0.80297	-1.48561	-0.9364	-0.5339	-0.20111	1.774741	-0.23519	-0.86448	1.170013
C	4.27376	0.725348	1.37568	4.161197	-1.35165	-1.24171	4.63755	-0.34324	1.188848
H	1.701232	0.907334	1.070827	1.504392	-1.10514	-0.85976	2.137141	0.565815	1.389489
H	2.81932	-1.55395	1.338382	2.822063	0.671732	-2.2849	2.544148	-2.06027	0.805802
H	4.084069	-1.95384	0.172688	3.965946	1.621148	-1.34413	3.537372	-2.37464	-0.63557
H	2.547149	-1.67805	-1.70108	2.18066	2.564238	-0.06354	1.93383	-1.2677	-2.07689
H	1.524303	-2.49524	-0.53253	1.13229	1.904883	-1.3012	0.843504	-2.00767	-0.88916
H	-0.99147	1.159446	-1.06475	-0.68405	-1.07167	-0.55952	-0.99329	-1.16765	-1.33457
H	-0.49076	1.493175	0.602089	-0.50566	0.434864	-1.46472	-1.30836	0.536267	-1.68025

H	-2.56672	0.542276	1.51121	-2.73709	1.219491	-0.69486	-2.66732	-1.04141	0.601392
H	-3.34876	0.491622	-1.44715	-2.95928	-1.80035	-0.17456	-3.7326	0.903034	-1.5488
H	-5.7012	-0.77509	-1.50283	-5.02099	1.208786	-1.50005	-4.68867	-0.554	1.929276
H	-6.8902	-0.07859	-0.3661	-6.40892	0.760305	-0.48049	-6.35291	-0.85167	1.363027
H	-5.97077	0.961112	-1.49047	-4.95413	1.491834	0.238586	-5.01243	-1.89752	0.827322
H	-1.12168	-1.40314	1.719595	-1.66886	2.056257	1.293073	-1.49504	1.513012	1.220983
H	0.460121	-2.08696	1.360722	-0.01298	2.50684	1.720536	0.221204	1.801319	1.558046
H	0.331201	-0.47191	2.082843	-0.57386	2.694963	0.05383	-0.48365	2.343413	0.025912
H	5.015709	-0.09524	-0.98881	4.99809	0.441317	0.432718	4.77566	0.797473	-1.12783
H	2.736153	2.838265	-0.11843	2.573431	-2.70649	0.746224	3.642544	2.456166	0.689767
H	1.075429	2.571293	-0.65085	0.998204	-2.14195	1.323729	1.959222	2.824209	0.312468
H	2.421809	2.048824	-1.66991	2.481607	-1.4774	2.017932	3.043085	2.297916	-0.9787
H	-1.68843	-1.68066	-0.59756	-0.05425	0.122266	2.549583	0.310115	-0.60575	1.939735
H	5.256356	0.24722	1.444871	5.101596	-0.94373	-1.62723	5.423735	-1.06411	0.942429
H	4.428212	1.800564	1.250194	4.40004	-2.21185	-0.61125	5.107349	0.64047	1.320966
H	3.749522	0.557008	2.320261	3.569965	-1.69715	-2.09399	4.191963	-0.63062	2.148045

Table S 15. Energy analysis for 2*S*, 3*R*, 6*R*, 7*R*-3

Conf.	Steric Energy (kJ/mol)	Relative Energy (kJ/mol)	Distribution (%)
3a-1	-773.149	0.000499	6.21%
3a-2	-773.149	0.000113	9.34%
3a-3	-773.147	0.002224	1.00%
3a-4	-773.147	0.002236	0.99%
3a-5	-773.148	0.001295	2.67%
3a-6	-773.147	0.00217	1.06%
3a-7	-773.147	0.002173	1.06%
3a-8	-773.147	0.002474	0.77%
3a-9	-773.149	0.000465	6.44%
3a-10	-773.147	0.001541	2.06%
3a-11	-773.149	0	10.53%
3a-12	-773.149	0	10.53%
3a-13	-773.148	0.001163	3.07%
3a-14	-773.147	0.001672	1.79%
3a-15	-773.149	0.000114	9.33%
3a-16	-773.148	0.000732	4.85%
3a-17	-773.148	0.000725	4.89%
3a-18	-773.148	0.00121	2.93%
3a-19	-773.148	0.000671	5.18%
3a-20	-773.148	0.001126	3.20%
3a-21	-773.147	0.001973	1.30%
3a-22	-773.147	0.001873	1.45%
3a-23	-773.147	0.001835	1.51%
3a-24	-773.149	0.000278	7.85%

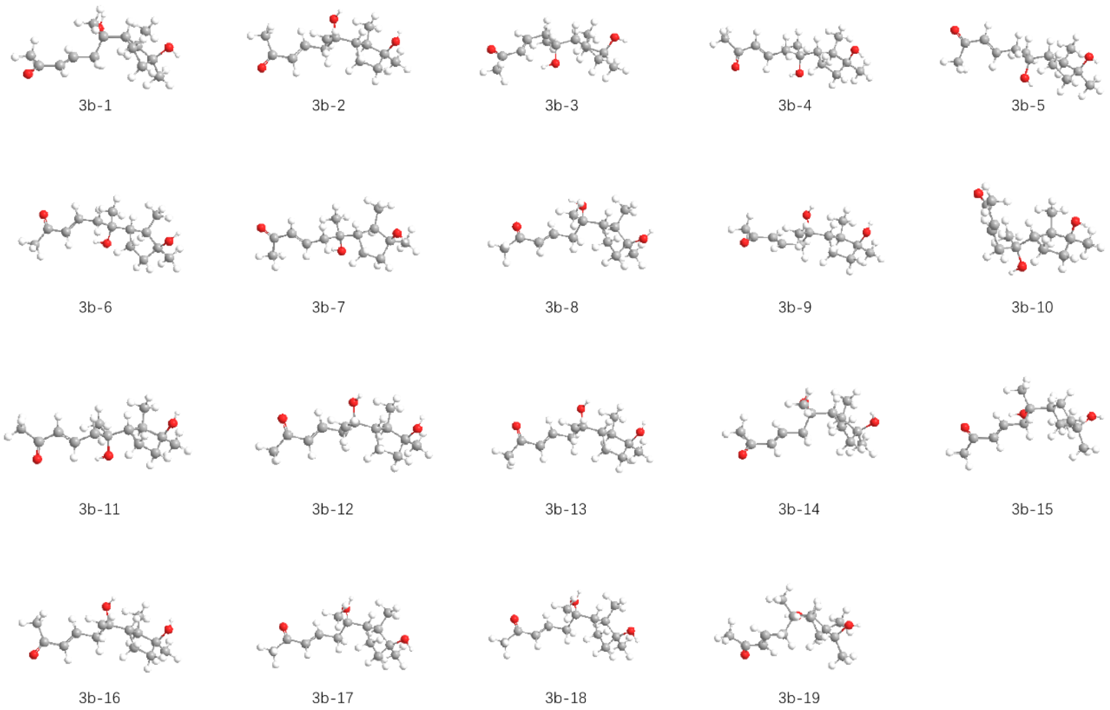

Figure S 82. Most stable conformers of 2*R*, 3*S*, 6*S*, 7*S*-3 at the CAM-B3LYP/DGDZVP level

Table S 16. Optimized Z-Matrixes of 2*R*, 3*S*, 6*S*, 7*S*-3 conformations in the methanol at CAM-B3LYP/DGDZVP level

	3b-1			3b-2			3b-3		
C	1.394937	0.608427	0.74754	1.336989	0.177046	0.695123	-1.23644	0.249049	-0.6288
C	2.221085	0.459049	-0.56061	2.110939	0.312271	-0.62864	-2.40638	0.606707	0.331429
C	3.146366	-0.75533	-0.33745	3.421691	-0.45263	-0.33385	-3.40986	-0.55743	0.211865
C	2.317314	-1.64737	0.591	2.936062	-1.68419	0.438351	-2.49567	-1.76185	-0.0105
C	1.702081	-0.66105	1.584239	1.631628	-1.27035	1.152385	-1.44054	-1.24868	-0.99054
C	-0.11403	0.87412	0.518161	-0.15706	0.566899	0.700639	0.170192	0.545725	-0.06212
C	-0.83229	-0.33003	-0.12034	-0.92292	-0.06715	-0.47364	1.219233	0.326155	-1.19096
C	-2.28191	-0.07487	-0.39279	-2.3969	0.186244	-0.42314	2.629685	0.526917	-0.73061
O	-0.27453	1.943635	-0.42682	-0.29785	1.983519	0.50382	0.386574	-0.39372	0.994924
C	3.573417	-1.44377	-1.62896	4.229964	-0.80631	-1.57756	-4.33356	-0.70148	1.416398
H	1.765898	1.486695	1.289975	1.82088	0.852377	1.414157	-1.33492	0.85762	-1.53401
C	2.950826	1.730763	-0.98544	2.300459	1.735604	-1.14359	-3.0334	1.97714	0.082146
O	4.306639	-0.27758	0.3645	4.247112	0.289345	0.58151	-4.19099	-0.31959	-0.97249
C	-0.7893	1.267876	1.836049	-0.79113	0.191329	2.045058	0.291325	1.969315	0.490786
C	-3.2831	-0.84805	0.049896	-3.33314	-0.77202	-0.45735	3.439239	-0.46244	-0.32744
C	-4.71498	-0.6046	-0.22942	-4.79014	-0.52154	-0.41356	4.82165	-0.20309	0.158291
C	-5.12383	0.588683	-1.06187	-5.30381	0.896076	-0.31094	5.621016	-1.41773	0.557347
O	-5.55904	-1.3758	0.221414	-5.56909	-1.47115	-0.45686	5.287623	0.928981	0.230679
H	1.546516	0.181118	-1.37671	1.596614	-0.27368	-1.40145	-2.0307	0.568585	1.360209
H	2.922266	-2.4257	1.067092	3.71313	-2.02226	1.127334	-3.04501	-2.63525	-0.37738
H	1.537292	-2.14847	0.00738	2.747429	-2.4984	-0.26574	-2.02756	-2.03627	0.940088

H	0.827085	-1.07367	2.091484	0.820192	-1.94504	0.865501	-0.52143	-1.83137	-0.91846
H	2.431321	-0.42011	2.360542	1.718338	-1.32994	2.239072	-1.8054	-1.33606	-2.01645
H	-0.72426	-1.20879	0.518593	-0.72723	-1.14142	-0.5117	0.998306	1.0271	-2.00153
H	-0.34885	-0.55659	-1.07709	-0.53743	0.373667	-1.39936	1.104591	-0.68652	-1.58166
H	-2.49934	0.80269	-0.99723	-2.69192	1.231481	-0.35742	3.016361	1.543515	-0.68917
H	0.09729	2.744499	-0.03297	0.172715	2.431065	1.22018	1.26308	-0.22952	1.370698
H	4.133695	-0.75466	-2.2668	4.608794	0.091834	-2.07588	-4.94927	0.193132	1.543029
H	4.215595	-2.30359	-1.41171	5.08783	-1.42574	-1.30178	-5.00368	-1.55723	1.283767
H	2.704303	-1.80415	-2.18586	3.619758	-1.35906	-2.29673	-3.75933	-0.86139	2.332882
H	3.566733	1.55785	-1.87331	2.968472	1.760575	-2.01046	-3.87226	2.158102	0.761162
H	2.231333	2.516459	-1.22839	1.345132	2.169674	-1.44174	-2.30889	2.780002	0.23387
H	3.603292	2.094526	-0.18821	2.716893	2.386098	-0.36715	-3.40743	2.048983	-0.94179
H	4.832679	-1.04946	0.613245	4.536281	1.09667	0.134899	-4.70794	-1.11862	-1.14175
H	-0.77432	0.449034	2.559376	-0.8744	-0.89135	2.166299	0.088902	2.713741	-0.28395
H	-0.2746	2.123196	2.285133	-0.18793	0.580335	2.871748	-0.40372	2.124058	1.317581
H	-1.83122	1.547778	1.661733	-1.79337	0.620513	2.120726	1.298898	2.150417	0.877072
H	-3.07962	-1.72447	0.661735	-3.05124	-1.82118	-0.51807	3.105278	-1.49745	-0.35757
H	-4.68199	0.530326	-2.05951	-4.97242	1.488555	-1.16709	5.710157	-2.09657	-0.29554
H	-6.20924	0.61346	-1.1493	-6.39253	0.884767	-0.28029	6.612062	-1.12966	0.905262
H	-4.77605	1.51678	-0.602	-4.92115	1.379076	0.591398	5.095259	-1.96461	1.345058
	3b-4			3b-5			3b-6		
C	-1.22322	0.081929	-0.55394	-1.20889	0.102094	-0.54118	-1.23474	0.247973	0.247973
C	-2.38381	0.70914	0.267515	-2.37766	0.688832	0.301466	-2.4044	0.59594	0.59594
C	-3.58628	-0.23435	0.068034	-3.5764	-0.24871	0.052037	-3.41139	-0.573	-0.573
C	-2.91877	-1.60668	-0.01599	-2.90239	-1.61281	-0.09469	-2.5004	-1.77013	-1.77013
C	-1.68511	-1.36502	-0.88622	-1.66628	-1.32538	-0.94742	-1.43674	-1.24687	-1.24687
C	0.151678	0.121698	0.149021	0.17023	0.106255	0.167886	0.171442	0.543867	0.543867
C	1.241956	-0.33478	-0.85803	1.257273	-0.31409	-0.84292	1.220646	0.331172	0.331172
C	2.589141	-0.53318	-0.24192	2.624763	-0.41654	-0.24392	2.630842	0.531665	0.531665
O	0.077777	-0.80306	1.243535	0.20948	-0.88465	1.205673	0.388831	-0.40011	-0.40011
C	-4.64144	-0.13094	1.163829	-4.63659	-0.2008	1.146675	-4.32759	-0.73089	-0.73089
H	-1.10965	0.648398	-1.4846	-1.09767	0.718741	-1.43971	-1.33444	0.863166	0.863166
C	-2.69054	2.1664	-0.07256	-2.68295	2.159732	0.025288	-3.03151	1.968099	1.968099
O	-4.1725	0.09644	-1.20352	-4.15595	0.142454	-1.20391	-4.20265	-0.44142	-0.44142
C	0.502899	1.514033	0.676572	0.522389	1.474225	0.757384	0.289578	1.965174	1.965174
C	3.698445	0.138603	-0.57689	3.713135	0.202159	-0.72246	3.441727	-0.45838	-0.45838
C	5.005953	-0.1242	0.079748	5.066374	0.084879	-0.13725	4.82368	-0.19903	-0.19903
C	6.16537	0.716567	-0.39396	5.27978	-0.76949	1.091179	5.624647	-1.41404	-1.41404
O	5.141319	-0.9719	0.956439	6.004297	0.687357	-0.65485	5.287959	0.933416	0.933416
H	-2.12617	0.637973	1.330582	-2.14497	0.582535	1.369475	-2.03006	0.555241	0.555241
H	-3.59303	-2.37125	-0.41601	-3.57213	-2.36054	-0.53175	-2.03861	-2.05692	-2.05692
H	-2.6167	-1.91938	0.988475	-2.60452	-1.97364	0.895645	-3.05686	-2.6294	-2.6294
H	-0.91442	-2.11206	-0.69067	-0.89364	-2.07762	-0.78219	-0.51792	-1.82969	-1.82969

H	-1.94944	-1.43656	-1.94369	-1.92588	-1.34682	-2.00801	-1.79148	-1.32749	-1.32749
H	1.300112	0.394735	-1.67082	1.25929	0.39143	-1.67763	0.998776	1.035642	1.035642
H	0.932453	-1.28984	-1.29421	0.99653	-1.29782	-1.2472	1.107345	-0.67981	-0.67981
H	2.672929	-1.29253	0.53454	2.704592	-1.0516	0.635451	3.016229	1.548546	1.548546
H	0.747178	-0.56232	1.896866	-0.34902	-0.58458	1.934721	1.265941	-0.23806	-0.23806
H	-5.07416	0.872658	1.193447	-5.06997	0.799933	1.224134	-4.96024	0.152539	0.152539
H	-5.45273	-0.84366	0.982438	-5.44618	-0.90373	0.924996	-4.9811	-1.5969	-1.5969
H	-4.21046	-0.35308	2.143729	-4.2111	-0.47212	2.116576	-3.74658	-0.87393	-0.87393
H	-3.5382	2.538551	0.5109	-3.53511	2.504703	0.61838	-3.89507	2.135195	2.135195
H	-1.83703	2.813314	0.140761	-1.83061	2.79575	0.272664	-2.32156	2.774218	2.774218
H	-2.93846	2.272087	-1.13122	-2.92253	2.309928	-1.02968	-3.36023	2.071927	2.071927
H	-4.82153	-0.5895	-1.40953	-4.80277	-0.53333	-1.44715	-4.79735	0.311388	0.311388
H	0.494082	2.251688	-0.12979	0.526127	2.245566	-0.01741	0.08069	2.712663	2.712663
H	-0.20129	1.829927	1.448699	-0.19045	1.772211	1.530841	-0.40222	2.112472	2.112472
H	1.506154	1.516882	1.11529	1.514471	1.442662	1.214951	1.297922	2.14858	2.14858
H	3.671225	0.905722	-1.34779	3.64744	0.845731	-1.5973	3.1093	-1.49374	-1.49374
H	6.315579	0.567943	-1.46708	5.002943	-1.80727	0.89064	5.714736	-2.08963	-2.08963
H	5.937842	1.776354	-0.24859	6.327944	-0.7263	1.384145	6.615269	-1.1259	-1.1259
H	7.07522	0.45674	0.145415	4.657871	-0.41727	1.917635	5.099555	-1.9645	-1.9645
	3b-7			3b-8			3b-9		
C	-1.21653	0.12721	-0.5645	1.405833	0.614045	0.744647	-1.38988	0.622944	0.417336
C	-2.37361	0.677488	0.314774	2.242104	0.434507	-0.55347	-1.98194	-0.73699	0.004069
C	-3.55408	-0.28832	0.092756	3.156974	-0.79026	-0.2992	-3.42625	-0.36639	-0.40379
C	-2.85151	-1.63505	-0.07693	2.318366	-1.65287	0.639702	-3.23611	0.934463	-1.19029
C	-1.6445	-1.31468	-0.95913	1.694818	-0.64337	1.604774	-1.97609	1.614994	-0.61428
C	0.173381	0.17137	0.107164	-0.09435	0.887885	0.498001	0.140104	0.717261	0.610398
C	1.250774	-0.1987	-0.94873	-0.81621	-0.34323	-0.10902	0.901965	0.088577	-0.57195
C	2.608884	-0.42799	-0.36601	-2.2351	-0.07418	-0.49722	2.378465	0.331586	-0.55106
O	0.154458	-0.81147	1.152871	-0.13377	1.974733	-0.43938	0.537953	-0.05768	1.752113
C	-4.58596	-0.27176	1.215166	3.582813	-1.50608	-1.57613	-4.14667	-1.44889	-1.20008
H	-1.14173	0.742844	-1.4675	1.774717	1.501078	1.270089	-1.82607	0.872216	1.394633
C	-2.72843	2.141099	0.059225	2.986128	1.696166	-0.9837	-1.87157	-1.85051	1.040744
O	-4.17874	0.090731	-1.14656	4.321236	-0.41892	0.458336	-4.20816	-0.01971	0.752942
C	0.4989	1.543665	0.699258	-0.77296	1.321021	1.799993	0.542628	2.178548	0.846515
C	3.692622	0.312824	-0.63686	-3.30612	-0.67805	0.033566	3.304114	-0.61506	-0.35129
C	5.035144	0.07968	-0.05908	-4.68927	-0.35689	-0.40758	4.75773	-0.30716	-0.3509
C	5.248967	-1.06538	0.9036	-5.79746	-1.10898	0.286234	5.681182	-1.47124	-0.08983
O	5.962572	0.823947	-0.368	-4.92251	0.470829	-1.28259	5.189831	0.824074	-0.55018
H	-2.08869	0.558098	1.366563	1.575471	0.15493	-1.37573	-1.49327	-1.0707	-0.92039
H	-3.51373	-2.39677	-0.50176	2.924371	-2.41595	1.135179	-4.13231	1.553806	-1.11202
H	-2.51855	-1.99024	0.903358	1.543371	-2.16256	0.057857	-3.09484	0.696121	-2.24747
H	-0.84856	-2.04715	-0.81622	0.812943	-1.04347	2.110258	-1.26134	1.816143	-1.4173
H	-1.92903	-1.34267	-2.01336	2.415976	-0.39187	2.385222	-2.20213	2.576745	-0.14964

H	1.290483	0.591441	-1.70372	-0.77903	-1.17737	0.595668	0.498498	0.49957	-1.50367
H	0.944252	-1.12069	-1.45241	-0.27761	-0.64778	-1.01258	0.707808	-0.9863	-0.57157
H	2.693088	-1.26822	0.320867	-2.40802	0.664315	-1.27917	2.724968	1.3492	-0.72304
H	0.818147	-0.57252	1.812766	-1.00356	2.392904	-0.38842	0.053363	0.276404	2.519272
H	-5.04542	0.716168	1.306524	4.166719	-0.84337	-2.22392	-4.31934	-2.34248	-0.59151
H	-5.38131	-0.99702	1.01409	4.201236	-2.37423	-1.33252	-5.11845	-1.0794	-1.539
H	-4.12666	-0.53191	2.172593	2.711901	-1.84692	-2.14221	-3.56306	-1.74449	-2.07594
H	-3.57436	2.456362	0.677641	3.60361	1.518234	-1.87097	-2.41954	-2.74427	0.725779
H	-1.88977	2.800872	0.291027	2.272544	2.485735	-1.22563	-0.82897	-2.13132	1.196699
H	-3.00084	2.295404	-0.98745	3.63301	2.068545	-0.18291	-2.2633	-1.52887	2.011678
H	-4.81327	-0.60203	-1.37387	4.872901	0.145413	-0.09955	-4.31591	-0.81677	1.289382
H	0.44086	2.323137	-0.06444	-0.75176	0.525744	2.548638	0.449738	2.781826	-0.0598
H	-0.18926	1.7944	1.508946	-0.26548	2.19776	2.210296	-0.09648	2.6245	1.615332
H	1.515571	1.555209	1.1058	-1.82288	1.579439	1.628139	1.576504	2.234058	1.194755
H	3.630651	1.156041	-1.32166	-3.19088	-1.42715	0.813981	3.011	-1.64789	-0.17623
H	5.00077	-2.01831	0.430164	-5.66522	-2.18406	0.134679	5.443284	-1.92353	0.877044
H	6.290736	-1.08095	1.220992	-5.74776	-0.93131	1.364172	5.52566	-2.24317	-0.8489
H	4.605972	-0.95586	1.780306		-0.79959	-0.09499	6.721179	-1.1477	-0.10068
	3b-13			3b-14			3b-15		
C	1.353905	0.211406	0.68756	1.396401	0.620119	0.738692	-1.41239	0.892777	0.431604
C	2.132094	0.271109	-0.63954	2.221643	0.447007	-0.56833	-1.98021	-0.54664	0.583474
C	3.414259	-0.53026	-0.31701	3.149301	-0.77183	-0.32831	-3.10962	-0.66184	-0.46098
C	2.884479	-1.71493	0.497704	2.326265	-1.64081	0.628168	-2.61498	0.241755	-1.59391
C	1.600778	-1.22607	1.201499	1.703272	-0.63519	1.599606	-2.05006	1.460424	-0.86292
C	-0.12682	0.649867	0.676159	-0.11334	0.88137	0.506044	0.129376	0.982225	0.468162
C	-0.91475	-0.00935	-0.46974	-0.83099	-0.33452	-0.1105	0.780782	0.287408	-0.75934
C	-2.37866	0.294844	-0.43533	-2.27983	-0.082	-0.39018	2.27645	0.349061	-0.73184
O	-0.22094	2.06031	0.418964	-0.27266	1.932794	-0.45793	0.520733	0.294885	1.661762
C	4.208413	-0.95665	-1.54685	3.551063	-1.48761	-1.60756	-3.39556	-2.09411	-0.89856
H	1.85934	0.898139	1.380535	1.764095	1.509749	1.265593	-1.75237	1.484682	1.287973
C	2.373621	1.667664	-1.20391	2.954722	1.709122	-1.01421	-2.4034	-0.89879	2.006805
O	4.267032	0.212785	0.571884	4.386612	-0.38561	0.293723	-4.28965	-0.09031	0.129681
C	-0.77012	0.353567	2.035886	-0.78726	1.298476	1.817311	0.569796	2.447823	0.567421
C	-3.35231	-0.62463	-0.41699	-3.283	-0.84575	0.063908	3.065812	-0.642	-0.29485
C	-4.78932	-0.24881	-0.38882	-4.71403	-0.60408	-0.22212	4.547574	-0.51056	-0.25348
C	-5.77207	-1.39308	-0.35145	-5.11891	0.57618	-1.07479	5.303099	-1.70447	0.272827
O	-5.1636	0.919965	-0.39303	-5.5603	-1.36583	0.240293	5.123754	0.506629	-0.62349
H	1.596671	-0.3222	-1.39209	1.542618	0.161567	-1.37776	-1.21419	-1.26734	0.28045
H	3.65068	-2.06086	1.19493	2.942905	-2.39964	1.116475	-3.40991	0.488684	-2.30509
H	2.660253	-2.54371	-0.17845	1.542589	-2.15536	0.061967	-1.82643	-0.27762	-2.1496
H	0.764322	-1.88311	0.947686	0.826518	-1.04101	2.10832	-1.35393	2.029087	-1.4835
H	1.693384	-1.24406	2.289203	2.413032	-0.38223	2.393715	-2.86322	2.140575	-0.60098
H	-0.75375	-1.09014	-0.45991	-0.72585	-1.20128	0.545047	0.425275	0.769767	-1.67398

H	-0.51374	0.37736	-1.41305	-0.34635	-0.57999	-1.06194	0.460455	-0.75634	-0.78099
H	-2.66211	1.345742	-0.42796	-2.49441	0.785301	-1.01016	2.754879	1.270468	-1.05893
H	0.265437	2.521883	1.115542	0.087165	2.744211	-0.0747	1.48588	0.334014	1.728899
H	4.619391	-0.09053	-2.07542	4.106675	-0.81756	-2.26949	-3.71336	-2.70154	-0.04668
H	5.043357	-1.59678	-1.24881	4.188078	-2.34578	-1.3766	-4.1942	-2.11542	-1.64752
H	3.57829	-1.5116	-2.24691	2.666809	-1.84575	-2.13986	-2.50683	-2.55295	-1.3403
H	3.040179	1.637423	-2.07181	3.572159	1.517389	-1.89675	-2.82725	-1.9067	2.057531
H	1.434438	2.126732	-1.51565	2.238525	2.493291	-1.27061	-1.53819	-0.8588	2.671396
H	2.816236	2.32855	-0.45099	3.60904	2.092593	-0.22622	-3.15604	-0.19769	2.375425
H	4.586988	0.991145	0.095886	4.191281	0.0994	1.106525	-4.96188	-0.03457	-0.56261
H	-0.88656	-0.71963	2.204887	-0.77052	0.493351	2.555797	0.333621	3.006904	-0.34121
H	-0.15365	0.759561	2.844509	-0.27369	2.162897	2.24992	0.067644	2.928159	1.410808
H	-1.75821	0.816744	2.092711	-1.82973	1.573249	1.638698	1.648588	2.52341	0.733246
H	-3.11337	-1.68605	-0.41555	-3.08242	-1.71186	0.691155	2.637013	-1.58482	0.038238
H	-5.58533	-2.01245	0.530416	-4.67429	0.501681	-2.07008	5.082598	-2.58134	-0.34245
H	-6.79563	-1.0212	-0.33115	-6.20403	0.601001	-1.16569	6.375158	-1.51263	0.272006
H	-5.62767	-2.03354	-1.22612	-4.77123	1.510929	-0.62847	4.967976	-1.93604	1.287784
	3b-16			3b-17			3b-18		
C	1.33699	0.177098	0.695111	1.412594	0.625244	0.726125	1.412474	0.62493	0.726303
C	2.110939	0.312228	-0.62866	2.257079	0.417325	-0.56188	2.257036	0.417539	-0.56176
C	3.421686	-0.45266	-0.33382	3.146726	-0.81248	-0.28658	3.146893	-0.81217	-0.28676
C	2.936053	-1.68417	0.438469	2.275306	-1.65833	0.646306	2.275543	-1.65852	0.645747
C	1.631619	-1.27027	1.152471	1.665769	-0.6308	1.600535	1.665796	-0.63138	1.600287
C	-0.15706	0.566961	0.700604	-0.08304	0.931387	0.462192	-0.08321	0.930926	0.462522
C	-0.92292	-0.06716	-0.47364	-0.82673	-0.26482	-0.16257	-0.82666	-0.26509	-0.16292
C	-2.3969	0.186227	-0.42314	-2.26416	0.022773	-0.46143	-2.26406	0.022515	-0.46195
O	-0.29784	1.983569	0.503695	-0.19211	1.983493	-0.50882	-0.1925	1.983547	-0.50792
C	4.229951	-0.80643	-1.57751	3.584651	-1.54578	-1.54941	3.585071	-1.54491	-1.54983
H	1.820886	0.852474	1.414099	1.801186	1.5055	1.252819	1.800967	1.50503	1.253349
C	2.300465	1.735524	-1.14371	3.025841	1.657342	-1.0104	3.025554	1.657842	-1.00996
O	4.247117	0.289373	0.581481	4.302939	-0.34432	0.428581	4.302891	-0.34406	0.428765
C	-0.79113	0.19148	2.045046	-0.76889	1.374779	1.758797	-0.76914	1.373609	1.759323
C	-3.33313	-0.77205	-0.45729	-3.29761	-0.70696	-0.02155	-3.29761	-0.70675	-0.02151
C	-4.79014	-0.52158	-0.41353	-4.70243	-0.37004	-0.36813	-4.70238	-0.36982	-0.36825
C	-5.30382	0.896037	-0.31093	-5.76172	-1.28121	0.201467	-5.7618	-1.28074	0.201478
O	-5.56908	-1.47119	-0.45685	-4.99343	0.590864	-1.07384	-4.99326	0.590941	-1.07421
H	1.596609	-0.27377	-1.40143	1.589831	0.131842	-1.38137	1.589843	0.132155	-1.38133
H	3.713119	-2.02218	1.127479	2.849504	-2.4394	1.154858	2.849942	-2.43954	1.154121
H	2.747419	-2.49843	-0.26556	1.494565	-2.15373	0.05875	1.494927	-2.15389	0.058016
H	0.82018	-1.94498	0.865627	0.768342	-1.00652	2.097239	0.768399	-1.00742	2.096788
H	1.718323	-1.32979	2.239162	2.383838	-0.39053	2.387374	2.383787	-0.39126	2.387237
H	-0.72722	-1.14143	-0.51164	-0.74995	-1.13352	0.49453	-0.74996	-1.13398	0.493944
H	-0.53744	0.373611	-1.39939	-0.3363	-0.52274	-1.10788	-0.33599	-0.52264	-1.10817

H	-2.69193	1.231465	-0.35748	-2.47235	0.887666	-1.08783	-2.47223	0.887009	-1.0889
H	0.172709	2.431157	1.220041	0.194971	2.781797	-0.12467	0.195498	2.781383	-0.1237
H	4.608787	0.091671	-2.0759	4.174911	-0.88777	-2.19303	4.175085	-0.88651	-2.19326
H	5.087813	-1.42585	-1.30169	4.20124	-2.41409	-1.29493	4.20198	-2.41311	-1.2957
H	3.619739	-1.35924	-2.29664	2.719272	-1.90081	-2.11551	2.719799	-1.90007	-2.116
H	2.968477	1.760432	-2.01058	3.649739	1.4438	-1.88374	3.649972	1.444532	-1.88297
H	1.345139	2.169578	-1.44189	2.330286	2.453974	-1.28548	2.329857	2.454211	-1.28544
H	2.716902	2.386071	-0.36732	3.676215	2.025943	-0.21365	3.675412	2.026739	-0.21292
H	4.536285	1.096667	0.134812	4.802639	-1.12208	0.711158	4.80336	-1.12177	0.710065
H	-0.87441	-0.89119	2.166355	-0.79392	0.570906	2.498525	-0.79418	0.569351	2.498628
H	-0.18792	0.580531	2.871712	-0.23439	2.221782	2.200572	-0.2347	2.220412	2.201533
H	-1.79336	0.620677	2.120688	-1.79755	1.685004	1.559339	-1.79778	1.683907	1.559951
H	-3.05123	-1.82121	-0.51795	-3.13417	-1.5753	0.613291	-3.13427	-1.57476	0.613818
H	-4.97255	1.488446	-1.16718	-5.69222	-1.2935	1.292861	-5.69201	-1.29339	1.292846
H	-6.39253	0.884711	-0.28016	-6.75517	-0.95313	-0.10158	-6.75522	-0.95225	-0.10122
H	-4.92105	1.379123	0.591314	-5.59012	-2.30605	-0.13977	-5.59065	-2.30555	-0.14011
	3b-19								
C	-1.38896	0.920152	0.395523						
C	-2.0055	-0.49223	0.595695						
C	-3.11546	-0.62276	-0.47753						
C	-2.58074	0.228821	-1.62528						
C	-1.98956	1.456256	-0.92945						
C	0.154165	0.956759	0.453891						
C	0.797794	0.192355	-0.73541						
C	2.29505	0.201641	-0.68628						
O	0.500696	0.301698	1.678969						
C	-3.4136	-2.06544	-0.87078						
H	-1.71962	1.557664	1.222283						
C	-2.47708	-0.75902	2.022814						
O	-4.32752	0.022883	-0.05171						
C	0.646411	2.40804	0.505819						
C	3.034211	-0.81021	-0.2082						
C	4.512792	-0.8022	-0.11512						
C	5.285153	0.410917	-0.57695						
O	5.091896	-1.78369	0.341964						
H	-1.25387	-1.25061	0.355307						
H	-3.36499	0.470214	-2.34749						
H	-1.80041	-0.33271	-2.1495						
H	-1.2667	1.980428	-1.5589						
H	-2.78536	2.17016	-0.70732						
H	0.473807	0.650366	-1.67394						
H	0.441776	-0.83984	-0.72277						
H	2.78992	1.104415	-1.0379						

H	1.464716	0.316765	1.767115						
H	-3.77966	-2.64071	-0.01365						
H	-4.1799	-2.09259	-1.65018						
H	-2.51563	-2.56123	-1.24888						
H	-2.95475	-1.74073	2.113543						
H	-1.62722	-0.73623	2.707157						
H	-3.18965	0.002877	2.354886						
H	-4.68392	-0.47466	0.696325						
H	0.445195	2.940482	-0.42695						
H	0.149495	2.937788	1.322229						
H	1.724631	2.450689	0.687597						
H	2.559164	-1.72414	0.142405						
H	4.984036	1.295641	-0.01066						
H	6.350771	0.237085	-0.43432						
H	5.08955	0.613161	-1.6327						

Table S 17. Energy analysis for 2*R*, 3*S*, 6*S*, 7*S*-3

Conf.	Steric Energy (kJ/mol)	Relative Energy (kJ/mol)	Distribution (%)
3b-1	-773.148508	0.000499	7.68%
3b-2	-773.148894	0.000113	11.56%
3b-3	-773.146783	0.002224	1.24%
3b-4	-773.146771	0.002236	1.22%
3b-5	-773.147712	0.001295	3.31%
3b-6	-773.146837	0.00217	1.31%
3b-7	-773.146834	0.002173	1.31%
3b-8	-773.146743	0.002264	1.19%
3b-9	-773.147468	0.001539	2.56%
3b-10	-773.148542	0.000465	7.97%
3b-11	-773.147466	0.001541	2.55%
3b-12	-773.149007	0	13.03%
3b-13	-773.149007	0	13.03%
3b-14	-773.147844	0.001163	3.80%
3b-15	-773.147335	0.001672	2.22%
3b-16	-773.148893	0.000114	11.55%
3b-17	-773.148275	0.000732	6.00%
3b-18	-773.148282	0.000725	6.05%
3b-19	-773.147417	0.00159	2.42%

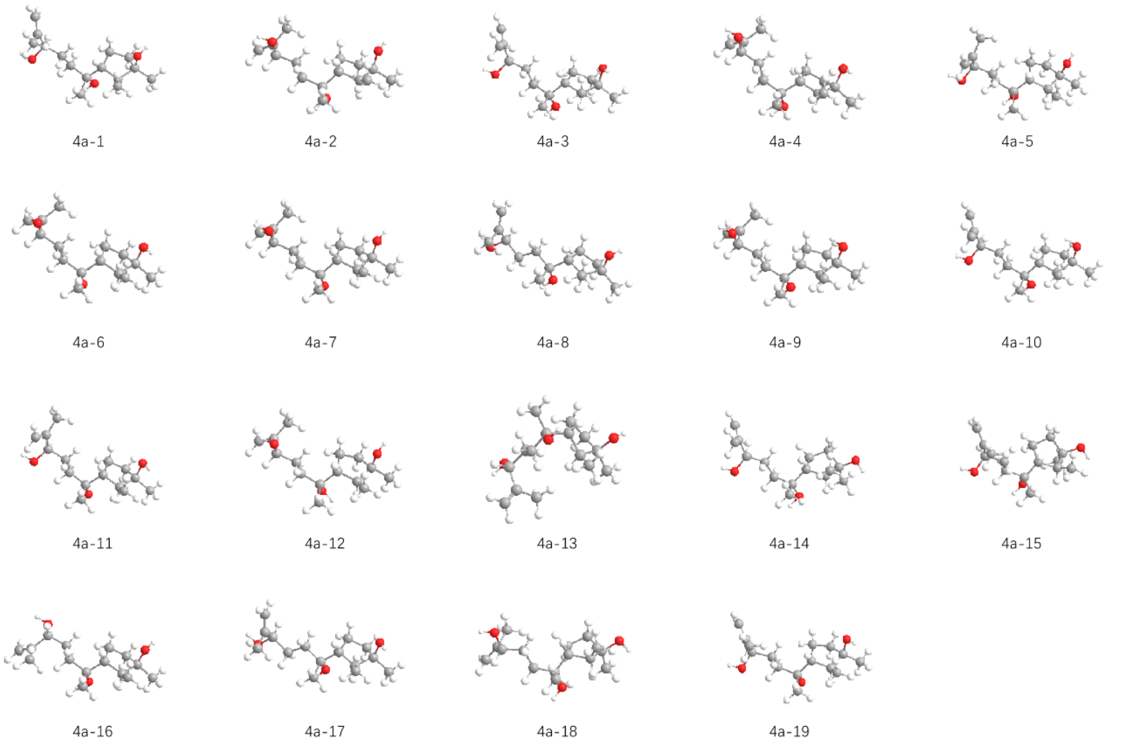

Figure S 83. Most stable conformers of 2*S*, 3*R*, 6*R*, 7*R*, 10*R*-4 calculated at MPW1PW91/6-31G+d, p level

Table S 18. Optimized Z-Matrixes of 2*S*, 3*R*, 6*R*, 7*R*, 10*R*-4 conformations in the Chloroform at MPW1PW91/6-31G+d, p level

	4a-1			4a-2			4a-3		
C	-1.09804	-0.00313	0.038075	0.986949	0.204776	-0.16385	-1.09433	0.002971	0.039489
C	-2.50778	0.169326	0.67366	2.503805	0.540786	-0.24911	-2.50987	0.172793	0.660645
C	-3.45026	-0.72013	-0.16146	3.237098	-0.80349	-0.06764	-3.44422	-0.73228	-0.18023
C	-2.84221	-0.62885	-1.56125	2.316122	-1.56047	0.889777	-2.82206	-0.65634	-1.5691
C	-1.33691	-0.7278	-1.31395	0.912047	-1.25073	0.370819	-1.31724	-0.73307	-1.30908
C	-0.30712	1.31308	-0.11	0.167687	1.206271	0.677032	-0.30724	1.321469	-0.10713
C	1.096232	1.08414	-0.70776	-1.33011	0.840874	0.714652	1.098091	1.096426	-0.70148
C	1.99609	0.101013	0.040381	-2.03495	0.744145	-0.63672	1.992076	0.101947	0.03865
C	3.413867	0.075918	-0.52374	-3.52345	0.439872	-0.49479	3.414229	0.086042	-0.51478
C	4.260405	-1.03856	0.062116	-3.80733	-0.86602	0.224812	4.254649	-1.04152	0.054548
O	-1.05376	2.123556	-1.03443	0.677601	1.108543	2.018177	-1.05444	2.130047	-1.03214
C	-4.91354	-0.29589	-0.09752	4.667179	-0.65964	0.442	-4.90759	-0.30521	-0.13931
C	4.832138	-1.94173	-0.7403	-4.61223	-0.88602	1.291567	4.830063	-1.92965	-0.76188
C	4.426393	-1.05765	1.558932	-3.15824	-2.10641	-0.33027	4.409916	-1.09141	1.551825
H	-0.5083	-0.64924	0.695466	0.577282	0.238942	-1.17754	-0.50683	-0.63627	0.705811
C	-2.56613	-0.12432	2.171603	2.914634	1.287945	-1.51643	-2.57631	-0.11298	2.160027
O	-3.31316	-2.06173	0.337437	3.242942	-1.45447	-1.34963	-3.33037	-2.10828	0.220211
C	-0.19216	2.064773	1.221503	0.346044	2.646178	0.182597	-0.19779	2.071992	1.225696
O	4.000161	1.353017	-0.24961	-4.05631	0.408357	-1.82252	3.999466	1.356634	-0.20997
H	-2.83776	1.19937	0.499547	2.769309	1.14658	0.624038	-2.84332	1.201233	0.483526
H	-3.22834	-1.40479	-2.23149	2.543883	-2.63154	0.924379	-3.19637	-1.45066	-2.22036

H	-3.08812	0.344303	-1.99754	2.439188	-1.15782	1.89993	-3.07611	0.308352	-2.01822
H	-0.77075	-0.29037	-2.1369	0.164288	-1.37963	1.154337	-0.75092	-0.29348	-2.13091
H	-1.03855	-1.77571	-1.22967	0.654392	-1.93137	-0.44474	-1.00685	-1.77664	-1.21755
H	0.983856	0.749146	-1.74341	-1.44083	-0.10196	1.257267	0.988778	0.773295	-1.74116
H	1.602084	2.055791	-0.75208	-1.84089	1.597288	1.326415	1.606644	2.067218	-0.73268
H	2.050888	0.359138	1.102737	-1.9449	1.683522	-1.19075	2.038733	0.343439	1.105423
H	1.590439	-0.91339	-0.02739	-1.58154	-0.03393	-1.25808	1.586759	-0.91111	-0.04856
H	3.355317	-0.07146	-1.6111	-3.9977	1.254908	0.069334	3.363907	-0.03914	-1.6053
H	-0.57978	2.958758	-1.14265	0.185854	1.732772	2.568194	-0.577	2.96228	-1.14807
H	-5.53014	-0.94864	-0.72481	5.127864	-1.64428	0.576889	-5.5069	-0.95961	-0.77804
H	-5.29065	-0.35925	0.926794	5.27706	-0.09719	-0.27007	-5.31117	-0.36312	0.877714
H	-5.03972	0.730174	-0.45329	4.689467	-0.14327	1.405414	-5.02439	0.724189	-0.48799
H	5.448383	-2.74589	-0.34633	-4.84283	-1.80988	1.815899	5.441695	-2.74307	-0.37993
H	4.69588	-1.90964	-1.81869	-5.06232	0.02503	1.67863	4.70126	-1.87568	-1.8403
H	4.708082	-0.06765	1.927845	-3.31215	-2.1732	-1.41085	4.693516	-0.11046	1.942885
H	3.486728	-1.32919	2.050322	-2.07616	-2.08741	-0.16066	3.465511	-1.3686	2.030896
H	5.188156	-1.7767	1.865998	-3.55683	-3.00835	0.138041	5.166208	-1.82011	1.849582
H	-3.58611	-0.02546	2.555686	3.995109	1.459839	-1.54253	-3.60419	-0.05651	2.535144
H	-1.93448	0.566201	2.734999	2.424909	2.262328	-1.57855	-1.98687	0.606613	2.731542
H	-2.22719	-1.14177	2.379598	2.644797	0.71493	-2.4062	-2.18044	-1.10751	2.389277
H	-3.77882	-2.64618	-0.27496	3.571463	-2.35315	-1.21598	-3.71023	-2.19444	1.104332
H	0.247523	1.442322	2.00489	0.103727	2.743951	-0.87861	0.239581	1.448853	2.010062
H	0.442598	2.949623	1.102842	-0.31058	3.323275	0.740135	0.436242	2.957766	1.110954
H	-1.17424	2.400378	1.560591	1.374709	2.980059	0.332548	-1.18162	2.406787	1.560713
H	4.881148	1.360188	-0.64601	-5.00196	0.221775	-1.75185	4.882788	1.372049	-0.60094
	4a-4			4a-5			4a-6		
C	0.97601	0.197297	-0.14965	1.087849	-0.01906	0.055792	0.974604	0.200171	-0.15673
C	2.490055	0.535429	-0.25859	2.497857	-0.21461	0.684136	2.487788	0.54019	-0.2546
C	3.23173	-0.81098	-0.0677	3.440506	0.701976	-0.12083	3.23351	-0.80559	-0.07704
C	2.325535	-1.55706	0.903626	2.83301	0.656196	-1.52301	2.325595	-1.56714	0.881388
C	0.912405	-1.25093	0.406346	1.327386	0.746501	-1.27355	0.91627	-1.25975	0.371819
C	0.164327	1.209568	0.686078	0.29326	-1.32818	-0.13229	0.162916	1.203711	0.701762
C	-1.33376	0.847726	0.736927	-1.11211	-1.07868	-0.7165	-1.33557	0.845088	0.750447
C	-2.05964	0.792407	-0.60567	-2.02104	-0.14932	0.08688	-2.06159	0.804433	-0.59259
C	-3.53745	0.44441	-0.4534	-3.43422	-0.0694	-0.49346	-3.53962	0.455685	-0.44254
C	-3.77699	-0.90377	0.201425	-4.25506	1.009235	0.193284	-3.77821	-0.90267	0.191573
O	0.68416	1.122839	2.023772	1.03498	-2.10765	-1.08675	0.657479	1.215122	2.052134
C	4.665163	-0.6528	0.427739	4.903908	0.276272	-0.07021	4.664182	-0.64977	0.426525
C	-4.56941	-1.0005	1.273295	-5.15367	0.713828	1.136781	-4.5568	-1.01484	1.272072
C	-3.09992	-2.09556	-0.42233	-3.98813	2.419317	-0.26416	-3.11497	-2.08673	-0.46137
H	0.553034	0.214818	-1.15841	0.501692	0.608258	0.734227	0.552339	0.229881	-1.16571
C	2.879846	1.266457	-1.54229	2.557556	0.027559	2.191214	2.882868	1.290854	-1.52501
O	3.221126	-1.58524	-1.27853	3.302941	2.026701	0.421132	3.228244	-1.56743	-1.29504

C	0.342075	2.644394	0.176521	0.182362	-2.12377	1.173606	0.342147	2.640639	0.215431
O	-4.09348	0.468172	-1.77193	-4.00531	-1.3663	-0.37293	-4.09837	0.500809	-1.75913
H	2.766985	1.158073	0.599088	2.826711	-1.23832	0.474193	2.757155	1.151378	0.614132
H	2.559295	-2.6245	0.931281	3.079582	-0.30209	-1.99073	2.561642	-2.63393	0.89719
H	2.465998	-1.14445	1.906918	3.219151	1.453743	-2.16733	2.467338	-1.17013	1.891807
H	0.179419	-1.36784	1.205602	0.762463	0.333425	-2.10985	0.161419	-1.4217	1.146742
H	0.639741	-1.94389	-0.39355	1.028172	1.791324	-1.15808	0.665389	-1.9383	-0.44638
H	-1.44054	-0.10905	1.255278	-1.00389	-0.68322	-1.73114	-1.45573	-0.12321	1.249767
H	-1.83204	1.589636	1.376169	-1.60787	-2.05113	-0.81714	-1.82104	1.578359	1.404742
H	-2.00298	1.757736	-1.11774	-2.11338	-0.48317	1.125785	-2.00467	1.777112	-1.09035
H	-1.59902	0.054688	-1.26947	-1.59762	0.858606	0.109343	-1.60254	0.075972	-1.26761
H	-4.02084	1.217193	0.160237	-3.35583	0.196565	-1.5598	-4.02	1.219087	0.184704
H	0.197048	1.75176	2.57252	0.558757	-2.93756	-1.22242	0.450174	0.361526	2.455338
H	5.120853	-1.63548	0.575668	5.520425	0.948597	-0.67658	5.12165	-1.63283	0.565679
H	5.276941	-0.10133	-0.29487	5.281007	0.307533	0.955592	5.277051	-0.08929	-0.28787
H	4.693847	-0.10892	1.375489	5.030505	-0.73802	-0.45815	4.688136	-0.11531	1.379733
H	-4.76924	-1.95621	1.751063	-5.73335	1.491906	1.625449	-4.75482	-1.9781	1.735317
H	-5.03932	-0.12325	1.711551	-5.32678	-0.31121	1.450994	-5.01734	-0.14361	1.731596
H	-3.25622	-2.10721	-1.5046	-2.93763	2.692785	-0.12401	-3.28812	-2.08095	-1.54104
H	-2.01808	-2.05918	-0.25402	-4.20408	2.525483	-1.33237	-2.03031	-2.05667	-0.31035
H	-3.47425	-3.03135	-0.00303	-4.59965	3.138582	0.283329	-3.48581	-3.02785	-0.05098
H	3.963464	1.415089	-1.60736	3.578182	-0.08263	2.57044	3.966281	1.443555	-1.58124
H	2.417344	2.253822	-1.59757	1.927805	-0.68275	2.731538	2.417927	2.277545	-1.56716
H	2.555178	0.708982	-2.42666	2.216982	1.036703	2.434082	2.564722	0.746152	-2.41961
H	3.785499	-1.13938	-1.92345	3.77231	2.630002	-0.16985	3.789156	-1.11143	-1.93587
H	0.101123	2.730889	-0.886	-0.24777	-1.52505	1.980334	0.116813	2.731046	-0.84938
H	-0.31609	3.326724	0.725878	-0.45939	-2.99985	1.029651	-0.32307	3.308677	0.769329
H	1.370311	2.98056	0.325128	1.164754	-2.47672	1.493898	1.367471	2.978185	0.380449
H	-5.03177	0.249374	-1.69569	-4.89886	-1.33112	-0.73911	-5.03877	0.291096	-1.68356
	4a-7			4a-8			4a-9		
C	0.980818	0.204817	-0.16566	-1.26727	0.136635	0.325119	0.988681	0.203174	-0.162
C	2.495599	0.544287	-0.25226	-2.6041	0.669163	-0.26254	2.506676	0.538445	-0.25078
C	3.236751	-0.79687	-0.0795	-3.69781	-0.2728	0.277952	3.245664	-0.81168	-0.06805
C	2.321735	-1.56366	0.877161	-2.97736	-1.61984	0.328778	2.322547	-1.56852	0.888393
C	0.917291	-1.25766	0.354681	-1.59415	-1.27772	0.881977	0.913914	-1.25196	0.379464
C	0.16496	1.203384	0.694619	-0.08457	0.124023	-0.669	0.168609	1.204558	0.67905
C	-1.33333	0.842843	0.737179	1.212579	-0.22099	0.096744	-1.32918	0.838624	0.715512
C	-2.04914	0.77874	-0.61036	2.403036	-0.60141	-0.78176	-2.03346	0.74333	-0.63625
C	-3.53264	0.452881	-0.46302	3.670869	-0.85347	0.028924	-3.52297	0.44278	-0.49623
C	-3.79324	-0.8845	0.206075	4.129568	0.353073	0.827021	-3.81129	-0.86066	0.225914
O	0.653956	1.210388	2.047341	-0.3928	-0.88961	-1.63801	0.679907	1.103404	2.018237
C	4.666823	-0.64856	0.428254	-4.97011	-0.28841	-0.56253	4.668166	-0.66204	0.446032
C	-4.57872	-0.95706	1.284972	4.338588	0.251018	2.143057	-4.61627	-0.87599	1.292606

C	-3.14325	-2.09448	-0.41183	4.342249	1.634239	0.064791	-3.16627	-2.10432	-0.32666
H	0.565657	0.24155	-1.17721	-0.96495	0.789369	1.151488	0.571715	0.238733	-1.17378
C	2.902328	1.303461	-1.51346	-2.88086	2.144569	0.019506	2.916311	1.285276	-1.51799
O	3.240607	-1.44316	-1.36275	-4.0038	0.163486	1.613412	3.36618	-1.52441	-1.31042
C	0.344235	2.642492	0.21499	0.081475	1.470248	-1.38284	0.348095	2.644102	0.184657
O	-4.08119	0.468675	-1.78443	4.67672	-1.24647	-0.9106	-4.05224	0.409336	-1.82525
H	2.758123	1.144396	0.626163	-2.58652	0.509253	-1.34676	2.769578	1.146526	0.621366
H	2.552946	-2.63363	0.905476	-3.51945	-2.35668	0.931552	2.553868	-2.63666	0.912541
H	2.452759	-1.16928	1.890693	-2.88802	-2.01802	-0.68679	2.443464	-1.16464	1.897924
H	0.154686	-1.42459	1.120749	-0.85714	-2.02693	0.589795	0.173765	-1.3733	1.170691
H	0.675359	-1.93088	-0.47078	-1.62209	-1.24947	1.973645	0.62431	-1.94199	-0.42173
H	-1.45559	-0.11783	1.250707	1.470133	0.642266	0.721017	-1.44089	-0.10443	1.257595
H	-1.82555	1.584616	1.376696	1.019298	-1.04831	0.786483	-1.83977	1.594575	1.327663
H	-1.97546	1.737757	-1.1319	2.184458	-1.52314	-1.33007	-1.94045	1.682018	-1.19086
H	-1.59402	0.027853	-1.26313	2.622303	0.170814	-1.52637	-1.58244	-0.03676	-1.25708
H	-4.00782	1.239161	0.139425	3.482011	-1.67906	0.728567	-3.99624	1.260431	0.064763
H	0.443484	0.356075	2.447267	0.238911	-0.81501	-2.36481	0.191489	1.727833	2.570971
H	5.132285	-1.63139	0.559879	-5.69584	-0.99558	-0.14653	5.12301	-1.64624	0.586222
H	5.272468	-0.08149	-0.2835	-5.43586	0.700518	-0.58006	5.28229	-0.10261	-0.26497
H	4.688933	-0.13419	1.392655	-4.75644	-0.59213	-1.59087	4.6761	-0.13538	1.403163
H	-4.79234	-1.90461	1.773062	4.680272	1.096846	2.734188	-4.84986	-1.79806	1.818687
H	-5.02972	-0.06756	1.718194	4.16748	-0.68283	2.67311	-5.06361	0.037194	1.677735
H	-3.3131	-2.1161	-1.49184	4.949704	1.457764	-0.82717	-3.31975	-2.17239	-1.40722
H	-2.05867	-2.07375	-0.25841	3.385365	2.046642	-0.27373	-2.0843	-2.08921	-0.15558
H	-3.52733	-3.01913	0.023153	4.834629	2.388436	0.681475	-3.56824	-3.00413	0.1427
H	3.982481	1.476518	-1.54045	-3.85113	2.450077	-0.38402	3.997855	1.447064	-1.54813
H	2.411756	2.27791	-1.56381	-2.12116	2.785388	-0.43325	2.433148	2.262877	-1.5787
H	2.630545	0.739162	-2.40815	-2.88983	2.336442	1.094759	2.642021	0.721503	-2.41379
H	3.60697	-2.32888	-1.24114	-4.57127	-0.50829	2.013672	2.481522	-1.6789	-1.66684
H	0.116636	2.738287	-0.84881	0.226119	2.280699	-0.66355	0.106069	2.742411	-0.87656
H	-0.31903	3.308116	0.774087	0.952006	1.45798	-2.0465	-0.30852	3.321015	0.742112
H	1.370329	2.97821	0.378423	-0.79242	1.698634	-1.99607	1.376776	2.977392	0.335241
H	-5.02525	0.275766	-1.70994	5.485655	-1.42501	-0.41281	-4.99903	0.227685	-1.75677
	4a-10			4a-11			4a-12		
C	-1.09896	0.001236	0.03738	1.08381	-0.02955	0.058691	0.991241	0.213076	-0.16199
C	-2.51165	0.168457	0.670189	2.502003	-0.22867	0.664862	2.511413	0.534967	-0.2459
C	-3.45406	-0.7322	-0.16786	3.430556	0.721588	-0.13113	3.232404	-0.81652	-0.05955
C	-2.8419	-0.64041	-1.56657	2.806051	0.713453	-1.52089	2.302594	-1.5613	0.89914
C	-1.33286	-0.71822	-1.32065	1.300936	0.768001	-1.2557	0.901411	-1.23751	0.380656
C	-0.30845	1.318507	-0.10766	0.293979	-1.33919	-0.14432	0.161572	1.221142	0.672517
C	1.095522	1.090806	-0.7045	-1.11403	-1.08731	-0.72078	-1.33283	0.857922	0.689229
C	1.992818	0.10129	0.038353	-2.01487	-0.15266	0.085452	-1.98852	0.666422	-0.6758
C	3.413628	0.083982	-0.51885	-3.43288	-0.07476	-0.48314	-3.49024	0.419535	-0.56623

C	4.257175	-1.0379	0.057177	-4.24365	1.015553	0.196977	-3.84078	-0.81017	0.251297
O	-1.05663	2.126427	-1.03137	1.035643	-2.10441	-1.10989	0.536242	1.153145	2.059679
C	-4.91266	-0.3092	-0.10887	4.89589	0.299666	-0.11469	4.664362	-0.68656	0.448615
C	4.828847	-1.93368	-0.75355	-5.13287	0.736041	1.154107	-4.67653	-0.71348	1.289664
C	4.42017	-1.07294	1.554077	-3.97821	2.418007	-0.28416	-3.21793	-2.11337	-0.17529
H	-0.50436	-0.64232	0.694678	0.501559	0.579422	0.757194	0.59562	0.244601	-1.18139
C	-2.57241	-0.12077	2.168173	2.574717	-0.02084	2.176871	2.929999	1.279156	-1.51242
O	-3.43558	-2.0938	0.29322	3.311141	2.075085	0.338272	3.229011	-1.46609	-1.34043
C	-0.19629	2.067229	1.225415	0.190102	-2.15012	1.152883	0.34829	2.659093	0.178252
O	3.99647	1.357823	-0.22509	-4.00719	-1.3681	-0.34127	-3.97548	0.287958	-1.9064
H	-2.84145	1.197831	0.492695	2.836649	-1.2457	0.432649	2.806022	1.144589	0.619005
H	-3.22307	-1.42437	-2.22641	3.174551	1.542585	-2.13077	2.519643	-2.6342	0.936335
H	-3.09053	0.331258	-2.00381	3.065491	-0.22494	-2.01979	2.431903	-1.16202	1.910739
H	-0.77602	-0.26529	-2.14092	0.737316	0.361376	-2.09614	0.155191	-1.34898	1.168347
H	-1.00044	-1.76097	-1.26174	0.983136	1.80441	-1.11787	0.632272	-1.92121	-0.42833
H	0.984733	0.763238	-1.74272	-1.01	-0.694	-1.73659	-1.45895	-0.04804	1.288678
H	1.602858	2.061857	-0.74146	-1.61326	-2.05847	-0.81665	-1.85503	1.649905	1.238414
H	2.042052	0.348442	1.103623	-2.09855	-0.48018	1.127228	-1.84824	1.551491	-1.30446
H	1.589512	-0.91325	-0.04243	-1.58986	0.854788	0.097717	-1.54059	-0.17582	-1.21173
H	3.360656	-0.04973	-1.60825	-3.36294	0.177576	-1.55332	-3.95785	1.293775	-0.09296
H	-0.58712	2.964338	-1.13831	0.557103	-2.93026	-1.26141	1.420071	1.531414	2.154604
H	-5.51941	-0.95818	-0.74605	5.490857	0.9884	-0.72052	5.114716	-1.67582	0.582614
H	-5.29779	-0.38029	0.912068	5.301536	0.307444	0.903126	5.279036	-0.13024	-0.26397
H	-5.02652	0.721399	-0.45302	5.016443	-0.71011	-0.51554	4.694546	-0.17154	1.412774
H	5.442856	-2.74302	-0.36681	-5.70545	1.522555	1.637626	-4.95234	-1.58126	1.88338
H	4.6956	-1.88955	-1.83189	-5.30562	-0.28405	1.484207	-5.1056	0.241192	1.58441
H	4.704958	-0.08793	1.933795	-2.92453	2.68903	-0.16462	-3.34146	-2.26754	-1.25079
H	3.478638	-1.34591	2.041206	-4.21028	2.509818	-1.35031	-2.14137	-2.11404	0.027545
H	5.17864	-1.79797	1.855087	-4.57835	3.147529	0.262346	-3.66108	-2.95885	0.354303
H	-3.59511	-0.03121	2.546211	3.604491	-0.09348	2.544013	4.01115	1.445505	-1.53565
H	-1.94816	0.574031	2.734206	1.990164	-0.77041	2.713562	2.44354	2.254858	-1.57724
H	-2.22763	-1.13505	2.387506	2.177431	0.959388	2.459184	2.658866	0.704424	-2.40027
H	-2.53127	-2.42863	0.232119	3.692898	2.118677	1.224617	3.542613	-2.37033	-1.20838
H	0.24358	1.444001	2.00812	-0.23529	-1.56049	1.968989	0.106807	2.75543	-0.88325
H	0.437367	2.952976	1.109015	-0.45262	-3.02432	1.0029	-0.29824	3.334105	0.745353
H	-1.17925	2.400812	1.563734	1.174344	-2.50755	1.462557	1.380902	2.995162	0.31303
H	4.878966	1.372674	-0.61798	-4.90322	-1.33486	-0.70151	-4.92908	0.138862	-1.85698
	4a-13			4a-14			4a-15		
C	1.478699	0.975601	-0.13768	-1.09237	-0.0188	0.187056	1.110707	0.004646	-0.20665
C	1.554601	-0.34328	0.663451	-2.54013	0.153538	0.734609	2.484667	0.732077	-0.13981
C	2.39704	-1.26629	-0.23339	-3.44785	-0.69918	-0.20972	3.543948	-0.38646	-0.07232
C	1.835941	-0.96383	-1.62588	-2.48437	-1.71488	-0.82366	2.821701	-1.48458	0.709039
C	1.571762	0.549806	-1.63244	-1.2094	-0.91373	-1.06303	1.409766	-1.48185	0.123705

C	0.325628	1.942043	0.230636	-0.32805	1.294916	-0.04793	0.027271	0.621801	0.70168
C	-1.05167	1.283288	0.395903	1.048863	1.045011	-0.69098	-1.32043	-0.11912	0.588357
C	-1.62018	0.544289	-0.81234	2.013857	0.167572	0.105547	-1.88228	-0.26427	-0.82527
C	-3.04108	0.042987	-0.56563	3.363935	0.019021	-0.5901	-3.32095	-0.78965	-0.86195
C	-3.14483	-0.95476	0.572676	4.260569	-1.00524	0.080094	-4.29956	0.122048	-0.14154
O	0.572463	2.485576	1.542529	-1.1168	2.055125	-0.97872	0.504498	0.472741	2.050056
C	2.323982	-2.74205	0.141745	-4.08978	0.18958	-1.2761	4.861256	0.051888	0.558525
C	-3.94569	-0.70985	1.613957	4.729187	-2.04595	-0.61548	-4.97343	-0.26636	0.944443
C	-2.32261	-2.21142	0.456787	4.59277	-0.77959	1.5317	-4.45022	1.499	-0.73453
H	2.399543	1.529721	0.087654	-0.52715	-0.57559	0.943397	0.737019	0.079492	-1.23244
C	2.055054	-0.21015	2.097429	-2.63949	-0.26226	2.201946	2.725826	1.729809	-1.27114
O	3.753509	-0.80274	-0.14125	-4.46821	-1.43276	0.471878	3.77682	-0.82731	-1.42113
C	0.271894	3.116068	-0.75472	-0.18628	2.092922	1.252803	-0.1742	2.115393	0.416085
O	-3.47774	-0.54635	-1.79438	3.981803	1.310173	-0.59202	-3.29739	-2.11488	-0.35035
H	0.557449	-0.79814	0.689183	-2.84216	1.201699	0.651668	2.546265	1.259099	0.818535
H	2.517651	-1.28545	-2.41946	-2.30561	-2.50592	-0.08694	3.328349	-2.45245	0.626205
H	0.902007	-1.5202	-1.76177	-2.88915	-2.18109	-1.72607	2.790348	-1.21156	1.768376
H	0.668919	0.779041	-2.19875	-1.30945	-0.29997	-1.96267	0.689046	-1.91653	0.816685
H	2.388304	1.086744	-2.11964	-0.33259	-1.5525	-1.19926	1.38307	-2.07658	-0.79266
H	-0.99936	0.606666	1.254832	0.885657	0.601023	-1.6786	-1.22089	-1.10992	1.037077
H	-1.74822	2.07774	0.688696	1.524163	2.018294	-0.8618	-2.0459	0.421931	1.209757
H	-1.66182	1.200834	-1.68693	2.181522	0.588984	1.101707	-1.85453	0.690859	-1.35846
H	-0.99548	-0.31044	-1.08786	1.596921	-0.83537	0.24288	-1.272	-0.96327	-1.40437
H	-3.67843	0.905651	-0.32793	3.189444	-0.30239	-1.62635	-3.62692	-0.81564	-1.91928
H	1.451282	2.888186	1.535764	-0.64816	2.881488	-1.15598	-0.18	0.808818	2.643689
H	2.879013	-3.34983	-0.58103	-4.64375	-0.41754	-1.99778	5.564364	-0.78697	0.600891
H	2.758511	-2.91254	1.129798	-4.78526	0.897549	-0.81048	5.324479	0.850298	-0.02749
H	1.288664	-3.0947	0.150108	-3.33659	0.778951	-1.80415	4.705808	0.412482	1.578875
H	-4.04176	-1.41232	2.437851	5.377002	-2.79096	-0.16061	-5.68127	0.397687	1.432849
H	-4.52444	0.208197	1.679784	4.473803	-2.18999	-1.66263	-4.8246	-1.2462	1.387652
H	-2.44432	-2.66555	-0.53031	4.924932	0.249196	1.695537	-3.54876	2.096308	-0.55978
H	-1.25659	-1.99111	0.578811	3.711109	-0.93752	2.160949	-4.59672	1.450717	-1.8184
H	-2.60138	-2.94216	1.218194	5.376065	-1.45983	1.871275	-5.2956	2.031513	-0.29528
H	1.373842	0.412377	2.679923	-3.64012	-0.09299	2.609091	3.716715	2.187743	-1.19226
H	3.045888	0.251321	2.120064	-1.93243	0.316065	2.804466	1.988415	2.535727	-1.25096
H	2.126213	-1.18817	2.583027	-2.40411	-1.32308	2.329425	2.661771	1.235047	-2.24298
H	4.275155	-1.29398	-0.78933	-5.13189	-0.80369	0.783495	4.319186	-1.62555	-1.37388
H	-0.01904	2.813006	-1.76233	0.304304	1.508014	2.035111	-0.4194	2.301578	-0.63304
H	-0.44837	3.856968	-0.39788	0.411226	2.995591	1.084798	-0.99371	2.509862	1.027271
H	1.253147	3.598053	-0.82574	-1.16712	2.400166	1.622676	0.726413	2.681229	0.662482
H	-4.38357	-0.85653	-1.66293	4.816251	1.233535	-1.07288	-4.17689	-2.49646	-0.46948
	4a-16			4a-17			4a-18		
C	-1.26703	0.148704	0.329808	1.021556	0.268131	-0.26673	-1.08345	-0.00785	0.059596

C	-2.60261	0.654683	-0.28187	2.548356	0.57734	-0.28859	-2.49966	0.173312	0.675796
C	-3.70137	-0.27834	0.284154	3.255343	-0.79409	-0.04308	-3.44013	-0.71954	-0.15874
C	-2.97496	-1.61227	0.401425	2.214628	-1.82367	-0.48251	-2.80882	-0.65998	-1.55111
C	-1.59361	-1.2423	0.942534	0.893606	-1.24345	0.011176	-1.31079	-0.77964	-1.26851
C	-0.08292	0.101313	-0.66135	0.196276	1.157015	0.678515	-0.30791	1.321419	-0.12403
C	1.214374	-0.21716	0.115604	-1.28079	0.721093	0.724086	1.098521	1.099071	-0.71674
C	2.400226	-0.63766	-0.7509	-2.02332	0.718117	-0.6111	1.989163	0.103524	0.026199
C	3.673084	-0.85278	0.062495	-3.52121	0.474869	-0.44482	3.417079	0.095027	-0.51254
C	4.136411	0.388406	0.80272	-3.85496	-0.83436	0.245358	4.254401	-1.03285	0.061151
O	-0.3914	-0.94412	-1.59515	0.749864	0.970283	1.991402	-1.02624	2.202089	-1.00607
C	-4.95723	-0.33773	-0.57884	3.618468	-0.95159	1.434243	-4.89823	-0.27512	-0.12587
C	4.353365	0.345741	2.120723	-4.60557	-0.84059	1.351044	4.84286	-1.91482	-0.7527
C	4.344503	1.633771	-0.01786	-3.31679	-2.09329	-0.3811	4.39205	-1.08931	1.559893
H	-0.96641	0.833909	1.130341	0.63935	0.454512	-1.27693	-0.49139	-0.62986	0.73805
C	-2.88318	2.137744	-0.04606	2.966329	1.242816	-1.59957	-2.57409	-0.09901	2.176694
O	-4.05418	0.091313	1.627563	4.424108	-0.99486	-0.84114	-3.32695	-2.05403	0.361549
C	0.084123	1.42307	-1.42002	0.319002	2.635413	0.293384	-0.2028	2.098078	1.187812
O	4.672892	-1.2872	-0.86514	-4.08234	0.509531	-1.76034	3.994503	1.365455	-0.19589
H	-2.57795	0.467216	-1.36146	2.801117	1.249764	0.536542	-2.82216	1.20289	0.484635
H	-3.52231	-2.3124	1.038162	2.223204	-1.87488	-1.57697	-3.19278	-1.4418	-2.21485
H	-2.8807	-2.05238	-0.59572	2.428448	-2.82423	-0.09712	-3.04213	0.307641	-2.00845
H	-0.85392	-2.00092	0.682551	0.772159	-1.42775	1.082248	-0.70511	-0.415	-2.10255
H	-1.62432	-1.17053	2.032084	0.032055	-1.68503	-0.49657	-1.04543	-1.83029	-1.13218
H	1.47703	0.669931	0.703277	-1.32446	-0.27469	1.176229	1.004298	0.765821	-1.75777
H	1.020275	-1.01499	0.83888	-1.8045	1.391623	1.419724	1.595266	2.072904	-0.75268
H	2.178427	-1.58355	-1.25489	-1.91032	1.678822	-1.12237	2.023937	0.342564	1.093884
H	2.615434	0.099242	-1.53172	-1.61604	-0.04557	-1.2815	1.589086	-0.91115	-0.06672
H	3.489011	-1.64619	0.799577	-3.94203	1.295263	0.152848	3.379336	-0.02608	-1.60423
H	0.245284	-0.89978	-2.32011	0.247426	1.521882	2.605596	-1.01077	1.817933	-1.8928
H	-5.67476	-1.04096	-0.1478	4.023209	-1.94993	1.623658	-5.51416	-0.92891	-0.75274
H	-5.44231	0.642473	-0.64506	4.373567	-0.21007	1.719816	-5.29157	-0.31716	0.893303
H	-4.7187	-0.66286	-1.59485	2.748932	-0.7882	2.074955	-5.00484	0.746945	-0.49893
H	4.698764	1.217229	2.671053	-4.86761	-1.76578	1.8579	5.453172	-2.72768	-0.36745
H	4.185407	-0.56322	2.693313	-4.97705	0.082717	1.789068	4.727513	-1.85548	-1.83241
H	4.945704	1.416594	-0.90503	-3.51066	-2.10477	-1.45706	4.666637	-0.109	1.958833
H	3.385512	2.031372	-0.36804	-2.23157	-2.15745	-0.24879	3.443258	-1.37269	2.026474
H	4.841671	2.414557	0.560691	-3.76204	-2.9837	0.066611	5.147896	-1.81617	1.863178
H	-3.86639	2.427989	-0.4325	4.025915	1.512539	-1.60285	-3.59699	0.011025	2.5496
H	-2.14511	2.770094	-0.5431	2.388172	2.158681	-1.75598	-1.94351	0.597569	2.733945
H	-2.8476	2.379288	1.021058	2.789319	0.581408	-2.45296	-2.24219	-1.11485	2.403068
H	-4.51894	0.937547	1.592817	5.118148	-0.41104	-0.5083	-3.8192	-2.63874	-0.22962
H	0.220433	2.258378	-0.72789	0.008065	2.813194	-0.73915	0.236604	1.48635	1.978802
H	0.959964	1.391194	-2.07592	-0.30814	3.253991	0.944853	0.421593	2.984291	1.047965

H	-0.78517	1.626681	-2.04845	1.351764	2.974544	0.399765	-1.18832	2.431046	1.519726
H	5.484117	-1.44706	-0.36476	-5.0351	0.371998	-1.67452	4.882325	1.385835	-0.57626
	4a-19								
C	-1.07943	0.001353	0.061731						
C	-2.50241	0.180386	0.660356						
C	-3.43089	-0.73557	-0.17553						
C	-2.78501	-0.69687	-1.55588						
C	-1.28726	-0.7878	-1.25955						
C	-0.30902	1.33297	-0.12567						
C	1.100478	1.113233	-0.71221						
C	1.983953	0.107539	0.025699						
C	3.416736	0.106398	-0.5001						
C	4.2455	-1.03702	0.05475						
O	-1.02803	2.206796	-1.01332						
C	-4.89057	-0.29478	-0.16892						
C	4.839354	-1.90106	-0.77425						
C	4.368018	-1.12989	1.552942						
H	-0.49009	-0.60969	0.752607						
C	-2.58711	-0.07585	2.163782						
O	-3.33538	-2.10355	0.253324						
C	-0.21179	2.114944	1.183737						
O	3.994641	1.367615	-0.14914						
H	-2.8303	1.206467	0.459533						
H	-3.15473	-1.50157	-2.19627						
H	-3.02924	0.258403	-2.03195						
H	-0.68201	-0.42122	-2.09316						
H	-1.00609	-1.83265	-1.11252						
H	1.011131	0.79024	-1.75676						
H	1.600168	2.085936	-0.73633						
H	2.009353	0.331637	1.097041						
H	1.5841	-0.90543	-0.08521						
H	3.388544	0.010428	-1.59451						
H	-1.01594	1.814699	-1.89665						
H	-5.48578	-0.95814	-0.80212						
H	-5.30993	-0.32615	0.842703						
H	-4.99243	0.727732	-0.54167						
H	5.443588	-2.72477	-0.40276						
H	4.734501	-1.81565	-1.85326						
H	4.645322	-0.16129	1.977784						
H	3.412682	-1.41761	2.003296						
H	5.115834	-1.8691	1.846177						
H	-3.61837	-0.00448	2.526759						
H	-1.99817	0.65187	2.725468						

H	-2.20047	-1.06813	2.417058						
H	-3.72658	-2.16885	1.134252						
H	0.226304	1.507256	1.978755						
H	0.410514	3.00253	1.043764						
H	-1.19969	2.447089	1.509517						
H	4.884649	1.395666	-0.52384						

Table S 19. Energy analysis for 2*S*, 3*R*, 6*R*, 7*R*, 10*R*-4 in the Chloroform at MPW1PW91/6-31G+d, p level

Conf.	Steric Energy	Relative Energy	Distribution
	(kJ/mol)	(kJ/mol)	(%)
4a-1	-813.598597	0.001109	4.79%
4a-2	-813.59922	0.000486	9.26%
4a-3	-813.598498	0.001208	4.31%
4a-4	-813.599042	0.000664	7.67%
4a-5	-813.598122	0.001584	2.90%
4a-6	-813.599706	0	15.49%
4a-7	-813.599199	0.000507	9.06%
4a-8	-813.597465	0.002241	1.44%
4a-9	-813.59855	0.001156	4.56%
4a-10	-813.598617	0.001089	4.89%
4a-11	-813.598069	0.001637	2.74%
4a-12	-813.59861	0.001096	4.86%
4a-13	-813.5991	0.000606	8.16%
4a-14	-813.597672	0.002034	1.80%
4a-15	-813.598028	0.001678	2.62%
4a-16	-813.598111	0.001595	2.86%
4a-17	-813.59847	0.001236	4.19%
4a-18	-813.598152	0.001554	2.99%
4a-19	-813.598714	0.000992	5.42%

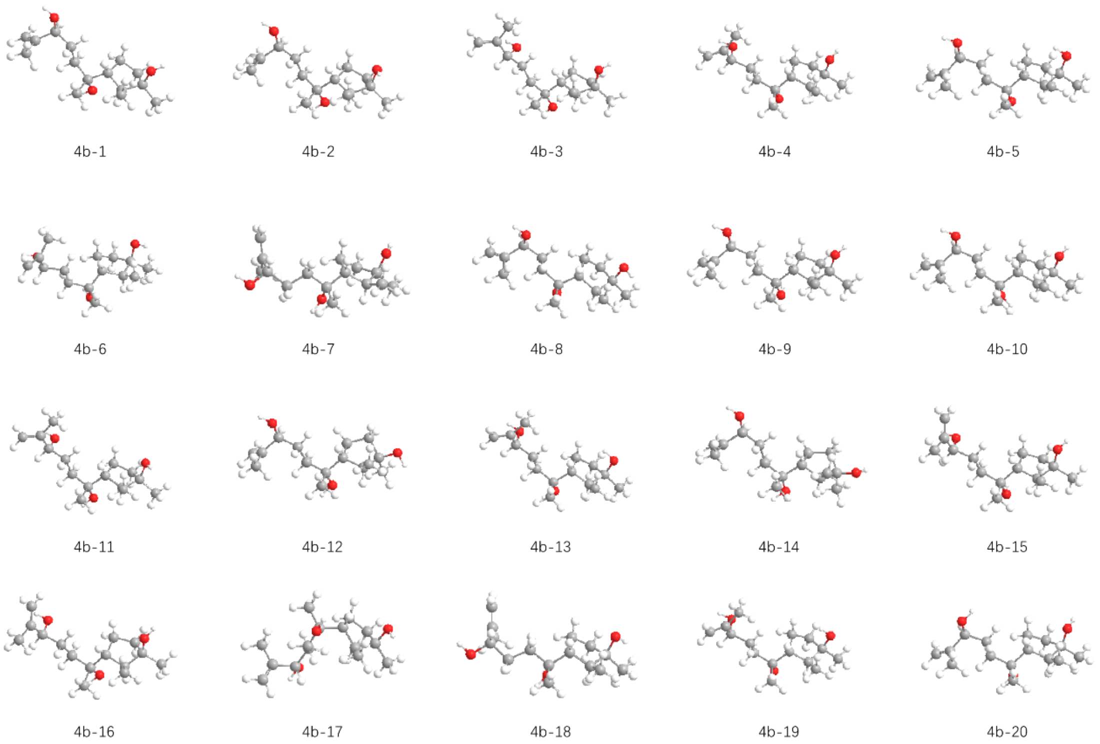

Figure S 84. Most stable conformers of 2*S*, 3*R*, 6*R*, 7*R*, 10*S*-4 calculated at MPW1PW91/6-31G+d, p level

Table S 20. Optimized Z-Matrixes of 2*S*, 3*R*, 6*R*, 7*R*, 10*S*-4 conformations in the Chloroform at MPW1PW91/6-31G+d, p level

	4b-1			4b-2			4b-3		
C	-1.12698	-0.10403	0.122564	-1.12628	-0.10677	0.12409	-1.07246	0.04884	0.090861
C	-2.4931	0.546571	0.485234	-2.49498	0.539834	0.481041	-2.49753	0.188895	0.699936
C	-3.56894	-0.40013	-0.08421	-3.57138	-0.40938	-0.10277	-3.40381	-0.71798	-0.15632
C	-2.89346	-0.95838	-1.33746	-2.88642	-0.9712	-1.34284	-2.77169	-0.60729	-1.54395
C	-1.45913	-1.23444	-0.88738	-1.45195	-1.23846	-0.88655	-1.26943	-0.67308	-1.26927
C	-0.07772	0.894668	-0.40709	-0.07738	0.896236	-0.39809	-0.30682	1.382753	-0.03438
C	1.265167	0.209728	-0.7345	1.267156	0.215423	-0.72655	1.109562	1.189299	-0.61402
C	1.882397	-0.62355	0.387356	1.886073	-0.61851	0.393805	2.034242	0.250703	0.160281
C	3.295869	-1.09423	0.05738	3.297607	-1.09243	0.059942	3.4316	0.185244	-0.44934
C	4.279432	0.040106	-0.16476	4.28189	0.039639	-0.17034	4.409879	-0.59993	0.404628
O	-0.61368	1.427358	-1.63041	-0.61201	1.435369	-1.61879	-1.05762	2.184062	-0.96324
C	-4.90462	0.282835	-0.35858	-4.89938	0.28133	-0.39412	-4.87724	-0.3271	-0.1182
C	5.001494	0.096467	-1.28806	4.99798	0.091968	-1.29764	5.543109	-0.02909	0.824891
C	4.401168	1.061797	0.934667	4.411093	1.064024	0.92573	4.047424	-2.02553	0.728266
H	-0.70752	-0.5431	1.032617	-0.70833	-0.54544	1.035251	-0.48381	-0.58909	0.756849
C	-2.67326	0.846728	1.972021	-2.68106	0.828954	1.969628	-2.57871	-0.11208	2.195365
O	-3.75245	-1.4546	0.875916	-3.79299	-1.53694	0.760327	-3.24588	-2.05813	0.340582
C	0.155337	2.050661	0.57306	0.151361	2.046629	0.58999	-0.22919	2.124905	1.305032
O	3.712583	-1.91039	1.156562	3.717309	-1.90507	1.160604	3.297642	-0.41078	-1.74353
H	-2.58785	1.479789	-0.0805	-2.58724	1.479709	-0.07394	-2.84636	1.212252	0.52332
H	-2.89978	-0.19381	-2.12042	-3.40141	-1.85994	-1.71695	-3.12756	-1.38887	-2.22438

H	-3.40922	-1.84323	-1.72647	-2.89342	-0.20927	-2.12771	-3.03165	0.361804	-1.98127
H	-0.77411	-1.26295	-1.73542	-0.76374	-1.26433	-1.73221	-0.69774	-0.21901	-2.07889
H	-1.40283	-2.20443	-0.38723	-1.39389	-2.20855	-0.38711	-0.94755	-1.71381	-1.18517
H	1.131247	-0.4204	-1.61887	1.135073	-0.41313	-1.61224	1.025775	0.837529	-1.64416
H	1.969765	0.997178	-1.03335	1.969818	1.005361	-1.02336	1.580703	2.180873	-0.66655
H	1.910207	-0.06597	1.32897	1.918176	-0.06022	1.33491	2.135704	0.575129	1.201158
H	1.28007	-1.51822	0.569848	1.282608	-1.51196	0.578469	1.618279	-0.76122	0.173411
H	3.258715	-1.70276	-0.85668	3.255763	-1.70429	-0.85161	3.816235	1.209048	-0.55831
H	0.028167	2.057129	-1.98458	0.033227	2.061696	-1.9729	-0.59417	3.025385	-1.069
H	-5.62089	-0.42968	-0.78181	-5.60243	-0.42958	-0.83653	-5.46719	-0.99139	-0.75881
H	-5.33247	0.681835	0.565118	-5.35064	0.677551	0.522468	-5.27166	-0.40262	0.898743
H	-4.78543	1.103464	-1.07115	-4.76315	1.114667	-1.08829	-5.02003	0.697144	-0.47302
H	5.724392	0.889236	-1.46321	5.721056	0.883221	-1.47881	6.264442	-0.57117	1.43107
H	4.888232	-0.64839	-2.07211	4.879458	-0.65481	-2.07906	5.78315	1.00298	0.58045
H	3.496423	1.676853	0.993266	3.507072	1.679768	0.988535	3.202504	-2.0634	1.423072
H	4.519982	0.572726	1.905415	4.535822	0.577325	1.896934	3.744284	-2.56093	-0.17559
H	5.251005	1.726748	0.769739	5.260223	1.728095	0.753755	4.88514	-2.55508	1.185955
H	-3.66173	1.270644	2.174354	-3.68274	1.218822	2.182249	-3.60718	-0.0318	2.560732
H	-1.92876	1.564906	2.323443	-1.96541	1.57332	2.324753	-1.96934	0.586424	2.773344
H	-2.57163	-0.0648	2.565087	-2.52869	-0.07477	2.568177	-2.22679	-1.12463	2.405587
H	-4.30444	-2.13018	0.460726	-4.22287	-1.21778	1.564424	-3.68071	-2.65108	-0.28612
H	0.455073	1.693052	1.561605	0.445691	1.68333	1.578159	0.211673	1.504407	2.089465
H	0.947737	2.709129	0.199924	0.945879	2.706994	0.224956	0.386881	3.025415	1.204108
H	-0.74959	2.650965	0.684961	-0.75383	2.647066	0.699828	-1.22332	2.436229	1.631958
H	4.60171	-2.23343	0.958704	4.603561	-2.2335	0.958791	4.173489	-0.42316	-2.15114
	4b-4			4b-5			4b-6		
C	-1.06166	0.045741	0.114406	-1.12865	-0.10382	0.118235	-1.05531	0.051007	0.123305
C	-2.49468	0.196435	0.699345	-2.49728	0.539287	0.489313	-2.49636	0.194101	0.687658
C	-3.39483	-0.72024	-0.15354	-3.57757	-0.40846	-0.09153	-3.38317	-0.73624	-0.17732
C	-2.73792	-0.64369	-1.53294	-2.89964	-0.95243	-1.35056	-2.70385	-0.67447	-1.54069
C	-1.243	-0.72461	-1.22187	-1.45731	-1.21877	-0.91316	-1.2121	-0.73266	-1.20822
C	-0.30799	1.390845	-0.04562	-0.07831	0.901965	-0.39691	-0.31103	1.40148	-0.03432
C	1.112161	1.200666	-0.61598	1.265169	0.220938	-0.73023	1.113097	1.221517	-0.59838
C	2.027643	0.252865	0.157847	1.882593	-0.62248	0.383866	2.014612	0.242535	0.152789
C	3.432244	0.198642	-0.43577	3.295175	-1.09255	0.048848	3.434676	0.224466	-0.40509
C	4.400768	-0.6018	0.414933	4.279999	0.04202	-0.16579	4.381008	-0.62711	0.420652
O	-1.02639	2.262803	-0.93638	-0.61508	1.446461	-1.61338	-1.03217	2.269183	-0.9263
C	-4.86476	-0.3154	-0.14937	-4.90686	0.277408	-0.3609	-4.85179	-0.32704	-0.20486
C	5.527717	-0.03784	0.860535	5.000048	0.105782	-1.28996	5.489197	-0.0894	0.939701
C	4.036089	-2.03374	0.706838	4.405229	1.054963	0.941319	4.017432	-2.07746	0.602586
H	-0.47483	-0.56847	0.804193	-0.70453	-0.5542	1.021799	-0.47087	-0.55226	0.824969
C	-2.5975	-0.07557	2.198722	-2.67794	0.824927	1.978465	-2.61285	-0.07536	2.186691
O	-3.25633	-2.05139	0.369642	-3.88306	-1.48381	0.812011	-3.26916	-2.10476	0.245649

C	-0.24397	2.160613	1.272655	0.151754	2.045744	0.597802	-0.25835	2.169478	1.285752
O	3.312563	-0.37446	-1.74222	3.710645	-1.91781	1.141616	3.351124	-0.26625	-1.747
H	-2.8361	1.218111	0.498551	-2.59052	1.478302	-0.06667	-2.84182	1.214207	0.485775
H	-3.0884	-1.43486	-2.20416	-3.41484	-1.836	-1.73664	-3.03889	-1.48376	-2.19427
H	-2.98764	0.317428	-1.99543	-2.90807	-0.17983	-2.12511	-2.95855	0.277452	-2.01815
H	-0.63156	-0.34031	-2.04228	-0.77871	-1.22044	-1.7662	-0.59463	-0.34739	-2.02377
H	-0.95068	-1.76794	-1.08607	-1.36739	-2.20669	-0.44732	-0.90957	-1.77135	-1.06101
H	1.047835	0.845778	-1.6493	1.133035	-0.40044	-1.62102	1.055876	0.900162	-1.64288
H	1.573704	2.193824	-0.66447	1.968913	1.012026	-1.02085	1.581343	2.212543	-0.61265
H	2.114316	0.56569	1.20361	1.912012	-0.073	1.330214	2.072635	0.504028	1.214661
H	1.614264	-0.76023	0.153742	1.280161	-1.51872	0.559029	1.606189	-0.77061	0.088175
H	3.817155	1.224239	-0.52243	3.256122	-1.69404	-0.86972	3.819875	1.253549	-0.41728
H	-0.98598	1.883425	-1.82445	0.024813	2.081474	-1.96162	-0.99088	1.888088	-1.81361
H	-5.44997	-0.98588	-0.78786	-5.61529	-0.43178	-0.79746	-5.41637	-0.99933	-0.85643
H	-5.27691	-0.36856	0.86181	-5.34028	0.663424	0.56574	-5.29554	-0.37339	0.795751
H	-4.99191	0.703458	-0.52484	-4.77415	1.109362	-1.05647	-4.96647	0.695212	-0.57457
H	6.241781	-0.59066	1.465621	5.723798	0.898747	-1.46043	6.187034	-0.67861	1.529182
H	5.769223	0.999095	0.63942	4.884384	-0.63301	-2.07937	5.730956	0.961082	0.796459
H	3.18483	-2.08575	1.392968	3.501209	1.670351	1.007276	3.146497	-2.18091	1.257489
H	3.741947	-2.55138	-0.21027	4.526466	0.55833	1.907922	3.754294	-2.53186	-0.35647
H	4.8698	-2.57186	1.16183	5.255052	1.720587	0.779284	4.840949	-2.6416	1.044415
H	-3.63034	0.016779	2.548378	-3.67173	1.233445	2.184302	-3.654	-0.02779	2.524448
H	-1.99238	0.632267	2.769741	-1.94148	1.547288	2.33763	-2.05316	0.659683	2.768436
H	-2.25317	-1.08526	2.43371	-2.56702	-0.08694	2.571664	-2.2124	-1.06151	2.442227
H	-3.71257	-2.65001	-0.23629	-3.07104	-1.97414	0.99595	-3.6859	-2.18549	1.113492
H	0.187238	1.550078	2.069186	0.449014	1.676638	1.582844	0.178132	1.562011	2.082005
H	0.368705	3.058214	1.15217	0.945159	2.708187	0.23437	0.344168	3.074239	1.168747
H	-1.24145	2.476725	1.584888	-0.75339	2.644768	0.713953	-1.25993	2.474414	1.596113
H	4.192375	-0.37714	-2.14146	4.599855	-2.23964	0.942072	4.241427	-0.2452	-2.12169
	4b-7			4b-8			4b-9		
C	1.256279	0.181376	-0.50917	-1.12473	-0.11216	0.12546	-1.12508	-0.10874	0.127542
C	2.669764	0.623531	-0.03804	-2.48652	0.550024	0.47432	-2.48604	0.556901	0.476571
C	3.484554	-0.67706	0.10449	-3.57391	-0.401	-0.08493	-3.57121	-0.39294	-0.06977
C	2.430045	-1.67684	0.577969	-2.90125	-0.98876	-1.32021	-2.90301	-0.9834	-1.3131
C	1.206796	-1.3563	-0.28079	-1.46862	-1.26275	-0.85939	-1.47195	-1.26271	-0.85206
C	0.085147	0.924275	0.174998	-0.07154	0.8807	-0.42712	-0.07179	0.879533	-0.43331
C	-1.23339	0.534284	-0.52668	1.271162	0.190108	-0.74163	1.269589	0.185441	-0.74612
C	-2.50567	1.096026	0.107336	1.888296	-0.61668	0.399657	1.885666	-0.61938	0.397113
C	-3.77018	0.444381	-0.44509	3.305243	-1.08761	0.084951	3.30346	-1.08926	0.084743
C	-3.82686	-1.05583	-0.21917	4.282275	0.046094	-0.16643	4.280219	0.045302	-0.16393
O	0.074563	0.470324	1.539024	-0.54731	1.497406	-1.63565	-0.54846	1.489215	-1.64538
C	4.681717	-0.5566	1.041309	-4.89944	0.292482	-0.37983	-4.90171	0.294268	-0.35715
C	-4.06605	-1.88196	-1.24222	5.010276	0.072227	-1.28689	5.011378	0.071981	-1.28234

C	-3.61739	-1.5424	1.190296	4.390573	1.102664	0.90093	4.384598	1.101995	0.903673
H	1.167862	0.389504	-1.58097	-0.70523	-0.54007	1.041169	-0.70524	-0.53275	1.044742
C	3.341392	1.664028	-0.93233	-2.66461	0.874252	1.956402	-2.66298	0.894594	1.955451
O	3.933582	-1.03579	-1.21349	-3.79505	-1.51373	0.796092	-3.75875	-1.42598	0.910995
C	0.266115	2.444421	0.151389	0.159549	2.049513	0.53002	0.162813	2.054363	0.515375
O	-4.87053	1.090108	0.204552	3.723956	-1.87086	1.207234	3.720764	-1.87329	1.206996
H	2.579171	1.028022	0.976618	-2.57225	1.478015	-0.10211	-2.573	1.476865	-0.1124
H	2.771045	-2.71387	0.484514	-3.4243	-1.8791	-1.67801	-3.42434	-1.87281	-1.68228
H	2.207338	-1.49146	1.633421	-2.91574	-0.23937	-2.11836	-2.91476	-0.23585	-2.11373
H	0.287927	-1.68334	0.207281	-0.77674	-1.34451	-1.70238	-0.78055	-1.35187	-1.69454
H	1.273392	-1.87541	-1.23967	-1.42833	-2.22007	-0.33573	-1.435	-2.21687	-0.3222
H	-1.17827	0.857223	-1.57299	1.147442	-0.47201	-1.60719	1.144579	-0.47896	-1.60987
H	-1.2994	-0.55676	-0.54613	1.964068	0.973881	-1.06639	1.963992	0.966853	-1.07341
H	-2.49894	0.950514	1.193026	1.911884	-0.03574	1.32706	1.907171	-0.03746	1.323893
H	-2.59275	2.171923	-0.06917	1.290233	-1.50968	0.603542	1.287919	-1.51251	0.60138
H	-3.82237	0.633547	-1.52608	3.274131	-1.72164	-0.81192	3.274569	-1.72254	-0.81281
H	-0.46477	1.078971	2.059078	-0.60193	0.816666	-2.31959	-0.60012	0.80511	-2.3261
H	5.199748	-1.51785	1.127172	-5.60906	-0.4212	-0.80683	-5.62386	-0.42044	-0.76653
H	5.397305	0.17885	0.664073	-5.34251	0.705012	0.53326	-5.3254	0.714146	0.558935
H	4.365841	-0.25453	2.04351	-4.76295	1.114736	-1.08702	-4.7769	1.100439	-1.08496
H	-4.12986	-2.95828	-1.10407	5.728244	0.864453	-1.48315	5.72932	0.864737	-1.47655
H	-4.19923	-1.50671	-2.25408	4.906083	-0.69715	-2.04819	4.90992	-0.69747	-2.04396
H	-2.57592	-1.39602	1.497638	3.482931	1.715303	0.932058	3.476792	1.714529	0.931422
H	-4.24223	-0.98084	1.890548	4.503968	0.645582	1.887822	4.494394	0.645061	1.89103
H	-3.85239	-2.60424	1.284521	5.238589	1.766076	0.721284	5.233214	1.76544	0.727015
H	4.342132	1.914163	-0.56682	-3.66255	1.276258	2.163296	-3.64908	1.328355	2.147696
H	2.764091	2.590476	-0.96797	-1.94243	1.62163	2.290784	-1.91481	1.617908	2.287352
H	3.442115	1.287502	-1.95277	-2.51625	-0.01634	2.575299	-2.56528	-0.00225	2.571106
H	4.30918	-1.92424	-1.1593	-4.21417	-1.18012	1.600012	-4.35015	-2.08685	0.527557
H	0.329	2.816392	-0.87473	0.432669	1.700537	1.528408	0.440889	1.711826	1.514554
H	-0.58047	2.944016	0.632975	0.962856	2.687936	0.152322	0.96381	2.69081	0.129447
H	1.168748	2.742516	0.688066	-0.73956	2.663181	0.614834	-0.73645	2.667509	0.601254
H	-5.68283	0.690768	-0.13424	4.617524	-2.18945	1.022506	4.616171	-2.18827	1.024987
	4b-10			4b-11			4b-12		
C	-1.12251	-0.09524	0.087468	-1.06897	0.049541	0.095177	-1.14479	-0.07179	0.281321
C	-2.48474	0.534765	0.499037	-2.49907	0.184139	0.691603	-2.56404	0.512089	0.547029
C	-3.56452	-0.4088	-0.07002	-3.40035	-0.72509	-0.18041	-3.5505	-0.4228	-0.22427
C	-2.91849	-0.91078	-1.36172	-2.75209	-0.61888	-1.5553	-2.77544	-1.73275	-0.36736
C	-1.46634	-1.17992	-0.96716	-1.25182	-0.67367	-1.26613	-1.34566	-1.28432	-0.65003
C	-0.06509	0.917047	-0.41875	-0.3071	1.386353	-0.01969	-0.11117	0.947128	-0.2266
C	1.27109	0.233214	-0.75391	1.110848	1.201056	-0.59768	1.231762	0.275255	-0.5687
C	1.865533	-0.63741	0.350881	2.031151	0.247358	0.163197	1.932859	-0.44635	0.580389
C	3.283728	-1.0993	0.029401	3.433499	0.198647	-0.43662	3.264556	-1.06019	0.157529

C	4.272923	0.038932	-0.14087	4.404468	-0.6112	0.402558	4.267397	-0.0436	-0.35607
O	-0.47488	1.487918	-1.67367	-1.0587	2.192251	-0.94341	-0.65054	1.493415	-1.44144
C	-4.92008	0.25797	-0.27827	-4.8715	-0.324	-0.16281	-3.90151	0.179805	-1.5856
C	5.021393	0.122168	-1.24493	5.531695	-0.05175	0.853234	4.817141	-0.19056	-1.56535
C	4.369846	1.032525	0.986327	4.04207	-2.04724	0.676501	4.607547	1.10183	0.560358
H	-0.70244	-0.57387	0.977111	-0.48254	-0.58662	0.765111	-0.76628	-0.44625	1.239188
C	-2.63224	0.798435	1.996308	-2.58835	-0.12553	2.185037	-2.8634	0.60018	2.043289
O	-3.69547	-1.49545	0.859971	-3.27025	-2.10436	0.2036	-4.75486	-0.71149	0.489559
C	0.170029	2.050405	0.585483	-0.23483	2.117778	1.32616	0.091048	2.081877	0.783382
O	3.681438	-1.95	1.109926	3.30944	-0.3597	-1.7482	3.777946	-1.73571	1.309776
H	-2.62387	1.488093	-0.02906	-2.84833	1.208796	0.523546	-2.63498	1.516998	0.120668
H	-3.43368	-1.78768	-1.76831	-3.09961	-1.41021	-2.22486	-2.82628	-2.26759	0.587634
H	-2.96345	-0.12014	-2.1184	-3.01365	0.347705	-1.99607	-3.19496	-2.38323	-1.13962
H	-0.80861	-1.14538	-1.83623	-0.67562	-0.21637	-2.0709	-1.24213	-0.98237	-1.69606
H	-1.37472	-2.17357	-0.52242	-0.92545	-1.71262	-1.18003	-0.6137	-2.07485	-0.46418
H	1.139417	-0.37116	-1.65645	1.029637	0.866645	-1.63374	1.062477	-0.42647	-1.39207
H	1.976429	1.025466	-1.02791	1.583694	2.192614	-0.63205	1.897848	1.052649	-0.96666
H	1.880076	-0.11057	1.310609	2.123604	0.548159	1.212067	2.111108	0.230031	1.422124
H	1.261152	-1.53755	0.496317	1.616859	-0.76526	0.149393	1.309858	-1.2624	0.958714
H	3.261398	-1.67944	-0.90336	3.81911	1.225087	-0.51273	3.074827	-1.79105	-0.64057
H	-1.22027	2.079682	-1.50834	-0.59248	3.031991	-1.04997	-0.01712	2.139088	-1.78175
H	-5.63568	-0.45383	-0.70307	-5.44663	-0.98005	-0.82176	-4.50977	-0.51867	-2.16741
H	-5.3255	0.616918	0.671435	-5.29385	-0.40233	0.84522	-4.46877	1.108436	-1.45346
H	-4.84072	1.1042	-0.96632	-4.99945	0.707771	-0.5004	-3.00107	0.431209	-2.15075
H	5.748602	0.918228	-1.38326	6.247532	-0.61144	1.449863	5.547612	0.516488	-1.95028
H	4.924443	-0.60145	-2.05073	5.772142	0.987956	0.64415	4.548522	-1.0222	-2.21228
H	3.465429	1.648654	1.037037	3.192024	-2.10929	1.363304	3.756181	1.783738	0.661654
H	4.463567	0.519194	1.94728	3.746637	-2.5531	-0.24668	4.846578	0.736816	1.562896
H	5.224961	1.698949	0.858859	4.877296	-2.59023	1.122854	5.45619	1.675741	0.183523
H	-3.61901	1.208377	2.231593	-3.62388	-0.09282	2.541586	-3.83711	1.056828	2.24059
H	-1.88489	1.51305	2.348007	-2.02255	0.595567	2.778265	-2.10275	1.207776	2.542969
H	-2.50918	-0.12764	2.56147	-2.17915	-1.11631	2.406854	-2.8613	-0.39107	2.506121
H	-4.24153	-2.1729	0.440034	-3.66939	-2.21028	1.077006	-5.29292	0.090737	0.506848
H	0.465067	1.668268	1.566082	0.204063	1.491273	2.107131	0.391557	1.703097	1.763521
H	0.958352	2.71334	0.218388	0.38004	3.020062	1.23527	0.869814	2.769841	0.435323
H	-0.73252	2.652964	0.725334	-1.23064	2.42564	1.65149	-0.83178	2.652862	0.907806
H	4.575235	-2.26402	0.918773	4.188598	-0.36301	-2.14886	4.613168	-2.15074	1.05644
	4b-13			4b-14			4b-15		
C	-1.07845	0.046976	0.07685	-1.14624	-0.07344	0.289438	-1.06804	0.058414	0.086382
C	-2.49739	0.174996	0.704783	-2.56677	0.507049	0.560501	-2.48996	0.433062	0.591859
C	-3.41963	-0.71661	-0.1647	-3.54476	-0.40632	-0.22964	-3.40892	-0.72622	0.156319
C	-2.80285	-0.57155	-1.55661	-2.77697	-1.7272	-0.37163	-2.77697	-1.17549	-1.16245
C	-1.29351	-0.62871	-1.30665	-1.34226	-1.28582	-0.64419	-1.2767	-1.1488	-0.86808

C	-0.30388	1.378917	-0.01765	-0.11502	0.949218	-0.21573	-0.30635	1.238487	-0.56905
C	1.109835	1.189012	-0.60448	1.229667	0.281128	-0.55891	1.102986	0.829952	-1.04314
C	2.048889	0.27028	0.176458	1.934589	-0.43831	0.589185	2.016356	0.215177	0.016597
C	3.427043	0.169809	-0.47152	3.261235	-1.05937	0.161	3.426369	-0.05177	-0.51274
C	4.426141	-0.58461	0.386033	4.26365	-0.04977	-0.36689	4.360015	-0.50447	0.597232
O	-1.05337	2.201715	-0.92711	-0.65429	1.497245	-1.42963	-1.03045	1.727766	-1.71194
C	-4.88584	-0.32156	-0.09987	-3.86906	0.206909	-1.59366	-4.87689	-0.33328	0.031638
C	5.573869	-0.0036	0.748861	4.803844	-0.20695	-1.57918	4.683333	-1.79004	0.763355
C	4.066338	-1.99305	0.78036	4.614453	1.100557	0.539278	4.89211	0.589891	1.485087
H	-0.48243	-0.61247	0.716558	-0.76571	-0.44855	1.24601	-0.4741	-0.26502	0.946684
C	-2.56414	-0.15818	2.193295	-2.86968	0.570633	2.05794	-2.56986	0.742022	2.085459
O	-3.37799	-2.09072	0.256022	-4.7579	-0.56988	0.51155	-3.26569	-1.76707	1.136602
C	-0.21949	2.088932	1.338139	0.083895	2.080997	0.798151	-0.21645	2.441529	0.368592
O	3.25014	-0.48033	-1.73401	3.780837	-1.72907	1.313903	3.298407	-1.00558	-1.55974
H	-2.8424	1.20371	0.554126	-2.63861	1.51892	0.152938	-2.82946	1.309052	0.027726
H	-3.16666	-1.34174	-2.24201	-2.83603	-2.26315	0.582313	-3.14128	-2.15657	-1.48531
H	-3.06787	0.40817	-1.96532	-3.19137	-2.37602	-1.15018	-3.03256	-0.45333	-1.94553
H	-0.74287	-0.13657	-2.10789	-1.22951	-0.98487	-1.68941	-0.68052	-1.09864	-1.78288
H	-0.93889	-1.66525	-1.28365	-0.61398	-2.07825	-0.45169	-0.9833	-2.07101	-0.36229
H	1.023264	0.823003	-1.62949	1.061639	-0.42105	-1.38218	1.01911	0.124337	-1.8753
H	1.571855	2.183825	-0.67226	1.892871	1.060425	-0.95787	1.577117	1.728667	-1.4546
H	2.183486	0.631968	1.201006	2.120456	0.241167	1.426772	2.087677	0.876735	0.88492
H	1.627161	-0.73756	0.23912	1.310706	-1.25026	0.974873	1.616517	-0.74031	0.371044
H	3.813609	1.185389	-0.63594	3.063269	-1.79493	-0.6308	3.826319	0.888886	-0.92389
H	-0.59444	3.048273	-1.00787	-0.02671	2.152421	-1.76251	-0.99655	1.049779	-2.39993
H	-5.47815	-0.96106	-0.75981	-4.47908	-0.47817	-2.19386	-5.47667	-1.1862	-0.30372
H	-5.27436	-0.43074	0.916426	-4.42695	1.137173	-1.45431	-5.27148	-0.00267	0.996074
H	-5.01644	0.717065	-0.41262	-2.95944	0.440348	-2.15143	-5.0059	0.474663	-0.6935
H	6.309851	-0.52414	1.356282	5.533774	0.494896	-1.9745	5.349741	-2.10397	1.561819
H	5.811875	1.015685	0.454087	4.527543	-1.04213	-2.21829	4.284237	-2.56218	0.11238
H	3.241914	-1.99626	1.5005	3.765807	1.785453	0.643121	5.455754	1.322958	0.898678
H	3.735118	-2.56483	-0.09079	4.860535	0.741258	1.542166	4.08036	1.134096	1.977966
H	4.914332	-2.50765	1.236068	5.461611	1.669605	0.151898	5.549079	0.19063	2.25969
H	-3.59062	-0.09385	2.566259	-3.85217	1.005969	2.251191	-3.59679	0.967467	2.389083
H	-1.95404	0.528447	2.784165	-2.11612	1.182901	2.563206	-1.9534	1.606118	2.343262
H	-2.20695	-1.17371	2.38503	-2.84932	-0.42594	2.509684	-2.22548	-0.11025	2.675475
H	-2.47076	-2.41234	0.171235	-5.34541	-1.12296	-0.01978	-3.74037	-2.54217	0.80876
H	0.211281	1.444953	2.10905	0.385253	1.699254	1.776852	0.221073	2.164949	1.330515
H	0.409519	2.982479	1.258437	0.860973	2.772287	0.452801	0.401098	3.22261	-0.08325
H	-1.21008	2.406345	1.669756	-0.84051	2.648846	0.924796	-1.20672	2.863303	0.552127
H	4.111962	-0.5128	-2.1695	4.613784	-2.14692	1.057716	4.182161	-1.18366	-1.90778
	4b-16			4b-17			4b-18		
C	-1.07152	0.057366	0.082622	1.441674	0.892169	-0.38788	1.253806	0.168651	-0.49417

C	-2.49417	0.418815	0.599743	1.942634	-0.06506	0.713631	2.670685	0.615103	-0.04159
C	-3.40905	-0.73299	0.136975	2.99043	-0.93232	-0.01896	3.490525	-0.69028	0.103741
C	-2.77576	-1.14964	-1.19096	2.308657	-1.20965	-1.35711	2.444887	-1.68542	0.59007
C	-1.27476	-1.12263	-0.90459	1.538408	0.07549	-1.70773	1.203479	-1.36568	-0.24372
C	-0.30913	1.246545	-0.53821	0.100017	1.616908	-0.12368	0.088738	0.924898	0.184895
C	1.098777	0.845463	-1.024	-1.01683	0.73538	0.451645	-1.23377	0.524257	-0.50342
C	2.016546	0.21272	0.021302	-1.43716	-0.47997	-0.37036	-2.50379	1.084364	0.135794
C	3.425268	-0.04591	-0.51578	-2.60901	-1.22495	0.263168	-3.7708	0.454577	-0.43611
C	4.359835	-0.52049	0.584371	-3.86423	-0.38357	0.399172	-3.84253	-1.04899	-0.23998
O	-1.07331	1.653298	-1.68694	0.295782	2.59655	0.915672	0.086565	0.49602	1.556434
C	-4.87841	-0.34145	0.022066	3.387646	-2.19881	0.730879	4.692069	-0.55974	1.033574
C	4.678769	-1.80984	0.728094	-4.46561	-0.25767	1.585926	-4.07732	-1.85316	-1.28134
C	4.897462	0.556705	1.489692	-4.38568	0.271366	-0.85252	-3.6526	-1.56431	1.162047
H	-0.47757	-0.28298	0.936165	2.189389	1.693102	-0.46469	1.157194	0.360082	-1.56856
C	-2.57135	0.688777	2.101246	2.444362	0.613528	1.984584	3.329914	1.646042	-0.95641
O	-3.2646	-1.79492	1.095708	4.16319	-0.1618	-0.3275	3.920726	-1.17491	-1.17917
C	-0.21351	2.429516	0.432816	-0.36877	2.358976	-1.38098	0.273659	2.443971	0.132491
O	3.295378	-0.97914	-1.58038	-2.85486	-2.36376	-0.56815	-4.86923	1.097816	0.218988
H	-2.83809	1.307937	0.060256	1.132492	-0.75108	0.990367	2.593067	1.035279	0.967844
H	-3.13882	-2.12417	-1.53602	3.040625	-1.496	-2.11588	2.791828	-2.71645	0.481784
H	-3.02493	-0.40815	-1.95644	1.617831	-2.04914	-1.23142	2.244591	-1.49795	1.649119
H	-0.69572	-1.01538	-1.82197	0.561233	-0.1687	-2.12531	0.294379	-1.67911	0.271343
H	-0.96602	-2.05761	-0.43082	2.064209	0.656379	-2.46824	1.243099	-1.8998	-1.19559
H	0.999563	0.164953	-1.87167	-0.70838	0.408519	1.450706	-1.18769	0.840808	-1.55221
H	1.582735	1.751629	-1.41453	-1.88233	1.388827	0.608949	-1.29562	-0.56725	-0.51562
H	2.091451	0.856518	0.902677	-1.71629	-0.19517	-1.38979	-2.50244	0.917328	1.218395
H	1.615908	-0.74892	0.3576	-0.61599	-1.19736	-0.45777	-2.58204	2.164071	-0.02072
H	3.826306	0.902137	-0.90916	-2.30886	-1.56231	1.264928	-3.81436	0.665186	-1.51353
H	-0.60364	2.384447	-2.10974	0.99312	3.199594	0.625102	-0.45507	1.110413	2.067379
H	-5.4757	-1.18934	-0.33015	4.057811	-2.80387	0.114439	5.20109	-1.52302	1.124639
H	-5.27356	-0.03172	0.993366	3.910105	-1.96177	1.663598	5.414737	0.170007	0.651818
H	-5.01013	0.480468	-0.68679	2.508601	-2.7989	0.981152	4.382011	-0.23434	2.030034
H	5.345558	-2.13972	1.519789	-5.37358	0.327685	1.706518	-4.15099	-2.93139	-1.16487
H	4.275186	-2.56947	0.065268	-4.06285	-0.73073	2.478258	-4.19649	-1.45735	-2.28709
H	5.463685	1.29709	0.915017	-3.71953	1.080275	-1.17182	-2.61202	-1.43897	1.481085
H	4.088352	1.095864	1.992433	-4.43524	-0.448	-1.67458	-4.27544	-1.0067	1.867193
H	5.553514	0.142001	2.256925	-5.37928	0.695447	-0.69628	-3.90322	-2.62424	1.234593
H	-3.59761	0.907038	2.412694	1.629088	1.133681	2.489481	4.35347	1.870833	-0.63712
H	-1.95336	1.545098	2.381191	3.209625	1.364166	1.757606	2.781451	2.589818	-0.96109
H	-2.2268	-0.17907	2.668083	2.87003	-0.11273	2.684729	3.362785	1.288795	-1.99066
H	-3.70813	-2.5732	0.733704	4.596373	0.067355	0.5053	4.588156	-0.56559	-1.52056
H	0.241324	2.140058	1.383792	-0.65296	1.683395	-2.19021	0.338514	2.796459	-0.90045
H	0.397032	3.228922	-0.0017	-1.23252	2.982322	-1.13439	-0.57108	2.955711	0.604251

H	-1.20375	2.840746	0.63837	0.428186	3.008976	-1.75886	1.176897	2.748536	0.664782
H	4.179416	-1.15694	-1.92783	-3.59306	-2.85275	-0.18085	-5.68297	0.711673	-0.13148
	4b-19			4b-20					
C	-1.06136	0.049461	0.119733	1.132834	0.105387	0.122547			
C	-2.50103	0.199178	0.691723	2.503264	-0.52624	0.501879			
C	-3.39641	-0.73477	-0.16167	3.570463	0.398588	-0.11687			
C	-2.72331	-0.67661	-1.53499	2.886456	0.890465	-1.39288			
C	-1.22744	-0.73168	-1.21439	1.452192	1.180433	-0.95088			
C	-0.31014	1.396327	-0.04529	0.076059	-0.91824	-0.34046			
C	1.11111	1.207138	-0.61274	-1.27245	-0.25176	-0.68216			
C	2.026126	0.260306	0.162802	-1.90336	0.595348	0.421395			
C	3.429162	0.201064	-0.43431	-3.30241	1.104974	0.054571			
C	4.397703	-0.60081	0.414953	-4.2898	-0.01101	-0.21416			
O	-1.03121	2.261131	-0.93875	0.594633	-1.50815	-1.54447			
C	-4.85965	-0.32434	-0.18273	4.910266	-0.28683	-0.36284			
C	5.526849	-0.03915	0.857677	-4.89826	-0.10596	-1.39917			
C	4.030851	-2.03201	0.708065	-4.52348	-0.99577	0.900673			
H	-0.47279	-0.55311	0.819904	0.727032	0.592374	1.014435			
C	-2.616	-0.05586	2.192728	2.698354	-0.76187	1.998391			
O	-3.38899	-2.08271	0.335818	3.74798	1.499823	0.790608			
C	-0.25087	2.169192	1.271368	-0.14164	-2.02603	0.697281			
O	3.303273	-0.37419	-1.73908	-3.84306	1.892694	1.119648			
H	-2.84071	1.218446	0.477491	2.595415	-1.48248	-0.0244			
H	-3.06449	-1.4848	-2.18671	3.39518	1.75798	-1.82746			
H	-2.97188	0.276225	-2.01412	2.893746	0.088393	-2.13746			
H	-0.61983	-0.34089	-2.03368	0.764321	1.155387	-1.79677			
H	-0.89925	-1.7688	-1.08881	1.390489	2.176872	-0.50634			
H	1.050008	0.852305	-1.6463	-1.14578	0.36174	-1.57929			
H	1.571642	2.200634	-0.66039	-1.97001	-1.05099	-0.96397			
H	2.115356	0.576211	1.207397	-1.96902	0.040271	1.362539			
H	1.611443	-0.75242	0.162159	-1.27343	1.471261	0.616612			
H	3.816531	1.225394	-0.52391	-3.22289	1.72092	-0.85154			
H	-0.97105	1.893036	-1.83042	-0.05246	-2.15277	-1.86042			
H	-5.43136	-0.99987	-0.82477	5.618724	0.408715	-0.82557			
H	-5.28921	-0.36617	0.821796	5.345824	-0.63508	0.577611			
H	-4.96682	0.693397	-0.56509	4.794309	-1.14339	-1.0323			
H	6.241161	-0.59335	1.461154	-5.60221	-0.9059	-1.61414			
H	5.770237	0.997064	0.635415	-4.71191	0.61313	-2.19265			
H	3.18203	-2.08218	1.39746	-3.62663	-1.59814	1.083414			
H	3.732835	-2.54908	-0.20814	-4.75791	-0.47507	1.832569			
H	4.864824	-2.572	1.160265	-5.34338	-1.67597	0.662681			
H	-3.6549	0.018321	2.527071	3.688826	-1.1772	2.208309			
H	-2.03103	0.66795	2.764262	1.958094	-1.46407	2.388522			

H	-2.25643	-1.05529	2.453057	2.603671	0.174597	2.552536			
H	-2.47565	-2.39527	0.375959	4.302022	2.154392	0.345571			
H	0.17607	1.560493	2.071639	-0.41389	-1.62131	1.675441			
H	0.363362	3.065527	1.150834	-0.94832	-2.69374	0.374613			
H	-1.24931	2.487754	1.577735	0.761656	-2.62789	0.814036			
H	4.18107	-0.37795	-2.14275	-3.18876	2.564159	1.353155			

Table S 21. Energy analysis for 2*S*, 3*R*, 6*R*, 7*R*, 10*S*-4 in the Chloroform at MPW1PW91/6-31G+d, p level

Conf.	Steric Energy	Relative Energy	Distribution
	(kJ/mol)	(kJ/mol)	(%)
4b-1	-813.5989	0.000216	8.24%
4b-2	-813.598911	0.000205	8.33%
4b-3	-813.597793	0.001323	2.55%
4b-4	-813.598993	0.000123	9.09%
4b-5	-813.598302	0.000814	4.37%
4b-6	-813.598688	0.000428	6.58%
4b-7	-813.598224	0.000892	4.03%
4b-8	-813.598481	0.000635	5.29%
4b-9	-813.598171	0.000945	3.81%
4b-10	-813.598587	0.000529	5.91%
4b-11	-813.598173	0.000943	3.82%
4b-12	-813.598122	0.000994	3.61%
4b-13	-813.599116	0	10.35%
4b-14	-813.597439	0.001677	1.75%
4b-15	-813.597662	0.001454	2.22%
4b-16	-813.597876	0.00124	2.79%
4b-17	-813.597071	0.002045	1.19%
4b-18	-813.598869	0.000247	7.97%
4b-19	-813.598096	0.00102	3.52%
4b-20	-813.598346	0.00077	4.58%

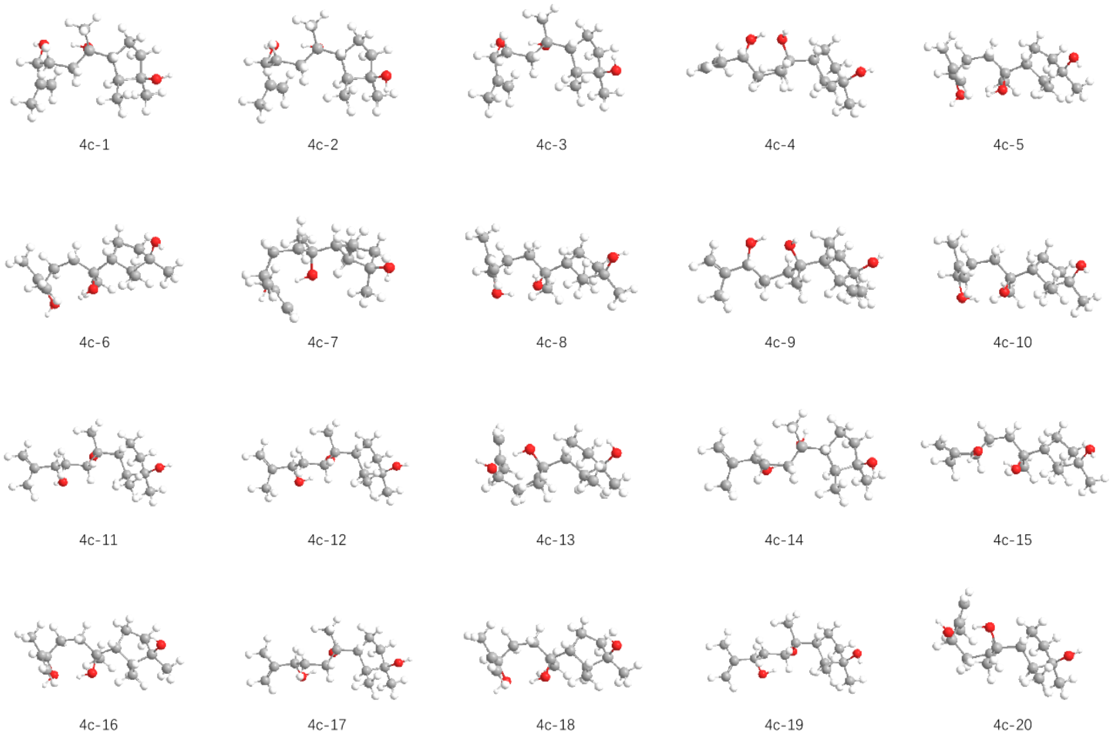

Figure S 85. Most stable conformers of 2*S*, 3*R*, 6*R*, 7*S*, 10*R*-4 calculated at MPW1PW91/6-31G+d, p level

Table S 22. Optimized Z-Matrixes of 2*S*, 3*R*, 6*R*, 7*S*, 10*R*-4 conformations in the Chloroform at MPW1PW91/6-31G+d, p level

	4c-1			4c-2			4c-3		
C	1.355076	0.900366	0.541457	1.354225	0.904468	0.543396	1.354816	0.900072	0.534797
C	1.709382	-0.61277	0.448493	1.709443	-0.60697	0.441734	1.710996	-0.61395	0.447749
C	3.049803	-0.6829	-0.30998	3.05464	-0.67388	-0.32286	3.05789	-0.68695	-0.31467
C	2.944021	0.492052	-1.2808	2.956807	0.510863	-1.27448	2.947608	0.484311	-1.2905
C	2.328873	1.609743	-0.43982	2.332353	1.620387	-0.42868	2.321582	1.606109	-0.45922
C	-0.12898	1.232407	0.245741	-0.12991	1.235058	0.246924	-0.13055	1.232528	0.243791
C	-1.05163	0.365352	1.133531	-1.05109	0.365165	1.134026	-1.05059	0.366643	1.134929
C	-2.55785	0.671022	1.168875	-2.55776	0.668373	1.170297	-2.55684	0.671877	1.170271
C	-3.4008	0.239369	-0.03661	-3.39986	0.23631	-0.0357	-3.39934	0.238285	-0.03489
C	-3.24029	-1.23005	-0.37978	-3.23793	-1.23293	-0.37918	-3.23802	-1.23144	-0.37647
O	-0.32497	0.949503	-1.14037	-0.32437	0.95239	-1.13908	-0.32713	0.947506	-1.14069
C	3.302046	-2.02241	-0.99434	3.292797	-2.00668	-1.02521	3.305887	-2.02484	-0.99195
C	-2.69966	-1.65017	-1.5262	-2.69384	-1.65224	-1.52426	-2.69606	-1.6525	-1.5219
C	-3.73187	-2.18677	0.675477	-3.73266	-2.19042	0.673937	-3.73069	-2.18731	0.679063
H	1.555159	1.246177	1.561861	1.550184	1.244928	1.566589	1.555393	1.254348	1.553427
C	1.740246	-1.351	1.786068	1.742209	-1.34766	1.77833	1.742811	-1.34676	1.787509
O	4.090217	-0.42664	0.649886	4.161802	-0.38301	0.547273	4.177429	-0.49486	0.567565
C	-0.39508	2.721553	0.513148	-0.39902	2.723329	0.515478	-0.39432	2.72232	0.509871
O	-3.09802	1.114121	-1.12483	-3.09722	1.111579	-1.12337	-3.09633	1.112119	-1.12387
H	0.973593	-1.09137	-0.20778	0.970603	-1.08658	-0.21025	0.972501	-1.09278	-0.20506
H	3.913239	0.755698	-1.71896	3.933699	0.772441	-1.69021	3.918648	0.742852	-1.72134

H	2.267648	0.22284	-2.09793	2.287541	0.248199	-2.09894	2.268514	0.211141	-2.10361
H	1.828545	2.344494	-1.07101	1.833508	2.357971	-1.05796	1.811585	2.326952	-1.09786
H	3.108623	2.130386	0.121553	3.108133	2.139684	0.139193	3.092098	2.162834	0.086115
H	-0.68889	0.462309	2.162561	-0.68786	0.460652	2.163189	-0.68693	0.465776	2.163485
H	-0.91667	-0.68284	0.85124	-0.91538	-0.68228	0.849087	-0.91543	-0.68188	0.854249
H	-2.7445	1.734689	1.339397	-2.74592	1.731619	1.341585	-2.74394	1.73564	1.339634
H	-2.97423	0.1572	2.040633	-2.97332	0.153255	2.041685	-2.97259	0.158748	2.042666
H	-4.4527	0.395738	0.241661	-4.45199	0.391714	0.242229	-4.45127	0.394806	0.242928
H	-1.27433	1.063691	-1.32741	-1.27351	1.066571	-1.32719	-1.27688	1.060377	-1.32776
H	4.251504	-2.00152	-1.54052	4.23582	-1.97546	-1.57777	4.249185	-1.99814	-1.54427
H	3.354545	-2.83063	-0.2598	3.349559	-2.83192	-0.3065	3.367229	-2.8309	-0.25558
H	2.507546	-2.25169	-1.70963	2.484673	-2.22838	-1.72719	2.4992	-2.25371	-1.69234
H	-2.61302	-2.71031	-1.74622	-2.6066	-2.71224	-1.74481	-2.60886	-2.71281	-1.74072
H	-2.30601	-0.96245	-2.26772	-2.29854	-0.96399	-2.26439	-2.30232	-0.96568	-2.26418
H	-3.08291	-2.15977	1.556962	-3.08807	-2.16216	1.558619	-3.08377	-2.1586	1.562001
H	-4.74018	-1.92371	1.010999	-4.74301	-1.92916	1.004653	-4.74006	-1.92503	1.011953
H	-3.74857	-3.21237	0.303248	-3.74584	-3.21611	0.301753	-3.74538	-3.21337	0.308064
H	2.06228	-2.38908	1.657328	2.097292	-2.37751	1.658256	2.076491	-2.3812	1.662482
H	0.754267	-1.37018	2.255059	0.751642	-1.40421	2.233217	0.756642	-1.37307	2.255441
H	2.436105	-0.86938	2.476823	2.396999	-0.84005	2.494026	2.430942	-0.86156	2.48535
H	4.913542	-0.31078	0.157882	4.241545	-1.10794	1.180739	4.083631	0.364014	1.000394
H	0.334181	3.351002	0.000044	0.328526	3.354544	0.002283	0.334966	3.350798	-0.00456
H	-1.38629	3.00633	0.150328	-1.39104	3.006012	0.153273	-1.38528	3.007355	0.147002
H	-0.34647	2.94371	1.584011	-0.35048	2.945031	1.586449	-0.34542	2.945772	1.580434
H	-3.66366	0.881126	-1.87373	-3.66226	0.878603	-1.87273	-3.66184	0.878756	-1.87278
	4c-4			4c-5			4c-6		
C	-1.54224	0.491463	0.762304	1.137329	-0.05066	-0.53455	1.137335	-0.05065	-0.53455
C	-2.4767	0.580663	-0.47898	2.214989	0.705606	0.289286	2.215009	0.705609	0.289272
C	-3.40461	-0.65237	-0.39652	3.570475	0.064941	-0.09749	3.570486	0.064923	-0.09751
C	-2.53433	-1.69159	0.317677	3.187836	-1.38168	-0.3954	3.187827	-1.38169	-0.39539
C	-1.83428	-0.88381	1.409591	1.871899	-1.26165	-1.16637	1.871887	-1.26166	-1.16635
C	-0.0395	0.734851	0.468507	-0.12341	-0.41959	0.284316	-0.1234	-0.41956	0.28433
C	0.582544	-0.38003	-0.38614	-1.23141	-0.83707	-0.71084	-1.23141	-0.83706	-0.71081
C	2.01517	-0.17749	-0.89908	-2.53028	-1.45316	-0.16839	-2.53027	-1.45315	-0.16834
C	3.095586	0.066397	0.172735	-3.54406	-0.51547	0.497364	-3.54406	-0.51545	0.497389
C	4.479	-0.09714	-0.42273	-3.9539	0.646567	-0.38822	-3.95393	0.646536	-0.38824
O	0.663787	0.679552	1.72979	-0.49037	0.779748	0.970877	-0.49035	0.779794	0.970872
C	-3.92445	-1.11398	-1.75381	4.641429	0.20781	0.978972	4.641447	0.207792	0.978953
C	5.174585	0.970608	-0.82458	-3.68733	1.918342	-0.08114	-3.68733	1.918325	-0.08124
C	4.997456	-1.50359	-0.56177	-4.68492	0.259855	-1.64788	-4.68505	0.25976	-1.64783
H	-1.8313	1.283587	1.464834	0.788884	0.614657	-1.33208	0.78889	0.614659	-1.33209
C	-3.22379	1.906346	-0.61031	2.161444	2.221505	0.120015	2.161484	2.221508	0.119983
O	-4.50759	-0.28634	0.446995	4.062281	0.594263	-1.33993	4.062294	0.594223	-1.33995

C	0.170596	2.115715	-0.15698	0.148257	-1.51946	1.319786	0.148275	-1.51941	1.319817
O	2.967567	-0.8267	1.273313	-3.01069	-0.10776	1.758484	-3.01067	-0.10769	1.758482
H	-1.87942	0.444333	-1.38754	2.061628	0.492055	1.352137	2.06165	0.492073	1.352127
H	-3.12575	-2.52918	0.702458	3.975196	-1.90018	-0.94904	3.975178	-1.90021	-0.94904
H	-1.80554	-2.10289	-0.38906	3.032503	-1.91098	0.550629	3.032495	-1.91099	0.550643
H	-0.93534	-1.36755	1.795908	1.293858	-2.18837	-1.13471	1.293835	-2.18837	-1.13468
H	-2.51329	-0.75071	2.254897	2.08308	-1.05644	-2.21814	2.083064	-1.05647	-2.21813
H	-0.0501	-0.53542	-1.26449	-0.802	-1.57329	-1.39766	-0.802	-1.57328	-1.39763
H	0.543555	-1.31137	0.186001	-1.47032	0.045054	-1.31568	-1.47033	0.045053	-1.31566
H	2.055418	0.652924	-1.61152	-2.3155	-2.26185	0.535951	-2.31547	-2.26182	0.536022
H	2.274855	-1.07851	-1.46529	-3.04763	-1.92634	-1.00852	-3.04761	-1.92636	-1.00846
H	2.999241	1.095677	0.537728	-4.44711	-1.11275	0.687843	-4.44709	-1.11275	0.687911
H	0.346493	1.393828	2.29858	-1.34488	0.606192	1.406241	-1.34486	0.606255	1.406239
H	-4.56504	-1.99511	-1.64135	5.563319	-0.28676	0.661041	5.563331	-0.28679	0.661023
H	-4.51429	-0.32573	-2.22933	4.870575	1.261763	1.171064	4.870604	1.261745	1.171033
H	-3.09921	-1.38203	-2.41932	4.31259	-0.23981	1.920843	4.312609	-0.23982	1.92083
H	6.147833	0.870741	-1.29818	-4.00908	2.728045	-0.7297	-4.00912	2.727995	-0.72983
H	4.791265	1.980167	-0.69539	-3.1244	2.195384	0.804459	-3.12434	2.195409	0.804306
H	5.099989	-1.96587	0.422981	-4.00855	-0.23332	-2.35362	-4.00875	-0.23355	-2.35355
H	4.30485	-2.12932	-1.13317	-5.49656	-0.44356	-1.43579	-5.49674	-0.44357	-1.43562
H	5.968054	-1.51869	-1.0617	-5.10754	1.134641	-2.14473	-5.10763	1.134531	-2.14474
H	-3.90219	1.894969	-1.46852	2.961156	2.72156	0.678581	2.961206	2.721559	0.678539
H	-2.5238	2.73371	-0.75183	1.206557	2.598669	0.489322	1.206604	2.598689	0.489292
H	-3.8169	2.108517	0.283962	2.244018	2.508214	-0.93385	2.244055	2.508203	-0.93389
H	-5.02526	-1.08522	0.612507	4.255892	1.531521	-1.20785	4.255916	1.531481	-1.20789
H	-0.33695	2.887467	0.430873	1.01335	-1.26543	1.937247	1.013356	-1.26536	1.937283
H	1.232806	2.365721	-0.19485	-0.70873	-1.6266	1.989797	-0.70872	-1.62656	1.989819
H	-0.22239	2.155529	-1.17621	0.33131	-2.49131	0.852598	0.33135	-2.49126	0.852642
H	2.181952	-0.51786	1.756656	-3.67149	0.433849	2.211336	-3.67146	0.433949	2.211317
	4c-7			4c-8			4c-9		
C	1.336501	0.889481	0.624015	-1.10335	-0.06881	0.543025	1.533422	0.060629	-0.90232
C	1.664811	-0.63151	0.539772	-2.14415	0.69967	-0.31916	2.457892	0.742813	0.147457
C	2.949496	-0.73639	-0.34162	-3.51736	0.110209	0.061499	3.416069	-0.36266	0.644634
C	3.605179	0.635076	-0.18913	-3.18052	-1.34731	0.387085	2.574159	-1.63526	0.508106
C	2.43007	1.607263	-0.1967	-1.88204	-1.24605	1.187911	1.861211	-1.45069	-0.83079
C	-0.10394	1.261986	0.210702	0.163884	-0.50048	-0.23859	0.02376	0.378749	-0.75039
C	-1.09535	0.533457	1.148992	1.244308	-0.94165	0.769114	-0.57979	-0.22127	0.528833
C	-2.59691	0.844347	1.045279	2.573353	-1.49819	0.237437	-2.01689	0.171827	0.897165
C	-3.36699	0.249607	-0.13977	3.531726	-0.52358	-0.47809	-3.09819	-0.14369	-0.15735
C	-3.17616	-1.24924	-0.28002	3.819037	0.720547	0.35031	-4.48386	0.00182	0.433153
O	-0.24878	0.84014	-1.14502	0.641534	0.634324	-0.99664	-0.66878	-0.27546	-1.83719
C	2.57407	-1.02791	-1.79508	-4.57526	0.266919	-1.02507	3.943168	-0.12172	2.055111
C	-2.54172	-1.80424	-1.31573	3.625759	1.955324	-0.12283	-5.34695	-1.01628	0.473723

C	-3.7468	-2.06957	0.847679	4.370831	0.472512	1.731471	-4.82409	1.369755	0.968889
H	1.450712	1.198385	1.670449	-0.75658	0.601697	1.339285	1.809056	0.437179	-1.8957
C	1.837975	-1.25081	1.926711	-2.06529	2.218906	-0.19178	3.172568	1.993563	-0.36011
O	3.887738	-1.71699	0.110681	-3.94314	0.783358	1.256904	4.512362	-0.40979	-0.28187
C	-0.29942	2.781672	0.291569	-0.11569	-1.57784	-1.28375	-0.22162	1.88665	-0.84318
O	-3.007	0.982507	-1.3122	3.131176	-0.24085	-1.80489	-2.93269	-1.44052	-0.70254
H	0.851918	-1.15367	0.025934	-1.9838	0.448617	-1.37235	1.858851	1.032027	1.018302
H	4.11929	0.663553	0.778147	-3.98937	-1.84867	0.928914	3.187245	-2.54137	0.557498
H	4.346106	0.832599	-0.96887	-3.01736	-1.8988	-0.54528	1.851697	-1.68357	1.329956
H	2.084568	1.777611	-1.22016	-1.32748	-2.187	1.191398	0.976679	-2.08071	-0.9399
H	2.692941	2.579596	0.22675	-2.1094	-1.00749	2.228987	2.542165	-1.71311	-1.6436
H	-0.79519	0.773269	2.175143	0.805087	-1.711	1.411473	0.052255	0.063152	1.374679
H	-0.95381	-0.54519	1.031137	1.446212	-0.08862	1.430332	-0.52066	-1.31074	0.457169
H	-2.78254	1.921954	1.051934	2.396594	-2.33786	-0.44077	-2.05887	1.237923	1.141236
H	-3.07261	0.459429	1.952428	3.108659	-1.91901	1.094921	-2.27161	-0.36774	1.816505
H	-4.43409	0.426606	0.05529	4.485469	-1.0547	-0.58195	-3.01635	0.595107	-0.96831
H	-1.18658	0.948257	-1.38755	0.900986	1.32512	-0.36872	-0.36013	0.094911	-2.67506
H	3.461612	-0.99784	-2.43387	-5.52087	-0.18652	-0.7092	4.609504	-0.93533	2.360566
H	2.120308	-2.02272	-1.87556	-4.76062	1.323816	-1.23407	4.508187	0.813138	2.101614
H	1.837747	-0.30926	-2.16121	-4.26127	-0.22161	-1.95148	3.12332	-0.0702	2.776929
H	-2.43384	-2.88252	-1.39036	3.856961	2.829811	0.47938	-6.34082	-0.89014	0.89561
H	-2.09107	-1.21091	-2.1048	3.263396	2.126739	-1.13181	-5.08146	-1.99254	0.083531
H	-3.17855	-1.91035	1.770082	3.623574	0.014855	2.387684	-4.2619	1.591171	1.881536
H	-4.78391	-1.78868	1.057224	5.22135	-0.21598	1.690253	-4.57481	2.149869	0.241617
H	-3.71791	-3.13551	0.616133	4.70434	1.401345	2.197723	-5.8874	1.44722	1.203614
H	2.022013	-2.32747	1.874789	-2.86257	2.706118	-0.76106	3.843805	2.402129	0.401059
H	0.933891	-1.09505	2.523203	-1.11241	2.584582	-0.58216	2.453021	2.773854	-0.62063
H	2.67731	-0.79654	2.462014	-2.15968	2.531251	0.850726	3.768579	1.767201	-1.24656
H	3.517892	-2.59066	-0.07173	-4.74631	0.34503	1.567334	5.048645	-1.18177	-0.05801
H	0.440111	3.294276	-0.32668	-0.935	-1.28517	-1.94377	0.272691	2.304489	-1.72622
H	-1.28791	3.065575	-0.07944	0.765759	-1.74232	-1.90689	-1.28949	2.100826	-0.92295
H	-0.20285	3.141924	1.320848	-0.37526	-2.52668	-0.80982	0.163281	2.408468	0.036899
H	-3.51606	0.638765	-2.05905	2.231593	0.136712	-1.75353	-2.15798	-1.37067	-1.28618
	4c-10			4c-11			4c-12		
C	-1.1013	-0.06706	0.548806	1.554904	0.715571	0.642585	1.548605	0.776084	0.575474
C	-2.14208	0.691531	-0.32064	2.118468	-0.69135	0.294493	2.11386	-0.6614	0.386678
C	-3.52082	0.098353	0.062728	3.458844	-0.43951	-0.42432	3.455526	-0.49249	-0.35438
C	-3.18161	-1.34739	0.411999	3.188752	0.85974	-1.18142	3.191552	0.721586	-1.24524
C	-1.87994	-1.23676	1.207076	2.411037	1.715804	-0.18262	2.433525	1.680449	-0.32797
C	0.165145	-0.5046	-0.2305	0.034738	0.885916	0.374786	0.032623	0.911631	0.271992
C	1.246753	-0.93836	0.77886	-0.76482	-0.23711	1.060827	-0.78182	-0.1356	1.046451
C	2.576079	-1.49617	0.249278	-2.29807	-0.14322	1.064581	-2.31238	-0.015	1.040359
C	3.530986	-0.52558	-0.4764	-2.95913	-0.013	-0.30716	-2.99215	-0.01293	-0.34301

C	3.814926	0.727602	0.339488	-4.46394	-0.1912	-0.26044	-4.48273	-0.2445	-0.2014
O	0.641586	0.625899	-0.99656	-0.17139	0.860799	-1.04766	-0.19999	0.626503	-1.1256
C	-4.56951	0.233593	-1.03585	3.904848	-1.5897	-1.32109	3.894045	-1.7329	-1.12487
C	3.617439	1.957149	-0.14565	-5.26352	0.800091	-0.66631	-5.3316	0.787415	-0.19801
C	4.368696	0.494937	1.7225	-4.99587	-1.5104	0.238192	-4.93717	-1.67014	-0.03537
H	-0.75272	0.611306	1.337796	1.71247	0.900648	1.711395	1.682638	1.074927	1.620936
C	-2.06331	2.211586	-0.19929	2.243804	-1.64692	1.480033	2.233409	-1.47718	1.672501
O	-4.01392	0.680279	1.279957	4.445187	-0.21158	0.596819	4.442573	-0.15898	0.634824
C	-0.11629	-1.58972	-1.26699	-0.43072	2.25215	0.89024	-0.45387	2.324846	0.607082
O	3.129153	-0.25732	-1.8059	-2.35544	-0.99666	-1.16837	-2.4579	-1.01244	-1.20342
H	-1.97611	0.440868	-1.3731	1.463715	-1.14503	-0.45992	1.459557	-1.19391	-0.313
H	-3.99226	-1.82794	0.96548	4.111646	1.330382	-1.53843	4.11518	1.145798	-1.65254
H	-3.02131	-1.90988	-0.51364	2.563713	0.642967	-2.05337	2.563464	0.416919	-2.08914
H	-1.32531	-2.17777	1.218538	1.806883	2.463095	-0.69846	1.872588	2.425956	-0.89812
H	-2.10553	-0.98908	2.246213	3.101047	2.247995	0.476694	3.140142	2.237301	0.29061
H	0.808967	-1.70451	1.425883	-0.44259	-0.28409	2.106065	-0.4562	-0.09994	2.090699
H	1.448069	-0.08136	1.435202	-0.48509	-1.19406	0.611355	-0.5119	-1.12718	0.673038
H	2.399971	-2.34191	-0.42146	-2.63706	0.698297	1.675853	-2.63286	0.885976	1.573278
H	3.113807	-1.90852	1.109316	-2.66613	-1.05056	1.553208	-2.69284	-0.86296	1.620158
H	4.486338	-1.05462	-0.57545	-2.74342	0.98136	-0.71421	-2.84197	0.97052	-0.80445
H	0.916594	1.31426	-0.37242	-0.6821	0.065699	-1.27142	0.096841	1.375005	-1.65923
H	-5.50824	-0.2271	-0.71705	4.843851	-1.33936	-1.82648	4.833771	-1.54264	-1.65426
H	-4.76922	1.285635	-1.26695	4.069862	-2.49813	-0.73559	4.054351	-2.57464	-0.44615
H	-4.2372	-0.25329	-1.95656	3.155955	-1.80074	-2.0893	3.143143	-2.02013	-1.86567
H	3.846699	2.838198	0.447686	-6.34592	0.702795	-0.64673	-6.39886	0.646019	-0.04826
H	3.254893	2.117524	-1.1564	-4.86055	1.744412	-1.02378	-4.98562	1.808482	-0.34137
H	3.62426	0.039573	2.38346	-4.45168	-2.34395	-0.21386	-4.6964	-2.24921	-0.93032
H	5.222441	-0.18981	1.68682	-4.87758	-1.59763	1.322491	-4.42667	-2.15659	0.801508
H	4.69816	1.429561	2.179954	-6.05717	-1.61974	0.007925	-6.01367	-1.72287	0.139442
H	-2.86708	2.702747	-0.75868	2.696754	-2.59596	1.177137	2.695117	-2.45025	1.480221
H	-1.11926	2.580365	-0.6061	1.268699	-1.87448	1.915581	1.254188	-1.66238	2.118611
H	-2.12465	2.532749	0.84535	2.871684	-1.21125	2.26046	2.849939	-0.95376	2.406578
H	-4.22718	1.606191	1.104966	5.248219	0.098555	0.158194	5.259404	0.061168	0.167835
H	-0.93561	-1.30173	-1.92906	0.152822	3.06219	0.448764	0.154719	3.092227	0.122162
H	0.764676	-1.76085	-1.88902	-1.47824	2.428041	0.635311	-1.48749	2.468831	0.286075
H	-0.37734	-2.53401	-0.7849	-0.32617	2.309862	1.977891	-0.40425	2.495557	1.685821
H	2.228458	0.117786	-1.75837	-2.79642	-0.9431	-2.02737	-1.57242	-0.68814	-1.44256
	4c-13			4c-14			4c-15		
C	-1.2755	0.593799	0.714904	1.546311	0.769681	0.577474	1.414509	0.107124	-0.62664
C	-2.38312	0.502285	-0.37386	2.114716	-0.66258	0.36744	2.448999	0.630481	0.406943
C	-3.10241	-0.85493	-0.14079	3.467747	-0.47587	-0.36322	3.742674	-0.18714	0.167348
C	-1.99704	-1.72975	0.461405	3.211093	0.751142	-1.22873	3.210057	-1.52757	-0.33106
C	-1.26346	-0.7877	1.417939	2.433404	1.690614	-0.30712	2.070273	-1.13967	-1.27474

C	0.119561	1.039744	0.211576	0.03058	0.906855	0.274353	0.008542	-0.13802	-0.01739
C	0.747305	-0.02014	-0.7236	-0.7822	-0.14902	1.039052	-0.99745	-0.30871	-1.1713
C	2.051002	0.321163	-1.46364	-2.31266	-0.02784	1.039647	-2.44304	-0.68664	-0.82492
C	3.354407	0.31403	-0.65689	-2.9965	-0.01028	-0.34158	-3.17079	0.266867	0.129963
C	3.581983	-0.98407	0.095521	-4.4867	-0.24348	-0.19847	-4.64661	-0.06797	0.22651
O	0.883787	1.18486	1.411345	-0.20132	0.634307	-1.12551	-0.33128	1.0166	0.771181
C	-3.72118	-1.43379	-1.40282	3.910769	-1.70145	-1.15469	4.628655	-0.29885	1.403532
C	3.613714	-1.05077	1.428724	-5.33573	0.788208	-0.18507	-5.58308	0.674132	-0.37101
C	3.765983	-2.20013	-0.77506	-4.9405	-1.67048	-0.04283	-4.97533	-1.29452	1.037033
H	-1.55636	1.361905	1.444121	1.676797	1.052553	1.627957	1.275213	0.878451	-1.39266
C	-3.33053	1.699479	-0.40961	2.227542	-1.49681	1.642406	2.642363	2.143597	0.364309
O	-4.1908	-0.72633	0.787256	4.503109	-0.08901	0.554297	4.506159	0.35148	-0.92454
C	0.024569	2.405439	-0.47965	-0.45711	2.316398	0.62287	-0.01859	-1.33615	0.935272
O	3.36019	1.468091	0.184769	-2.46438	-1.00005	-1.21449	-2.92552	1.596571	-0.33205
H	-1.91187	0.426548	-1.36014	1.464422	-1.18857	-0.34098	2.103341	0.377233	1.414562
H	-2.41011	-2.61992	0.94326	4.143031	1.180382	-1.60494	3.994736	-2.11942	-0.80974
H	-1.32222	-2.05952	-0.33589	2.597237	0.456132	-2.08575	2.82702	-2.09768	0.521959
H	-0.25448	-1.12088	1.665798	1.871108	2.437196	-0.87471	1.37276	-1.96468	-1.43952
H	-1.79659	-0.727	2.373355	3.128941	2.246983	0.323908	2.481204	-0.87374	-2.25108
H	0.01351	-0.25945	-1.49943	-0.45299	-0.12629	2.082665	-0.61719	-1.08025	-1.84745
H	0.899283	-0.93927	-0.14933	-0.51428	-1.13598	0.6519	-1.00126	0.620416	-1.75188
H	1.977639	1.289486	-1.9662	-2.63094	0.867243	1.583635	-2.47663	-1.69299	-0.39857
H	2.17496	-0.41551	-2.26313	-2.6916	-0.88204	1.611193	-3.0108	-0.72485	-1.76076
H	4.173998	0.418349	-1.38197	-2.84808	0.978191	-0.79253	-2.73627	0.16101	1.133483
H	1.798931	1.391737	1.147567	0.098829	1.386209	-1.65254	-1.0079	1.524215	0.29474
H	-4.20114	-2.39121	-1.18319	4.847529	-1.49037	-1.67718	5.509667	-0.90697	1.181459
H	-4.47913	-0.75822	-1.80907	4.076753	-2.56174	-0.49721	4.971476	0.688098	1.733453
H	-2.95542	-1.59594	-2.16545	3.156014	-1.98506	-1.89274	4.08705	-0.75991	2.233937
H	3.794146	-1.99422	1.935784	-6.40268	0.645398	-0.0345	-6.63629	0.421163	-0.28855
H	3.441062	-0.18292	2.057163	-4.99026	1.810454	-0.321	-5.32597	1.544384	-0.96749
H	2.827657	-2.4687	-1.27141	-4.69859	-2.24333	-0.94146	-4.55252	-2.19473	0.580089
H	4.504419	-2.01653	-1.56214	-4.43074	-2.16237	0.791352	-4.55455	-1.21964	2.044919
H	4.093054	-3.06051	-0.18906	-6.01712	-1.72499	0.130651	-6.05394	-1.43626	1.123115
H	-4.12863	1.550981	-1.14299	2.72043	-2.45604	1.450495	3.409214	2.476497	1.073208
H	-2.79343	2.612095	-0.67892	1.246804	-1.72246	2.064406	1.70626	2.640078	0.626341
H	-3.80091	1.863914	0.563484	2.797028	-0.96525	2.411229	2.927721	2.480518	-0.63777
H	-3.84777	-0.37836	1.620877	4.695864	-0.84438	1.124905	4.819539	1.227469	-0.66413
H	-0.52554	3.106075	0.154221	0.150262	3.088425	0.144102	0.723662	-1.22073	1.729129
H	1.022469	2.820476	-0.64493	-1.49112	2.462188	0.304137	-0.99631	-1.41558	1.415866
H	-0.47914	2.342419	-1.44911	-0.40652	2.477582	1.703046	0.180441	-2.2756	0.413398
H	4.204461	1.503167	0.65489	-1.58007	-0.67266	-1.45372	-3.4018	2.204527	0.250047
	4c-16			4c-17			4c-18		
C	1.138907	-0.04868	-0.53382	1.528406	0.875478	0.378	1.138887	-0.04864	-0.53392

C	2.218611	0.713897	0.285907	2.137907	-0.54872	0.525017	2.218541	0.713922	0.285898
C	3.575674	0.0712	-0.09545	3.484348	-0.50834	-0.22543	3.575631	0.071222	-0.09534
C	3.188638	-1.38327	-0.38275	3.19511	0.458994	-1.37348	3.188615	-1.38321	-0.38288
C	1.872026	-1.26814	-1.15547	2.394112	1.576549	-0.70655	1.872089	-1.26795	-1.15571
C	-0.12289	-0.41429	0.286148	0.011679	0.891417	0.050232	-0.1229	-0.41437	0.286032
C	-1.22987	-0.83883	-0.70747	-0.77737	0.028991	1.047137	-1.22986	-0.83882	-0.70763
C	-2.52737	-1.45431	-0.16108	-2.3099	0.110331	1.020477	-2.52741	-1.45432	-0.16134
C	-3.54149	-0.51492	0.50174	-2.98509	-0.22955	-0.32441	-3.54147	-0.51501	0.501669
C	-3.95573	0.641437	-0.38928	-4.47726	-0.40976	-0.14803	-3.95569	0.641544	-0.38911
O	-0.49013	0.788364	0.964092	-0.20318	0.282376	-1.24273	-0.49014	0.788193	0.964111
C	4.641638	0.217629	0.977936	3.974764	-1.87621	-0.68711	4.641438	0.217519	0.978213
C	-3.68975	1.915271	-0.09033	-5.09377	-1.55267	-0.45906	-3.68965	1.915314	-0.08994
C	-4.69111	0.246565	-1.64389	-5.21925	0.784112	0.398011	-4.69108	0.246938	-1.6438
H	0.785997	0.610982	-1.33545	1.644728	1.409736	1.32736	0.785918	0.611097	-1.33546
C	2.168836	2.228891	0.114124	2.265107	-1.0474	1.963251	2.168747	2.228921	0.114133
O	4.140739	0.671544	-1.27303	4.444175	0.07759	0.668604	4.140898	0.671667	-1.27278
C	0.150003	-1.50769	1.32817	-0.51714	2.329107	0.047524	0.150017	-1.50789	1.327943
O	-3.00577	-0.0994	1.759205	-2.41967	-1.38595	-0.9154	-3.00571	-0.09978	1.759212
H	2.062602	0.499394	1.347855	1.509019	-1.24645	-0.03968	2.062428	0.499426	1.347825
H	3.976013	-1.91025	-0.92803	4.110172	0.810087	-1.86174	3.976041	-1.91011	-0.92817
H	3.022398	-1.90704	0.56479	2.589102	-0.05279	-2.12865	3.0223	-1.90712	0.564572
H	1.292674	-2.19293	-1.11548	1.816049	2.149612	-1.43657	1.292744	-2.19276	-1.11601
H	2.069815	-1.08915	-2.21791	3.073883	2.285923	-0.2304	2.070036	-1.08872	-2.21808
H	-0.80015	-1.57795	-1.39112	-0.45511	0.311516	2.054102	-0.80015	-1.57788	-1.39134
H	-1.47071	0.040146	-1.31615	-0.48165	-1.01449	0.910085	-1.47069	0.040206	-1.31624
H	-2.31055	-2.25966	0.546398	-2.63596	1.104697	1.340648	-2.31064	-2.25982	0.54598
H	-3.04485	-1.93173	-0.99868	-2.68038	-0.59438	1.773612	-3.04491	-1.93155	-0.99903
H	-4.44272	-1.11314	0.697579	-2.84509	0.622019	-1.00717	-4.44272	-1.11323	0.697418
H	-1.34348	0.616587	1.402706	0.078058	0.89336	-1.93611	-1.34347	0.616359	1.402743
H	5.568416	-0.26747	0.65959	4.916181	-1.77981	-1.23874	5.568264	-0.26754	0.659948
H	4.856885	1.272567	1.168225	4.151426	-2.53398	0.167839	4.856643	1.272438	1.168654
H	4.311096	-0.24359	1.911795	3.245231	-2.35156	-1.34813	4.310758	-0.24381	1.911966
H	-4.01508	2.720906	-0.74215	-6.16796	-1.66083	-0.33214	-4.01494	2.721074	-0.74162
H	-3.12465	2.198135	0.792025	-4.54121	-2.39988	-0.84985	-3.12449	2.198001	0.792435
H	-4.0175	-0.25148	-2.3489	-4.97823	0.95479	1.451919	-4.01747	-0.25097	-2.3489
H	-5.50214	-0.45524	-1.4243	-4.94971	1.696724	-0.14436	-5.50211	-0.4549	-1.42436
H	-5.1154	1.118143	-2.14491	-6.29925	0.645516	0.319783	-5.11536	1.118622	-2.14465
H	2.960917	2.720309	0.687638	2.759561	-2.02265	1.999469	2.960753	2.720365	0.687728
H	1.20459	2.605595	0.45979	1.286562	-1.15974	2.434284	1.204451	2.605572	0.459724
H	2.291993	2.517942	-0.93405	2.855422	-0.35082	2.562628	2.291973	2.518013	-0.93402
H	3.475267	0.66626	-1.97329	5.263263	0.206251	0.172518	3.475464	0.666638	-1.97308
H	1.014841	-1.24956	1.944103	0.070387	2.979083	-0.60557	1.014821	-1.24977	1.943932
H	-0.70692	-1.60962	1.998869	-1.55336	2.364897	-0.29485	-0.70692	-1.60992	1.998609

H	0.331935	-2.4832	0.868274	-0.47576	2.748499	1.056396	0.332011	-2.48334	0.867942
H	-3.66674	0.442371	2.211657	-1.54596	-1.10141	-1.23397	-3.66664	0.441929	2.211773
	4c-19			4c-20					
C	1.527475	0.879371	0.377964	1.276181	0.585184	-0.71899			
C	2.136988	-0.54443	0.519998	2.381105	0.505578	0.372301			
C	3.487003	-0.50284	-0.23828	3.104791	-0.84063	0.144896			
C	3.201003	0.472618	-1.37277	2.003528	-1.72862	-0.44324			
C	2.395012	1.58488	-0.70218	1.271723	-0.79982	-1.41148			
C	0.010767	0.894662	0.050347	-0.11938	1.033562	-0.21974			
C	-0.77676	0.031185	1.047767	-0.7498	-0.02063	0.720242			
C	-2.30936	0.11058	1.021905	-2.0528	0.326195	1.459146			
C	-2.98385	-0.23095	-0.32297	-3.35741	0.318154	0.654192			
C	-4.47571	-0.41374	-0.14667	-3.59013	-0.98367	-0.09011			
O	-0.20377	0.285451	-1.24212	-0.88397	1.172676	-1.42048			
C	3.965063	-1.87051	-0.71334	3.744324	-1.40873	1.407721			
C	-5.09022	-1.55746	-0.45863	-3.62147	-1.05876	-1.42287			
C	-5.21965	0.778547	0.400135	-3.77995	-2.19326	0.78824			
H	1.641879	1.411143	1.329093	1.560668	1.345997	-1.45315			
C	2.267545	-1.04065	1.959112	3.321086	1.709142	0.403965			
O	4.508936	0.120379	0.555925	4.117819	-0.60905	-0.84721			
C	-0.51936	2.331868	0.048282	-0.02621	2.403322	0.464068			
O	-2.41626	-1.38669	-0.91329	-3.36189	1.467226	-0.19402			
H	1.504479	-1.24458	-0.03759	1.91106	0.427037	1.359309			
H	4.122607	0.823495	-1.84354	2.410745	-2.62843	-0.91756			
H	2.598717	-0.03641	-2.13198	1.335432	-2.05682	0.360786			
H	1.817079	2.159752	-1.43105	0.265327	-1.13991	-1.66205			
H	3.073277	2.293509	-0.22328	1.829884	-0.73906	-2.34865			
H	-0.45404	0.313925	2.054673	-0.01658	-0.25759	1.497435			
H	-0.48059	-1.01207	0.909691	-0.90226	-0.94195	0.149514			
H	-2.63667	1.104639	1.341661	-1.97724	1.296773	1.95712			
H	-2.67875	-0.59434	1.775388	-2.178	-0.40628	2.262399			
H	-2.84511	0.620473	-1.00607	-4.17558	0.42872	1.380092			
H	0.087236	0.892125	-1.93536	-1.79807	1.383876	-1.15751			
H	4.898039	-1.76657	-1.27354	4.239135	-2.36215	1.193859			
H	4.150935	-2.54279	0.131244	4.495671	-0.71982	1.803469			
H	3.220919	-2.34295	-1.35973	2.992503	-1.58648	2.181897			
H	-6.16425	-1.66752	-0.33198	-3.80566	-2.00463	-1.92406			
H	-4.5362	-2.40334	-0.85023	-3.4439	-0.19574	-2.05656			
H	-4.97862	0.949295	1.454035	-2.84318	-2.4621	1.287398			
H	-4.95194	1.691831	-0.14199	-4.51848	-2.00152	1.573313			
H	-6.29941	0.638036	0.322165	-4.10981	-3.05627	0.207644			
H	2.790219	-2.00225	2.004993	4.114316	1.575213	1.146009			
H	1.291475	-1.18745	2.4246	2.774448	2.619535	0.661993			

H	2.815915	-0.32105	2.575133	3.792374	1.857996	-0.56955			
H	4.717049	-0.469	1.292623	4.466315	-1.47064	-1.11138			
H	0.066784	2.982209	-0.60555	0.523943	3.100354	-0.17371			
H	-1.55591	2.36643	-0.29313	-1.02424	2.819216	0.626964			
H	-0.47744	2.751463	1.057081	0.477474	2.346124	1.434017			
H	-1.54465	-1.09996	-1.23558	-4.20555	1.499537	-0.6654			

Table S 23. Energy analysis for 2*S*, 3*R*, 6*R*, 7*S*, 10*R*-4 in the Chloroform at MPW1PW91/6-31G+d, p level

Conf.	Steric Energy	Relative Energy	Distribution
	(kJ/mol)	(kJ/mol)	(%)
4c-1	-813.598574	0.00232	3.65%
4c-2	-813.598518	0.002376	3.44%
4c-3	-813.598132	0.002762	2.29%
4c-4	-813.598232	0.002662	2.54%
4c-5	-813.598855	0.002039	4.92%
4c-6	-813.598856	0.002038	4.92%
4c-7	-813.597949	0.002945	1.88%
4c-8	-813.599858	0.001036	14.22%
4c-9	-813.597445	0.003449	1.11%
4c-10	-813.600894	0	42.59%
4c-11	-813.597637	0.003257	1.35%
4c-12	-813.597969	0.002925	1.93%
4c-13	-813.597692	0.003202	1.44%
4c-14	-813.598282	0.002612	2.68%
4c-15	-813.597422	0.003472	1.08%
4c-16	-813.5974	0.003494	1.05%
4c-17	-813.598173	0.002721	2.39%
4c-18	-813.5974	0.003494	1.05%
4c-19	-813.598468	0.002426	3.27%
4c-20	-813.598091	0.002803	2.19%

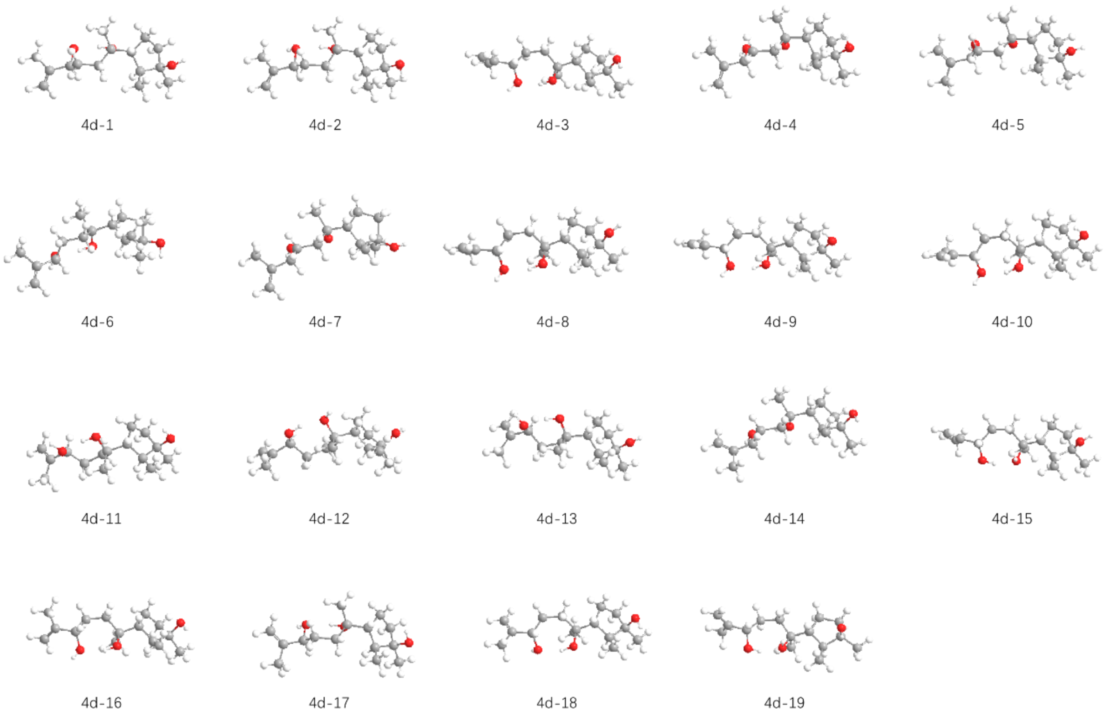

Figure S 86. Most stable conformers of 2*S*, 3*R*, 6*R*, 7*S*, 10*S*-4 calculated at MPW1PW91/6-31G+d, p level

Table S 24. Optimized Z-Matrixes of 2*S*, 3*R*, 6*R*, 7*S*, 10*S*-4 conformations in the Chloroform at MPW1PW91/6-31G+d, p level

	4d-1			4d-2			4d-3		
C	1.580598	0.701285	0.596315	1.577437	0.693117	0.598125	1.435202	-0.04766	-0.63808
C	2.147354	-0.72434	0.33148	2.151436	-0.72398	0.309776	2.514784	0.719463	0.173172
C	3.505951	-0.51378	-0.3665	3.520967	-0.49176	-0.37557	3.786806	-0.1616	0.124255
C	3.268708	0.757075	-1.1809	3.286261	0.79067	-1.1624	3.216597	-1.57532	0.06167
C	2.477252	1.659637	-0.23545	2.473724	1.670068	-0.21226	2.035652	-1.45305	-0.9032
C	0.074876	0.86693	0.272188	0.07148	0.858414	0.275751	0.043539	-0.06081	0.039364
C	-0.75058	-0.2211	0.994417	-0.75008	-0.24328	0.981916	-0.99172	-0.54796	-0.99923
C	-2.28489	-0.12812	0.978294	-2.28447	-0.15102	0.974492	-2.434	-0.82409	-0.5476
C	-2.9738	-0.40896	-0.35629	-2.97945	-0.40902	-0.3615	-3.28903	0.39365	-0.20082
C	-4.44997	-0.71499	-0.19781	-4.45641	-0.71057	-0.20142	-4.77037	0.074009	-0.16199
O	-0.04158	0.73247	-1.14626	-0.0445	0.744031	-1.14418	-0.21681	1.301135	0.393305
C	3.957608	-1.70401	-1.20661	3.980909	-1.66387	-1.23608	4.73241	0.068349	1.298478
C	-4.95102	-1.86103	-0.66838	-4.96443	-1.84808	-0.68497	-5.62312	0.753577	-0.93457
C	-5.29413	0.326151	0.488848	-5.29328	0.325335	0.501949	-5.2107	-1.01415	0.781632
H	1.696575	0.932053	1.661276	1.689747	0.905786	1.667418	1.295352	0.468863	-1.59415
C	2.240834	-1.61757	1.567598	2.238727	-1.63793	1.531638	2.726196	2.157534	-0.29215
O	4.47412	-0.25581	0.665386	4.538004	-0.17432	0.589858	4.499698	0.020172	-1.11042
C	-0.40236	2.259838	0.709345	-0.41009	2.243232	0.73324	0.01452	-0.92382	1.307854
O	-2.80789	0.747652	-1.20006	-2.81145	0.758725	-1.189	-2.86775	0.889413	1.085155
H	1.50651	-1.2105	-0.41266	1.516937	-1.20276	-0.44468	2.205108	0.760461	1.222374
H	4.205633	1.203805	-1.53215	4.229098	1.242932	-1.48193	3.970629	-2.29982	-0.25726

H	2.662749	0.513441	-2.05908	2.694656	0.556821	-2.05236	2.869361	-1.86313	1.059639
H	1.900737	2.398868	-0.79235	1.894889	2.41078	-0.76487	1.31762	-2.26653	-0.77451
H	3.157838	2.200322	0.426981	3.142403	2.20829	0.4637	2.397276	-1.51085	-1.93214
H	-0.44563	-0.22032	2.046806	-0.44044	-0.26155	2.032921	-0.61829	-1.47764	-1.44047
H	-0.46663	-1.19651	0.58699	-0.46723	-1.21055	0.554463	-1.00838	0.191092	-1.80885
H	-2.6261	0.838903	1.359581	-2.62384	0.809153	1.374157	-2.45226	-1.52505	0.292614
H	-2.65451	-0.88132	1.682	-2.65101	-0.9164	1.6666	-2.93855	-1.32933	-1.37778
H	-2.48534	-1.26819	-0.83359	-2.4968	-1.26355	-0.853	-3.11623	1.17275	-0.95414
H	-0.98417	0.852373	-1.36462	-0.98725	0.865715	-1.36065	-1.10461	1.31927	0.796642
H	4.91228	-1.48646	-1.69773	4.930208	-1.42146	-1.72113	5.595144	-0.59914	1.223265
H	4.095252	-2.59087	-0.58213	4.130327	-2.56698	-0.63382	5.101468	1.099813	1.311585
H	3.224481	-1.93482	-1.98423	3.243557	-1.89457	-2.00943	4.228496	-0.11885	2.250607
H	-6.00621	-2.10302	-0.57175	-6.02037	-2.08649	-0.58756	-6.69014	0.546999	-0.92313
H	-4.31981	-2.59768	-1.15923	-4.33849	-2.58104	-1.18794	-5.27997	1.533699	-1.6096
H	-5.03159	0.402248	1.548605	-5.02772	0.385234	1.562004	-4.8474	-1.99036	0.445685
H	-5.12824	1.311666	0.045256	-5.12332	1.316068	0.07174	-4.80289	-0.84635	1.782201
H	-6.35633	0.084888	0.419525	-6.35691	0.090677	0.432032	-6.29886	-1.06341	0.850665
H	2.705773	-2.57879	1.327301	2.73985	-2.58257	1.2922	3.527994	2.651607	0.268617
H	1.253254	-1.82663	1.984272	1.249855	-1.89152	1.91778	1.809452	2.730342	-0.1439
H	2.843288	-1.14055	2.344064	2.788526	-1.15462	2.345647	2.973493	2.199313	-1.35825
H	5.286463	0.036403	0.231528	4.707108	-0.96634	1.116571	4.826641	0.92904	-1.13309
H	0.239882	3.043481	0.303456	0.230129	3.034403	0.339065	0.819278	-0.63965	1.99033
H	-1.4184	2.450078	0.354011	-1.42645	2.43549	0.380017	-0.92975	-0.78365	1.840123
H	-0.40134	2.3471	1.800517	-0.40988	2.314608	1.825586	0.11492	-1.98905	1.081416
H	-3.24224	0.563305	-2.04447	-3.25041	0.588964	-2.03409	-3.40666	1.664989	1.294382
	4d-4			4d-5			4d-6		
C	1.580442	0.700741	0.589694	1.58691	-0.57489	-0.71672	1.573856	0.705942	0.636847
C	2.150167	-0.72543	0.328917	2.122896	0.781095	-0.1707	2.148796	-0.73088	0.453928
C	3.517158	-0.51364	-0.36966	3.489791	0.463515	0.467934	3.446793	-0.55944	-0.39824
C	3.275814	0.753963	-1.18929	3.27917	-0.94665	1.017021	3.860149	0.88852	-0.14112
C	2.469868	1.658581	-0.25449	2.507578	-1.65965	-0.09232	2.538815	1.648804	-0.11345
C	0.07317	0.865518	0.269947	0.08629	-0.83646	-0.43558	0.093755	0.872056	0.230896
C	-0.74997	-0.22191	0.99515	-0.76537	0.35303	-0.93194	-0.77819	-0.06392	1.098455
C	-2.28429	-0.12873	0.979931	-2.29721	0.226027	-0.94247	-2.30865	0.041287	1.009524
C	-2.9737	-0.41239	-0.35383	-3.00237	0.210906	0.416901	-2.95539	-0.44578	-0.28631
C	-4.45038	-0.71527	-0.19459	-4.47723	0.542447	0.274996	-4.43965	-0.71261	-0.1328
O	-0.0431	0.729332	-1.14729	-0.031	-0.98754	0.981388	0.012603	0.513071	-1.14999
C	3.967914	-1.70232	-1.20273	3.927931	1.47433	1.522625	3.149461	-0.81036	-1.8772
C	-4.95302	-1.86225	-0.6611	-5.41115	-0.41204	0.239524	-4.93703	-1.91852	-0.42308
C	-5.29308	0.329956	0.487644	-4.80362	2.009385	0.179138	-5.29532	0.433041	0.340047
H	1.695427	0.938915	1.654255	1.705378	-0.58514	-1.80609	1.632119	0.954011	1.703896
C	2.242134	-1.61703	1.565594	2.190065	1.90721	-1.20122	2.402458	-1.41434	1.797589
O	4.572071	-0.29717	0.583382	4.45375	0.428786	-0.5989	4.529858	-1.39654	0.016227

C	-0.40254	2.259203	0.706424	-0.35907	-2.12622	-1.14058	-0.33642	2.333789	0.408553
O	-2.80542	0.741595	-1.20082	-2.79616	-1.07814	1.003675	-2.74661	0.559928	-1.29737
H	1.50828	-1.21106	-0.41447	1.476171	1.092548	0.657421	1.440635	-1.33987	-0.11598
H	4.214499	1.197076	-1.53291	4.224769	-1.43501	1.278241	4.349738	0.935982	0.838053
H	2.669401	0.504673	-2.06517	2.667194	-0.89243	1.922742	4.567235	1.259604	-0.88824
H	1.884401	2.381489	-0.82241	1.951027	-2.51047	0.301356	2.180091	1.824694	-1.13148
H	3.13394	2.238059	0.396942	3.200438	-2.03748	-0.84824	2.629611	2.621677	0.375406
H	-0.44396	-0.21954	2.047276	-0.45999	0.566659	-1.96236	-0.51003	0.116101	2.14573
H	-0.46609	-1.19758	0.588511	-0.50385	1.23543	-0.33915	-0.49246	-1.09836	0.880338
H	-2.6251	0.839082	1.359502	-2.62918	-0.65466	-1.50107	-2.64547	1.06206	1.213807
H	-2.65336	-0.88052	1.685351	-2.67589	1.091218	-1.4945	-2.71592	-0.58079	1.813378
H	-2.4867	-1.27381	-0.82858	-2.53956	0.973345	1.061125	-2.46144	-1.37352	-0.60238
H	-0.98623	0.846028	-1.36617	-0.97045	-1.16801	1.168331	-0.91973	0.620411	-1.41512
H	4.915247	-1.47612	-1.69952	4.889601	1.181827	1.95802	4.030393	-0.59122	-2.48749
H	4.116712	-2.58564	-0.57562	4.046551	2.468405	1.082994	2.869087	-1.85857	-2.0343
H	3.223059	-1.93863	-1.96627	3.196706	1.535889	2.333203	2.311152	-0.19873	-2.21843
H	-6.00864	-2.10213	-0.56406	-6.46332	-0.16472	0.131358	-5.99744	-2.13559	-0.32455
H	-4.32286	-2.60169	-1.14907	-5.14972	-1.46349	0.311985	-4.29786	-2.73014	-0.76182
H	-5.03109	0.409716	1.547277	-4.5006	2.530724	1.092992	-5.0638	0.68857	1.378745
H	-5.12542	1.313466	0.04027	-4.26897	2.484931	-0.64918	-5.10894	1.327881	-0.26003
H	-6.35563	0.090192	0.41865	-5.87228	2.170723	0.028572	-6.35688	0.186464	0.280524
H	2.713296	-2.57504	1.326319	2.62953	2.812322	-0.77054	1.478628	-1.4495	2.38278
H	1.255422	-1.82977	1.981999	1.196531	2.1695	-1.57102	3.151992	-0.87192	2.381553
H	2.840494	-1.1431	2.348767	2.803806	1.612285	-2.05535	2.758644	-2.44085	1.674525
H	4.349471	0.476321	1.11799	5.274177	0.071374	-0.2348	4.317394	-2.30571	-0.23178
H	0.239956	3.04254	0.300161	0.300721	-2.95996	-0.89399	0.305575	2.994963	-0.17681
H	-1.41812	2.449636	0.350257	-1.37053	-2.40476	-0.83437	-1.36299	2.482102	0.063799
H	-0.40238	2.347168	1.797531	-0.35604	-1.99693	-2.22753	-0.28119	2.636414	1.45916
H	-3.24056	0.55626	-2.04465	-3.2627	-1.10118	1.850825	-3.15551	0.246554	-2.11611
	4d-7			4d-8			4d-9		
C	1.577518	0.713034	0.635816	1.43652	-0.0443	-0.64125	1.436836	-0.04795	-0.63546
C	2.148816	-0.72884	0.475467	2.516361	0.731216	0.163738	2.518962	0.726829	0.16972
C	3.423868	-0.57777	-0.39878	3.781167	-0.14999	0.131089	3.792417	-0.15407	0.126944
C	3.859957	0.871854	-0.15546	3.203645	-1.56768	0.090846	3.21626	-1.57304	0.081517
C	2.545785	1.646148	-0.124	2.03627	-1.4527	-0.89036	2.034503	-1.45941	-0.88506
C	0.098054	0.878134	0.227226	0.044202	-0.05316	0.035737	0.043375	-0.05672	0.039895
C	-0.77542	-0.04895	1.102825	-0.99063	-0.54851	-0.99964	-0.98996	-0.5535	-0.99635
C	-2.30577	0.055046	1.009537	-2.4319	-0.82615	-0.54547	-2.43201	-0.82788	-0.54298
C	-2.94927	-0.44712	-0.28219	-3.29047	0.390723	-0.20437	-3.28816	0.391337	-0.20409
C	-4.43294	-0.71686	-0.12857	-4.77052	0.065573	-0.16011	-4.76897	0.069772	-0.16115
O	0.016142	0.509004	-1.15067	-0.21805	1.310558	0.379726	-0.21755	1.307026	0.381074
C	3.097065	-0.8282	-1.87231	4.728387	0.094342	1.300834	4.736604	0.083505	1.293952
C	-4.92616	-1.92716	-0.4075	-5.62765	0.735678	-0.93608	-5.6235	0.742489	-0.93771

C	-5.29292	0.430885	0.33122	-5.20468	-1.0167	0.793124	-5.2067	-1.01166	0.791359
H	1.633879	0.974573	1.699536	1.298004	0.462885	-1.60234	1.293222	0.460497	-1.5963
C	2.419634	-1.38165	1.831843	2.736299	2.162754	-0.31606	2.730957	2.163005	-0.29911
O	4.418281	-1.51559	0.027586	4.458182	0.127531	-1.10601	4.577349	0.096484	-1.05089
C	-0.3296	2.342097	0.393398	0.014595	-0.90661	1.310834	0.013979	-0.90922	1.315454
O	-2.7421	0.5493	-1.30305	-2.86842	0.896324	1.077389	-2.86569	0.896883	1.077553
H	1.434773	-1.3526	-0.06919	2.203864	0.779526	1.211772	2.20726	0.767527	1.218049
H	4.35669	0.922044	0.820063	3.951134	-2.31262	-0.20319	3.968406	-2.3047	-0.22496
H	4.564039	1.234626	-0.91157	2.84249	-1.83974	1.088808	2.859266	-1.85016	1.079312
H	2.183243	1.821361	-1.14065	1.315745	-2.26456	-0.76641	1.315043	-2.26923	-0.74727
H	2.647035	2.619937	0.361104	2.411138	-1.51555	-1.91453	2.381249	-1.54542	-1.92057
H	-0.5091	0.142149	2.148596	-0.61556	-1.47952	-1.43672	-0.6159	-1.48625	-1.43076
H	-0.48949	-1.08564	0.896605	-1.00957	0.186479	-1.81285	-1.00737	0.17978	-1.81121
H	-2.64365	1.077908	1.201458	-2.44747	-1.52287	0.298334	-2.4494	-1.52344	0.301673
H	-2.71439	-0.55813	1.819489	-2.93583	-1.33709	-1.37257	-2.93592	-1.33898	-1.36992
H	-2.45189	-1.37628	-0.58834	-3.12228	1.165505	-0.96314	-3.11742	1.165123	-0.96332
H	-0.91668	0.612067	-1.41598	-1.10542	1.329981	0.783646	-1.10496	1.328339	0.785385
H	3.968163	-0.62209	-2.50525	5.598638	-0.56816	1.238525	5.6061	-0.5751	1.217584
H	2.806378	-1.87351	-2.01075	5.08803	1.12681	1.295191	5.091876	1.117536	1.300385
H	2.267546	-0.20059	-2.20525	4.229522	-0.09523	2.255265	4.231714	-0.11632	2.242199
H	-5.9861	-2.14641	-0.30861	-6.69382	0.52494	-0.9206	-6.69025	0.534744	-0.92324
H	-4.2839	-2.74024	-0.73672	-5.28911	1.511977	-1.6178	-5.28228	1.518218	-1.61874
H	-5.06422	0.697326	1.367814	-4.83801	-1.99416	0.464531	-4.84249	-1.99002	0.462688
H	-5.10798	1.320283	-0.27734	-4.79573	-0.839	1.791519	-4.79813	-0.83547	1.79017
H	-6.35364	0.180578	0.27205	-6.2925	-1.06982	0.864571	-6.29469	-1.06185	0.861929
H	2.792078	-2.40207	1.719963	3.516204	2.66353	0.266808	3.523441	2.655022	0.27318
H	1.497157	-1.41444	2.41985	1.809502	2.729547	-0.20873	1.807376	2.730312	-0.1714
H	3.161422	-0.81539	2.403849	3.038508	2.18058	-1.36603	3.012548	2.202802	-1.3557
H	5.18145	-1.40625	-0.55473	5.183085	-0.50599	-1.18766	4.006351	0.019619	-1.82639
H	0.314762	2.997833	-0.19558	0.81714	-0.61459	1.992584	0.817826	-0.61914	1.996342
H	-1.35522	2.489653	0.045454	-0.93093	-0.76451	1.840302	-0.93068	-0.76349	1.845242
H	-0.27604	2.652491	1.441825	0.117897	-1.97346	1.093206	0.113139	-1.97669	1.099344
H	-3.14664	0.225173	-2.11978	-3.40802	1.672776	1.281524	-3.40587	1.672672	1.282764
	4d-10			4d-11			4d-12		
C	1.436836	-0.04795	-0.63546	-1.58325	0.62867	0.663002	-1.57924	0.638861	0.63727
C	2.518959	0.72683	0.169717	-2.49614	0.422949	-0.58004	-2.47427	0.405826	-0.61434
C	3.792416	-0.15407	0.126946	-3.4212	-0.7778	-0.23983	-3.42084	-0.75704	-0.24109
C	3.216262	-1.57304	0.081519	-2.5612	-1.61315	0.715364	-2.58238	-1.58209	0.740798
C	2.034509	-1.45941	-0.88506	-1.87846	-0.56943	1.601517	-1.90295	-0.52312	1.607884
C	0.043376	-0.05673	0.039892	-0.07718	0.819515	0.357663	-0.0664	0.802517	0.340541
C	-0.98996	-0.55351	-0.99635	0.554149	-0.46556	-0.22328	0.570951	-0.49839	-0.1752
C	-2.43201	-0.82789	-0.54298	2.00024	-0.41287	-0.74254	2.014744	-0.46694	-0.7025
C	-3.28815	0.391335	-0.20409	3.103311	-0.29122	0.307732	3.12783	-0.30115	0.346431

C	-4.76897	0.069777	-0.16115	4.468196	-0.66611	-0.23497	4.480386	-0.6617	-0.23566
O	-0.21756	1.307019	0.381072	0.496418	1.128942	1.631806	0.586987	1.087792	1.60111
C	4.736598	0.083514	1.293958	-3.89175	-1.54156	-1.46697	-3.91489	-1.5503	-1.44606
C	-5.6235	0.742502	-0.93771	5.180934	-1.63167	0.352824	5.112317	-1.77499	0.146585
C	-5.2067	-1.01165	0.791354	4.96256	0.102104	-1.43238	5.048933	0.280992	-1.26292
H	1.29322	0.460495	-1.5963	-1.87341	1.556203	1.168799	-1.88681	1.584987	1.101167
C	2.730951	2.163006	-0.29911	-3.25403	1.674093	-1.01891	-3.19885	1.654495	-1.11319
O	4.577353	0.096489	-1.05089	-4.62262	-0.35255	0.421969	-4.53752	-0.177	0.450468
C	0.013982	-0.90922	1.315452	0.126517	2.013331	-0.58277	0.174945	1.975285	-0.61304
O	-2.86568	0.896873	1.077562	3.127384	1.070129	0.779091	3.21894	1.027128	0.847795
H	2.207254	0.767533	1.218045	-1.88102	0.097244	-1.42621	-1.85238	0.032737	-1.4357
H	3.968411	-2.3047	-0.22496	-3.16582	-2.33806	1.266921	-3.19417	-2.28869	1.311491
H	2.859265	-1.85015	1.079313	-1.81306	-2.16699	0.137996	-1.8402	-2.1654	0.184994
H	1.315053	-2.26923	-0.74728	-0.98232	-0.94362	2.098576	-1.02038	-0.89384	2.131986
H	2.381259	-1.54542	-1.92057	-2.55222	-0.25669	2.407079	-2.60409	-0.17479	2.369527
H	-0.6159	-1.48626	-1.43076	-0.06081	-0.79941	-1.06519	-0.05339	-0.86541	-0.99467
H	-1.00737	0.179769	-1.81122	0.490507	-1.25248	0.535455	0.50675	-1.24937	0.618979
H	-2.4494	-1.52345	0.301669	2.12204	0.383455	-1.48295	2.12829	0.300661	-1.47402
H	-2.93592	-1.33898	-1.36992	2.181251	-1.35345	-1.273	2.184937	-1.42888	-1.19821
H	-3.11741	1.165124	-0.96331	2.864934	-0.95349	1.149829	2.917427	-0.99422	1.174179
H	-1.10497	1.328329	0.785384	1.449914	1.272777	1.485008	0.194699	1.891083	1.971225
H	5.606095	-0.57509	1.217596	-4.52397	-2.38198	-1.16882	-4.57079	-2.36695	-1.12623
H	5.091866	1.117546	1.300391	-4.47515	-0.89376	-2.1272	-4.48173	-0.90799	-2.12538
H	4.231703	-0.11631	2.242203	-3.03811	-1.93068	-2.02744	-3.07828	-1.98844	-1.99726
H	-6.69025	0.534765	-0.92324	6.163489	-1.91704	-0.01387	6.070153	-2.06033	-0.28126
H	-5.28227	1.518233	-1.61874	4.801187	-2.17301	1.215802	4.692362	-2.43764	0.899364
H	-4.84251	-1.99001	0.462678	4.366581	-0.1343	-2.3194	4.401	0.341163	-2.14337
H	-4.79814	-0.83547	1.790165	4.874826	1.178903	-1.2639	5.123043	1.290242	-0.85072
H	-6.2947	-1.06183	0.861926	6.005149	-0.13412	-1.65239	6.039592	-0.04056	-1.59057
H	3.52343	2.655028	0.273175	-3.85317	2.083741	-0.20134	-3.85163	1.420638	-1.95929
H	1.807368	2.73031	-0.17141	-3.93172	1.455017	-1.8492	-2.48418	2.411388	-1.44568
H	3.012546	2.202801	-1.35571	-2.5618	2.45295	-1.34753	-3.81548	2.088481	-0.32349
H	4.006366	0.019588	-1.82639	-4.38157	0.1265	1.22573	-5.07795	-0.90226	0.790328
H	0.817827	-0.61914	1.99634	-0.41491	2.883984	-0.20308	-0.36	2.866196	-0.26869
H	-0.93068	-0.76349	1.845241	1.185359	2.27825	-0.64267	1.238964	2.216058	-0.6627
H	0.113144	-1.9767	1.099343	-0.22386	1.799762	-1.59709	-0.16977	1.744564	-1.62455
H	-3.40587	1.672661	1.282778	3.822011	1.140287	1.4486	2.354894	1.219669	1.260368
	4d-13			4d-14			4d-15		
C	1.583724	0.623815	-0.67186	1.584618	-0.57517	-0.71329	1.432374	-0.0569	-0.6426
C	2.494232	0.431656	0.574347	2.125553	0.774016	-0.15838	2.510745	0.722707	0.16257
C	3.414414	-0.76633	0.248102	3.502151	0.446448	0.471272	3.770744	-0.16653	0.142247
C	2.554005	-1.61595	-0.69288	3.295854	-0.96543	1.00306	3.186076	-1.58116	0.115871
C	1.88369	-0.58172	-1.59635	2.504086	-1.66736	-0.09967	2.024707	-1.4727	-0.87222

C	0.077989	0.817543	-0.3671	0.083195	-0.83388	-0.43464	0.031246	-0.06052	0.02025
C	-0.55433	-0.4623	0.224425	-0.7646	0.361521	-0.92383	-1.00639	-0.56029	-1.00585
C	-2.00024	-0.40611	0.743864	-2.29661	0.237654	-0.93983	-2.44745	-0.82411	-0.54482
C	-3.10431	-0.29257	-0.30634	-3.00526	0.210361	0.417571	-3.29539	0.410844	-0.19235
C	-4.46783	-0.66776	0.239783	-4.47955	0.544025	0.274595	-4.76976	0.060962	-0.14166
O	-0.49865	1.116972	-1.64272	-0.0355	-0.99262	0.981082	-0.2932	1.294728	0.413926
C	3.898102	-1.51643	1.484867	3.941885	1.445237	1.536607	4.716998	0.083741	1.31115
C	-5.1782	-1.63963	-0.34047	-5.4136	-0.40973	0.225744	-5.60569	0.4924	-1.09024
C	-4.96357	0.107246	1.43225	-4.80504	2.012064	0.193794	-5.22311	-0.79953	1.007797
H	1.878412	1.544506	-1.18581	1.701907	-0.57791	-1.80299	2.754551	2.14805	-0.32769
C	3.251481	1.688397	0.999517	2.188191	1.90657	-1.18265	4.446296	0.097955	-1.09748
O	4.542555	-0.24431	-0.47189	4.520421	0.337065	-0.53792	-0.01387	-0.86424	1.317539
C	-0.12603	2.019178	0.563523	-0.36471	-2.11793	-1.14827	-2.95062	0.980915	1.064513
O	-3.13198	1.065974	-0.78502	-2.80116	-1.08399	0.992929	1.308124	0.433553	-1.61644
H	1.880723	0.111873	1.424318	1.482485	1.084609	0.673003	2.193884	0.783184	1.208381
H	3.149575	-2.3566	-1.23773	4.247953	-1.44951	1.236944	3.930687	-2.33214	-0.16806
H	1.80798	-2.16365	-0.10646	2.698005	-0.91527	1.917838	2.820298	-1.84123	1.115146
H	0.991969	-0.95877	-2.1001	1.943947	-2.51384	0.298486	1.297074	-2.27648	-0.74052
H	2.584227	-0.27477	-2.37645	3.186299	-2.05054	-0.86203	2.40213	-1.54988	-1.89396
H	0.060626	-0.78946	1.069029	-0.45565	0.583802	-1.9515	-0.62761	-1.49345	-1.43387
H	-0.48992	-1.25485	-0.52843	-0.50353	1.237712	-0.32155	-1.0254	0.157507	-1.83732
H	-2.12206	0.395828	1.478278	-2.62845	-0.63692	-1.50798	-2.45871	-1.5213	0.298598
H	-2.18097	-1.34263	1.281722	-2.67243	1.108938	-1.48424	-2.953	-1.3335	-1.37237
H	-2.86504	-0.95901	-1.14491	-2.54384	0.966701	1.069982	-3.15254	1.156319	-0.9893
H	-1.45103	1.264724	-1.49444	-0.97488	-1.17556	1.165798	-0.24062	1.856453	-0.37236
H	4.537546	-2.35857	1.199098	4.897344	1.135378	1.96854	5.580704	-0.58756	1.25725
H	4.479358	-0.85586	2.134104	4.071405	2.44808	1.114473	5.087092	1.112238	1.295441
H	3.055281	-1.91405	2.057205	3.202479	1.51206	2.339008	4.214815	-0.09135	2.266401
H	-6.15954	-1.92545	0.029177	-6.46536	-0.161	0.116944	-6.65973	0.226497	-1.08246
H	-4.79764	-2.18576	-1.20008	-5.15267	-1.46199	0.287677	-5.26361	1.124607	-1.90602
H	-4.36548	-0.12048	2.320124	-4.50523	2.52305	1.114536	-4.71486	-1.76912	0.99847
H	-4.87991	1.183087	1.255836	-4.26709	2.49652	-0.62723	-4.98032	-0.3202	1.959359
H	-6.00501	-0.13118	1.655381	-5.87305	2.175607	0.041051	-6.29944	-0.97844	0.967348
H	3.915171	1.485064	1.845572	2.670264	2.798335	-0.76647	3.574965	2.620094	0.2208
H	2.555776	2.474322	1.30368	1.19261	2.207969	-1.51319	1.864918	2.764638	-0.17562
H	3.861337	2.070505	0.178257	2.744642	1.600052	-2.07421	3.011242	2.160384	-1.38929
H	5.053858	-0.99775	-0.79514	4.671404	1.217348	-0.90615	5.179673	-0.52708	-1.16876
H	0.413362	2.887043	0.174713	0.293273	-2.95451	-0.90671	0.781777	-0.55848	2.000074
H	-1.18515	2.283342	0.623932	-1.377	-2.39617	-0.8447	-0.96607	-0.70884	1.828657
H	0.227192	1.815265	1.578931	-0.36059	-1.98147	-2.23436	0.09141	-1.93302	1.11971
H	-3.82483	1.129865	-1.45695	-3.27138	-1.11543	1.837771	-2.01142	1.241428	1.002972
	4d-16			4d-17			4d-18		
C	1.425649	-0.33706	-0.5335	1.585927	-0.57549	-0.7076	1.448619	-0.15484	-0.61326

C	2.5195	0.711132	-0.19152	2.126829	0.78114	-0.1659	2.513961	0.757315	0.054527
C	3.790741	-0.10099	0.157572	3.504052	0.46216	0.468738	3.801834	-0.09689	0.150752
C	3.219887	-1.37745	0.767701	3.2924	-0.94713	1.021869	3.257355	-1.51077	0.329249
C	2.023776	-1.70904	-0.12664	2.497768	-1.66429	-0.07159	2.075676	-1.57348	-0.64009
C	0.048335	-0.02413	0.099969	0.08343	-0.83398	-0.4317	0.055294	-0.07897	0.058754
C	-1.00731	-0.9279	-0.5753	-0.76447	0.357538	-0.92867	-0.96565	-0.75339	-0.88599
C	-2.44037	-0.95375	-0.02263	-2.29631	0.231584	-0.94228	-2.39958	-1.02022	-0.39916
C	-3.29191	0.301314	-0.2354	-3.00347	0.212888	0.415996	-3.30036	0.205166	-0.25019
C	-4.76858	-0.00051	-0.05175	-4.47849	0.54272	0.272199	-4.78699	-0.07832	-0.10327
O	-0.20772	1.349162	-0.21178	-0.03443	-0.98549	0.984057	-0.2259	1.317531	0.18282
C	4.754153	0.642455	1.076815	3.942564	1.470572	1.517967	4.743065	0.344484	1.266501
C	-5.39874	0.250305	1.099145	-5.41061	-0.41324	0.230528	-5.27573	-1.28787	0.186656
C	-5.45987	-0.59618	-1.24955	-4.80679	2.00959	0.182459	-5.67298	1.129603	-0.26624
H	1.265328	-0.32249	-1.61731	1.703732	-0.59266	-1.79802	1.300373	0.191157	-1.64219
C	2.722411	1.762917	-1.27866	2.189819	1.905629	-1.19757	2.700594	2.099606	-0.64747
O	4.484488	-0.52091	-1.02919	4.552714	0.459145	-0.51515	4.510752	-0.11397	-1.09926
C	0.047256	-0.20268	1.624015	-0.3608	-2.12285	-1.13913	0.044734	-0.72212	1.452058
O	-2.83181	1.304649	0.675709	-2.79637	-1.07722	1.000177	-2.8964	1.052424	0.846299
H	2.227677	1.240513	0.721275	1.481627	1.092119	0.663447	2.204413	0.96922	1.083043
H	3.969097	-2.17227	0.810695	4.241321	-1.431	1.268747	4.02441	-2.26509	0.13488
H	2.889047	-1.16654	1.790079	2.686654	-0.8881	1.931055	2.915364	-1.63282	1.362418
H	1.309059	-2.36636	0.374312	1.929113	-2.49653	0.342854	1.373241	-2.36799	-0.37698
H	2.369464	-2.23898	-1.01691	3.168741	-2.09044	-0.82609	2.44073	-1.79536	-1.64518
H	-0.64091	-1.95915	-0.54363	-0.45666	0.572894	-1.95806	-0.55512	-1.72287	-1.18575
H	-1.04132	-0.64671	-1.63446	-0.50325	1.238128	-0.33323	-1.00804	-0.14548	-1.79738
H	-2.45557	-1.19067	1.046119	-2.62791	-0.6471	-1.50421	-2.39035	-1.59355	0.534731
H	-2.95383	-1.78155	-0.52053	-2.67326	1.098839	-1.49218	-2.86576	-1.66832	-1.14773
H	-3.13955	0.658738	-1.26458	-2.54267	0.974457	1.06261	-3.16244	0.840967	-1.13093
H	-1.08325	1.559902	0.161888	-0.97422	-1.16533	1.17096	-1.1277	1.396417	0.545736
H	5.61608	0.011235	1.309554	4.89818	1.167697	1.954384	5.615933	-0.31282	1.304741
H	5.122413	1.559344	0.603516	4.070783	2.463001	1.077221	5.096248	1.368591	1.103
H	4.264582	0.923072	2.013362	3.201075	1.537612	2.317597	4.241043	0.314564	2.237445
H	-6.45386	0.02357	1.222742	-6.46298	-0.16731	0.121341	-6.34374	-1.44182	0.314836
H	-4.87759	0.682638	1.948087	-5.14756	-1.46455	0.299215	-4.64352	-2.16296	0.298574
H	-5.44107	0.104197	-2.09093	-4.50815	2.526654	1.100163	-5.36529	1.928578	0.413809
H	-4.95981	-1.50966	-1.58654	-4.2696	2.490193	-0.6413	-5.5916	1.5261	-1.28365
H	-6.49992	-0.84138	-1.0284	-5.87509	2.170062	0.028557	-6.72019	0.889862	-0.07325
H	3.529117	2.460148	-1.02412	2.653542	2.802193	-0.77551	3.491353	2.696208	-0.17826
H	1.806035	2.341372	-1.40603	1.194737	2.183876	-1.55057	1.773584	2.672871	-0.59955
H	2.957792	1.299345	-2.24259	2.781685	1.608066	-2.06761	2.951371	1.96434	-1.70495
H	4.81594	0.269545	-1.47494	4.340893	-0.20078	-1.18845	4.820358	0.783818	-1.27685
H	0.864962	0.358879	2.082538	0.29937	-2.95699	-0.89453	0.842546	-0.31293	2.076352
H	-0.88681	0.173598	2.048536	-1.37178	-2.40245	-0.83277	-0.90017	-0.5163	1.961536

H	0.14557	-1.25267	1.913532	-0.35884	-1.99136	-2.2258	0.164375	-1.80807	1.406124
H	-3.38791	2.088497	0.565186	-3.26595	-1.10375	1.845576	-3.12822	0.599876	1.669485
	4d-19								
C	1.446473	-0.28462	-0.23522						
C	2.592616	0.62247	0.25692						
C	3.854044	-0.18729	-0.12148						
C	3.468009	-1.61733	0.262193						
C	1.939401	-1.71782	0.08252						
C	0.016544	0.037218	0.260263						
C	-0.95587	-1.02388	-0.29694						
C	-2.43668	-0.97544	0.110007						
C	-3.2728	0.202464	-0.42024						
C	-4.75541	-0.10173	-0.33158						
O	-0.35955	1.343225	-0.23898						
C	5.13156	0.29049	0.560367						
C	-5.47778	-0.30257	-1.4373						
C	-5.34894	-0.18281	1.049893						
H	1.418529	-0.18727	-1.33008						
C	2.580848	2.057378	-0.26222						
O	4.042203	-0.19591	-1.54603						
C	-0.07937	0.164848	1.776941						
O	-3.04249	1.408999	0.297812						
H	2.577547	0.649558	1.353695						
H	4.019923	-2.33332	-0.35031						
H	3.745124	-1.79417	1.304707						
H	1.479315	-2.09888	0.99835						
H	1.669357	-2.40994	-0.71719						
H	-0.58186	-2.00767	-0.0005						
H	-0.88693	-0.99738	-1.39328						
H	-2.53689	-1.02034	1.198828						
H	-2.89183	-1.89386	-0.27685						
H	-3.02057	0.348515	-1.48147						
H	-0.22045	1.354549	-1.1964						
H	5.955403	-0.38887	0.325902						
H	5.415099	1.291878	0.220402						
H	5.008098	0.326119	1.646077						
H	-6.53584	-0.54743	-1.38817						
H	-5.03493	-0.22983	-2.42764						
H	-4.88684	-0.98877	1.629144						
H	-5.17036	0.746794	1.595772						
H	-6.4244	-0.36677	1.008629						
H	3.49841	2.587235	0.013078						
H	1.737434	2.61325	0.148514						

H	2.484542	2.084412	-1.35339						
H	4.263376	0.703598	-1.82189						
H	0.589098	0.947221	2.140939						
H	-1.09439	0.433999	2.075793						
H	0.181469	-0.77528	2.268089						
H	-2.09468	1.61383	0.182043						

Table S 25. Energy analysis for 2*S*, 3*R*, 6*R*, 7*S*, 10*S*-4 in the Chloroform at MPW1PW91/6-31G+d, p level

Conf.	Steric Energy (kJ/mol)	Relative Energy (kJ/mol)	Distribution (%)
4d-1	-813.599408	0.000497	8.42%
4d-2	-813.599905	0	14.25%
4d-3	-813.599108	0.000797	6.13%
4d-4	-813.598916	0.000989	5.00%
4d-5	-813.599032	0.000873	5.65%
4d-6	-813.598156	0.001749	2.24%
4d-7	-813.597454	0.002451	1.06%
4d-8	-813.598659	0.001246	3.81%
4d-9	-813.597978	0.001927	1.85%
4d-10	-813.597978	0.001927	1.85%
4d-11	-813.597806	0.002099	1.54%
4d-12	-813.598986	0.000919	5.38%
4d-13	-813.598605	0.0013	3.60%
4d-14	-813.599069	0.000836	5.88%
4d-15	-813.599103	0.000802	6.09%
4d-16	-813.599265	0.00064	7.23%
4d-17	-813.59871	0.001195	4.02%
4d-18	-813.598022	0.001883	1.94%
4d-19	-813.599892	0.000013	14.05%

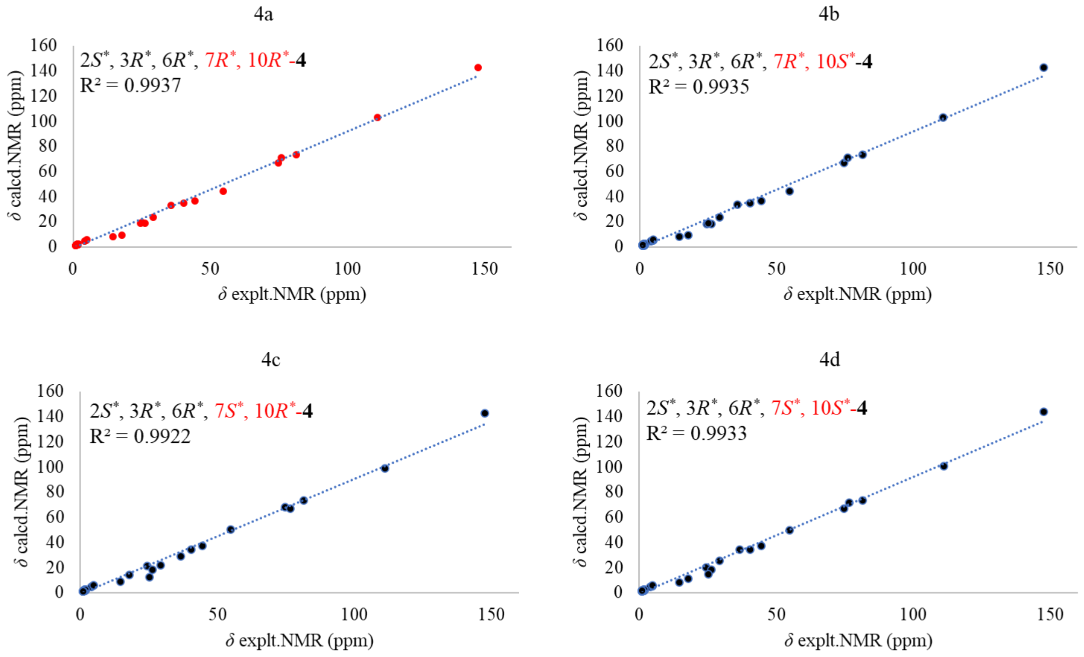

Figure S 87. Correlation between the calculated ^{13}C NMR data for 2*S*, 3*R*, 6*R*, 7*R*, 10*R*-4 (a); 2*S*, 3*R*, 6*R*, 7*R*, 10*S*-4 (b); 2*S*, 3*R*, 6*R*, 7*S*, 10*R*-4 (c) and 2*S*, 3*R*, 6*R*, 7*S*, 10*S*-4 (d) and experimental ^{13}C NMR data of 4

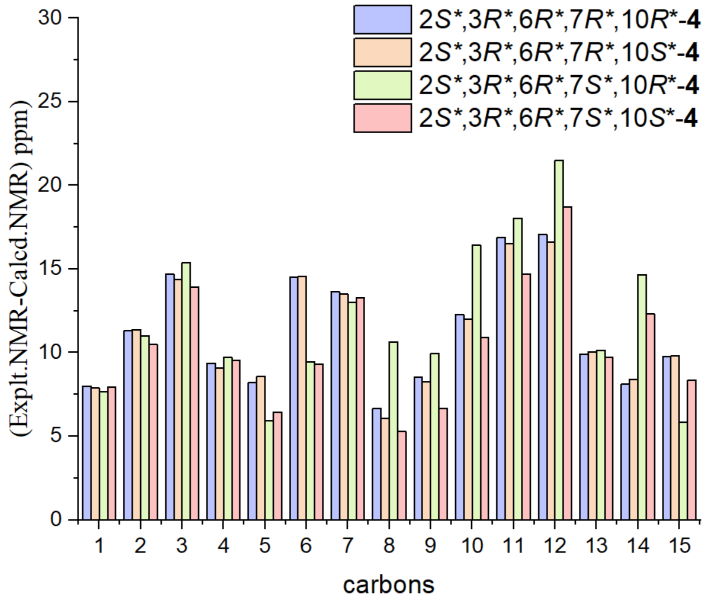

Figure S 88. Differences between experimental NMR chemical shifts of 3 and theoretical NMR chemical shifts for 2*S*, 3*R*, 6*R*, 7*R*, 10*R*-4 (purple bar); 2*S*, 3*R*, 6*R*, 7*R*, 10*S*-4 (yellow bar); 2*S*, 3*R*, 6*R*, 7*S*, 10*R*-4 (green bar) and 2*S*, 3*R*, 6*R*, 7*S*, 10*R*-4 (pink bar).

Table S 26. Experimental chemical shifts of 4, the calculated shielding tensors for 2*S*, 3*R*, 6*R*, 7*R*, 10*R*-4 (isomer 1); 2*S*, 3*R*, 6*R*, 7*R*, 10*S*-4 (isomer 2); 2*S*, 3*R*, 6*R*, 7*S*, 10*R*-4 (isomer 3) and 2*S*, 3*R*, 6*R*, 7*S*, 10*S*-4 (isomer 4), as well as their DP4+ probability

	A	B	C	D	E	F	G	H
1	Functional		Solvent?		Basis Set		Type of Data	
2	mPW1PW91		PCM		6-31+G(d,p)		Shielding Tensors	
3								
12			DP4+	100.00%	0.00%	0.00%	0.00%	-
14	Nuclei	sp2?	xperimenta	Isomer 1	Isomer 2	Isomer 3	Isomer 4	Isomer 5
15	C		54.8	144.2	144.3	138.2	138.9	
16	C		44.5	151.8	152	151.2	151.3	
17	C		81.5	115.4	115.2	115.5	115.1	
18	C		40.5	154.1	153.8	154.2	154.5	
19	C		24.5	170.1	170.6	167.6	168.4	
20	C		74.7	121.6	121.6	120.4	121.7	
21	C		36.6	155.4	154.8	159.4	154.2	
22	C		29.4	165.2	164.9	166.5	163.4	
23	C		76.7	118	117.8	121.9	117.0	
24	C	x	147.7	46.1	45.9	46.0	44.6	
25	C		26.2	170.1	170.3	170.3	170.1	
26	C	x	111.2	85.8	85.5	89.71	88.22	
27	C		17.8	179.1	179.1	174.77	177.71	
28	H		1.87	29.8	29.85	30.06	29.96	
29	C		14.7	180.5	180.4	180.15	180.60	
30	C		25.2	169.3	169.6	176.29	174.01	
31	H		1.62	29.64	29.62	29.78	29.74	
32	H		1.57	30.14	30.1	30.11	30.12	
33	H		1.69	29.99	30.01	30.07	29.99	
34	H		1.56	30.05	29.96	30.34	30.03	
35	H		1.86	29.7	29.7	29.60	29.72	
36	H		1.48	30.23	29.85	30.10	29.92	
37	H		1.6	30.41	30.65	30.30	30.09	
38	H		1.65	30.12	30.32	29.64	29.63	
39	H		1.65	30.13	29.99	29.88	30.21	
40	H		4.05	27.61	27.59	27.37	27.45	
41	H		1.26	30.48	30.48	30.44	30.44	
42	H		1.26	30.28	30.25	30.32	30.3	
43	H		1.26	30.7	30.71	30.76	30.76	
44	H	x	4.84	26.46	26.47	26.34	26.52	
45	H	x	4.95	26.38	26.37	26.24	26.31	
46	H		1.74	29.55	29.9	29.91	29.8	
47	H		1.74	29.88	29.54	30.05	29.51	
48	H		1.74	30.03	30.03	29.78	29.98	
49	H		1.05	30.6	30.62	30.68	30.67	
50	H		1.05	30.59	30.61	30.53	30.37	
51	H		1.05	30.7	30.68	30.82	30.81	
52	H		1.16	30.34	30.4	30.48	30.4	
53	H		1.16	30.78	30.83	30.29	30.16	
54	H		1.16	30.31	30.28	30.87	30.88	

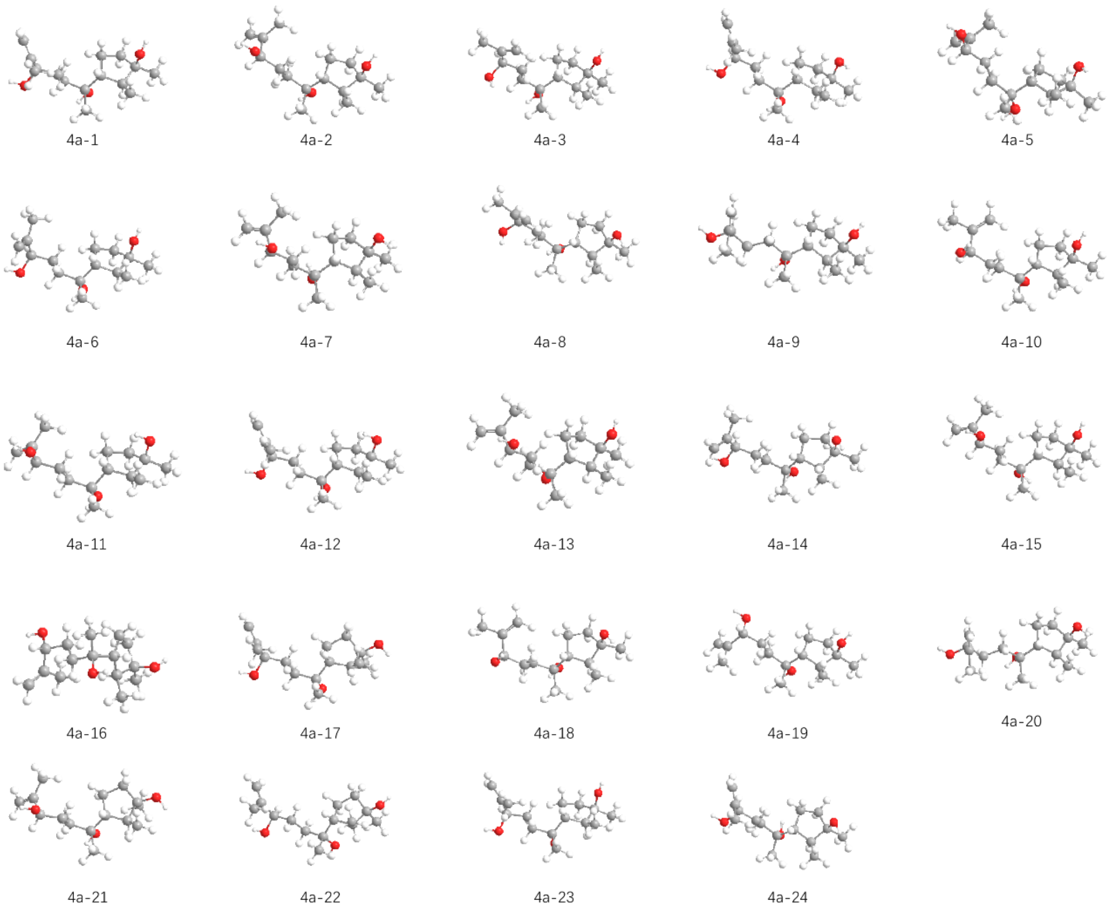

Figure S 89. Most stable conformers of 2*S*, 3*R*, 6*R*, 7*R*, 10*R*-4 at the CAM-B3LYP/DGDZVP level

Table S 27. Optimized Z-Matrixes of 2*S*, 3*R*, 6*R*, 7*R*, 10*R*-4 conformations in the methanol at CAM-B3LYP/DGDZVP level

	4a-1			4a-2			4a-3		
C	-1.0965	0.005629	0.037523	0.985579	0.200464	0.200464	-1.12765	-0.00125	0.048889
C	-2.5077	0.172427	0.671331	2.500823	0.540954	0.540954	-2.55545	0.131403	0.652541
C	-3.44381	-0.72813	-0.15936	3.240532	-0.79996	-0.79996	-3.473	-0.7027	-0.26412
C	-2.8367	-0.63955	-1.55969	2.328456	-1.55736	-1.55736	-2.8233	-0.52669	-1.63701
C	-1.33041	-0.72455	-1.31255	0.919295	-1.25261	-1.25261	-1.32579	-0.64072	-1.35163
C	-0.30857	1.323537	-0.11276	0.165288	1.2043	1.2043	-0.33085	1.319333	0.00843
C	1.096877	1.094461	-0.70613	-1.33184	0.836426	0.836426	1.087483	1.12396	-0.56653
C	1.996225	0.11588	0.048842	-2.04279	0.756161	0.756161	1.944838	0.053314	0.108612
C	3.410587	0.073378	-0.52313	-3.52884	0.440973	0.440973	3.390955	0.087167	-0.38254
C	4.247521	-1.04732	0.065262	-3.802	-0.87611	-0.87611	4.279225	-1.04509	0.110855
O	-1.05325	2.131255	-1.04248	0.674387	1.113006	1.113006	-1.05034	2.196059	-0.87761
C	-4.91046	-0.3156	-0.09758	4.673109	-0.64916	-0.64916	-4.93723	-0.27958	-0.21786
C	4.796673	-1.96717	-0.73436	-4.60375	-0.91507	-0.91507	3.905457	-1.91444	1.055213
C	4.428411	-1.05593	1.560566	-3.14575	-2.10522	-2.10522	5.639177	-1.10347	-0.53647
H	-0.50473	-0.63567	0.697699	0.570328	0.227228	0.227228	-0.55651	-0.68738	0.681739
C	-2.56468	-0.11134	2.171374	2.90188	1.289506	1.289506	-2.65623	-0.25484	2.127036
O	-3.29724	-2.06785	0.344977	3.242495	-1.4585	-1.4585	-3.35324	-2.07481	0.153413
C	-0.19905	2.080006	1.21614	0.341493	2.642687	2.642687	-0.24838	1.979229	1.389841

O	4.013165	1.346296	-0.26127	-4.06778	0.42355	0.42355	4.011823	1.345813	-0.08496
H	-2.84521	1.199192	0.49202	2.770009	1.14996	1.14996	-2.88186	1.170591	0.535327
H	-3.21731	-1.42136	-2.22576	2.559929	-2.62723	-2.62723	-3.18841	-1.25932	-2.36477
H	-3.09235	0.329354	-2.00012	2.459876	-1.15206	-1.15206	-3.05936	0.471408	-2.01904
H	-0.76702	-0.28556	-2.1366	0.177491	-1.37995	-1.37995	-0.73411	-0.15573	-2.12876
H	-1.02269	-1.76967	-1.22685	0.655472	-1.93876	-1.93876	-1.02885	-1.69211	-1.32393
H	0.987965	0.753181	-1.74008	-1.43906	-0.11403	-0.11403	1.002277	0.891547	-1.63242
H	1.599423	2.067602	-0.75515	-1.84029	1.584504	1.584504	1.607832	2.086912	-0.50284
H	2.057082	0.384791	1.108353	-1.96062	1.705579	1.705579	1.934157	0.180376	1.197516
H	1.584366	-0.89676	-0.00512	-1.5873	-0.00932	-0.00932	1.530978	-0.93709	-0.10233
H	3.344862	-0.07996	-1.60882	-4.00605	1.245556	1.245556	3.38362	0.049486	-1.4778
H	-0.58385	2.970279	-1.14406	0.18636	1.745878	1.745878	-0.57429	3.03688	-0.90951
H	-5.52126	-0.97843	-0.71955	5.138794	-1.63168	-1.63168	-5.53541	-0.893	-0.89985
H	-5.28681	-0.37303	0.927517	5.276061	-0.08207	-0.08207	-5.34417	-0.4003	0.789897
H	-5.04486	0.70683	-0.46074	4.698478	-0.13306	-0.13306	-5.0516	0.765999	-0.51617
H	5.403745	-2.77743	-0.33845	-4.82586	-1.84741	-1.84741	4.578923	-2.69967	1.388862
H	4.651568	-1.94154	-1.81175	-5.06006	-0.01221	-0.01221	2.927047	-1.88431	1.524159
H	4.741232	-0.07054	1.916685	-3.30688	-2.16336	-2.16336	5.541595	-1.2649	-1.61524
H	3.487162	-1.29646	2.064902	-2.06265	-2.07868	-2.07868	6.171384	-0.15792	-0.40198
H	5.173994	-1.79201	1.866979	-3.53349	-3.01512	-3.01512	6.247926	-1.90902	-0.12145
H	-3.58629	-0.02149	2.55354	3.983008	1.455494	1.455494	-3.68736	-0.18229	2.486637
H	-1.9419	0.590233	2.730731	2.41738	2.266859	2.266859	-2.04388	0.400112	2.750695
H	-2.21227	-1.12267	2.387828	2.61748	0.72291	0.72291	-2.31729	-1.28192	2.281758
H	-3.75282	-2.65829	-0.26983	3.571419	-2.3567	-2.3567	-3.80021	-2.61735	-0.50998
H	0.242032	1.461503	2.001832	0.102582	2.735238	2.735238	0.181797	1.307787	2.137342
H	0.430948	2.967727	1.09457	-0.31861	3.319773	3.319773	0.379243	2.875692	1.343223
H	-1.18312	2.412199	1.553081	1.36905	2.979635	2.979635	-1.23946	2.283241	1.732484
H	4.886229	1.34591	-0.67559	-5.01467	0.242697	0.242697	4.074471	1.411979	0.878234
	4a-4			4a-5			4a-6		
C	-1.09263	0.010507	0.038307	0.979057	0.194365	-0.14078	1.080746	-0.04449	0.061119
C	-2.50928	0.173535	0.658698	2.49196	0.535357	-0.25505	2.501386	-0.24598	0.662469
C	-3.43833	-0.73903	-0.17984	3.236215	-0.81023	-0.07053	3.415001	0.733069	-0.10159
C	-2.81631	-0.66418	-1.56897	2.337632	-1.55821	0.906454	2.795887	0.745469	-1.49932
C	-1.31056	-0.72815	-1.30973	0.919395	-1.25111	0.423546	1.290559	0.785288	-1.23437
C	-0.30868	1.330958	-0.10851	0.16256	1.208691	0.687713	0.297841	-1.35289	-0.17478
C	1.098872	1.107103	-0.69867	-1.33475	0.842703	0.734151	-1.11337	-1.09092	-0.74018
C	1.991485	0.113694	0.044858	-2.05614	0.784776	-0.61091	-2.00505	-0.15886	0.079602
C	3.412629	0.088226	-0.51111	-3.53581	0.442622	-0.46265	-3.43053	-0.07957	-0.46995
C	4.243616	-1.04884	0.053729	-3.78335	-0.89917	0.202237	-4.22431	1.026209	0.205354
O	-1.05375	2.138159	-1.03805	0.675428	1.129466	2.029702	1.038472	-2.09095	-1.16391
C	-4.90418	-0.32071	-0.1406	4.673262	-0.65263	0.414264	4.888599	0.341813	-0.08536
C	4.805893	-1.94204	-0.76674	-4.57913	-0.98327	1.272911	-5.11244	0.767276	1.169635
C	4.403674	-1.10441	1.550385	-3.11024	-2.09977	-0.40906	-3.94655	2.420658	-0.29239

H	-0.50314	-0.62542	0.705846	0.555304	0.204217	-1.14932	0.495027	0.542582	0.774991
C	-2.57442	-0.10752	2.159175	2.877057	1.270901	-1.53775	2.576446	-0.08718	2.180013
O	-3.31706	-2.11438	0.225247	3.217936	-1.58332	-1.28375	3.251518	2.026213	0.508457
C	-0.20486	2.084174	1.222817	0.339194	2.641805	0.173756	0.201483	-2.19914	1.099669
O	4.010446	1.353429	-0.20449	-4.08624	0.456719	-1.78526	-4.01237	-1.36729	-0.30136
H	-2.8495	1.19935	0.479036	2.771611	1.156434	0.602967	2.848283	-1.24988	0.393756
H	-3.18532	-1.46237	-2.21886	2.571661	-2.62586	0.933129	3.06389	-0.18064	-2.01754
H	-3.07946	0.29642	-2.02169	2.48907	-1.14771	1.909093	3.157173	1.584035	-2.10415
H	-0.74724	-0.28556	-2.13205	0.194964	-1.36262	1.231407	0.727584	0.396102	-2.0833
H	-0.99	-1.76884	-1.21847	0.633495	-1.94788	-0.36856	0.964876	1.815805	-1.07167
H	0.993423	0.781901	-1.73812	-1.44019	-0.11523	1.250875	-1.01441	-0.68692	-1.75229
H	1.604566	2.079318	-0.73053	-1.83671	1.582957	1.37167	-1.61513	-2.05999	-0.84345
H	2.039526	0.358212	1.110985	-1.99371	1.748874	-1.12463	-2.07337	-0.48686	1.122604
H	1.582219	-0.89814	-0.03722	-1.59458	0.043258	-1.27018	-1.57954	0.848149	0.087536
H	3.360155	-0.03299	-1.60156	-4.01998	1.222204	0.140586	-3.37513	0.156807	-1.54404
H	-0.58468	2.977333	-1.14002	0.191143	1.76918	2.569026	0.568728	-2.92068	-1.32368
H	-5.49944	-0.97814	-0.78021	5.12904	-1.63537	0.56271	5.481954	1.060946	-0.65984
H	-5.30829	-0.38023	0.875769	5.28034	-0.10414	-0.31404	5.27474	0.326383	0.937553
H	-5.02589	0.707723	-0.49038	4.708664	-0.10638	1.360384	5.035677	-0.64708	-0.52782
H	5.409234	-2.7633	-0.38819	-4.78386	-1.93423	1.758218	-5.67295	1.565892	1.647613
H	4.675687	-1.8827	-1.8447	-5.04788	-0.10038	1.700823	-5.30018	-0.2475	1.507871
H	4.713605	-0.13147	1.941623	-3.27372	-2.12705	-1.49005	-2.88901	2.681172	-0.18477
H	3.455004	-1.35832	2.033847	-2.02727	-2.06165	-0.24858	-4.18613	2.502466	-1.3576
H	5.143473	-1.85151	1.84389	-3.48259	-3.02936	0.02545	-4.53457	3.162432	0.250803
H	-3.60239	-0.05541	2.533978	3.960434	1.417526	-1.60691	3.60489	-0.19317	2.538981
H	-1.9881	0.617086	2.727345	2.415571	2.258859	-1.58666	1.972207	-0.84171	2.68818
H	-2.17363	-1.09914	2.392272	2.548115	0.717691	-2.42318	2.213472	0.897013	2.485021
H	-3.69422	-2.19978	1.110946	3.775673	-1.13432	-1.93277	3.696324	2.66985	-0.05909
H	0.235243	1.464238	2.008079	0.10509	2.722748	-0.89063	-0.23441	-1.63649	1.929077
H	0.423341	2.973696	1.105945	-0.32443	3.323871	0.716241	-0.42801	-3.07791	0.923358
H	-1.19069	2.413677	1.557293	1.365624	2.981551	0.326935	1.189237	-2.55092	1.404417
H	4.887828	1.366698	-0.60935	-5.02947	0.258842	-1.7095	-4.9064	-1.33825	-0.66757
	4a-7			4a-8			4a-9		
C	0.980011	0.202098	-0.15526	0.988723	0.209344	-0.17117	-1.26572	0.131696	0.320647
C	2.493997	0.540621	-0.24553	2.505095	0.545328	-0.24161	-2.60706	0.672266	-0.24925
C	3.236719	-0.80798	-0.07723	3.24162	-0.79954	-0.07605	-3.69679	-0.28105	0.279676
C	2.324882	-1.57628	0.871909	2.315056	-1.57591	0.861119	-2.97415	-1.62762	0.305289
C	0.916071	-1.26043	0.36586	0.915932	-1.25904	0.330964	-1.58763	-1.29253	0.85482
C	0.163013	1.201776	0.702277	0.166188	1.199207	0.692372	-0.08644	0.14048	-0.6782
C	-1.33517	0.840025	0.742342	-1.33176	0.834634	0.722205	1.213609	-0.21302	0.078721
C	-2.04792	0.780494	-0.60722	-2.02518	0.734418	-0.63494	2.407501	-0.56491	-0.80709
C	-3.53198	0.45434	-0.46546	-3.51852	0.448674	-0.50373	3.669649	-0.84945	0.001942
C	-3.7954	-0.88469	0.199009	-3.82344	-0.84914	0.221916	4.12756	0.32621	0.845319

O	0.650254	1.210346	2.056568	0.645957	1.197646	2.049429	-0.39114	-0.85885	-1.66476
C	4.666459	-0.65974	0.430996	4.666435	-0.65987	0.448652	-4.97266	-0.28528	-0.55538
C	-4.58615	-0.95909	1.274098	-4.6258	-0.85043	1.290958	4.331705	0.174209	2.157536
C	-3.14206	-2.0939	-0.41704	-3.19644	-2.10339	-0.3279	4.345973	1.63481	0.132724
H	0.56269	0.237861	-1.16608	0.581414	0.260733	-1.18534	-0.95964	0.770567	1.156472
C	2.895149	1.302328	-1.50762	2.924311	1.319693	-1.48966	-2.88362	2.142427	0.059408
O	3.234603	-1.55838	-1.30407	3.260323	-1.43371	-1.3668	-3.99986	0.130462	1.624886
C	0.342616	2.640349	0.220998	0.345782	2.642302	0.224761	0.071383	1.49875	-1.36987
O	-4.07623	0.473988	-1.79024	-4.04054	0.414601	-1.83706	4.681746	-1.21295	-0.94476
H	2.762337	1.143716	0.629182	2.762646	1.135482	0.645023	-2.5965	0.533254	-1.33639
H	2.557866	-2.64405	0.880179	2.543285	-2.6464	0.877305	-3.51184	-2.37431	0.899265
H	2.466363	-1.18988	1.88633	2.437806	-1.19596	1.881076	-2.89253	-2.00918	-0.71738
H	0.163145	-1.42262	1.142205	0.147358	-1.43153	1.089375	-0.85059	-2.03535	0.546782
H	0.657717	-1.93342	-0.45482	0.676498	-1.92202	-0.50357	-1.60818	-1.28436	1.947114
H	-1.45708	-0.12228	1.252269	-1.45804	-0.11372	1.256644	1.46464	0.638041	0.721982
H	-1.82971	1.580141	1.38194	-1.8376	1.589393	1.335278	1.02606	-1.0573	0.749402
H	-1.97196	1.741913	-1.12418	-1.91824	1.670481	-1.19165	2.188163	-1.46577	-1.38906
H	-1.59033	0.031489	-1.26064	-1.57464	-0.05246	-1.24752	2.630501	0.233154	-1.52256
H	-4.01003	1.239131	0.135762	-3.98788	1.272891	0.049674	3.475206	-1.69908	0.669938
H	0.451148	0.352073	2.454712	0.452282	0.333446	2.437333	0.222681	-0.75049	-2.40292
H	5.120586	-1.645	0.566669	5.129879	-1.64496	0.567103	-5.69585	-0.99811	-0.14537
H	5.282838	-0.09718	-0.27838	5.279611	-0.07923	-0.24584	-5.43861	0.703874	-0.55911
H	4.688544	-0.1315	1.387666	4.677859	-0.16254	1.422139	-4.76272	-0.57464	-1.58859
H	-4.80063	-1.90767	1.759857	-4.86907	-1.76798	1.820738	4.672843	0.99682	2.78099
H	-5.04087	-0.07048	1.705211	-5.06152	0.069643	1.672885	4.158835	-0.77932	2.650682
H	-3.31292	-2.11856	-1.49692	-3.36383	-2.17915	-1.4059	4.960779	1.491877	-0.76031
H	-2.05753	-2.07078	-0.26423	-2.11218	-2.0982	-0.1715	3.392295	2.060347	-0.19812
H	-3.52378	-3.01861	0.019669	-3.60177	-2.99526	0.153554	4.833954	2.36493	0.781019
H	3.979014	1.452072	-1.55888	4.005919	1.485054	-1.50848	-3.85848	2.452504	-0.32949
H	2.432425	2.290316	-1.54178	2.441429	2.298496	-1.52829	-2.12974	2.791774	-0.3906
H	2.579103	0.767005	-2.40852	2.651486	0.772764	-2.39509	-2.88021	2.318112	1.137646
H	3.80012	-1.09737	-1.93775	3.603023	-2.32912	-1.24387	-4.56094	-0.55293	2.015236
H	0.121063	2.733786	-0.84423	0.119121	2.746763	-0.83838	0.217967	2.296659	-0.63728
H	-0.32588	3.306509	0.773441	-0.31867	3.303787	0.787495	0.937766	1.500349	-2.03865
H	1.367416	2.978273	0.389127	1.371955	2.976991	0.390617	-0.80671	1.734973	-1.97414
H	-5.0222	0.287905	-1.71871	-4.99213	0.256621	-1.77276	5.484956	-1.41761	-0.4472
	4a-10			4a-11			4a-12		
C	0.995105	0.157819	-0.09713	0.986105	0.198103	-0.15298	-1.09652	0.011251	0.037807
C	2.491262	0.504055	-0.34769	2.502168	0.537774	-0.25662	-2.51257	0.174329	0.664154
C	3.26622	-0.80715	-0.1104	3.246725	-0.80856	-0.0715	-3.44443	-0.7433	-0.16692
C	2.454102	-1.47668	0.998514	2.335308	-1.56202	0.898535	-2.82935	-0.66237	-1.565
C	1.002122	-1.23755	0.584865	0.92072	-1.25239	0.402133	-1.32017	-0.72107	-1.31512
C	0.225736	1.226576	0.709829	0.166891	1.205189	0.682146	-0.31066	1.330618	-0.11275

C	-1.26997	0.876559	0.845053	-1.33024	0.837687	0.723389	1.095953	1.102742	-0.70374
C	-2.09142	0.931846	-0.44509	-2.0435	0.761751	-0.62506	1.990215	0.114542	0.044775
C	-3.52173	0.427281	-0.26513	-3.52893	0.444011	-0.4792	3.410189	0.085471	-0.51412
C	-3.68975	-1.06077	0.009275	-3.79945	-0.87704	0.217405	4.243045	-1.04614	0.058892
O	0.805093	1.214215	2.026811	0.678471	1.114786	2.023229	-1.05698	2.133376	-1.0442
C	4.734814	-0.59968	0.243635	4.673885	-0.65217	0.427629	-4.90713	-0.33308	-0.11786
C	-2.7415	-1.96561	-0.25244	-4.59857	-0.92234	1.28791	4.802976	-1.94694	-0.75483
C	-5.03976	-1.44766	0.554226	-3.14424	-2.10266	-0.36296	4.407535	-1.08747	1.555537
H	0.499631	0.080724	-1.06973	0.562986	0.220643	-1.16227	-0.50208	-0.6248	0.701966
C	2.774996	1.142361	-1.70591	2.899263	1.278868	-1.53138	-2.5765	-0.09759	2.165559
O	3.165104	-1.58205	-1.31876	3.35594	-1.53053	-1.31126	-3.41489	-2.10103	0.309004
C	0.38218	2.62823	0.111103	0.344276	2.641591	0.17834	-0.20517	2.087768	1.21582
O	-4.32869	0.784984	-1.39745	-4.06976	0.432784	-1.80559	4.007365	1.353676	-0.21964
H	2.820434	1.189004	0.441479	2.771409	1.152279	0.609098	-2.85113	1.198754	0.474588
H	2.7079	-2.53558	1.116212	2.569429	-2.62949	0.927437	-3.20044	-1.45827	-2.21631
H	2.662342	-0.97382	1.948011	2.467886	-1.1522	1.904352	-3.09026	0.300405	-2.01494
H	0.328679	-1.29683	1.44062	0.188843	-1.36878	1.201793	-0.76586	-0.26883	-2.13753
H	0.688595	-2.00198	-0.13061	0.624918	-1.94948	-0.39003	-0.97712	-1.75928	-1.24678
H	-1.35846	-0.11076	1.304006	-1.43784	-0.11386	1.251415	0.989627	0.771446	-1.7412
H	-1.71008	1.584621	1.559805	-1.83674	1.584568	1.349296	1.601053	2.074918	-0.74228
H	-2.17056	1.966405	-0.79148	-1.96359	1.713339	-1.15931	2.040467	0.366496	1.10901
H	-1.60599	0.373851	-1.2531	-1.58874	-0.00075	-1.26497	1.581658	-0.89823	-0.02952
H	-3.9761	0.976717	0.566512	-4.00591	1.245182	0.100971	3.355382	-0.04526	-1.60334
H	0.37078	1.903278	2.547497	0.192972	1.748711	2.568294	-0.59048	2.973712	-1.14819
H	5.225878	-1.56257	0.419268	5.133911	-1.63395	0.569808	-5.50658	-0.99422	-0.74983
H	5.26299	-0.09502	-0.56997	5.279724	-0.09278	-0.29066	-5.29566	-0.3929	0.90262
H	4.837335	0.001833	1.150813	4.689307	-0.12106	1.382097	-5.02903	0.691849	-0.47565
H	-2.91585	-3.02504	-0.08359	-4.819	-1.85764	1.79621	5.407631	-2.76447	-0.37037
H	-1.76102	-1.69209	-0.63081	-5.05449	-0.02198	1.692612	4.669711	-1.89765	-1.83292
H	-5.18721	-1.01084	1.547605	-3.30816	-2.15481	-1.44296	4.716967	-0.1104	1.936688
H	-5.83748	-1.06218	-0.08718	-2.06076	-2.07687	-0.20325	3.460751	-1.33839	2.04425
H	-5.1495	-2.5308	0.635073	-3.53028	-3.01531	0.09483	5.149424	-1.83058	1.853832
H	3.846347	1.316369	-1.84611	3.980531	1.441262	-1.57251	-3.60235	-0.01776	2.537712
H	2.270505	2.106065	-1.80361	2.4156	2.25607	-1.59105	-1.96556	0.613377	2.725611
H	2.429974	0.496212	-2.51661	2.614083	0.711879	-2.4219	-2.21533	-1.10288	2.399653
H	3.507078	-2.46493	-1.12415	2.469041	-1.66996	-1.6693	-2.50192	-2.41783	0.287425
H	0.090942	2.65291	-0.94178	0.105373	2.731195	-0.88425	0.236125	1.470704	2.002549
H	-0.25105	3.341964	0.649782	-0.31556	3.320339	0.729246	0.423113	2.976539	1.094417
H	1.416271	2.96986	0.190612	1.371958	2.978312	0.329042	-1.19047	2.418265	1.550609
H	-4.00886	0.267641	-2.1497	-5.01658	0.251979	-1.73179	4.883845	1.36448	-0.62654
	4a-13			4a-14			4a-15		
C	-1.12514	-0.0011	0.050687	1.080369	-0.03903	0.059178	0.993128	0.212499	-0.16022
C	-2.55673	0.130591	0.643659	2.501168	-0.23701	0.659483	2.513143	0.536156	-0.24415

C	-3.47183	-0.70571	-0.285	3.421234	0.732225	-0.12328	3.235907	-0.81473	-0.06076
C	-2.8083	-0.54113	-1.64697	2.793593	0.741908	-1.51178	2.307482	-1.56275	0.896595
C	-1.31189	-0.64457	-1.34958	1.288399	0.77988	-1.24339	0.905423	-1.23921	0.380073
C	-0.33039	1.320792	0.016144	0.296132	-1.34927	-0.16115	0.160263	1.218672	0.673196
C	1.089183	1.130463	-0.55716	-1.11487	-1.09372	-0.72945	-1.33337	0.851936	0.686154
C	1.945	0.054042	0.11062	-2.00889	-0.15744	0.082908	-1.98575	0.658371	-0.6803
C	3.391782	0.091753	-0.37833	-3.43323	-0.08306	-0.47034	-3.48876	0.417544	-0.57396
C	4.278812	-1.04593	0.104578	-4.2309	1.02417	0.197952	-3.84635	-0.80409	0.252776
O	-1.05029	2.199616	-0.8672	1.036511	-2.09924	-1.14117	0.531457	1.151069	2.062369
C	-4.93264	-0.26961	-0.25688	4.889676	0.321466	-0.11621	4.667365	-0.68354	0.447961
C	3.904178	-1.92336	1.041073	-5.12254	0.767481	1.159601	-4.68465	-0.69455	1.288127
C	5.638463	-1.09995	-0.54374	-3.95312	2.417219	-0.30368	-3.22788	-2.11416	-0.15935
H	-0.55659	-0.68532	0.688079	0.495799	0.555278	0.768144	0.596764	0.245302	-1.17942
C	-2.66494	-0.26189	2.116289	2.57547	-0.054	2.174725	2.93001	1.286741	-1.5076
O	-3.3807	-2.10814	0.02346	3.293409	2.078318	0.368497	3.235041	-1.46606	-1.34262
C	-0.2518	1.973946	1.401106	0.199906	-2.1792	1.124192	0.34552	2.657352	0.180683
O	4.013038	1.346985	-0.06735	-4.01305	-1.37109	-0.29731	-3.97061	0.27664	-1.91568
H	-2.88198	1.170856	0.532984	2.843716	-1.24757	0.411	2.808612	1.14344	0.621546
H	-3.16743	-1.28609	-2.36232	3.154901	1.582203	-2.11095	2.526123	-2.63507	0.930987
H	-3.04616	0.452859	-2.037	3.060987	-0.18619	-2.02566	2.438828	-1.16467	1.908343
H	-0.7178	-0.15769	-2.12384	0.725031	0.38236	-2.08834	0.158918	-1.35373	1.167223
H	-1.00851	-1.69397	-1.31864	0.960717	1.811092	-1.09002	0.635228	-1.9224	-0.42909
H	1.006094	0.906928	-1.6251	-1.01597	-0.69713	-1.74447	-1.45898	-0.05548	1.283765
H	1.60935	2.092848	-0.48439	-1.61478	-2.06444	-0.82581	-1.86008	1.643064	1.232342
H	1.932698	0.172065	1.200552	-2.07924	-0.47943	1.127639	-1.8389	1.541652	-1.30996
H	1.53107	-0.9344	-0.10931	-1.58435	0.850021	0.085647	-1.53794	-0.18752	-1.21082
H	3.385656	0.065088	-1.47391	-3.37561	0.148554	-1.54533	-3.95526	1.297187	-0.11091
H	-0.5742	3.040467	-0.89777	0.56562	-2.92987	-1.29267	1.421528	1.515051	2.158009
H	-5.51756	-0.87071	-0.95844	5.478029	1.020633	-0.71675	5.119438	-1.6725	0.576437
H	-5.36604	-0.39607	0.741166	5.297966	0.321147	0.900249	5.280901	-0.12089	-0.26088
H	-5.03114	0.782947	-0.53474	5.015961	-0.68277	-0.52897	4.694861	-0.17266	1.414213
H	4.576764	-2.71236	1.367536	-5.68614	1.567027	1.632359	-4.96505	-1.55614	1.888805
H	2.925777	-1.8964	1.510249	-5.31026	-0.24633	1.500703	-5.11311	0.264007	1.570769
H	5.540237	-1.2531	-1.62366	-2.89682	2.68047	-0.19082	-3.36129	-2.2855	-1.23113
H	6.171104	-0.15566	-0.40231	-4.18652	2.494343	-1.37061	-2.14952	-2.11368	0.033648
H	6.247091	-1.90892	-0.13524	-4.54595	3.159834	0.233095	-3.66759	-2.95087	0.386663
H	-3.70146	-0.22105	2.468311	3.606255	-0.12586	2.538441	4.012382	1.444273	-1.53573
H	-2.08265	0.407702	2.752166	1.996151	-0.81649	2.698651	2.452295	2.26732	-1.56075
H	-2.2853	-1.27501	2.282766	2.172565	0.918601	2.474674	2.647055	0.724364	-2.40001
H	-3.79143	-2.25146	0.886433	3.675776	2.110253	1.255435	3.547492	-2.37056	-1.20686
H	0.176382	1.298591	2.146334	-0.23165	-1.60449	1.947627	0.111544	2.752408	-0.88259
H	0.375659	2.870769	1.361053	-0.43313	-3.05787	0.960826	-0.30848	3.330162	0.741869
H	-1.24391	2.276346	1.742268	1.187202	-2.53097	1.430571	1.375476	2.997148	0.32422

H	4.075297	1.403064	0.896506	-4.90558	-1.34597	-0.66742	-4.92725	0.145616	-1.86856
	4a-16			4a-17			4a-18		
C	1.479777	0.977718	-0.13712	-1.09107	-0.01113	0.182805	1.000083	0.159938	-0.10025
C	1.558157	-0.34111	0.664179	-2.53904	0.154088	0.732216	2.499663	0.505573	-0.32564
C	2.399476	-1.26582	-0.23213	-3.44284	-0.70659	-0.20866	3.270044	-0.81965	-0.1067
C	1.843436	-0.96075	-1.62596	-2.47355	-1.7155	-0.82497	2.440909	-1.51292	0.967378
C	1.575098	0.552196	-1.63174	-1.2053	-0.90469	-1.06821	0.99349	-1.25048	0.550068
C	0.323741	1.941512	0.228985	-0.3292	1.304571	-0.0487	0.225275	1.214893	0.719214
C	-1.05211	1.279656	0.393696	1.050126	1.057337	-0.68825	-1.27094	0.862773	0.841757
C	-1.61953	0.539957	-0.81475	2.014384	0.180501	0.11016	-2.08337	0.918757	-0.45381
C	-3.04138	0.040389	-0.56866	3.361705	0.019958	-0.58843	-3.52113	0.433861	-0.27837
C	-3.1481	-0.95269	0.573629	4.249443	-1.01307	0.080611	-3.70951	-1.04921	0.009566
O	0.569323	2.487272	1.541742	-1.11509	2.066339	-0.98202	0.79722	1.182193	2.038935
C	2.319506	-2.74185	0.140383	-4.09571	0.17494	-1.27444	4.731908	-0.62107	0.278564
C	-3.95216	-0.70213	1.611476	4.702956	-2.06059	-0.61519	-2.77271	-1.96887	-0.24154
C	-2.32692	-2.21079	0.46621	4.58925	-0.7904	1.530988	-5.06573	-1.4132	0.554792
H	2.397556	1.536278	0.089344	-0.52418	-0.56807	0.93751	0.515258	0.105833	-1.0798
C	2.059832	-0.2063	2.097724	-2.63248	-0.25751	2.201249	2.799089	1.169343	-1.66866
O	3.759585	-0.80998	-0.13662	-4.4579	-1.44698	0.476471	3.187231	-1.66552	-1.2676
C	0.268433	3.115268	-0.75627	-0.19268	2.101293	1.253116	0.385182	2.625399	0.142208
O	-3.47664	-0.55367	-1.79672	3.993428	1.305447	-0.59349	-4.31629	0.790404	-1.41939
H	0.561991	-0.79767	0.692035	-2.84874	1.199881	0.648147	2.822825	1.177389	0.477184
H	2.529322	-1.2802	-2.41657	-2.28374	-2.50445	-0.08851	2.689936	-2.57461	1.046497
H	0.911419	-1.51916	-1.76552	-2.87661	-2.18516	-1.72656	2.645119	-1.03805	1.931482
H	0.671762	0.77907	-2.1982	-1.31295	-0.29101	-1.96708	0.314928	-1.32338	1.400873
H	2.389432	1.092524	-2.11893	-0.32453	-1.53694	-1.20794	0.677031	-1.99652	-0.18315
H	-0.99796	0.600923	1.251008	0.890518	0.612704	-1.67621	-1.3616	-0.12535	1.298401
H	-1.7517	2.071933	0.68567	1.521913	2.032494	-0.85795	-1.71698	1.569035	1.554683
H	-1.6588	1.197146	-1.68898	2.185433	0.605027	1.104512	-2.14717	1.951409	-0.80905
H	-0.99405	-0.315	-1.08797	1.593347	-0.81972	0.253734	-1.60032	0.347498	-1.25392
H	-3.67904	0.903864	-0.33665	3.182744	-0.29994	-1.62393	-3.9739	0.997184	0.544782
H	1.44855	2.889653	1.534957	-0.65112	2.89846	-1.14598	0.355542	1.858731	2.569762
H	2.879227	-3.34855	-0.57923	-4.64918	-0.43752	-1.99211	5.206947	-1.5891	0.459657
H	2.744934	-2.91581	1.131952	-4.79246	0.880643	-0.80805	5.287699	-0.11475	-0.51803
H	1.28308	-3.09101	0.139926	-3.34742	0.764099	-1.80966	4.818347	-0.01653	1.185122
H	-4.05093	-1.40094	2.438273	5.342843	-2.81289	-0.16089	-2.96154	-3.0243	-0.06348
H	-4.53296	0.215187	1.669224	4.443673	-2.20154	-1.66178	-1.78769	-1.71188	-0.61976
H	-2.45856	-2.67819	-0.5134	4.947495	0.230304	1.690756	-5.20959	-0.9653	1.543758
H	-1.25944	-1.9906	0.574096	3.705258	-0.92384	2.16257	-5.85688	-1.02323	-0.092
H	-2.59901	-2.93156	1.23937	5.356568	-1.48819	1.87144	-5.18992	-2.494	0.645373
H	1.369242	0.400826	2.685483	-3.63406	-0.09585	2.609171	3.874503	1.321958	-1.81031
H	3.041131	0.275342	2.121635	-1.93012	0.329838	2.800227	2.322939	2.148608	-1.74544
H	2.149548	-1.1851	2.578667	-2.38353	-1.31501	2.332229	2.425746	0.562899	-2.50003

H	4.275891	-1.29614	-0.79326	-5.11577	-0.81993	0.805222	3.701345	-1.25364	-1.97469
H	-0.0176	2.810401	-1.76461	0.299404	1.516777	2.034681	0.105102	2.664445	-0.91335
H	-0.45736	3.852728	-0.40318	0.400284	3.007027	1.08633	-0.25464	3.330584	0.684242
H	1.247739	3.601342	-0.82288	-1.17524	2.403526	1.622809	1.417757	2.967498	0.238027
H	-4.38511	-0.85785	-1.66738	4.820599	1.221536	-1.08616	-3.99965	0.260073	-2.16389
	4a-19			4a-20			4a-21		
C	1.114318	0.007751	-0.20441	-1.26247	0.133481	0.308695	1.022726	0.264653	-0.26198
C	2.496826	0.719848	-0.15565	-2.60868	0.655644	-0.26436	2.548713	0.576999	-0.28841
C	3.542224	-0.40893	-0.0523	-3.69541	-0.29244	0.299318	3.258798	-0.79285	-0.04471
C	2.806984	-1.47258	0.763812	-2.9693	-1.6305	0.356245	2.218471	-1.82494	-0.47927
C	1.392576	-1.46922	0.183737	-1.57424	-1.28048	0.875687	0.898381	-1.2465	0.019384
C	0.030277	0.672771	0.6689	-0.08825	0.127932	-0.69605	0.195313	1.155022	0.679885
C	-1.32632	-0.05415	0.568928	1.21698	-0.20335	0.062316	-1.28134	0.717109	0.722791
C	-1.89454	-0.21593	-0.84021	2.416226	-0.54454	-0.82042	-2.02043	0.711166	-0.61446
C	-3.31621	-0.78656	-0.86612	3.673424	-0.83784	-0.0066	-3.51964	0.473779	-0.45081
C	-4.31348	0.080697	-0.11743	4.120857	0.324499	0.860676	-3.85944	-0.8302	0.24635
O	0.489483	0.565755	2.028667	-0.39268	-0.89422	-1.65923	0.744347	0.973026	1.9964
C	4.864942	0.032307	0.564976	-4.97446	-0.32262	-0.53011	3.628113	-0.94934	1.431149
C	-4.97672	-0.35384	0.957846	4.306962	0.151796	2.172972	-4.60879	-0.82625	1.353143
C	-4.49887	1.469258	-0.67252	4.350065	1.644015	0.172101	-3.32795	-2.09575	-0.37261
H	0.749559	0.048217	-1.23523	-0.9551	0.791028	1.129451	0.638816	0.44656	-1.27225
C	2.753086	1.682769	-1.31353	-2.88461	2.132209	0.015572	2.960654	1.245093	-1.59994
O	3.770908	-0.89745	-1.38664	-4.01128	0.035326	1.663986	4.427486	-0.99204	-0.84579
C	-0.14711	2.157066	0.327606	0.057313	1.47161	-1.41771	0.317309	2.632653	0.292136
O	-3.24434	-2.12036	-0.37727	4.693241	-1.18272	-0.9521	-4.07801	0.502477	-1.76925
H	2.56295	1.274114	0.78694	-2.60606	0.496703	-1.3488	2.803597	1.250083	0.535319
H	3.300895	-2.44878	0.710683	-3.50258	-2.35194	0.981287	2.220038	-1.87659	-1.57388
H	2.784088	-1.16559	1.814147	-2.90235	-2.03739	-0.65721	2.434764	-2.82506	-0.09366
H	0.66836	-1.86452	0.896751	-0.84068	-2.02738	0.568977	0.781851	-1.43021	1.091085
H	1.350768	-2.10074	-0.70719	-1.57586	-1.25221	1.967753	0.035765	-1.6906	-0.48402
H	-1.23624	-1.03842	1.034464	1.45714	0.654752	0.70038	-1.32429	-0.27899	1.174577
H	-2.04314	0.505984	1.183194	1.03884	-1.04631	0.737307	-1.8072	1.387476	1.416209
H	-1.90096	0.740337	-1.37146	2.201938	-1.43915	-1.41386	-1.90216	1.670281	-1.12756
H	-1.26899	-0.8948	-1.42709	2.642403	0.262033	-1.52507	-1.61291	-0.05637	-1.28061
H	-3.63594	-0.80614	-1.9189	3.476604	-1.69902	0.645701	-3.93977	1.299089	0.139426
H	-0.17563	0.978118	2.596307	0.209435	-0.79195	-2.40784	0.247192	1.53826	2.603046
H	5.557709	-0.81322	0.631378	-5.67772	-1.04584	-0.10826	4.034324	-1.94721	1.620173
H	5.337131	0.80897	-0.04291	-5.46399	0.6572	-0.54318	4.382902	-0.2067	1.713302
H	4.71368	0.424567	1.574205	-4.76259	-0.60646	-1.56421	2.759487	-0.7892	2.073991
H	-5.69844	0.280401	1.465266	4.638935	0.964567	2.814026	-4.87373	-1.74747	1.865871
H	-4.80945	-1.34375	1.371193	4.127587	-0.80936	2.648674	-4.97684	0.101419	1.7847
H	-3.6106	2.082522	-0.48649	4.985379	1.515948	-0.70869	-3.53779	-2.12097	-1.44539
H	-4.6495	1.445676	-1.75656	3.402796	2.071423	-0.17418	-2.24076	-2.1572	-0.25616

H	-5.35403	1.969741	-0.21494	4.820873	2.36622	0.841641	-3.76569	-2.98119	0.091981
H	3.754294	2.120521	-1.25149	-3.87592	2.431226	-0.34184	4.020612	1.513185	-1.60799
H	2.034289	2.505156	-1.31079	-2.15707	2.777324	-0.4802	2.383081	2.162239	-1.74992
H	2.670428	1.165978	-2.27254	-2.82664	2.34691	1.087404	2.774813	0.587145	-2.45438
H	4.298565	-1.70374	-1.31121	-4.46605	0.888061	1.669159	5.114899	-0.39249	-0.52616
H	-0.37039	2.308362	-0.73166	0.206849	2.285632	-0.70361	0.009441	2.807276	-0.74179
H	-0.97285	2.581757	0.908842	0.917583	1.46193	-2.09406	-0.31254	3.251108	0.940679
H	0.756118	2.720237	0.571014	-0.82688	1.69055	-2.01964	1.349485	2.973365	0.40018
H	-4.10903	-2.53217	-0.50582	5.492362	-1.39811	-0.45233	-5.03325	0.381144	-1.68362
	4a-22			4a-23			4a-24		
C	-1.09218	-0.01663	0.192456	-1.08084	0.003525	0.061047	-1.07713	0.008038	0.062039
C	-2.54016	0.15012	0.743809	-2.50078	0.182295	0.669321	-2.50198	0.182744	0.657729
C	-3.44331	-0.6872	-0.20467	-3.43024	-0.7313	-0.15534	-3.42485	-0.74186	-0.17501
C	-2.48099	-1.71564	-0.81304	-2.79551	-0.6862	-1.54635	-2.776	-0.70963	-1.55413
C	-1.20333	-0.91749	-1.05439	-1.29686	-0.78508	-1.25926	-1.27779	-0.78788	-1.25637
C	-0.33183	1.299134	-0.04291	-0.30968	1.333589	-0.13365	-0.31016	1.340883	-0.1294
C	1.046301	1.052499	-0.6851	1.099253	1.109001	-0.71996	1.101191	1.121741	-0.71198
C	2.015086	0.180085	0.112598	1.985015	0.112655	0.02796	1.981978	0.114776	0.027636
C	3.358654	0.017415	-0.59278	3.415281	0.099593	-0.50449	3.416614	0.11236	-0.49321
C	4.251947	-1.01049	0.076708	4.241252	-1.04013	0.062505	4.236489	-1.04208	0.052319
O	-1.11971	2.059317	-0.97551	-1.0272	2.203957	-1.02816	-1.02864	2.209911	-1.02412
C	-4.06688	0.203558	-1.28122	-4.89289	-0.30162	-0.1333	-4.88621	-0.3068	-0.17464
C	4.702773	-2.06127	-0.61594	4.823383	-1.92083	-0.75761	4.825054	-1.90254	-0.78451
C	4.600547	-0.77891	1.523588	4.373426	-1.11243	1.561161	4.354778	-1.15234	1.549753
H	-0.52441	-0.56888	0.949704	-0.48778	-0.60632	0.749575	-0.48626	-0.59652	0.757172
C	-2.63335	-0.27786	2.208892	-2.57893	-0.06661	2.17427	-2.58762	-0.06548	2.162637
O	-4.48978	-1.31153	0.548421	-3.30714	-2.05853	0.384567	-3.32613	-2.10764	0.264641
C	-0.19333	2.096577	1.258283	-0.21138	2.124059	1.170361	-0.21812	2.128782	1.176727
O	3.98842	1.303757	-0.60889	4.001701	1.36379	-0.17396	4.001456	1.367471	-0.12693
H	-2.84568	1.197751	0.673796	-2.83215	1.205962	0.461778	-2.83577	1.205847	0.45127
H	-2.30208	-2.50211	-0.07124	-3.16951	-1.48162	-2.19908	-3.13955	-1.52044	-2.19067
H	-2.88214	-2.18695	-1.7159	-3.03822	0.271437	-2.01937	-3.02548	0.239959	-2.03851
H	-1.29923	-0.30962	-1.95837	-0.69457	-0.42206	-2.09602	-0.67586	-0.41949	-2.09117
H	-0.32599	-1.55698	-1.18254	-1.01815	-1.83082	-1.11199	-0.98591	-1.82938	-1.10556
H	0.884866	0.604766	-1.67136	1.009234	0.775143	-1.76065	1.015346	0.800478	-1.75692
H	1.515612	2.028119	-0.85896	1.596976	2.082689	-0.75508	1.600532	2.095007	-0.73352
H	2.191064	0.609635	1.103917	2.014224	0.349353	1.096556	2.002418	0.334164	1.100207
H	1.595576	-0.81975	0.263255	1.582569	-0.90064	-0.06793	1.581176	-0.89716	-0.08784
H	3.174069	-0.30857	-1.6254	3.382284	-0.01029	-1.59696	3.392623	0.029342	-1.58825
H	-0.65671	2.891689	-1.14103	-1.01845	1.8052	-1.90892	-1.02036	1.810342	-1.90453
H	-4.62804	-0.39693	-2.00618	-5.49945	-0.97409	-0.7489	-5.47727	-0.97492	-0.80693
H	-4.75194	0.918462	-0.8165	-5.28842	-0.32754	0.885741	-5.30834	-0.33444	0.835671
H	-3.30273	0.7692	-1.819	-5.00889	0.712104	-0.52585	-4.99004	0.713118	-0.55379

H	5.34666	-2.80999	-0.1614	5.423976	-2.74331	-0.37734	5.421313	-2.73513	-0.41977
H	4.437257	-2.20851	-1.6601	4.713187	-1.84946	-1.83708	4.724657	-1.80379	-1.86277
H	4.95808	0.24328	1.675201	4.670216	-0.14246	1.969565	4.65237	-0.19427	1.984781
H	3.720798	-0.91013	2.161515	3.417313	-1.37738	2.02358	3.393222	-1.42397	1.996781
H	5.371182	-1.47346	1.863168	5.111867	-1.85882	1.859836	5.086999	-1.90954	1.836299
H	-3.63886	-0.12968	2.608594	-3.60494	0.036874	2.540722	-3.61953	0.002631	2.523718
H	-1.93452	0.309715	2.812216	-1.95961	0.64581	2.723879	-2.00213	0.667833	2.720605
H	-2.37382	-1.33413	2.331022	-2.23396	-1.07398	2.418915	-2.19714	-1.05457	2.422072
H	-5.03702	-1.80424	-0.07755	-3.77579	-2.65766	-0.21188	-3.7211	-2.16818	1.144572
H	0.3024	1.513122	2.038326	0.231924	1.523285	1.967461	0.22458	1.527234	1.973697
H	0.397054	3.003665	1.089741	0.407087	3.013534	1.022706	0.398164	3.020398	1.033314
H	-1.17541	2.396472	1.631118	-1.19929	2.453046	1.499523	-1.20792	2.454682	1.50331
H	4.812837	1.218252	-1.10587	4.886268	1.385137	-0.56249	4.888302	1.398517	-0.50954

Table S 28. Energy analysis for 2*S*, 3*R*, 6*R*, 7*R*, 10*R*-4

Conf.	Steric Energy (kJ/mol)	Relative Energy (kJ/mol)	Distribution (%)
4a-1	-813.602588	0.001547	2.77%
4a-2	-813.604135	0	14.24%
4a-3	-813.600842	0.003293	0.44%
4a-4	-813.602682	0.001453	3.06%
4a-5	-813.603421	0.000714	6.69%
4a-6	-813.602665	0.00147	3.00%
4a-7	-813.604095	4E-05	13.65%
4a-8	-813.604076	5.9E-05	13.38%
4a-9	-813.601964	0.002171	1.43%
4a-10	-813.599841	0.004294	0.15%
4a-11	-813.603001	0.001134	4.29%
4a-12	-813.602641	0.001494	2.93%
4a-13	-813.600855	0.00328	0.44%
4a-14	-813.602651	0.001484	2.96%
4a-15	-813.603233	0.000902	5.48%
4a-16	-813.602977	0.001158	4.18%
4a-17	-813.602031	0.002104	1.54%
4a-18	-813.600405	0.00373	0.27%
4a-19	-813.602718	0.001417	3.18%
4a-20	-813.603104	0.001031	4.78%
4a-21	-813.602457	0.001678	2.41%
4a-22	-813.601284	0.002851	0.70%
4a-23	-813.603009	0.001126	4.32%
4a-24	-813.60287	0.001265	3.73%

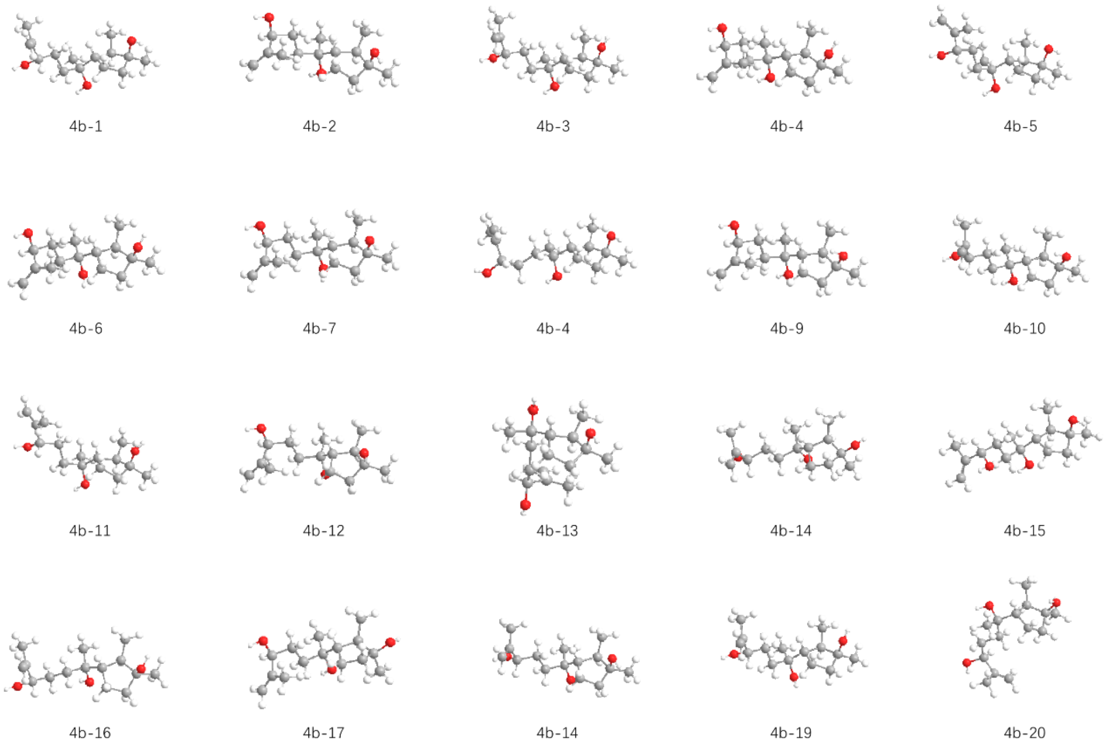

Figure S 90. Most stable conformers of 2*R*, 3*S*, 6*S*, 7*S*, 10*S*-4 at the CAM-B3LYP/DGDZVP level

Table S 29. Optimized Z-Matrixes of 2*R*, 3*S*, 6*S*, 7*S*, 10*S*-4 conformations in the methanol at CAM-B3LYP/DGDZVP level

	4b-1			4b-2			4b-3		
C	1.096501	0.005629	0.037523	-0.98558	0.200464	-0.15727	1.092633	0.010507	0.038307
C	2.507697	0.172427	0.671331	-2.50082	0.540954	-0.2531	2.509279	0.173535	0.658698
C	3.443812	-0.72813	-0.15936	-3.24053	-0.79996	-0.07156	3.438328	-0.73903	-0.17984
C	2.836701	-0.63955	-1.55969	-2.32846	-1.55736	0.893984	2.816307	-0.66418	-1.56897
C	1.330411	-0.72455	-1.31255	-0.9193	-1.25261	0.385553	1.310558	-0.72815	-1.30973
C	0.308568	1.323537	-0.11276	-0.16529	1.2043	0.680246	0.308681	1.330958	-0.10851
C	-1.09688	1.094461	-0.70613	1.331838	0.836426	0.720112	-1.09887	1.107103	-0.69867
C	-1.99623	0.11588	0.048842	2.042785	0.756161	-0.62925	-1.99149	0.113694	0.044858
C	-3.41059	0.073378	-0.52313	3.528842	0.440973	-0.4845	-3.41263	0.088226	-0.51111
C	-4.24752	-1.04732	0.065262	3.802002	-0.87611	0.218581	-4.24362	-1.04884	0.053729
O	1.053254	2.131255	-1.04248	-0.67439	1.113006	2.023062	1.053751	2.138159	-1.03805
C	4.91046	-0.3156	-0.09758	-4.67311	-0.64916	0.42886	4.904181	-0.32071	-0.1406
C	-4.79667	-1.96717	-0.73436	4.603746	-0.91507	1.28742	-4.80589	-1.94204	-0.76674
C	-4.42841	-1.05593	1.560566	3.145749	-2.10522	-0.35318	-4.40367	-1.10441	1.550385
H	0.504729	-0.63567	0.697699	-0.57033	0.227228	-1.16898	0.503139	-0.62542	0.705846
C	2.564675	-0.11134	2.171374	-2.90188	1.289506	-1.52288	2.574417	-0.10752	2.159175
O	3.297243	-2.06785	0.344977	-3.2425	-1.4585	-1.35112	3.317057	-2.11438	0.225247
C	0.199047	2.080006	1.21614	-0.34149	2.642687	0.181103	0.204856	2.084174	1.222817
O	-4.01317	1.346296	-0.26127	4.067775	0.42355	-1.81168	-4.01045	1.353429	-0.20449
H	2.845205	1.199192	0.49202	-2.77001	1.14996	0.61671	2.849495	1.19935	0.479036
H	3.217311	-1.42136	-2.22576	-2.55993	-2.62723	0.929267	3.185318	-1.46237	-2.21886

H	3.092349	0.329354	-2.00012	-2.45988	-1.15206	1.902129	3.079459	0.29642	-2.02169
H	0.767024	-0.28556	-2.1366	-0.17749	-1.37995	1.17503	0.747236	-0.28556	-2.13205
H	1.022685	-1.76967	-1.22685	-0.65547	-1.93876	-0.4235	0.989997	-1.76884	-1.21847
H	-0.98797	0.753181	-1.74008	1.439058	-0.11403	1.250134	-0.99342	0.781901	-1.73812
H	-1.59942	2.067602	-0.75515	1.840292	1.584504	1.343265	-1.60457	2.079318	-0.73053
H	-2.05708	0.384791	1.108353	1.960616	1.705579	-1.16718	-2.03953	0.358212	1.110985
H	-1.58437	-0.89676	-0.00512	1.587297	-0.00932	-1.26493	-1.58222	-0.89814	-0.03722
H	-3.34486	-0.07996	-1.60882	4.006049	1.245556	0.090875	-3.36016	-0.03299	-1.60156
H	0.583847	2.970279	-1.14406	-0.18636	1.745878	2.567181	0.584684	2.977333	-1.14002
H	5.28681	-0.37303	0.927517	-5.27606	-0.08207	-0.28566	5.308289	-0.38023	0.875769
H	5.52126	-0.97843	-0.71955	-5.13879	-1.63168	0.559754	5.49944	-0.97814	-0.78021
H	5.044864	0.70683	-0.46074	-4.69848	-0.13306	1.392335	5.025891	0.707723	-0.49038
H	-5.40375	-2.77743	-0.33845	4.825862	-1.84741	1.80043	-5.40923	-2.7633	-0.38819
H	-4.65157	-1.94154	-1.81175	5.060056	-0.01221	1.686096	-4.67569	-1.8827	-1.8447
H	-3.48716	-1.29646	2.064902	2.06265	-2.07868	-0.19096	-3.455	-1.35832	2.033847
H	-4.74123	-0.07054	1.916685	3.306876	-2.16336	-1.43331	-4.71361	-0.13147	1.941623
H	-5.17399	-1.79201	1.866979	3.533491	-3.01512	0.108725	-5.14347	-1.85151	1.84389
H	1.941904	0.590233	2.730731	-3.98301	1.455494	-1.56025	3.602393	-0.05541	2.533978
H	2.212265	-1.12267	2.387828	-2.41738	2.266859	-1.57635	1.988099	0.617086	2.727345
H	3.586293	-0.02149	2.55354	-2.61748	0.72291	-2.41256	2.173629	-1.09914	2.392272
H	3.752815	-2.65829	-0.26983	-3.57142	-2.3567	-1.21209	3.694223	-2.19978	1.110946
H	-0.24203	1.461503	2.001832	-0.10258	2.735238	-0.88129	-0.23524	1.464238	2.008079
H	1.183121	2.412199	1.553081	-1.36905	2.979635	0.332643	1.190688	2.413677	1.557293
H	-0.43095	2.967727	1.09457	0.318611	3.319773	0.734012	-0.42334	2.973696	1.105945
H	-4.88623	1.34591	-0.67559	5.014673	0.242697	-1.73847	-4.88783	1.366698	-0.60935
	4b-4			4b-5			4b-6		
C	0.010507	0.194365	-0.14078	-1.08075	-0.04449	0.061119	-0.98001	0.202098	-0.15526
C	0.173535	0.535357	-0.25505	-2.50139	-0.24598	0.662469	-2.494	0.540621	-0.24553
C	-0.73903	-0.81023	-0.07053	-3.415	0.733069	-0.10159	-3.23672	-0.80798	-0.07723
C	-0.66418	-1.55821	0.906454	-2.79589	0.745469	-1.49932	-2.32488	-1.57628	0.871909
C	-0.72815	-1.25111	0.423546	-1.29056	0.785288	-1.23437	-0.91607	-1.26043	0.36586
C	1.330958	1.208691	0.687713	-0.29784	-1.35289	-0.17478	-0.16301	1.201776	0.702277
C	1.107103	0.842703	0.734151	1.113367	-1.09092	-0.74018	1.335165	0.840025	0.742342
C	0.113694	0.784776	-0.61091	2.005052	-0.15886	0.079602	2.04792	0.780494	-0.60722
C	0.088226	0.442622	-0.46265	3.430532	-0.07957	-0.46995	3.531978	0.45434	-0.46546
C	-1.04884	-0.89917	0.202237	4.224311	1.026209	0.205354	3.795396	-0.88469	0.199009
O	2.138159	1.129466	2.029702	-1.03847	-2.09095	-1.16391	-0.65025	1.210346	2.056568
C	-0.32071	-0.65263	0.414264	-4.8886	0.341813	-0.08536	-4.66646	-0.65974	0.430996
C	-1.94204	-0.98327	1.272911	5.112444	0.767276	1.169635	4.586148	-0.95909	1.274098
C	-1.10441	-2.09977	-0.40906	3.946545	2.420658	-0.29239	3.142063	-2.0939	-0.41704
H	-0.62542	0.204217	-1.14932	-0.49503	0.542582	0.774991	-0.56269	0.237861	-1.16608
C	-0.10752	1.270901	-1.53775	-2.57645	-0.08718	2.180013	-2.89515	1.302328	-1.50762
O	-2.11438	-1.58332	-1.28375	-3.25152	2.026213	0.508457	-3.2346	-1.55838	-1.30407

C	2.084174	2.641805	0.173756	-0.20148	-2.19914	1.099669	-0.34262	2.640349	0.220998
O	1.353429	0.456719	-1.78526	4.012368	-1.36729	-0.30136	4.076227	0.473988	-1.79024
H	1.19935	1.156434	0.602967	-2.84828	-1.24988	0.393756	-2.76234	1.143716	0.629182
H	-1.46237	-1.14771	1.909093	-3.15717	1.584035	-2.10415	-2.55787	-2.64405	0.880179
H	0.29642	-2.62586	0.933129	-3.06389	-0.18064	-2.01754	-2.46636	-1.18988	1.88633
H	-0.28556	-1.36262	1.231407	-0.72758	0.396102	-2.0833	-0.16315	-1.42262	1.142205
H	-1.76884	-1.94788	-0.36856	-0.96488	1.815805	-1.07167	-0.65772	-1.93342	-0.45482
H	0.781901	-0.11523	1.250875	1.014412	-0.68692	-1.75229	1.457083	-0.12228	1.252269
H	2.079318	1.582957	1.37167	1.615125	-2.05999	-0.84345	1.829705	1.580141	1.38194
H	0.358212	1.748874	-1.12463	2.073374	-0.48686	1.122604	1.97196	1.741913	-1.12418
H	-0.89814	0.043258	-1.27018	1.579537	0.848149	0.087536	1.590329	0.031489	-1.26064
H	-0.03299	1.222204	0.140586	3.375127	0.156807	-1.54404	4.010029	1.239131	0.135762
H	2.977333	1.76918	2.569026	-0.56873	-2.92068	-1.32368	-0.45115	0.352073	2.454712
H	-0.38023	-0.10414	-0.31404	-5.27474	0.326383	0.937553	-5.28284	-0.09718	-0.27838
H	-0.97814	-1.63537	0.56271	-5.48195	1.060946	-0.65984	-5.12059	-1.645	0.566669
H	0.707723	-0.10638	1.360384	-5.03568	-0.64708	-0.52782	-4.68854	-0.1315	1.387666
H	-2.7633	-1.93423	1.758218	5.672954	1.565892	1.647613	4.800625	-1.90767	1.759857
H	-1.8827	-0.10038	1.700823	5.30018	-0.2475	1.507871	5.040868	-0.07048	1.705211
H	-1.35832	-2.06165	-0.24858	4.186125	2.502466	-1.3576	2.057525	-2.07078	-0.26423
H	-0.13147	-2.12705	-1.49005	2.88901	2.681172	-0.18477	3.312923	-2.11856	-1.49692
H	-1.85151	-3.02936	0.02545	4.534568	3.162432	0.250803	3.523779	-3.01861	0.019669
H	-0.05541	1.417526	-1.60691	-3.60489	-0.19317	2.538981	-3.97901	1.452072	-1.55888
H	0.617086	2.258859	-1.58666	-1.97221	-0.84171	2.68818	-2.43243	2.290316	-1.54178
H	-1.09914	0.717691	-2.42318	-2.21347	0.897013	2.485021	-2.5791	0.767005	-2.40852
H	-2.19978	-1.13432	-1.93277	-3.69632	2.66985	-0.05909	-3.80012	-1.09737	-1.93775
H	1.464238	2.722748	-0.89063	0.234408	-1.63649	1.929077	-0.12106	2.733786	-0.84423
H	2.413677	2.981551	0.326935	-1.18924	-2.55092	1.404417	-1.36742	2.978273	0.389127
H	2.973696	3.323871	0.716241	0.428012	-3.07791	0.923358	0.325875	3.306509	0.773441
H	1.366698	0.258842	-1.7095	4.906396	-1.33825	-0.66757	5.0222	0.287905	-1.71871
	4b-7			4b-8			4b-9		
C	-0.98872	0.209344	-0.17117	1.265716	0.131696	0.320647	-0.98611	0.198103	-0.15298
C	-2.5051	0.545328	-0.24161	2.607063	0.672266	-0.24925	-2.50217	0.537774	-0.25662
C	-3.24162	-0.79954	-0.07605	3.696791	-0.28105	0.279676	-3.24673	-0.80856	-0.0715
C	-2.31506	-1.57591	0.861119	2.974146	-1.62762	0.305289	-2.33531	-1.56202	0.898535
C	-0.91593	-1.25904	0.330964	1.587632	-1.29253	0.85482	-0.92072	-1.25239	0.402133
C	-0.16619	1.199207	0.692372	0.086439	0.14048	-0.6782	-0.16689	1.205189	0.682146
C	1.331759	0.834634	0.722205	-1.21361	-0.21302	0.078721	1.330241	0.837687	0.723389
C	2.025181	0.734418	-0.63494	-2.4075	-0.56491	-0.80709	2.043501	0.761751	-0.62506
C	3.518515	0.448674	-0.50373	-3.66965	-0.84945	0.001942	3.528928	0.444011	-0.4792
C	3.823439	-0.84914	0.221916	-4.12756	0.32621	0.845319	3.799447	-0.87704	0.217405
O	-0.64596	1.197646	2.049429	0.391137	-0.85885	-1.66476	-0.67847	1.114786	2.023229
C	-4.66644	-0.65987	0.448652	4.972656	-0.28528	-0.55538	-4.67389	-0.65217	0.427629
C	4.625803	-0.85043	1.290958	-4.33171	0.174209	2.157536	4.598567	-0.92234	1.28791

C	3.196439	-2.10339	-0.3279	-4.34597	1.63481	0.132724	3.144238	-2.10266	-0.36296
H	-0.58141	0.260733	-1.18534	0.959636	0.770567	1.156472	-0.56299	0.220643	-1.16227
C	-2.92431	1.319693	-1.48966	2.883624	2.142427	0.059408	-2.89926	1.278868	-1.53138
O	-3.26032	-1.43371	-1.3668	3.999857	0.130462	1.624886	-3.35594	-1.53053	-1.31126
C	-0.34578	2.642302	0.224761	-0.07138	1.49875	-1.36987	-0.34428	2.641591	0.17834
O	4.040537	0.414601	-1.83706	-4.68175	-1.21295	-0.94476	4.069764	0.432784	-1.80559
H	-2.76265	1.135482	0.645023	2.596498	0.533254	-1.33639	-2.77141	1.152279	0.609098
H	-2.54329	-2.6464	0.877305	3.51184	-2.37431	0.899265	-2.56943	-2.62949	0.927437
H	-2.43781	-1.19596	1.881076	2.892529	-2.00918	-0.71738	-2.46789	-1.1522	1.904352
H	-0.14736	-1.43153	1.089375	0.850588	-2.03535	0.546782	-0.18884	-1.36878	1.201793
H	-0.6765	-1.92202	-0.50357	1.608184	-1.28436	1.947114	-0.62492	-1.94948	-0.39003
H	1.458036	-0.11372	1.256644	-1.46464	0.638041	0.721982	1.437841	-0.11386	1.251415
H	1.837595	1.589393	1.335278	-1.02606	-1.0573	0.749402	1.836736	1.584568	1.349296
H	1.918241	1.670481	-1.19165	-2.18816	-1.46577	-1.38906	1.963594	1.713339	-1.15931
H	1.574639	-0.05246	-1.24752	-2.6305	0.233154	-1.52256	1.588744	-0.00075	-1.26497
H	3.987884	1.272891	0.049674	-3.47521	-1.69908	0.669938	4.00591	1.245182	0.100971
H	-0.45228	0.333446	2.437333	-0.22268	-0.75049	-2.40292	-0.19297	1.748711	2.568294
H	-5.27961	-0.07923	-0.24584	5.438606	0.703874	-0.55911	-5.27972	-0.09278	-0.29066
H	-5.12988	-1.64496	0.567103	5.69585	-0.99811	-0.14537	-5.13391	-1.63395	0.569808
H	-4.67786	-0.16254	1.422139	4.762724	-0.57464	-1.58859	-4.68931	-0.12106	1.382097
H	4.869068	-1.76798	1.820738	-4.67284	0.99682	2.78099	4.819	-1.85764	1.79621
H	5.061521	0.069643	1.672885	-4.15884	-0.77932	2.650682	5.054488	-0.02198	1.692612
H	2.112177	-2.0982	-0.1715	-3.3923	2.060347	-0.19812	2.060759	-2.07687	-0.20325
H	3.363831	-2.17915	-1.4059	-4.96078	1.491877	-0.76031	3.308161	-2.15481	-1.44296
H	3.601767	-2.99526	0.153554	-4.83395	2.36493	0.781018	3.530283	-3.01531	0.09483
H	-4.00592	1.485054	-1.50848	3.858478	2.452504	-0.32949	-3.98053	1.441262	-1.57251
H	-2.44143	2.298496	-1.52829	2.129742	2.791774	-0.3906	-2.4156	2.25607	-1.59105
H	-2.65149	0.772764	-2.39509	2.880211	2.318112	1.137646	-2.61408	0.711879	-2.4219
H	-3.60302	-2.32912	-1.24387	4.560944	-0.55293	2.015236	-2.46904	-1.66996	-1.6693
H	-0.11912	2.746763	-0.83838	-0.21797	2.296659	-0.63728	-0.10537	2.731195	-0.88425
H	-1.37196	2.976991	0.390617	0.806714	1.734973	-1.97414	-1.37196	2.978312	0.329042
H	0.318666	3.303787	0.787495	-0.93777	1.500349	-2.03865	0.315559	3.320339	0.729246
H	4.992126	0.256621	-1.77276	-5.48496	-1.41761	-0.4472	5.016583	0.251979	-1.73179
	4b-10			44b-11			4b-12		
C	1.096518	0.011251	0.037807	-1.08037	-0.03903	0.059178	-0.99313	0.212499	-0.16022
C	2.512574	0.174329	0.664154	-2.50117	-0.23701	0.659483	-2.51314	0.536156	-0.24415
C	3.444432	-0.7433	-0.16692	-3.42123	0.732225	-0.12328	-3.23591	-0.81473	-0.06076
C	2.829352	-0.66237	-1.565	-2.79359	0.741908	-1.51178	-2.30748	-1.56275	0.896595
C	1.320165	-0.72107	-1.31512	-1.2884	0.77988	-1.24339	-0.90542	-1.23921	0.380073
C	0.310656	1.330618	-0.11275	-0.29613	-1.34927	-0.16115	-0.16026	1.218672	0.673196
C	-1.09595	1.102742	-0.70374	1.11487	-1.09372	-0.72945	1.333372	0.851936	0.686154
C	-1.99022	0.114542	0.044775	2.008893	-0.15744	0.082908	1.985745	0.658371	-0.6803
C	-3.41019	0.085471	-0.51412	3.433234	-0.08306	-0.47034	3.488762	0.417544	-0.57396

C	-4.24305	-1.04614	0.058892	4.230903	1.02417	0.197952	3.846353	-0.80409	0.252776
O	1.056984	2.133376	-1.0442	-1.03651	-2.09924	-1.14117	-0.53146	1.151069	2.062369
C	4.907128	-0.33308	-0.11786	-4.88968	0.321466	-0.11621	-4.66737	-0.68354	0.447961
C	-4.80298	-1.94694	-0.75483	5.122542	0.767481	1.159601	4.684645	-0.69455	1.288127
C	-4.40754	-1.08747	1.555537	3.953116	2.417219	-0.30368	3.227877	-2.11416	-0.15935
H	0.50208	-0.6248	0.701966	-0.4958	0.555278	0.768144	-0.59676	0.245302	-1.17942
C	2.576503	-0.09759	2.165559	-2.57547	-0.054	2.174725	-2.93001	1.286741	-1.5076
O	3.414885	-2.10103	0.309004	-3.29341	2.078318	0.368497	-3.23504	-1.46606	-1.34262
C	0.205165	2.087768	1.21582	-0.19991	-2.1792	1.124192	-0.34552	2.657352	0.180683
O	-4.00737	1.353676	-0.21964	4.013047	-1.37109	-0.29731	3.970612	0.27664	-1.91568
H	2.851127	1.198754	0.474588	-2.84372	-1.24757	0.411	-2.80861	1.14344	0.621546
H	3.200436	-1.45827	-2.21631	-3.1549	1.582203	-2.11095	-2.52612	-2.63507	0.930987
H	3.090261	0.300405	-2.01494	-3.06099	-0.18619	-2.02566	-2.43883	-1.16467	1.908343
H	0.765855	-0.26883	-2.13753	-0.72503	0.38236	-2.08834	-0.15892	-1.35373	1.167223
H	0.977117	-1.75928	-1.24678	-0.96072	1.811092	-1.09002	-0.63523	-1.9224	-0.42909
H	-0.98963	0.771446	-1.7412	1.015973	-0.69713	-1.74447	1.458982	-0.05548	1.283765
H	-1.60105	2.074918	-0.74228	1.614784	-2.06444	-0.82581	1.860077	1.643064	1.232342
H	-2.04047	0.366496	1.10901	2.07924	-0.47943	1.127639	1.838898	1.541652	-1.30996
H	-1.58166	-0.89823	-0.02952	1.584353	0.850021	0.085647	1.537943	-0.18752	-1.21082
H	-3.35538	-0.04526	-1.60334	3.375609	0.148554	-1.54533	3.955259	1.297187	-0.11091
H	0.590479	2.973712	-1.14819	-0.56562	-2.92987	-1.29267	-1.42153	1.515051	2.158009
H	5.295658	-0.3929	0.90262	-5.29797	0.321147	0.900249	-5.2809	-0.12089	-0.26088
H	5.506582	-0.99422	-0.74983	-5.47803	1.020633	-0.71675	-5.11944	-1.6725	0.576437
H	5.029026	0.691849	-0.47565	-5.01596	-0.68277	-0.52897	-4.69486	-0.17266	1.414213
H	-5.40763	-2.76447	-0.37037	5.686143	1.567027	1.632359	4.965047	-1.55614	1.888805
H	-4.66971	-1.89765	-1.83292	5.310259	-0.24633	1.500703	5.113112	0.264007	1.570769
H	-3.46075	-1.33839	2.04425	4.18652	2.494343	-1.37061	2.149516	-2.11368	0.033648
H	-4.71697	-0.1104	1.936688	2.896815	2.68047	-0.19082	3.361289	-2.2855	-1.23113
H	-5.14942	-1.83058	1.853832	4.545947	3.159834	0.233095	3.667592	-2.95087	0.386663
H	3.602345	-0.01776	2.537712	-3.60626	-0.12586	2.538441	-4.01238	1.444273	-1.53573
H	1.965559	0.613377	2.725611	-1.99615	-0.81649	2.698651	-2.4523	2.26732	-1.56075
H	2.215326	-1.10288	2.399653	-2.17257	0.918601	2.474674	-2.64706	0.724364	-2.40001
H	2.501919	-2.41783	0.287425	-3.67578	2.110253	1.255435	-3.54749	-2.37056	-1.20686
H	-0.23613	1.470704	2.002549	0.231651	-1.60449	1.947627	-0.11154	2.752408	-0.88259
H	1.190468	2.418265	1.550609	-1.1872	-2.53097	1.430571	-1.37548	2.997148	0.32422
H	-0.42311	2.976539	1.094417	0.433131	-3.05787	0.960826	0.308483	3.330162	0.741869
H	-4.88385	1.36448	-0.62654	4.905578	-1.34597	-0.66742	4.927249	0.145616	-1.86856
	4b-13			4b-14			4b-15		
C	-1.47978	0.977718	-0.13712	1.091065	-0.01113	0.182805	-1.11432	-1.11432	-0.20441
C	-1.55816	-0.34111	0.664179	2.539038	0.154088	0.732216	-2.49683	-2.49683	-0.15565
C	-2.39948	-1.26582	-0.23213	3.442842	-0.70659	-0.20866	-3.54222	-3.54222	-0.0523
C	-1.84344	-0.96075	-1.62596	2.473545	-1.7155	-0.82497	-2.80698	-2.80698	0.763812
C	-1.5751	0.552196	-1.63174	1.205297	-0.90469	-1.06821	-1.39258	-1.39258	0.183737

C	-0.32374	1.941512	0.228985	0.329195	1.304571	-0.0487	-0.03028	-0.03028	0.6689
C	1.052112	1.279656	0.393696	-1.05013	1.057337	-0.68825	1.326321	1.326321	0.568928
C	1.619532	0.539957	-0.81475	-2.01438	0.180501	0.11016	1.894536	1.894536	-0.84021
C	3.04138	0.040389	-0.56866	-3.36171	0.019958	-0.58843	3.31621	3.31621	-0.86612
C	3.148102	-0.95269	0.573629	-4.24944	-1.01307	0.080611	4.313483	4.313483	-0.11743
O	-0.56932	2.487272	1.541742	1.115092	2.066339	-0.98202	-0.48948	-0.48948	2.028667
C	-2.31951	-2.74185	0.140383	4.095706	0.17494	-1.27444	-4.86494	-4.86494	0.564976
C	3.952163	-0.70213	1.611476	-4.70296	-2.06059	-0.61519	4.976719	4.976719	0.957846
C	2.326915	-2.21079	0.46621	-4.58925	-0.7904	1.530988	4.498867	4.498867	-0.67252
H	-2.39756	1.536278	0.089344	0.524181	-0.56807	0.93751	-0.74956	-0.74956	-1.23523
C	-2.05983	-0.2063	2.097724	2.63248	-0.25751	2.201249	-2.75309	-2.75309	-1.31353
O	-3.75959	-0.80998	-0.13662	4.457903	-1.44698	0.476471	-3.77091	-3.77091	-1.38664
C	-0.26843	3.115268	-0.75627	0.192681	2.101293	1.253116	0.147114	0.147114	0.327606
O	3.476637	-0.55367	-1.79672	-3.99343	1.305447	-0.59349	3.244336	3.244336	-0.37727
H	-0.56199	-0.79767	0.692035	2.84874	1.199881	0.648147	-2.56295	-2.56295	0.78694
H	-2.52932	-1.2802	-2.41657	2.28374	-2.50445	-0.08851	-3.3009	-3.3009	0.710683
H	-0.91142	-1.51916	-1.76552	2.876606	-2.18516	-1.72656	-2.78409	-2.78409	1.814147
H	-0.67176	0.77907	-2.1982	1.312948	-0.29101	-1.96708	-0.66836	-0.66836	0.896751
H	-2.38943	1.092524	-2.11893	0.324532	-1.53694	-1.20794	-1.35077	-1.35077	-0.70719
H	0.997955	0.600923	1.251008	-0.89052	0.612704	-1.67621	1.236239	1.236239	1.034464
H	1.751702	2.071933	0.68567	-1.52191	2.032494	-0.85795	2.043142	2.043142	1.183194
H	1.658804	1.197146	-1.68898	-2.18543	0.605027	1.104512	1.900961	1.900961	-1.37146
H	0.994051	-0.315	-1.08797	-1.59335	-0.81972	0.253734	1.268988	1.268988	-1.42709
H	3.679037	0.903864	-0.33665	-3.18274	-0.29994	-1.62393	3.63594	3.63594	-1.9189
H	-1.44855	2.889653	1.534957	0.651119	2.89846	-1.14598	0.175629	0.175629	2.596307
H	-2.74493	-2.91581	1.131952	4.792461	0.880643	-0.80805	-5.33713	-5.33713	-0.04291
H	-2.87923	-3.34855	-0.57923	4.649177	-0.43752	-1.99211	-5.55771	-5.55771	0.631378
H	-1.28308	-3.09101	0.139926	3.347418	0.764099	-1.80966	-4.71368	-4.71368	1.574205
H	4.050926	-1.40094	2.438273	-5.34284	-2.81289	-0.16089	5.69844	5.69844	1.465266
H	4.532963	0.215187	1.669224	-4.44367	-2.20154	-1.66178	4.80945	4.80945	1.371193
H	1.259439	-1.9906	0.574096	-3.70526	-0.92384	2.16257	4.649496	4.649496	-1.75656
H	2.458562	-2.67819	-0.5134	-4.9475	0.230304	1.690756	3.610597	3.610597	-0.48649
H	2.599008	-2.93156	1.23937	-5.35657	-1.48819	1.87144	5.354031	5.354031	-0.21494
H	-1.36924	0.400826	2.685483	3.634064	-0.09585	2.609171	-3.75429	-3.75429	-1.25149
H	-3.04113	0.275342	2.121635	1.930122	0.329838	2.800227	-2.03429	-2.03429	-1.31079
H	-2.14955	-1.1851	2.578667	2.383525	-1.31501	2.332229	-2.67043	-2.67043	-2.27254
H	-4.27589	-1.29614	-0.79326	5.11577	-0.81993	0.805222	-4.29857	-4.29857	-1.31121
H	0.017603	2.810401	-1.76461	-0.2994	1.516777	2.034681	0.370389	0.370389	-0.73166
H	-1.24774	3.601342	-0.82288	1.175235	2.403526	1.622809	-0.75612	-0.75612	0.571014
H	0.457359	3.852728	-0.40318	-0.40028	3.007027	1.08633	0.972848	0.972848	0.908842
H	4.385105	-0.85785	-1.66738	-4.8206	1.221536	-1.08616	4.109034	4.109034	-0.50582
	4b-16			4b-17			4b-18		
C	1.262466	0.133482	0.308695	-1.02273	0.264653	-0.26198	1.080837	0.003525	0.061047

C	2.608683	0.655644	-0.26436	-2.54871	0.576999	-0.28841	2.500784	0.182295	0.669321
C	3.695407	-0.29244	0.299318	-3.2588	-0.79285	-0.04471	3.430235	-0.7313	-0.15534
C	2.969297	-1.6305	0.356246	-2.21847	-1.82494	-0.47927	2.795506	-0.6862	-1.54635
C	1.574243	-1.28048	0.875688	-0.89838	-1.2465	0.019384	1.296856	-0.78508	-1.25926
C	0.088248	0.127932	-0.69605	-0.19531	1.155022	0.679885	0.309675	1.333589	-0.13365
C	-1.21698	-0.20335	0.062317	1.28134	0.717109	0.722791	-1.09925	1.109001	-0.71996
C	-2.41623	-0.54455	-0.82042	2.020434	0.711166	-0.61446	-1.98502	0.112655	0.02796
C	-3.67342	-0.83784	-0.0066	3.519639	0.473779	-0.45081	-3.41528	0.099593	-0.50449
C	-4.12086	0.324499	0.860675	3.859443	-0.8302	0.24635	-4.24125	-1.04013	0.062505
O	0.392678	-0.89422	-1.65923	-0.74435	0.973026	1.9964	1.027197	2.203957	-1.02816
C	4.974455	-0.32262	-0.53011	-3.62811	-0.94934	1.431149	4.892885	-0.30162	-0.1333
C	-4.30696	0.151797	2.172971	4.608792	-0.82625	1.353143	-4.82338	-1.92083	-0.75761
C	-4.35007	1.644015	0.1721	3.327951	-2.09575	-0.37261	-4.37343	-1.11243	1.561161
H	0.955103	0.791029	1.129451	-0.63882	0.44656	-1.27225	0.487778	-0.60632	0.749575
C	2.884614	2.132209	0.015571	-2.96065	1.245093	-1.59994	2.578934	-0.06661	2.17427
O	4.011281	0.035327	1.663986	-4.42749	-0.99204	-0.84579	3.30714	-2.05853	0.384567
C	-0.05731	1.471609	-1.41771	-0.31731	2.632653	0.292136	0.211378	2.124059	1.170361
O	-4.69324	-1.18272	-0.9521	4.078006	0.502477	-1.76925	-4.0017	1.36379	-0.17396
H	2.606062	0.496703	-1.3488	-2.8036	1.250083	0.535319	2.832148	1.205962	0.461778
H	3.502576	-2.35194	0.981288	-2.22004	-1.87659	-1.57388	3.169513	-1.48162	-2.19908
H	2.902354	-2.03739	-0.65721	-2.43476	-2.82506	-0.09366	3.038218	0.271437	-2.01937
H	0.840683	-2.02738	0.568978	-0.78185	-1.43021	1.091085	0.694565	-0.42206	-2.09602
H	1.575862	-1.25221	1.967753	-0.03577	-1.6906	-0.48402	1.018146	-1.83082	-1.11199
H	-1.45714	0.654752	0.70038	1.324285	-0.27899	1.174577	-1.00923	0.775143	-1.76065
H	-1.03884	-1.04631	0.737308	1.807204	1.387476	1.416209	-1.59698	2.082689	-0.75508
H	-2.20194	-1.43915	-1.41386	1.90216	1.670281	-1.12756	-2.01422	0.349353	1.096556
H	-2.6424	0.262032	-1.52507	1.612906	-0.05637	-1.28061	-1.58257	-0.90064	-0.06793
H	-3.4766	-1.69902	0.645702	3.939772	1.299089	0.139426	-3.38228	-0.01029	-1.59696
H	-0.20944	-0.79196	-2.40784	-0.24719	1.53826	2.603046	1.018446	1.8052	-1.90892
H	5.463993	0.6572	-0.54318	-4.3829	-0.2067	1.713302	5.288423	-0.32754	0.885741
H	5.677721	-1.04584	-0.10826	-4.03432	-1.94721	1.620173	5.49945	-0.97409	-0.7489
H	4.762585	-0.60646	-1.56421	-2.75949	-0.7892	2.073991	5.008893	0.712104	-0.52585
H	-4.63894	0.964569	2.814025	4.873732	-1.74747	1.865871	-5.42398	-2.74331	-0.37734
H	-4.12759	-0.80936	2.648675	4.976839	0.101419	1.7847	-4.71319	-1.84946	-1.83708
H	-3.4028	2.071423	-0.17418	2.240761	-2.1572	-0.25616	-3.41731	-1.37738	2.02358
H	-4.98538	1.515947	-0.70869	3.537794	-2.12097	-1.44539	-4.67022	-0.14246	1.969565
H	-4.82087	2.366221	0.841639	3.765692	-2.98119	0.091981	-5.11187	-1.85882	1.859836
H	3.875919	2.431226	-0.34184	-4.02061	1.513185	-1.60799	3.604936	0.036874	2.540722
H	2.157065	2.777324	-0.4802	-2.38308	2.162239	-1.74992	1.95961	0.64581	2.723879
H	2.826635	2.346911	1.087403	-2.77481	0.587145	-2.45438	2.233962	-1.07398	2.418915
H	4.466047	0.888062	1.669158	-5.1149	-0.39249	-0.52616	3.775787	-2.65766	-0.21188
H	-0.20685	2.285632	-0.70361	-0.00944	2.807276	-0.74179	-0.23192	1.523285	1.967461
H	0.826875	1.690549	-2.01964	-1.34949	2.973365	0.40018	1.199294	2.453046	1.499523

H	-0.91758	1.461929	-2.09407	0.312539	3.251108	0.940679	-0.40709	3.013534	1.022706
H	-5.49236	-1.39811	-0.45233	5.033254	0.381144	-1.68362	-4.88627	1.385137	-0.56249
	4b-19			4b-20					
C	1.077133	0.008038	0.062039	-1.58074	-0.89974	0.606275			
C	2.501984	0.182744	0.657729	-2.28458	-0.24743	-0.61934			
C	3.424851	-0.74186	-0.17501	-2.82109	1.122572	-0.12867			
C	2.775998	-0.70963	-1.55413	-1.81862	1.509713	0.963797			
C	1.277792	-0.78788	-1.25637	-1.5539	0.196504	1.703964			
C	0.310156	1.340883	-0.1294	-0.19586	-1.51249	0.290835			
C	-1.10119	1.121741	-0.71198	0.83853	-0.43432	-0.08511			
C	-1.98198	0.114776	0.027636	2.21685	-0.9564	-0.48923			
C	-3.41661	0.11236	-0.49321	3.14435	0.169923	-0.93956			
C	-4.23649	-1.04208	0.052319	3.357278	1.234315	0.121098			
O	1.028635	2.209911	-1.02412	-0.42817	-2.38584	-0.82729			
C	4.886214	-0.3068	-0.17464	-2.94174	2.155342	-1.23744			
C	-4.82505	-1.90254	-0.78451	3.071917	2.513361	-0.14206			
C	-4.35478	-1.15234	1.549753	3.898085	0.776861	1.450036			
H	0.486264	-0.59652	0.757172	-2.19476	-1.7397	0.949434			
C	2.587619	-0.06548	2.162637	-3.33926	-1.13754	-1.27038			
O	3.326133	-2.10764	0.264641	-4.14214	1.012013	0.429662			
C	0.218116	2.128782	1.176727	0.283831	-2.34926	1.482763			
O	-4.00146	1.367471	-0.12693	4.385754	-0.44684	-1.29762			
H	2.835772	1.205847	0.45127	-1.53161	-0.02563	-1.38119			
H	3.13955	-1.52044	-2.19067	-2.20706	2.306003	1.604084			
H	3.025475	0.239959	-2.03851	-0.89641	1.870556	0.495652			
H	0.675858	-0.41949	-2.09117	-0.62328	0.223962	2.274356			
H	0.98591	-1.82938	-1.10556	-2.3442	0.008875	2.438277			
H	-1.01535	0.800478	-1.75692	0.955194	0.249825	0.760966			
H	-1.60053	2.095007	-0.73352	0.442368	0.163221	-0.91277			
H	-2.00242	0.334164	1.100207	2.136803	-1.65626	-1.32791			
H	-1.58118	-0.89716	-0.08784	2.696315	-1.4938	0.334541			
H	-3.39262	0.029342	-1.58825	2.707157	0.64901	-1.82563			
H	1.02036	1.810342	-1.90453	0.3623	-2.92612	-0.95756			
H	5.308343	-0.33444	0.835671	-3.63976	1.816984	-2.00836			
H	5.47727	-0.97492	-0.80693	-3.30945	3.103204	-0.83475			
H	4.990038	0.713118	-0.55379	-1.96937	2.331316	-1.70352			
H	-5.42131	-2.73513	-0.41977	3.220398	3.29481	0.598876			
H	-4.72466	-1.80379	-1.86277	2.675159	2.818207	-1.10742			
H	-3.39322	-1.42397	1.996781	3.160202	0.165629	1.981047			
H	-4.65237	-0.19427	1.984781	4.788499	0.157141	1.31342			
H	-5.087	-1.90954	1.836299	4.155391	1.62443	2.08784			
H	3.619533	0.002631	2.523718	-3.83569	-0.62389	-2.09946			
H	2.002132	0.667833	2.720605	-2.86981	-2.04357	-1.65895			

H	2.197137	-1.05457	2.422072	-4.11014	-1.43628	-0.55383			
H	3.721099	-2.16818	1.144572	-4.13494	0.33379	1.118099			
H	-0.22458	1.527234	1.973697	0.517009	-1.72412	2.348255			
H	1.207918	2.454682	1.50331	-0.48787	-3.06776	1.771079			
H	-0.39816	3.020398	1.033314	1.18808	-2.91224	1.232482			
H	-4.8883	1.398517	-0.50954	4.975601	0.249341	-1.61701			

Table S 30. Energy analysis for 2*R*, 3*S*, 6*S*, 7*S*, 10*S*-4

Conf.	Steric Energy	Relative Energy	Distribution
	(kJ/mol)	(kJ/mol)	(%)
4b-1	-813.602588	0.001547	2.77%
4b-2	-813.604135	0.000000	14.27%
4b-3	-813.602682	0.001453	3.06%
4b-4	-813.603421	0.000714	6.70%
4b-5	-813.602665	0.001470	3.01%
4b-6	-813.604095	0.000040	13.67%
4b-7	-813.604076	0.000059	13.40%
4b-8	-813.601964	0.002171	1.43%
4b-9	-813.603001	0.001134	4.29%
4b-10	-813.602641	0.001494	2.93%
4b-11	-813.602651	0.001484	2.96%
4b-12	-813.603233	0.000902	5.49%
4b-13	-813.602977	0.001158	4.19%
4b-14	-813.602031	0.002104	1.54%
4b-15	-813.602718	0.001417	3.18%
4b-16	-813.603104	0.001031	4.79%
4b-17	-813.602457	0.001678	2.41%
4b-18	-813.603009	0.001126	4.33%
4b-19	-813.60287	0.001265	3.74%
4b-20	-813.60219	0.001945	1.82%

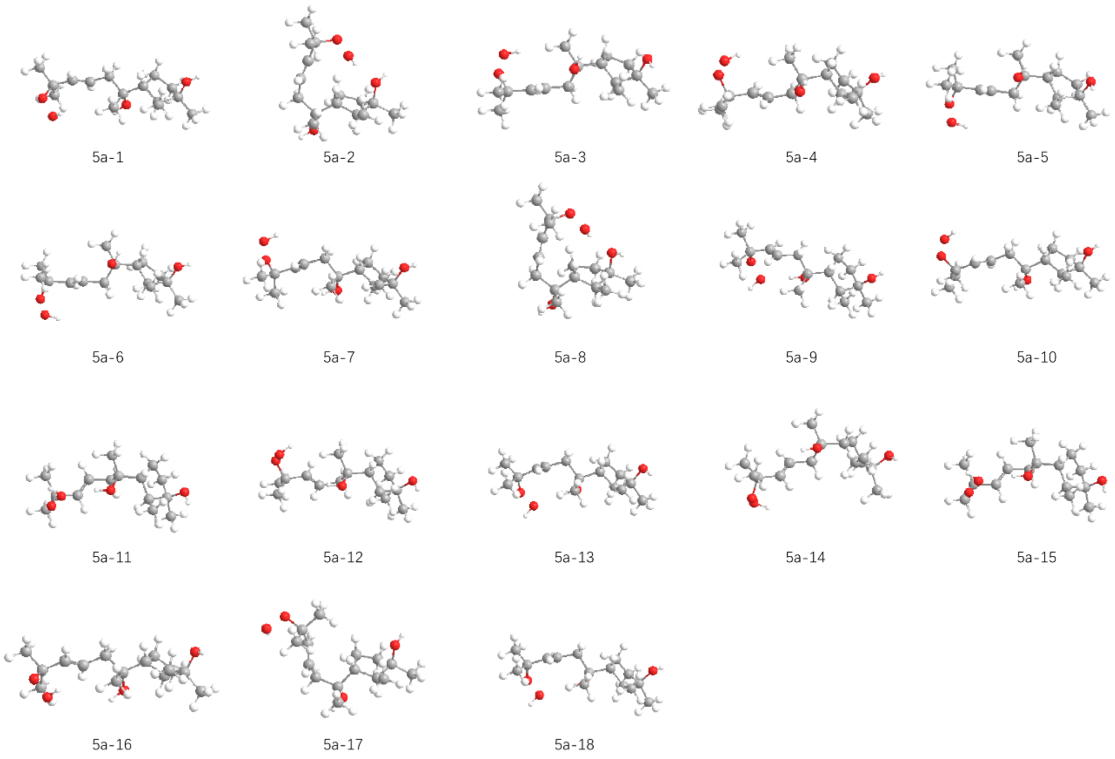

Figure S 91. Most stable conformers of 2*S*, 3*R*, 6*R*, 7*R*-5 calculated at MPW1PW91/6-31G+d, p level

Table S 31. Optimized Z-Matrixes of 2*S*, 3*R*, 6*R*, 7*R*-5 conformations in the Chloroform at MPW1PW91/6-31G+d, p level

	5a-1			5a-2			5a-3		
C	1.849777	-0.19564	-0.57989	-1.01285	0.57233	-0.13224	1.921446	0.568737	0.387647
C	2.957909	0.74749	-0.03399	-2.2272	-0.06767	0.580379	2.758394	-0.68888	0.091514
C	4.187487	-0.15209	0.199278	-2.44873	-1.3509	-0.24619	4.011095	-0.10572	-0.60225
C	3.552043	-1.47096	0.639461	-2.29816	-0.85014	-1.6799	3.426342	0.968108	-1.5266
C	2.358976	-1.63812	-0.3018	-1.10817	0.113495	-1.6149	2.114012	1.446934	-0.86991
C	0.444257	0.061817	0.004814	-0.75493	2.081393	0.00209	0.452646	0.371636	0.822099
C	-0.59352	-0.78878	-0.77418	0.631598	2.408233	-0.63158	-0.2986	-0.60865	-0.09454
C	-1.95405	-0.81346	-0.14066	1.738048	1.576789	-0.04272	-1.76568	-0.698	0.208371
C	-3.05283	-0.28387	-0.68044	2.263305	0.517935	-0.66081	-2.73242	-0.44387	-0.67452
C	-4.44503	-0.29178	-0.0927	3.177134	-0.53029	-0.07354	-4.22221	-0.48599	-0.42098
O	0.49633	-0.35413	1.37723	-1.77309	2.760451	-0.74308	0.404314	-0.23673	2.123064
C	5.185405	0.414442	1.203383	-3.76091	-2.07343	0.021442	4.85116	-1.14035	-1.34351
C	-5.40851	-1.00171	-1.04628	4.340055	-0.81033	-1.02601	-4.91199	-1.33951	-1.48565
C	-4.54136	-0.87302	1.313138	3.684818	-0.21886	1.328953	-4.61994	-0.93289	0.980543
C	3.250645	1.960657	-0.91441	-2.04492	-0.35249	2.071787	3.049379	-1.58898	1.288247
O	4.827585	-0.32485	-1.07625	-1.3384	-2.22856	0.065792	4.83523	0.594502	0.344475
C	0.041602	1.536771	-0.0603	-0.79262	2.57194	1.450858	-0.25642	1.729597	0.890813
O	-4.95428	1.066816	-0.10512	2.46499	-1.79819	-0.08447	-4.75793	0.837074	-0.69599
O	-4.17032	1.884741	0.764824	1.419817	-1.78354	0.894654	-4.2506	1.774802	0.252055
H	1.765339	-0.04395	-1.66163	-0.13572	0.104369	0.314257	2.413941	1.084255	1.224201
H	2.652085	1.092177	0.960554	-3.11689	0.5571	0.433434	2.244768	-1.28287	-0.67586

H	3.205164	-1.37513	1.673146	-3.21165	-0.32106	-1.96914	3.229089	0.530433	-2.50852
H	4.26046	-2.30567	0.599579	-2.15521	-1.67175	-2.38907	4.152693	1.771171	-1.6669
H	1.595191	-2.27778	0.142503	-1.25144	0.957249	-2.29086	1.28032	1.319777	-1.5666
H	2.67859	-2.10494	-1.23628	-0.18763	-0.39357	-1.91344	2.145696	2.506821	-0.61001
H	-0.66186	-0.40256	-1.79614	0.56545	2.234707	-1.70857	-0.15039	-0.32265	-1.13983
H	-0.2242	-1.81654	-0.84073	0.829107	3.477365	-0.48529	0.15029	-1.59814	0.042062
H	-2.0209	-1.31353	0.824266	2.029089	1.801783	0.981931	-2.01937	-0.98353	1.227036
H	-2.98475	0.204128	-1.65456	1.949175	0.299147	-1.68201	-2.46122	-0.14956	-1.69014
H	-0.27368	0.009936	1.833042	-1.63924	3.711193	-0.63268	0.861884	0.349038	2.740838
H	4.712129	0.582143	2.174577	-4.60858	-1.39997	-0.12935	4.244264	-1.69624	-2.06329
H	5.598303	1.362026	0.847326	-3.79779	-2.45888	1.042906	5.297299	-1.86187	-0.65115
H	6.017974	-0.28183	1.350316	-3.87952	-2.91995	-0.66328	5.663431	-0.6451	-1.88235
H	-6.43393	-0.93053	-0.67509	4.963472	-1.62192	-0.64305	-5.99713	-1.30195	-1.36191
H	-5.13391	-2.05563	-1.12027	4.95323	0.087937	-1.12451	-4.58344	-2.37617	-1.38824
H	-5.36593	-0.56074	-2.045	3.97359	-1.08847	-2.01738	-4.66184	-0.98925	-2.49006
H	-5.57043	-0.78784	1.669776	4.311653	-1.04121	1.682012	-5.70777	-0.89964	1.076086
H	-4.26616	-1.93022	1.310547	4.285137	0.694198	1.317304	-4.28831	-1.95798	1.161127
H	-3.88958	-0.34254	2.009135	2.86204	-0.08893	2.032971	-4.18818	-0.28483	1.744433
H	2.37267	2.603179	-1.01089	-2.0308	0.561906	2.664812	2.134494	-2.05584	1.655287
H	3.553319	1.645663	-1.91554	-1.10982	-0.88823	2.254615	3.469553	-1.01585	2.121733
H	4.058158	2.568675	-0.49535	-2.86357	-0.96886	2.454196	3.753674	-2.3856	1.02677
H	5.50875	-1.00154	-0.96854	-1.42667	-3.02144	-0.48008	5.197776	-0.05528	0.961298
H	0.705464	2.152742	0.549309	-1.81204	2.526738	1.838366	0.341583	2.443721	1.466914
H	0.063908	1.907042	-1.0887	-0.14583	1.97615	2.099691	-0.42036	2.153377	-0.10362
H	-0.97776	1.666727	0.318352	-0.458	3.613917	1.506921	-1.22574	1.618996	1.38301
H	-3.53585	2.285441	0.148241	0.602984	-1.84885	0.356352	-3.38845	2.014371	-0.1289
	5a-4			5a-5			5a-6		
C	-1.96356	-0.80573	-0.43114	-2.00929	-0.74589	0.036067	-2.01199	-0.75693	-0.01954
C	-2.88267	-0.1925	0.661486	-2.71556	0.563974	0.430045	-2.7324	0.495715	0.519678
C	-3.78161	0.819719	-0.0762	-3.99493	0.534452	-0.43758	-3.9691	0.595629	-0.39168
C	-2.86091	1.34008	-1.1834	-3.48491	0.019444	-1.78817	-3.38117	0.308482	-1.78001
C	-2.15966	0.079219	-1.69106	-2.24805	-0.85362	-1.48771	-2.24193	-0.70478	-1.55245
C	-0.48649	-0.98545	-0.00016	-0.54075	-0.93458	0.476626	-0.54248	-0.97913	0.403104
C	0.223344	0.356632	0.256961	0.325292	0.294562	0.152287	0.320014	0.276047	0.187479
C	1.671982	0.198994	0.618125	1.784886	0.092549	0.437962	1.781137	0.051822	0.44724
C	2.676131	0.777831	-0.04167	2.748663	0.180946	-0.4799	2.741713	0.224761	-0.46204
C	4.151813	0.640632	0.255581	4.229232	-0.0329	-0.2618	4.223355	-0.00595	-0.2684
O	-0.43599	-1.67562	1.258373	-0.47079	-1.06196	1.906552	-0.4692	-1.23263	1.815508
C	-4.34091	1.916287	0.823321	-4.70875	1.878143	-0.5407	-4.70267	1.929773	-0.313
C	4.789986	2.02318	0.395999	4.618149	-0.40158	1.164967	4.616	-0.50752	1.116282
C	4.483148	-0.23918	1.454961	4.750596	-1.07197	-1.25597	4.744647	-0.94497	-1.35742
C	-3.6501	-1.22285	1.4851	-2.95768	0.76198	1.923072	-3.03838	0.493114	2.013303
O	-4.85955	0.070715	-0.66005	-4.91212	-0.4614	0.044685	-4.85148	-0.46854	-0.00057

C	0.271052	-1.80941	-1.04783	0.032847	-2.20687	-0.15755	0.033513	-2.19108	-0.33936
O	4.801266	0.12513	-0.9382	4.930978	1.168081	-0.67997	4.921731	1.231097	-0.57207
O	4.34777	-1.20135	-1.2046	4.600438	2.251457	0.187322	4.594249	2.2255	0.397157
H	-2.33019	-1.81558	-0.65391	-2.57143	-1.56153	0.512511	-2.56523	-1.62474	0.364351
H	-2.26997	0.393245	1.354092	-2.12487	1.410668	0.056438	-2.12754	1.381025	0.284846
H	-2.13661	2.039036	-0.75019	-4.27962	-0.52578	-2.30144	-4.14878	-0.05596	-2.46948
H	-3.411	1.877674	-1.96294	-3.21246	0.870017	-2.41826	-2.98819	1.238441	-2.20198
H	-1.22752	0.307293	-2.21302	-1.38423	-0.48171	-2.04632	-1.34262	-0.38172	-2.08341
H	-2.80344	-0.43695	-2.40607	-2.39299	-1.89222	-1.79138	-2.49215	-1.69488	-1.93857
H	0.13254	0.995456	-0.62522	0.18639	0.574183	-0.89603	0.175097	0.650559	-0.83014
H	-0.28923	0.863296	1.081357	-0.03872	1.125402	0.765839	-0.04238	1.045742	0.876862
H	1.877844	-0.4402	1.473741	2.033916	-0.1565	1.467272	2.034803	-0.28949	1.448584
H	2.453044	1.410518	-0.903	2.480421	0.423112	-1.51003	2.46995	0.559743	-1.46493
H	-0.80305	-2.55993	1.125765	-1.00561	-1.82546	2.161809	-1.03029	-1.9955	2.009359
H	-3.53546	2.483608	1.297553	-4.02887	2.65797	-0.89421	-4.02572	2.763861	-0.51702
H	-4.97304	1.48822	1.60576	-5.10726	2.193501	0.42925	-5.14273	2.071182	0.677153
H	-4.94963	2.617713	0.242813	-5.546	1.803412	-1.23984	-5.51201	1.966414	-1.05032
H	5.872252	1.934081	0.518822	5.704001	-0.50362	1.229276	5.702252	-0.61223	1.168852
H	4.377409	2.525315	1.273283	4.164839	-1.35446	1.448137	4.165524	-1.48411	1.30869
H	4.586993	2.638793	-0.48366	4.301996	0.362054	1.876846	4.299303	0.1843	1.897888
H	5.567943	-0.30731	1.565518	5.834075	-1.17981	-1.16329	5.828565	-1.05877	-1.27735
H	4.069671	0.192418	2.369403	4.282692	-2.03697	-1.05066	4.279103	-1.92607	-1.24325
H	4.085776	-1.2477	1.332572	4.514105	-0.78359	-2.28305	4.505499	-0.56132	-2.35224
H	-2.95406	-1.84728	2.049916	-2.01401	0.883843	2.456337	-2.11457	0.489286	2.593969
H	-4.25597	-1.86562	0.842316	-3.45995	-0.1074	2.361128	-3.61698	-0.39314	2.286876
H	-4.31967	-0.73883	2.202554	-3.57158	1.648239	2.114453	-3.61775	1.376587	2.298075
H	-5.36077	0.673355	-1.22506	-5.23536	-0.17389	0.908927	-5.58968	-0.48032	-0.62361
H	-0.26037	-2.74373	-1.2567	-0.64056	-3.05387	0.012649	-0.63978	-3.0497	-0.24357
H	0.378894	-1.26751	-1.99121	0.171758	-2.09685	-1.2359	0.175832	-1.98993	-1.40399
H	1.269411	-2.05856	-0.68031	1.002533	-2.44232	0.286719	1.001997	-2.46381	0.085814
H	3.530916	-1.04663	-1.7089	3.770218	2.582221	-0.19583	3.754489	2.581083	0.059179
	5a-7			5a-8			5a-9		
C	1.866306	0.173371	-0.67827	-1.01827	0.56525	-0.11727	1.784264	0.429494	-0.68405
C	3.078633	0.630545	0.182638	-2.2805	-0.04092	0.5379	2.876946	0.534504	0.417839
C	4.030457	-0.58084	0.23344	-2.45544	-1.36218	-0.25116	3.836029	-0.64533	0.164942
C	3.061324	-1.76285	0.230556	-2.1969	-0.92844	-1.68629	2.894621	-1.73	-0.35807
C	2.003869	-1.36945	-0.80049	-1.0238	0.053116	-1.5847	1.963207	-0.98094	-1.31057
C	0.491987	0.612611	-0.12692	-0.76568	2.078358	-0.02252	0.345227	0.682726	-0.18208
C	-0.61108	0.254808	-1.16057	0.637657	2.392252	-0.62257	-0.61603	0.741855	-1.40632
C	-2.00028	0.555697	-0.67179	1.724047	1.553767	-0.0082	-2.05052	0.841869	-0.97611
C	-2.78851	-0.34808	-0.08291	2.303807	0.530027	-0.63642	-2.8191	-0.2278	-0.76477
C	-4.16757	-0.11499	0.491034	3.214206	-0.51477	-0.03763	-4.18537	-0.24646	-0.12748
O	0.28578	-0.12586	1.080891	-1.7628	2.730213	-0.81866	0.010622	-0.42119	0.659705

C	4.980256	-0.56883	1.426294	-3.79445	-2.05834	-0.05552	4.634278	-1.06487	1.394539
C	-4.19682	-0.56337	1.953544	4.432359	-0.73284	-0.93562	-4.69749	1.117755	0.320827
C	-4.68731	1.31055	0.347378	3.638851	-0.24135	1.400212	-5.19814	-0.94653	-1.03325
C	3.752856	1.912071	-0.30306	-2.19259	-0.26545	2.048489	3.581922	1.887959	0.4828
O	4.78599	-0.5711	-0.98948	-1.38092	-2.27647	0.071595	4.734901	-0.23897	-0.8811
C	0.440607	2.112339	0.185452	-0.85078	2.610671	1.409938	0.228751	1.982298	0.62367
O	-5.08391	-1.04963	-0.13375	2.538562	-1.7991	-0.12701	-4.11499	-1.14996	1.009617
O	-5.2196	-0.7409	-1.52026	1.423671	-1.82779	0.770727	-3.08709	-0.71701	1.90463
H	1.957353	0.625952	-1.67173	-0.17234	0.108443	0.399403	1.984661	1.192869	-1.44402
H	2.732307	0.777778	1.211898	-3.15413	0.583703	0.314314	2.406838	0.330013	1.38633
H	2.603679	-1.85473	1.220573	-3.09068	-0.42396	-2.0646	2.322093	-2.14332	0.47807
H	3.563245	-2.70969	0.003308	-1.99498	-1.7872	-2.33093	3.436541	-2.55458	-0.8342
H	1.065208	-1.8939	-0.61761	-1.13962	0.871927	-2.296	1.018104	-1.51164	-1.43113
H	2.341087	-1.63295	-1.80558	-0.0804	-0.44736	-1.81639	2.424637	-0.89325	-2.29704
H	-0.41357	0.815555	-2.07967	0.597365	2.218152	-1.7006	-0.33563	1.601036	-2.0236
H	-0.53524	-0.80787	-1.40059	0.840581	3.460084	-0.47298	-0.47575	-0.15818	-2.00864
H	-2.3496	1.580903	-0.77362	1.958897	1.74618	1.03743	-2.42873	1.832329	-0.73255
H	-2.43564	-1.37722	0.008123	2.049073	0.342213	-1.68005	-2.42927	-1.21752	-1.00482
H	-0.62153	0.040035	1.375731	-1.62765	3.684217	-0.74334	-0.92432	-0.34667	0.911746
H	4.425645	-0.55067	2.36831	-4.61869	-1.37835	-0.28534	3.969689	-1.35517	2.21267
H	5.637082	0.304391	1.390837	-3.92419	-2.40729	0.973997	5.275078	-0.2483	1.737784
H	5.609888	-1.46508	1.423551	-3.86444	-2.92633	-0.71623	5.275519	-1.92169	1.161315
H	-5.2104	-0.48749	2.354508	5.053322	-1.54522	-0.55033	-5.66154	1.000443	0.822009
H	-3.53881	0.077531	2.544074	5.028839	0.181392	-0.96628	-4.83847	1.777047	-0.53857
H	-3.85687	-1.59685	2.053545	4.125793	-0.9801	-1.955	-4.00101	1.601545	1.009536
H	-5.69862	1.369162	0.756147	4.272593	-1.05744	1.75538	-6.15711	-1.06221	-0.52174
H	-4.0523	2.005849	0.901635	4.209116	0.689174	1.454865	-5.35178	-0.34844	-1.93345
H	-4.72017	1.621142	-0.69779	2.776334	-0.16394	2.063445	-4.83674	-1.93398	-1.32867
H	3.06428	2.759371	-0.27281	-2.25102	0.66701	2.6093	2.87831	2.69033	0.715984
H	4.103924	1.796407	-1.33081	-1.24762	-0.74713	2.321253	4.057878	2.120541	-0.47252
H	4.615794	2.16635	0.319897	-3.01357	-0.89662	2.403683	4.357419	1.894936	1.254937
H	5.278655	-1.40114	-1.03165	-1.53878	-2.62826	0.958209	5.237082	-1.01956	-1.14941
H	1.166145	2.372626	0.957918	-1.88399	2.585102	1.760498	0.853846	1.941103	1.517404
H	0.645271	2.711937	-0.70614	-0.23392	2.028306	2.099136	0.524026	2.850668	0.027391
H	-0.54777	2.392591	0.562038	-0.50955	3.651037	1.449564	-0.80236	2.133728	0.957186
H	-4.50346	-1.26155	-1.92117	0.650408	-1.8775	0.172573	-3.59812	-0.29414	2.613621
	5a-10			5a-11			5a-12		
C	1.87303	-0.14987	-0.56797	1.909501	0.983018	0.172158	-1.97677	-0.85633	-0.40299
C	3.061412	0.721035	-0.07229	2.41785	-0.39333	0.681628	-2.83705	-0.16646	0.691912
C	4.215336	-0.26592	0.19221	3.624178	-0.75843	-0.2192	-3.79837	0.783143	-0.06608
C	3.478044	-1.50881	0.690551	3.2678	-0.09924	-1.54887	-2.97798	1.194483	-1.28546
C	2.268265	-1.61672	-0.23771	2.685828	1.255677	-1.14132	-2.29565	-0.10362	-1.72066
C	0.502527	0.244239	0.022365	0.373519	1.074779	0.03593	-0.47237	-0.93902	-0.06433

C	-0.61448	-0.55115	-0.7065	-0.1719	0.152028	-1.09088	0.204669	0.454379	-0.05372
C	-1.94332	-0.50174	-0.00931	-1.67218	0.176736	-1.13185	1.617473	0.432055	0.455447
C	-3.00117	0.187391	-0.44163	-2.43285	-0.69329	-0.46604	2.693705	0.684232	-0.29093
C	-4.35255	0.291684	0.228328	-3.92633	-0.62155	-0.27135	4.134307	0.681437	0.165632
O	0.544319	-0.11485	1.410961	-0.14316	0.648003	1.298162	-0.41286	-1.51199	1.251021
C	5.264339	0.257771	1.16706	3.871478	-2.25956	-0.32405	-4.28086	1.959296	0.775994
C	-4.47751	-0.47494	1.539677	-4.59439	0.590802	-0.91031	4.769932	2.045546	-0.11058
C	-4.71873	1.764714	0.418941	-4.59519	-1.92073	-0.71969	4.343619	0.265568	1.616882
C	3.443033	1.867499	-1.00638	2.715854	-0.41837	2.178287	-3.53146	-1.14685	1.633601
O	4.829086	-0.54303	-1.07776	4.823022	-0.10214	0.223337	-4.92806	0.067849	-0.59082
C	0.217699	1.742706	-0.10388	-0.04714	2.530842	-0.2046	0.23643	-1.87486	-1.04739
O	-5.36263	-0.15518	-0.71331	-4.17219	-0.62808	1.161534	4.890079	-0.18865	-0.71615
O	-5.20544	-1.55005	-0.96829	-3.47903	0.463272	1.772609	4.440352	-1.53608	-0.56833
H	1.784111	-0.03412	-1.65384	2.179356	1.742998	0.91309	-2.31003	-1.89533	-0.49381
H	2.795653	1.13255	0.908168	1.649035	-1.14948	0.494094	-2.19293	0.470889	1.306496
H	3.147609	-1.34134	1.720469	4.138163	-0.02268	-2.2057	-2.23543	1.93968	-0.98105
H	4.115567	-2.39953	0.683238	2.511999	-0.70815	-2.05634	-3.60652	1.639833	-2.0609
H	1.459796	-2.17718	0.23396	2.071485	1.696844	-1.92987	-1.41842	0.079624	-2.34566
H	2.543222	-2.142	-1.15525	3.499781	1.957192	-0.94691	-2.99051	-0.6959	-2.3193
H	-0.70775	-0.1586	-1.72404	0.237758	0.473048	-2.0527	0.187008	0.874587	-1.06303
H	-0.30769	-1.59773	-0.78939	0.171062	-0.86913	-0.90667	-0.38068	1.121362	0.587143
H	-2.01731	-1.06996	0.916382	-2.13703	1.011507	-1.65185	1.743591	0.200181	1.511842
H	-2.92249	0.748652	-1.37462	-1.95684	-1.52171	0.059595	2.562886	0.930189	-1.3464
H	-0.20702	0.303487	1.8515	-1.11293	0.628785	1.243066	0.492486	-1.81094	1.409129
H	4.815083	0.499399	2.134082	2.990236	-2.77624	-0.71391	-3.43811	2.552947	1.140476
H	5.747428	1.155309	0.771891	4.106099	-2.68986	0.655812	-4.84937	1.614413	1.646654
H	6.040614	-0.4963	1.335236	4.714733	-2.45622	-0.99152	-4.93119	2.606137	0.181149
H	-5.4934	-0.36676	1.926449	-5.65984	0.591987	-0.66707	5.835792	2.025132	0.129794
H	-3.78278	-0.07505	2.282203	-4.49344	0.557031	-1.99743	4.284829	2.803272	0.507681
H	-4.27304	-1.53776	1.403881	-4.15376	1.525836	-0.55689	4.652758	2.32617	-1.16
H	-5.72811	1.857153	0.827136	-5.6599	-1.9111	-0.47296	5.413406	0.248317	1.837362
H	-4.01559	2.226881	1.114901	-4.48758	-2.02813	-1.8007	3.866032	0.97929	2.292078
H	-4.67376	2.30517	-0.52957	-4.13092	-2.783	-0.23604	3.935748	-0.72782	1.810122
H	2.62365	2.581594	-1.11413	1.799744	-0.22835	2.740136	-2.78636	-1.72101	2.186773
H	3.696323	1.48858	-1.99903	3.439125	0.356435	2.453278	-4.1523	-1.85829	1.079398
H	4.309443	2.416962	-0.62578	3.110712	-1.38981	2.496877	-4.16504	-0.62811	2.361887
H	5.453905	-1.26799	-0.94484	5.053957	-0.4572	1.091667	-5.45745	-0.24368	0.154874
H	0.928132	2.326661	0.484697	0.366579	3.168275	0.580905	-0.23422	-2.86102	-1.02855
H	0.274757	2.067973	-1.146	0.298431	2.903855	-1.17248	0.198532	-1.49112	-2.06991
H	-0.78989	1.971143	0.259181	-1.13642	2.630395	-0.1755	1.291086	-1.99246	-0.7779
H	-4.55472	-1.55967	-1.69053	-4.19487	1.10205	1.921722	3.790478	-1.61341	-1.28616
	5a-13			5a-14			5a-15		
C	1.782981	0.424538	-0.68069	-2.08207	-1.01154	-0.13943	1.909495	0.983004	0.172178

C	2.876828	0.524304	0.419286	-2.5488	0.15698	0.771447	2.417853	-0.39336	0.681607
C	3.841825	-0.65713	0.153862	-3.59313	0.945964	-0.05776	3.624199	-0.75841	-0.21922
C	2.905859	-1.73537	-0.37669	-3.10793	0.748671	-1.49132	3.267832	-0.09918	-1.54887
C	1.960516	-0.98336	-1.31381	-2.68979	-0.72218	-1.53579	2.685824	1.255711	-1.14128
C	0.344851	0.679358	-0.17743	-0.55413	-1.23724	-0.14586	0.373512	1.074761	0.035952
C	-0.61651	0.740796	-1.40128	0.212668	-0.05798	-0.79965	-0.17191	0.152034	-1.09088
C	-2.0509	0.842859	-0.97128	1.704333	-0.23698	-0.75233	-1.67219	0.176749	-1.13185
C	-2.82204	-0.22556	-0.76304	2.487965	0.313392	0.178879	-2.43286	-0.69329	-0.46606
C	-4.18979	-0.24215	-0.12881	3.98115	0.133576	0.331983	-3.92634	-0.62154	-0.27136
O	0.009494	-0.42442	0.663895	-0.18025	-1.33454	1.232503	-0.14316	0.647958	1.298178
C	4.638509	-1.08503	1.381519	-3.71615	2.409154	0.354044	3.871522	-2.25953	-0.32411
C	-4.69932	1.122371	0.32155	4.294623	-0.36188	1.745218	-4.5944	0.590825	-0.9103
C	-5.20214	-0.93704	-1.0391	4.627618	-0.7638	-0.71664	-4.59522	-1.92071	-0.71972
C	3.578482	1.879594	0.48762	-3.04371	-0.29622	2.142444	2.715836	-0.41844	2.178269
O	4.741373	-0.35781	-0.92662	-4.88335	0.317467	-0.0024	4.823028	-0.10211	0.223361
C	0.231277	1.978605	0.629412	-0.22068	-2.56123	-0.84509	-0.04715	2.530828	-0.20454
O	-4.12463	-1.14864	1.006	4.600246	1.44504	0.322692	-4.17219	-0.62809	1.161524
O	-3.09836	-0.7199	1.904943	4.445219	2.044521	-0.96293	-3.47901	0.463245	1.772608
H	1.98311	1.19027	-1.43858	-2.50865	-1.94122	0.251763	2.179342	1.742964	0.913134
H	2.410649	0.318971	1.389578	-1.70787	0.837068	0.940914	1.649051	-1.14951	0.494037
H	3.45877	-2.5419	-0.8655	-2.24719	1.402276	-1.66883	4.138205	-0.02258	-2.20569
H	2.343189	-2.15872	0.46051	-3.88346	1.005544	-2.21741	2.512053	-0.70809	-2.05637
H	1.015162	-1.51608	-1.42411	-2.00902	-0.93489	-2.36357	2.071477	1.696887	-1.92983
H	2.409229	-0.89242	-2.3056	-3.57297	-1.34611	-1.68674	3.499759	1.957238	-0.94685
H	-0.33487	1.599961	-2.01807	-0.10399	0.044215	-1.84156	0.237752	0.473071	-2.05269
H	-0.47745	-0.15906	-2.00409	-0.05607	0.864962	-0.27949	0.171048	-0.86913	-0.90669
H	-2.42743	1.833647	-0.72637	2.144651	-0.88913	-1.50365	-2.13704	1.011535	-1.65183
H	-2.43423	-1.21567	-1.00473	2.039324	0.972355	0.924709	-1.95685	-1.52172	0.059552
H	-0.92646	-0.35151	0.912653	0.786575	-1.38168	1.271111	-1.11293	0.628753	1.243092
H	3.972877	-1.36106	2.203435	-2.75563	2.92397	0.264291	2.990292	-2.77621	-0.714
H	5.29126	-0.27858	1.733865	-4.04925	2.500149	1.393776	4.106137	-2.68986	0.655741
H	5.26687	-1.94593	1.138062	-4.44525	2.918941	-0.28133	4.714788	-2.45616	-0.99157
H	-5.66485	1.006433	0.820194	5.375038	-0.41435	1.899386	-5.65985	0.592009	-0.66706
H	-4.8366	1.784017	-0.53665	3.872827	-1.35971	1.882924	-4.49346	0.557073	-1.99741
H	-4.00318	1.602646	1.013062	3.865214	0.303575	2.49785	-4.15376	1.525851	-0.55687
H	-6.16294	-1.05086	-0.53063	5.705531	-0.80262	-0.54396	-5.65992	-1.91107	-0.47298
H	-5.35125	-0.33645	-1.9384	4.231959	-1.77981	-0.64563	-4.48761	-2.02809	-1.80073
H	-4.84293	-1.92491	-1.33571	4.454068	-0.38868	-1.72614	-4.13095	-2.78299	-0.23608
H	2.886461	2.676016	0.768111	-2.22924	-0.77074	2.692448	1.799712	-0.22847	2.740111
H	4.003897	2.150762	-0.48395	-3.85194	-1.02921	2.051564	3.439084	0.356367	2.453301
H	4.385232	1.878687	1.228871	-3.40687	0.546993	2.740843	3.110713	-1.38989	2.496827
H	5.355762	0.323345	-0.62368	-5.20776	0.380465	0.905377	5.053958	-0.45721	1.091681
H	0.851907	1.932326	1.526094	-0.78442	-3.37519	-0.38268	0.366562	3.168244	0.580976

H	0.533392	2.846377	0.035526	-0.46288	-2.53333	-1.91067	0.298422	2.903864	-1.17242
H	-0.80027	2.135012	0.959062	0.843648	-2.79624	-0.74991	-1.13643	2.630375	-0.17544
H	-3.61088	-0.29986	2.614532	3.596823	2.510464	-0.87512	-4.19484	1.102032	1.921736
	5a-16			5a-17			5a-18		
C	1.848546	-0.19586	-0.57443	-1.183	0.268557	-0.02746	1.784371	0.429424	-0.68405
C	2.958703	0.74021	-0.02296	-2.54344	-0.001	0.675384	2.877074	0.534544	0.417814
C	4.193371	-0.16791	0.196785	-3.30178	-0.96955	-0.25394	3.836193	-0.64526	0.164976
C	3.56078	-1.48771	0.619708	-2.84425	-0.52506	-1.64333	2.894908	-1.73001	-0.3581
C	2.355507	-1.64162	-0.30883	-1.3462	-0.27123	-1.4744	1.963337	-0.98105	-1.31053
C	0.443098	0.060892	0.010331	-0.72382	1.739648	0.022517	0.345346	0.682649	-0.18207
C	-0.59454	-0.78425	-0.77474	0.679511	1.909784	-0.61541	-0.616	0.74159	-1.40622
C	-1.95612	-0.81191	-0.14358	1.767105	1.131863	0.067637	-2.05045	0.841775	-0.97593
C	-3.05306	-0.27636	-0.68128	2.331589	0.038977	-0.44833	-2.81917	-0.22781	-0.76462
C	-4.44689	-0.2884	-0.09755	3.366133	-0.84458	0.208399	-4.18555	-0.24632	-0.12754
O	0.493553	-0.36144	1.380463	-1.66328	2.483611	-0.76573	0.010845	-0.42128	0.659825
C	5.190757	0.38659	1.208069	-4.81504	-0.94429	-0.06822	4.634463	-1.06476	1.394566
C	-5.40791	-0.98933	-1.06027	3.827529	-0.3757	1.583265	-4.6976	1.117897	0.320824
C	-4.54763	-0.88208	1.302735	2.840313	-2.28033	0.267837	-5.19824	-0.94618	-1.03358
C	3.247678	1.959988	-0.89617	-2.42377	-0.50985	2.110778	3.582046	1.888011	0.482697
O	4.862348	-0.439	-1.0459	-2.80175	-2.28925	0.020117	4.735087	-0.23883	-0.88104
C	0.043277	1.536981	-0.04815	-0.71213	2.29596	1.449753	0.228776	1.982221	0.623651
O	-4.95531	1.070567	-0.09959	4.500948	-0.96943	-0.68693	-4.11552	-1.15001	1.009447
O	-4.17308	1.880574	0.779294	5.150305	0.293898	-0.82435	-3.0878	-0.71722	1.904736
H	1.763882	-0.0366	-1.65522	-0.40977	-0.31203	0.484073	1.984763	1.192766	-1.44406
H	2.656471	1.082029	0.97376	-3.12237	0.929483	0.672745	2.40698	0.330064	1.386316
H	3.226461	-1.40265	1.657946	-3.35506	0.405847	-1.90914	2.322446	-2.14346	0.478021
H	4.273459	-2.31384	0.55267	-3.07804	-1.26793	-2.41386	3.436913	-2.5545	-0.83429
H	1.593155	-2.27909	0.141258	-0.97725	0.420741	-2.23222	1.018251	-1.51184	-1.43088
H	2.66254	-2.10676	-1.24809	-0.78943	-1.20544	-1.58357	2.42459	-0.89339	-2.29708
H	-0.66126	-0.39187	-1.79447	0.624147	1.627393	-1.66892	-0.33561	1.600647	-2.02369
H	-0.22578	-1.81178	-0.84683	0.925976	2.978931	-0.58508	-0.47581	-0.15857	-2.00838
H	-2.02571	-1.32037	0.816738	2.069391	1.476973	1.054735	-2.42856	1.832287	-0.73242
H	-2.98212	0.219682	-1.65113	2.014584	-0.30302	-1.43541	-2.42945	-1.21756	-1.00471
H	-0.28093	-0.00589	1.835479	-1.44043	3.421041	-0.69202	-0.92407	-0.34675	0.911958
H	4.712151	0.559364	2.175523	-5.21547	0.056718	-0.24968	3.969882	-1.35493	2.212751
H	5.615746	1.337771	0.86886	-5.08663	-1.25068	0.945512	5.275346	-0.24822	1.737712
H	6.013418	-0.31972	1.34782	-5.29867	-1.63293	-0.76934	5.275614	-1.92167	1.161387
H	-6.43433	-0.92129	-0.69127	4.600758	-1.05195	1.955056	-5.66173	1.000618	0.821864
H	-5.13337	-2.04259	-1.14319	2.993449	-0.38667	2.288912	-4.83842	1.777259	-0.53856
H	-5.36246	-0.53922	-2.05478	4.241881	0.632744	1.543872	-4.00123	1.601634	1.009677
H	-5.57764	-0.79933	1.657183	3.602553	-2.9497	0.674175	-6.15739	-1.06161	-0.52236
H	-4.27322	-1.93941	1.291421	1.959365	-2.31763	0.911884	-5.35144	-0.3481	-1.93386
H	-3.89742	-0.35841	2.005324	2.556722	-2.63401	-0.72632	-4.83695	-1.93372	-1.32883

H	2.386562	2.628711	-0.95068	-1.94227	0.228614	2.755581	2.878442	2.690441	0.715679
H	3.488187	1.660757	-1.92134	-1.83323	-1.42817	2.14539	4.058171	2.120446	-0.47257
H	4.085125	2.546803	-0.50328	-3.4076	-0.72723	2.537807	4.357425	1.895051	1.254956
H	5.281812	0.378316	-1.34457	-3.16532	-2.88079	-0.65171	5.23731	-1.01939	-1.14933
H	0.704896	2.147591	0.569391	-1.72565	2.348315	1.852098	0.853719	1.941038	1.517491
H	0.071704	1.913483	-1.07425	-0.10688	1.678452	2.118286	0.524171	2.850557	0.027385
H	-0.97773	1.666834	0.32593	-0.29354	3.308644	1.4595	-0.80239	2.133694	0.956981
H	-3.53718	2.286446	0.167604	4.6565	0.704422	-1.55408	-3.59899	-0.29446	2.613664

Table S 32. Energy analysis for 2*S*, 3*R*, 6*R*, 7*R*-5 in the Chloroform at MPW1PW91/6-31G+d, p level

Conf.	Steric Energy	Relative Energy	Distribution
	(kJ/mol)	(kJ/mol)	(%)
5a-1	-888.719963	0.002358	1.63%
5a-2	-888.720788	0.001533	3.90%
5a-3	-888.721669	0.000652	9.91%
5a-4	-888.720912	0.001409	4.45%
5a-5	-888.722321	0	19.77%
5a-6	-888.721761	0.00056	10.93%
5a-7	-888.720337	0.001984	2.42%
5a-8	-888.721019	0.001302	4.98%
5a-9	-888.720709	0.001612	3.59%
5a-10	-888.719785	0.002536	1.35%
5a-11	-888.721102	0.001219	5.44%
5a-12	-888.720349	0.001972	2.45%
5a-13	-888.720907	0.001414	4.43%
5a-14	-888.720476	0.001845	2.80%
5a-15	-888.721101	0.00122	5.43%
5a-16	-888.719821	0.0025	1.40%
5a-17	-888.721815	0.000506	11.57%
5a-18	-888.720695	0.001626	3.54%

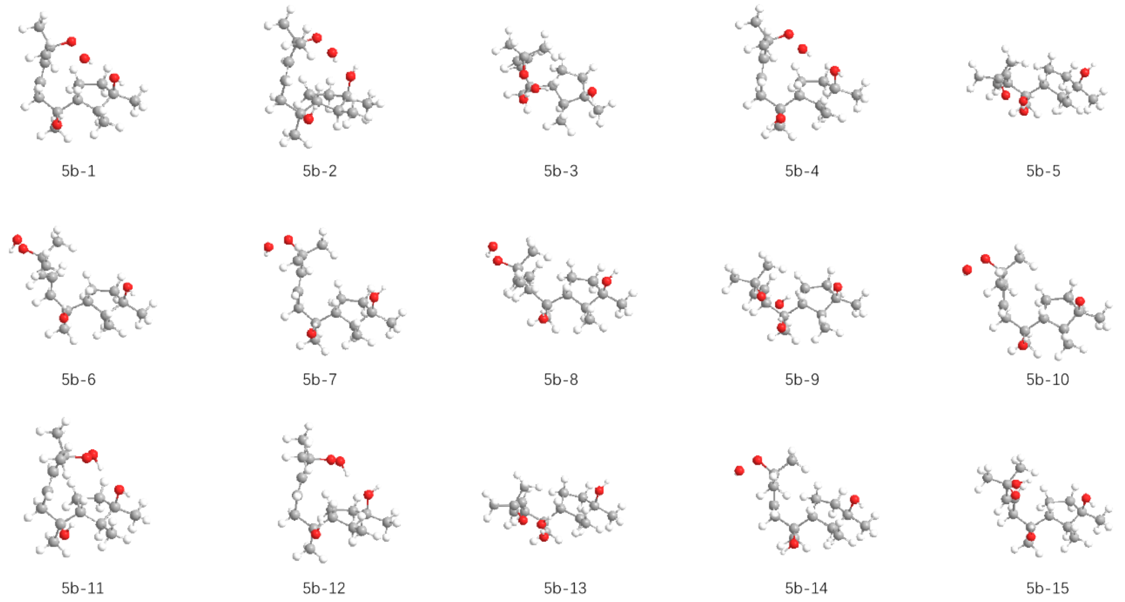

Figure S 92. Most stable conformers of 2*S*, 3*R*, 6*R*, 7*S*-5 calculated at MPW1PW91/6-31G+d, p level

Table S 33. Optimized Z-Matrixes of 2*S*, 3*R*, 6*R*, 7*S*-5 conformations in the Chloroform at MPW1PW91/6-31G+d, p level

	5b-1			5b-2			5b-3		
C	-1.06138	0.555242	-0.15595	-1.05797	-1.05797	-0.18901	-1.22065	-1.22065	-0.21773
C	-2.36273	-0.03272	0.422449	-2.28426	-2.28426	0.499926	-2.4551	-2.4551	0.613507
C	-2.40078	-1.42676	-0.24867	-2.39855	-2.39855	-0.24824	-3.15189	-3.15189	-0.2903
C	-2.04887	-1.10121	-1.69625	-2.2282	-2.2282	-1.70853	-3.01848	-3.01848	-1.69082
C	-1.00546	0.027104	-1.61303	-1.17818	-1.17818	-1.67566	-1.75697	-1.75697	-1.66501
C	-0.78321	2.063808	-0.00723	-0.74674	-0.74674	0.031998	-0.42528	-0.42528	0.240583
C	0.608449	2.397792	-0.61764	0.608648	0.608648	-0.65163	0.678707	0.678707	-0.79637
C	1.700682	1.557498	-0.0159	1.736165	1.736165	-0.09176	1.60658	1.60658	-1.01361
C	2.30183	0.545378	-0.64265	2.226621	2.226621	-0.6798	2.713093	2.713093	-0.29454
C	3.233456	-0.47793	-0.038	3.172616	3.172616	-0.08687	3.551248	3.551248	-0.25605
O	-0.77002	2.431036	1.381728	-0.63062	-0.63062	1.435042	0.199583	0.199583	1.49973
C	-3.71544	-2.17709	-0.0926	-3.6784	-3.6784	0.020586	-4.59675	-4.59675	0.097739
C	4.421867	-0.72339	-0.96787	4.285678	4.285678	-1.08242	3.060229	3.060229	-1.16201
C	3.70746	-0.15548	1.374135	3.751331	3.751331	1.269922	5.023333	5.023333	-0.51655
C	-0.25101	0.064655	0.393458	-0.19445	-0.19445	0.225825	-0.5271	-0.5271	-0.15718
O	-2.4571	-0.06272	1.945172	-2.20749	-2.20749	2.018127	-2.17214	-2.17214	2.035684
C	-1.31551	-2.25037	0.236755	-1.25838	-1.25838	0.15639	-2.40242	-2.40242	-0.31919
O	-1.85308	2.950878	-0.63286	-1.84044	-1.84044	-0.46267	-1.30734	-1.30734	0.480365
O	2.559671	-1.76622	-0.0608	2.464413	2.464413	0.004668	3.575871	3.575871	1.127217
H	1.480538	-1.76854	0.879715	1.457164	1.457164	1.019992	2.239633	2.239633	1.596042
H	-3.22789	0.516385	0.02901	-3.18894	-3.18894	0.23959	-3.1387	-3.1387	0.660312
H	-1.68668	-1.98879	-2.21954	-1.93638	-1.93638	-2.34126	-2.96593	-2.96593	-2.43668
H	-2.95234	-0.75817	-2.20778	-3.18604	-3.18604	-2.08468	-3.90717	-3.90717	-1.91193
H	-1.2269	0.809916	-2.34219	-1.484	-1.484	-2.31725	-2.01809	-2.01809	-1.93991

H	-0.0068	-0.34431	-1.84792	-0.21682	-0.21682	-2.05466	-1.00646	-1.00646	-2.38484
H	0.795688	3.459923	-0.42817	0.794757	0.794757	-0.48165	1.229655	1.229655	-0.39417
H	0.578317	2.2545	-1.70156	0.523595	0.523595	-1.73112	0.213567	0.213567	-1.73535
H	1.943747	1.755776	1.027167	2.090675	2.090675	0.899834	1.27811	1.27811	-1.70909
H	2.060932	0.355352	-1.689	1.868186	1.868186	-1.67388	3.031835	3.031835	0.409171
H	-0.25511	1.771075	1.864974	-0.09081	-0.09081	1.838266	0.790977	0.790977	1.39411
H	-3.69442	-3.09776	-0.6813	-3.73176	-3.73176	-0.61967	-5.05028	-5.05028	-0.63492
H	-3.9019	-2.44764	0.951616	-3.72329	-3.72329	1.060736	-4.65545	-4.65545	1.079244
H	-4.55321	-1.56382	-0.43427	-4.55627	-4.55627	-0.18869	-5.18768	-5.18768	0.138582
H	5.060886	-1.51711	-0.5736	4.927452	4.927452	-0.69247	3.689295	3.689295	-1.02386
H	4.082757	-1.01085	-1.96622	3.869693	3.869693	-2.03939	2.024331	2.024331	-0.94438
H	5.010985	0.192147	-1.05345	4.893391	4.893391	-1.25492	3.118469	3.118469	-2.21108
H	4.360005	-0.95571	1.731397	4.403517	4.403517	1.627553	5.641636	5.641636	-0.36894
H	2.87098	-0.06319	2.067897	2.966045	2.966045	2.010312	5.375191	5.375191	0.153835
H	4.271561	0.780258	1.377903	4.341585	4.341585	1.183775	5.142111	5.142111	-1.54666
H	-3.33237	-0.62862	2.279054	-3.04174	-3.04174	2.403186	-3.07581	-3.07581	2.512809
H	-2.54083	0.94722	2.348248	-2.24848	-2.24848	2.493316	-1.79469	-1.79469	2.648702
H	-1.56584	-0.52064	2.389624	-1.2795	-1.2795	2.321894	-1.40488	-1.40488	2.046964
H	-1.51447	-2.50991	1.146744	-1.30729	-1.30729	-0.31029	-2.44006	-2.44006	0.564528
H	-2.82317	2.769859	-0.16418	-2.79373	-2.79373	0.018209	-1.9866	-1.9866	1.31749
H	-1.59653	4.002714	-0.48216	-1.57861	-1.57861	-0.21325	-0.6869	-0.6869	0.727628
H	-1.94532	2.774155	-1.70637	-1.96975	-1.96975	-1.54474	-1.90009	-1.90009	-0.40417
H	0.687279	-1.85712	0.312794	0.623191	0.623191	0.512862	2.137841	2.137841	1.530062
	5b-4			5b-5			5b-6		
C	-1.04766	0.542831	-0.12012	-1.16231	0.349454	-0.28406	-1.40384	0.332499	-0.18857
C	-2.36213	-0.04856	0.425606	-2.11314	-0.52026	0.583995	-2.76607	0.183493	0.513166
C	-2.38845	-1.44081	-0.24961	-3.39493	-0.69625	-0.25428	-3.39236	-1.02484	-0.22025
C	-2.00031	-1.11111	-1.68557	-3.47015	0.620604	-1.03274	-3.03172	-0.76279	-1.68613
C	-0.91779	-0.0272	-1.55825	-2.02365	0.866749	-1.46496	-1.72196	0.053965	-1.67538
C	-0.81267	2.058826	-0.02273	-0.43473	1.46279	0.510717	-0.61059	1.631815	0.075134
C	0.59484	2.39775	-0.58613	0.673472	2.135984	-0.35343	0.64801	1.70531	-0.83055
C	1.660902	1.53918	0.03424	1.688959	1.131869	-0.81284	1.547748	0.512479	-0.67515
C	2.330667	0.591859	-0.62323	2.766153	0.808121	-0.09594	2.622992	0.488772	0.116802
C	3.242553	-0.45479	-0.02694	3.681327	-0.36651	-0.32962	3.524786	-0.6952	0.382421
O	-0.828	2.371995	1.378502	0.164475	0.89904	1.678678	-0.18286	1.67623	1.441641
C	-3.70776	-2.1887	-0.12389	-4.6372	-0.9904	0.579259	-4.89144	-1.18504	0.008028
C	4.452818	-0.67513	-0.93452	3.304165	-1.24023	-1.52069	3.609993	-0.94448	1.889602
C	3.683809	-0.17478	1.404862	5.140545	0.079682	-0.40949	3.140964	-1.96998	-0.35975
C	-0.253	0.103235	0.488108	-0.38257	-0.31205	-0.67634	-0.77868	-0.49333	0.17629
O	-2.48172	-0.0819	1.946348	-1.492	-1.83117	1.059106	-2.71815	0.056	2.032366
C	-1.31427	-2.26692	0.253183	-3.15439	-1.78029	-1.16682	-2.718	-2.24181	0.141289
O	-1.88057	2.893769	-0.73038	-1.37478	2.549389	1.028599	-1.44569	2.893795	-0.11669
O	2.568872	-1.74226	-0.10405	3.669256	-1.16113	0.890307	4.886074	-0.31386	0.057489

H	1.496009	-1.78924	0.841404	2.325833	-1.52434	1.218181	5.003243	-0.08685	-1.34634
H	-3.22085	0.500564	0.01797	-2.4027	0.049139	1.472745	-3.39327	1.046654	0.254508
H	-1.6617	-2.00242	-2.2187	-4.17434	0.569763	-1.87002	-2.9418	-1.70995	-2.22199
H	-2.88075	-0.72361	-2.20627	-3.81238	1.416617	-0.36223	-3.83655	-0.1942	-2.15895
H	-1.04748	0.738398	-2.32673	-1.84058	1.915365	-1.71078	-1.85997	0.987875	-2.22745
H	0.077346	-0.44996	-1.70978	-1.80436	0.286995	-2.36446	-0.90489	-0.48077	-2.16512
H	0.788682	3.458526	-0.38129	1.150061	2.897193	0.271125	1.188113	2.616119	-0.55558
H	0.597913	2.279055	-1.6735	0.216022	2.645553	-1.2062	0.339057	1.806037	-1.87523
H	1.799526	1.656716	1.106717	1.453891	0.569462	-1.71382	1.260025	-0.39225	-1.20706
H	2.161592	0.47392	-1.69437	2.992339	1.372873	0.808854	2.912782	1.399345	0.644437
H	-0.70436	3.326832	1.46744	0.813357	0.232614	1.397131	0.41934	0.93104	1.583294
H	-3.67393	-3.11117	-0.70931	-5.51832	-1.09314	-0.06327	-5.28025	-1.99538	-0.61455
H	-3.9191	-2.45582	0.916374	-4.51509	-1.92316	1.136252	-5.11086	-1.42844	1.052753
H	-4.53624	-1.57512	-0.48753	-4.83204	-0.1833	1.29068	-5.42731	-0.2657	-0.24294
H	5.086523	-1.47516	-0.54448	3.980484	-2.09695	-1.575	4.325193	-1.74228	2.103955
H	4.137394	-0.94116	-1.94668	2.278574	-1.60838	-1.44045	3.923249	-0.04154	2.41904
H	5.03992	0.244403	-0.9869	3.390399	-0.6782	-2.45339	2.629002	-1.2442	2.264391
H	4.328221	-0.98548	1.75368	5.806954	-0.78551	-0.45401	3.865491	-2.75436	-0.12911
H	2.832155	-0.10182	2.081755	5.408937	0.685088	0.459052	3.132315	-1.81714	-1.43982
H	4.247023	0.760571	1.446855	5.285619	0.679265	-1.31016	2.152157	-2.31218	-0.04506
H	-3.37677	-0.62679	2.263526	-2.21253	-2.43314	1.62135	-3.70593	-0.17466	2.444783
H	-2.53607	0.929195	2.349444	-0.64282	-1.6278	1.715985	-2.36952	0.98369	2.487302
H	-1.6059	-0.55685	2.401563	-1.14429	-2.42828	0.212395	-2.02154	-0.73068	2.342097
H	-1.50337	-2.48402	1.176294	-3.90573	-1.8211	-1.77306	-2.90705	-2.41984	1.072269
H	-2.85459	2.743479	-0.25892	-2.15403	2.130272	1.669073	-2.23711	2.947338	0.633955
H	-1.63434	3.959095	-0.66494	-0.80704	3.269967	1.62266	-0.81607	3.780006	-0.00421
H	-1.96467	2.641373	-1.79004	-1.85407	3.087148	0.207583	-1.90421	2.922306	-1.10785
H	0.696564	-1.84211	0.279357	2.27431	-2.44151	0.90377	4.764466	0.851922	-1.42709
	5b-7			5b-8			5b-9		
C	-1.25905	0.413284	-0.06795	-1.36282	0.291414	-0.13178	-1.22064	0.446993	-0.21773
C	-2.70807	0.140424	0.394495	-2.6651	0.012831	0.656327	-2.45509	0.053217	0.613508
C	-2.99307	-1.26393	-0.16811	-3.4674	-0.90626	-0.28249	-3.15189	-0.98955	-0.2903
C	-2.39935	-1.17482	-1.57727	-3.22789	-0.26063	-1.6493	-3.01848	-0.38074	-1.69082
C	-1.12533	-0.32641	-1.42644	-1.75003	0.155046	-1.63164	-1.75697	0.508933	-1.66501
C	-0.79451	1.886038	-0.09146	-0.61606	1.599865	0.21656	-0.42528	1.690572	0.240584
C	0.650659	1.997449	-0.64351	0.654642	1.766335	-0.66094	0.678707	2.051703	-0.79637
C	1.639066	1.12851	0.078139	1.563948	0.57229	-0.61444	1.60658	0.893396	-1.01361
C	2.295554	0.111661	-0.48212	2.639187	0.483112	0.172814	2.713092	0.700672	-0.29454
C	3.228679	-0.85678	0.205767	3.559002	-0.70857	0.314991	3.551244	-0.55165	-0.25605
O	-0.81375	2.432746	1.237292	-0.21059	1.58206	1.590109	0.199585	1.429851	1.499731
C	-4.46455	-1.66193	-0.157	-4.94272	-1.03938	0.076964	-4.59674	-1.28521	0.09774
C	3.474501	-0.57024	1.682357	3.713114	-1.06358	1.794739	3.060225	-1.67536	-1.16201
C	2.708445	-2.28198	0.004433	3.150812	-1.93118	-0.49848	5.02333	-0.2349	-0.51654

C	-0.6013	-0.10344	0.6437	-0.67559	-0.52799	0.111819	-0.5271	-0.40213	-0.15719
O	-2.96121	0.286195	1.891941	-2.46276	-0.54348	2.062401	-2.17214	-0.42078	2.035684
C	-2.23081	-2.1853	0.628549	-2.83298	-2.19447	-0.23593	-2.40241	-2.21669	-0.31919
O	-1.71099	2.795851	-0.90432	-1.48524	2.846089	0.07595	-1.30734	2.911969	0.480368
O	4.485141	-0.88113	-0.51857	4.902483	-0.29133	-0.04198	3.575861	-1.00453	1.127217
H	5.139375	0.380823	-0.39316	4.956804	0.03357	-1.43011	2.239622	-1.20207	1.596038
H	-3.38798	0.813402	-0.14197	-3.24799	0.938374	0.728638	-3.1387	0.911183	0.660315
H	-2.20958	-2.16578	-2.00142	-3.47739	-0.93698	-2.47308	-2.96593	-1.17666	-2.43668
H	-3.11807	-0.67513	-2.23453	-3.8724	0.619518	-1.74457	-3.90717	0.215874	-1.91193
H	-1.02602	0.363797	-2.26757	-1.60472	1.081546	-2.19154	-2.0181	1.534886	-1.93991
H	-0.22813	-0.94896	-1.42507	-1.12665	-0.6023	-2.11274	-1.00646	0.173747	-2.38484
H	0.944409	3.047477	-0.53647	1.181429	2.650969	-0.29108	1.229657	2.906865	-0.39417
H	0.660256	1.762878	-1.71155	0.362292	1.967016	-1.69571	0.213566	2.365308	-1.73535
H	1.794993	1.345733	1.133995	1.28554	-0.27804	-1.23396	1.278112	0.123746	-1.70909
H	2.142293	-0.09731	-1.54241	2.919791	1.342314	0.784664	3.031831	1.470009	0.409175
H	-0.45343	1.771998	1.843137	0.388785	0.830595	1.708539	0.790976	0.666324	1.394112
H	-4.60313	-2.62983	-0.65077	-5.46614	-1.64586	-0.67008	-5.05028	-1.95827	-0.63492
H	-4.83482	-1.75094	0.867175	-5.0624	-1.52483	1.048687	-4.65545	-1.76713	1.079243
H	-5.07501	-0.92531	-0.68634	-5.42805	-0.06027	0.113882	-5.18767	-0.36626	0.138586
H	4.192415	-1.29348	2.07597	4.442591	-1.86731	1.919804	3.689288	-2.5583	-1.02386
H	3.877125	0.432233	1.834341	4.043521	-0.19746	2.373032	2.024325	-1.94592	-0.94438
H	2.544961	-0.66665	2.248478	2.752243	-1.39932	2.190292	3.118469	-1.37645	-2.21108
H	3.402539	-3.00742	0.435987	3.884279	-2.72676	-0.34848	5.641631	-1.12405	-0.36894
H	2.584146	-2.50517	-1.0581	3.103165	-1.70575	-1.56467	5.375188	0.552353	0.153844
H	1.738635	-2.38613	0.495639	2.174215	-2.29685	-0.17216	5.142113	0.10639	-1.54666
H	-3.97513	-0.03053	2.153378	-3.42051	-0.78335	2.534099	-3.0758	-0.81458	2.512808
H	-2.84574	1.326344	2.201655	-1.94973	0.187252	2.68955	-1.79468	0.398159	2.648704
H	-2.26113	-0.32731	2.466003	-1.86421	-1.45784	2.034055	-1.40488	-1.20263	2.046962
H	-2.31456	-3.05954	0.226241	-3.25279	-2.75133	-0.90448	-2.44004	-2.60629	0.564524
H	-2.69619	2.864261	-0.43914	-2.2732	2.853967	0.831425	-1.9866	2.736825	1.317492
H	-1.29169	3.8043	-0.95036	-0.87874	3.744296	0.217098	-0.6869	3.777185	0.727633
H	-1.83159	2.429726	-1.92666	-1.94986	2.897961	-0.9116	-1.90009	3.157148	-0.40417
H	4.747376	0.893673	-1.12006	4.68256	0.96628	-1.43813	2.137825	-2.16542	1.530055
	5b-10			5b-11			5b-12		
C	-1.28849	0.412716	-0.09633	-1.05272	0.560436	-0.18441	-1.03694	0.558068	-0.16527
C	-2.75292	0.163988	0.314642	-2.32251	-0.02843	0.457684	-2.31412	-0.00432	0.490766
C	-2.9925	-1.29152	-0.14755	-2.30205	-1.47287	-0.09645	-2.37051	-1.42389	-0.10544
C	-2.31677	-1.32456	-1.51961	-1.97287	-1.25927	-1.57199	-2.08176	-1.1703	-1.58648
C	-1.13999	-0.33053	-1.44562	-1.04382	-0.02975	-1.61564	-1.04046	-0.03569	-1.59882
C	-0.7923	1.874734	-0.10495	-0.80115	2.079881	-0.11001	-0.76787	2.075924	-0.09976
C	0.650818	1.962251	-0.66342	0.57376	2.415781	-0.76038	0.606181	2.394795	-0.75971
C	1.633306	1.091872	0.064926	1.688449	1.657846	-0.09451	1.718774	1.628126	-0.10145
C	2.316594	0.093013	-0.49568	2.254561	0.56334	-0.60502	2.277002	0.533009	-0.61916

C	3.258877	-0.86399	0.196377	3.143324	-0.41794	0.129649	3.17242	-0.4487	0.108003
O	-0.79406	2.40676	1.230402	-0.77065	2.511691	1.26002	-0.7246	2.515045	1.266988
C	-4.46061	-1.70048	-0.1928	-3.57757	-2.2694	0.136102	-3.66945	-2.17373	0.148533
C	3.444821	-0.61314	1.688144	4.16582	-1.03318	-0.82394	4.137374	-1.11722	-0.86919
C	2.796838	-2.29948	-0.06327	3.830041	0.162692	1.362092	3.925498	0.151454	1.291675
C	-0.66202	-0.12231	0.631603	-0.21662	0.100829	0.355643	-0.20354	0.095977	0.376401
O	-3.08712	0.432385	1.779073	-2.41288	0.064424	1.978261	-2.36394	0.040438	2.014484
C	-2.26017	-2.21321	0.677076	-1.17726	-2.20466	0.44661	-1.26613	-2.16036	0.475422
O	-1.69583	2.812891	-0.8996	-1.9005	2.917111	-0.75329	-1.86172	2.920841	-0.74282
O	4.539675	-0.8234	-0.48396	2.3137	-1.46229	0.705079	2.345874	-1.45612	0.75046
H	5.145766	0.455022	-0.29798	1.585155	-2.12763	-0.33807	1.542148	-2.11877	-0.23613
H	-3.40734	0.785245	-0.31	-3.2118	0.448467	0.025279	-3.19208	0.519943	0.091956
H	-2.00496	-2.34241	-1.76306	-1.5206	-2.15742	-1.99857	-1.73403	-2.07916	-2.08541
H	-3.03867	-1.01099	-2.27846	-2.90103	-1.0667	-2.11624	-3.00522	-0.85714	-2.0829
H	-1.17905	0.36249	-2.29037	-1.40408	0.694149	-2.35043	-1.29957	0.713277	-2.35045
H	-0.17527	-0.83858	-1.49936	-0.03329	-0.30819	-1.91641	-0.05067	-0.41274	-1.86046
H	0.958751	3.009605	-0.5686	0.724901	3.495216	-0.65798	0.769432	3.472731	-0.66024
H	0.652482	1.715977	-1.72904	0.540255	2.182433	-1.82849	0.56424	2.160321	-1.82723
H	1.768671	1.298811	1.125672	1.95095	1.978867	0.912692	1.989547	1.945562	0.904624
H	2.189186	-0.10317	-1.56175	1.988265	0.262148	-1.61642	1.998906	0.234827	-1.62858
H	-0.45075	1.73028	1.828433	-0.24188	1.87977	1.765178	-0.21163	1.873227	1.77606
H	-4.55596	-2.70121	-0.62262	-3.51864	-3.23004	-0.38185	-3.67966	-3.12296	-0.39809
H	-4.89982	-1.72212	0.809909	-3.73833	-2.46908	1.20007	-3.79413	-2.39181	1.211748
H	-5.04406	-1.00367	-0.80051	-4.44707	-1.72351	-0.23905	-4.52631	-1.5857	-0.18947
H	4.168049	-1.32798	2.087334	4.77093	-1.77999	-0.30398	4.751383	-1.85859	-0.35195
H	3.814618	0.394662	1.882192	3.667629	-1.51441	-1.66797	3.590154	-1.61584	-1.67191
H	2.498717	-0.74994	2.217371	4.826969	-0.25396	-1.21298	4.794948	-0.36629	-1.31568
H	3.500169	-3.01274	0.373443	4.45338	-0.60013	1.833876	4.550245	-0.61204	1.760659
H	2.718625	-2.49636	-1.13527	3.105597	0.511704	2.100955	3.24205	0.540587	2.049083
H	1.814175	-2.45163	0.38811	4.465942	1.000894	1.071229	4.56773	0.964106	0.947289
H	-4.10238	0.10414	2.023783	-3.25415	-0.52001	2.363481	-3.23924	-0.49443	2.394421
H	-3.01792	1.49778	2.003376	-2.5522	1.098196	2.295763	-2.42278	1.070496	2.368676
H	-2.39038	-0.08618	2.447584	-1.49693	-0.30196	2.456383	-1.47413	-0.42651	2.446613
H	-2.65306	-2.1991	1.55993	-1.31374	-2.32748	1.396069	-1.37169	-3.08984	0.231035
H	-2.68076	2.88274	-0.43369	-2.86552	2.710945	-0.28417	-2.82346	2.738963	-0.25758
H	-1.2635	3.816554	-0.9221	-1.67935	3.978962	-0.61804	-1.62182	3.981065	-0.62837
H	-1.81917	2.471648	-1.93003	-1.98304	2.721806	-1.82413	-1.96205	2.709143	-1.80915
H	4.763609	0.976086	-1.02426	0.656522	-2.01253	-0.03802	0.632121	-1.91848	0.072771
	5b-13			5b-14			5b-15		
C	-1.15592	0.356002	-0.26016	-1.17291	0.311463	0.003578	-1.41996	0.377099	-0.14642
C	-2.13066	-0.4792	0.614987	-2.54476	0.012805	0.664257	-2.6708	-0.03248	0.653725
C	-3.36941	-0.71841	-0.27018	-3.21215	-1.0483	-0.2444	-3.3141	-1.10956	-0.24923
C	-3.44278	0.570019	-1.09431	-2.71356	-0.66008	-1.63282	-3.16832	-0.51015	-1.6501

C	-1.98643	0.835985	-1.47954	-1.23883	-0.31366	-1.41428	-1.89209	0.356769	-1.62005
C	-0.43317	1.4919	0.50546	-0.76375	1.797233	0.011251	-0.69881	1.673623	0.286028
C	0.67532	2.144354	-0.3745	0.677461	1.975348	-0.53019	0.443575	2.027975	-0.70404
C	1.678841	1.125075	-0.82598	1.680052	1.077138	0.131037	1.428764	0.906696	-0.87487
C	2.757573	0.800756	-0.11176	2.363023	0.126002	-0.5076	2.57904	0.820465	-0.20269
C	3.658237	-0.38707	-0.33496	3.314865	-0.87289	0.107676	3.59087	-0.29857	-0.29717
O	0.167334	0.960769	1.688086	-0.7951	2.194063	1.390645	-0.12759	1.506593	1.588288
C	-4.64129	-1.01861	0.514929	-4.73178	-1.0872	-0.12582	-4.75677	-1.4467	0.111738
C	3.260154	-1.2754	-1.50839	3.539474	-0.70218	1.605384	3.158196	-1.4614	-1.18283
C	5.121356	0.041741	-0.43612	2.837899	-2.29047	-0.21567	4.943807	0.266309	-0.73482
C	-0.37427	-0.32563	-0.61222	-0.405	-0.21166	0.58057	-0.69464	-0.43739	-0.01987
O	-1.51751	-1.75426	1.187882	-2.44371	-0.38963	2.133281	-2.41883	-0.47025	2.093189
C	-3.05742	-1.82478	-1.13321	-2.67306	-2.35615	0.005901	-2.52419	-2.31057	-0.25036
O	-1.37603	2.589997	0.992442	-1.70737	2.694655	-0.79574	-1.63957	2.867061	0.419332
O	3.649396	-1.1618	0.897628	4.579294	-0.80426	-0.60156	3.654118	-0.72512	1.091038
H	2.304912	-1.5016	1.246118	5.196809	0.460994	-0.36874	4.674533	-1.71566	1.250318
H	-2.46966	0.133443	1.456357	-3.17742	0.904807	0.609398	-3.37762	0.807379	0.663344
H	-4.11358	0.47726	-1.95511	-2.87262	-1.46221	-2.35803	-3.13076	-1.30542	-2.39759
H	-3.82683	1.37873	-0.46253	-3.26863	0.22092	-1.97289	-4.04556	0.104531	-1.86856
H	-1.81659	1.884228	-1.73583	-0.85592	0.341939	-2.19984	-2.11676	1.365213	-1.97798
H	-1.72342	0.247616	-2.36151	-0.63393	-1.22271	-1.44186	-1.11609	-0.04179	-2.27776
H	1.161415	2.909272	0.238025	0.960558	3.023471	-0.36686	0.950016	2.911927	-0.30591
H	0.217958	2.647053	-1.23129	0.689451	1.815386	-1.61184	0.015568	2.300733	-1.6732
H	1.431757	0.552152	-1.71711	1.797876	1.203221	1.205316	1.14666	0.108781	-1.55854
H	2.995933	1.376105	0.783211	2.217872	-0.001	-1.58213	2.860042	1.624488	0.477483
H	0.811371	0.282864	1.423174	-0.59766	3.139648	1.431754	0.540255	0.80721	1.530029
H	-5.48965	-1.15853	-0.16387	-5.14335	-1.83032	-0.81393	-5.17406	-2.13983	-0.62366
H	-4.5252	-1.93299	1.102737	-5.04243	-1.35643	0.889564	-4.82048	-1.92208	1.096067
H	-4.88598	-0.19712	1.193738	-5.1691	-0.11343	-0.36233	-5.37689	-0.54647	0.132443
H	3.926142	-2.14061	-1.55513	4.268375	-1.44019	1.948384	3.921279	-2.24267	-1.17131
H	2.231218	-1.63034	-1.41294	3.919187	0.292886	1.841592	2.212392	-1.88801	-0.83874
H	3.34413	-0.72933	-2.45075	2.606143	-0.86093	2.150686	3.03937	-1.13371	-2.21798
H	5.777938	-0.83132	-0.47285	3.550569	-3.02908	0.159665	5.709457	-0.51164	-0.7199
H	5.404786	0.65832	0.419674	2.724864	-2.4285	-1.29368	5.25425	1.07508	-0.06889
H	5.264004	0.624818	-1.34795	1.869216	-2.46317	0.257882	4.866489	0.661419	-1.75045
H	-2.25762	-2.33563	1.746416	-3.42334	-0.64775	2.550816	-3.32895	-0.86637	2.555418
H	-0.70309	-1.50662	1.872373	-2.03488	0.435102	2.719094	-2.07016	0.368695	2.696179
H	-1.12415	-2.38999	0.390781	-1.77559	-1.24745	2.263855	-1.64155	-1.24069	2.143031
H	-3.77751	-1.90696	-1.77211	-2.93635	-2.62103	0.896928	-2.55806	-2.68918	0.638188
H	-2.14194	2.190048	1.660366	-2.71617	2.682992	-0.37843	-2.33912	2.71251	1.243602
H	-0.80766	3.339387	1.549159	-1.35045	3.729978	-0.77637	-1.06697	3.773542	0.631182
H	-1.87184	3.089696	0.157266	-1.762	2.386748	-1.84282	-2.21196	3.029274	-0.49695
H	2.238859	-2.42399	0.950238	4.793425	1.020178	-1.05406	4.148818	-2.52898	1.315293

Table S 34. Energy analysis for 2*S*, 3*R*, 6*R*, 7*S*-5 in the Chloroform at MPW1PW91/6-31G+d, p level

Conf.	Steric Energy (kJ/mol)	Relative Energy (kJ/mol)	Distribution (%)
5b-1	-888.723671	0	12.91%
5b-2	-888.723149	0.000522	7.43%
5b-3	-888.72367	1E-06	12.89%
5b-4	-888.72302	0.000651	6.48%
5b-5	-888.72221	0.001461	2.75%
5b-6	-888.723128	0.000543	7.26%
5b-7	-888.72293	0.000741	5.89%
5b-8	-888.722312	0.001359	3.06%
5b-9	-888.72367	1E-06	12.89%
5b-10	-888.72343	0.000241	10.00%
5b-11	-888.722509	0.001162	3.77%
5b-12	-888.721545	0.002126	1.36%
5b-13	-888.722108	0.001563	2.47%
5b-14	-888.723278	0.000393	8.51%
5b-15	-888.721532	0.002139	1.34%

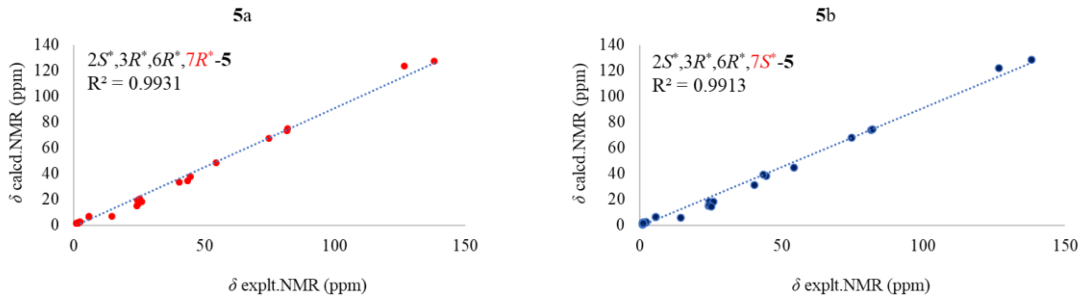

Figure S 93. Correlation between the calculated ^{13}C NMR data for 2*S*, 3*R*, 6*R*, 7*R*-5 (a) and 2*S*, 3*R*, 6*R*, 7*S*-5 (b) and experimental ^{13}C NMR data of 5

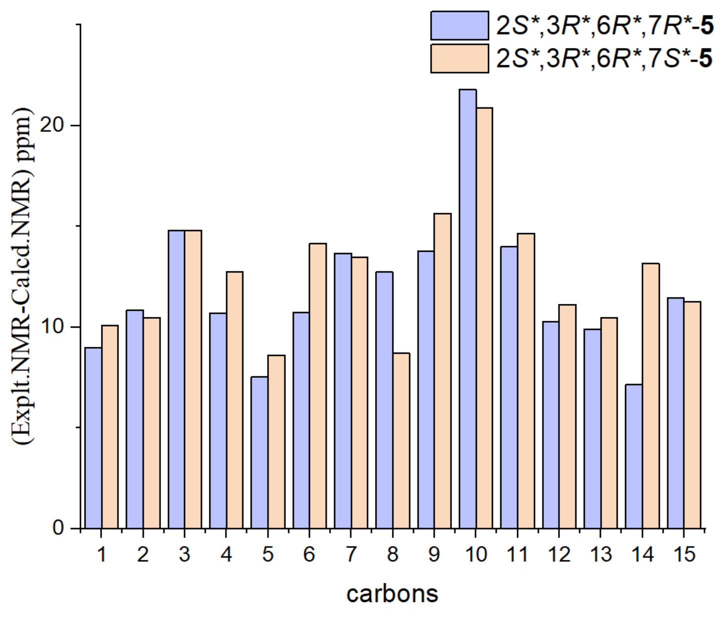

Figure S 94. Differences between experimental NMR chemical shifts of 5 and theoretical NMR chemical shifts for 2*S*, 3*R*, 6*R*, 7*R*-5 (purple bar) and 2*S*, 3*R*, 6*R*, 7*S*-5 (yellow bar)

Table S 35. Experimental chemical shifts of 5, the calculated shielding tensors for 2*S*, 3*R*, 6*R*, 7*R*-5 (isomer 1) and 2*S*, 3*R*, 6*R*, 7*S*-5 (isomer 2), as well as their DP4+ probability

	A	B	C	D	E	F	G	H
1	Functional		Solvent?		Basis Set		Type of Data	
2	mPW1PW91		PCM		6-31+G(d,p)		Shielding Tensors	
3								
12			DP4+	100.00%	0.00%	-	-	-
14	Nuclei	sp2?	xperimenta	Isomer 1	Isomer 2	Isomer 3	Isomer 4	Isomer 5
15	C		54.4	140.3	143.7			
16	C		44.6	151	150.4			
17	C		81.6	115.1	114.8			
18	C		40.4	155.5	157.4			
19	C		24.5	169.3	170.2			
20	C		74.8	121.3	120.8			
21	C		43.6	154.2	149.6			
22	C	x	126.7	65	66.7			
23	C	x	138.1	61.4	60			
24	C		82	113.8	114.3			
25	C		26.1	170.1	170.5			
26	C		24.2	173.9	173.5			
27	C		24.6	172.2	172.9			
28	C		14.5	181.8	182.7			
29	C		25.2	168.1	174.4			
30	H		1.86	29.71	29.6			
31	H		1.6	29.84	29.95			
32	H		1.68	29.98	30.01			
33	H		1.56	30.12	30.07			
34	H		1.57	30.18	30.45			
35	H		1.86	29.73	29.73			
36	H		2.23	29.51	29.63			
37	H		2.23	29.37	29.37			
38	H	x	5.74	25.18	25.3			
39	H	x	5.63	25.71	25.57			
40	H		1.25	30.8	30.35			
41	H		1.25	30.33	30.44			
42	H		1.25	30.43	30.85			
43	H		1.33	30.5	30.5			
44	H		1.33	30.47	29.97			
45	H		1.33	29.9	30.45			
46	H		1.32	30.47	30.48			
47	H		1.32	30.52	30.04			
48	H		1.32	30.11	30.54			
49	H		1.04	30.21	30.78			
50	H		1.04	30.88	30.01			
51	H		1.04	30.71	31.21			
52	H		1.14	30.66	30.4			
53	H		1.14	30.54	30.73			
54	H		1.14	30.61	30.57			

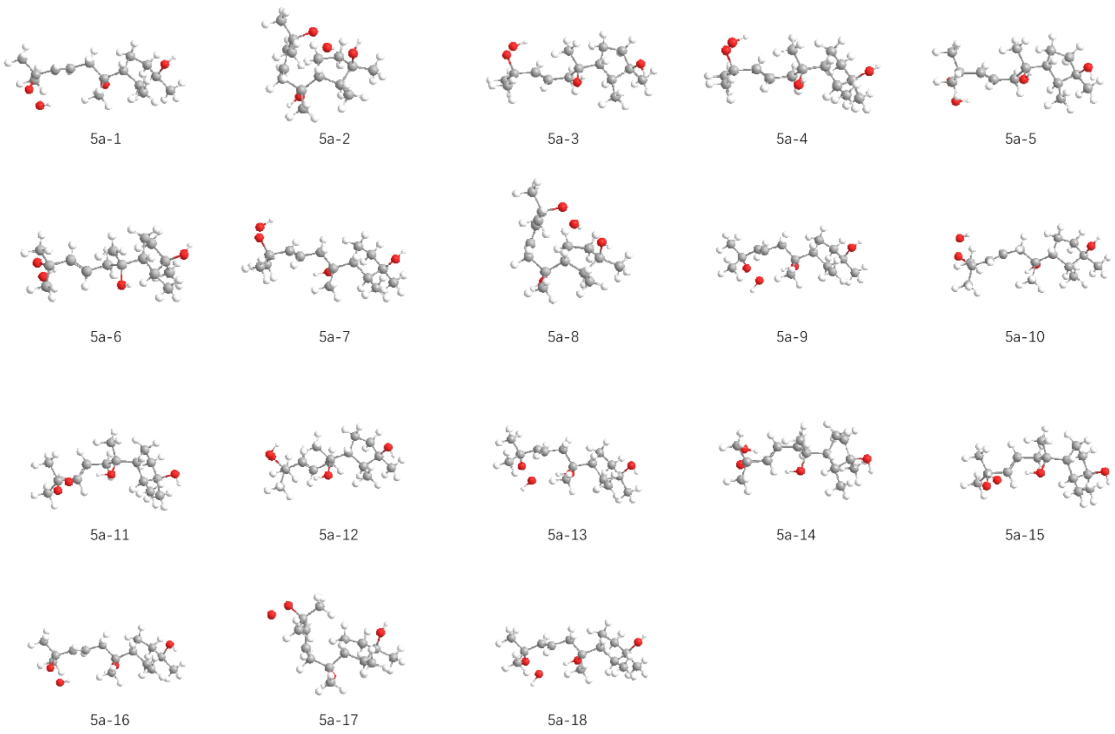

Figure S 95. Most stable conformers of 2*S*, 3*R*, 6*R*, 7*R*-5 at the CAM-B3LYP/DGDZVP level

Table S 36. Optimized Z-Matrixes of 2*S*, 3*R*, 6*R*, 7*R*-5 conformations in the methanol at CAM-B3LYP/DGDZVP level

	5a-1			5a-2			5a-3		
C	1.84712	-0.18919	-0.56599	-1.0163	0.574646	-0.13407	1.920837	0.563067	0.399127
C	2.961359	0.748297	-0.02265	-2.22652	-0.06857	0.582856	2.758101	-0.68947	0.082648
C	4.198459	-0.14989	0.17453	-2.44861	-1.35217	-0.24324	4.006935	-0.09643	-0.60915
C	3.576281	-1.47977	0.600097	-2.30356	-0.85076	-1.67725	3.415479	0.986397	-1.51824
C	2.366328	-1.63427	-0.32165	-1.11546	0.115378	-1.61626	2.108458	1.459316	-0.84675
C	0.448817	0.053938	0.042926	-0.75533	2.083188	0.00179	0.452983	0.358982	0.834396
C	-0.59527	-0.78781	-0.73595	0.630482	2.407183	-0.63512	-0.29749	-0.61048	-0.09426
C	-1.95863	-0.79916	-0.10816	1.736521	1.575806	-0.04524	-1.76521	-0.70491	0.204309
C	-3.05744	-0.29865	-0.6753	2.259546	0.513911	-0.66057	-2.72946	-0.43199	-0.67586
C	-4.45341	-0.29776	-0.09683	3.177853	-0.52894	-0.07157	-4.21941	-0.48142	-0.42696
O	0.51895	-0.38608	1.408272	-1.77313	2.767714	-0.74058	0.408375	-0.26427	2.129746
C	5.209896	0.401932	1.173172	-3.75883	-2.07705	0.027088	4.846006	-1.12062	-1.3656
C	-5.40614	-1.04574	-1.03176	4.344831	-0.80299	-1.02079	-4.90466	-1.32481	-1.50258
C	-4.55568	-0.83004	1.327669	3.679493	-0.217	1.332801	-4.62076	-0.94349	0.968503
C	3.233048	1.978891	-0.88565	-2.03805	-0.35106	2.073974	3.054348	-1.60566	1.265755
O	4.821956	-0.29776	-1.11378	-1.3359	-2.22989	0.064683	4.835727	0.593714	0.343293
C	0.041369	1.528229	0.007965	-0.78889	2.57203	1.451117	-0.25706	1.715137	0.92173
O	-4.97107	1.05834	-0.16034	2.472468	-1.80311	-0.0857	-4.75917	0.844421	-0.68962
O	-4.1985	1.914194	0.682508	1.414063	-1.79442	0.879202	-4.24666	1.778648	0.259881
H	1.744557	-0.0191	-1.64333	-0.1375	0.106631	0.308991	2.41392	1.06706	1.242057
H	2.671879	1.075332	0.982716	-3.11937	0.552592	0.440456	2.242714	-1.27327	-0.69101

H	3.249594	-1.40408	1.642055	-3.21985	-0.32408	-1.96208	3.20913	0.55632	-2.50151
H	4.287229	-2.31	0.531744	-2.16224	-1.67173	-2.387	4.140306	1.790988	-1.66012
H	1.612611	-2.28492	0.124022	-1.26084	0.958526	-2.29262	1.271011	1.34494	-1.54075
H	2.669772	-2.08529	-1.26927	-0.195	-0.38964	-1.91841	2.1442	2.515146	-0.57092
H	-0.6575	-0.40398	-1.75907	0.562869	2.230698	-1.71159	-0.1485	-0.31062	-1.1353
H	-0.23507	-1.81931	-0.79801	0.829459	3.475846	-0.49076	0.151999	-1.60154	0.029147
H	-2.02891	-1.26501	0.873511	2.031017	1.806307	0.97716	-2.02316	-1.01198	1.215731
H	-2.9855	0.156876	-1.66455	1.943744	0.290637	-1.68023	-2.45552	-0.1163	-1.68408
H	-0.23433	-0.0144	1.885642	-1.63674	3.717948	-0.62574	0.874156	0.31204	2.75079
H	4.75241	0.543549	2.155993	-4.60782	-1.4059	-0.12631	4.235405	-1.67212	-2.08543
H	5.60815	1.361683	0.832945	-3.79583	-2.45833	1.050224	5.300763	-1.84542	-0.68265
H	6.050062	-0.29089	1.288162	-3.87364	-2.92627	-0.65447	5.651531	-0.61707	-1.90722
H	-6.43488	-0.96902	-0.67056	4.970811	-1.61282	-0.63799	-5.99052	-1.28974	-1.38342
H	-5.12532	-2.1	-1.06596	4.955374	0.097559	-1.11327	-4.57686	-2.36222	-1.41203
H	-5.35791	-0.64073	-2.04538	3.983146	-1.07794	-2.01478	-4.64874	-0.9667	-2.5027
H	-5.58788	-0.74077	1.674579	4.310493	-1.03584	1.687002	-5.70904	-0.91433	1.061691
H	-4.27399	-1.8848	1.362736	4.275644	0.69858	1.323924	-4.28859	-1.97003	1.138761
H	-3.9105	-0.2733	2.009239	2.854376	-0.08904	2.034577	-4.19066	-0.30448	1.741074
H	2.354356	2.624575	-0.94529	-2.03563	0.563395	2.667009	2.140579	-2.0804	1.625679
H	3.505663	1.684911	-1.90192	-1.09421	-0.87235	2.255338	3.474259	-1.04397	2.107005
H	4.052964	2.576958	-0.47624	-2.84717	-0.97913	2.457687	3.760035	-2.39646	0.991485
H	5.503363	-0.97779	-1.02727	-1.41471	-3.01186	-0.49884	5.195649	-0.06274	0.9551
H	0.713819	2.136557	0.615884	-1.80713	2.526506	1.84184	0.341043	2.420746	1.507873
H	0.045042	1.913531	-1.01499	-0.14054	1.974737	2.097032	-0.42325	2.151524	-0.06659
H	-0.9716	1.650885	0.404953	-0.45357	3.613401	1.50732	-1.22635	1.597525	1.412497
H	-3.52539	2.254814	0.06981	0.601636	-1.84241	0.331361	-3.38626	2.021306	-0.12396
	5a-4			5a-5			5a-6		
C	-1.96677	-0.81947	-0.42588	-2.00833	-0.74948	-0.01875	-2.06759	-0.90595	-0.37197
C	-2.88477	-0.19384	0.661063	-2.72096	0.52026	0.482505	-2.85562	-0.0976	0.696633
C	-3.77169	0.826858	-0.08022	-3.98461	0.574927	-0.40608	-3.69775	0.928167	-0.08953
C	-2.85029	1.324873	-1.19675	-3.44522	0.199125	-1.78911	-2.82159	1.219495	-1.31088
C	-2.16336	0.052394	-1.69444	-2.2359	-0.72905	-1.54933	-2.29255	-0.15784	-1.71238
C	-0.489	-0.99277	0.006354	-0.54187	-0.97205	0.413102	-0.57849	-1.1485	-0.01687
C	0.219823	0.353255	0.244448	0.32263	0.281313	0.195245	0.2443	0.153531	0.000208
C	1.667283	0.204014	0.614455	1.783191	0.05777	0.459015	1.682177	-0.05118	0.381567
C	2.672923	0.76843	-0.05562	2.743696	0.221446	-0.45199	2.715212	0.238441	-0.41108
C	4.146895	0.643668	0.253298	4.223706	-0.01382	-0.25689	4.179501	0.035634	-0.09734
O	-0.43927	-1.66844	1.274234	-0.48019	-1.21924	1.828774	-0.48645	-1.66697	1.320634
C	-4.30722	1.939357	0.814224	-4.70281	1.919846	-0.38748	-4.05953	2.170294	0.717099
C	4.775769	2.031509	0.384199	4.62063	-0.46738	1.142822	4.46473	-0.53104	1.288262
C	4.476653	-0.22256	1.462875	4.733611	-0.99454	-1.31433	4.824923	-0.8262	-1.18403
C	-3.66422	-1.21533	1.484832	-2.98869	0.580162	1.983179	-3.66468	-0.96112	1.660889
O	-4.86825	0.093096	-0.65231	-4.9073	-0.46751	-0.04299	-4.89811	0.255947	-0.50841

C	0.268337	-1.82987	-1.03057	0.03668	-2.18774	-0.31994	0.034946	-2.16438	-0.98608
O	4.809373	0.120547	-0.93183	4.930941	1.207352	-0.60797	4.864433	1.306544	-0.26991
O	4.358143	-1.20721	-1.19849	4.602149	2.24505	0.314894	4.406174	2.244034	0.703801
H	-2.3309	-1.83272	-0.63654	-2.56897	-1.60469	0.383623	-2.51764	-1.90373	-0.44659
H	-2.26926	0.387838	1.354481	-2.12483	1.397197	0.199056	-2.1473	0.485265	1.293821
H	-2.11721	2.020345	-0.77337	-4.23266	-0.26555	-2.38667	-3.37689	1.72396	-2.10834
H	-3.39843	1.860186	-1.97887	-3.13383	1.108556	-2.30936	-1.99882	1.879195	-1.01438
H	-1.23271	0.266316	-2.22474	-1.35844	-0.3407	-2.07355	-1.39562	-0.09233	-2.33231
H	-2.81549	-0.46625	-2.4003	-2.4077	-1.73685	-1.93269	-3.04638	-0.6824	-2.30334
H	0.133524	0.975436	-0.6497	0.181673	0.650419	-0.82448	0.190127	0.628311	-0.98253
H	-0.29661	0.876175	1.056344	-0.04162	1.056082	0.878162	-0.20959	0.842345	0.720585
H	1.872406	-0.41357	1.486096	2.037706	-0.2743	1.463352	1.855511	-0.47624	1.367394
H	2.452187	1.379471	-0.93273	2.472102	0.545762	-1.45817	2.525497	0.654901	-1.40201
H	-0.81156	-2.55237	1.151905	-1.02116	-1.99847	2.015906	-0.94076	-2.52024	1.337372
H	-3.48853	2.500539	1.27276	-4.01829	2.733084	-0.64282	-3.16059	2.693367	1.054318
H	-4.93467	1.527646	1.609422	-5.12585	2.131552	0.599827	-4.65475	1.90114	1.593988
H	-4.9137	2.641526	0.23268	-5.52257	1.915957	-1.11117	-4.6473	2.865709	0.108877
H	5.858155	1.951353	0.513089	5.706661	-0.5756	1.194937	5.544997	-0.60577	1.43443
H	4.356779	2.537472	1.256101	4.168276	-1.43517	1.371014	4.035549	-1.5308	1.385541
H	4.570858	2.63892	-0.50067	4.307726	0.251709	1.901235	4.052047	0.104167	2.073371
H	5.561192	-0.28314	1.581235	5.817483	-1.11209	-1.23721	5.901322	-0.91315	-1.01563
H	4.056518	0.216133	2.370699	4.264443	-1.96824	-1.16006	4.385596	-1.82543	-1.16068
H	4.083869	-1.23412	1.34972	4.48802	-0.64753	-2.3209	4.657205	-0.39726	-2.17489
H	-2.97667	-1.84656	2.052758	-2.05354	0.658037	2.539341	-2.99822	-1.59084	2.255318
H	-4.27174	-1.85653	0.841793	-3.49307	-0.32707	2.333057	-4.35978	-1.60789	1.120355
H	-4.33211	-0.72369	2.198926	-3.61021	1.442925	2.243701	-4.24599	-0.34559	2.354346
H	-5.34884	0.697487	-1.23379	-5.23216	-0.2794	0.847942	-5.36443	0.84831	-1.11338
H	-0.262	-2.76756	-1.22566	-0.63808	-3.04502	-0.22462	-0.55378	-3.08741	-0.9923
H	0.376121	-1.29984	-1.98041	0.184291	-1.98875	-1.38427	0.066827	-1.77969	-2.00859
H	1.267869	-2.072	-0.66148	1.003046	-2.45983	0.110789	1.056119	-2.40843	-0.68443
H	3.548833	-1.05551	-1.71625	3.772296	2.598041	-0.04967	3.610629	2.613514	0.283714
	5a-7			5a-8			5a-9		
C	1.864553	0.168311	-0.67366	-1.02372	0.566603	-0.11764	1.777227	0.438796	-0.67786
C	3.081865	0.63032	0.177561	-2.28624	-0.04399	0.532906	2.873503	0.52717	0.422032
C	4.033394	-0.58133	0.232932	-2.44941	-1.36881	-0.25269	3.825376	-0.65585	0.155933
C	3.064444	-1.76345	0.241527	-2.18701	-0.93782	-1.68795	2.877081	-1.73068	-0.37505
C	1.998857	-1.37582	-0.78365	-1.02046	0.051445	-1.58408	1.944248	-0.96816	-1.31572
C	0.492091	0.616197	-0.12423	-0.77234	2.080153	-0.02312	0.340777	0.703146	-0.1746
C	-0.61157	0.253357	-1.15514	0.633283	2.393386	-0.61852	-0.61903	0.779447	-1.39927
C	-2.00025	0.556647	-0.66687	1.717234	1.552845	-0.00264	-2.05315	0.86674	-0.9656
C	-2.79326	-0.34932	-0.08816	2.30185	0.533455	-0.634	-2.81744	-0.21074	-0.77869
C	-4.16986	-0.11258	0.488844	3.216058	-0.50883	-0.03753	-4.17639	-0.25065	-0.12762
O	0.282142	-0.11203	1.09069	-1.76568	2.732887	-0.82496	-0.00924	-0.40232	0.662469

C	4.988135	-0.56164	1.421725	-3.78429	-2.07341	-0.06067	4.624296	-1.09155	1.379463
C	-4.19631	-0.55656	1.95269	4.43074	-0.72668	-0.94005	-4.69388	1.100586	0.351689
C	-4.68904	1.312592	0.341776	3.645962	-0.23472	1.398279	-5.19505	-0.94371	-1.03177
C	3.75337	1.909073	-0.31956	-2.20747	-0.26163	2.044918	3.58545	1.87638	0.49974
O	4.786534	-0.58243	-0.99291	-1.3695	-2.27565	0.078944	4.725536	-0.24634	-0.88932
C	0.445917	2.11814	0.177059	-0.86387	2.614341	1.408026	0.236632	1.998258	0.639342
O	-5.09038	-1.04936	-0.13129	2.541066	-1.79672	-0.12461	-4.08374	-1.17503	0.994356
O	-5.22023	-0.756	-1.52219	1.432975	-1.83464	0.780952	-3.04568	-0.7576	1.884361
H	1.952245	0.612307	-1.67128	-0.17913	0.112779	0.404058	1.981876	1.206264	-1.43252
H	2.742057	0.786078	1.207784	-3.16291	0.573616	0.301922	2.405856	0.316949	1.390568
H	2.615839	-1.85094	1.23616	-3.08292	-0.441	-2.07127	2.308159	-2.15048	0.46057
H	3.565436	-2.71079	0.01579	-1.97773	-1.79659	-2.33046	3.413601	-2.55259	-0.86106
H	1.060271	-1.8967	-0.59022	-1.1373	0.868041	-2.29781	0.995094	-1.49166	-1.43718
H	2.326086	-1.64964	-1.7894	-0.0729	-0.44277	-1.81208	2.399474	-0.87685	-2.30477
H	-0.41451	0.810862	-2.07621	0.596384	2.220889	-1.69701	-0.34044	1.650368	-2.0003
H	-0.53658	-0.81036	-1.39099	0.83719	3.460102	-0.46575	-0.47609	-0.1097	-2.01709
H	-2.34363	1.584607	-0.7596	1.948845	1.742925	1.044075	-2.43246	1.8493	-0.69436
H	-2.44561	-1.38069	-0.00478	2.051406	0.349976	-1.6794	-2.42617	-1.19228	-1.04884
H	-0.62377	0.063618	1.385168	-1.63301	3.68714	-0.74394	-0.94753	-0.32023	0.902026
H	4.437015	-0.54123	2.365731	-4.61149	-1.39812	-0.29331	3.959552	-1.38753	2.19546
H	5.641341	0.314274	1.381237	-3.9142	-2.42283	0.968357	5.268152	-0.28113	1.731875
H	5.620524	-1.45565	1.418976	-3.84761	-2.94165	-0.72191	5.261464	-1.94805	1.135445
H	-5.20852	-0.47772	2.357042	5.056143	-1.53623	-0.55577	-5.64975	0.965879	0.863735
H	-3.5359	0.086107	2.538131	5.025193	0.188633	-0.97388	-4.85197	1.772084	-0.49507
H	-3.85559	-1.58957	2.055205	4.120999	-0.97473	-1.95833	-3.99352	1.580908	1.038727
H	-5.6997	1.373802	0.752191	4.282716	-1.04934	1.751905	-6.14619	-1.07649	-0.50994
H	-4.05291	2.008176	0.893853	4.216305	0.695746	1.448871	-5.3669	-0.32984	-1.91787
H	-4.7206	1.621986	-0.70393	2.787031	-0.1543	2.065751	-4.82912	-1.92193	-1.35183
H	3.068154	2.758526	-0.28035	-2.27372	0.673221	2.600642	2.888896	2.678033	0.754606
H	4.087182	1.792505	-1.35319	-1.26252	-0.73839	2.326166	4.049013	2.124051	-0.45808
H	4.626212	2.161311	0.290459	-3.02755	-0.89554	2.396589	4.370562	1.867331	1.262148
H	5.268696	-1.41909	-1.03389	-1.53218	-2.62664	0.965371	5.216772	-1.02996	-1.17013
H	1.173763	2.382072	0.946334	-1.89793	2.58602	1.755921	0.857961	1.943616	1.535113
H	0.651205	2.709581	-0.71966	-0.24745	2.03447	2.099662	0.544855	2.866324	0.049591
H	-0.54087	2.404368	0.552774	-0.52606	3.655514	1.446608	-0.79368	2.160792	0.969534
H	-4.49639	-1.27297	-1.91489	0.651537	-1.85603	0.191021	-3.54054	-0.29796	2.582578
	5a-10			5a-11			5a-12		
C	1.870775	-0.15896	-0.5544	1.904529	0.982587	0.202394	-1.97934	-0.86257	-0.39337
C	3.059651	0.721881	-0.07782	2.40215	-0.41194	0.672602	-2.86109	-0.16736	0.681409
C	4.223349	-0.25614	0.17761	3.606277	-0.76201	-0.23756	-3.78325	0.806065	-0.09497
C	3.501106	-1.50144	0.691517	3.261703	-0.05552	-1.54573	-2.92358	1.208083	-1.28999
C	2.278184	-1.6212	-0.21806	2.691041	1.291077	-1.09718	-2.25199	-0.09889	-1.71501
C	0.502135	0.231385	0.043716	0.370491	1.088558	0.055602	-0.48495	-0.96763	-0.01654

C	-0.61358	-0.57629	-0.67267	-0.17093	0.201479	-1.10136	0.204903	0.417481	0.035349
C	-1.94549	-0.50906	0.016814	-1.67134	0.220719	-1.13631	1.628149	0.366491	0.51247
C	-3.00254	0.162136	-0.44413	-2.42315	-0.68028	-0.50204	2.683797	0.694407	-0.23393
C	-4.35297	0.292876	0.221415	-3.91363	-0.61806	-0.28541	4.133687	0.675375	0.190832
O	0.553539	-0.11859	1.435379	-0.16303	0.630643	1.302443	-0.46674	-1.56257	1.292034
C	5.281176	0.278951	1.136544	3.83516	-2.26162	-0.39136	-4.26315	1.986416	0.742366
C	-4.49598	-0.46651	1.53489	-4.59299	0.612637	-0.87466	4.736667	2.074662	0.050139
C	-4.69348	1.773041	0.405035	-4.5884	-1.90358	-0.76238	4.384417	0.10951	1.583551
C	3.421779	1.867344	-1.02107	2.70157	-0.4823	2.167672	-3.60109	-1.14321	1.592681
O	4.824904	-0.53804	-1.09865	4.812897	-0.13563	0.231676	-4.91566	0.116084	-0.65117
C	0.207914	1.727123	-0.09004	-0.03968	2.553262	-0.14728	0.239989	-1.89618	-0.9946
O	-5.37034	-0.13955	-0.72185	-4.13732	-0.66973	1.152877	4.886952	-0.07847	-0.79656
O	-5.22747	-1.53459	-0.9879	-3.43314	0.39703	1.793219	4.461645	-1.44202	-0.79689
H	1.770261	-0.05145	-1.64006	2.173224	1.718804	0.967643	-2.3227	-1.89697	-0.49968
H	2.804193	1.13762	0.903563	1.628282	-1.15697	0.46306	-2.22601	0.454656	1.320154
H	3.186476	-1.33067	1.725948	4.134184	0.032941	-2.19858	-2.17595	1.937009	-0.96028
H	4.144367	-2.38761	0.678927	2.501674	-0.63952	-2.0751	-3.52148	1.671201	-2.07945
H	1.478707	-2.18205	0.268295	2.085418	1.76345	-1.874	-1.35595	0.073031	-2.31573
H	2.541707	-2.15388	-1.13474	3.509818	1.979027	-0.87527	-2.93915	-0.67798	-2.33555
H	-0.70167	-0.20501	-1.69846	0.235434	0.559219	-2.05131	0.171578	0.877783	-0.95583
H	-0.30836	-1.62508	-0.73182	0.177435	-0.82382	-0.95407	-0.3629	1.062598	0.713622
H	-2.02194	-1.04456	0.961564	-2.1428	1.074982	-1.61682	1.781179	0.049412	1.542962
H	-2.92067	0.693732	-1.39389	-1.93945	-1.52936	-0.01778	2.526392	1.02543	-1.26196
H	-0.19224	0.306529	1.879459	-1.13227	0.611711	1.229938	0.428716	-1.88185	1.466366
H	4.841972	0.521018	2.108038	2.948073	-2.75217	-0.80077	-3.41715	2.557939	1.133308
H	5.751863	1.179622	0.733179	4.061498	-2.72723	0.573972	-4.86286	1.64778	1.594126
H	6.064674	-0.46905	1.296936	4.677076	-2.44728	-1.06389	-4.88194	2.652471	0.134956
H	-5.51225	-0.34262	1.916425	-5.65421	0.604779	-0.61426	5.80853	2.052177	0.262796
H	-3.80045	-0.0726	2.279469	-4.50883	0.61191	-1.96364	4.253657	2.749336	0.759337
H	-4.30234	-1.53228	1.405029	-4.14824	1.537724	-0.50109	4.586141	2.466009	-0.95881
H	-5.7029	1.886243	0.808361	-5.64884	-1.90344	-0.49772	5.459332	0.088706	1.778157
H	-3.98538	2.222865	1.103712	-4.50047	-1.97548	-1.84802	3.912724	0.737998	2.342249
H	-4.63159	2.309806	-0.54462	-4.11399	-2.78054	-0.31614	3.99413	-0.9051	1.678046
H	2.597903	2.577448	-1.11838	1.787926	-0.30331	2.737484	-2.88376	-1.73549	2.163578
H	3.659756	1.48763	-2.01743	3.430696	0.278729	2.464232	-4.2174	-1.8387	1.013797
H	4.291377	2.421888	-0.65514	3.091262	-1.46464	2.456974	-4.2481	-0.61975	2.305326
H	5.451641	-1.2622	-0.968	5.046221	-0.53594	1.079874	-5.47312	-0.18077	0.080352
H	0.921101	2.319182	0.487049	0.375136	3.16797	0.655836	-0.25774	-2.86882	-1.02607
H	0.252105	2.04428	-1.13522	0.3122	2.948764	-1.10362	0.259387	-1.48216	-2.00563
H	-0.79676	1.952571	0.28227	-1.12827	2.65949	-0.12101	1.278224	-2.05134	-0.6845
H	-4.57891	-1.5461	-1.71242	-4.13025	1.061604	1.920291	3.777599	-1.44468	-1.48725
	5a-13			5a-14			5a-15		
C	1.775496	0.439296	-0.67393	-2.08289	-1.0125	-0.13631	1.905524	0.981653	0.204274

C	2.871741	0.51496	0.425748	-2.54958	0.15861	0.771555	2.402172	-0.41451	0.670604
C	3.8282	-0.66915	0.142418	-3.59136	0.948079	-0.06039	3.607466	-0.76157	-0.23903
C	2.885658	-1.73262	-0.40659	-3.10625	0.745292	-1.49301	3.263515	-0.05232	-1.54585
C	1.941524	-0.96014	-1.32857	-2.68904	-0.72591	-1.53412	2.692029	1.293062	-1.09469
C	0.339573	0.700466	-0.16805	-0.55525	-1.24041	-0.14358	0.371513	1.089498	0.058345
C	-0.62017	0.789002	-1.39183	0.212946	-0.06103	-0.79433	-0.17144	0.205652	-1.10032
C	-2.05401	0.875103	-0.95708	1.704153	-0.24281	-0.74751	-1.67188	0.225556	-1.13428
C	-2.82042	-0.20243	-0.77961	2.489884	0.318784	0.174982	-2.4237	-0.67775	-0.5033
C	-4.17934	-0.24516	-0.12863	3.981733	0.132987	0.328933	-3.91442	-0.61711	-0.28765
O	-0.01115	-0.41233	0.65875	-0.18043	-1.34282	1.235763	-0.16194	0.629239	1.304378
C	4.620998	-1.1221	1.363544	-3.71061	2.412484	0.347279	3.837873	-2.26062	-0.3959
C	-4.6935	1.102844	0.363281	4.293162	-0.36594	1.741335	-4.59386	0.615806	-0.87215
C	-5.19976	-0.9271	-1.03923	4.626147	-0.76374	-0.72121	-4.58798	-1.90088	-0.77106
C	3.583156	1.864141	0.513751	-3.04801	-0.29017	2.142853	2.699374	-0.49033	2.165844
O	4.732496	-0.3583	-0.93237	-4.88497	0.322811	-0.00163	4.813014	-0.1352	0.23296
C	0.237831	1.988303	0.657719	-0.22356	-2.56343	-0.84549	-0.03733	2.555071	-0.14091
O	-4.08869	-1.18018	0.984573	4.606858	1.44378	0.322891	-4.1396	-0.67539	1.150125
O	-3.04923	-0.77369	1.878039	4.443345	2.055197	-0.95659	-3.43761	0.389554	1.795914
H	1.980509	1.214597	-1.42052	-2.51074	-1.94133	0.256095	2.175187	1.715539	0.971421
H	2.406511	0.299333	1.394296	-1.70865	0.838713	0.939867	1.628522	-1.15868	0.457149
H	3.432748	-2.53469	-0.90953	-2.24449	1.396813	-1.67165	4.136347	0.037921	-2.19798
H	2.324176	-2.16868	0.425051	-3.88006	1.002097	-2.22129	2.503979	-0.63543	-2.07692
H	0.992237	-1.48455	-1.44582	-2.00706	-0.93984	-2.36029	2.086298	1.766549	-1.87072
H	2.385444	-0.85777	-2.32155	-3.57126	-1.35144	-1.68571	3.510319	1.981142	-0.87142
H	-0.34022	1.664568	-1.98543	-0.10294	0.042043	-1.8363	0.234321	0.565486	-2.04974
H	-0.47875	-0.09516	-2.0171	-0.05378	0.862256	-0.27393	0.17635	-0.82024	-0.95596
H	-2.43133	1.855922	-0.67694	2.140902	-0.90631	-1.49079	-2.14337	1.081498	-1.61178
H	-2.43132	-1.18236	-1.05856	2.044675	0.988013	0.913433	-1.94	-1.52858	-0.02213
H	-0.94967	-0.33245	0.898146	0.786668	-1.39237	1.27274	-1.13112	0.609889	1.231553
H	3.95242	-1.40241	2.181605	-2.74807	2.922879	0.256397	2.951466	-2.75122	-0.80673
H	5.28173	-0.32719	1.725998	-4.04464	2.507188	1.386205	4.064277	-2.72798	0.56858
H	5.240215	-1.98657	1.109011	-4.43679	2.923341	-0.29083	4.680296	-2.44407	-1.0684
H	-5.64946	0.965649	0.8745	5.373411	-0.42155	1.89688	-5.6553	0.606316	-0.61268
H	-4.85045	1.782392	-0.47725	3.869409	-1.36322	1.874874	-4.50881	0.619778	-1.96105
H	-3.99174	1.575257	1.05437	3.862618	0.29765	2.495003	-4.14986	1.539497	-0.49424
H	-6.15121	-1.06239	-0.51862	5.704144	-0.80628	-0.54886	-5.64872	-1.9024	-0.50767
H	-5.37008	-0.30447	-1.9195	4.228166	-1.77863	-0.65105	-4.4987	-1.96804	-1.85689
H	-4.83629	-1.90317	-1.3685	4.451201	-0.38831	-1.73051	-4.11368	-2.77954	-0.32806
H	2.897022	2.660049	0.809174	-2.23538	-0.76425	2.696332	1.784671	-0.3143	2.734853
H	4.007566	2.148393	-0.45442	-3.85747	-1.02187	2.05279	3.427476	0.270106	2.466463
H	4.392361	1.845074	1.251729	-3.41174	0.554858	2.737935	3.089413	-1.47346	2.451938
H	5.346536	0.319099	-0.61935	-5.21351	0.401058	0.903817	5.045417	-0.53703	1.080687
H	0.856472	1.922574	1.554638	-0.79602	-3.37672	-0.39215	0.378322	3.167453	0.663557

H	0.550576	2.86078	0.076842	-0.45617	-2.52893	-1.91287	0.314642	2.952527	-1.0964
H	-0.79255	2.151583	0.987147	0.83859	-2.80416	-0.74257	-1.12581	2.662282	-0.11405
H	-3.54273	-0.31982	2.580985	3.591652	2.514768	-0.86298	-4.13609	1.052093	1.925948
	5a-16			5a-17			5a-18		
C	1.846606	-0.18951	-0.56064	-1.18047	0.271222	-0.03068	1.777142	0.439339	-0.67769
C	2.963062	0.740262	-0.01103	-2.53765	-0.00958	0.674324	2.873447	0.526995	0.422251
C	4.204156	-0.16685	0.1727	-3.29558	-0.97453	-0.25924	3.825032	-0.65613	0.155637
C	3.583662	-1.49713	0.580667	-2.84302	-0.52038	-1.647	2.876495	-1.7305	-0.37585
C	2.360786	-1.63834	-0.32698	-1.34571	-0.2605	-1.48042	1.943913	-0.96732	-1.31623
C	0.447923	0.054145	0.046657	-0.72469	1.743282	0.028271	0.340743	0.703571	-0.17428
C	-0.59619	-0.78099	-0.73927	0.677704	1.919607	-0.61017	-0.61903	0.780702	-1.39891
C	-1.96031	-0.7963	-0.11318	1.769266	1.148383	0.074378	-2.05315	0.867557	-0.96517
C	-3.05785	-0.28956	-0.67728	2.327463	0.048437	-0.43348	-2.81723	-0.21015	-0.77877
C	-4.45502	-0.29448	-0.10171	3.362121	-0.83034	0.227955	-4.17613	-0.25067	-0.12762
O	0.51452	-0.39278	1.409718	-1.666	2.492335	-0.75487	-0.00943	-0.4024	0.662035
C	5.215543	0.372136	1.178453	-4.80846	-0.95499	-0.06982	4.62384	-1.09256	1.37898
C	-5.40548	-1.03302	-1.04647	3.874804	-0.31739	1.568337	-4.69378	1.100224	0.352524
C	-4.56028	-0.84175	1.316883	2.810626	-2.25176	0.359776	-5.1947	-0.94322	-1.03227
C	3.233999	1.976879	-0.86648	-2.41186	-0.52495	2.106979	3.58576	1.875981	0.500509
O	4.856221	-0.41164	-1.086	-2.79221	-2.29682	0.003102	4.725275	-0.24634	-0.88941
C	0.044683	1.529721	0.018174	-0.71323	2.290565	1.458775	0.236722	1.998192	0.640476
O	-4.97302	1.06199	-0.15233	4.468403	-1.01563	-0.69484	-4.08332	-1.1758	0.993706
O	-4.20273	1.909369	0.701151	5.130018	0.229784	-0.91684	-3.04513	-0.75896	1.883912
H	1.745931	-0.01247	-1.63712	-0.40322	-0.30894	0.475322	1.98186	1.207152	-1.43198
H	2.676802	1.06404	0.996377	-3.12125	0.917931	0.680609	2.405775	0.31647	1.390707
H	3.270318	-1.43255	1.626912	-3.35987	0.409365	-1.90557	2.307433	-2.15052	0.459561
H	4.297266	-2.32012	0.486734	-3.07637	-1.25964	-2.42068	3.412841	-2.55232	-0.8622
H	1.607966	-2.28461	0.126725	-0.98024	0.436901	-2.23496	0.994641	-1.49053	-1.43802
H	2.648442	-2.09001	-1.27908	-0.78481	-1.19123	-1.59849	2.399234	-0.87561	-2.3052
H	-0.65705	-0.38948	-1.75955	0.62471	1.634678	-1.66317	-0.34049	1.652074	-1.99931
H	-0.23674	-1.81229	-0.8086	0.919093	2.989498	-0.58051	-0.47602	-0.10801	-2.01734
H	-2.03247	-1.27172	0.863743	2.079666	1.505931	1.054401	-2.43259	1.8499	-0.69332
H	-2.98395	0.175328	-1.66201	2.00057	-0.30664	-1.41261	-2.42579	-1.19149	-1.0494
H	-0.24162	-0.02594	1.886345	-1.43943	3.429046	-0.67842	-0.9478	-0.32052	0.901319
H	4.750227	0.527125	2.155329	-5.21202	0.045366	-0.24791	3.959016	-1.38878	2.194823
H	5.633841	1.329381	0.84928	-5.07726	-1.26288	0.944385	5.267862	-0.28243	1.731779
H	6.041215	-0.33513	1.295129	-5.29079	-1.64391	-0.77118	5.260834	-1.94908	1.134567
H	-6.43498	-0.96068	-0.68657	4.648463	-0.99201	1.942678	-5.64956	0.965067	0.864626
H	-5.124	-2.08671	-1.0913	3.064163	-0.28761	2.299946	-4.85211	1.772177	-0.49384
H	-5.3554	-0.61723	-2.05563	4.300564	0.683085	1.477449	-3.9934	1.580268	1.039736
H	-5.59319	-0.75592	1.662561	3.573077	-2.92267	0.763674	-6.14582	-1.07648	-0.51052
H	-4.27891	-1.89688	1.3413	1.954766	-2.24543	1.037602	-5.36664	-0.32878	-1.91797
H	-3.9164	-0.29251	2.00573	2.481353	-2.63493	-0.60906	-4.82864	-1.92117	-1.35297

H	2.371125	2.644801	-0.8912	-1.93882	0.21571	2.755282	2.889465	2.677695	0.755876
H	3.454557	1.69824	-1.90186	-1.80873	-1.43547	2.138245	4.049251	2.123984	-0.45726
H	4.078733	2.556527	-0.47908	-3.39317	-0.75566	2.533072	4.370991	1.866346	1.262788
H	5.256499	0.416643	-1.38197	-3.14438	-2.87964	-0.68281	5.216322	-1.02995	-1.17057
H	0.714509	2.131933	0.635102	-1.72645	2.33632	1.862938	0.858267	1.943012	1.536068
H	0.056561	1.92155	-1.00229	-0.10449	1.670702	2.121917	0.544767	2.866623	0.051182
H	-0.97059	1.65273	0.409023	-0.29887	3.304527	1.473771	-0.79351	2.160483	0.971011
H	-3.53046	2.25913	0.092723	4.624166	0.607714	-1.65626	-3.5399	-0.29966	2.582427

Table S 37. Energy analysis for 2*S*, 3*R*, 6*R*, 7*R*-5

Conf.	Steric Energy (kJ/mol)	Relative Energy (kJ/mol)	Distribution (%)
5a-1	-888.725	0.001768	2.20%
5a-2	-888.725	0.001407	3.22%
5a-3	-888.726	0.000547	8.00%
5a-4	-888.726	0.000347	9.89%
5a-5	-888.726	0	14.28%
5a-6	-888.725	0.00101	4.90%
5a-7	-888.725	0.001598	2.63%
5a-8	-888.725	0.001448	3.08%
5a-9	-888.725	0.001584	2.67%
5a-10	-888.725	0.001738	2.27%
5a-11	-888.726	0.000512	8.30%
5a-12	-888.725	0.001738	2.27%
5a-13	-888.725	0.001145	4.25%
5a-14	-888.725	0.001247	3.81%
5a-15	-888.726	0.000242	11.05%
5a-16	-888.725	0.001297	3.62%
5a-17	-888.726	0.000256	10.89%
5a-18	-888.725	0.001586	2.66%

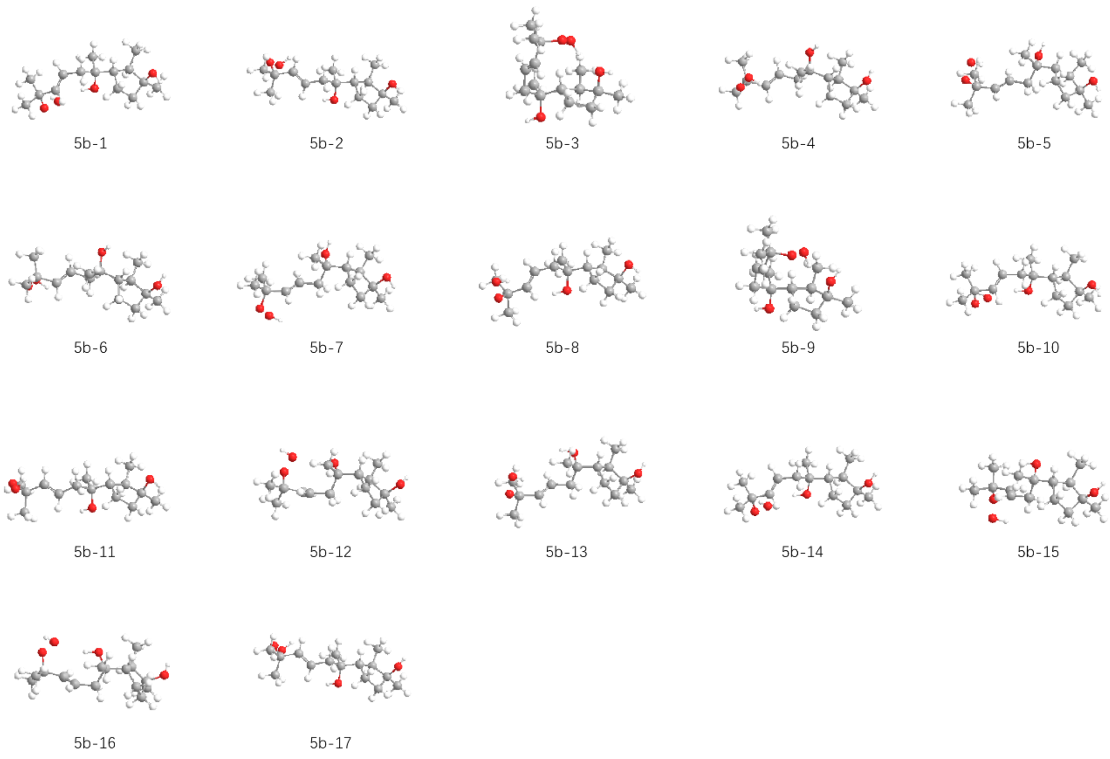

Figure S 96. Most stable conformers of 2*R*, 3*S*, 6*S*, 7*S*-5 at the CAM-B3LYP/DGDZVP level

Table S 38. Optimized Z-Matrixes of 2*R*, 3*S*, 6*S*, 7*S*-5 conformations in the methanol at CAM-B3LYP/DGDZVP level

	5b-1			5b-2			5b-3		
C	-1.77714	0.439339	-0.67769	-1.84712	-0.18919	-0.56599	1.016302	0.574646	-0.13407
C	-2.87345	0.526995	0.422251	-2.96136	0.748297	-0.02265	2.226515	-0.06857	0.582856
C	-3.82503	-0.65613	0.155637	-4.19846	-0.14989	0.17453	2.448608	-1.35217	-0.24324
C	-2.8765	-1.7305	-0.37585	-3.57628	-1.47977	0.600097	2.303557	-0.85076	-1.67725
C	-1.94391	-0.96732	-1.31623	-2.36633	-1.63427	-0.32165	1.115459	0.115378	-1.61626
C	-0.34074	0.703571	-0.17428	-0.44882	0.053938	0.042926	0.755328	2.083188	0.00179
C	0.619028	0.780702	-1.39891	0.595269	-0.78781	-0.73595	-0.63048	2.407183	-0.63512
C	2.05315	0.867557	-0.96517	1.958632	-0.79916	-0.10816	-1.73652	1.575806	-0.04524
C	2.81723	-0.21015	-0.77877	3.057438	-0.29865	-0.6753	-2.25955	0.513911	-0.66057
C	4.176128	-0.25067	-0.12762	4.453411	-0.29776	-0.09683	-3.17785	-0.52894	-0.07157
O	0.009426	-0.4024	0.662035	-0.51895	-0.38608	1.408272	1.773126	2.767714	-0.74058
C	-4.62384	-1.09256	1.37898	-5.2099	0.401932	1.173172	3.758833	-2.07705	0.027088
C	5.194702	-0.94322	-1.03227	4.555682	-0.83004	1.327669	-3.67949	-0.217	1.332801
C	4.693781	1.100224	0.352524	5.406144	-1.04574	-1.03176	-4.34483	-0.80299	-1.02079
H	-1.98186	1.207152	-1.43198	-1.74456	-0.0191	-1.64333	0.137497	0.106631	0.308991
C	-3.58576	1.875981	0.500509	-3.23305	1.978891	-0.88565	2.038046	-0.35106	2.073974
O	-4.72528	-0.24634	-0.88941	-4.82196	-0.29776	-1.11378	1.335895	-2.22989	0.064683
C	-0.23672	1.998192	0.640476	-0.04137	1.528229	0.007965	0.788888	2.57203	1.451117
O	4.083318	-1.1758	0.993706	4.971073	1.05834	-0.16034	-2.47247	-1.80311	-0.0857
O	3.045126	-0.75896	1.883912	4.198496	1.914194	0.682508	-1.41406	-1.79442	0.879202
H	-2.40578	0.31647	1.390707	-2.67188	1.075332	0.982716	3.119369	0.552592	0.440456

H	-2.30743	-2.15052	0.459561	-3.24959	-1.40408	1.642055	3.219854	-0.32408	-1.96208
H	-3.41284	-2.55232	-0.8622	-4.28723	-2.31	0.531744	2.162238	-1.67173	-2.387
H	-0.99464	-1.49053	-1.43802	-1.61261	-2.28492	0.124022	1.260842	0.958526	-2.29262
H	-2.39923	-0.87561	-2.3052	-2.66977	-2.08529	-1.26927	0.194999	-0.38964	-1.91841
H	0.340491	1.652074	-1.99931	0.657503	-0.40398	-1.75907	-0.56287	2.230698	-1.71159
H	0.476022	-0.10801	-2.01734	0.235067	-1.81931	-0.79801	-0.82946	3.475846	-0.49076
H	2.432585	1.8499	-0.69332	2.028912	-1.26501	0.873511	-2.03102	1.806307	0.97716
H	2.425786	-1.19149	-1.0494	2.985504	0.156876	-1.66455	-1.94374	0.290637	-1.68023
H	0.947801	-0.32052	0.901319	0.234326	-0.0144	1.885642	1.636741	3.717948	-0.62574
H	-5.26083	-1.94908	1.134567	-5.60815	1.361683	0.832945	3.795826	-2.45833	1.050224
H	-3.95902	-1.38878	2.194823	-6.05006	-0.29089	1.288162	3.873641	-2.92627	-0.65447
H	-5.26786	-0.28243	1.731779	-4.75241	0.543549	2.155993	4.607818	-1.4059	-0.12631
H	6.145819	-1.07648	-0.51052	5.587884	-0.74077	1.674579	-4.31049	-1.03584	1.687002
H	4.828641	-1.92117	-1.35297	3.910496	-0.2733	2.009239	-2.85438	-0.08904	2.034577
H	5.366637	-0.32878	-1.91797	4.273993	-1.8848	1.362736	-4.27564	0.69858	1.323924
H	5.649557	0.965067	0.864626	6.434879	-0.96902	-0.67056	-4.97081	-1.61282	-0.63799
H	3.993402	1.580268	1.039736	5.357914	-0.64073	-2.04538	-3.98315	-1.07794	-2.01478
H	4.852111	1.772177	-0.49384	5.125315	-2.1	-1.06596	-4.95537	0.097559	-1.11327
H	-4.37099	1.866346	1.262788	-4.05296	2.576958	-0.47624	2.847166	-0.97913	2.457687
H	-2.88947	2.677695	0.755876	-2.35436	2.624575	-0.94529	2.035633	0.563395	2.667009
H	-4.04925	2.123984	-0.45726	-3.50566	1.684911	-1.90192	1.094209	-0.87235	2.255338
H	-5.21632	-1.02995	-1.17057	-5.50336	-0.97779	-1.02727	1.414711	-3.01186	-0.49884
H	-0.54477	2.866623	0.051182	-0.04504	1.913531	-1.01499	0.14054	1.974737	2.097032
H	-0.85827	1.943012	1.536068	-0.71382	2.136557	0.615884	1.807131	2.526506	1.84184
H	0.793513	2.160483	0.971011	0.9716	1.650885	0.404953	0.453567	3.613401	1.50732
H	3.539899	-0.29966	2.582427	3.525385	2.254814	0.06981	-0.60164	-1.84241	0.331361
	5b-4			5b-5			5b-6		
C	-1.92084	0.563067	0.399127	1.966514	-0.8189	-0.42653	2.008333	-0.74948	-0.01875
C	-2.7581	-0.68947	0.082648	2.884964	-0.19449	0.660715	2.720964	0.52026	0.482505
C	-4.00694	-0.09643	-0.60915	3.771495	0.827081	-0.07982	3.984612	0.574927	-0.40608
C	-3.41548	0.986397	-1.51824	2.849452	1.326472	-1.19518	3.445224	0.199125	-1.78911
C	-2.10846	1.459315	-0.84675	2.162291	0.054596	-1.6941	2.235901	-0.72905	-1.54933
C	-0.45298	0.358983	0.834396	0.488978	-0.99294	0.00617	0.541869	-0.97205	0.413102
C	0.297487	-0.61048	-0.09426	-0.21979	0.352722	0.246443	-0.32263	0.281313	0.195245
C	1.765211	-0.70491	0.204309	-1.66742	0.203071	0.615619	-1.78319	0.05777	0.459015
C	2.729456	-0.43199	-0.67586	-2.67265	0.768593	-0.05416	-2.7437	0.221446	-0.45199
C	4.219414	-0.48142	-0.42696	-4.1468	0.643454	0.25367	-4.22371	-0.01382	-0.25689
O	-0.40838	-0.26427	2.129746	0.439849	-1.67032	1.273183	0.48019	-1.21924	1.828774
C	-4.84601	-1.12062	-1.3656	4.307672	1.938411	0.815681	4.702812	1.919846	-0.38748
C	4.620764	-0.94349	0.968504	-4.47732	-0.22397	1.462173	-4.73361	-0.99454	-1.31433
C	4.904664	-1.32481	-1.50258	-4.77588	2.03111	0.385451	-4.62063	-0.46738	1.142822
H	-2.41392	1.06706	1.242057	2.330709	-1.83183	-0.63864	2.568971	-1.60469	0.383623
C	-3.05435	-1.60565	1.265756	3.664818	-1.21692	1.482945	2.988694	0.580162	1.983179

O	-4.83573	0.593714	0.343293	4.867692	0.093957	-0.6535	4.907296	-0.46751	-0.04299
C	0.257055	1.715137	0.921729	-0.26869	-1.82876	-1.03153	-0.03668	-2.18774	-0.31994
O	4.75917	0.844421	-0.68962	-4.8085	0.121436	-0.93243	-4.93094	1.207352	-0.60797
O	4.246657	1.778648	0.259881	-4.35701	-1.20599	-1.20016	-4.60215	2.24505	0.314894
H	-2.24271	-1.27327	-0.69101	2.269771	0.386393	1.355083	2.124831	1.397197	0.199056
H	-3.20913	0.556319	-2.50151	2.116572	2.021375	-0.77051	3.133825	1.108556	-2.30936
H	-4.14031	1.790987	-1.66012	3.397097	1.862832	-1.97693	4.232662	-0.26555	-2.38667
H	-1.27101	1.344939	-1.54075	1.231302	0.26915	-2.22354	1.358436	-0.3407	-2.07355
H	-2.1442	2.515146	-0.57092	2.814008	-0.4631	-2.40103	2.407702	-1.73685	-1.93269
H	0.148504	-0.31062	-1.1353	-0.1331	0.976544	-0.64651	-0.18167	0.650419	-0.82448
H	-0.152	-1.60154	0.029148	0.296473	0.87404	1.059474	0.041622	1.056082	0.878162
H	2.023163	-1.01198	1.215732	-1.87306	-0.41573	1.486272	-2.03771	-0.2743	1.463352
H	2.45552	-0.1163	-1.68408	-2.45131	1.380878	-0.93025	-2.4721	0.545762	-1.45817
H	-0.87416	0.312041	2.750789	0.811614	-2.55426	1.149381	1.021159	-1.99847	2.015906
H	-5.30076	-1.84542	-0.68264	4.935628	1.525614	1.609916	5.125849	2.131552	0.599827
H	-5.65153	-0.61707	-1.90722	4.913824	2.641276	0.234645	5.522572	1.915957	-1.11117
H	-4.23541	-1.67213	-2.08543	3.489331	2.499063	1.27549	4.018286	2.733084	-0.64282
H	5.709043	-0.91433	1.061691	-5.56192	-0.28441	1.580017	-5.81748	-1.11209	-1.23721
H	4.190658	-0.30448	1.741075	-4.08474	-1.2355	1.348098	-4.48802	-0.64753	-2.3209
H	4.288585	-1.97003	1.138761	-4.05745	0.213623	2.370656	-4.26444	-1.96824	-1.16006
H	5.990523	-1.28974	-1.38342	-5.85835	1.950726	0.513448	-5.70666	-0.5756	1.194937
H	4.648742	-0.9667	-2.5027	-4.57035	2.63941	-0.49866	-4.30773	0.251709	1.901235
H	4.576863	-2.36222	-1.41203	-4.35761	2.536257	1.258173	-4.16828	-1.43517	1.371014
H	-3.76004	-2.39646	0.991486	4.332658	-0.7261	2.197654	3.610214	1.442925	2.243701
H	-2.14058	-2.08039	1.62568	2.977493	-1.84911	2.050061	2.053544	0.658037	2.539341
H	-3.47426	-1.04397	2.107005	4.272417	-1.85703	0.838905	3.493065	-0.32707	2.333057
H	-5.19565	-0.06274	0.9551	5.347457	0.69881	-1.23518	5.232156	-0.2794	0.847942
H	0.423254	2.151524	-0.06659	-0.37703	-1.29748	-1.9806	-0.18429	-1.98875	-1.38427
H	-0.34104	2.420747	1.507873	0.261778	-2.76605	-1.22813	0.638078	-3.04502	-0.22462
H	1.226353	1.597525	1.412497	-1.26798	-2.07164	-0.66228	-1.00305	-2.45983	0.110789
H	3.386255	2.021306	-0.12396	-3.5473	-1.05368	-1.71711	-3.7723	2.598041	-0.04967
	5b-7			5b-8			5b-9		
C	2.067585	-0.90595	-0.37197	-1.86455	0.168311	-0.67366	1.023719	0.566603	-0.11764
C	2.85562	-0.0976	0.696633	-3.08187	0.63032	0.177561	2.286242	-0.04399	0.532906
C	3.697752	0.928167	-0.08953	-4.03339	-0.58133	0.232932	2.449408	-1.36881	-0.25269
C	2.821586	1.219495	-1.31088	-3.06444	-1.76345	0.241527	2.187009	-0.93782	-1.68795
C	2.292548	-0.15784	-1.71238	-1.99886	-1.37582	-0.78365	1.02046	0.051445	-1.58408
C	0.57849	-1.1485	-0.01687	-0.49209	0.616197	-0.12423	0.772341	2.080153	-0.02312
C	-0.2443	0.153531	0.000208	0.611572	0.253357	-1.15514	-0.63328	2.393386	-0.61852
C	-1.68218	-0.05118	0.381567	2.000254	0.556647	-0.66687	-1.71723	1.552845	-0.00264
C	-2.71521	0.238441	-0.41108	2.793259	-0.34932	-0.08816	-2.30185	0.533455	-0.634
C	-4.1795	0.035634	-0.09734	4.169864	-0.11258	0.488844	-3.21606	-0.50883	-0.03753
O	0.486446	-1.66697	1.320634	-0.28214	-0.11203	1.09069	1.765677	2.732887	-0.82496

C	4.059528	2.170294	0.717099	-4.98814	-0.56164	1.421725	3.784287	-2.07341	-0.06067
C	-4.82492	-0.8262	-1.18403	4.689036	1.312592	0.341776	-3.64596	-0.23472	1.398279
C	-4.46473	-0.53104	1.288262	4.196308	-0.55656	1.95269	-4.43074	-0.72668	-0.94005
H	2.517639	-1.90373	-0.44659	-1.95225	0.612307	-1.67128	0.179125	0.112779	0.404058
C	3.664677	-0.96112	1.660889	-3.75337	1.909073	-0.31956	2.207471	-0.26163	2.044918
O	4.898114	0.255947	-0.50841	-4.78653	-0.58243	-0.99291	1.369499	-2.27565	0.078944
C	-0.03495	-2.16438	-0.98608	-0.44592	2.11814	0.177059	0.863865	2.614341	1.408026
O	-4.86443	1.306544	-0.26991	5.090381	-1.04936	-0.13129	-2.54107	-1.79672	-0.12461
O	-4.40617	2.244034	0.703801	5.220226	-0.756	-1.52219	-1.43298	-1.83464	0.780952
H	2.147298	0.485265	1.293821	-2.74206	0.786078	1.207784	3.162911	0.573616	0.301922
H	1.998816	1.879195	-1.01438	-2.61584	-1.85094	1.23616	3.082922	-0.441	-2.07127
H	3.376885	1.72396	-2.10834	-3.56544	-2.71079	0.01579	1.977727	-1.79659	-2.33046
H	1.395617	-0.09233	-2.33231	-1.06027	-1.8967	-0.59022	1.137304	0.868041	-2.29781
H	3.046379	-0.6824	-2.30334	-2.32609	-1.64964	-1.7894	0.072904	-0.44277	-1.81208
H	-0.19013	0.628311	-0.98253	0.414513	0.810862	-2.07621	-0.59638	2.220889	-1.69701
H	0.209594	0.842345	0.720585	0.536581	-0.81036	-1.39099	-0.83719	3.460102	-0.46575
H	-1.85551	-0.47624	1.367394	2.343628	1.584607	-0.7596	-1.94885	1.742925	1.044075
H	-2.5255	0.654901	-1.40201	2.445614	-1.38069	-0.00478	-2.05141	0.349976	-1.6794
H	0.940756	-2.52024	1.337372	0.623768	0.063618	1.385168	1.63301	3.68714	-0.74394
H	4.654749	1.90114	1.593988	-5.64134	0.314274	1.381237	3.914195	-2.42283	0.968357
H	4.647297	2.865709	0.108877	-5.62052	-1.45565	1.418976	3.847613	-2.94165	-0.72191
H	3.160589	2.693367	1.054318	-4.43702	-0.54123	2.365731	4.611493	-1.39812	-0.29331
H	-5.90132	-0.91315	-1.01563	5.6997	1.373802	0.752191	-4.28272	-1.04934	1.751905
H	-4.65721	-0.39726	-2.17489	4.7206	1.621986	-0.70393	-2.78703	-0.1543	2.065751
H	-4.3856	-1.82543	-1.16068	4.052908	2.008176	0.893853	-4.21631	0.695746	1.448871
H	-5.545	-0.60577	1.43443	5.208519	-0.47772	2.357042	-5.05614	-1.53623	-0.55577
H	-4.05205	0.104167	2.073371	3.855585	-1.58957	2.055205	-4.121	-0.97473	-1.95833
H	-4.03555	-1.5308	1.385541	3.535899	0.086107	2.538131	-5.02519	0.188633	-0.97388
H	4.245988	-0.34559	2.354346	-4.62621	2.161311	0.290459	3.027554	-0.89554	2.396589
H	2.998216	-1.59084	2.255318	-3.06815	2.758526	-0.28035	2.273724	0.673221	2.600642
H	4.359783	-1.60789	1.120355	-4.08718	1.792505	-1.35319	1.26252	-0.73839	2.326166
H	5.364434	0.84831	-1.11338	-5.2687	-1.41909	-1.03389	1.532184	-2.62664	0.965371
H	-0.06683	-1.77969	-2.00859	-0.65121	2.709581	-0.71966	0.247445	2.03447	2.099662
H	0.553783	-3.08741	-0.9923	-1.17376	2.382072	0.946334	1.89793	2.58602	1.755921
H	-1.05612	-2.40843	-0.68443	0.540866	2.404368	0.552774	0.526057	3.655514	1.446608
H	-3.61063	2.613514	0.283714	4.496387	-1.27297	-1.91489	-0.65154	-1.85603	0.191021
	5b-10			5b-11			5b-12		
C	-1.77723	0.438796	-0.67786	-1.87078	-0.15896	-0.5544	-1.90454	0.982561	0.202458
C	-2.8735	0.52717	0.422032	-3.05965	0.721881	-0.07782	-2.40215	-0.41201	0.67255
C	-3.82538	-0.65585	0.155933	-4.22335	-0.25614	0.17761	-3.6063	-0.762	-0.23761
C	-2.87708	-1.73068	-0.37505	-3.50111	-1.50144	0.691517	-3.26175	-0.05541	-1.54572
C	-1.94425	-0.96816	-1.31572	-2.27818	-1.6212	-0.21806	-2.69106	1.29115	-1.09709
C	-0.34078	0.703146	-0.1746	-0.50214	0.231385	0.043716	-0.37051	1.088575	0.055676

C	0.619025	0.779447	-1.39927	0.613577	-0.57629	-0.67267	0.17095	0.201599	-1.10135
C	2.053148	0.86674	-0.9656	1.945491	-0.50906	0.016814	1.671355	0.220853	-1.13627
C	2.817438	-0.21074	-0.77869	3.002541	0.162136	-0.44413	2.423162	-0.68022	-0.5021
C	4.176386	-0.25065	-0.12762	4.352967	0.292876	0.221415	3.913651	-0.61803	-0.28546
O	0.009239	-0.40232	0.662469	-0.55354	-0.11859	1.435379	0.163023	0.630576	1.302482
C	-4.6243	-1.09155	1.379463	-5.28118	0.278951	1.136544	-3.83522	-2.26159	-0.39152
C	5.195045	-0.94371	-1.03177	4.693479	1.773041	0.405035	4.588401	-1.9035	-0.76261
C	4.693877	1.100586	0.351689	4.495977	-0.46651	1.53489	4.593016	0.612735	-0.87457
H	-1.98188	1.206264	-1.43252	-1.77026	-0.05145	-1.64006	-2.17325	1.718708	0.967769
C	-3.58545	1.87638	0.49974	-3.42178	1.867344	-1.02107	-2.70152	-0.48253	2.167622
O	-4.72554	-0.24634	-0.88932	-4.8249	-0.53804	-1.09865	-4.8129	-0.13563	0.231713
C	-0.23663	1.998258	0.639342	-0.20791	1.727123	-0.09004	0.039637	2.553302	-0.14709
O	4.083741	-1.17503	0.994356	5.370339	-0.13955	-0.72185	4.137348	-0.66989	1.15281
O	3.045677	-0.7576	1.884361	5.227466	-1.53459	-0.9879	3.433196	0.396816	1.79328
H	-2.40586	0.316949	1.390568	-2.80419	1.13762	0.903563	-1.62829	-1.15703	0.462909
H	-2.30816	-2.15048	0.46057	-3.18648	-1.33067	1.725948	-2.50174	-0.63937	-2.07516
H	-3.4136	-2.55259	-0.86106	-4.14437	-2.38761	0.678927	-4.13425	0.033114	-2.19855
H	-0.99509	-1.49166	-1.43718	-1.47871	-2.18205	0.268295	-2.08544	1.763569	-1.87388
H	-2.39947	-0.87685	-2.30477	-2.54171	-2.15388	-1.13474	-3.50983	1.979097	-0.87512
H	0.340436	1.650368	-2.0003	0.701672	-0.20501	-1.69846	-0.2354	0.559418	-2.05128
H	0.476087	-0.1097	-2.01709	0.308357	-1.62508	-0.73182	-0.17741	-0.82371	-0.95416
H	2.432462	1.8493	-0.69436	2.021935	-1.04456	0.961564	2.142818	1.075163	-1.6167
H	2.426167	-1.19228	-1.04884	2.920672	0.693732	-1.39389	1.939462	-1.52935	-0.01793
H	0.947532	-0.32023	0.902026	0.192235	0.306529	1.879459	1.132267	0.611661	1.22997
H	-5.26815	-0.28113	1.731875	-5.75186	1.179622	0.733179	-4.06155	-2.72726	0.573787
H	-5.26146	-1.94805	1.135445	-6.06467	-0.46905	1.296936	-4.67715	-2.44718	-1.06404
H	-3.95955	-1.38753	2.19546	-4.84197	0.521018	2.108038	-2.94815	-2.75212	-0.80098
H	6.146187	-1.07649	-0.50994	5.702898	1.886243	0.808361	5.64884	-1.9034	-0.49796
H	4.829122	-1.92193	-1.35183	4.631587	2.309806	-0.54462	4.113994	-2.7805	-0.31647
H	5.366898	-0.32984	-1.91787	3.985381	2.222865	1.103712	4.500459	-1.97527	-1.84825
H	5.649753	0.965879	0.863735	5.512248	-0.34262	1.916425	5.654233	0.604833	-0.61418
H	3.993516	1.580908	1.038727	4.302344	-1.53228	1.405029	4.148272	1.537776	-0.50087
H	4.851971	1.772084	-0.49507	3.80045	-0.0726	2.279469	4.508851	0.612152	-1.96355
H	-4.37056	1.867331	1.262148	-4.29138	2.421888	-0.65514	-3.09123	-1.46488	2.456831
H	-2.8889	2.678033	0.754606	-2.5979	2.577448	-1.11838	-1.78786	-0.30361	2.737421
H	-4.04901	2.124051	-0.45808	-3.65976	1.48763	-2.01743	-3.43062	0.278491	2.464285
H	-5.21677	-1.02996	-1.17013	-5.45164	-1.2622	-0.968	-5.04621	-0.536	1.079887
H	-0.54486	2.866324	0.049591	-0.25211	2.04428	-1.13522	-0.31225	2.948869	-1.1034
H	-0.85796	1.943616	1.535113	-0.9211	2.319182	0.487049	-0.3752	3.167937	0.656076
H	0.793676	2.160792	0.969534	0.796755	1.952571	0.28227	1.128223	2.659556	-0.1208
H	3.540544	-0.29796	2.582578	4.578905	-1.5461	-1.71242	4.13035	1.061312	1.920519
	5b-13			5b-14			5b-15		
C	1.979336	-0.86257	-0.39337	-1.7755	0.439296	-0.67393	2.082887	-1.0125	-0.13631

C	2.861089	-0.16736	0.681409	-2.87174	0.51496	0.425748	2.549577	0.15861	0.771555
C	3.783245	0.806065	-0.09497	-3.8282	-0.66915	0.142418	3.591356	0.948079	-0.06039
C	2.923583	1.208083	-1.28999	-2.88566	-1.73262	-0.40659	3.106253	0.745292	-1.49301
C	2.251994	-0.09889	-1.71501	-1.94152	-0.96014	-1.32857	2.689038	-0.72591	-1.53412
C	0.484954	-0.96763	-0.01654	-0.33957	0.700466	-0.16805	0.555251	-1.24041	-0.14358
C	-0.2049	0.417481	0.035349	0.620173	0.789002	-1.39183	-0.21295	-0.06103	-0.79433
C	-1.62815	0.366491	0.51247	2.05401	0.875103	-0.95708	-1.70415	-0.24281	-0.74751
C	-2.6838	0.694407	-0.23393	2.820422	-0.20243	-0.77961	-2.48988	0.318784	0.174982
C	-4.13369	0.675375	0.190832	4.179337	-0.24516	-0.12863	-3.98173	0.132987	0.328933
O	0.466738	-1.56257	1.292034	0.011149	-0.41233	0.65875	0.180427	-1.34282	1.235763
C	4.263145	1.986416	0.742366	-4.621	-1.1221	1.363544	3.710612	2.412484	0.347279
C	-4.38442	0.10951	1.583551	5.199758	-0.9271	-1.03923	-4.62615	-0.76374	-0.72121
C	-4.73667	2.074662	0.050139	4.693498	1.102844	0.363281	-4.29316	-0.36594	1.741335
H	2.322701	-1.89697	-0.49968	-1.98051	1.214597	-1.42052	2.510743	-1.94133	0.256095
C	3.601093	-1.14321	1.592681	-3.58316	1.864141	0.513751	3.048012	-0.29017	2.142853
O	4.91566	0.116084	-0.65117	-4.7325	-0.3583	-0.93237	4.884971	0.322811	-0.00163
C	-0.23999	-1.89618	-0.9946	-0.23783	1.988303	0.657719	0.22356	-2.56343	-0.84549
O	-4.88695	-0.07847	-0.79656	4.088694	-1.18018	0.984573	-4.60686	1.44378	0.322891
O	-4.46165	-1.44202	-0.79689	3.049229	-0.77369	1.878039	-4.44335	2.055197	-0.95659
H	2.22601	0.454656	1.320154	-2.40651	0.299333	1.394296	1.708653	0.838713	0.939867
H	2.17595	1.937009	-0.96028	-2.32418	-2.16868	0.425051	2.244486	1.396813	-1.67165
H	3.521481	1.671201	-2.07945	-3.43275	-2.53469	-0.90953	3.880055	1.002097	-2.22129
H	1.355946	0.073031	-2.31573	-0.99224	-1.48455	-1.44582	2.007062	-0.93984	-2.36029
H	2.939149	-0.67798	-2.33555	-2.38544	-0.85777	-2.32155	3.571256	-1.35144	-1.68571
H	-0.17158	0.877783	-0.95583	0.340215	1.664568	-1.98543	0.102941	0.042043	-1.8363
H	0.362896	1.062598	0.713622	0.47875	-0.09516	-2.0171	0.05378	0.862256	-0.27393
H	-1.78118	0.049412	1.542962	2.431326	1.855922	-0.67694	-2.1409	-0.90631	-1.49079
H	-2.52639	1.02543	-1.26196	2.431315	-1.18236	-1.05856	-2.04468	0.988013	0.913433
H	-0.42872	-1.88185	1.466366	0.949674	-0.33245	0.898146	-0.78667	-1.39237	1.27274
H	4.862856	1.64778	1.594126	-5.28173	-0.32719	1.725998	4.044637	2.507188	1.386205
H	4.881939	2.652471	0.134956	-5.24022	-1.98657	1.109011	4.436791	2.923341	-0.29083
H	3.417152	2.557939	1.133308	-3.95242	-1.40241	2.181605	2.748067	2.922879	0.256397
H	-5.45933	0.088706	1.778156	6.151209	-1.06239	-0.51862	-5.70414	-0.80628	-0.54886
H	-3.99413	-0.9051	1.678046	4.836288	-1.90317	-1.3685	-4.4512	-0.38831	-1.73051
H	-3.91272	0.737998	2.342249	5.370084	-0.30447	-1.9195	-4.22817	-1.77863	-0.65105
H	-5.80853	2.052177	0.262796	5.649459	0.965649	0.8745	-5.37341	-0.42155	1.89688
H	-4.58614	2.466009	-0.95881	3.991738	1.575257	1.05437	-3.86262	0.29765	2.495003
H	-4.25366	2.749336	0.759337	4.85045	1.782392	-0.47725	-3.86941	-1.36322	1.874873
H	4.2481	-0.61975	2.305326	-4.39236	1.845074	1.251729	3.411739	0.554858	2.737935
H	2.883755	-1.73549	2.163578	-2.89702	2.660049	0.809174	2.235377	-0.76425	2.696332
H	4.217399	-1.8387	1.013797	-4.00757	2.148393	-0.45442	3.857473	-1.02187	2.05279
H	5.473118	-0.18077	0.080352	-5.34654	0.319099	-0.61935	5.213509	0.401058	0.903817
H	-0.25939	-1.48216	-2.00563	-0.55058	2.86078	0.076842	0.456167	-2.52893	-1.91287

H	0.257744	-2.86882	-1.02607	-0.85647	1.922574	1.554638	0.796021	-3.37672	-0.39215
H	-1.27822	-2.05134	-0.6845	0.792548	2.151583	0.987147	-0.83859	-2.80416	-0.74257
H	-3.7776	-1.44468	-1.48725	3.542729	-0.31982	2.580985	-3.59165	2.514768	-0.86298
	5b-16			5b-17					
C	-1.90462	0.982619	0.202349	-1.84661	-0.18951	-0.56064			
C	-2.40219	-0.4119	0.672647	-2.96306	0.740262	-0.01103			
C	-3.60625	-0.76209	-0.23754	-4.20416	-0.16685	0.1727			
C	-3.26167	-0.05569	-1.54575	-3.58366	-1.49713	0.580667			
C	-2.69112	1.290977	-1.09726	-2.36079	-1.63834	-0.32698			
C	-0.37058	1.088639	0.055569	-0.44792	0.054145	0.046657			
C	0.170921	0.201463	-1.10132	0.596189	-0.78099	-0.73927			
C	1.67133	0.220736	-1.1363	1.960307	-0.7963	-0.11318			
C	2.423182	-0.68022	-0.502	3.057849	-0.28956	-0.67728			
C	3.913668	-0.61799	-0.28549	4.455021	-0.29448	-0.10171			
O	0.162881	0.630897	1.302503	-0.51452	-0.39278	1.409718			
C	-3.83505	-2.26172	-0.39121	-5.21554	0.372136	1.178453			
C	4.588462	-1.90336	-0.76285	4.560282	-0.84175	1.316883			
C	4.592948	0.612899	-0.87439	5.405477	-1.03302	-1.04647			
H	-2.17334	1.718887	0.967536	-1.74593	-0.01247	-1.63712			
C	-2.70166	-0.48215	2.16771	-3.234	1.976879	-0.86648			
O	-4.81294	-0.13573	0.231578	-4.85622	-0.41164	-1.086			
C	0.039501	2.553345	-0.1474	-0.04468	1.529721	0.018174			
O	4.137475	-0.67005	1.152807	4.973016	1.06199	-0.15233			
O	3.433484	0.396589	1.793532	4.202725	1.909369	0.701151			
H	-1.62828	-1.15691	0.463205	-2.6768	1.06404	0.996377			
H	-2.50159	-0.63965	-2.07508	-3.27032	-1.43255	1.626912			
H	-4.13415	0.032686	-2.19861	-4.29727	-2.32012	0.486734			
H	-2.08552	1.763353	-1.87409	-1.60797	-2.28461	0.126725			
H	-3.50996	1.978867	-0.8754	-2.64844	-2.09001	-1.27908			
H	-0.23545	0.559108	-2.0513	0.657048	-0.38948	-1.75955			
H	-0.17742	-0.82383	-0.95396	0.236744	-1.81229	-0.8086			
H	2.142756	1.074924	-1.61697	2.032473	-1.27172	0.863743			
H	1.939517	-1.52929	-0.01771	2.983951	0.175328	-1.66201			
H	1.132136	0.612073	1.230061	0.241623	-0.02594	1.886345			
H	-4.06136	-2.72723	0.574176	-5.63384	1.329381	0.84928			
H	-4.67696	-2.44749	-1.06372	-6.04122	-0.33513	1.295129			
H	-2.94793	-2.75226	-0.80057	-4.75023	0.527125	2.155329			
H	5.648928	-1.90321	-0.49831	5.593186	-0.75592	1.662561			
H	4.114159	-2.78044	-0.31674	3.916404	-0.29251	2.00573			
H	4.500412	-1.97503	-1.84849	4.278911	-1.89688	1.3413			
H	5.65415	0.605064	-0.61394	6.434982	-0.96068	-0.68657			
H	4.148073	1.537836	-0.5006	5.355399	-0.61723	-2.05563			
H	4.508858	0.61246	-1.96338	5.124002	-2.08671	-1.0913			

H	-3.0914	-1.46445	2.457061	-4.07873	2.556527	-0.47908			
H	-1.78801	-0.30316	2.737512	-2.37113	2.644801	-0.8912			
H	-3.43075	0.278938	2.464206	-3.45456	1.69824	-1.90186			
H	-5.04625	-0.53594	1.079823	-5.2565	0.416643	-1.38197			
H	-0.3123	2.948712	-1.10382	-0.05656	1.92155	-1.00229			
H	-0.37548	3.16809	0.655603	-0.71451	2.131933	0.635102			
H	1.128075	2.659685	-0.12099	0.970587	1.65273	0.409023			
H	4.13059	1.0612	1.920501	3.530456	2.25913	0.092723			

Table S 39. Energy analysis for 2*R*, 3*S*, 6*S*, 7*S*-5

Conf.	Steric Energy (kJ/mol)	Relative Energy (kJ/mol)	Distribution (%)
5b-1	-888.724866	0.001586	3.09%
5b-2	-888.724684	0.001768	2.55%
5b-3	-888.725045	0.001407	3.74%
5b-4	-888.725905	0.000547	9.30%
5b-5	-888.726063	0.000389	10.99%
5b-6	-888.726452	0	16.59%
5b-7	-888.725442	0.00101	5.69%
5b-8	-888.724854	0.001598	3.06%
5b-9	-888.725004	0.001448	3.58%
5b-10	-888.724868	0.001584	3.10%
5b-11	-888.724714	0.001738	2.63%
5b-12	-888.725946	0.000506	9.71%
5b-13	-888.724714	0.001738	2.63%
5b-14	-888.725307	0.001145	4.94%
5b-15	-888.725205	0.001247	4.43%
5b-16	-888.725952	0.0005	9.77%
5b-17	-888.725155	0.001297	4.20%

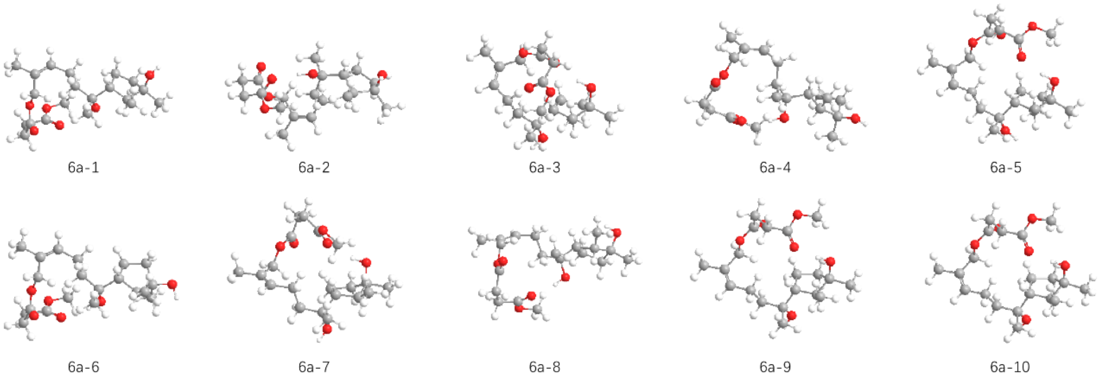

Figure S 97. Most stable conformers of 2*S*, 3*R*, 6*R*, 7*R*-6 calculated at MPW1PW91/6-31G+d, p level

Table S 40. Optimized Z-Matrixes of 2*S*, 3*R*, 6*R*, 7*R*-6 conformations in the Chloroform at MPW1PW91/6-31G+d, p level

	6a-1			6a-2			66a-3		
C	-2.47532	-0.59877	-0.09777	-2.56476	0.256819	0.933045	1.143065	-1.17999	0.184328

C	-3.58681	-0.12854	0.883248	-3.25782	0.445958	-0.4325	2.553229	-0.53057	0.16179
C	-4.79441	0.235772	-0.0027	-4.64579	-0.19834	-0.22033	2.991351	-0.62534	-1.32068
C	-4.12667	0.77862	-1.26651	-4.30361	-1.46278	0.565664	2.477558	-2.00962	-1.71764
C	-2.96666	-0.18639	-1.5113	-3.12653	-1.08295	1.482578	1.090667	-2.09925	-1.07279
O	-5.47894	-0.99185	-0.30776	-5.45499	0.602265	0.655356	2.367758	0.390961	-2.11735
C	-5.76323	1.215982	0.650001	-5.40831	-0.47741	-1.51122	4.486286	-0.45371	-1.53701
C	-1.07154	-0.05362	0.23335	-1.03042	0.409669	0.95781	0.747932	-1.92453	1.476503
C	-0.00141	-0.59673	-0.73552	-0.2877	-0.28561	-0.19854	-0.72955	-2.37121	1.406384
C	0.138374	-2.12567	-0.82573	-0.42253	-1.81012	-0.34072	-1.75584	-1.22964	1.533582
C	1.419026	-2.51425	-1.52577	0.552711	-2.3382	-1.36564	-3.13239	-1.61442	1.054398
C	2.650116	-2.46001	-0.99919	1.867814	-2.5181	-1.18216	-3.58423	-1.46324	-0.19787
C	3.890356	-2.79036	-1.78687	2.789372	-2.97496	-2.28201	-4.9849	-1.82257	-0.61626
O	-1.1337	1.369429	0.06686	-0.79522	1.820863	0.817937	1.580218	-3.09485	1.528222
C	-0.65748	-0.3583	1.679109	-0.45917	-0.02295	2.314165	0.983629	-1.09433	2.740725
C	2.880871	-2.04084	0.426697	2.525378	-2.25378	0.144473	-2.6853	-0.90842	-1.2747
O	3.423955	-0.69492	0.407422	3.275612	-1.01778	0.023725	-2.73886	0.543364	-1.24068
C	3.591175	-0.10912	1.599912	3.847183	-0.56315	1.146216	-1.63084	1.202612	-1.57306
O	3.327925	-0.64882	2.65579	3.778165	-1.13687	2.214579	-0.60497	0.664021	-1.95351
H	-2.41016	-1.69022	-0.04109	-2.92959	1.057156	1.586006	0.415958	-0.37152	0.060429
C	-3.92176	-1.12854	1.987957	-3.28687	1.879126	-0.95533	2.626605	0.894023	0.70362
C	4.182065	1.279316	1.494681	4.621249	0.719093	0.935104	-1.79399	2.69934	-1.46405
C	4.039258	1.96374	0.143373	4.290397	1.498446	-0.32876	-0.5626	3.370314	-0.86709
C	2.604255	2.243875	-0.22835	2.876487	2.024114	-0.34469	-0.37	3.014411	0.587842
O	2.491469	2.642361	-1.49317	2.543657	2.506923	-1.53975	0.498598	3.834112	1.187699
O	1.659143	2.139936	0.53626	2.130285	2.029565	0.62038	-0.9242	2.091732	1.153601
C	1.170322	2.986324	-1.94784	1.230718	3.082473	-1.66214	0.748508	3.577789	2.578553
H	1.328852	-2.8179	-2.56766	0.149184	-2.54008	-2.35682	-3.80694	-2.04474	1.793663
H	-3.25949	0.811365	1.341088	-2.75658	-0.18043	-1.18154	3.237378	-1.16994	0.733022
H	-3.74199	1.783256	-1.06541	-4.00461	-2.24409	-0.14025	3.138942	-2.77193	-1.29321
H	-4.82554	0.847437	-2.10752	-5.17646	-1.82962	1.110849	2.46399	-2.13531	-2.80345
H	-3.3187	-1.06712	-2.05428	-3.4533	-0.95623	2.516683	0.327009	-1.74338	-1.76921
H	-2.18463	0.279759	-2.11199	-2.38183	-1.87986	1.490143	0.844567	-3.13044	-0.81821
H	-6.14295	-0.79239	-0.98071	-5.61632	1.449748	0.220098	1.40336	0.361275	-2.00429
H	-6.58311	1.46005	-0.03427	-6.33413	-1.01527	-1.2899	4.731922	-0.60525	-2.59178
H	-6.1979	0.785314	1.556096	-5.674	0.452521	-2.02466	4.809764	0.552446	-1.25736
H	-5.2572	2.148266	0.914739	-4.81046	-1.08231	-2.1987	5.047663	-1.17906	-0.94275
H	0.95953	-0.1773	-0.42128	0.775413	-0.05241	-0.077	-0.90684	-3.10124	2.206807
H	-0.18891	-0.19953	-1.73796	-0.59081	0.187508	-1.13922	-0.88992	-2.90794	0.467072
H	-0.71018	-2.54352	-1.37366	-1.43863	-2.07434	-0.64632	-1.41143	-0.34667	0.992327
H	0.101181	-2.56868	0.173585	-0.25909	-2.29551	0.625408	-1.82492	-0.91809	2.579743
H	3.652168	-3.06379	-2.81658	2.252083	-3.12711	-3.22006	-5.57294	-2.19715	0.223884
H	4.572369	-1.93409	-1.80951	3.578392	-2.23604	-2.45559	-5.49809	-0.94798	-1.03035
H	4.434748	-3.62187	-1.3268	3.283424	-3.91421	-2.01158	-4.97709	-2.58863	-1.39891

H	-0.2578	1.727997	0.290973	0.167197	1.962593	0.834063	1.381733	-3.56279	2.349983
H	-0.71124	-1.4258	1.909408	-0.56856	-1.09314	2.503181	0.492109	-0.11988	2.68154
H	0.370529	-0.02345	1.854858	0.60847	0.217064	2.360919	0.5839	-1.61947	3.616226
H	-1.30276	0.173442	2.38146	-0.9664	0.51776	3.117981	2.052878	-0.94049	2.901034
H	1.969361	-2.03496	1.023526	1.81306	-2.14504	0.961678	-1.65097	-1.21939	-1.14278
H	3.608837	-2.68998	0.921321	3.22783	-3.0496	0.407395	-3.01154	-1.22017	-2.26976
H	-4.72849	-0.75751	2.627752	-3.87035	1.956939	-1.87888	3.630372	1.311241	0.579833
H	-3.05581	-1.31616	2.62673	-2.27517	2.233148	-1.15857	2.384975	0.93736	1.766122
H	-4.24329	-2.08196	1.562639	-3.7127	2.563653	-0.21402	1.932113	1.547423	0.169289
H	3.733152	1.87414	2.290291	4.469699	1.328149	1.826573	-1.94224	3.072442	-2.48128
H	5.244137	1.18006	1.736321	5.678732	0.440828	0.92082	-2.69133	2.929827	-0.89083
H	4.558742	2.926171	0.16341	4.954643	2.363804	-0.40954	0.340901	3.068903	-1.40563
H	4.496311	1.381942	-0.65819	4.447639	0.902132	-1.22852	-0.63403	4.455489	-0.957
H	0.748962	3.77529	-1.32402	1.101096	3.88825	-0.93895	1.110909	2.559438	2.719108
H	1.299143	3.335955	-2.96884	1.178798	3.468722	-2.67678	1.508198	4.296373	2.875483
H	0.520054	2.111748	-1.92234	0.466477	2.321546	-1.50277	-0.16395	3.724529	3.15789
	6a-4			6a-5			6a-6		
C	-2.47737	-0.72084	0.027326	-1.59801	-1.02338	-0.1314	-2.47612	-0.72328	0.028813
C	-3.62972	-0.19067	0.931323	-3.05405	-0.54602	-0.40499	-3.62971	-0.18622	0.926043
C	-4.72758	0.312134	-0.04718	-3.32467	0.577165	0.630275	-4.73212	0.309768	-0.06372
C	-4.45302	-0.46965	-1.33867	-2.5171	0.109478	1.843629	-4.44742	-0.4698	-1.34751
C	-2.93042	-0.49623	-1.42992	-1.21311	-0.43013	1.251177	-2.92495	-0.49716	-1.42919
O	-6.02315	0.022342	0.488152	-2.84894	1.844716	0.169303	-6.06563	-0.00555	0.344649
C	-4.59968	1.821203	-0.26541	-4.80152	0.782025	0.931441	-4.61197	1.81912	-0.27976
C	-1.08845	-0.1455	0.345557	-1.38477	-2.5556	-0.2095	-1.08697	-0.15042	0.350951
C	-0.02592	-0.66026	-0.6438	0.085741	-2.93777	0.028844	-0.02277	-0.66459	-0.63669
C	0.172771	-2.18335	-0.71769	1.11422	-2.25601	-0.88225	0.176414	-2.1876	-0.70978
C	1.429034	-2.52736	-1.48164	2.509985	-2.74015	-0.5884	1.432445	-2.53173	-1.47412
C	2.682527	-2.42787	-1.01832	3.386666	-2.14991	0.233522	2.686202	-2.42961	-1.01208
C	3.89186	-2.71245	-1.86931	4.778776	-2.6762	0.461415	3.8952	-2.71501	-1.86328
O	-1.18105	1.275824	0.187705	-2.07803	-3.21683	0.862327	-1.17859	1.271374	0.195399
C	-0.67119	-0.45555	1.788522	-1.86783	-3.13347	-1.54328	-0.67322	-0.46251	1.794518
C	2.971017	-2.00097	0.394755	3.027882	-0.88811	0.974194	2.975293	-1.99865	0.399647
O	3.462183	-0.63594	0.351687	3.364799	0.237135	0.122366	3.46576	-0.63349	0.352231
C	3.666699	-0.04583	1.53641	2.968298	1.440497	0.549469	3.668459	-0.03842	1.534724
O	3.478823	-0.5973	2.602235	2.418577	1.634145	1.616385	3.479185	-0.58526	2.602713
H	-2.40928	-1.80288	0.191553	-0.96298	-0.57493	-0.90094	-2.41229	-1.80547	0.194537
C	-4.12253	-1.26485	1.90114	-3.31718	-0.11408	-1.84643	-4.12777	-1.25378	1.899683
C	4.196647	1.36493	1.407168	3.224158	2.510519	-0.48635	4.198525	1.371793	1.3999
C	3.970189	2.042848	0.064185	2.105914	2.513828	-1.54478	3.967701	2.045844	0.055685
C	2.511255	2.2688	-0.24589	0.745635	2.744454	-0.93146	2.507678	2.269934	-0.25068
O	2.331468	2.671613	-1.50163	0.647975	3.943669	-0.36552	2.32404	2.671634	-1.50607
O	1.603105	2.122253	0.555605	-0.15428	1.920924	-0.93913	1.601889	2.122543	0.553466

C	0.980753	2.966834	-1.9001	-0.57976	4.232519	0.326815	0.971863	2.96406	-1.9019
H	1.296902	-2.83339	-2.51835	2.811128	-3.66398	-1.08122	1.299761	-2.84055	-2.50991
H	-3.27686	0.662521	1.516748	-3.75777	-1.35317	-0.158	-3.27471	0.670674	1.506
H	-4.84457	-1.48557	-1.21473	-3.05698	-0.70029	2.34692	-4.84169	-1.48499	-1.22638
H	-4.93788	-0.0249	-2.21389	-2.37195	0.920004	2.562702	-4.92961	-0.02395	-2.22185
H	-2.5633	-1.27522	-2.10373	-0.48353	0.375484	1.123724	-2.55568	-1.2752	-2.1028
H	-2.55413	0.461272	-1.80092	-0.76169	-1.16896	1.914519	-2.54659	0.46064	-1.79761
H	-6.67447	0.38118	-0.12861	-1.9303	1.763111	-0.14036	-6.28566	0.550187	1.10363
H	-5.30436	2.166083	-1.03122	-4.92421	1.566903	1.682771	-5.31413	2.153332	-1.04909
H	-4.81628	2.346861	0.668831	-5.34358	1.087114	0.032067	-4.8348	2.353531	0.650993
H	-3.58824	2.091886	-0.57667	-5.25128	-0.13715	1.315392	-3.5963	2.095017	-0.57239
H	0.927754	-0.20037	-0.3653	0.167799	-4.02503	-0.0789	0.93033	-0.20435	-0.35683
H	-0.26696	-0.28272	-1.64335	0.339999	-2.72139	1.070418	-0.26236	-0.28736	-1.63665
H	-0.68545	-2.64817	-1.2105	1.059874	-1.17029	-0.77101	-0.6819	-2.65309	-1.20176
H	0.210507	-2.60856	0.288906	0.885478	-2.46844	-1.93152	0.21504	-2.61213	0.297091
H	3.611287	-2.99422	-2.88603	4.97715	-3.56523	-0.14031	3.614145	-2.99973	-2.87903
H	4.539967	-1.83173	-1.92505	5.523738	-1.91548	0.204477	4.54226	-1.8337	-1.92182
H	4.489228	-3.52348	-1.4394	4.933183	-2.93236	1.514787	4.493809	-3.52425	-1.43171
H	-0.30521	1.650454	0.384795	-3.0278	-3.1222	0.711958	-0.30106	1.644882	0.387286
H	-0.68489	-1.52827	1.999888	-1.40338	-2.62915	-2.39449	-0.68888	-1.5354	2.004981
H	0.341056	-0.08298	1.979319	-1.62898	-4.19917	-1.59585	0.339106	-0.09153	1.987993
H	-1.34655	0.034615	2.493406	-2.95162	-3.02941	-1.64959	-1.34945	0.028152	2.498319
H	2.092229	-2.02929	1.038166	1.966613	-0.82849	1.220416	2.09686	-2.02561	1.043606
H	3.747064	-2.62322	0.849158	3.594018	-0.79506	1.904047	3.751953	-2.61916	0.855404
H	-4.9044	-0.88268	2.560663	-4.33192	0.278274	-1.96133	-4.90099	-0.86929	2.570622
H	-3.29044	-1.61412	2.52052	-3.20266	-0.9483	-2.54175	-3.2985	-1.6117	2.517589
H	-4.52795	-2.12875	1.365126	-2.62038	0.671276	-2.15016	-4.54741	-2.11217	1.366447
H	3.758378	1.941941	2.22167	3.268429	3.473286	0.020959	3.763256	1.951252	2.214257
H	5.270993	1.308265	1.604025	4.175491	2.321689	-0.98484	5.273561	1.315318	1.593067
H	4.453792	3.023989	0.06303	2.306172	3.312623	-2.26184	4.450954	3.027117	0.05007
H	4.413956	1.478368	-0.75703	2.076239	1.564324	-2.07814	4.409127	1.479077	-0.76523
H	0.551802	3.728657	-1.24838	-0.45706	5.236271	0.724586	0.542398	3.724583	-1.24903
H	1.055613	3.335977	-2.91963	-1.42337	4.193231	-0.36234	1.043976	3.333816	-2.92139
H	0.368349	2.065716	-1.86378	-0.73042	3.516831	1.135302	0.361631	2.061511	-1.86501
	6a-7			6a-8			6a-9		
C	-1.74963	-0.77101	0.059002	-2.47477	-0.5925	-0.10109	-1.56794	-1.0345	-0.14243
C	-3.18319	-0.18462	0.004764	-3.58663	-0.12356	0.881981	-3.03509	-0.58464	-0.40027
C	-3.12586	1.006552	0.995911	-4.80361	0.236025	-0.00625	-3.3182	0.529408	0.639745
C	-2.29103	0.439259	2.144711	-4.13823	0.78006	-1.27158	-2.48595	0.079022	1.842993
C	-1.19796	-0.39834	1.465806	-2.96425	-0.17155	-1.51533	-1.1766	-0.42667	1.233113
O	-2.46906	2.137174	0.407008	-5.59241	-0.92443	-0.32057	-2.87867	1.812248	0.17913
C	-4.491	1.509492	1.437548	-5.7689	1.212913	0.644466	-4.79575	0.69709	0.959969
C	-1.59119	-2.27086	-0.25581	-1.06879	-0.05094	0.228572	-1.3466	-2.55902	-0.21796

C	-0.11016	-2.69819	-0.15379	-0.00164	-0.59656	-0.74225	0.129798	-2.93686	0.021396
C	0.884193	-1.86396	-0.97492	0.137606	-2.12577	-0.83038	1.15831	-2.27644	-0.90735
C	2.288939	-2.39991	-0.8777	1.419707	-2.51609	-1.52699	2.560244	-2.72852	-0.5913
C	3.20144	-2.02528	0.027646	2.64927	-2.46251	-0.99689	3.410642	-2.1139	0.240546
C	4.602977	-2.57459	0.052341	3.891334	-2.79454	-1.78086	4.811701	-2.60522	0.490289
O	-2.33064	-2.97506	0.754376	-1.13134	1.370823	0.061399	-2.12385	-3.12875	0.848436
C	-2.15154	-2.65333	-1.62945	-0.65453	-0.35683	1.67378	-1.82812	-3.14342	-1.55016
C	2.87432	-1.00371	1.088108	2.876492	-2.04236	0.42937	3.010533	-0.85885	0.971432
O	3.050412	0.316201	0.518426	3.420481	-0.69703	0.410444	3.346404	0.273236	0.12867
C	2.784532	1.346494	1.333659	3.584014	-0.1104	1.603283	2.918383	1.468052	0.550438
O	2.489809	1.217534	2.504348	3.315621	-0.64918	2.658288	2.340284	1.647012	1.604829
H	-1.16167	-0.2372	-0.69461	-2.40518	-1.68494	-0.04754	-0.94134	-0.58705	-0.91942
C	-3.68055	0.23834	-1.37627	-3.91674	-1.12129	1.989499	-3.31946	-0.15055	-1.83714
C	2.939431	2.68127	0.640154	4.177667	1.276854	1.499402	3.178478	2.54737	-0.47445
C	2.621791	2.676335	-0.84899	4.038321	1.962206	0.148208	2.069591	2.553497	-1.54254
C	1.200846	2.261601	-1.13775	2.604493	2.243764	-0.22652	0.70148	2.769691	-0.94094
O	0.93085	2.283535	-2.44187	2.49556	2.651389	-1.4886	0.595014	3.957343	-0.35237
O	0.388937	1.942845	-0.28656	1.656681	2.1325	0.533911	-0.19701	1.945661	-0.97722
C	-0.39883	1.894107	-2.82306	1.175516	2.996234	-1.94583	-0.64182	4.230277	0.330277
H	2.568744	-3.16126	-1.60489	1.33191	-2.8202	-2.5689	2.891076	-3.64623	-1.07638
H	-3.87394	-0.92357	0.428695	-3.25712	0.817035	1.336453	-3.69758	-1.41818	-0.14193
H	-2.9269	-0.20384	2.76124	-3.75615	1.785445	-1.07076	-2.99906	-0.74714	2.346012
H	-1.89787	1.235637	2.781762	-4.84283	0.836677	-2.10568	-2.35084	0.891317	2.562461
H	-0.2688	0.170325	1.371534	-3.28676	-1.04949	-2.08669	-0.4723	0.398808	1.089664
H	-0.97667	-1.28545	2.060202	-2.18345	0.309472	-2.10482	-0.69166	-1.14707	1.893223
H	-1.53955	1.921559	0.223709	-5.02431	-1.57589	-0.75249	-1.96768	1.749564	-0.15537
H	-4.37544	2.277901	2.206732	-6.58154	1.45584	-0.04556	-4.92966	1.477435	1.714445
H	-5.03518	1.951954	0.598959	-6.20877	0.782595	1.548251	-5.3566	0.989609	0.06782
H	-5.08909	0.692492	1.848992	-5.25337	2.137623	0.914057	-5.21588	-0.2347	1.346558
H	-0.03422	-3.74982	-0.46025	0.959838	-0.17705	-0.43025	0.220086	-4.02762	-0.06867
H	0.188248	-2.66876	0.897913	-0.19028	-0.20055	-1.74496	0.38691	-2.7012	1.057846
H	0.861606	-0.82488	-0.63747	-0.70945	-2.54427	-1.38045	1.086933	-1.18865	-0.83177
H	0.584166	-1.85479	-2.02802	0.097905	-2.56778	0.169283	0.936249	-2.52646	-1.94943
H	4.776816	-3.27524	-0.76665	3.655918	-3.06842	-2.81106	5.040074	-3.49185	-0.10434
H	5.333967	-1.76298	-0.0297	4.574147	-1.93892	-1.80194	5.541361	-1.82775	0.239844
H	4.804834	-3.09169	0.996246	4.433533	-3.6262	-1.31854	4.957792	-2.85247	1.54698
H	-2.23248	-3.92186	0.587602	-0.25513	1.730058	0.284087	-2.01917	-4.08869	0.810962
H	-1.76461	-2.01045	-2.4247	-0.7119	-1.42393	1.905099	-1.3651	-2.6425	-2.40425
H	-1.88422	-3.68743	-1.87322	0.374853	-0.02569	1.847548	-1.57909	-4.20891	-1.60975
H	-3.24026	-2.58406	-1.62639	-1.2966	0.178243	2.37641	-2.91181	-3.04845	-1.64197
H	1.846527	-1.08394	1.446683	1.963483	-2.03548	1.023929	1.942842	-0.81866	1.191973
H	3.537754	-1.10108	1.950324	3.602555	-2.69182	0.926338	3.552087	-0.75584	1.914831
H	-4.66629	0.707751	-1.30538	-4.73153	-0.75483	2.620989	-4.34279	0.22289	-1.94068

H	-3.77245	-0.60211	-2.06447	-3.05358	-1.30084	2.633989	-3.19762	-0.97973	-2.53758
H	-3.0026	0.970115	-1.82215	-4.22917	-2.08336	1.573756	-2.64163	0.649201	-2.14752
H	2.311642	3.391463	1.177824	3.728918	1.872195	2.294695	3.21228	3.506201	0.041085
H	3.97735	2.995737	0.782323	5.239234	1.175368	1.742372	4.13539	2.368044	-0.9656
H	2.771234	3.674288	-1.26864	4.559008	2.923914	0.169563	2.271277	3.360173	-2.25039
H	3.285009	2.006309	-1.39965	4.49626	1.380256	-0.65279	2.050756	1.609242	-2.08568
H	-1.13979	2.498912	-2.29902	0.749644	3.77884	-1.31714	-0.52592	5.226094	0.749521
H	-0.4564	2.06211	-3.89532	1.307702	3.354802	-2.96326	-1.47656	4.203493	-0.37021
H	-0.55722	0.838162	-2.59746	0.527349	2.119825	-1.93068	-0.80199	3.49816	1.122021
	6a-10								
C	-2.4736	-0.5976	-0.10505						
C	-3.58499	-0.12794	0.875018						
C	-4.79662	0.241502	-0.01532						
C	-4.13158	0.772644	-1.27921						
C	-2.96527	-0.18673	-1.51897						
O	-5.51895	-0.93314	-0.42245						
C	-5.75471	1.232359	0.63715						
C	-1.07009	-0.05363	0.228525						
C	0.000514	-0.59419	-0.74111						
C	0.140238	-2.123	-0.83446						
C	1.42254	-2.51059	-1.53205						
C	2.652206	-2.45825	-1.00195						
C	3.894407	-2.78769	-1.7869						
O	-1.13258	1.36958	0.067045						
C	-0.65787	-0.36374	1.673884						
C	2.879329	-2.04202	0.425396						
O	3.4237	-0.6966	0.410226						
C	3.586192	-0.11222	1.604015						
O	3.317466	-0.65274	2.65816						
H	-2.40724	-1.68913	-0.04898						
C	-3.92052	-1.13447	1.97413						
C	4.179282	1.275541	1.502621						
C	4.03938	1.962777	0.152416						
C	2.605134	2.243781	-0.22156						
O	2.494891	2.646467	-1.48509						
O	1.658432	2.136549	0.540775						
C	1.174502	2.990703	-1.94205						
H	1.334904	-2.81178	-2.57484						
H	-3.25702	0.807099	1.342266						
H	-3.75211	1.77947	-1.08166						
H	-4.83746	0.824572	-2.1126						
H	-3.31162	-1.0681	-2.06424						
H	-2.18376	0.283675	-2.11719						

H	-5.94566	-1.3069	0.359512						
H	-6.56517	1.481085	-0.05333						
H	-6.20107	0.814321	1.546331						
H	-5.23589	2.154331	0.912193						
H	0.961241	-0.17543	-0.42543						
H	-0.18683	-0.19484	-1.74267						
H	-0.70703	-2.5393	-1.38558						
H	0.100678	-2.56829	0.163788						
H	3.658847	-3.05916	-2.81772						
H	4.576821	-1.93166	-1.80618						
H	4.437212	-3.62025	-1.32684						
H	-0.25612	1.727557	0.290145						
H	-0.71659	-1.43163	1.901703						
H	0.371417	-0.03396	1.851408						
H	-1.30073	0.169935	2.377078						
H	1.96613	-2.03638	1.019654						
H	3.605349	-2.6927	0.920879						
H	-4.75708	-0.79163	2.593266						
H	-3.07199	-1.29391	2.64229						
H	-4.18312	-2.10889	1.549575						
H	3.7301	1.869411	2.298779						
H	5.240903	1.174243	1.745451						
H	4.559086	2.925012	0.175386						
H	4.497848	1.382461	-0.64941						
H	0.750581	3.776811	-1.31638						
H	1.305683	3.344304	-2.96135						
H	0.525241	2.115258	-1.92178						

Table S 41. Energy analysis for 2*S*, 3*R*, 6*R*, 7*R*-6 in the Chloroform at MPW1PW91/6-31G+d, p level

Conf.	Steric Energy	Relative Energy	Distribution
	(kJ/mol)	(kJ/mol)	(%)
6a-1	-1233.284625	0.000307	20.02%
6a-2	-1233.282722	0.00221	2.67%
6a-3	-1233.282365	0.002567	1.83%
6a-4	-1233.283296	0.001636	4.90%
6a-5	-1233.282801	0.002131	2.90%
6a-6	-1233.284482	0.00045	17.20%
6a-7	-1233.283484	0.001448	5.98%
6a-8	-1233.284287	0.000645	13.99%
6a-9	-1233.282767	0.002165	2.80%
6a-10	-1233.284932	0	27.70%

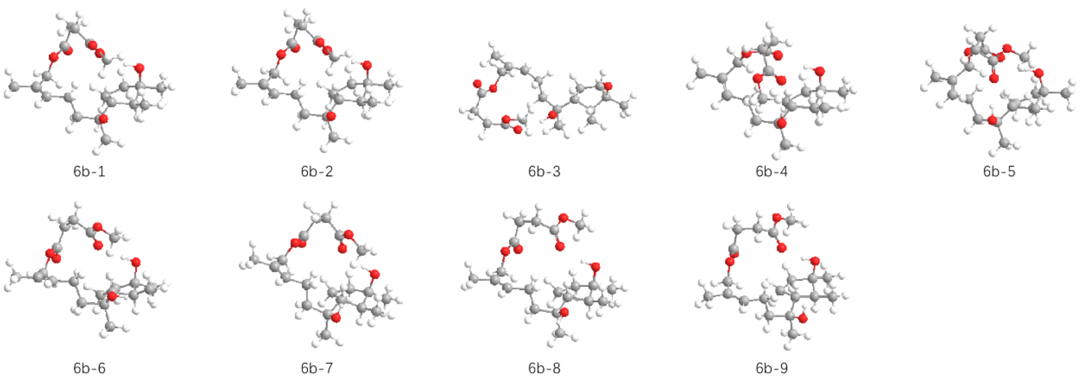

Figure S 98. Most stable conformers of 2*S*, 3*R*, 6*R*, 7*S*-6 calculated at MPW1PW91/6-31G+d, p level

Table S 42. Optimized Z-Matrixes of 2*S*, 3*R*, 6*R*, 7*S*-6 conformations in the Chloroform at MPW1PW91/6-31G+d, p level

	6b-1			6b-2			6b-3		
C	-1.77629	0.761291	-0.18637	-2.43123	0.483992	-0.16265	-2.72255	0.208865	0.113169
C	-3.22154	0.232832	-0.25034	-3.37443	-0.74305	-0.00935	-3.94563	-0.72782	0.05425
C	-3.04792	-1.13003	-0.96456	-4.81283	-0.1728	-0.07341	-5.06404	0.191456	-0.49222
C	-2.02642	-0.80691	-2.07252	-4.65174	1.207675	0.57257	-4.79367	1.539799	0.21194
C	-1.19808	0.396039	-1.57056	-3.33615	1.735379	-0.00709	-3.31821	1.518162	0.667775
O	-2.53205	-2.12022	-0.06686	-5.27316	-0.03273	-1.42778	-4.97049	0.334988	-1.91887
C	-4.34754	-1.7062	-1.50642	-5.84813	-1.04894	0.612438	-6.46873	-0.3355	-0.24279
C	-1.56076	2.224801	0.241039	-1.21864	0.471075	0.79048	-1.47085	-0.30073	0.856138
C	-0.09612	2.661935	0.035754	-0.22121	1.601474	0.460626	-0.44181	0.828003	1.074507
C	0.937937	1.794285	0.764597	0.265982	1.622226	-0.99737	0.068453	1.515017	-0.19988
C	2.32907	2.355621	0.646892	1.621367	2.263037	-1.09741	1.02689	2.616655	0.154962
C	3.270623	1.94937	-0.2139	2.741503	1.697836	-1.56708	2.327294	2.725059	-0.14603
C	4.654328	2.541782	-0.25355	4.084439	2.376112	-1.49978	3.180171	3.854687	0.373317
O	-1.85471	2.262492	1.649148	-0.5748	-0.79088	0.578318	-0.89644	-1.3015	0.008701
C	-2.48965	3.196232	-0.49159	-1.61098	0.586701	2.269451	-1.80658	-0.92944	2.213338
C	2.994311	0.842869	-1.2018	2.733988	0.305855	-2.14753	3.057538	1.722059	-0.99705
O	3.072468	-0.42224	-0.50226	2.827347	-0.63132	-1.0468	3.632412	0.72539	-0.11457
C	2.758204	-1.51439	-1.21211	2.390902	-1.88015	-1.24841	4.660828	0.005353	-0.58419
O	2.483708	-1.49177	-2.39482	1.967589	-2.29098	-2.30928	5.191855	0.197942	-1.65857
H	-1.25331	0.161861	0.565259	-2.01791	0.460172	-1.17585	-2.38492	0.3786	-0.91953
C	-3.97	0.156051	1.074139	-3.09289	-1.86696	-1.00173	-3.76525	-2.03022	-0.7153
C	2.828984	-2.77425	-0.37818	2.460759	-2.70827	0.016228	5.087868	-1.06614	0.395546
C	2.507391	-2.58685	1.098767	3.442183	-2.19858	1.071002	3.974766	-1.55795	1.317886
C	1.111099	-2.06216	1.322488	3.045888	-0.85525	1.634124	2.710826	-1.98779	0.612186
O	0.855336	-1.85076	2.61021	4.0707	-0.0123	1.726099	2.908774	-2.27763	-0.67224
O	0.306311	-1.85894	0.428454	1.916056	-0.57287	2.000508	1.627414	-2.07461	1.166207
C	-0.45192	-1.34373	2.937755	3.759513	1.313015	2.18563	1.760769	-2.6776	-1.44174
H	2.573711	3.173048	1.325036	1.695574	3.270297	-0.68501	0.596582	3.400891	0.77965
H	-3.79067	0.866283	-0.94484	-3.25406	-1.15513	0.997181	-4.2357	-0.96363	1.086913

H	-2.55399	-0.55097	-2.99527	-4.56153	1.087779	1.657863	-5.46058	1.657665	1.069686
H	-1.41152	-1.68477	-2.28546	-5.51238	1.851027	0.371947	-5.01269	2.359707	-0.4763
H	-0.13535	0.151637	-1.50929	-3.51004	2.201541	-0.98275	-2.76633	2.398569	0.329688
H	-1.29403	1.234968	-2.2664	-2.89518	2.514462	0.618304	-3.26774	1.517422	1.76072
H	-1.58954	-1.94671	0.091951	-4.60557	0.44631	-1.93605	-4.10588	0.707332	-2.13561
H	-4.14233	-2.60359	-2.09681	-6.83681	-0.58616	0.54984	-7.2095	0.395485	-0.57863
H	-5.01775	-1.98558	-0.68912	-5.90105	-2.03119	0.13531	-6.6383	-1.26609	-0.79042
H	-4.85908	-0.97992	-2.14355	-5.59577	-1.18729	1.666602	-6.625	-0.52316	0.822294
H	0.135717	2.686218	-1.03435	-0.65086	2.573224	0.725889	-0.86641	1.576783	1.752395
H	-0.00148	3.698313	0.387179	0.64088	1.458789	1.121667	0.420414	0.396603	1.597716
H	0.665913	1.741123	1.823589	0.292913	0.59671	-1.36984	0.52325	0.76548	-0.85071
H	0.90651	0.770679	0.385873	-0.44883	2.174578	-1.61806	-0.77154	1.950158	-0.75289
H	4.784487	3.313682	0.507464	4.01546	3.362861	-1.03722	2.616863	4.517181	1.033292
H	5.409181	1.765552	-0.08746	4.784844	1.770896	-0.91276	4.035433	3.461884	0.933716
H	4.863796	2.985885	-1.2324	4.521252	2.493228	-2.4971	3.58501	4.451637	-0.45066
H	-1.76761	3.180115	1.941706	0.231537	-0.79888	1.123523	-0.10663	-1.65016	0.458681
H	-3.53201	3.003354	-0.22889	-2.25663	-0.23959	2.574775	-2.41261	-1.82836	2.082217
H	-2.25643	4.229975	-0.21514	-0.71049	0.551782	2.890504	-0.88506	-1.21843	2.728205
H	-2.38424	3.113421	-1.5767	-2.12598	1.526849	2.484649	-2.34811	-0.2357	2.862841
H	2.002803	0.927284	-1.65074	1.832286	0.088374	-2.7183	2.409962	1.201473	-1.70379
H	3.727847	0.836332	-2.01107	3.595582	0.13871	-2.79826	3.863312	2.197034	-1.5591
H	-4.95563	-0.30079	0.939945	-3.79505	-2.69628	-0.87222	-4.70808	-2.58265	-0.77394
H	-4.10685	1.149568	1.50408	-2.07921	-2.24363	-0.85377	-3.02139	-2.66545	-0.23223
H	-3.41336	-0.44772	1.795594	-3.1783	-1.51928	-2.03557	-3.4231	-1.83673	-1.73504
H	2.160507	-3.4994	-0.8415	1.445562	-2.74778	0.421053	5.513068	-1.87894	-0.19226
H	3.846276	-3.1642	-0.47571	2.718197	-3.72644	-0.27699	5.898242	-0.65091	1.001204
H	2.591315	-3.54181	1.624238	3.450291	-2.90156	1.908541	4.328665	-2.41877	1.892242
H	3.208275	-1.90397	1.58203	4.455753	-2.14256	0.67489	3.683224	-0.79542	2.039683
H	-0.5014	-1.35333	4.023531	4.708999	1.842074	2.210424	2.150286	-2.94881	-2.41927
H	-0.56412	-0.3248	2.564533	3.07404	1.79259	1.484138	1.269846	-3.53133	-0.97411
H	-1.22848	-1.98171	2.514463	3.312833	1.279802	3.179705	1.058613	-1.84759	-1.52687
	6b-4			6b-5			6b-6		
C	1.656458	-0.28324	0.704367	-1.14783	-1.34054	-0.06534	-1.76726	0.765815	-0.20389
C	3.02594	0.232395	0.228221	-2.65489	-1.11444	0.137788	-3.21601	0.245116	-0.28828
C	3.281679	-0.63305	-1.03212	-2.68253	-0.46576	1.544297	-3.03389	-1.13626	-0.96419
C	2.812717	-2.03285	-0.58596	-1.67748	-1.33125	2.332235	-1.9715	-0.85224	-2.04691
C	1.774904	-1.81199	0.536178	-0.7159	-1.93975	1.286814	-1.18861	0.395315	-1.58388
O	2.513884	-0.16738	-2.14888	-2.24344	0.901974	1.494817	-2.56396	-2.10516	-0.02043
C	4.730201	-0.61321	-1.4979	-4.06497	-0.42757	2.176628	-4.32025	-1.71147	-1.53851
C	1.128034	0.182177	2.082344	-0.66782	-2.1056	-1.31778	-1.54311	2.227628	0.243439
C	-0.15128	-0.59741	2.469996	0.841508	-2.41917	-1.21366	-0.08387	2.662268	0.039561
C	-1.30754	-0.37527	1.488572	1.726281	-1.16979	-1.09707	0.953898	1.789466	0.758345
C	-2.4684	-1.30832	1.664333	3.147971	-1.4932	-0.74123	2.344929	2.346967	0.62211

C	-2.9486	-2.14117	0.731349	3.791809	-1.11916	0.372938	3.278324	1.93335	-0.244
C	-4.14327	-3.02711	0.971252	5.232735	-1.47249	0.635426	4.661533	2.525662	-0.30162
O	0.816595	1.582943	2.039259	-0.89651	-1.31345	-2.49074	-1.74833	2.33726	1.664629
C	2.165589	0.031759	3.191889	-1.43687	-3.40449	-1.54513	-2.4699	3.211444	-0.47514
C	-2.30174	-2.26931	-0.63165	3.112089	-0.29309	1.442983	2.99276	0.81942	-1.22085
O	-2.42782	-1.03276	-1.3835	2.991498	1.084726	0.998013	3.058151	-0.44006	-0.50945
C	-1.35553	-0.57992	-2.03909	1.795876	1.673613	1.023078	2.727735	-1.5353	-1.20641
O	-0.2826	-1.15799	-2.06729	0.781909	1.164698	1.472256	2.444802	-1.52164	-2.38737
H	0.932415	0.077247	-0.03623	-0.70348	-0.34125	-0.14286	-1.25501	0.156419	0.54724
C	3.132795	1.734167	-0.00642	-3.38279	-0.3385	-0.95122	-4.003	0.196084	1.018354
C	-1.63481	0.713659	-2.76748	1.83839	3.023567	0.351652	2.790467	-2.78768	-0.36042
C	-2.44735	1.734067	-1.9782	1.402883	2.890255	-1.11904	2.48858	-2.58068	1.118016
C	-1.69431	2.302287	-0.80027	-0.06756	2.577247	-1.276	1.099749	-2.04171	1.353282
O	-2.46589	3.088233	-0.05237	-0.82714	3.541725	-0.76222	0.872943	-1.78479	2.638127
O	-0.5189	2.084348	-0.56055	-0.51159	1.583401	-1.82195	0.275517	-1.86705	0.470853
C	-1.83422	3.727085	1.072545	-2.25429	3.350707	-0.81445	-0.42496	-1.26303	2.976845
H	-2.96996	-1.27565	2.631547	3.69557	-2.09031	-1.47066	2.595837	3.169674	1.291333
H	3.78638	-0.06923	0.962106	-3.13673	-2.0962	0.25199	-3.7658	0.866086	-1.00863
H	3.665734	-2.6058	-0.21256	-2.20955	-2.11773	2.873711	-2.45986	-0.66763	-3.00717
H	2.406044	-2.58114	-1.43944	-1.16155	-0.72099	3.078045	-1.32893	-1.72636	-2.17654
H	0.810455	-2.26587	0.297807	0.328997	-1.72372	1.515764	-0.11614	0.197562	-1.53127
H	2.12213	-2.27296	1.465855	-0.81856	-3.02922	1.26746	-1.33081	1.216323	-2.29345
H	1.589713	-0.45201	-2.05588	-1.2747	0.934139	1.445577	-1.61965	-1.9522	0.149242
H	4.872866	-1.32982	-2.31156	-3.99654	-0.08386	3.212502	-4.10203	-2.62513	-2.09799
H	5.00261	0.378019	-1.87006	-4.71896	0.260855	1.634806	-5.02494	-1.96587	-0.74225
H	5.405845	-0.87578	-0.67952	-4.52224	-1.42044	2.168172	-4.79902	-0.99675	-2.21293
H	0.077908	-1.66562	2.53751	1.017487	-3.07346	-0.35346	0.13947	2.689268	-1.03186
H	-0.45516	-0.27514	3.472168	1.131573	-2.98576	-2.1055	0.005891	3.692151	0.403453
H	-1.67105	0.651592	1.605923	1.710802	-0.63347	-2.05266	0.695744	1.743376	1.820414
H	-0.92806	-0.44799	0.472044	1.301283	-0.48938	-0.36005	0.914712	0.764703	0.38315
H	-4.54191	-2.89904	1.979345	5.659084	-2.05725	-0.18179	4.797849	3.304866	0.450791
H	-4.94053	-2.80348	0.254224	5.83568	-0.56659	0.760591	5.418355	1.751449	-0.13486
H	-3.88307	-4.08282	0.839624	5.332938	-2.05269	1.558908	4.862122	2.960762	-1.28637
H	0.448287	1.78993	1.164387	-0.70631	-0.3849	-2.28074	-2.66395	2.102289	1.858501
H	3.046417	0.642387	2.98253	-2.49943	-3.20395	-1.69874	-3.51856	3.021635	-0.22726
H	1.741703	0.366786	4.142552	-1.05643	-3.90944	-2.43732	-2.23379	4.23509	-0.17368
H	2.480259	-1.00853	3.307756	-1.32906	-4.08531	-0.6968	-2.3654	3.140184	-1.56132
H	-1.24485	-2.52283	-0.55694	2.121434	-0.66604	1.695802	2.002576	0.90925	-1.6715
H	-2.79522	-3.03874	-1.22804	3.711479	-0.26025	2.354054	3.726646	0.797377	-2.02951
H	4.107135	1.995266	-0.43139	-4.4106	-0.1109	-0.65075	-4.90831	-0.40749	0.911085
H	3.011765	2.28339	0.929488	-3.41931	-0.91544	-1.87747	-4.33955	1.191957	1.328028
H	2.359255	2.071691	-0.70111	-2.86995	0.599253	-1.16999	-3.4086	-0.24136	1.824664
H	-0.67453	1.124962	-3.07559	1.171867	3.703087	0.882214	2.107179	-3.50839	-0.80876

H	-2.18875	0.449452	-3.67291	2.854101	3.415908	0.386667	3.80122	-3.19234	-0.46542
H	-2.71296	2.57324	-2.62671	1.595847	3.840724	-1.62171	2.568786	-3.53111	1.652463
H	-3.38704	1.311974	-1.61821	1.974568	2.113607	-1.6276	3.201882	-1.90017	1.585871
H	-2.59728	4.368472	1.505368	-2.68091	4.291123	-0.47358	-0.44755	-1.22853	4.063024
H	-1.50666	2.982983	1.799767	-2.56864	3.136629	-1.8364	-0.5454	-0.25938	2.565937
H	-0.9798	4.31746	0.741009	-2.53739	2.534969	-0.14726	-1.21182	-1.91728	2.599598
	6b-7			6b-8			6b-9		
C	1.784574	0.800007	0.127977	-1.85746	-0.94477	-0.02481	-1.84496	-0.95837	-0.02035
C	3.222511	0.255322	0.21779	-3.30064	-0.40311	0.019468	-3.29046	-0.42241	0.014938
C	3.027252	-1.0985	0.943032	-3.1126	1.035435	0.55633	-3.11408	1.016233	0.554951
C	2.011253	-0.7447	2.042417	-2.07935	0.83568	1.677325	-2.08394	0.821405	1.679577
C	1.15154	0.410336	1.485114	-1.20272	-0.36171	1.251578	-1.19312	-0.36521	1.253394
O	2.494027	-2.0856	0.051458	-2.60697	1.904181	-0.46255	-2.61197	1.888623	-0.46268
C	4.316695	-1.69007	1.492113	-4.40141	1.680228	1.044413	-4.40908	1.650899	1.03989
C	1.594386	2.279485	-0.27339	-1.68228	-2.46752	-0.1924	-1.66226	-2.48476	-0.18938
C	0.114716	2.702159	-0.15944	-0.21727	-2.89889	0.017025	-0.19173	-2.9073	0.004114
C	-0.87824	1.83046	-0.94217	0.808205	-2.19956	-0.88464	0.829556	-2.21426	-0.90873
C	-2.27993	2.379441	-0.88057	2.17361	-2.81685	-0.73378	2.200909	-2.81568	-0.73982
C	-3.21653	2.032293	0.010856	3.150034	-2.39733	0.081019	3.161566	-2.38232	0.086046
C	-4.6099	2.602537	-0.00161	4.499585	-3.06171	0.152773	4.516225	-3.03331	0.178749
O	2.024906	2.486107	-1.63091	-2.05507	-2.75544	-1.55077	-2.08581	-2.89113	-1.50245
C	2.453696	3.233189	0.55304	-2.58035	-3.27142	0.753377	-2.5365	-3.29208	0.768025
C	-2.92311	1.023931	1.093236	2.960238	-1.19507	0.966652	2.947115	-1.18033	0.966137
O	-3.05455	-0.30357	0.52924	3.403152	-0.03006	0.226401	3.400956	-0.01384	0.234903
C	-2.74462	-1.32221	1.342728	3.117835	1.162021	0.75995	3.088548	1.177082	0.755775
O	-2.42336	-1.17992	2.505136	2.584828	1.312805	1.841727	2.520533	1.326173	1.819821
H	1.277228	0.2081	-0.64807	-1.37124	-0.49611	-0.90002	-1.35335	-0.49214	-0.88758
C	3.981246	0.158181	-1.09971	-4.08848	-0.49312	-1.281	-4.06881	-0.51408	-1.29165
C	-2.89008	-2.66373	0.660353	3.501712	2.283569	-0.17788	3.484621	2.299472	-0.17558
C	-2.59756	-2.66613	-0.83417	2.556656	2.344687	-1.39244	2.543763	2.365517	-1.39333
C	-1.18062	-2.25749	-1.14733	1.137024	2.628495	-0.96684	1.12138	2.638538	-0.97004
O	-0.91594	-2.32293	-2.44968	0.957308	3.911605	-0.66221	0.939966	3.912722	-0.63411
O	-0.36654	-1.90576	-0.3096	0.265852	1.779008	-0.88149	0.249543	1.78656	-0.91264
C	0.411499	-1.94687	-2.85434	-0.34176	4.287601	-0.17202	-0.36026	4.278089	-0.13898
H	-2.53419	3.132718	-1.62532	2.362665	-3.70493	-1.33683	2.405085	-3.70448	-1.33614
H	3.791818	0.889739	0.91003	-3.84884	-0.94161	0.804229	-3.84087	-0.96681	0.79332
H	2.547313	-0.42235	2.939232	-2.60175	0.612426	2.611886	-2.60902	0.587535	2.609888
H	1.420027	-1.62211	2.316111	-1.50367	1.750271	1.841993	-1.51837	1.74064	1.852922
H	0.109913	0.106949	1.362781	-0.17272	-0.0527	1.059764	-0.16767	-0.04414	1.0566
H	1.158787	1.255125	2.178944	-1.16826	-1.10235	2.055428	-1.14601	-1.1057	2.056221
H	1.540611	-1.93628	-0.06042	-1.65498	1.747952	-0.58118	-1.65638	1.748997	-0.57307
H	4.095492	-2.57398	2.096695	-4.18351	2.639061	1.523534	-4.19913	2.6103	1.521195
H	4.979715	-1.99553	0.678279	-5.0798	1.867943	0.207994	-5.08617	1.835651	0.201787

H	4.843846	-0.96384	2.116385	-4.91031	1.037357	1.767526	-4.91577	1.003356	1.760257
H	-0.1813	2.721006	0.894953	0.063883	-2.75696	1.066032	0.102152	-2.75173	1.047732
H	0.043576	3.733822	-0.52117	-0.16014	-3.98136	-0.16107	-0.14211	-3.98754	-0.17298
H	-0.57601	1.788068	-1.9945	0.486256	-2.29652	-1.92542	0.530874	-2.34121	-1.95478
H	-0.86053	0.802693	-0.57131	0.839658	-1.12785	-0.67311	0.851649	-1.13721	-0.72217
H	-4.75434	3.297094	-0.83139	4.575724	-3.89638	-0.54686	4.611139	-3.86781	-0.51865
H	-5.35155	1.801453	-0.09156	5.29393	-2.3452	-0.08239	5.306783	-2.30902	-0.04486
H	-4.82489	3.133126	0.931896	4.697761	-3.43913	1.161531	4.702237	-3.40776	1.190847
H	1.723872	1.740018	-2.16451	-2.00072	-3.71324	-1.67168	-1.76041	-2.24359	-2.14084
H	3.514792	3.049487	0.372634	-3.63257	-3.12807	0.498913	-3.59428	-3.1453	0.541524
H	2.238235	4.267132	0.270657	-2.35683	-4.34078	0.674692	-2.31586	-4.35782	0.665667
H	2.256189	3.12402	1.621992	-2.43263	-2.97977	1.7969	-2.35789	-3.0056	1.807398
H	-1.91413	1.127502	1.495723	1.92054	-1.04568	1.259127	1.900859	-1.03542	1.236274
H	-3.62487	1.116024	1.925266	3.559245	-1.2686	1.877904	3.526532	-1.25072	1.890175
H	4.951666	-0.32746	-0.95978	-5.07553	-0.03215	-1.17299	-5.05296	-0.04543	-1.19535
H	4.154666	1.14852	-1.52434	-4.22538	-1.53314	-1.58125	-4.21597	-1.55506	-1.5851
H	3.415426	-0.43391	-1.82546	-3.55954	0.023194	-2.08607	-3.53333	-0.00155	-2.09605
H	-2.24453	-3.36214	1.192317	3.475845	3.22025	0.377794	3.457511	3.234972	0.381942
H	-3.9215	-2.99049	0.821089	4.517196	2.114215	-0.53975	4.500965	2.128058	-0.5335
H	-2.7577	-3.66484	-1.24765	2.894728	3.139356	-2.05903	2.880005	3.167521	-2.05208
H	-3.26738	-1.99542	-1.37616	2.567968	1.397459	-1.93026	2.562028	1.42297	-1.93919
H	0.602099	-0.90565	-2.59108	-0.27078	5.349979	0.045922	-0.29029	5.335886	0.100202
H	1.154274	-2.58821	-2.37939	-1.10334	4.100033	-0.92845	-1.12041	4.105274	-0.90035
H	0.432349	-2.0729	-3.93354	-0.57931	3.726385	0.731777	-0.59816	3.699228	0.753496

Table S 43. Energy analysis for 2*S*, 3*R*, 6*R*, 7*S*-6 in the Chloroform at MPW1PW91/6-31G+d, p level

Conf.	Steric Energy (kJ/mol)	Relative Energy (kJ/mol)	Distribution (%)
6b-1	-1233.285156	0.000422	21.06%
6b-2	-1233.283553	0.002025	3.86%
6b-3	-1233.282887	0.002691	1.91%
6b-4	-1233.284166	0.001412	7.38%
6b-5	-1233.283026	0.002552	2.21%
6b-6	-1233.285007	0.000571	17.99%
6b-7	-1233.285578	0	32.92%
6b-8	-1233.284347	0.001231	8.94%
6b-9	-1233.283521	0.002057	3.73%

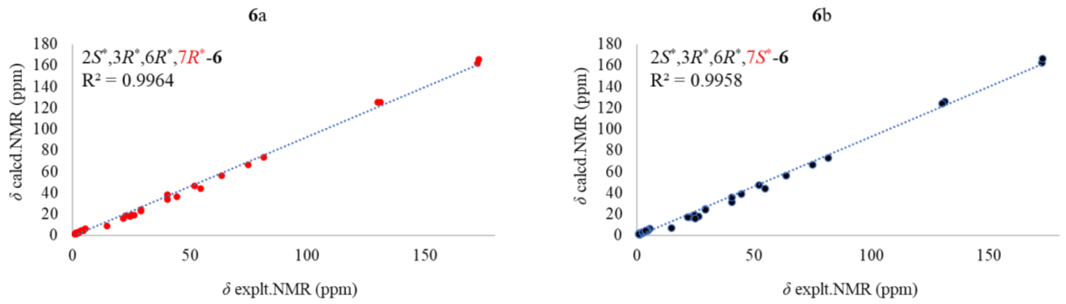

Figure S 99. Correlation between the calculated ^{13}C NMR data for 2*S*, 3*R*, 6*R*, 7*R*-6 (a) and 2*S*, 3*R*, 6*R*, 7*S*-6 (b) and experimental ^{13}C NMR data of 6

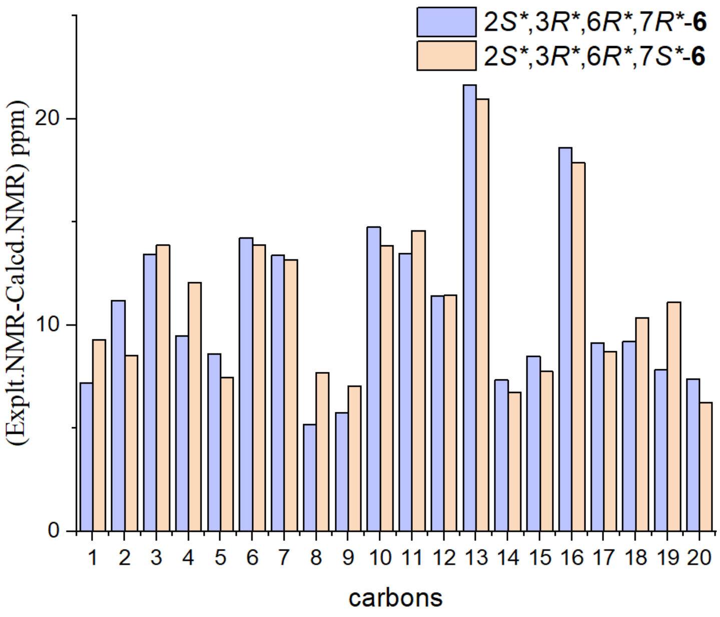

Figure S 100. Differences between experimental NMR chemical shifts of 6 and theoretical NMR chemical shifts for 2*S*, 3*R*, 6*R*, 7*R*-6 (purple bar) and 2*S*, 3*R*, 6*R*, 7*S*-6 (yellow bar)

Table S 44. Experimental chemical shifts of 6, the calculated shielding tensors for 2*S*, 3*R*, 6*R*, 7*R*-6 (isomer 1) and 2*S*, 3*R*, 6*R*, 7*S*-6 (isomer 2), as well as their DP4+ probability

Functional		Solvent?		Basis Set		Type of Data	
mPW1PW91		PCM		6-31+G(d,p)		Shielding Tensors	
		DP4+	99.14%	0.86%	-	-	-
Nuclei	sp2?	xperimenta	Isomer 1	Isomer 2	Isomer 3	Isomer 4	Isomer 5
C		54.6	144.7	144.3			
C		44.4	152.4	149.4			
C		81.4	115.3	115.7			
C		40.5	154.7	157.4			
C		24.5	170.9	169.5			
C		26.2	169.7	170.8			
C		74.8	122.3	122			
C		40.5	150.1	152.7			
C		22.5	169.9	171.2			
C	x	131.2	63.6	62.7			
C	x	129.9	63.5	64.8			
C		21.6	172.6	171.3			
C		25	169.5	172.9			
C		63.6	132.1	132.1			
C	x	172.4	26.9	26.4			
H		1.84	29.98	29.5			
C		14.7	179.8	181.9			
C		29.1	164.6	163.8			
C		29.3	165.6	164.7			
C	x	172.9	23.2	22.6			
C		52	142.1	141.6			
H	x	5.41	25.36	25.46			
H		1.6	29.64	30.07			
H		1.56	30.17	29.88			
H		1.68	29.9	29.87			
H		1.85	29.89	29.85			
H		1.55	29.9	30.48			
H		1.26	30.5	30.33			
H		1.26	30.4	30.26			
H		1.26	30.56	31.09			
H		1.49	30.76	30.34			
H		1.49	29.87	30.46			
H		2.12	29.82	29.75			
H		2.19	29.69	29.6			
H		1.74	29.96	29.96			
H		1.74	29.57	29.65			
H		1.74	29.9	29.87			
H		1.15	30.31	30.48			
H		1.15	30.66	30.85			
H		1.15	30.28	30.54			
H		4.6	26.62	26.72			
H		4.66	27.57	27.65			
H		1.04	30.65	30.66			
H		1.04	30.61	30.31			
H		1.04	30.8	30.8			
H		2.64	28.89	29.02			
H		2.64	29.25	29.26			
H		2.64	29.22	29.15			
H		2.64	28.76	28.74			
H		3.69	27.83	27.86			
H		3.69	27.96	27.55			
H		3.69	27.53	27.68			

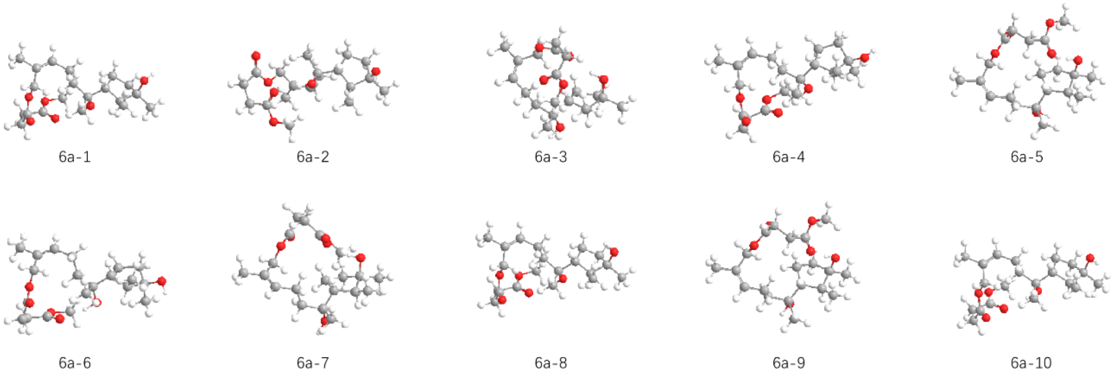

Figure S 101. Most stable conformers of 2*S*, 3*R*, 6*R*, 7*R*-6 at the CAM-B3LYP/DGDZVP level

Table S 45. Optimized Z-Matrixes of 2*S*, 3*R*, 6*R*, 7*R*-6 conformations in the methanol at CAM-B3LYP/DGDZVP level

	6a-1			6a-2			6a-3		
C	-2.47238	-0.58931	-0.10543	-2.56076	0.254676	0.933879	1.165022	-1.16846	0.183453
C	-3.58318	-0.1288	0.880954	-3.25273	0.448465	-0.43177	2.557406	-0.48105	0.161682
C	-4.79479	0.235907	0.000272	-4.64224	-0.19313	-0.22223	3.00521	-0.57399	-1.31749
C	-4.13332	0.788196	-1.26266	-4.30445	-1.45898	0.563278	2.524691	-1.97133	-1.71041
C	-2.96656	-0.16689	-1.51508	-3.12418	-1.08616	1.47914	1.135529	-2.08604	-1.07537
O	-5.47742	-0.99261	-0.31042	-5.45217	0.611426	0.652408	2.359079	0.4237	-2.12364
C	-5.76566	1.208869	0.660676	-5.40297	-0.47026	-1.5143	4.496564	-0.36897	-1.52872
C	-1.06723	-0.04871	0.226995	-1.02607	0.403565	0.961802	0.787292	-1.92248	1.475406
C	-0.00076	-0.58664	-0.74848	-0.28448	-0.28309	-0.19988	-0.68161	-2.39632	1.40638
C	0.133344	-2.1156	-0.84966	-0.42333	-1.80625	-0.35347	-1.72819	-1.27349	1.538126
C	1.418369	-2.5056	-1.54118	0.555366	-2.33022	-1.37747	-3.09568	-1.67886	1.049078
C	2.644999	-2.46187	-1.00262	1.869128	-2.51603	-1.1881	-3.54258	-1.53067	-0.20579
C	3.890847	-2.79592	-1.78001	2.795055	-2.97188	-2.28485	-4.93292	-1.91685	-0.63504
O	-1.12466	1.377218	0.074464	-0.78527	1.816782	0.837836	1.640696	-3.07866	1.524696
C	-0.65053	-0.3653	1.669518	-0.45669	-0.04226	2.314948	1.010407	-1.09013	2.740157
C	2.864399	-2.05022	0.427479	2.520369	-2.25909	0.143297	-2.64739	-0.95564	-1.27526
O	3.415566	-0.70679	0.41714	3.274643	-1.02391	0.030004	-2.73484	0.495402	-1.23924
C	3.567574	-0.11725	1.607427	3.826869	-0.55901	1.155301	-1.64555	1.183052	-1.57443
O	3.275854	-0.64874	2.66225	3.731726	-1.11853	2.231429	-0.60467	0.668821	-1.95116
H	-2.40557	-1.68107	-0.05732	-2.92519	1.053598	1.589391	0.417138	-0.37913	0.059681
C	-3.91014	-1.13532	1.982293	-3.27866	1.882268	-0.95298	2.587046	0.947744	0.696935
C	4.178294	1.262086	1.507374	4.6147	0.713822	0.943142	-1.84724	2.674986	-1.47161
C	4.045809	1.95191	0.157989	4.290653	1.494486	-0.32147	-0.64141	3.378176	-0.86084
C	2.615356	2.240699	-0.22196	2.878172	2.021958	-0.34517	-0.45074	3.02483	0.59483
O	2.512182	2.645219	-1.4845	2.549691	2.499599	-1.54215	0.426973	3.836248	1.190427
O	1.663084	2.135634	0.535737	2.126722	2.03104	0.617575	-1.0144	2.109184	1.164812
C	1.196994	3.001328	-1.94853	1.239055	3.079613	-1.67415	0.67215	3.589411	2.585071
H	1.335755	-2.80278	-2.58548	0.155526	-2.52522	-2.37138	-3.76594	-2.12513	1.782626
H	-3.25937	0.809796	1.344105	-2.75285	-0.17772	-1.18129	3.258048	-1.09585	0.739591

H	-3.75721	1.795379	-1.0574	-4.00919	-2.24041	-0.14357	3.200119	-2.71576	-1.27629
H	-4.83465	0.856298	-2.10119	-5.17803	-1.82421	1.108718	2.522722	-2.10592	-2.79539
H	-3.31175	-1.04453	-2.06733	-3.44588	-0.96469	2.51571	0.371008	-1.74057	-1.77622
H	-2.18707	0.308578	-2.11173	-2.38145	-1.88473	1.479177	0.902172	-3.12142	-0.82612
H	-6.13421	-0.79289	-0.99086	-5.62454	1.452453	0.208186	1.397352	0.39304	-1.98749
H	-6.58939	1.450428	-0.01934	-6.3285	-1.01006	-1.29569	4.749857	-0.51247	-2.58301
H	-6.1935	0.77408	1.568219	-5.66818	0.459944	-2.02704	4.799259	0.64196	-1.24283
H	-5.26277	2.142646	0.926203	-4.80288	-1.07401	-2.20046	5.070947	-1.08459	-0.93543
H	0.961889	-0.17264	-0.43222	0.779121	-0.05357	-0.07536	-0.84441	-3.13048	2.205384
H	-0.18864	-0.18126	-1.74745	-0.5861	0.197639	-1.13695	-0.83362	-2.93238	0.465141
H	-0.71188	-2.52481	-1.4089	-1.43832	-2.06492	-0.66678	-1.39619	-0.38064	1.004507
H	0.085289	-2.56654	0.145602	-0.26591	-2.29981	0.609448	-1.807	-0.97391	2.587015
H	3.660475	-3.06286	-2.81315	2.26199	-3.1177	-3.22626	-5.5205	-2.30128	0.200907
H	4.578645	-1.94409	-1.79164	3.588509	-2.23596	-2.4508	-5.45914	-1.05343	-1.05612
H	4.425546	-3.63249	-1.31804	3.283731	-3.91394	-2.0148	-4.9028	-2.68343	-1.4164
H	-0.23733	1.72631	0.268802	0.178623	1.950929	0.815876	1.460741	-3.54321	2.353058
H	-0.70511	-1.43433	1.891946	-0.56132	-1.11483	2.491926	0.497822	-0.12644	2.684873
H	0.378841	-0.03322	1.842977	0.609835	0.202216	2.364844	0.625267	-1.62525	3.615589
H	-1.29297	0.162312	2.377794	-0.96764	0.486596	3.124602	2.076328	-0.91161	2.89708
H	1.946719	-2.0413	1.014709	1.803304	-2.15142	0.956325	-1.6075	-1.24297	-1.13444
H	3.585154	-2.70438	0.925613	3.22057	-3.05684	0.405649	-2.95863	-1.27288	-2.27303
H	-4.72179	-0.77427	2.621661	-3.86742	1.963092	-1.87258	3.574596	1.399168	0.563191
H	-3.04379	-1.31645	2.622234	-2.26671	2.230847	-1.16463	2.353884	0.98676	1.761393
H	-4.21928	-2.0917	1.553987	-3.69957	2.567647	-0.20954	1.86394	1.574582	0.168278
H	3.741181	1.862712	2.305115	4.474573	1.326574	1.833871	-1.99167	3.042176	-2.49131
H	5.239354	1.145987	1.74561	5.668705	0.42321	0.924304	-2.75691	2.886347	-0.91081
H	4.570209	2.911182	0.185576	4.956614	2.358673	-0.39724	0.27555	3.109157	-1.39337
H	4.506029	1.371131	-0.64244	4.453322	0.898602	-1.22046	-0.74656	4.460874	-0.94695
H	0.773807	3.784921	-1.3195	1.108149	3.886551	-0.95269	1.017851	2.566827	2.735853
H	1.337597	3.362681	-2.96362	1.195102	3.465311	-2.6891	1.443425	4.297981	2.875305
H	0.544258	2.128235	-1.9422	0.472297	2.319847	-1.52182	-0.238	3.758759	3.161458
	6a-4			6a-5			6a-6		
C	-2.47171	-0.71157	0.017331	-1.56635	-1.04597	-0.14868	-2.47111	-0.71107	0.015872
C	-3.62141	-0.18841	0.929064	-3.03683	-0.60574	-0.40843	-3.6215	-0.18372	0.922981
C	-4.72668	0.311933	-0.04281	-3.32723	0.519781	0.618819	-4.7337	0.308661	-0.05744
C	-4.45412	-0.46345	-1.33826	-2.47695	0.099602	1.820581	-4.45058	-0.461	-1.34761
C	-2.93175	-0.48129	-1.43659	-1.16827	-0.40349	1.207175	-2.92831	-0.47671	-1.43797
O	-6.0202	0.015128	0.497056	-2.9127	1.801466	0.133403	-6.06341	-0.01873	0.358457
C	-4.6096	1.822388	-0.25732	-4.80467	0.669063	0.949142	-4.62885	1.820103	-0.26621
C	-1.08117	-0.13931	0.333411	-1.32921	-2.57645	-0.18477	-1.08027	-0.14079	0.33439
C	-0.02375	-0.64996	-0.66326	0.149861	-2.93091	0.044084	-0.02178	-0.65193	-0.66082
C	0.16803	-2.17362	-0.7478	1.159673	-2.24225	-0.88256	0.169414	-2.17573	-0.74413
C	1.431574	-2.51932	-1.4991	2.564897	-2.70507	-0.59785	1.43381	-2.52291	-1.49344

C	2.679869	-2.43143	-1.01882	3.434766	-2.10328	0.223537	2.681586	-2.43359	-1.01208
C	3.898704	-2.72042	-1.85486	4.835999	-2.60732	0.446213	3.901365	-2.72467	-1.84602
O	-1.16965	1.284789	0.187563	-1.99828	-3.21629	0.916883	-1.16804	1.28353	0.188936
C	-0.65825	-0.45898	1.772919	-1.82169	-3.19863	-1.49472	-0.65984	-0.46079	1.774503
C	2.951885	-2.01274	0.40017	3.055801	-0.85052	0.970101	2.952059	-2.01137	0.406167
O	3.453164	-0.6503	0.368157	3.360716	0.284264	0.117911	3.455029	-0.64967	0.370998
C	3.625705	-0.05318	1.552002	2.92428	1.474887	0.538007	3.626228	-0.04883	1.553128
O	3.390914	-0.59263	2.616948	2.355298	1.651057	1.599689	3.389352	-0.58455	2.619519
H	-2.40214	-1.79433	0.175166	-0.95316	-0.61195	-0.9439	-2.40473	-1.79422	0.173171
C	-4.1021	-1.26822	1.899043	-3.32675	-0.19909	-1.85197	-4.10546	-1.25713	1.897355
C	4.182889	1.346873	1.429788	3.160424	2.553043	-0.49309	4.184959	1.350252	1.426851
C	3.977587	2.030044	0.086244	2.035342	2.550874	-1.54325	3.979094	2.029975	0.081625
C	2.524916	2.267497	-0.24122	0.67304	2.779682	-0.93433	2.526198	2.267088	-0.24525
O	2.361928	2.672622	-1.49722	0.594544	3.94434	-0.29806	2.362359	2.670963	-1.5015
O	1.604953	2.124107	0.5494	-0.25024	1.984797	-1.00987	1.606712	2.124162	0.546055
C	1.018599	2.979596	-1.91342	-0.64441	4.232719	0.373838	1.018565	2.976332	-1.91742
H	1.310224	-2.81826	-2.53911	2.878924	-3.62143	-1.09624	1.313602	-2.82491	-2.53268
H	-3.26923	0.664375	1.515813	-3.71709	-1.42707	-0.1466	-3.26928	0.673372	1.504259
H	-4.83791	-1.48268	-1.21686	-2.97774	-0.72128	2.345427	-4.83429	-1.48096	-1.23135
H	-4.94625	-0.01801	-2.20869	-2.34534	0.924812	2.525686	-4.93994	-0.01334	-2.21718
H	-2.56411	-1.25537	-2.11531	-0.47517	0.426409	1.038413	-2.55785	-1.24744	-2.11887
H	-2.56193	0.479427	-1.80564	-0.66781	-1.10572	1.875393	-2.55872	0.48606	-1.80213
H	-6.67486	0.355473	-0.12723	-1.99566	1.757545	-0.18942	-6.27683	0.52108	1.131179
H	-5.32644	2.165841	-1.01177	-4.9454	1.459882	1.6914	-5.34036	2.152654	-1.02765
H	-4.81474	2.345153	0.681346	-5.37858	0.934503	0.056762	-4.84702	2.347832	0.669142
H	-3.6045	2.09829	-0.58401	-5.20688	-0.26166	1.356503	-3.61907	2.104858	-0.57064
H	0.932409	-0.19593	-0.38344	0.250525	-4.01752	-0.05427	0.934177	-0.19796	-0.38025
H	-0.26593	-0.26416	-1.65924	0.409688	-2.69886	1.081167	-0.26273	-0.26664	-1.65728
H	-0.68641	-2.62932	-1.25499	1.091472	-1.15669	-0.77768	-0.6846	-2.63131	-1.25217
H	0.191448	-2.6077	0.255363	0.923064	-2.46574	-1.92753	0.191184	-2.60928	0.259326
H	3.62958	-2.99416	-2.87681	5.047137	-3.49108	-0.15887	3.633345	-3.00158	-2.86741
H	4.554773	-1.84484	-1.89636	5.567978	-1.83361	0.190875	4.557156	-1.84898	-1.88952
H	4.48363	-3.5381	-1.42073	4.996633	-2.8634	1.49858	4.486154	-3.54082	-1.40884
H	-0.28293	1.650652	0.352189	-2.95176	-3.15301	0.771915	-0.28088	1.648972	0.352167
H	-0.67316	-1.53273	1.978376	-1.36964	-2.71557	-2.36468	-0.67636	-1.53448	1.980215
H	0.356113	-0.08968	1.959589	-1.57222	-4.26288	-1.51889	0.354703	-0.09268	1.96261
H	-1.32934	0.028524	2.484029	-2.90731	-3.10804	-1.59304	-1.33137	0.027751	2.48452
H	2.063808	-2.03771	1.030638	1.995641	-0.81477	1.224764	2.063042	-2.03356	1.035433
H	3.718805	-2.64079	0.861637	3.628875	-0.74703	1.894381	3.717545	-2.6391	0.870467
H	-4.87919	-0.89239	2.568269	-4.35345	0.162864	-1.96099	-4.87836	-0.87974	2.572641
H	-3.26364	-1.61666	2.509986	-3.19684	-1.04016	-2.5363	-3.27	-1.60887	2.510231
H	-4.5066	-2.13233	1.362647	-2.65387	0.59949	-2.17513	-4.51772	-2.11926	1.3641
H	3.753515	1.932555	2.242703	3.200259	3.513748	0.018219	3.756875	1.938546	2.238555

H	5.255261	1.268936	1.629749	4.109413	2.377089	-1.00001	5.257431	1.271673	1.626044
H	4.468994	3.006971	0.094701	2.229464	3.349435	-2.2624	4.471082	3.006622	0.086976
H	4.428299	1.464621	-0.73046	2.006569	1.602945	-2.0794	4.428759	1.462026	-0.73392
H	0.586261	3.740803	-1.26351	-0.50624	5.210132	0.82774	0.586426	3.73895	-1.26903
H	1.110206	3.354037	-2.92938	-1.46679	4.255391	-0.34128	1.109234	3.348342	-2.93436
H	0.401696	2.081054	-1.89305	-0.83973	3.480485	1.138627	0.402034	2.077605	-1.89443
	6a-7			6a-8			6a-9		
C	-1.71264	-0.76828	0.029681	-2.47191	-0.58904	-0.10707	-1.54237	-1.05435	-0.15175
C	-3.14875	-0.18429	-0.02323	-3.58404	-0.12811	0.879521	-3.02062	-0.63832	-0.40367
C	-3.12431	0.95891	1.023302	-4.80145	0.239709	-0.0049	-3.3296	0.470328	0.634933
C	-2.28908	0.35358	2.151952	-4.13787	0.787305	-1.26967	-2.46823	0.056605	1.831067
C	-1.16291	-0.40683	1.440346	-2.96378	-0.16277	-1.51851	-1.15196	-0.41512	1.209158
O	-2.48173	2.130431	0.498862	-5.59594	-0.91727	-0.32319	-2.94238	1.767818	0.164277
C	-4.50292	1.414601	1.473917	-5.76157	1.217083	0.653089	-4.80869	0.585891	0.971449
C	-1.55943	-2.26588	-0.30023	-1.06604	-0.04931	0.225066	-1.29365	-2.57593	-0.20679
C	-0.08122	-2.70356	-0.20079	-0.0002	-0.58854	-0.75037	0.189177	-2.92649	0.03407
C	0.922718	-1.85737	-0.99766	0.134991	-2.1176	-0.8493	1.205875	-2.2506	-0.8965
C	2.330265	-2.3791	-0.86677	1.420787	-2.50727	-1.53964	2.613618	-2.6892	-0.58678
C	3.211231	-2.00299	0.069296	2.646863	-2.46236	-1	3.461605	-2.06603	0.241626
C	4.61762	-2.53689	0.132527	3.893573	-2.79597	-1.77615	4.867518	-2.54487	0.488873
O	-2.30336	-2.9787	0.702615	-1.12538	1.375527	0.07113	-2.05883	-3.14749	0.868938
C	-2.12135	-2.62789	-1.67825	-0.65086	-0.3661	1.667807	-1.76782	-3.18421	-1.53084
C	2.839113	-0.99493	1.128335	2.864871	-2.04976	0.430071	3.05081	-0.81391	0.972243
O	3.006861	0.332809	0.571687	3.413253	-0.70524	0.419508	3.345103	0.319908	0.115018
C	2.678005	1.355164	1.369934	3.564281	-0.11547	1.609892	2.890857	1.506451	0.528041
O	2.310745	1.217489	2.5212	3.272853	-0.64744	2.66453	2.314679	1.678988	1.586458
H	-1.12331	-0.22998	-0.71901	-2.40066	-1.68128	-0.0599	-0.9306	-0.60916	-0.94171
C	-3.61808	0.295072	-1.39558	-3.91331	-1.135	1.97916	-3.32174	-0.21787	-1.84112
C	2.85723	2.693382	0.690806	4.173019	1.264726	1.510299	3.116846	2.584239	-0.50564
C	2.5994	2.692418	-0.80972	4.04184	1.954315	0.160717	1.987963	2.573487	-1.55166
C	1.192308	2.276761	-1.1576	2.611803	2.241743	-0.22153	0.62564	2.792782	-0.93903
O	0.990846	2.253587	-2.47256	2.510708	2.650421	-1.48281	0.542026	3.954383	-0.29763
O	0.333087	1.996012	-0.3384	1.657927	2.132023	0.533589	-0.29373	1.993783	-1.01676
C	-0.32419	1.874588	-2.9127	1.195831	3.004714	-1.94906	-0.69715	4.231929	0.378406
H	2.639676	-3.1295	-1.59307	1.33906	-2.80489	-2.58385	2.949967	-3.60431	-1.07274
H	-3.84462	-0.94437	0.35145	-3.25607	0.808614	1.343088	-3.66215	-1.48674	-0.14153
H	-2.91238	-0.34459	2.719912	-3.75843	1.793299	-1.06623	-2.95081	-0.77895	2.348439
H	-1.93048	1.122987	2.840724	-4.84318	0.846708	-2.10305	-2.34936	0.878697	2.542395
H	-0.26807	0.215372	1.348543	-3.28729	-1.0391	-2.09089	-0.47834	0.431342	1.042433
H	-0.88188	-1.29344	2.009733	-2.18407	0.319471	-2.1085	-0.63128	-1.1085	1.871033
H	-1.57295	1.917681	0.227397	-5.02967	-1.57446	-0.7496	-2.03129	1.740704	-0.17606
H	-4.411	2.161607	2.26757	-6.57738	1.464543	-0.0318	-4.96428	1.365754	1.722549
H	-5.05656	1.866506	0.646472	-6.1965	0.787338	1.559748	-5.39135	0.84817	0.083693

H	-5.08079	0.570615	1.858338	-5.2429	2.140303	0.921353	-5.18915	-0.35776	1.369782
H	-0.00961	-3.74838	-0.52898	0.962073	-0.17346	-0.43475	0.298177	-4.01531	-0.05337
H	0.211283	-2.69873	0.853027	-0.18801	-0.18458	-1.74993	0.442409	-2.68328	1.069934
H	0.882889	-0.81945	-0.65839	-0.70925	-2.5286	-1.40882	1.122597	-1.16386	-0.8163
H	0.646608	-1.84847	-2.05687	0.086878	-2.56725	0.146509	0.982744	-2.50012	-1.9383
H	4.823383	-3.22926	-0.68603	3.664404	-3.06284	-2.80956	5.101836	-3.43029	-0.10515
H	5.341839	-1.71685	0.079309	4.581097	-1.94393	-1.78685	5.590228	-1.7614	0.236902
H	4.79618	-3.0586	1.078507	4.42797	-3.63244	-1.31367	5.017337	-2.78889	1.545696
H	-2.21167	-3.924	0.522034	-0.23846	1.726418	0.264762	-1.94201	-4.10661	0.836819
H	-1.71367	-1.98935	-2.46644	-0.70683	-1.43493	1.890632	-1.31201	-2.68721	-2.39102
H	-1.87609	-3.66613	-1.92582	0.378741	-0.03514	1.841242	-1.50316	-4.24616	-1.5765
H	-3.20847	-2.53313	-1.68235	-1.29282	0.162747	2.375515	-2.85284	-3.106	-1.62404
H	1.80422	-1.09615	1.459204	1.946981	-2.04258	1.017014	1.986983	-0.79545	1.213007
H	3.483412	-1.08535	2.005519	3.586672	-2.70235	0.928707	3.609839	-0.69267	1.90292
H	-4.61564	0.74012	-1.3309	-4.73088	-0.77597	2.611644	-4.35512	0.127715	-1.94271
H	-3.67074	-0.51429	-2.12399	-3.05088	-1.31528	2.624313	-3.18083	-1.04759	-2.53718
H	-2.94307	1.058751	-1.78951	-4.21823	-2.09603	1.555477	-2.66478	0.5964	-2.15885
H	2.208921	3.402751	1.2044	3.733855	1.864847	2.307296	3.152058	3.545929	0.004173
H	3.888818	3.007261	0.873503	5.233855	1.150153	1.750291	4.06516	2.413794	-1.01565
H	2.760407	3.693864	-1.21636	4.565429	2.914003	0.18891	2.173997	3.372902	-2.27204
H	3.288499	2.030166	-1.33687	4.503685	1.373894	-0.63905	1.964078	1.625184	-2.08743
H	-1.07624	2.525101	-2.46507	0.768296	3.784007	-1.31767	-0.56414	5.208166	0.836396
H	-0.31506	1.989458	-3.99317	1.338286	3.371249	-2.96202	-1.52137	4.252567	-0.33468
H	-0.5235	0.835906	-2.64557	0.545886	2.129517	-1.94898	-0.88601	3.474952	1.140151
	6a-10								
C	-2.47166	-0.58631	-0.11315						
C	-3.58296	-0.12825	0.872462						
C	-4.7982	0.241453	-0.01287						
C	-4.13858	0.785495	-1.27427						
C	-2.96407	-0.16196	-1.52292						
O	-5.51571	-0.93529	-0.42636						
C	-5.76122	1.221518	0.648302						
C	-1.06673	-0.04807	0.223293						
C	0.000753	-0.58368	-0.75225						
C	0.135003	-2.11247	-0.85639						
C	1.420682	-2.50112	-1.54744						
C	2.646681	-2.45942	-1.00728						
C	3.893325	-2.79183	-1.78408						
O	-1.12349	1.378167	0.075684						
C	-0.65335	-0.37051	1.665589						
C	2.864582	-2.05195	0.424242						
O	3.415456	-0.70836	0.418505						
C	3.566141	-0.12243	1.610727						

O	3.273272	-0.65716	2.663611						
H	-2.4048	-1.67823	-0.06796						
C	-3.91184	-1.14285	1.966306						
C	4.176762	1.257302	1.515648						
C	4.046659	1.950976	0.168004						
C	2.616889	2.240551	-0.21386						
O	2.51605	2.648452	-1.47545						
O	1.663221	2.133189	0.541793						
C	1.201757	3.005626	-1.94121						
H	1.339149	-2.79525	-2.59267						
H	-3.25925	0.805314	1.345762						
H	-3.76892	1.794809	-1.07019						
H	-4.84518	0.839716	-2.10709						
H	-3.3004	-1.03948	-2.0807						
H	-2.18482	0.320762	-2.11423						
H	-5.94007	-1.31557	0.354142						
H	-6.57124	1.475387	-0.04096						
H	-6.20748	0.791769	1.551695						
H	-5.24621	2.14248	0.93361						
H	0.963043	-0.17035	-0.43406						
H	-0.18627	-0.17625	-1.75054						
H	-0.70965	-2.52055	-1.41734						
H	0.086142	-2.56539	0.137921						
H	3.664119	-3.05561	-2.81829						
H	4.581595	-1.94035	-1.79232						
H	4.426951	-3.63006	-1.32388						
H	-0.23606	1.726405	0.271199						
H	-0.71307	-1.43992	1.885007						
H	0.37713	-0.04342	1.841725						
H	-1.29431	0.158404	2.374282						
H	1.946314	-2.04499	1.010581						
H	3.584976	-2.70742	0.921158						
H	-4.74931	-0.80765	2.58793						
H	-3.06125	-1.30214	2.631718						
H	-4.17077	-2.11589	1.536644						
H	3.738067	1.855604	2.314265						
H	5.237393	1.14072	1.755556						
H	4.57071	2.910327	0.199442						
H	4.508635	1.372734	-0.63324						
H	0.776922	3.787085	-1.31066						
H	1.344446	3.370209	-2.95485						
H	0.549339	2.132283	-1.93915						

Table S 46. Energy analysis for 2*S*, 3*R*, 6*R*, 7*R*-6

Conf.	Steric Energy (kJ/mol)	Relative Energy (kJ/mol)	Distribution (%)
6a-1	-1233.289525	0.000368	16.24%
6a-2	-1233.288339	0.001554	4.63%
6a-3	-1233.287717	0.002176	2.39%
6a-4	-1233.288706	0.001187	6.82%
6a-5	-1233.287679	0.002214	2.30%
6a-6	-1233.289341	0.000552	13.36%
6a-7	-1233.289696	0.000197	19.46%
6a-8	-1233.289076	0.000817	10.09%
6a-9	-1233.28659	0.003303	0.73%
6a-10	-1233.289893	0	23.97%

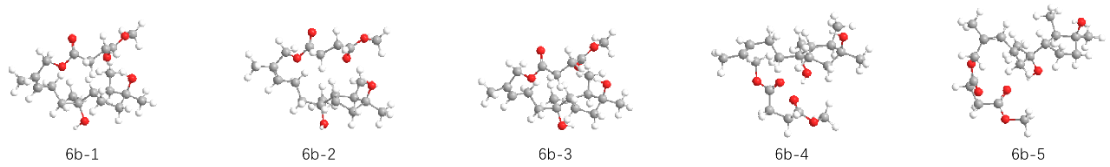

Figure S 102. Most stable conformers of 2*R*, 3*S*, 6*S*, 7*S*-6 at the CAM-B3LYP/DGDZVP level

Table S 47. Optimized Z-Matrixes of 2*R*, 3*S*, 6*S*, 7*S*-6 conformations in the methanol at CAM-B3LYP/DGDZVP level

	6b-1			6b-2			6b-3		
C	-1.13831	-1.41475	0.014058	1.123763	1.415133	0.02811	-1.1418	-1.41854	0.012079
C	-2.61575	-1.27402	0.485228	2.605122	1.283604	0.487196	-2.61601	-1.276	0.49688
C	-3.32661	-0.41107	-0.58796	3.319706	0.435272	-0.59522	-3.33354	-0.40252	-0.5648
C	-2.58395	-0.79663	-1.86974	2.562474	0.817669	-1.86996	-2.59934	-0.77898	-1.85441
C	-1.11673	-0.88359	-1.44587	1.098645	0.874074	-1.42885	-1.12938	-0.8736	-1.44164
C	-4.82627	-0.65405	-0.66273	4.815119	0.699677	-0.68084	-4.83409	-0.64241	-0.63055
C	-0.56811	-2.84414	0.148724	0.552868	2.850959	0.164104	-0.5628	-2.85354	0.123047
C	0.929779	-2.89728	-0.21698	-0.94421	2.909027	-0.20173	0.931394	-2.88891	-0.23918
C	1.8942	-2.26535	0.797523	-1.90658	2.250247	0.79722	1.885286	-2.27011	0.791496
C	3.295567	-2.2476	0.252409	-3.30667	2.232109	0.248656	3.291473	-2.25308	0.258704
C	4.107933	-1.19035	0.128127	-4.11607	1.172811	0.122546	4.106657	-1.19708	0.143058
C	5.469872	-1.28613	-0.50969	-5.47769	1.26516	-0.5163	5.474502	-1.29481	-0.48174
O	-1.28858	-3.64263	-0.80662	1.262811	3.746009	-0.7116	-1.17155	-3.71083	-0.86008
C	-0.7855	-3.42774	1.54844	0.769589	3.426858	1.561533	-0.78095	-3.46197	1.51169
C	3.706292	0.181561	0.604542	-3.71087	-0.19791	0.600002	3.702379	0.17546	0.615467
O	2.939159	0.817881	-0.45023	-2.93702	-0.83141	-0.45166	2.94152	0.810126	-0.44483
C	2.455223	2.031543	-0.17042	-2.44143	-2.03921	-0.16622	2.45769	2.025043	-0.17073
O	2.637385	2.601768	0.889198	-2.61604	-2.60525	0.896807	2.635211	2.598048	0.888181
C	1.686672	2.608823	-1.33587	-1.67109	-2.61618	-1.33066	1.695178	2.59971	-1.34144
C	0.743592	3.721292	-0.91066	-0.72396	-3.72479	-0.90441	0.754514	3.717143	-0.92406
C	-0.35227	3.227538	0.00019	0.374511	-3.22638	0.000657	-0.34404	3.231598	-0.01212

O	-1.08001	4.223652	0.495676	1.10423	-4.21963	0.498442	-1.06507	4.232694	0.482653
O	-0.56365	2.050266	0.248659	0.586786	-2.04776	0.242547	-0.56279	2.055791	0.237599
C	-2.1628	3.855973	1.371834	2.190843	-3.84726	1.367849	-2.14998	3.873189	1.35958
H	-0.5144	-0.76666	0.638166	0.506548	0.763979	0.655879	-0.51833	-0.77475	0.641283
C	-2.77547	-0.73464	1.905082	2.779745	0.738922	1.903126	-2.75988	-0.75023	1.923348
O	-3.17725	0.992087	-0.32985	3.19123	-0.97073	-0.34534	-3.1796	0.995784	-0.29362
H	-3.08473	-2.26219	0.428784	3.063481	2.277102	0.43247	-3.11073	-2.25521	0.450727
H	-2.92986	-1.77987	-2.20413	2.89659	1.806238	-2.20312	-2.78898	-0.05176	-2.64831
H	-2.76914	-0.07581	-2.67093	2.750577	0.104883	-2.67706	-2.95232	-1.75711	-2.19949
H	-0.54589	-1.52089	-2.12215	0.48964	1.467972	-2.11571	-0.56706	-1.51009	-2.12579
H	-0.65181	0.106489	-1.46904	0.661748	-0.1281	-1.43678	-0.66108	0.114578	-1.46118
H	-5.26996	-0.03377	-1.44694	5.261202	0.089071	-1.47117	-5.28137	-0.01442	-1.40625
H	-5.03667	-1.70161	-0.89124	5.009402	1.751083	-0.90579	-5.04956	-1.68733	-0.86651
H	-5.31014	-0.40118	0.285083	5.30919	0.449129	0.262182	-5.30998	-0.39613	0.322881
H	1.20407	-3.95286	-0.34129	-1.20919	3.967338	-0.30405	1.201845	-3.94071	-0.38452
H	1.076659	-2.42685	-1.19315	-1.09819	2.455194	-1.18685	1.080013	-2.39537	-1.20388
H	1.560637	-1.25725	1.052565	-1.56736	1.239806	1.034056	1.554212	-1.26328	1.054905
H	1.881476	-2.85056	1.723675	-1.89839	2.818541	1.733884	1.86359	-2.86549	1.710909
H	3.659444	-3.2099	-0.10977	-3.67187	3.194264	-0.11236	3.656934	-3.21547	-0.10149
H	5.676832	-2.29524	-0.87109	-5.68702	2.273835	-0.8775	5.683339	-2.30419	-0.8413
H	5.545238	-0.59617	-1.3571	-5.5506	0.575183	-1.3639	5.558973	-0.60484	-1.32827
H	6.255463	-1.00614	0.199871	-6.26302	0.982918	0.192641	6.2537	-1.01612	0.235351
H	-1.00884	-4.56117	-0.69508	1.055031	3.503837	-1.62421	-2.1172	-3.76929	-0.66795
H	-0.38472	-2.77386	2.326604	0.3832	2.75583	2.33135	-0.39676	-2.81252	2.301884
H	-1.849	-3.58076	1.742901	1.832059	3.591431	1.752716	-1.84326	-3.63154	1.709164
H	-0.28385	-4.39821	1.635473	0.257227	4.389065	1.652912	-0.27043	-4.42711	1.578372
H	3.095571	0.153598	1.507284	-3.10433	-0.16757	1.505432	3.086204	0.148754	1.514548
H	4.582844	0.799921	0.810887	-4.58581	-0.81983	0.802467	4.577641	0.794143	0.826153
H	2.421392	3.002195	-2.0442	-2.40496	-3.01345	-2.03771	2.433429	2.987369	-2.04923
H	1.15484	1.804398	-1.84498	-1.14286	-1.81099	-1.84231	1.162373	1.794748	-1.84869
H	1.277121	4.52804	-0.40555	-1.25407	-4.53098	-0.39487	1.289365	4.525482	-0.423
H	0.264675	4.163028	-1.78841	-0.24705	-4.16853	-1.78226	0.277841	4.154797	-1.8051
H	-2.59347	4.795641	1.707113	2.621504	-4.78508	1.708188	-2.57294	4.816078	1.695574
H	-2.90422	3.266839	0.831418	2.931198	-3.26352	0.82014	-2.89647	3.29025	0.81948
H	-1.78226	3.286065	2.21964	1.814563	-3.27052	2.212883	-1.77329	3.299886	2.206801
H	-3.83049	-0.59135	2.157848	3.837776	0.604499	2.147663	-3.8116	-0.60519	2.187308
H	-2.34924	-1.4219	2.639481	2.352256	1.419277	2.643114	-2.32833	-1.44684	2.645534
H	-2.27054	0.228965	2.020716	2.284476	-0.22974	2.017977	-2.2492	0.209784	2.040519
H	-2.2476	1.188455	-0.12461	2.268057	-1.1808	-0.12475	-2.24592	1.193767	-0.10778
	6b-4			6b-5					
C	1.663117	-1.29334	-0.06487	-2.35142	-0.36353	0.30562			
C	3.167205	-0.89669	-0.03752	-3.36481	-0.00404	-0.81982			
C	3.353315	0.151955	-1.16454	-4.60934	0.568981	-0.09703			

C	1.99631	0.857816	-1.19523	-3.99464	1.266902	1.117596			
C	0.98209	-0.27215	-1.01606	-2.91032	0.298448	1.595925			
C	4.523715	1.09319	-0.93089	-5.452	1.486657	-0.96767			
C	1.011053	-1.35978	1.342214	-0.89547	0.044559	-0.00187			
C	-0.49889	-1.66976	1.265862	0.065829	-0.34592	1.144978			
C	-0.91428	-2.86215	0.391234	0.015935	-1.78203	1.697024			
C	-2.41112	-3.06077	0.41471	0.330585	-2.87243	0.701109			
C	-3.31936	-2.34127	-0.25937	1.539571	-3.22528	0.243901			
C	-4.80165	-2.57464	-0.12755	1.726779	-4.28697	-0.80752			
O	1.167689	-0.11088	2.03047	-0.88492	1.477619	-0.09704			
C	1.715818	-2.38981	2.224204	-0.41678	-0.53236	-1.34083			
C	-2.92081	-1.22202	-1.18256	2.796113	-2.54621	0.714499			
O	-3.24047	0.024907	-0.51111	3.254927	-1.66049	-0.34441			
C	-2.74943	1.141925	-1.05425	3.735912	-0.46503	0.005224			
O	-2.07395	1.162314	-2.0666	3.831938	-0.07223	1.153702			
C	-3.16498	2.375757	-0.28607	4.149224	0.342317	-1.20452			
C	-2.05001	3.408874	-0.18635	4.256779	1.826224	-0.88371			
C	-0.91245	2.942179	0.685909	2.925486	2.401692	-0.46879			
O	-0.06554	3.922289	0.984374	3.042773	3.457935	0.328935			
O	-0.77804	1.793408	1.077031	1.850895	1.956454	-0.83989			
C	1.034713	3.581667	1.847688	1.811384	4.068284	0.757783			
H	1.579645	-2.29703	-0.49548	-2.34773	-1.45244	0.426971			
C	4.1353	-2.07337	-0.13988	-3.68933	-1.15321	-1.77149			
O	3.612916	-0.47032	-2.43527	-5.50361	-0.47477	0.330577			
H	3.35338	-0.36574	0.902474	-2.94404	0.825829	-1.39744			
H	1.857869	1.436962	-2.11179	-4.74771	1.497863	1.87594			
H	1.934593	1.546224	-0.34679	-3.53155	2.203948	0.793927			
H	0.030759	0.1088	-0.64355	-2.14267	0.815063	2.172279			
H	0.756346	-0.74159	-1.98013	-3.33543	-0.46144	2.261248			
H	4.589427	1.82443	-1.74131	-6.29465	1.88344	-0.39456			
H	4.398129	1.631236	0.011727	-4.85356	2.326028	-1.32919			
H	5.466848	0.54093	-0.89183	-5.85108	0.947594	-1.83148			
H	-0.8584	-1.82969	2.288526	1.084855	-0.12836	0.807173			
H	-1.02683	-0.78206	0.904528	-0.12204	0.335093	1.980015			
H	-0.57	-2.70869	-0.6351	0.714669	-1.82605	2.53811			
H	-0.42765	-3.77555	0.744866	-0.97119	-1.97184	2.127727			
H	-2.78097	-3.83989	1.079748	-0.52748	-3.40358	0.290326			
H	-5.02098	-3.38045	0.575522	0.773564	-4.72329	-1.11244			
H	-5.30679	-1.66829	0.221401	2.209951	-3.86727	-1.69611			
H	-5.24141	-2.83293	-1.09669	2.373841	-5.09107	-0.44129			
H	0.544992	0.536223	1.656852	0.019659	1.743127	-0.33791			
H	1.743548	-3.37234	1.746485	-0.5534	-1.61411	-1.39432			
H	2.742621	-2.08115	2.432951	-0.96413	-0.07103	-2.16655			

H	1.193872	-2.48265	3.180529	0.647672	-0.32161	-1.48114			
H	-1.85831	-1.22575	-1.42267	2.648371	-1.96345	1.620718			
H	-3.48566	-1.25351	-2.11873	3.600254	-3.26635	0.886021			
H	-4.00375	2.815258	-0.83293	5.119486	-0.03608	-1.53754			
H	-3.52999	2.089717	0.699882	3.440515	0.158226	-2.01246			
H	-1.6395	3.634884	-1.17417	4.990461	2.017185	-0.10109			
H	-2.43245	4.348147	0.216508	4.581238	2.37218	-1.77395			
H	1.579918	4.508784	2.001109	2.105907	4.902819	1.387971			
H	0.661944	3.194581	2.796104	1.244057	4.421041	-0.10363			
H	1.677564	2.839649	1.373333	1.216982	3.352362	1.326477			
H	5.174142	-1.73007	-0.15977	-4.44477	-0.85719	-2.5058			
H	4.026128	-2.75074	0.70988	-2.80214	-1.475	-2.32085			
H	3.959521	-2.65325	-1.05029	-4.07608	-2.0199	-1.22791			
H	2.892598	-1.08398	-2.6326	-5.01489	-1.09039	0.893192			

Table S 48. Energy analysis for 2*R*, 3*S*, 6*S*, 7*S*-6

Conf.	Steric Energy (kJ/mol)	Relative Energy (kJ/mol)	Distribution (%)
6b-1	-1233.289125	0.001833	8.90%
6b-2	-1233.289276	0.001682	10.45%
6b-3	-1233.289713	0.001245	16.59%
6b-4	-1233.290958	0	61.98%
6b-5	-1233.28775	0.003208	2.08%

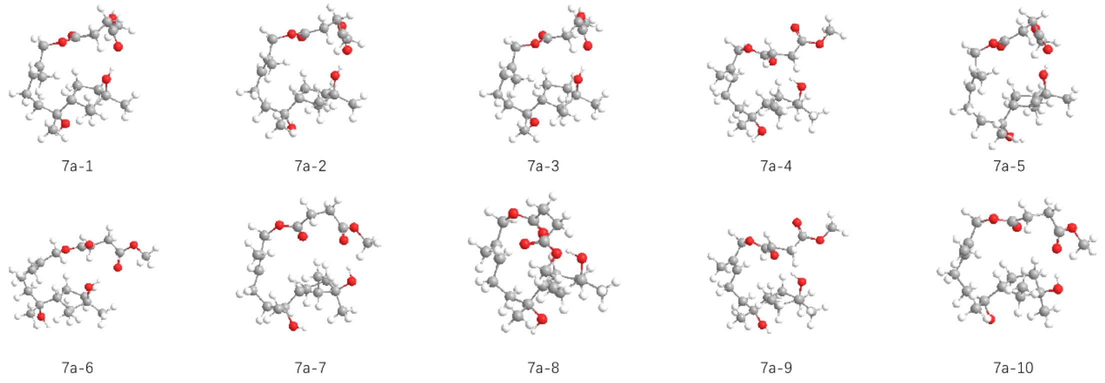

Figure S 103. Most stable conformers of 2*S*, 3*R*, 6*R*, 7*R*-7 calculated at MPW1PW91/6-31G+d, p level

Table S 49. Optimized Z-Matrixes of 2*S*, 3*R*, 6*R*, 7*R*-7 conformations in the Chloroform at MPW1PW91/6-31G+d, p level

	7a-1			7a-2			7a-3		
C	-1.80882	-0.70223	-0.09808	-1.82737	-0.69292	-0.08018	-1.80867	-0.7022	-0.09811
C	-3.19303	-0.67171	-0.77259	-3.20973	-0.68568	-0.77234	-3.19287	-0.67187	-0.77268
C	-3.3438	0.506806	-1.76	-3.37968	0.50514	-1.74213	-3.34369	0.506367	-1.76044
C	-3.25243	1.933787	-1.18944	-3.27779	1.92326	-1.15155	-3.25256	1.933512	-1.19034
C	-1.85223	2.448632	-0.99315	-1.87538	2.442404	-0.98368	-1.8525	2.448437	-0.99337
C	-1.26616	2.843388	0.144585	-1.26606	2.838265	0.14151	-1.26702	2.843428	0.144591
C	-1.86995	2.759316	1.521474	-1.84058	2.754576	1.530916	-1.87149	2.75984	1.521209

C	-4.33331	-0.64374	0.251887	-4.35655	-0.69917	0.237102	-4.3331	-0.64357	0.251872
O	-3.29088	-1.8895	-1.53327	-3.39545	-1.89344	-1.53308	-3.29071	-1.88985	-1.53306
C	0.109997	3.45027	0.090627	0.105651	3.452333	0.057654	0.109265	3.450044	0.091109
O	1.033747	2.745146	0.957925	1.045321	2.768996	0.925362	1.032648	2.744227	0.958393
C	1.90475	1.899785	0.395518	1.914186	1.918091	0.369268	1.90471	1.900089	0.395782
O	1.990221	1.691133	-0.79936	1.987964	1.687556	-0.82255	1.991388	1.692739	-0.79926
C	2.7792	1.242056	1.436621	2.801585	1.282726	1.413406	2.778378	1.241518	1.437024
C	4.09356	0.736508	0.850649	4.107484	0.761582	0.822856	4.093079	0.736131	0.851703
C	3.916686	-0.37827	-0.15115	3.917181	-0.38293	-0.14188	3.91684	-0.37788	-0.15105
O	4.700167	-0.2363	-1.21707	4.691312	-0.278	-1.2186	4.701618	-0.2355	-1.216
O	3.18375	-1.34125	0.007112	3.181665	-1.33732	0.053124	3.183308	-1.34065	0.005678
C	4.595192	-1.26312	-2.21702	4.576686	-1.33733	-2.18299	4.597625	-1.26179	-2.21661
C	-1.6239	-1.8298	0.95621	-1.62806	-1.83566	0.953483	-1.62379	-1.82975	0.956266
C	-0.19155	-2.3543	0.732484	-0.18562	-2.33335	0.732509	-0.19169	-2.35481	0.732221
C	-0.03332	-2.2386	-0.78373	-0.01643	-2.18675	-0.78078	-0.03373	-2.23901	-0.78404
C	-0.64612	-0.87768	-1.11078	-0.64425	-0.82507	-1.07725	-0.64588	-0.87772	-1.11067
C	-1.88314	-1.39277	2.396765	-1.90725	-1.43009	2.399238	-1.88256	-1.39252	2.396859
O	0.702294	-1.44285	1.386681	0.687826	-1.41973	1.408934	0.70266	-1.44384	1.386396
C	0.043223	-3.76451	1.264172	0.067433	-3.74977	1.237986	0.042564	-3.76523	1.263619
H	-1.69136	0.254215	0.414227	-1.734	0.257536	0.448335	-1.69133	0.25433	0.414072
H	-2.6127	0.390274	-2.56507	-2.65897	0.415284	-2.56323	-2.61252	0.389651	-2.56542
H	-4.32916	0.398607	-2.23079	-4.3683	0.388044	-2.19751	-4.32901	0.397899	-2.23126
H	-3.84319	2.017497	-0.27516	-3.84446	1.987709	-0.22066	-3.84388	2.017567	-0.27645
H	-3.73325	2.600396	-1.91443	-3.78157	2.598765	-1.85229	-3.73295	2.599892	-1.91584
H	-1.26805	2.522265	-1.91083	-1.31111	2.519971	-1.91361	-1.26776	2.52184	-1.91071
H	-2.87489	2.336926	1.519867	-2.84624	2.334505	1.550312	-2.87641	2.337361	1.519273
H	-1.91672	3.749824	1.986571	-1.87553	3.745215	1.996753	-1.91861	3.750545	1.985842
H	-1.24566	2.131879	2.165265	-1.20385	2.126223	2.161517	-1.24751	2.132705	2.16558
H	-4.22674	0.180304	0.961882	-4.26545	0.115881	0.958531	-4.22639	0.180657	0.961628
H	-5.29892	-0.5269	-0.25332	-5.31468	-0.59921	-0.28057	-5.29875	-0.52685	-0.25327
H	-4.36139	-1.57741	0.817052	-4.37013	-1.64147	0.788142	-4.36112	-1.57709	0.817309
H	-4.17116	-1.92207	-1.93074	-2.7233	-1.91534	-2.22735	-4.17098	-1.92256	-1.93053
H	0.507411	3.467288	-0.92325	0.489384	3.453868	-0.96165	0.506968	3.467272	-0.92264
H	0.096503	4.466535	0.491945	0.0908	4.475415	0.441089	0.095983	4.466157	0.492815
H	2.197884	0.427295	1.877327	2.225275	0.479177	1.880643	2.196616	0.426661	1.876977
H	2.976429	1.95555	2.23794	3.009568	2.014032	2.195706	2.975213	1.95455	2.238864
H	4.655634	1.548672	0.389969	4.661696	1.559936	0.329773	4.655681	1.548513	0.392055
H	4.705557	0.328512	1.660065	4.732356	0.378261	1.63452	4.704348	0.327421	1.661316
H	5.282712	-0.97035	-3.00632	5.257528	-1.07175	-2.98751	5.286748	-0.96917	-3.00457
H	4.879634	-2.22964	-1.80033	4.863973	-2.28976	-1.73685	4.880548	-2.22874	-1.7999
H	3.574363	-1.31128	-2.59676	3.55256	-1.39698	-2.55225	3.577422	-1.30895	-2.59818
H	-2.29697	-2.6577	0.704891	-2.28566	-2.66956	0.680136	-2.29724	-2.65742	0.70521
H	-0.61128	-3.03585	-1.26289	-0.57264	-2.98978	-1.27802	-0.61225	-3.03587	-1.26315

H	1.010079	-2.33615	-1.09977	1.030331	-2.26087	-1.09031	1.009534	-2.33702	-1.10038
H	-0.97878	-0.83565	-2.1489	-0.93521	-0.73126	-2.12805	-0.9782	-0.83492	-2.14886
H	0.091586	-0.08405	-0.97245	0.081847	-0.03044	-0.89718	0.0922	-0.08453	-0.97169
H	-2.91771	-1.07215	2.538996	-2.94852	-1.13032	2.536684	-2.9171	-1.07193	2.539356
H	-1.22949	-0.55978	2.666526	-1.2704	-0.59132	2.690273	-1.22884	-0.55949	2.666326
H	-1.69305	-2.20994	3.09989	-1.70949	-2.25703	3.088524	-1.69222	-2.20958	3.100045
H	1.570609	-1.51983	0.954219	1.568004	-1.4964	1.000862	1.570659	-1.52051	0.953248
H	-0.64187	-4.48093	0.801219	-0.60285	-4.46771	0.756214	-0.64277	-4.48129	0.800476
H	-0.0977	-3.79748	2.348189	-0.08189	-3.80536	2.319725	-0.09839	-3.79843	2.347627
H	1.068274	-4.08273	1.048385	1.099023	-4.04782	1.024717	1.06751	-4.08376	1.047781
	7a-4			7a-5			7a-6		
C	-1.78707	-0.85808	-0.17102	-1.81542	-0.706	-0.10425	-1.90492	-0.86938	-0.2196
C	-3.31472	-0.69457	-0.0377	-3.20314	-0.6718	-0.78536	-3.4174	-0.7916	-0.53338
C	-3.89812	0.316662	-1.04977	-3.34789	0.509252	-1.76248	-3.75957	0.428143	-1.41461
C	-3.61133	1.810607	-0.82712	-3.24793	1.927859	-1.17617	-3.65197	1.828397	-0.78024
C	-2.18497	2.220286	-1.06398	-1.84645	2.443383	-0.98953	-2.23396	2.227747	-0.48132
C	-1.37303	2.906735	-0.2508	-1.2507	2.836575	0.143776	-1.69693	2.538088	0.702228
C	-1.70067	3.359884	1.147765	-1.84125	2.747329	1.52614	-2.44535	2.642578	2.00556
C	-3.72412	-0.29889	1.38504	-4.34757	-0.65033	0.234163	-4.28436	-0.77477	0.729093
O	-3.87176	-1.98517	-0.34408	-3.37391	-1.82129	-1.63522	-3.81951	-1.90978	-1.34779
C	-0.00275	3.297689	-0.74258	0.120379	3.45363	0.077171	-0.2076	2.687218	0.883079
O	1.031941	2.971239	0.218795	1.05878	2.764715	0.942013	0.486236	2.270713	-0.30069
C	1.46568	1.711245	0.250533	1.914055	1.902922	0.381196	1.765613	1.910614	-0.16373
O	1.087311	0.849999	-0.52475	1.969941	1.662272	-0.80941	2.404763	2.028488	0.862575
C	2.441709	1.498444	1.382857	2.814643	1.272335	1.417281	2.305556	1.338872	-1.45387
C	3.261751	0.226731	1.249604	4.113007	0.750058	0.810908	3.803577	1.087766	-1.40427
C	4.164152	0.248237	0.039086	3.908559	-0.38822	-0.15837	4.194892	-0.03142	-0.47057
O	4.768051	-0.93154	-0.13905	4.667898	-0.27744	-1.24463	5.459484	0.076029	-0.06966
O	4.34292	1.210749	-0.68226	3.17457	-1.34316	0.040872	3.474456	-0.96243	-0.1497
C	5.649121	-1.02463	-1.26801	4.533718	-1.32711	-2.21725	5.942027	-0.96812	0.793179
C	-1.15158	-1.82545	0.869926	-1.62546	-1.81896	0.96607	-1.40005	-2.0067	0.706348
C	-0.1137	-2.66582	0.082161	-0.1847	-2.32863	0.756896	0.075609	-2.16936	0.280015
C	-0.76766	-2.78648	-1.29571	-0.02466	-2.24007	-0.76119	-0.00827	-2.06785	-1.24334
C	-1.34668	-1.39546	-1.56024	-0.65731	-0.89543	-1.11859	-1.0354	-0.95927	-1.50217
C	-0.56286	-1.13464	2.098264	-1.9033	-1.3702	2.399422	-1.54764	-1.74653	2.205834
O	1.148983	-1.99752	-0.02517	0.687892	-1.389	1.395499	0.7716	-1.03383	0.804823
C	0.192125	-4.00917	0.726214	0.07058	-3.72397	1.317741	0.737967	-3.45579	0.761846
H	-1.35624	0.134685	-0.02497	-1.68978	0.251398	0.404319	-1.6492	0.054779	0.295994
H	-3.58815	0.034595	-2.05986	-2.61473	0.397462	-2.56663	-3.14438	0.39689	-2.31904
H	-4.98833	0.193157	-1.02521	-4.33009	0.397465	-2.233	-4.794	0.284958	-1.74458
H	-3.95934	2.11596	0.1613	-3.8295	2.004308	-0.25534	-4.2876	1.888533	0.105889
H	-4.23209	2.358656	-1.54609	-3.73598	2.601863	-1.88934	-4.07151	2.53429	-1.50642
H	-1.78478	1.917084	-2.03226	-1.27095	2.522157	-1.91232	-1.55847	2.150168	-1.33044

H	-2.74613	3.200743	1.410755	-2.84082	2.31233	1.533593	-3.52021	2.755834	1.865717
H	-1.47804	4.424412	1.27251	-1.89666	3.737915	1.990203	-2.09456	3.500173	2.588794
H	-1.08588	2.820591	1.87606	-1.20349	2.128973	2.165535	-2.27191	1.748853	2.616885
H	-3.18526	0.588474	1.727758	-4.24135	0.167069	0.951965	-3.93682	-0.03096	1.451017
H	-4.7968	-0.07717	1.427615	-5.30541	-0.5378	-0.28144	-5.32238	-0.54911	0.467002
H	-3.52193	-1.11374	2.083055	-4.38318	-1.58341	0.804123	-4.2731	-1.75055	1.222213
H	-4.82891	-1.93481	-0.22102	-3.34998	-2.61085	-1.07885	-3.6379	-2.71776	-0.84991
H	0.234244	2.833583	-1.69998	0.510278	3.468265	-0.93972	0.073237	3.720553	1.112391
H	0.079131	4.382251	-0.83542	0.100753	4.472017	0.472668	0.118964	2.062388	1.719601
H	1.850548	1.46186	2.303677	2.245728	0.470528	1.896088	2.062847	2.02762	-2.26587
H	3.078921	2.380973	1.456381	3.033144	2.007244	2.193319	1.757701	0.414733	-1.6565
H	3.897462	0.105275	2.131246	4.663948	1.549544	0.315985	4.155454	0.795206	-2.39833
H	2.63149	-0.66441	1.19652	4.745318	0.360934	1.613979	4.352189	1.987778	-1.1256
H	6.035467	-2.04067	-1.25243	5.20249	-1.05612	-3.03004	6.970298	-0.70144	1.022771
H	5.10038	-0.84094	-2.19219	4.824625	-2.28481	-1.78497	5.344278	-1.00873	1.703591
H	6.463881	-0.30539	-1.17615	3.5035	-1.37915	-2.57026	5.901333	-1.93018	0.281827
H	-1.92349	-2.53101	1.196817	-2.28081	-2.6732	0.741648	-1.89672	-2.95697	0.458808
H	-1.58007	-3.51853	-1.24609	-0.58356	-3.05996	-1.22722	-0.36462	-3.02172	-1.6478
H	-0.04986	-3.11647	-2.05144	1.020767	-2.32866	-1.0719	0.973824	-1.87416	-1.68746
H	-2.16658	-1.44257	-2.27768	-1.0029	-0.88795	-2.15338	-1.63892	-1.1921	-2.38142
H	-0.58617	-0.73388	-1.98682	0.072221	-0.09021	-1.00927	-0.54599	0.001033	-1.68404
H	-1.33487	-0.62967	2.683369	-2.94246	-1.06051	2.529039	-2.58936	-1.75681	2.527609
H	0.175875	-0.38361	1.805805	-1.26128	-0.52659	2.66254	-1.11881	-0.77601	2.467237
H	-0.06278	-1.85167	2.756451	-1.70813	-2.17716	3.112458	-1.02219	-2.50937	2.78789
H	1.016502	-1.08448	-0.33408	1.563475	-1.46877	0.977621	1.680988	-1.05421	0.460684
H	-0.71719	-4.60765	0.822088	-0.6008	-4.4621	0.86854	0.183846	-4.3364	0.423759
H	0.627756	-3.87453	1.720286	-0.07153	-3.73733	2.401858	0.799347	-3.47876	1.852825
H	0.909971	-4.5632	0.114906	1.100867	-4.02945	1.109747	1.757215	-3.52385	0.367915
	7a-7			7a-8			7a-9		
C	-1.53679	-0.88598	-0.01507	-1.80547	-0.41738	-0.05495	-1.79711	-0.86028	-0.17444
C	-3.07579	-0.98786	0.044795	-2.95631	0.012163	0.8752	-3.33139	-0.69095	-0.0503
C	-3.76865	-0.08967	-0.99463	-3.49613	1.421696	0.548953	-3.89897	0.324674	-1.05936
C	-3.74093	1.429092	-0.76114	-2.5701	2.624191	0.794562	-3.60675	1.813663	-0.8217
C	-2.42122	2.103971	-1.01371	-1.48027	2.792376	-0.22799	-2.18047	2.224116	-1.05819
C	-1.6754	2.811844	-0.15576	-0.17493	3.009981	-0.02843	-1.36586	2.905142	-0.24321
C	-1.96014	3.002037	1.311035	0.524097	3.051119	1.303398	-1.68901	3.348376	1.159553
C	-3.59124	-0.67973	1.454996	-2.55102	-0.05744	2.351938	-3.748	-0.30032	1.371379
O	-3.50854	-2.30858	-0.33014	-4.02243	-0.92526	0.639622	-3.99906	-1.91429	-0.41152
C	-0.4609	3.539325	-0.66954	0.697314	3.293143	-1.22673	0.001511	3.301615	-0.73843
O	0.6949	3.351808	0.182862	1.983033	2.624216	-1.18668	1.041367	2.973966	0.217189
C	1.555421	2.376395	-0.131	1.990885	1.308863	-1.38645	1.470581	1.713043	0.249604
O	1.42623	1.624028	-1.07635	0.977909	0.657154	-1.57434	1.084596	0.851115	-0.5217
C	2.715827	2.345916	0.837399	3.388438	0.738253	-1.42312	2.450751	1.499525	1.378271

C	3.922666	1.609187	0.273281	3.490808	-0.63447	-0.77281	3.266668	0.225187	1.245475
C	3.71561	0.117982	0.18698	3.331137	-0.56816	0.726273	4.165841	0.241443	0.032427
O	4.377427	-0.42721	-0.82936	3.521264	-1.76264	1.29368	4.768246	-0.93958	-0.14285
O	3.059586	-0.52402	0.990709	3.05429	0.437804	1.352	4.343238	1.201029	-0.693
C	4.314368	-1.86032	-0.94387	3.334173	-1.82006	2.715751	5.646698	-1.03734	-1.27358
C	-0.77164	-1.81991	0.968642	-1.18952	-1.80532	0.28492	-1.15396	-1.81622	0.874439
C	0.247321	-2.61716	0.092378	-0.94117	-2.49993	-1.07847	-0.11385	-2.66025	0.0912
C	0.450472	-1.71017	-1.12899	-2.10333	-1.97843	-1.92774	-0.77083	-2.79347	-1.2843
C	-0.93833	-1.14578	-1.41217	-2.21889	-0.4997	-1.54973	-1.35633	-1.40707	-1.55872
C	-0.11365	-1.01575	2.090057	0.059271	-1.73627	1.159648	-0.57101	-1.11217	2.097811
O	1.462957	-2.93084	0.762675	0.312849	-2.11968	-1.65747	1.142946	-1.98677	-0.02211
C	-0.33842	-3.96657	-0.31578	-0.8988	-4.01756	-0.98282	0.199888	-3.99664	0.746174
H	-1.28473	0.141913	0.262003	-1.02355	0.33875	0.0498	-1.36203	0.131193	-0.03097
H	-3.36559	-0.31856	-1.98603	-3.84495	1.439188	-0.48785	-3.57614	0.051161	-2.06801
H	-4.81764	-0.40289	-1.01255	-4.39046	1.56573	1.168862	-4.98548	0.191362	-1.04321
H	-4.12495	1.661115	0.233973	-2.17028	2.593794	1.810011	-3.95367	2.113024	0.168883
H	-4.45931	1.864078	-1.46575	-3.19999	3.520941	0.748667	-4.22704	2.368711	-1.53564
H	-2.05995	2.018131	-2.03905	-1.82852	2.745761	-1.26098	-1.7834	1.928595	-2.03021
H	-2.96297	2.680533	1.591987	-0.16763	2.965598	2.141448	-2.7332	3.185231	1.425073
H	-1.84933	4.053167	1.59547	1.082823	3.986062	1.418211	-1.4681	4.412496	1.29072
H	-1.24377	2.432967	1.913135	1.250481	2.235703	1.379148	-1.0705	2.805541	1.882147
H	-3.21947	0.281902	1.817602	-1.6303	0.498754	2.546151	-3.20246	0.579575	1.723001
H	-4.68489	-0.65792	1.463952	-3.33931	0.364571	2.98587	-4.81912	-0.08058	1.401136
H	-3.26448	-1.4422	2.168345	-2.39499	-1.09419	2.656567	-3.5551	-1.11314	2.077564
H	-3.15516	-2.93438	0.316123	-4.74624	-0.71203	1.243302	-3.71555	-2.60546	0.201353
H	-0.21139	3.244728	-1.68867	0.191737	3.033754	-2.15831	0.236031	2.842304	-1.69878
H	-0.62714	4.618484	-0.63513	0.978508	4.347642	-1.25215	0.080551	4.386731	-0.82684
H	2.368935	1.866125	1.75708	4.081325	1.446291	-0.96981	1.8632	1.467557	2.30162
H	2.980802	3.371774	1.097045	3.650906	0.651097	-2.48163	3.090893	2.380302	1.446959
H	4.206706	1.993164	-0.70647	2.732018	-1.32114	-1.15937	3.904395	0.103743	2.125639
H	4.776905	1.759851	0.940156	4.459825	-1.08755	-0.99423	2.633716	-0.66418	1.195905
H	4.957142	-2.11029	-1.784	3.493152	-2.86037	2.988464	6.033139	-2.05328	-1.25469
H	4.678423	-2.32931	-0.02924	4.057156	-1.17814	3.220476	5.095705	-0.85745	-2.19716
H	3.290888	-2.18112	-1.13698	2.321573	-1.50846	2.975063	6.461595	-0.31768	-1.18664
H	-1.44405	-2.54246	1.447643	-1.95285	-2.40421	0.794311	-1.90594	-2.53445	1.230335
H	0.885686	-2.24858	-1.97675	-3.02071	-2.50378	-1.64247	-1.57811	-3.53263	-1.23117
H	1.126015	-0.88878	-0.86627	-1.9264	-2.14838	-2.99327	-0.05366	-3.1276	-2.03829
H	-1.54645	-1.87322	-1.95862	-3.22735	-0.12609	-1.73185	-2.17964	-1.46456	-2.2715
H	-0.89423	-0.23275	-2.01023	-1.53698	0.108615	-2.15155	-0.59973	-0.74664	-1.99333
H	-0.87441	-0.46094	2.64795	-0.17629	-1.40093	2.172252	-1.34774	-0.60871	2.677555
H	0.600725	-0.289	1.688814	0.777984	-1.02837	0.740132	0.160393	-0.35817	1.796478
H	0.421102	-1.66252	2.79018	0.552513	-2.71019	1.234672	-0.0649	-1.82024	2.760597
H	1.966816	-2.11045	0.899584	0.406527	-1.15155	-1.64241	1.008892	-1.07531	-0.33598

H	-1.29065	-3.84702	-0.83827	-1.8331	-4.40617	-0.57009	-0.70438	-4.60217	0.847764
H	-0.50616	-4.58573	0.570583	-0.07231	-4.34385	-0.34494	0.635776	-3.85112	1.738498
H	0.354704	-4.49256	-0.97832	-0.75072	-4.45203	-1.97549	0.920528	-4.5504	0.138384
	7a-10								
C	-1.54414	-0.89019	-0.01864						
C	-3.08369	-0.97341	0.055382						
C	-3.78022	-0.05956	-0.97477						
C	-3.73588	1.459496	-0.73989						
C	-2.41322	2.123003	-1.00481						
C	-1.65328	2.821494	-0.15167						
C	-1.92103	3.007722	1.31868						
C	-3.58136	-0.66971	1.467128						
O	-3.52398	-2.31983	-0.20055						
C	-0.44115	3.545562	-0.67635						
O	0.719234	3.360947	0.169118						
C	1.572797	2.378097	-0.1409						
O	1.435339	1.620139	-1.08069						
C	2.735407	2.347836	0.824491						
C	3.935399	1.597314	0.264628						
C	3.716226	0.107162	0.191021						
O	4.382511	-0.45268	-0.81481						
O	3.048038	-0.52174	0.994701						
C	4.309396	-1.88634	-0.91621						
C	-0.79603	-1.83875	0.960094						
C	0.233577	-2.62542	0.086034						
C	0.43992	-1.71422	-1.13229						
C	-0.94763	-1.14987	-1.41872						
C	-0.14707	-1.05685	2.102048						
O	1.452528	-2.9321	0.755221						
C	-0.34119	-3.97935	-0.32139						
H	-1.27527	0.136125	0.250568						
H	-3.39066	-0.27988	-1.97637						
H	-4.83296	-0.36159	-0.98105						
H	-4.1054	1.688115	0.261334						
H	-4.45938	1.903404	-1.4336						
H	-2.06404	2.039495	-2.03461						
H	-2.92013	2.684891	1.610863						
H	-1.80697	4.058166	1.604338						
H	-1.19751	2.437237	1.910601						
H	-3.17927	0.277786	1.832392						
H	-4.67384	-0.61579	1.484019						
H	-3.27114	-1.45632	2.158199						
H	-3.36953	-2.51327	-1.13442						

H	-0.19875	3.246896	-1.69608						
H	-0.60708	4.624851	-0.64532						
H	2.386529	1.878738	1.748919						
H	3.008159	3.374055	1.07445						
H	4.220889	1.970769	-0.71881						
H	4.792163	1.74691	0.928491						
H	4.956335	-2.14911	-1.74925						
H	4.663342	-2.3493	0.005427						
H	3.284877	-2.2016	-1.11284						
H	-1.49558	-2.55948	1.392762						
H	0.881672	-2.24551	-1.98115						
H	1.109384	-0.89002	-0.86275						
H	-1.53538	-1.88182	-1.98637						
H	-0.9069	-0.23856	-2.01952						
H	-0.90974	-0.50647	2.661566						
H	0.5782	-0.32722	1.724847						
H	0.374043	-1.71917	2.798174						
H	1.945961	-2.10765	0.903637						
H	-1.32122	-3.87203	-0.79098						
H	-0.46412	-4.60931	0.564051						
H	0.332934	-4.48541	-1.01864						

Table S 50. Energy analysis for 2*S*, 3*R*, 6*R*, 7*R*-7 in the Chloroform at MPW1PW91/6-31G+d, p level

Conf.	Steric Energy (kJ/mol)	Relative Energy (kJ/mol)	Distribution (%)
7a-1	-1233.286572	0.000813	11.01%
7a-2	-1233.28659	0.000795	11.22%
7a-3	-1233.286604	0.000781	11.39%
7a-4	-1233.286334	0.001051	8.56%
7a-5	-1233.286148	0.001237	7.03%
7a-6	-1233.285415	0.00197	3.23%
7a-7	-1233.285773	0.001612	4.73%
7a-8	-1233.287385	0	26.04%
7a-9	-1233.286621	0.000764	11.60%
7a-10	-1233.285864	0.001521	5.20%

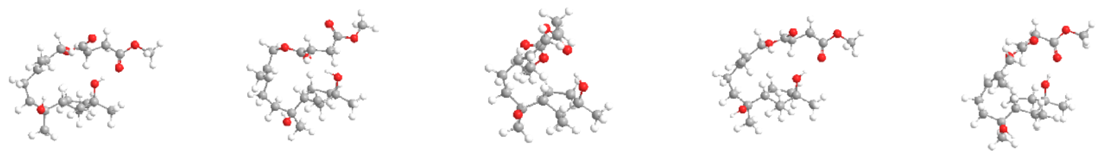

7b-1 7b-2 7b-3 7b-4 7b-5

Figure S 104. Most stable conformers of 2*S*, 3*R*, 6*R*, 7*S*-7 calculated at MPW1PW91/6-31G+d, p level

Table S 51. Optimized Z-Matrixes of 2*S*, 3*R*, 6*R*, 7*S*-7 conformations in the Chloroform at MPW1PW91/6-31G+d, p level

	7b-1			7b-2			7b-3		

C	-1.96917	-1.05157	-0.23355	1.997719	0.885501	-0.20316	1.687651	-0.66601	0.355574
C	-3.49657	-0.76211	-0.19619	3.509867	0.514725	-0.14667	2.262525	-0.31372	1.753974
C	-3.89789	0.375762	-1.18743	3.876705	-0.66937	-1.0925	1.212866	0.410906	2.653935
C	-3.62332	1.829877	-0.72214	3.388208	-2.08344	-0.69318	0.922144	1.903575	2.328589
C	-2.16655	2.183293	-0.54634	1.922628	-2.3576	-0.9222	0.122686	2.151248	1.072539
C	-1.59794	2.858333	0.469441	1.100347	-3.13712	-0.19129	0.511089	2.817509	-0.02935
C	-2.32537	3.452882	1.656103	1.481397	-3.88235	1.067195	1.857791	3.475483	-0.24182
C	-4.36274	-1.99992	-0.45728	4.443611	1.693924	-0.44847	2.833231	-1.52222	2.506928
O	-3.8922	-0.34474	1.168021	3.872205	0.131559	1.235709	3.432914	0.583386	1.606669
C	-0.10709	3.108854	0.528064	-0.32171	-3.3369	-0.65021	-0.35912	2.925276	-1.26468
O	0.58315	2.410351	-0.57015	-1.30369	-2.82859	0.364248	-1.773	2.561118	-1.0301
C	1.83828	1.907225	-0.33896	-1.61227	-1.50105	0.340509	-2.21655	1.30945	-1.33079
O	2.474966	2.099161	0.703111	-1.12018	-0.71454	-0.48757	-1.50215	0.405156	-1.79095
C	2.320215	1.096241	-1.51632	-2.55191	-1.12695	1.463058	-3.70266	1.184727	-1.08463
C	3.830565	0.841078	-1.46513	-3.37001	0.136634	1.178964	-4.12745	-0.25338	-0.78052
C	4.23886	-0.11799	-0.37286	-4.32336	-0.06583	0.029242	-3.61938	-0.72119	0.562068
O	5.505997	0.125104	0.065033	-5.15752	1.011688	-0.12862	-4.12849	-1.94751	0.890705
O	3.562782	-1.06683	0.057761	-4.37361	-1.0658	-0.6987	-2.83529	-0.09923	1.292165
C	6.031756	-0.75037	1.127557	-6.11125	0.95475	-1.24671	-3.68143	-2.54098	2.163319
C	-1.40522	-2.14825	0.722887	1.528645	1.990164	0.807405	2.611731	-1.34615	-0.69851
C	0.001253	-2.4085	0.112871	0.368375	2.735798	0.071751	1.578994	-1.75326	-1.79722
C	-0.30083	-2.49347	-1.40038	0.855066	2.75533	-1.39231	0.411339	-2.34196	-0.96849
C	-1.42412	-1.44953	-1.64843	1.514392	1.373184	-1.61654	0.429111	-1.591	0.395089
C	-1.38271	-1.79297	2.214256	1.146039	1.472488	2.200025	3.778242	-0.50853	-1.23451
O	0.775052	-1.18773	0.373187	-0.88968	1.990644	0.171045	1.14724	-0.53633	-2.50559
C	0.737	-3.62684	0.670864	0.05088	4.115729	0.638467	2.123017	-2.70139	-2.86295
H	-1.4619	-0.12381	0.054555	1.447206	-0.03391	0.038694	1.373201	0.280807	-0.09978
H	-3.41341	0.19883	-2.15633	3.534615	-0.43844	-2.11005	0.274441	-0.15776	2.651784
H	-4.97922	0.297087	-1.35893	4.972142	-0.70818	-1.13211	1.599662	0.382792	3.680982
H	-4.19491	2.007507	0.19446	3.681379	-2.28145	0.344404	1.876094	2.439316	2.322411
H	-4.05878	2.493751	-1.48658	3.968209	-2.79869	-1.2995	0.350774	2.304747	3.1794
H	-1.5067	1.844706	-1.34174	1.503929	-1.88492	-1.81145	-0.87727	1.720662	1.086418
H	-3.40114	3.269352	1.620904	2.475249	-3.61311	1.432656	2.48536	3.439134	0.651081
H	-2.18104	4.542116	1.699632	1.476715	-4.96836	0.894714	1.740847	4.533553	-0.51488
H	-1.94142	3.047881	2.603316	0.755017	-3.68739	1.865345	2.408217	2.996669	-1.06554
H	-4.24914	-2.3556	-1.48698	4.3436	2.02316	-1.48801	2.039982	-2.20793	2.823001
H	-5.41394	-1.74321	-0.29402	5.479356	1.38335	-0.28113	3.36113	-1.16977	3.398367
H	-4.10559	-2.8117	0.227788	4.240827	2.540278	0.211556	3.547878	-2.0696	1.886675
H	-3.2284	0.288749	1.510171	3.201161	-0.48876	1.586105	3.278742	1.198265	0.861866
H	0.124023	4.178096	0.4317	-0.52288	-2.83135	-1.59566	-0.4156	3.958296	-1.62012
H	0.319569	2.762147	1.475879	-0.59281	-4.39291	-0.7177	0.03549	2.291484	-2.06821
H	2.045154	1.612547	-2.44104	-1.93924	-0.96824	2.361508	-4.21681	1.534764	-1.99005
H	1.76099	0.152848	-1.49144	-3.19671	-1.98667	1.662168	-3.97608	1.865158	-0.27515

H	4.156127	0.4025	-2.4189	-3.94985	0.418958	2.06527	-5.21813	-0.35181	-0.79556
H	4.389375	1.770093	-1.33103	-2.71636	0.990748	0.954474	-3.73773	-0.93571	-1.54548
H	7.035462	-0.37749	1.318469	-6.65897	1.893224	-1.19164	-4.22087	-3.48295	2.232579
H	5.403408	-0.66917	2.016243	-5.57465	0.866918	-2.19377	-2.60225	-2.70495	2.138986
H	6.051697	-1.78698	0.785341	-6.78039	0.099542	-1.1293	-3.93165	-1.87887	2.994516
H	-1.97791	-3.07864	0.585153	2.336189	2.725135	0.926762	3.008544	-2.28346	-0.27647
H	-0.64934	-3.50479	-1.64827	1.59725	3.555557	-1.51469	0.577296	-3.41623	-0.82224
H	0.602019	-2.30524	-1.99184	0.027643	2.958828	-2.07951	-0.53846	-2.23361	-1.50399
H	-2.21228	-1.87438	-2.28044	2.333956	1.438802	-2.33915	0.503452	-2.30792	1.221723
H	-1.03864	-0.5682	-2.17134	0.788852	0.661074	-2.0254	-0.48258	-1.01017	0.55384
H	-0.78415	-0.89019	2.375132	0.35994	0.712762	2.128321	3.397646	0.406288	-1.70309
H	-0.93035	-2.59978	2.804065	0.759826	2.282948	2.828973	4.334122	-1.05991	-2.00231
H	-2.3967	-1.62884	2.586534	2.017292	1.045341	2.704391	4.474662	-0.24754	-0.43403
H	1.745486	-1.3377	0.299933	-0.83876	1.098288	-0.2443	0.237326	-0.27092	-2.25007
H	0.143527	-4.53903	0.539813	0.935914	4.761327	0.617524	2.565706	-3.59599	-2.40837
H	1.694551	-3.76902	0.153908	-0.74381	4.585493	0.050067	1.317233	-3.0119	-3.5372
H	0.945058	-3.49912	1.737979	-0.30235	4.035334	1.67137	2.884874	-2.198	-3.46558
	7b-4			7b-5					
C	-1.99551	-0.93722	-0.1827	-1.97559	-0.93438	-0.20212			
C	-3.52788	-0.76425	-0.3357	-3.51628	-0.78173	-0.36065			
C	-3.88979	0.492766	-1.18151	-3.88571	0.46309	-1.2066			
C	-3.59678	1.871055	-0.53591	-3.61688	1.845735	-0.55892			
C	-2.1362	2.249352	-0.46779	-2.16179	2.242122	-0.47801			
C	-1.50326	2.870062	0.542754	-1.53594	2.854422	0.542615			
C	-2.1496	3.355491	1.821398	-2.19008	3.315529	1.826163			
C	-4.2296	-2.00345	-0.92224	-4.19982	-2.03136	-0.9496			
O	-4.04805	-0.56406	1.035393	-4.14376	-0.51205	0.953039			
C	-0.01637	3.144235	0.514521	-0.0518	3.146853	0.516923			
O	0.6365	2.413311	-0.58993	0.612538	2.42428	-0.58779			
C	1.885044	1.889871	-0.37253	1.856165	1.892378	-0.36497			
O	2.550401	2.093307	0.649486	2.511487	2.073433	0.667812			
C	2.328187	1.039357	-1.53792	2.309892	1.06278	-1.54184			
C	3.832506	0.749156	-1.50288	3.81779	0.789393	-1.5109			
C	4.226293	-0.20342	-0.39966	4.224512	-0.18268	-0.42983			
O	5.504867	0.00997	0.021183	5.494691	0.047272	0.006106			
O	3.526863	-1.12429	0.052842	3.543366	-1.13181	-0.00758			
C	6.01404	-0.86122	1.095186	6.017027	-0.84067	1.0598			
C	-1.48316	-2.08069	0.750205	-1.45719	-2.01381	0.805203			
C	-0.0557	-2.35701	0.196806	-0.0489	-2.37083	0.243509			
C	-0.28161	-2.33686	-1.32936	-0.30134	-2.43543	-1.27633			
C	-1.26158	-1.15931	-1.55411	-1.23678	-1.23555	-1.55584			
C	-1.51698	-1.75714	2.24872	-1.41439	-1.55978	2.273016			
O	0.746451	-1.1847	0.566359	0.788618	-1.20264	0.531244			

C	0.604729	-3.63286	0.71971	0.582055	-3.63187	0.835155			
H	-1.62124	-0.00748	0.251426	-1.60931	0.022669	0.173958			
H	-3.39381	0.423905	-2.15888	-3.37142	0.396066	-2.17486			
H	-4.97052	0.448751	-1.38803	-4.96158	0.402603	-1.41503			
H	-4.049	1.881319	0.460258	-4.08936	1.851157	0.426492			
H	-4.13348	2.623959	-1.13681	-4.15172	2.589604	-1.17286			
H	-1.53946	1.972391	-1.33404	-1.55907	1.986219	-1.34745			
H	-3.2337	3.23106	1.811747	-3.26622	3.135083	1.829406			
H	-1.94018	4.42191	1.989691	-2.03314	4.392413	1.986042			
H	-1.75599	2.811561	2.691328	-1.75914	2.798832	2.695207			
H	-3.8967	-2.21842	-1.94297	-3.89193	-2.22069	-1.98289			
H	-5.31488	-1.8392	-0.96215	-5.28502	-1.88799	-0.93739			
H	-4.04324	-2.8843	-0.30201	-3.9676	-2.92881	-0.3632			
H	-5.02697	-0.53244	1.002936	-4.10192	-1.31937	1.502773			
H	0.198782	4.210621	0.365036	0.152076	4.215181	0.365981			
H	0.459062	2.836425	1.451801	0.426854	2.8447	1.45436			
H	2.04839	1.537795	-2.47093	2.025105	1.571306	-2.46773			
H	1.752242	0.107966	-1.48113	1.745279	0.123667	-1.49927			
H	4.135631	0.287802	-2.45326	4.126462	0.352394	-2.47103			
H	4.415245	1.665962	-1.38886	4.389369	1.710441	-1.37689			
H	7.029992	-0.5149	1.270902	7.023874	-0.47653	1.25107			
H	5.396235	-0.74536	1.987429	5.391743	-0.76219	1.950899			
H	6.000316	-1.90408	0.772128	6.028703	-1.87456	0.709023			
H	-2.06659	-2.99643	0.566039	-2.06413	-2.93013	0.719066			
H	-0.73523	-3.28666	-1.64462	-0.79818	-3.38358	-1.52488			
H	0.662601	-2.22767	-1.8759	0.636864	-2.3982	-1.84237			
H	-1.95419	-1.37774	-2.3742	-1.92806	-1.45622	-2.3756			
H	-0.71764	-0.25106	-1.83099	-0.65737	-0.3574	-1.85702			
H	-0.86125	-0.9066	2.456999	-0.66695	-0.76939	2.381444			
H	-1.16497	-2.6094	2.844291	-1.13626	-2.38674	2.938026			
H	-2.53333	-1.50757	2.558196	-2.37724	-1.16337	2.606166			
H	1.708028	-1.34107	0.426095	1.743366	-1.37657	0.364638			
H	-0.00496	-4.51511	0.491578	-0.05525	-4.50832	0.668435			
H	1.590459	-3.77409	0.258188	1.556088	-3.82952	0.369925			
H	0.743429	-3.57767	1.804076	0.739898	-3.51593	1.912232			

Table S 52. Energy analysis for 2*S*, 3*R*, 6*R*, 7*S*-7 in the Chloroform at MPW1PW91/6-31G+d, p level

Conf.	Steric Energy (kJ/mol)	Relative Energy (kJ/mol)	Distribution (%)
7b-1	-1233.285814	0.000765	20.62%
7b-2	-1233.285871	0.000708	21.90%
7b-3	-1233.286579	0	46.34%
7b-4	-1233.284414	0.002165	4.68%
7b-5	-1233.284718	0.001861	6.46%

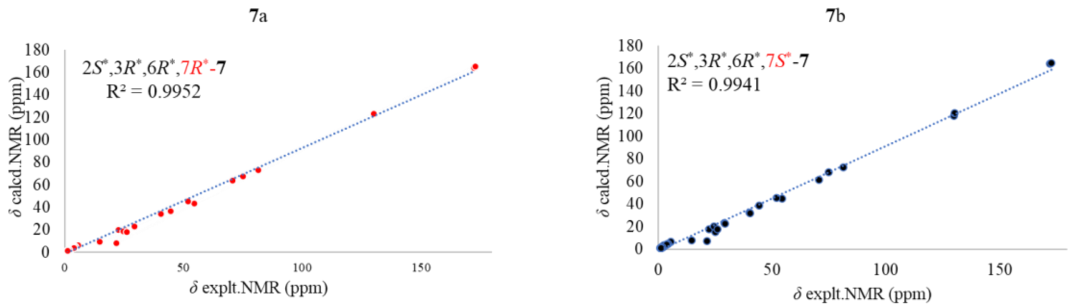

Figure S 105. Correlation between the calculated ^{13}C NMR data for 2*S*, 3*R*, 6*R*, 7*R*-7 (a) and 2*S*, 3*R*, 6*R*, 7*S*-7 (b) and experimental ^{13}C NMR data of 7

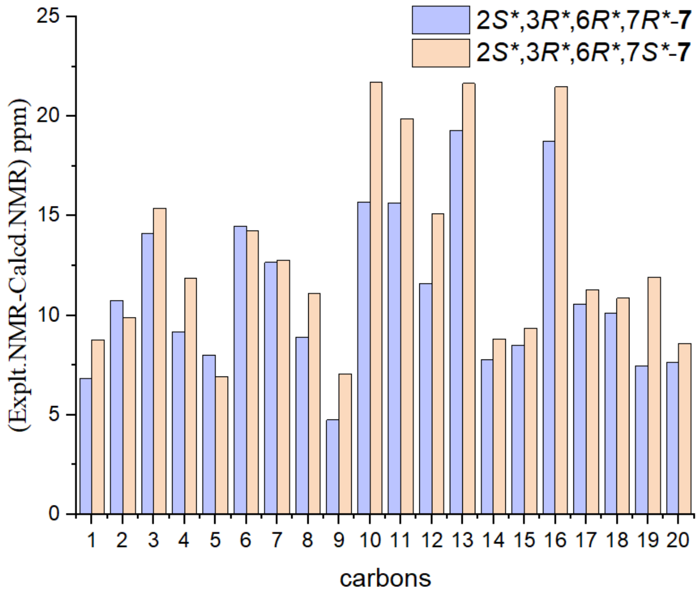

Figure S 106. Differences between experimental NMR chemical shifts of 7 and theoretical NMR chemical shifts for 2*S*, 3*R*, 6*R*, 7*R*-7 (purple bar) and 2*S*, 3*R*, 6*R*, 7*S*-7 (yellow bar)

Table S 53. Experimental chemical shifts of 7, the calculated shielding tensors for 2*S*, 3*R*, 6*R*, 7*R*-7 (isomer 1) and 2*S*, 3*R*, 6*R*, 7*S*-7 (isomer 2), as well as their DP4+ probability

	A	B	C	D	E	F	G	H
1	Functional		Solvent?		Basis Set		Type of Data	
2	mPW1PW91		PCM		6-31+G(d,p)		Shielding Tensors	
3								
12			DP4+	100.00%	0.00%	-	-	-
14	Nuclei	sp2?	xperimenta	Isomer 1	Isomer 2	Isomer 3	Isomer 4	Isomer 5
15	C		54.4	145	144.1			
16	C		74.9	121.2	120.4			
17	C		40	154.4	156.2			
18	C		22.5	168.7	170.76			
19	C	x	129.9	65.8	70.6			
20	C	x	130.2	65.4	68.3			
21	C		21.6	180.7	181.5			
22	C		25.1	168.8	173.2			
23	C		70.6	124.7	127.5			
24	C	x	172.3	24.4	24.8			
25	C		29.1	164.9	165.6			
26	C		29.3	165.4	165.9			
27	C	x	172.9	23.2	23.96			
28	C		52	143.4	143.48			
29	C		44.4	151.7	150.2			
30	C		81.5	115.7	116			
31	C		40.5	154.2	156.5			
32	C		24.5	170	168.5			
33	C		14.7	179.2	181.06			
34	C		26.2	170.5	170.9			
35	H		1.85	29.76	29.45			
36	H		1.51	29.72	29.86			
37	H		1.51	30.39	30.31			
38	H		2.12	29.31	28.99			
39	H		2.12	29.69	29.68			
40	H	x	5.47	25.7	25.39			
41	H		1.66	29.56	29.43			
42	H		1.66	30.13	30.23			
43	H		1.66	29.76	30.09			
44	H		1.17	30.28	30.63			
45	H		1.17	30.82	30.92			
46	H		1.17	30.33	30.46			
47	H		4.48	26.53	27.2			
48	H		4.48	27.35	26.89			
49	H		2.65	28.76	29.29			
50	H		2.65	29.18	28.69			
51	H		2.65	29.01	29.32			
52	H		2.65	29.06	28.54			
53	H		3.69	27.98	28			
54	H		3.69	27.83	27.71			
55	H		3.69	27.77	27.88			
56	H		1.61	29.92	30.07			
57	H		1.57	30.11	30.12			
58	H		1.69	30	29.95			
59	H		1.55	30.13	30.59			
60	H		1.86	29.92	30.04			
61	H		1.05	30.74	31.01			
62	H		1.05	30.96	30.67			
63	H		1.05	30.62	30.37			
64	H		1.26	30.88	31.06			
65	H		1.26	30.28	30.35			
66	H		1.26	30.44	30.26			

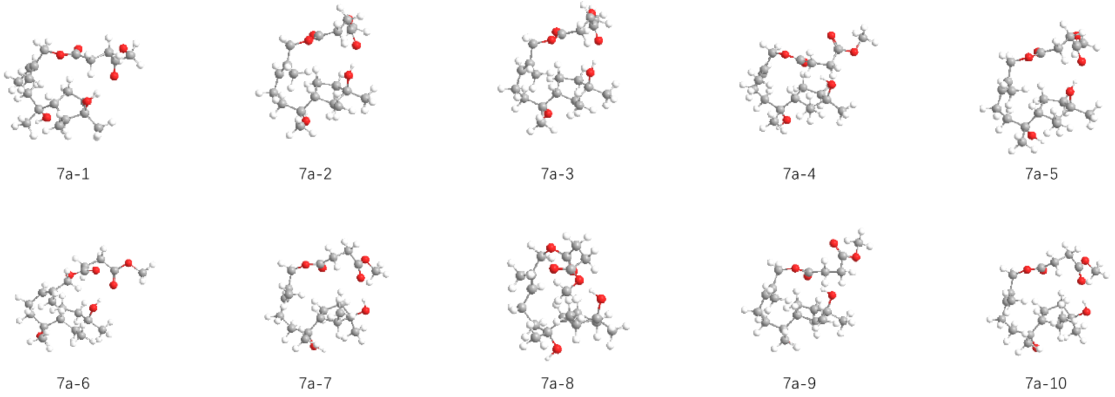

Figure S 107. Most stable conformers of 2*S*, 3*R*, 6*R*, 7*R*-7 at the CAM-B3LYP/DGDZVP level

Table S 54. Optimized Z-Matrixes of 2*S*, 3*R*, 6*R*, 7*R*-7 conformations in the methanol at CAM-B3LYP/DGDZVP level

	7a-1			7a-2			7a-3		
C	-1.84255	-0.70795	-0.08877	-1.8541	-0.69335	-0.0698	-1.84242	-0.70804	-0.08886
C	-3.2254	-0.69604	-0.76586	-3.23407	-0.7029	-0.76634	-3.22535	-0.69608	-0.76578
C	-3.3935	0.495931	-1.73418	-3.42107	0.495943	-1.72279	-3.39345	0.495796	-1.73425
C	-3.29514	1.913638	-1.141	-3.31512	1.90786	-1.11761	-3.29509	1.913559	-1.14124
C	-1.89221	2.435077	-0.98397	-1.91135	2.43162	-0.9767	-1.89218	2.434986	-0.98408
C	-1.27236	2.827759	0.136808	-1.27975	2.827337	0.136463	-1.27245	2.827758	0.136735
C	-1.83424	2.73986	1.531187	-1.82819	2.746397	1.5367	-1.83446	2.739894	1.531053
C	-4.36934	-0.70523	0.254309	-4.38288	-0.74636	0.239891	-4.36917	-0.70499	0.254523
O	-3.30015	-1.90432	-1.54618	-3.39631	-1.90659	-1.54097	-3.30035	-1.90444	-1.54597
C	0.096282	3.446574	0.040954	0.088678	3.444451	0.024328	0.096169	3.446622	0.040952
O	1.04769	2.767964	0.901635	1.043729	2.773228	0.886921	1.047553	2.768036	0.901719
C	1.926835	1.932063	0.341764	1.93211	1.944809	0.330968	1.926588	1.931984	0.341923
O	1.988024	1.690246	-0.84956	2.002342	1.704898	-0.86035	1.987746	1.690062	-0.84938
C	2.846873	1.331487	1.378545	2.846865	1.345903	1.373254	2.846566	1.331366	1.378723
C	4.140891	0.804838	0.769702	4.141286	0.812292	0.771926	4.140659	0.804878	0.769906
C	3.939392	-0.37639	-0.14603	3.939605	-0.37588	-0.13467	3.939335	-0.37619	-0.14606
O	4.717718	-0.32669	-1.22315	4.725119	-0.33966	-1.207	4.717904	-0.32634	-1.22299
O	3.190148	-1.3129	0.085396	3.184063	-1.30655	0.100033	3.190033	-1.31273	0.085022
C	4.612053	-1.43513	-2.13406	4.62176	-1.45667	-2.10775	4.612457	-1.43466	-2.13411
C	-1.63073	-1.83821	0.956816	-1.63405	-1.84418	0.950571	-1.63059	-1.83819	0.956855
C	-0.17721	-2.30603	0.745003	-0.17922	-2.30547	0.731179	-0.17712	-2.30615	0.744973
C	-0.01169	-2.18346	-0.76991	-0.0072	-2.13813	-0.77937	-0.0117	-2.18376	-0.76998
C	-0.67454	-0.84692	-1.10126	-0.66508	-0.7883	-1.06397	-0.67453	-0.84726	-1.10147
C	-1.92367	-1.42743	2.398722	-1.93229	-1.4657	2.399931	-1.92332	-1.42723	2.398753
O	0.674877	-1.35753	1.405549	0.670481	-1.37797	1.422424	0.675072	-1.35765	1.405365
C	0.110723	-3.70565	1.278036	0.105371	-3.72091	1.22217	0.110756	-3.70574	1.278112
H	-1.74694	0.246056	0.432618	-1.78317	0.252131	0.471038	-1.74663	0.246028	0.43237
H	-2.67234	0.394345	-2.55035	-2.71136	0.416783	-2.55425	-2.67227	0.394093	-2.55038
H	-4.38436	0.392216	-2.19301	-4.41523	0.381621	-2.16653	-4.3843	0.392006	-2.19307

H	-3.85472	1.975955	-0.20569	-3.86039	1.957795	-0.17313	-3.85481	1.976004	-0.20601
H	-3.80509	2.588969	-1.83718	-3.83841	2.588024	-1.79901	-3.80492	2.588841	-1.83755
H	-1.33854	2.518519	-1.91972	-1.36744	2.512898	-1.91843	-1.3384	2.518346	-1.91978
H	-2.84106	2.323079	1.558073	-2.83653	2.334095	1.574973	-2.84148	2.323548	1.557782
H	-1.86112	3.728639	2.001289	-1.84668	3.737146	2.003016	-1.86095	3.728603	2.001319
H	-1.19413	2.106821	2.153866	-1.18448	2.113488	2.155805	-1.19468	2.106469	2.153679
H	-4.28437	0.113521	0.973153	-4.30883	0.0646	0.967729	-4.28405	0.11388	0.973211
H	-5.3344	-0.60476	-0.25456	-5.34144	-0.65846	-0.27926	-5.33429	-0.60454	-0.25425
H	-4.37898	-1.64556	0.80911	-4.38088	-1.69293	0.784107	-4.37881	-1.64523	0.80949
H	-4.18099	-1.94718	-1.94235	-2.72998	-1.90459	-2.24159	-4.18134	-1.94741	-1.94179
H	0.471676	3.449642	-0.9813	0.457602	3.435414	-1.00021	0.47165	3.449688	-0.98126
H	0.081407	4.469533	0.424043	0.077177	4.471687	0.395914	0.081256	4.469576	0.424046
H	2.292998	0.537052	1.886343	2.288444	0.555133	1.881994	2.29267	0.536844	1.886364
H	3.06963	2.087004	2.133059	3.068524	2.103671	2.125845	3.069223	2.086793	2.13336
H	4.672189	1.591175	0.234115	4.675712	1.593098	0.231413	4.671965	1.591332	0.234491
H	4.79421	0.458866	1.575649	4.791959	0.471284	1.582147	4.793957	0.458821	1.575837
H	5.298414	-1.20945	-2.94574	5.31712	-1.24306	-2.91501	5.299064	-1.20887	-2.94554
H	4.899389	-2.36171	-1.637	4.898887	-2.3798	-1.59864	4.899607	-2.36132	-1.63708
H	3.59123	-1.51657	-2.50787	3.604134	-1.53599	-2.49061	3.591735	-1.51598	-2.50821
H	-2.26975	-2.68793	0.689367	-2.26997	-2.6899	0.662647	-2.2697	-2.68789	0.689561
H	-0.55248	-3.00427	-1.25281	-0.54316	-2.94725	-1.28837	-0.55254	-3.00463	-1.25273
H	1.036983	-2.24129	-1.07803	1.041919	-2.18613	-1.08602	1.036953	-2.24166	-1.07817
H	-1.00984	-0.81857	-2.13903	-0.95412	-0.69026	-2.11449	-1.00994	-0.81904	-2.13921
H	0.03434	-0.02704	-0.96571	0.041164	0.021293	-0.87156	0.034404	-0.02738	-0.96614
H	-2.97056	-1.14678	2.531818	-2.981	-1.19259	2.534715	-2.97015	-1.14641	2.531944
H	-1.30576	-0.57319	2.686076	-1.31908	-0.61508	2.707286	-1.30524	-0.57306	2.685961
H	-1.71241	-2.24543	3.094965	-1.71966	-2.29759	3.078944	-1.71211	-2.2452	3.095051
H	1.556179	-1.42062	0.997065	1.555323	-1.43263	1.020202	1.556339	-1.42069	0.996771
H	-0.54125	-4.44843	0.809137	-0.5431	-4.44881	0.725973	-0.54131	-4.44852	0.809333
H	-0.03816	-3.74699	2.360756	-0.05181	-3.79524	2.301832	-0.03803	-3.74699	2.36085
H	1.149227	-3.98267	1.069477	1.145387	-3.99133	1.012868	1.149223	-3.98285	1.069482
	7a-4			7a-5			7a-6		
C	-1.76419	-0.88061	-0.16325	-1.84986	-0.70719	-0.09281	-1.90162	-0.87316	-0.21533
C	-3.29303	-0.74993	-0.00842	-3.23406	-0.69276	-0.78106	-3.4144	-0.79813	-0.52958
C	-3.91211	0.247817	-1.01251	-3.39661	0.502595	-1.73808	-3.758	0.415644	-1.41825
C	-3.65365	1.747752	-0.79551	-3.28882	1.911575	-1.12929	-3.65328	1.819616	-0.7923
C	-2.24205	2.18871	-1.0632	-1.88481	2.432817	-0.97981	-2.23641	2.22391	-0.49424
C	-1.42657	2.888116	-0.2645	-1.25869	2.825362	0.137532	-1.70651	2.562232	0.685138
C	-1.73252	3.327075	1.143601	-1.81176	2.735116	1.535353	-2.46151	2.69488	1.982115
C	-3.69043	-0.36347	1.419887	-4.38394	-0.71134	0.231713	-4.27994	-0.77463	0.733515
O	-3.83028	-2.05223	-0.30719	-3.3756	-1.83306	-1.65055	-3.81558	-1.92327	-1.33703
C	-0.07733	3.312358	-0.78604	0.106306	3.451007	0.033107	-0.21846	2.723267	0.868876
O	0.983809	3.026019	0.16108	1.067141	2.78201	0.890721	0.482819	2.285478	-0.30361

C	1.442638	1.77715	0.216091	1.937195	1.937026	0.330523	1.75727	1.914179	-0.15802
O	1.078289	0.89244	-0.54112	1.982233	1.679251	-0.85814	2.389275	2.026796	0.875058
C	2.430142	1.607528	1.345034	2.869724	1.348434	1.363204	2.300494	1.335777	-1.4429
C	3.241214	0.326318	1.262677	4.152668	0.807307	0.744165	3.799387	1.091905	-1.39694
C	4.132182	0.277431	0.045184	3.933173	-0.38689	-0.15015	4.204008	-0.02136	-0.46295
O	4.700022	-0.92471	-0.09483	4.704269	-0.36294	-1.23305	5.47296	0.090202	-0.07896
O	4.333651	1.207497	-0.71343	3.176447	-1.31206	0.102136	3.489198	-0.95213	-0.12565
C	5.582413	-1.08284	-1.21733	4.581978	-1.48561	-2.12441	5.977992	-0.95667	0.769294
C	-1.09031	-1.83094	0.869489	-1.63697	-1.82852	0.964096	-1.40127	-2.00793	0.716954
C	-0.04539	-2.64802	0.067678	-0.17935	-2.29007	0.761272	0.069369	-2.19606	0.284563
C	-0.71556	-2.78612	-1.30074	-0.01295	-2.18387	-0.75489	-0.01709	-2.09111	-1.23819
C	-1.32986	-1.4089	-1.55781	-0.6825	-0.8548	-1.10321	-1.03008	-0.9697	-1.49589
C	-0.50315	-1.12483	2.090183	-1.94271	-1.4089	2.400601	-1.54129	-1.73218	2.214593
O	1.200432	-1.94879	-0.06188	0.660634	-1.32684	1.412949	0.793347	-1.07624	0.81028
C	0.302768	-3.98258	0.708704	0.11695	-3.68109	1.311679	0.70829	-3.49632	0.761296
H	-1.35485	0.122197	-0.02409	-1.75039	0.246299	0.428893	-1.64965	0.054967	0.29583
H	-3.61091	-0.02784	-2.02717	-2.67483	0.406092	-2.55465	-3.14253	0.381339	-2.3225
H	-4.99854	0.101268	-0.9709	-4.38579	0.398215	-2.1955	-4.79282	0.270859	-1.74661
H	-3.98751	2.045493	0.199931	-3.83988	1.966068	-0.18842	-4.29023	1.884263	0.09238
H	-4.30171	2.280596	-1.50154	-3.8046	2.594651	-1.81348	-4.07288	2.519667	-1.524
H	-1.85892	1.900356	-2.043	-1.33736	2.519282	-1.91897	-1.55724	2.129758	-1.33883
H	-2.77385	3.164651	1.42034	-2.81397	2.307677	1.569021	-3.53671	2.793634	1.834656
H	-1.50824	4.390365	1.275136	-1.84673	3.724797	2.003066	-2.12091	3.571141	2.543066
H	-1.10754	2.780068	1.857417	-1.16135	2.110746	2.156069	-2.28251	1.819542	2.617679
H	-3.16616	0.535316	1.75494	-4.30091	0.100087	0.959116	-3.93127	-0.02626	1.450075
H	-4.76678	-0.16579	1.476901	-5.34123	-0.61408	-0.28794	-5.31813	-0.54885	0.472018
H	-3.46045	-1.17363	2.114912	-4.40078	-1.65186	0.789854	-4.26795	-1.74752	1.232005
H	-4.78483	-2.0211	-0.15757	-3.36117	-2.63064	-1.10482	-3.63605	-2.72857	-0.83345
H	0.155913	2.845654	-1.74313	0.4763	3.453422	-0.99116	0.056361	3.762534	1.076342
H	-0.02754	4.397541	-0.89071	0.087332	4.474941	0.413302	0.106291	2.119215	1.721185
H	1.850141	1.617072	2.273158	2.320779	0.564041	1.891523	2.053728	2.016768	-2.25975
H	3.073713	2.48816	1.374057	3.106087	2.114484	2.10282	1.756836	0.407812	-1.63966
H	3.88597	0.245709	2.142303	4.679395	1.582941	0.188831	4.147282	0.797199	-2.39156
H	2.605005	-0.56173	1.261141	4.81649	0.471026	1.545654	4.344998	1.996541	-1.12739
H	5.9392	-2.10818	-1.16478	5.266138	-1.28093	-2.94345	7.010407	-0.68838	0.97627
H	5.041869	-0.91644	-2.14948	4.862255	-2.40658	-1.61311	5.402318	-1.00191	1.693647
H	6.417701	-0.38559	-1.14575	3.55828	-1.56241	-2.49125	5.928664	-1.9161	0.254267
H	-1.83938	-2.55509	1.208569	-2.26257	-2.69894	0.720657	-1.91079	-2.95375	0.481761
H	-1.50805	-3.5389	-1.239	-0.54611	-3.01562	-1.22927	-0.38772	-3.04025	-1.64035
H	-0.00144	-3.1009	-2.06669	1.036055	-2.24076	-1.06094	0.965404	-1.9105	-1.68717
H	-2.15663	-1.47173	-2.26606	-1.02325	-0.84494	-2.13966	-1.63233	-1.19261	-2.37854
H	-0.58867	-0.7314	-1.99349	0.022907	-0.03007	-0.98184	-0.52922	-0.01496	-1.67555
H	-1.28246	-0.65294	2.692474	-2.99224	-1.13448	2.524037	-2.58144	-1.74649	2.541039

H	0.198854	-0.34211	1.790409	-1.33202	-0.54814	2.682775	-1.12112	-0.75449	2.464053
H	0.035788	-1.82592	2.734853	-1.72966	-2.21986	3.104016	-1.00842	-2.48557	2.802361
H	1.039469	-1.0279	-0.33255	1.545562	-1.39014	1.01181	1.687427	-1.088	0.426796
H	-0.59022	-4.6025	0.819357	-0.5279	-4.43484	0.850434	0.137509	-4.36474	0.420228
H	0.749302	-3.83651	1.696467	-0.03362	-3.71031	2.394441	0.768998	-3.52563	1.852307
H	1.023151	-4.5222	0.08712	1.157811	-3.9523	1.108233	1.725795	-3.58242	0.366143
	7a-7			7a-8			7a-9		
C	-1.54969	-0.87841	-0.01714	-1.80423	-0.43823	-0.06388	-1.76899	-0.88035	-0.16387
C	-3.08927	-0.96379	0.046468	-2.97953	-0.03714	0.848737	-3.30506	-0.75111	-0.01809
C	-3.77242	-0.0636	-0.99778	-3.5406	1.363726	0.521619	-3.91402	0.248211	-1.01937
C	-3.72797	1.456293	-0.77282	-2.64173	2.581852	0.790898	-3.65321	1.744539	-0.79036
C	-2.40069	2.116865	-1.02532	-1.5435	2.782415	-0.21667	-2.2428	2.189442	-1.05864
C	-1.64948	2.81817	-0.16626	-0.24021	2.996471	0.000022	-1.42575	2.884769	-0.25808
C	-1.93558	3.01207	1.299883	0.446456	2.998636	1.338926	-1.72705	3.311899	1.154666
C	-3.59927	-0.64221	1.455089	-2.59941	-0.10969	2.331837	-3.71068	-0.37085	1.409253
O	-3.53618	-2.28404	-0.31972	-4.02554	-0.99297	0.589884	-3.94483	-1.99408	-0.36903
C	-0.42647	3.533898	-0.67695	0.640461	3.319447	-1.1822	-0.08036	3.317024	-0.78296
O	0.722047	3.336206	0.184034	1.942936	2.682327	-1.13575	0.986145	3.032046	0.158555
C	1.582085	2.360335	-0.12267	1.991435	1.370484	-1.34822	1.444544	1.78329	0.213376
O	1.464174	1.614081	-1.07619	0.998606	0.689007	-1.54288	1.075608	0.89749	-0.54061
C	2.725848	2.31438	0.863867	3.405759	0.844358	-1.38568	2.436704	1.615177	1.338444
C	3.939298	1.578793	0.312508	3.543063	-0.54364	-0.77748	3.243182	0.330971	1.259131
C	3.72397	0.091836	0.191219	3.356679	-0.53394	0.720039	4.130561	0.273697	0.039397
O	4.363395	-0.43189	-0.85032	3.537171	-1.74809	1.245304	4.695432	-0.9304	-0.09555
O	3.07769	-0.56859	0.989379	3.070005	0.448862	1.378946	4.331686	1.199155	-0.72487
C	4.298971	-1.8626	-0.99472	3.348384	-1.85783	2.665538	5.574109	-1.09662	-1.21983
C	-0.78871	-1.80809	0.974084	-1.16707	-1.81635	0.276772	-1.08671	-1.82117	0.874423
C	0.221861	-2.61971	0.101998	-0.88471	-2.49849	-1.08597	-0.04149	-2.64133	0.074199
C	0.430343	-1.72393	-1.12631	-2.03948	-1.99013	-1.95303	-0.71659	-2.78809	-1.29103
C	-0.95648	-1.15634	-1.41297	-2.18688	-0.51645	-1.56698	-1.33265	-1.41317	-1.55479
C	-0.12222	-0.99574	2.084604	0.065385	-1.7302	1.17278	-0.50367	-1.10305	2.089833
O	1.43924	-2.93617	0.771696	0.374023	-2.09331	-1.64171	1.199763	-1.93869	-0.06108
C	-0.37534	-3.96786	-0.2929	-0.81931	-4.01584	-0.99889	0.311832	-3.97099	0.722492
H	-1.28862	0.149838	0.249521	-1.03974	0.332531	0.060987	-1.35891	0.122198	-0.02423
H	-3.37287	-0.30128	-1.98868	-3.8709	1.382335	-0.52129	-3.60321	-0.01924	-2.03356
H	-4.82563	-0.3628	-1.01268	-4.44756	1.486492	1.126855	-4.99698	0.091265	-0.98261
H	-4.11237	1.697232	0.219881	-2.25493	2.549929	1.811209	-3.98578	2.036599	0.207142
H	-4.43995	1.894016	-1.482	-3.28942	3.465584	0.744764	-4.30263	2.281817	-1.4917
H	-2.04032	2.027916	-2.05077	-1.88319	2.766666	-1.25346	-1.86286	1.909698	-2.04223
H	-2.94457	2.707872	1.577768	-0.25347	2.895291	2.167975	-2.76785	3.148371	1.432742
H	-1.80758	4.061042	1.584754	1.010971	3.925895	1.483399	-1.50094	4.373645	1.295044
H	-1.23106	2.429936	1.903468	1.165935	2.175416	1.399854	-1.10106	2.757691	1.862083
H	-3.21577	0.316978	1.811223	-1.69438	0.464236	2.546695	-3.18158	0.522092	1.752472

H	-4.69253	-0.60592	1.465181	-3.40777	0.291225	2.953425	-4.78609	-0.17582	1.454843
H	-3.28075	-1.40446	2.172291	-2.427	-1.14502	2.632677	-3.48767	-1.17842	2.112326
H	-3.21028	-2.90667	0.344105	-4.75977	-0.79895	1.187996	-3.6451	-2.67402	0.249169
H	-0.17367	3.236366	-1.69427	0.154602	3.06289	-2.12484	0.151214	2.855319	-1.7429
H	-0.58283	4.614356	-0.6451	0.895878	4.380543	-1.18771	-0.03587	4.402807	-0.88339
H	2.360663	1.82826	1.773055	4.072503	1.558139	-0.90327	1.86093	1.631516	2.269109
H	2.992713	3.335634	1.138349	3.683753	0.799493	-2.44275	3.083336	2.49379	1.360296
H	4.248836	1.982509	-0.65132	2.817059	-1.24217	-1.20263	3.890056	0.251853	2.137316
H	4.777863	1.70934	1.002746	4.531879	-0.95395	-0.99426	2.604095	-0.55497	1.263086
H	4.91792	-2.09334	-1.85768	3.497339	-2.90901	2.898433	5.928587	-2.12251	-1.16289
H	4.690878	-2.34973	-0.10146	4.078686	-1.24341	3.193045	5.031191	-0.93383	-2.15123
H	3.27082	-2.18042	-1.16713	2.338718	-1.54741	2.936528	6.411269	-0.40101	-1.15439
H	-1.46483	-2.52092	1.46138	-1.92561	-2.43353	0.771031	-1.81476	-2.55744	1.241279
H	0.864152	-2.27048	-1.96951	-2.95203	-2.53426	-1.68839	-1.5081	-3.5428	-1.2241
H	1.109105	-0.90344	-0.86745	-1.84217	-2.15003	-3.01673	-0.00554	-3.10883	-2.05691
H	-1.56792	-1.88774	-1.95034	-3.19652	-0.1557	-1.76749	-2.16131	-1.48242	-2.26027
H	-0.91234	-0.25109	-2.02276	-1.503	0.106434	-2.15124	-0.59344	-0.7368	-1.99493
H	-0.87748	-0.42866	2.63712	-0.19197	-1.39461	2.179711	-1.2869	-0.63307	2.688341
H	0.596163	-0.27842	1.673567	0.784836	-1.0161	0.764235	0.19096	-0.31758	1.781651
H	0.408292	-1.63791	2.792387	0.566367	-2.69913	1.261761	0.04109	-1.79595	2.737917
H	1.955408	-2.11964	0.887097	0.460367	-1.12471	-1.60275	1.037372	-1.01936	-0.33689
H	-1.33271	-3.8448	-0.80465	-1.75613	-4.42189	-0.60967	-0.57779	-4.59514	0.836719
H	-0.54075	-4.58034	0.598548	-0.00356	-4.33372	-0.34306	0.758199	-3.81752	1.709086
H	0.306334	-4.50221	-0.96081	-0.64324	-4.44293	-1.99046	1.0342	-4.51003	0.103084
	7a-10								
C	-1.55752	-0.88184	-0.02011						
C	-3.09735	-0.94647	0.060135						
C	-3.78492	-0.03448	-0.97728						
C	-3.72294	1.48616	-0.75578						
C	-2.39163	2.133201	-1.01997						
C	-1.62719	2.826441	-0.16609						
C	-1.89904	3.020032	1.302764						
C	-3.58619	-0.62318	1.470408						
O	-3.55356	-2.29183	-0.1819						
C	-0.40457	3.535835	-0.6869						
O	0.746559	3.341346	0.170515						
C	1.602165	2.360141	-0.13143						
O	1.480282	1.609289	-1.08089						
C	2.745609	2.314719	0.855307						
C	3.95367	1.566005	0.310496						
C	3.724656	0.080568	0.195979						
O	4.369121	-0.45507	-0.83652						
O	3.064377	-0.56887	0.991538						

C	4.291888	-1.88576	-0.9742						
C	-0.81315	-1.82541	0.966429						
C	0.207016	-2.62834	0.09645						
C	0.417182	-1.72989	-1.13023						
C	-0.96921	-1.16352	-1.41913						
C	-0.15479	-1.03465	2.097005						
O	1.427331	-2.93769	0.765774						
C	-0.37922	-3.98131	-0.29742						
H	-1.27789	0.144413	0.237486						
H	-3.4003	-0.26857	-1.97735						
H	-4.84155	-0.32265	-0.97926						
H	-4.09501	1.728177	0.241283						
H	-4.43782	1.930799	-1.45763						
H	-2.04157	2.043375	-2.04893						
H	-2.90738	2.721895	1.589356						
H	-1.76141	4.067665	1.588013						
H	-1.19306	2.432178	1.898983						
H	-3.17268	0.323368	1.824604						
H	-4.67791	-0.55544	1.490657						
H	-3.28149	-1.40537	2.169302						
H	-3.42578	-2.48842	-1.11945						
H	-0.157	3.231522	-1.70353						
H	-0.55902	4.616727	-0.66128						
H	2.376447	1.838692	1.768203						
H	3.019409	3.336376	1.121356						
H	4.268853	1.961924	-0.65478						
H	4.792126	1.692579	1.001611						
H	4.918161	-2.12731	-1.82889						
H	4.668733	-2.37212	-0.07405						
H	3.262545	-2.19422	-1.15618						
H	-1.51614	-2.53605	1.410545						
H	0.855722	-2.27101	-1.97442						
H	1.090874	-0.90657	-0.86686						
H	-1.56231	-1.90067	-1.97326						
H	-0.9296	-0.2614	-2.03366						
H	-0.91107	-0.4694	2.649926						
H	0.575601	-0.31622	1.708576						
H	0.361252	-1.69188	2.801976						
H	1.936011	-2.11751	0.887096						
H	-1.35753	-3.86978	-0.76923						
H	-0.50836	-4.6022	0.593959						
H	0.289279	-4.49985	-0.99095						

Table S 55. Energy analysis for 2*S*, 3*R*, 6*R*, 7*R*-7

Conf.	Steric Energy (kJ/mol)	Relative Energy (kJ/mol)	Distribution (%)
7a-1	-1233.291511	0.000942	9.59%
7a-2	-1233.291149	0.001304	6.54%
7a-3	-1233.291515	0.000938	9.63%
7a-4	-1233.289971	0.002482	1.88%
7a-5	-1233.291307	0.001146	7.73%
7a-6	-1233.289781	0.002672	1.54%
7a-7	-1233.291528	0.000925	9.77%
7a-8	-1233.292392	0.000061	24.38%
7a-9	-1233.292453	0	26.01%
7a-10	-1233.290391	0.002062	2.93%

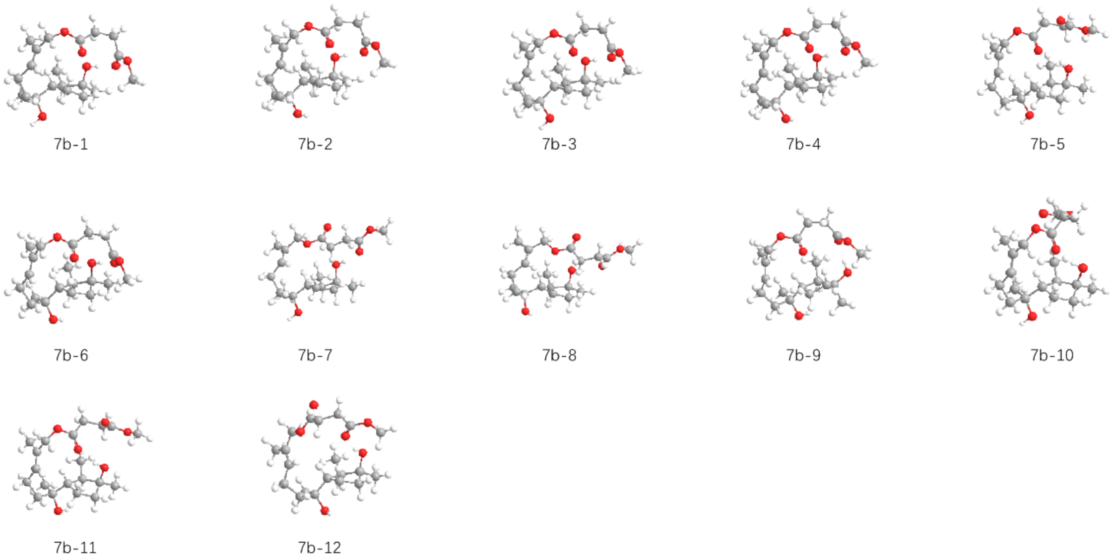

Figure S 108. Most stable conformers of 2*R*, 3*S*, 6*S*, 7*S*-7 at the CAM-B3LYP/DGDZVP level

Table S 56. Optimized Z-Matrixes of 2*R*, 3*S*, 6*S*, 7*S*-7 conformations in the methanol at CAM-B3LYP/DGDZVP level

	7b-1			7b-2			7b-3		
C	1.842564	-0.70793	-0.08879	1.854094	-0.69335	-0.0698	1.842294	-0.7081	-0.08908
C	1.630705	-1.83816	0.956819	1.634079	-1.84419	0.950569	1.63101	-1.83798	0.957001
C	0.177195	-2.306	0.744958	0.179266	-2.30552	0.731175	0.177597	-2.30632	0.745537
C	0.011718	-2.18345	-0.76997	0.007236	-2.13817	-0.77937	0.011992	-2.18471	-0.76946
C	0.674584	-0.84693	-1.10132	0.665072	-0.78832	-1.06396	0.674417	-0.84817	-1.10162
C	-0.11075	-3.7056	1.278016	-0.10528	-3.72098	1.22215	-0.11002	-3.70568	1.27941
C	3.225444	-0.69605	-0.76582	3.234059	-0.70287	-0.76634	3.225142	-0.69566	-0.76613
C	3.393586	0.49588	-1.7342	3.42103	0.495985	-1.72279	3.392575	0.49624	-1.7347
C	3.2952	1.91361	-1.14108	3.315061	1.907897	-1.1176	3.294149	1.91399	-1.14169
C	1.892263	2.435005	-0.98402	1.911281	2.431633	-0.9767	1.891231	2.43532	-0.98416
C	1.272431	2.827659	0.136776	1.279693	2.82739	0.136466	1.271798	2.827854	0.136913
C	-0.09623	3.446444	0.040966	-0.08874	3.444492	0.024336	-0.09674	3.446967	0.041628

O	3.300239	-1.90436	-1.5461	3.396312	-1.90655	-1.54099	3.300469	-1.90405	-1.54625
C	4.369338	-0.70517	0.254395	4.382879	-0.74631	0.239874	4.369136	-0.70411	0.253968
C	1.834348	2.739753	1.531138	1.828164	2.746515	1.536696	1.834153	2.739707	1.531082
O	-1.04759	2.767798	0.901672	-1.04378	2.773259	0.886931	-1.04821	2.768239	0.90219
H	1.746933	0.24609	0.432561	1.783152	0.252128	0.471046	1.746036	0.246076	0.431872
C	1.923569	-1.42733	2.398725	1.932311	-1.46571	2.399932	1.923957	-1.42652	2.398716
O	-0.67492	-1.35748	1.40546	-0.67046	-1.37806	1.422436	-0.67467	-1.35758	1.405511
C	-1.92684	1.932007	0.341815	-1.93214	1.94482	0.330977	-1.92692	1.932003	0.342146
C	-2.8468	1.331387	1.378641	-2.84686	1.345863	1.373266	-2.84693	1.331051	1.378723
O	-1.98816	1.69028	-0.84952	-2.00238	1.704926	-0.86035	-1.98774	1.69017	-0.84918
C	-4.14086	0.80477	0.769869	-4.14128	0.812229	0.771956	-4.14081	0.804276	0.76967
C	-3.93942	-0.37633	-0.14603	-3.93959	-0.37593	-0.13466	-3.93903	-0.3767	-0.14633
O	-4.71794	-0.32657	-1.22301	-4.72508	-0.33968	-1.20701	-4.7171	-0.32677	-1.22362
O	-3.19007	-1.3128	0.085154	-3.18405	-1.3066	0.100037	-3.18981	-1.31323	0.085052
C	-4.61236	-1.4349	-2.13407	-4.62169	-1.45666	-2.10779	-4.61113	-1.43497	-2.13483
H	2.269734	-2.68789	0.689425	2.270029	-2.68989	0.66264	2.270256	-2.68761	0.689812
H	-1.03695	-2.24128	-1.07811	-1.04189	-2.18619	-1.08602	-1.03668	-2.24303	-1.07751
H	0.55251	-3.00428	-1.25284	0.54321	-2.94726	-1.28838	0.552996	-3.00565	-1.25189
H	1.009911	-0.81859	-2.13909	0.954106	-0.69025	-2.11448	1.009828	-0.82041	-2.13937
H	-0.0343	-0.02704	-0.96581	-0.04119	0.021253	-0.87153	-0.03477	-0.02845	-0.96666
H	0.038092	-3.74692	2.360742	0.051897	-3.79532	2.301811	0.038815	-3.74636	2.362161
H	-1.14925	-3.98263	1.069429	-1.14529	-3.99142	1.012848	-1.14845	-3.98306	1.070964
H	0.541232	-4.4484	0.809155	0.543208	-4.44885	0.725942	0.542141	-4.44859	0.810973
H	4.384471	0.392147	-2.19297	4.415197	0.381683	-2.16654	4.383262	0.392629	-2.19392
H	2.672461	0.394259	-2.55039	2.711327	0.416813	-2.55425	2.671073	0.394327	-2.55053
H	3.854813	1.975988	-0.20579	3.860332	1.957841	-0.17312	3.854095	1.976601	-0.20661
H	3.805106	2.588921	-1.83731	3.838335	2.588076	-1.799	3.803709	2.589333	-1.83815
H	1.338568	2.51844	-1.91976	1.367352	2.512866	-1.91841	1.337262	2.518972	-1.91971
H	-0.08137	4.469398	0.42407	-0.07724	4.471728	0.395923	-0.08156	4.469731	0.425221
H	-0.47165	3.449521	-0.98127	-0.45767	3.435451	-1.0002	-0.47235	3.450626	-0.98054
H	4.181099	-1.94725	-1.94222	2.730001	-1.90454	-2.24161	4.181485	-1.94683	-1.94204
H	4.284344	0.113629	0.973175	4.30882	0.064622	0.967734	4.283825	0.114663	0.972741
H	4.378938	-1.64547	0.809265	4.380911	-1.6929	0.784068	4.379288	-1.64439	0.808858
H	5.334425	-0.60475	-0.25443	5.341433	-0.65838	-0.27928	5.334106	-0.60319	-0.255
H	2.841209	2.323049	1.557982	2.836506	2.334218	1.574969	2.840462	2.321648	1.557679
H	1.861169	3.728515	2.001279	1.846661	3.737286	2.002965	1.862504	3.728602	2.00086
H	1.194305	2.106639	2.153808	1.184464	2.113634	2.155841	1.193513	2.107658	2.154214
H	1.712315	-2.24532	3.094983	1.719707	-2.29762	3.078939	1.712953	-2.24429	3.095312
H	2.970443	-1.14664	2.531853	2.981016	-1.19257	2.534717	2.970783	-1.14556	2.531633
H	1.30562	-0.5731	2.686029	1.319069	-0.61512	2.707295	1.30585	-0.57231	2.68575
H	-1.55619	-1.42055	0.996915	-1.55531	-1.43274	1.02022	-1.55595	-1.42099	0.997047
H	-2.29288	0.536936	1.88637	-2.28841	0.555096	1.881977	-2.29295	0.536603	1.886381
H	-3.0695	2.086879	2.133196	-3.06853	2.103608	2.125878	-3.0699	2.086348	2.133401

H	-4.79409	0.458681	1.575838	-4.79193	0.471197	1.582189	-4.79411	0.457997	1.575508
H	-4.67224	1.591154	0.23443	-4.67573	1.59303	0.231464	-4.67224	1.590636	0.234236
H	-5.29889	-1.20918	-2.94559	-4.89882	-2.37981	-1.59871	-5.2973	-1.20908	-2.94661
H	-3.5916	-1.51622	-2.50808	-5.31704	-1.24303	-2.91506	-3.5902	-1.51623	-2.50839
H	-4.89953	-2.36156	-1.63705	-3.60406	-1.53597	-2.49063	-4.89853	-2.3617	-1.6381
	7b-4			7b-5			7b-6		
C	1.849746	-0.70703	-0.09277	1.759785	-0.88431	-0.16267	1.849859	-0.70719	-0.09281
C	1.63694	-1.82845	0.964065	1.081487	-1.83209	0.869516	1.636966	-1.82852	0.964096
C	0.179399	-2.29017	0.761206	0.034772	-2.64585	0.066688	0.179347	-2.29007	0.761272
C	0.012959	-2.1838	-0.75495	0.70618	-2.78671	-1.30085	0.012947	-2.18387	-0.75489
C	0.682347	-0.85461	-1.10312	1.325607	-1.41172	-1.55761	0.682497	-0.8548	-1.10321
C	-0.11672	-3.68128	1.311477	-0.3186	-3.97906	0.707689	-0.11695	-3.68109	1.311678
C	3.233904	-0.69259	-0.78108	3.288771	-0.75833	-0.00576	3.234056	-0.69276	-0.78106
C	3.396463	0.502745	-1.73812	3.912399	0.237105	-1.00931	3.396609	0.502595	-1.73808
C	3.288678	1.911796	-1.12946	3.658695	1.737935	-0.79293	3.288816	1.911575	-1.12929
C	1.884636	2.432912	-0.97977	2.249007	2.183629	-1.06276	1.884812	2.432817	-0.97981
C	1.258578	2.825358	0.137644	1.43487	2.886443	-0.2657	1.258693	2.825361	0.137532
C	-0.10647	3.45093	0.033358	0.087834	3.314898	-0.78946	-0.10631	3.451007	0.033106
O	3.375352	-1.8329	-1.65057	3.82242	-2.06238	-0.30333	3.3756	-1.83306	-1.65055
C	4.38384	-0.7112	0.231628	3.68529	-0.3726	1.423	4.383935	-0.71134	0.231713
C	1.811784	2.734994	1.535403	1.740314	3.325068	1.142632	1.811764	2.735115	1.535353
O	-1.06719	2.781792	0.890974	-0.97562	3.032853	0.15641	-1.06714	2.78201	0.890721
H	1.750309	0.24643	0.429	1.35335	0.119806	-0.02448	1.750391	0.246299	0.428894
C	1.942651	-1.40891	2.400591	0.495443	-1.12362	2.0894	1.942706	-1.4089	2.400601
O	-0.66072	-1.32714	1.413021	-1.20854	-1.94244	-0.06466	-0.66063	-1.32684	1.412949
C	-1.93731	1.936858	0.33075	-1.43753	1.785164	0.212782	-1.9372	1.937026	0.330523
C	-2.86963	1.348036	1.363503	-2.42686	1.619835	1.340755	-2.86972	1.348434	1.363204
O	-1.98252	1.679282	-0.85794	-1.07432	0.898452	-0.54263	-1.98223	1.679251	-0.85814
C	-4.15259	0.806769	0.744606	-3.23674	0.337625	1.263168	-4.15267	0.807307	0.744164
C	-3.93298	-0.38706	-0.15018	-4.12679	0.28241	0.045324	-3.93317	-0.38689	-0.15015
O	-4.70383	-0.36262	-1.23325	-4.69321	-0.92099	-0.08951	-4.70427	-0.36294	-1.23305
O	-3.17635	-1.31237	0.101915	-4.32869	1.208722	-0.71776	-3.17645	-1.31206	0.102136
C	-4.58133	-1.48488	-2.12509	-5.57405	-1.08548	-1.21228	-4.58198	-1.48561	-2.12441
H	2.262629	-2.69879	0.720547	1.827713	-2.55868	1.209649	2.262566	-2.69894	0.720657
H	-1.03603	-2.2408	-1.06101	-0.00812	-3.0992	-2.06757	-1.03606	-2.24076	-1.06094
H	0.546249	-3.01541	-1.22942	1.495929	-3.5423	-1.23796	0.546105	-3.01561	-1.22927
H	1.023027	-0.84455	-2.1396	2.153136	-1.47766	-2.26469	1.023251	-0.84494	-2.13966
H	-0.02314	-0.02997	-0.98161	0.587334	-0.73186	-1.99456	-0.02291	-0.03007	-0.98184
H	0.033841	-3.71058	2.394239	-0.76566	-3.83129	1.694966	0.033615	-3.71031	2.394441
H	-1.15755	-3.95262	1.107992	-1.04018	-4.51636	0.085487	-1.15781	-3.9523	1.108233
H	0.528234	-4.43491	0.85018	0.572145	-4.602	0.819399	0.527899	-4.43484	0.850434
H	4.385651	0.398327	-2.19553	4.998285	0.086948	-0.96632	4.385792	0.398215	-2.1955
H	2.674693	0.406273	-2.55472	3.611545	-0.03777	-2.02429	2.674827	0.406092	-2.55465

H	3.839928	1.966481	-0.18872	3.992116	2.03467	0.202963	3.839875	1.966068	-0.18842
H	3.804234	2.594805	-1.81389	4.309581	2.268458	-1.49812	3.804602	2.594651	-1.81348
H	1.337118	2.519407	-1.91889	1.866208	1.896002	-2.04291	1.337363	2.519282	-1.91897
H	-0.08751	4.474844	0.413612	0.041855	4.400164	-0.89494	-0.08733	4.474941	0.413301
H	-0.47651	3.453403	-0.99089	-0.14565	2.848301	-1.74655	-0.4763	3.453421	-0.99116
H	3.361319	-2.63045	-1.10478	4.776825	-2.03418	-0.15223	3.361174	-2.63064	-1.10482
H	4.300869	0.100287	0.958978	3.163096	0.527776	1.757064	4.30091	0.100087	0.959116
H	4.400696	-1.65167	0.789851	3.452011	-1.18193	2.117899	4.400775	-1.65186	0.789854
H	5.341124	-0.61399	-0.28807	4.762135	-0.178	1.481484	5.341235	-0.61408	-0.28794
H	2.814245	2.308144	1.568847	2.781473	3.162198	1.419777	2.813971	2.307677	1.569021
H	1.846195	3.724558	2.003411	1.516393	4.38841	1.274253	1.84673	3.724796	2.003066
H	1.161785	2.110064	2.155994	1.114843	2.778186	1.856133	1.161346	2.110746	2.156069
H	1.729991	-2.22006	3.103914	-0.04883	-1.82212	2.732384	1.729655	-2.21986	3.104016
H	2.992084	-1.13409	2.523964	1.275991	-0.65644	2.693765	2.992238	-1.13448	2.524037
H	1.331655	-0.54843	2.682939	-0.20146	-0.33671	1.788673	1.332019	-0.54814	2.682775
H	-1.54559	-1.39042	1.011744	-1.04394	-1.02205	-0.33485	-1.54556	-1.39014	1.01181
H	-2.32045	0.563694	1.891659	-1.8485	1.634333	2.269832	-2.32078	0.564041	1.891523
H	-3.10601	2.113988	2.103214	-3.07118	2.500111	1.364513	-3.10609	2.114484	2.102819
H	-4.81608	0.470012	1.546172	-2.59974	-0.54986	1.265491	-4.81649	0.471027	1.545654
H	-4.67967	1.582472	0.189705	-3.88192	0.259962	2.142741	-4.6794	1.582942	0.18883
H	-5.26509	-1.27969	-2.94435	-5.9294	-2.11109	-1.15554	-5.26614	-1.28093	-2.94345
H	-3.55749	-1.56169	-2.49152	-6.41046	-0.38915	-1.14485	-3.55828	-1.56241	-2.49125
H	-4.86203	-2.40604	-1.61436	-5.03267	-0.92249	-2.14456	-4.86226	-2.40658	-1.61311
	7b-7			7b-8			7b-9		
C	1.907806	-0.85845	-0.20226	1.901616	-0.87317	-0.21534	1.549694	-0.87841	-0.01714
C	1.426123	-2.01341	0.712519	1.401254	-2.00793	0.716959	0.78871	-1.80809	0.974084
C	-0.0375	-2.23004	0.273375	-0.06939	-2.19604	0.284565	-0.22186	-2.61971	0.101998
C	0.047613	-2.09434	-1.24664	0.017079	-2.09112	-1.23819	-0.43034	-1.72393	-1.12631
C	1.027481	-0.93944	-1.47981	1.030092	-0.96973	-1.49591	0.956476	-1.15634	-1.41297
C	-0.64477	-3.55434	0.724543	-0.70833	-3.49628	0.761317	0.375335	-3.96786	-0.2929
C	3.413179	-0.78023	-0.52046	3.414398	-0.79814	-0.52957	3.089268	-0.96379	0.046468
C	3.756716	0.447723	-1.39867	3.758002	0.415625	-1.41825	3.772424	-0.0636	-0.99778
C	3.64131	1.849413	-0.76682	3.653287	1.819603	-0.79231	3.727965	1.456292	-0.77282
C	2.219224	2.24596	-0.48381	2.236411	2.223898	-0.49425	2.400687	2.116865	-1.02532
C	1.676663	2.580693	0.690768	1.706515	2.562225	0.68513	1.64948	2.81817	-0.16626
C	0.186755	2.737969	0.861368	0.218467	2.723258	0.868875	0.42647	3.533898	-0.67695
O	3.71965	-1.96111	-1.28694	3.815594	-1.92329	-1.33701	3.536182	-2.28404	-0.31972
C	4.284868	-0.77342	0.738639	4.279929	-0.77462	0.733533	3.599273	-0.64221	1.455089
C	2.419352	2.713473	1.994924	2.461526	2.694879	1.982103	1.935583	3.01207	1.299883
O	-0.50452	2.287605	-0.31277	-0.48281	2.285478	-0.30361	-0.72205	3.336206	0.184034
H	1.659506	0.064842	0.31977	1.649643	0.054963	0.295808	1.288618	0.149838	0.249521
C	1.548936	-1.74928	2.213953	1.541273	-1.73216	2.214596	0.122216	-0.99574	2.084604
O	-0.79286	-1.13832	0.816722	-0.79335	-1.0762	0.810258	-1.43924	-2.93617	0.771696

C	-1.78054	1.918259	-0.17252	-1.75726	1.914163	-0.15802	-1.58209	2.360335	-0.12267
C	-2.31556	1.325615	-1.4543	-2.30048	1.335771	-1.4429	-2.72585	2.31438	0.863867
O	-2.41857	2.040722	0.855723	-2.38926	2.02676	0.875062	-1.46417	1.614081	-1.07619
C	-3.81241	1.069322	-1.40847	-3.79938	1.091906	-1.39695	-3.9393	1.578793	0.312508
C	-4.20606	-0.03864	-0.46334	-4.20401	-0.02135	-0.46295	-3.72397	0.091836	0.191219
O	-5.46882	0.077866	-0.06037	-5.47295	0.090223	-0.07895	-4.3634	-0.43189	-0.85032
O	-3.48803	-0.96924	-0.13309	-3.48921	-0.95213	-0.12566	-3.07769	-0.56859	0.989379
C	-5.96292	-0.96351	0.800841	-5.97799	-0.95664	0.769302	-4.29897	-1.8626	-0.99472
H	1.973904	-2.9292	0.458651	1.910763	-2.95375	0.481776	1.464831	-2.52092	1.46138
H	-0.93863	-1.93333	-1.69559	-0.96541	-1.9105	-1.68717	-1.10911	-0.90344	-0.86745
H	0.448001	-3.02388	-1.66514	0.387696	-3.04027	-1.64033	-0.86415	-2.27048	-1.96951
H	1.622978	-1.11794	-2.37656	1.632356	-1.19267	-2.37854	1.56792	-1.88774	-1.95034
H	0.498909	0.0065	-1.62352	0.529247	-0.01498	-1.6756	0.912342	-0.25109	-2.02276
H	-0.70494	-3.60606	1.814826	-0.76905	-3.52557	1.852328	0.540753	-4.58034	0.598548
H	-1.6598	-3.65913	0.327082	-1.72583	-3.58237	0.366158	-0.30633	-4.50221	-0.96081
H	-0.05158	-4.40118	0.367822	-0.13756	-4.36471	0.420269	1.332709	-3.8448	-0.80465
H	4.797376	0.32217	-1.7233	4.792823	0.270836	-1.74661	4.825625	-0.3628	-1.01268
H	3.149643	0.413757	-2.30831	3.142537	0.381314	-2.3225	3.372871	-0.30128	-1.98868
H	4.26672	1.909732	0.12641	4.290239	1.884258	0.092365	4.112365	1.697232	0.219881
H	4.067382	2.55576	-1.48873	4.072885	2.519648	-1.52402	4.43995	1.894016	-1.482
H	1.549669	2.150262	-1.33583	1.557242	2.129742	-1.33884	2.04032	2.027916	-2.05077
H	-0.14271	2.140527	1.716526	-0.10628	2.119199	1.72118	0.582832	4.614356	-0.6451
H	-0.09323	3.777943	1.05789	-0.05635	3.762523	1.076349	0.173673	3.236366	-1.69427
H	4.667601	-1.9536	-1.47668	3.636057	-2.72858	-0.83341	3.210283	-2.90667	0.344105
H	3.951177	-0.02276	1.459778	3.931249	-0.02625	1.450083	3.215774	0.316978	1.811223
H	4.26004	-1.75226	1.220964	4.267941	-1.74751	1.232032	3.280745	-1.40446	2.172291
H	5.326796	-0.5528	0.479905	5.318122	-0.54884	0.472044	4.692533	-0.60592	1.465181
H	3.494647	2.82445	1.857322	3.536722	2.793638	1.834639	2.944573	2.707872	1.577768
H	2.064806	3.582684	2.558117	2.120924	3.571138	2.543055	1.807582	4.061042	1.584754
H	2.244061	1.832326	2.623418	2.282535	1.819541	2.617669	1.23106	2.429937	1.903468
H	1.043308	-2.52936	2.791461	1.008384	-2.48554	2.802369	-0.40829	-1.63791	2.792387
H	2.587606	-1.72735	2.545016	2.581417	-1.74648	2.541048	0.877482	-0.42866	2.63712
H	1.090369	-0.7916	2.474022	1.12112	-0.75446	2.464045	-0.59616	-0.27842	1.673567
H	-1.67896	-1.15225	0.41606	-1.68744	-1.08797	0.426787	-1.95541	-2.11964	0.887097
H	-2.07236	2.002121	-2.27596	-2.05371	2.016765	-2.25975	-2.36066	1.82826	1.773055
H	-1.7647	0.400131	-1.6425	-1.75683	0.407805	-1.63966	-2.99271	3.335634	1.138349
H	-4.36645	1.971581	-1.14872	-4.34499	1.996545	-1.1274	-4.77786	1.70934	1.002746
H	-4.1555	0.760731	-2.40054	-4.14727	0.797198	-2.39157	-4.24884	1.982509	-0.65132
H	-5.92365	-1.92568	0.290092	-5.92868	-1.91607	0.254274	-4.91792	-2.09334	-1.85768
H	-5.37307	-1.00534	1.716375	-5.40231	-1.00189	1.69365	-3.27082	-2.18042	-1.16713
H	-6.99142	-0.69189	1.022478	-7.0104	-0.68834	0.976287	-4.69088	-2.34973	-0.10146
	7b-7			7b-8			7b-9		
C	1.907806	-0.85845	-0.20226	1.901616	-0.87317	-0.21534	1.549694	-0.87841	-0.01714

C	1.426123	-2.01341	0.712519	1.401254	-2.00793	0.716959	0.78871	-1.80809	0.974084
C	-0.0375	-2.23004	0.273375	-0.06939	-2.19604	0.284565	-0.22186	-2.61971	0.101998
C	0.047613	-2.09434	-1.24664	0.017079	-2.09112	-1.23819	-0.43034	-1.72393	-1.12631
C	1.027481	-0.93944	-1.47981	1.030092	-0.96973	-1.49591	0.956476	-1.15634	-1.41297
C	-0.64477	-3.55434	0.724543	-0.70833	-3.49628	0.761317	0.375335	-3.96786	-0.2929
C	3.413179	-0.78023	-0.52046	3.414398	-0.79814	-0.52957	3.089268	-0.96379	0.046468
C	3.756716	0.447723	-1.39867	3.758002	0.415625	-1.41825	3.772424	-0.0636	-0.99778
C	3.64131	1.849413	-0.76682	3.653287	1.819603	-0.79231	3.727965	1.456292	-0.77282
C	2.219224	2.24596	-0.48381	2.236411	2.223898	-0.49425	2.400687	2.116865	-1.02532
C	1.676663	2.580693	0.690768	1.706515	2.562225	0.68513	1.64948	2.81817	-0.16626
C	0.186755	2.737969	0.861368	0.218467	2.723258	0.868875	0.42647	3.533898	-0.67695
O	3.71965	-1.96111	-1.28694	3.815594	-1.92329	-1.33701	3.536182	-2.28404	-0.31972
C	4.284868	-0.77342	0.738639	4.279929	-0.77462	0.733533	3.599273	-0.64221	1.455089
C	2.419352	2.713473	1.994924	2.461526	2.694879	1.982103	1.935583	3.01207	1.299883
O	-0.50452	2.287605	-0.31277	-0.48281	2.285478	-0.30361	-0.72205	3.336206	0.184034
H	1.659506	0.064842	0.31977	1.649643	0.054963	0.295808	1.288618	0.149838	0.249521
C	1.548936	-1.74928	2.213953	1.541273	-1.73216	2.214596	0.122216	-0.99574	2.084604
O	-0.79286	-1.13832	0.816722	-0.79335	-1.0762	0.810258	-1.43924	-2.93617	0.771696
C	-1.78054	1.918259	-0.17252	-1.75726	1.914163	-0.15802	-1.58209	2.360335	-0.12267
C	-2.31556	1.325615	-1.4543	-2.30048	1.335771	-1.4429	-2.72585	2.31438	0.863867
O	-2.41857	2.040722	0.855723	-2.38926	2.02676	0.875062	-1.46417	1.614081	-1.07619
C	-3.81241	1.069322	-1.40847	-3.79938	1.091906	-1.39695	-3.9393	1.578793	0.312508
C	-4.20606	-0.03864	-0.46334	-4.20401	-0.02135	-0.46295	-3.72397	0.091836	0.191219
O	-5.46882	0.077866	-0.06037	-5.47295	0.090223	-0.07895	-4.3634	-0.43189	-0.85032
O	-3.48803	-0.96924	-0.13309	-3.48921	-0.95213	-0.12566	-3.07769	-0.56859	0.989379
C	-5.96292	-0.96351	0.800841	-5.97799	-0.95664	0.769302	-4.29897	-1.8626	-0.99472
H	1.973904	-2.9292	0.458651	1.910763	-2.95375	0.481776	1.464831	-2.52092	1.46138
H	-0.93863	-1.93333	-1.69559	-0.96541	-1.9105	-1.68717	-1.10911	-0.90344	-0.86745
H	0.448001	-3.02388	-1.66514	0.387696	-3.04027	-1.64033	-0.86415	-2.27048	-1.96951
H	1.622978	-1.11794	-2.37656	1.632356	-1.19267	-2.37854	1.56792	-1.88774	-1.95034
H	0.498909	0.0065	-1.62352	0.529247	-0.01498	-1.6756	0.912342	-0.25109	-2.02276
H	-0.70494	-3.60606	1.814826	-0.76905	-3.52557	1.852328	0.540753	-4.58034	0.598548
H	-1.6598	-3.65913	0.327082	-1.72583	-3.58237	0.366158	-0.30633	-4.50221	-0.96081
H	-0.05158	-4.40118	0.367822	-0.13756	-4.36471	0.420269	1.332709	-3.8448	-0.80465
H	4.797376	0.32217	-1.7233	4.792823	0.270836	-1.74661	4.825625	-0.3628	-1.01268
H	3.149643	0.413757	-2.30831	3.142537	0.381314	-2.3225	3.372871	-0.30128	-1.98868
H	4.26672	1.909732	0.12641	4.290239	1.884258	0.092365	4.112365	1.697232	0.219881
H	4.067382	2.55576	-1.48873	4.072885	2.519648	-1.52402	4.43995	1.894016	-1.482
H	1.549669	2.150262	-1.33583	1.557242	2.129742	-1.33884	2.04032	2.027916	-2.05077
H	-0.14271	2.140527	1.716526	-0.10628	2.119199	1.72118	0.582832	4.614356	-0.6451
H	-0.09323	3.777943	1.05789	-0.05635	3.762523	1.076349	0.173673	3.236366	-1.69427
H	4.667601	-1.9536	-1.47668	3.636057	-2.72858	-0.83341	3.210283	-2.90667	0.344105
H	3.951177	-0.02276	1.459778	3.931249	-0.02625	1.450083	3.215774	0.316978	1.811223

H	4.26004	-1.75226	1.220964	4.267941	-1.74751	1.232032	3.280745	-1.40446	2.172291
H	5.326796	-0.5528	0.479905	5.318122	-0.54884	0.472044	4.692533	-0.60592	1.465181
H	3.494647	2.82445	1.857322	3.536722	2.793638	1.834639	2.944573	2.707872	1.577768
H	2.064806	3.582684	2.558117	2.120924	3.571138	2.543055	1.807582	4.061042	1.584754
H	2.244061	1.832326	2.623418	2.282535	1.819541	2.617669	1.23106	2.429937	1.903468
H	1.043308	-2.52936	2.791461	1.008384	-2.48554	2.802369	-0.40829	-1.63791	2.792387
H	2.587606	-1.72735	2.545016	2.581417	-1.74648	2.541048	0.877482	-0.42866	2.63712
H	1.090369	-0.7916	2.474022	1.12112	-0.75446	2.464045	-0.59616	-0.27842	1.673567
H	-1.67896	-1.15225	0.41606	-1.68744	-1.08797	0.426787	-1.95541	-2.11964	0.887097
H	-2.07236	2.002121	-2.27596	-2.05371	2.016765	-2.25975	-2.36066	1.82826	1.773055
H	-1.7647	0.400131	-1.6425	-1.75683	0.407805	-1.63966	-2.99271	3.335634	1.138349
H	-4.36645	1.971581	-1.14872	-4.34499	1.996545	-1.1274	-4.77786	1.70934	1.002746
H	-4.1555	0.760731	-2.40054	-4.14727	0.797198	-2.39157	-4.24884	1.982509	-0.65132
H	-5.92365	-1.92568	0.290092	-5.92868	-1.91607	0.254274	-4.91792	-2.09334	-1.85768
H	-5.37307	-1.00534	1.716375	-5.40231	-1.00189	1.69365	-3.27082	-2.18042	-1.16713
H	-6.99142	-0.69189	1.022478	-7.0104	-0.68834	0.976287	-4.69088	-2.34973	-0.10146
	7b-10			7b-11			7b-12		
C	1.804225	-0.43823	-0.06388	1.768985	-0.88035	-0.16387	1.679143	-0.63141	-0.23443
C	1.167069	-1.81635	0.276772	1.086714	-1.82116	0.874423	1.742324	-1.66542	0.923474
C	0.884708	-2.49849	-1.08597	0.041485	-2.64133	0.074199	0.744973	-2.79671	0.559955
C	2.039476	-1.99013	-1.95303	0.716586	-2.78809	-1.29103	0.785131	-2.79918	-0.97072
C	2.186877	-0.51645	-1.56698	1.332648	-1.41317	-1.55479	0.849141	-1.31779	-1.35466
C	0.819313	-4.01584	-0.99889	-0.31183	-3.97099	0.722492	1.122222	-4.13726	1.174229
C	2.979525	-0.03714	0.848737	3.305056	-0.75111	-0.01809	3.070324	-0.13472	-0.70808
C	3.540603	1.363726	0.521619	3.914023	0.248212	-1.01937	2.973908	0.978656	-1.77678
C	2.641733	2.581852	0.790898	3.653211	1.744539	-0.79036	2.642417	2.405438	-1.30273
C	1.5435	2.782415	-0.21667	2.242802	2.189442	-1.05864	1.268051	2.553496	-0.71452
C	0.240208	2.996471	0.000022	1.425751	2.884769	-0.25808	0.933404	2.988801	0.505119
C	-0.64046	3.319447	-1.1822	0.08036	3.317024	-0.78296	-0.49784	2.994817	0.97753
O	4.025541	-0.99297	0.589884	3.944829	-1.99408	-0.36903	3.805636	-1.23484	-1.28147
C	2.599405	-0.10969	2.331837	3.710682	-0.37085	1.409253	3.937796	0.33875	0.455344
C	-0.44646	2.998636	1.338926	1.727048	3.311899	1.154666	1.886783	3.440871	1.580366
O	-1.94294	2.682327	-1.13575	-0.98615	3.032046	0.158555	-1.37986	2.62978	-0.08982
H	1.039739	0.332531	0.060987	1.358915	0.122198	-0.02423	1.136985	0.250105	0.116094
C	-0.06539	-1.7302	1.17278	0.503674	-1.10305	2.089833	1.49632	-1.06311	2.304171
O	-0.37402	-2.09331	-1.64171	-1.19976	-1.93869	-0.06108	-0.57762	-2.52275	1.035604
C	-1.99144	1.370484	-1.34822	-1.44454	1.78329	0.213376	-2.61821	2.252217	0.23844
C	-3.40576	0.844358	-1.38568	-2.4367	1.615176	1.338444	-3.42767	1.886359	-0.98395
O	-0.99861	0.689007	-1.54288	-1.07561	0.89749	-0.54061	-3.0451	2.237721	1.377487
C	-3.54306	-0.54364	-0.77748	-3.24318	0.330971	1.259131	-4.32378	0.679695	-0.74501
C	-3.35668	-0.53394	0.720039	-4.13056	0.273697	0.039397	-3.53131	-0.57206	-0.46138
O	-3.53717	-1.74809	1.245304	-4.69543	-0.9304	-0.09555	-4.28659	-1.66471	-0.50844
O	-3.07001	0.448862	1.378946	-4.33169	1.199155	-0.72487	-2.33732	-0.58222	-0.20611

C	-3.34838	-1.85783	2.665538	-5.57411	-1.09662	-1.21983	-3.62995	-2.91486	-0.22765
H	1.925608	-2.43353	0.771031	1.814757	-2.55744	1.241279	2.734545	-2.12995	0.91057
H	1.842165	-2.15003	-3.01673	0.005537	-3.10883	-2.05691	-0.07319	-3.32731	-1.39598
H	2.952027	-2.53426	-1.68839	1.508101	-3.5428	-1.2241	1.694245	-3.31271	-1.30088
H	3.196517	-0.1557	-1.76749	2.161313	-1.48242	-2.26027	1.257469	-1.18109	-2.36014
H	1.503	0.106434	-2.15124	0.593441	-0.7368	-1.99493	-0.15526	-0.88637	-1.38693
H	0.003558	-4.33372	-0.34306	-0.7582	-3.81752	1.709085	1.092039	-4.08377	2.266527
H	0.643241	-4.44293	-1.99046	-1.0342	-4.51004	0.103084	0.419835	-4.91121	0.852043
H	1.75613	-4.42189	-0.60967	0.577792	-4.59514	0.836718	2.129247	-4.43367	0.870944
H	4.447563	1.486492	1.126855	4.996984	0.091265	-0.98261	3.952194	1.01556	-2.26771
H	3.870895	1.382335	-0.52129	3.603205	-0.01924	-2.03356	2.252579	0.686693	-2.54857
H	2.254931	2.549929	1.811209	3.985783	2.036599	0.207142	3.417017	2.755953	-0.61837
H	3.289422	3.465584	0.744764	4.302627	2.281817	-1.4917	2.706189	3.049909	-2.18726
H	1.883191	2.766666	-1.25346	1.862859	1.909698	-2.04223	0.459982	2.230893	-1.36833
H	-0.89588	4.380543	-1.18771	0.035872	4.402807	-0.88339	-0.61964	2.286422	1.802895
H	-0.1546	3.06289	-2.12484	-0.15121	2.855319	-1.7429	-0.78518	3.9841	1.34828
H	4.759774	-0.79895	1.187996	3.645104	-2.67402	0.249169	3.369908	-1.49014	-2.1058
H	1.694375	0.464236	2.546695	3.181581	0.522092	1.752472	3.417004	1.087824	1.054725
H	2.427003	-1.14502	2.632677	3.487673	-1.17842	2.112326	4.203609	-0.49575	1.10742
H	3.407767	0.291225	2.953425	4.786094	-0.17582	1.454843	4.864754	0.780622	0.077715
H	0.253472	2.895291	2.167975	2.76785	3.148371	1.432742	2.917427	3.50258	1.23254
H	-1.01097	3.925895	1.483399	1.500944	4.373645	1.295044	1.603665	4.427943	1.960015
H	-1.16594	2.175416	1.399854	1.101056	2.757692	1.862083	1.858281	2.751225	2.431636
H	-0.56637	-2.69913	1.261761	-0.04109	-1.79595	2.737917	1.499577	-1.83392	3.081278
H	0.191968	-1.39461	2.179711	1.286899	-0.63307	2.688341	2.266586	-0.33105	2.558972
H	-0.78484	-1.0161	0.764235	-0.19096	-0.31758	1.781651	0.526844	-0.55819	2.33889
H	-0.46037	-1.12471	-1.60275	-1.03737	-1.01936	-0.33689	-0.98775	-1.80789	0.520241
H	-4.0725	1.558139	-0.90327	-1.86093	1.631516	2.26911	-4.05208	2.751239	-1.22375
H	-3.68375	0.799493	-2.44275	-3.08334	2.49379	1.360297	-2.75792	1.720534	-1.82749
H	-4.53188	-0.95395	-0.99426	-2.6041	-0.55497	1.263086	-4.9854	0.848911	0.108634
H	-2.81706	-1.24217	-1.20263	-3.89006	0.251852	2.137316	-4.966	0.500423	-1.60872
H	-3.49734	-2.90901	2.898433	-5.92859	-2.12251	-1.16289	-2.81711	-3.08026	-0.93521
H	-2.33872	-1.54741	2.936528	-6.41127	-0.40101	-1.15439	-3.23544	-2.91548	0.788071
H	-4.07869	-1.24341	3.193045	-5.03119	-0.93383	-2.15123	-4.39732	-3.67559	-0.3428

Table S 57. Energy analysis for 2*R*, 3*S*, 6*S*, 7*S*-7

Conf.	Steric Energy (kJ/mol)	Relative Energy (kJ/mol)	Distribution (%)
7b-1	-1233.291517	0.000936	8.00%
7b-2	-1233.291149	0.001304	5.42%
7b-3	-1233.291508	0.000945	7.93%
7b-4	-1233.291304	0.001149	6.39%
7b-5	-1233.291733	0.00072	10.06%
7b-6	-1233.291307	0.001146	6.41%

7b-7	-1233.290409	0.002044	2.48%
7b-8	-1233.289778	0.002675	1.27%
7b-9	-1233.291528	0.000925	8.10%
7b-10	-1233.292392	0.000061	20.21%
7b-11	-1233.292453	0	21.56%
7b-12	-1233.28993	0.002523	1.49%

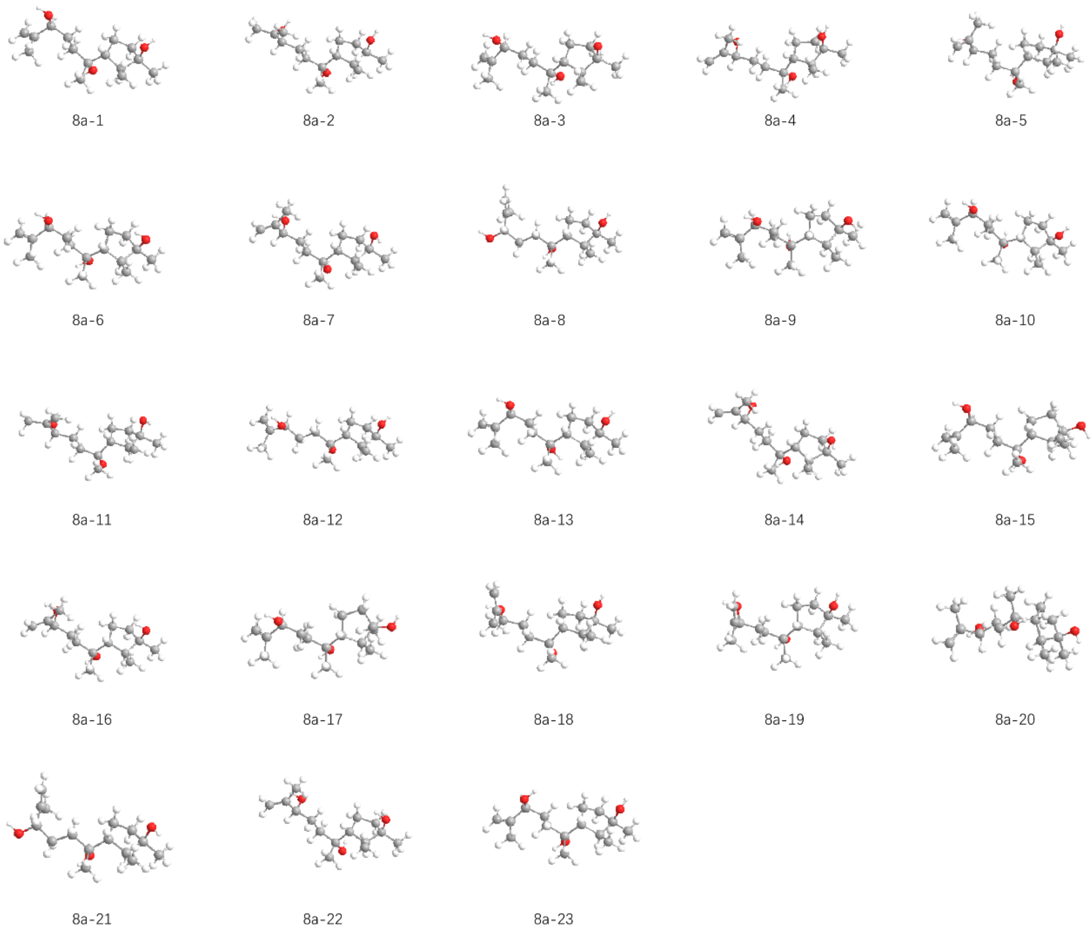

Figure S 109. Most stable conformers of 2*S*, 3*R*, 6*R*, 7*R, 10S*-8 at the CAM-B3LYP/DGDZVP level

Table S 58. Optimized Z-Matrixes of 2*S*, 3*R*, 6*R*, 7*R*, 10*S*-8 conformations in the methanol at CAM-B3LYP/DGDZVP level

	8a-1			8a-2			8a-3		
C	-1.12996	-0.1004	0.104643	-1.10811	0.023611	0.10292	-1.12955	-0.10439	0.104134
C	-2.49334	0.546252	0.482839	-2.55256	0.098587	0.676406	-2.4948	0.537741	0.480276
C	-3.57146	-0.40309	-0.0775	-3.42893	-0.71502	-0.29717	-3.57449	-0.41138	-0.09641
C	-2.91211	-0.9461	-1.34564	-2.75112	-0.46688	-1.64503	-2.90678	-0.95411	-1.35419
C	-1.46717	-1.2134	-0.92368	-1.2583	-0.55846	-1.3283	-1.4607	-1.21395	-0.92985
C	-0.07492	0.900444	-0.40942	-0.34273	1.363181	0.140059	-0.07463	0.901751	-0.39941
C	1.266118	0.211577	-0.73695	1.089714	1.23033	-0.41726	1.268329	0.217917	-0.72902
C	1.879497	-0.62517	0.384814	1.981269	0.189427	0.259647	1.88372	-0.62125	0.389763
C	3.293114	-1.09735	0.057292	3.407124	0.213598	-0.28748	3.295588	-1.09529	0.057192
C	4.279405	0.035582	-0.15966	4.38947	-0.72388	0.399088	4.282393	0.03633	-0.16415
O	-0.60118	1.447986	-1.6314	-1.06772	2.261292	-0.71981	-0.59905	1.457957	-1.61805

C	-4.91682	0.270803	-0.32509	-4.90283	-0.32525	-0.26967	-4.91244	0.27185	-0.35748
C	5.008709	0.088321	-1.27864	4.025994	-1.63195	1.310531	5.006121	0.088566	-1.28675
C	4.395413	1.060624	0.937431	5.822411	-0.5631	-0.04034	4.405141	1.060816	0.932733
H	-0.71071	-0.55613	1.006649	-0.53755	-0.67681	0.720092	-0.71204	-0.56393	1.005102
C	-2.66044	0.84424	1.971704	-2.67951	-0.35329	2.130133	-2.66681	0.818978	1.972237
O	-3.7326	-1.46867	0.876415	-3.2888	-2.10082	0.065182	-3.77376	-1.55061	0.759293
C	0.158894	2.046849	0.581375	-0.30436	1.95892	1.552057	0.154153	2.04012	0.601927
O	3.706564	-1.91801	1.15654	3.42496	-0.02276	-1.70212	3.712062	-1.91595	1.155262
H	-2.59702	1.480823	-0.07933	-2.89982	1.134393	0.595633	-2.59596	1.480613	-0.06831
H	-2.94118	-0.17512	-2.12195	-3.08137	-1.17655	-2.41114	-3.42119	-1.84213	-1.73144
H	-3.42734	-1.83147	-1.73334	-3.00166	0.540621	-1.99163	-2.93749	-0.18339	-2.12998
H	-0.79559	-1.21499	-1.78289	-0.66073	-0.02624	-2.0691	-0.7878	-1.20755	-1.78816
H	-1.38976	-2.19402	-0.44767	-0.93624	-1.60279	-1.33888	-1.3782	-2.19684	-0.45956
H	1.131908	-0.41806	-1.62161	1.032497	1.015509	-1.48649	1.136292	-0.40864	-1.61617
H	1.972355	0.997859	-1.03363	1.568612	2.215087	-0.32968	1.972368	1.007436	-1.02229
H	1.904857	-0.06871	1.327277	2.021431	0.368842	1.338413	1.912962	-0.06592	1.332798
H	1.274396	-1.51918	0.562652	1.562383	-0.81289	0.115518	1.277898	-1.51464	0.568243
H	3.257935	-1.70479	-0.85696	3.798257	1.232403	-0.18635	3.256132	-1.70311	-0.85662
H	0.040738	2.087893	-1.96778	-0.61244	3.11397	-0.70624	0.043603	2.099663	-1.94955
H	-5.63313	-0.44462	-0.74258	-5.47078	-0.92217	-0.99097	-5.61596	-0.43771	-0.80158
H	-5.33327	0.660013	0.608079	-5.33093	-0.49641	0.721824	-5.35336	0.649587	0.571427
H	-4.81522	1.098284	-1.03243	-5.03312	0.729211	-0.52742	-4.79299	1.116732	-1.04065
H	5.733091	0.880414	-1.45091	4.761676	-2.2885	1.767911	5.730506	0.879897	-1.46247
H	4.900331	-0.65919	-2.06079	2.998549	-1.75395	1.63835	4.892941	-0.65858	-2.06858
H	3.489563	1.674242	0.992022	5.914598	-0.71218	-1.11947	3.499893	1.674811	0.992893
H	4.513528	0.575363	1.910297	6.176184	0.451022	0.173509	4.528658	0.575042	1.904669
H	5.244317	1.726724	0.772683	6.480049	-1.27138	0.467206	5.253368	1.726626	0.763387
H	-3.65034	1.259895	2.184217	-3.72064	-0.3209	2.46608	-3.66861	1.201203	2.196435
H	-1.91923	1.568408	2.316744	-2.0993	0.288589	2.79663	-1.95196	1.565654	2.323307
H	-2.54352	-0.06627	2.563952	-2.31916	-1.37776	2.249611	-2.50364	-0.08643	2.565399
H	-4.27604	-2.14982	0.45824	-3.70748	-2.62395	-0.63146	-4.19406	-1.24266	1.573197
H	0.448884	1.679413	1.569109	0.116595	1.258242	2.277618	0.437907	1.664506	1.588421
H	0.958281	2.702346	0.218784	0.311429	2.864798	1.56399	0.956024	2.698111	0.249583
H	-0.74287	2.652405	0.691645	-1.30808	2.233374	1.882723	-0.74786	2.645536	0.711244
H	4.593142	-2.24727	0.956034	3.104301	-0.9242	-1.84584	4.597026	-2.24734	0.95116
	8a-4			8a-5			8a-6		
C	-1.07325	0.052483	0.084391	-1.05839	0.054328	0.116433	-1.13164	-0.10608	0.105421
C	-2.49535	0.184087	0.701913	-2.49419	0.19797	0.696249	-2.49659	0.535096	0.492127
C	-3.40416	-0.71857	-0.15635	-3.38505	-0.73032	-0.15413	-3.58146	-0.40613	-0.0899
C	-2.78103	-0.59477	-1.54694	-2.72519	-0.65701	-1.53205	-2.9152	-0.93455	-1.36156
C	-1.27652	-0.65545	-1.28238	-1.23026	-0.72254	-1.21748	-1.46769	-1.20338	-0.94267
C	-0.30569	1.386412	-0.02933	-0.3097	1.401826	-0.04553	-0.07619	0.9019	-0.39437
C	1.109981	1.194145	-0.61148	1.111886	1.214673	-0.61426	1.26547	0.218218	-0.72964

C	2.034056	0.250803	0.15803	2.017265	0.246969	0.147091	1.883048	-0.62562	0.384456
C	3.430329	0.181838	-0.45386	3.431185	0.207006	-0.42513	3.296121	-1.09428	0.049185
C	4.408104	-0.60231	0.401857	4.384244	-0.61619	0.421623	4.279974	0.041122	-0.16578
O	-1.05385	2.199241	-0.95194	-1.03009	2.270951	-0.93913	-0.60402	1.462777	-1.60862
C	-4.8787	-0.33346	-0.10661	-4.85814	-0.33747	-0.15652	-4.91418	0.281046	-0.33875
C	5.541615	-0.03078	0.821036	5.501393	-0.06332	0.904833	5.003598	0.101247	-1.28805
C	4.044746	-2.02654	0.731236	4.016333	-2.05593	0.668503	4.400063	1.05994	0.936666
H	-0.4812	-0.59233	0.740582	-0.46967	-0.55271	0.810906	-0.70826	-0.57393	1.000154
C	-2.56749	-0.12643	2.196001	-2.59967	-0.06402	2.197368	-2.66629	0.807959	1.985008
O	-3.23968	-2.064	0.327089	-3.23901	-2.05913	0.376501	-3.87717	-1.49451	0.803746
C	-0.22559	2.11671	1.315966	-0.24907	2.173698	1.271634	0.154918	2.034752	0.612273
O	3.295087	-0.41852	-1.7475	3.333685	-0.33252	-1.74864	3.714153	-1.91999	1.142762
H	-2.84839	1.207751	0.53547	-2.84317	1.215776	0.488668	-2.59399	1.479628	-0.05391
H	-3.13835	-1.37203	-2.23105	-3.06747	-1.45504	-2.19888	-3.43253	-1.81453	-1.75311
H	-3.04958	0.376521	-1.97423	-2.98188	0.298821	-2.00142	-2.9362	-0.1531	-2.12717
H	-0.71205	-0.18955	-2.09061	-0.6222	-0.33424	-2.03834	-0.79714	-1.18754	-1.80197
H	-0.94866	-1.6956	-1.21335	-0.92748	-1.76259	-1.07809	-1.371	-2.19859	-0.4951
H	1.022643	0.846705	-1.64308	1.047677	0.880353	-1.65438	1.131948	-0.40439	-1.61937
H	1.581956	2.184952	-0.66045	1.580622	2.205189	-0.64274	1.968889	1.008826	-1.02112
H	2.138556	0.57405	1.198794	2.087866	0.532371	1.201802	1.911195	-0.07554	1.330555
H	1.61516	-0.76007	0.172955	1.603166	-0.76532	0.111918	1.279911	-1.52183	0.558256
H	3.816391	1.203947	-0.56755	3.81846	1.233397	-0.47988	3.258232	-1.69704	-0.86802
H	-0.60046	3.049539	-1.02917	-0.99851	1.885019	-1.82509	0.036011	2.108004	-1.93833
H	-5.46963	-0.99678	-0.74693	-5.43558	-1.01936	-0.78949	-5.62606	-0.42291	-0.77858
H	-5.26674	-0.41437	0.912515	-5.27244	-0.38266	0.854347	-5.33994	0.657331	0.595628
H	-5.027	0.69225	-0.45482	-4.99214	0.676581	-0.54246	-4.78886	1.121174	-1.02565
H	6.261722	-0.57175	1.429814	6.203478	-0.63203	1.50935	5.726004	0.895328	-1.45938
H	5.782982	0.999778	0.571813	5.746617	0.978825	0.714613	4.892378	-0.64194	-2.07391
H	3.194605	-2.06185	1.419802	3.146819	-2.12761	1.329494	3.49339	1.671488	1.00009
H	3.749736	-2.56837	-0.17162	3.749608	-2.55237	-0.26861	4.524707	0.569267	1.905983
H	4.879882	-2.55222	1.197974	4.839501	-2.60256	1.132403	5.246666	1.728712	0.770984
H	-3.5957	-0.0615	2.565329	-3.63521	0.017244	2.542014	-3.6601	1.210961	2.202268
H	-1.96536	0.576388	2.775971	-2.00639	0.655422	2.766064	-1.93061	1.531119	2.343419
H	-2.19978	-1.13485	2.400192	-2.24128	-1.06662	2.442159	-2.5436	-0.10796	2.569698
H	-3.67663	-2.65177	-0.30358	-3.67995	-2.66422	-0.23486	-3.05985	-1.97694	0.987109
H	0.213545	1.487696	2.094404	0.189133	1.567549	2.067566	0.443483	1.654248	1.59541
H	0.393046	3.015844	1.221859	0.35685	3.075747	1.149495	0.954602	2.695126	0.25966
H	-1.21884	2.427825	1.646063	-1.24838	2.4812	1.586949	-0.74726	2.638394	0.728959
H	4.169374	-0.42301	-2.15932	4.219314	-0.31868	-2.13525	4.600502	-2.24711	0.937777
	8a-7			8a-8			8a-9		
C	-1.0552	0.052882	0.118357	1.246964	0.135232	-0.47446	-1.12301	-0.10696	0.127126
C	-2.49409	0.187663	0.690236	2.680249	0.612463	-0.10944	-2.48769	0.551813	0.471741
C	-3.38315	-0.73538	-0.18019	3.498654	-0.67299	0.122995	-3.57142	-0.40503	-0.0848

C	-2.70969	-0.65973	-1.54573	2.464921	-1.61643	0.737143	-2.89435	-0.9999	-1.31421
C	-1.21573	-0.7148	-1.22224	1.202336	-1.37092	-0.08929	-1.46072	-1.26491	-0.85123
C	-0.31008	1.404329	-0.02532	0.10453	0.950762	0.173847	-0.07178	0.885817	-0.42912
C	1.113081	1.228712	-0.59376	-1.23802	0.502965	-0.44508	1.271593	0.196211	-0.74298
C	2.012456	0.238808	0.145986	-2.49078	1.033286	0.249705	1.885154	-0.61571	0.396752
C	3.433911	0.223396	-0.40835	-3.7748	0.478724	-0.36137	3.301812	-1.08754	0.081717
C	4.377513	-0.63055	0.418357	-3.86797	-1.03541	-0.31032	4.281696	0.04547	-0.16191
O	-1.03198	2.283284	-0.90765	0.138662	0.638546	1.576828	-0.54874	1.497971	-1.64089
C	-4.85244	-0.3283	-0.19786	4.735878	-0.4691	0.990643	-4.89764	0.283521	-0.38738
C	5.484068	-0.09368	0.942266	-4.09624	-1.73353	-1.42714	5.013376	0.073611	-1.28017
C	4.013065	-2.08106	0.598217	-3.7057	-1.68625	1.037966	4.38881	1.099145	0.908613
H	-0.46789	-0.55788	0.810877	1.113949	0.234556	-1.55737	-0.70216	-0.52693	1.045887
C	-2.60375	-0.09568	2.187349	3.313387	1.559655	-1.12702	-2.66862	0.883289	1.951955
O	-3.26614	-2.10872	0.230815	3.890733	-1.16281	-1.17201	-3.7942	-1.51217	0.805349
C	-0.25387	2.157643	1.302924	0.2932	2.45916	-0.01071	0.157785	2.057579	0.524656
O	3.35559	-0.26361	-1.75316	-4.85599	1.073474	0.366732	3.718541	-1.87887	1.200921
H	-2.84302	1.208817	0.499813	2.636269	1.113709	0.864361	-2.57694	1.477085	-0.10851
H	-3.0453	-1.46317	-2.20652	2.799237	-2.65939	0.726756	-3.41354	-1.89433	-1.6679
H	-2.96932	0.29519	-2.01406	2.295506	-1.33019	1.779958	-2.91024	-0.25738	-2.11859
H	-0.60684	-0.31469	-2.03699	0.305941	-1.63687	0.472784	-0.76877	-1.34809	-1.69359
H	-0.90451	-1.7533	-1.09076	1.217486	-1.98624	-0.99198	-1.41412	-2.21917	-0.32212
H	1.052047	0.92161	-1.64239	-1.24888	0.809865	-1.49768	1.150421	-0.46203	-1.61145
H	1.584404	2.218378	-0.59533	-1.27386	-0.59004	-0.4453	1.966376	0.981229	-1.06098
H	2.068611	0.488038	1.210733	-2.47846	0.776777	1.314401	1.906313	-0.03802	1.326454
H	1.601859	-0.77279	0.070756	-2.54897	2.123702	0.182441	1.284107	-1.50844	0.59366
H	3.819305	1.251913	-0.41729	-3.82867	0.792581	-1.41228	3.271119	-1.71734	-0.81749
H	-1.00025	1.908528	-1.79836	-0.39556	1.292621	2.045616	-0.60583	0.814653	-2.32244
H	-5.4186	-0.99302	-0.85601	5.25845	-1.4195	1.141617	-5.60394	-0.43324	-0.81503
H	-5.29263	-0.38674	0.803394	5.432644	0.22748	0.516493	-5.34481	0.698431	0.522331
H	-4.96895	0.697781	-0.55611	4.463713	-0.07416	1.973138	-4.75994	1.102606	-1.09793
H	6.179183	-0.68424	1.533741	-4.18088	-2.81725	-1.41504	5.733377	0.865348	-1.47131
H	5.727223	0.956552	0.799785	-4.20104	-1.24142	-2.39108	4.911239	-0.69439	-2.04308
H	3.133282	-2.18474	1.241132	-2.67354	-1.59181	1.392776	3.479634	1.709188	0.944307
H	3.764254	-2.53835	-0.3634	-4.34614	-1.20304	1.781061	4.506487	0.640018	1.894157
H	4.83142	-2.64318	1.052011	-3.95369	-2.74856	0.999533	5.234633	1.765335	0.729066
H	-3.64326	-0.05221	2.52986	4.335152	1.825743	-0.8391	-3.66764	1.28397	2.155076
H	-2.04124	0.634186	2.772754	2.74498	2.488007	-1.214	-1.94772	1.633376	2.282973
H	-2.20196	-1.08392	2.432268	3.352345	1.094209	-2.11468	-2.52022	-0.00372	2.575877
H	-3.6798	-2.1977	1.099696	4.259348	-2.04732	-1.04587	-4.22102	-1.17265	1.603148
H	0.185275	1.541555	2.090776	0.340976	2.721131	-1.07083	0.434877	1.7114	1.522811
H	0.349016	3.063427	1.194821	-0.54042	3.013959	0.430636	0.958981	2.697595	0.144711
H	-1.25462	2.457616	1.620914	1.207437	2.803095	0.476923	-0.74328	2.668209	0.612317
H	4.245974	-0.23088	-2.12754	-5.68027	0.748977	-0.02042	4.607785	-2.20667	1.010023

	8a-13			8a-14			8a-15		
C	-1.12307	-0.09471	0.081764	-1.06722	0.054552	0.094619	-1.14559	-0.06775	0.281224
C	-2.48392	0.534589	0.49909	-2.49788	0.181718	0.691159	-2.56704	0.511985	0.544745
C	-3.56525	-0.40879	-0.06686	-3.39614	-0.72941	-0.18195	-3.54879	-0.42747	-0.22636
C	-2.92516	-0.90679	-1.36294	-2.74726	-0.62209	-1.55663	-2.77049	-1.73632	-0.36181
C	-1.47028	-1.17461	-0.97688	-1.2464	-0.66785	-1.26765	-1.34149	-1.2851	-0.64494
C	-0.06407	0.917617	-0.42077	-0.30768	1.393171	-0.01686	-0.11257	0.952093	-0.22631
C	1.271376	0.232011	-0.75609	1.111299	1.21095	-0.59412	1.232624	0.281343	-0.56246
C	1.864703	-0.63978	0.348605	2.025679	0.243319	0.156508	1.934102	-0.43089	0.592443
C	3.283914	-1.10011	0.028955	3.432363	0.201124	-0.4334	3.260617	-1.05717	0.171286
C	4.272618	0.039198	-0.13779	4.396039	-0.61884	0.404476	4.266053	-0.05344	-0.36208
O	-0.47188	1.492231	-1.67575	-1.05809	2.202564	-0.94041	-0.64683	1.495892	-1.44575
C	-4.92226	0.25695	-0.26743	-4.86829	-0.33238	-0.1654	-3.89656	0.168233	-1.59142
C	5.02668	0.120444	-1.23848	5.518317	-0.06371	0.87302	4.809987	-0.22323	-1.57122
C	4.363172	1.036322	0.986992	4.032434	-2.05826	0.659215	4.61542	1.10611	0.533001
H	-0.70098	-0.57715	0.968532	-0.47861	-0.58016	0.763834	-0.76764	-0.43831	1.240654
C	-2.62528	0.798519	1.996917	-2.58575	-0.12962	2.184451	-2.86707	0.601866	2.040842
O	-3.69219	-1.50038	0.860473	-3.26375	-2.10968	0.202276	-4.75756	-0.71464	0.483431
C	0.171437	2.048889	0.585503	-0.23715	2.120999	1.330545	0.08415	2.08911	0.782016
O	3.681043	-1.95308	1.109667	3.319645	-0.34278	-1.75373	3.777701	-1.72039	1.330931
H	-2.62598	1.487803	-0.02735	-2.85189	1.205004	0.524938	-2.64255	1.516436	0.118393
H	-3.44127	-1.78298	-1.76914	-3.09098	-1.41478	-2.22685	-2.81864	-2.26666	0.595997
H	-2.97692	-0.11295	-2.11573	-3.01533	0.342118	-1.99866	-3.18635	-2.39202	-1.13187
H	-0.81591	-1.13521	-1.84849	-0.67254	-0.20628	-2.07186	-1.23769	-0.98935	-1.69269
H	-1.37403	-2.17096	-0.53899	-0.91217	-1.70484	-1.18405	-0.60757	-2.07239	-0.45401
H	1.140406	-0.37341	-1.65818	1.029391	0.890716	-1.63487	1.066712	-0.42716	-1.38079
H	1.978421	1.02379	-1.02754	1.587197	2.200799	-0.61476	1.8963	1.05851	-0.96388
H	1.875607	-0.11323	1.308573	2.11114	0.527906	1.210301	2.118247	0.255654	1.424752
H	1.259275	-1.54005	0.489779	1.609558	-0.76838	0.126366	1.30714	-1.23893	0.981695
H	3.264353	-1.67993	-0.90351	3.81902	1.227389	-0.49609	3.063543	-1.79902	-0.61412
H	-1.22425	2.076316	-1.51276	-0.60497	3.052598	-1.02191	-0.01772	2.151506	-1.77592
H	-5.63909	-0.45557	-0.68844	-5.44131	-0.9873	-0.82751	-4.50147	-0.53376	-2.17266
H	-5.32266	0.616741	0.684216	-5.29123	-0.41465	0.841679	-4.46483	1.096741	-1.46546
H	-4.84562	1.103586	-0.95503	-4.99777	0.699959	-0.5005	-2.99386	0.415075	-2.15494
H	5.753847	0.917024	-1.37452	6.228082	-0.63011	1.470709	5.541177	0.474857	-1.97099
H	4.936253	-0.60693	-2.04163	5.760456	0.978083	0.676869	4.53628	-1.0656	-2.20192
H	3.455406	1.647441	1.036931	3.169225	-2.12905	1.328494	3.7648	1.788632	0.634983
H	4.460739	0.526809	1.949673	3.757447	-2.55779	-0.27384	4.86851	0.75677	1.537795
H	5.214499	1.707125	0.857643	4.860652	-2.60249	1.116911	5.458631	1.674976	0.136947
H	-3.61366	1.202096	2.236557	-3.62087	-0.09931	2.541615	-3.8432	1.053908	2.236597
H	-1.88189	1.519283	2.343963	-2.02024	0.592069	2.776948	-2.10966	1.215312	2.538058
H	-2.49093	-0.12525	2.563807	-2.17489	-1.11983	2.405441	-2.85643	-0.3882	2.506779
H	-4.23759	-2.17698	0.437485	-3.66239	-2.21652	1.076138	-5.29091	0.091121	0.505589

H	0.46147	1.664141	1.566496	0.205823	1.493718	2.108386	0.380779	1.711559	1.763754
H	0.964369	2.709011	0.223125	0.373463	3.025864	1.240423	0.863616	2.776852	0.435954
H	-0.72909	2.654822	0.721802	-1.23378	2.422801	1.658968	-0.84006	2.658946	0.902434
H	4.571685	-2.27413	0.914055	4.200931	-0.33042	-2.15013	4.60619	-2.14853	1.076127
	8a-16			8a-17			8a-18		
C	-1.07406	0.055386	0.086879	-1.14633	-0.06809	0.284553	-1.0677	0.059551	0.084149
C	-2.50089	0.183017	0.697059	-2.56755	0.510834	0.555603	-2.48961	0.434154	0.589717
C	-3.40681	-0.73086	-0.1662	-3.54521	-0.41052	-0.22609	-3.40836	-0.72761	0.16015
C	-2.77442	-0.61038	-1.55359	-2.77377	-1.7295	-0.36351	-2.77606	-1.18565	-1.15513
C	-1.26792	-0.65031	-1.28464	-1.3417	-1.28421	-0.64399	-1.27528	-1.15297	-0.86401
C	-0.30784	1.390718	-0.02332	-0.11443	0.953913	-0.2206	-0.30644	1.237335	-0.57584
C	1.108964	1.201133	-0.6034	1.23048	0.284319	-0.56059	1.102135	0.826076	-1.0501
C	2.032519	0.255295	0.163618	1.932666	-0.4335	0.590425	2.011976	0.206501	0.01025
C	3.427939	0.186707	-0.45048	3.257868	-1.05966	0.165015	3.426506	-0.05171	-0.51128
C	4.405599	-0.60305	0.400119	4.263984	-0.05497	-0.36523	4.356829	-0.50057	0.603177
O	-1.05705	2.201025	-0.94608	-0.64962	1.502486	-1.43735	-1.03138	1.724244	-1.72057
C	-4.87632	-0.34442	-0.12826	-3.87697	0.19207	-1.59291	-4.87612	-0.33522	0.031542
C	5.540058	-0.03499	0.821357	4.80577	-0.21999	-1.57601	4.702464	-1.78192	0.759405
C	4.041215	-2.02884	0.721686	4.616471	1.099861	0.534695	4.864268	0.593842	1.50562
H	-0.48002	-0.58652	0.745465	-0.76596	-0.44093	1.241983	-0.4717	-0.25786	0.945319
C	-2.58111	-0.12269	2.19114	-2.86453	0.583493	2.053893	-2.56773	0.751847	2.081604
O	-3.362	-2.0978	0.281292	-4.758	-0.57427	0.518431	-3.26836	-1.76443	1.147175
C	-0.2321	2.117981	1.323586	0.083534	2.086772	0.792205	-0.21462	2.442906	0.358381
O	3.289318	-0.40756	-1.74647	3.775401	-1.72863	1.321225	3.310875	-1.00544	-1.56107
H	-2.85282	1.205969	0.524974	-2.64283	1.520603	0.143121	-2.83167	1.307258	0.022753
H	-3.12398	-1.39778	-2.2268	-2.8254	-2.26038	0.593742	-3.13904	-2.16962	-1.46958
H	-3.04465	0.35768	-1.98635	-3.18978	-2.38375	-1.13622	-3.03373	-0.47046	-1.94371
H	-0.71066	-0.1713	-2.08954	-1.23377	-0.98816	-1.69113	-0.68286	-1.10482	-1.78122
H	-0.90799	-1.68379	-1.23469	-0.6101	-2.07322	-0.45093	-0.97694	-2.07224	-0.35532
H	1.024208	0.85805	-1.63667	1.063826	-0.42037	-1.38204	1.014958	0.12414	-1.88504
H	1.580041	2.192447	-0.64759	1.894197	1.063078	-0.95881	1.580542	1.724348	-1.45736
H	2.138238	0.576001	1.205021	2.118617	0.249445	1.425283	2.075099	0.862467	0.883066
H	1.613274	-0.75554	0.176269	1.305343	-1.24245	0.977122	1.61198	-0.75228	0.356365
H	3.815413	1.208694	-0.56009	3.058829	-1.79787	-0.62334	3.82378	0.89098	-0.91864
H	-0.60679	3.053014	-1.02291	-0.02112	2.159962	-1.76503	-1.00586	1.042045	-2.40511
H	-5.45687	-1.00068	-0.78258	-4.49373	-0.49598	-2.18209	-5.47537	-1.19166	-0.2947
H	-5.27751	-0.43246	0.88526	-4.42917	1.126718	-1.45931	-5.27095	0.007139	0.991965
H	-5.00942	0.686121	-0.46534	-2.96974	0.414727	-2.15892	-5.004	0.46509	-0.70219
H	6.260207	-0.5801	1.426353	5.53726	0.478936	-1.97376	5.368289	-2.091	1.560364
H	5.782241	0.996669	0.577595	4.529898	-1.05907	-2.21013	4.32764	-2.55484	0.095027
H	3.192389	-2.06736	1.411727	3.766641	1.782535	0.642199	5.417369	1.342295	0.928915
H	3.744053	-2.56496	-0.18387	4.871767	0.745327	1.537113	4.041054	1.119068	1.999748
H	4.876628	-2.55819	1.183718	5.459173	1.669984	0.13938	5.524609	0.19778	2.279008

H	-3.6129	-0.06492	2.550793	-3.84588	1.020946	2.249591	-3.59603	0.968221	2.387373
H	-1.9877	0.58406	2.774921	-2.10928	1.199043	2.552212	-1.95975	1.623893	2.332193
H	-2.20948	-1.12801	2.407987	-2.84056	-0.41035	2.511746	-2.21021	-0.09182	2.676491
H	-2.44494	-2.40239	0.2575	-5.33936	-1.14168	-0.00519	-3.72905	-2.54566	0.812911
H	0.205734	1.487721	2.101755	0.382349	1.705141	1.771687	0.223993	2.16897	1.320365
H	0.386036	3.017622	1.232587	0.861909	2.77647	0.447573	0.404046	3.222169	-0.09519
H	-1.22644	2.427736	1.651497	-0.8407	2.655569	0.917102	-1.20451	2.865573	0.542847
H	4.162675	-0.41128	-2.16028	4.603548	-2.15585	1.063831	4.195687	-1.1636	-1.91668
	8a-19			8a-20			8a-21		
C	-1.07407	0.057511	0.074306	1.44365	0.895401	-0.38469	1.245428	0.133168	-0.46785
C	-2.49341	0.423221	0.596814	1.939672	-0.06812	0.713832	2.678597	0.606254	-0.10088
C	-3.41228	-0.73061	0.147179	2.988563	-0.93407	-0.01845	3.49843	-0.68776	0.124031
C	-2.78813	-1.15766	-1.1817	2.311985	-1.20255	-1.36106	2.466485	-1.63362	0.724814
C	-1.2844	-1.12477	-0.9091	1.544108	0.085036	-1.70824	1.196586	-1.37408	-0.08702
C	-0.30772	1.24215	-0.55069	0.100835	1.619224	-0.12315	0.103551	0.950604	0.17864
C	1.099271	0.833553	-1.03342	-1.01744	0.735701	0.446573	-1.23886	0.50362	-0.44125
C	2.017311	0.211321	0.018268	-1.43864	-0.47456	-0.38273	-2.49173	1.03217	0.254625
C	3.423947	-0.06057	-0.51787	-2.60639	-1.22661	0.250324	-3.77567	0.481899	-0.36056
C	4.362644	-0.5114	0.588827	-3.86108	-0.38735	0.404481	-3.87035	-1.03237	-0.3171
O	-1.0682	1.647333	-1.7037	0.293937	2.59758	0.919537	0.136518	0.639995	1.581796
C	-4.88193	-0.3387	0.03792	3.378893	-2.20568	0.726277	4.731284	-0.48978	0.999132
C	4.697941	-1.79499	0.749222	-4.4536	-0.27554	1.597388	-4.09694	-1.72477	-1.43782
C	4.890263	0.583534	1.478938	-4.39294	0.281595	-0.83545	-3.71143	-1.69001	1.028285
H	-0.47781	-0.2825	0.926358	2.191044	1.697117	-0.45487	1.114123	0.234105	-1.55089
C	-2.56204	0.705354	2.09653	2.437378	0.60313	1.990346	3.310568	1.555944	-1.11744
O	-3.26444	-1.78856	1.111595	4.166657	-0.16517	-0.31691	3.885399	-1.27892	-1.12922
C	-0.2089	2.429091	0.414542	-0.36452	2.365289	-1.3793	0.294689	2.458502	-0.00776
O	3.290283	-1.01843	-1.56166	-2.85841	-2.35715	-0.59238	-4.85703	1.074255	0.369189
H	-2.84003	1.309156	0.053817	1.128339	-0.75512	0.983984	2.63766	1.108072	0.872801
H	-3.1528	-2.13533	-1.51487	3.045524	-1.48734	-2.11922	2.804888	-2.67275	0.690484
H	-3.04694	-0.42351	-1.95117	1.619858	-2.04175	-1.24166	2.307041	-1.3601	1.772065
H	-0.7146	-1.01658	-1.83244	0.568225	-0.15652	-2.13027	0.304798	-1.63502	0.484852
H	-0.96714	-2.05888	-0.43887	2.071461	0.669865	-2.46485	1.195646	-1.98734	-0.99111
H	0.996309	0.144037	-1.87366	-0.71111	0.401346	1.443811	-1.24966	0.812621	-1.49324
H	1.584128	1.734179	-1.43403	-1.88323	1.388859	0.604349	-1.27452	-0.58939	-0.4436
H	2.096338	0.868319	0.88901	-1.72109	-0.18135	-1.39896	-2.4808	0.770581	1.318085
H	1.613285	-0.74335	0.37111	-0.61553	-1.18893	-0.4766	-2.54876	2.122965	0.192492
H	3.824507	0.877031	-0.93394	-2.29935	-1.57623	1.245192	-3.82803	0.800952	-1.40998
H	-0.60745	2.392552	-2.11234	1.005612	3.189658	0.640385	-0.39852	1.294243	2.04941
H	-5.48159	-1.18936	-0.30257	4.045973	-2.81219	0.1076	5.235237	-1.44694	1.158724
H	-5.27109	-0.01791	1.008178	3.901767	-1.9753	1.660159	5.446384	0.195	0.530824
H	-5.01662	0.47693	-0.67759	2.496102	-2.8018	0.972399	4.4569	-0.07507	1.972558
H	5.369627	-2.10549	1.544729	-5.36102	0.307954	1.731176	-4.18244	-2.80846	-1.43124

H	4.308379	-2.56846	0.094	-4.04535	-0.76156	2.480214	-4.19938	-1.22782	-2.39952
H	5.444532	1.322916	0.891742	-3.72362	1.085869	-1.15957	-2.67996	-1.59818	1.385768
H	4.077339	1.119832	1.978208	-4.46163	-0.43165	-1.66152	-4.35308	-1.21003	1.772423
H	5.554791	0.185879	2.247916	-5.38026	0.713715	-0.66248	-3.96011	-2.75193	0.98401
H	-3.58794	0.917302	2.413571	1.617643	1.111628	2.499996	4.34603	1.796279	-0.85348
H	-1.94986	1.569182	2.365013	3.196939	1.361411	1.770542	2.76711	2.500577	-1.17678
H	-2.2038	-0.1536	2.668796	2.868156	-0.1261	2.68393	3.304535	1.118222	-2.12076
H	-3.70582	-2.56959	0.751916	4.592505	0.064375	0.519931	4.538704	-0.6996	-1.5435
H	0.243177	2.141896	1.367332	-0.64166	1.692131	-2.19287	0.349592	2.718271	-1.06812
H	0.405823	3.223062	-0.02313	-1.23353	2.982388	-1.1351	-0.54103	3.015145	0.427136
H	-1.19778	2.845494	0.616739	0.431069	3.02134	-1.74866	1.206003	2.802249	0.485549
H	4.170886	-1.18831	-1.92228	-3.58307	-2.85942	-0.19589	-5.68122	0.752105	-0.02011
	8a-22			8a-23					
C	-1.05746	0.053828	0.122745	1.132571	0.101813	0.107418			
C	-2.4992	0.195619	0.691423	2.499601	-0.52973	0.498063			
C	-3.38845	-0.74074	-0.16566	3.571412	0.399572	-0.10579			
C	-2.71059	-0.68206	-1.53643	2.902474	0.890956	-1.38974			
C	-1.21557	-0.72946	-1.21099	1.459756	1.171842	-0.96902			
C	-0.31223	1.403869	-0.04019	0.072676	-0.91895	-0.35491			
C	1.110206	1.220706	-0.6075	-1.27291	-0.246	-0.69642			
C	2.015393	0.250547	0.150884	-1.89722	0.605578	0.407917			
C	3.429299	0.213363	-0.42169	-3.30054	1.106788	0.049036			
C	4.382128	-0.6153	0.419921	-4.28949	-0.01363	-0.19631			
O	-1.03621	2.26888	-0.9333	0.586947	-1.51305	-1.55994			
C	-4.85264	-0.33366	-0.19252	4.91715	-0.27963	-0.33575			
C	5.499054	-0.06549	0.907046	-4.93259	-0.10445	-1.36377			
C	4.014545	-2.05683	0.656736	-4.4858	-1.0078	0.917844			
H	-0.46763	-0.54519	0.824323	0.723314	0.594125	0.994755			
C	-2.61626	-0.06101	2.192223	2.680381	-0.7724	1.995328			
O	-3.37942	-2.08985	0.33277	3.734448	1.501799	0.805153			
C	-0.25586	2.174225	1.27805	-0.14964	-2.02565	0.682602			
O	3.330815	-0.31826	-1.74821	-3.83284	1.907205	1.111386			
H	-2.84436	1.213302	0.478225	2.599406	-1.48417	-0.03035			
H	-3.04526	-1.4929	-2.18837	3.412999	1.761497	-1.81532			
H	-2.96242	0.268151	-2.01893	2.92792	0.090203	-2.13551			
H	-0.60884	-0.33539	-2.02927	0.783153	1.135452	-1.82355			
H	-0.88318	-1.76453	-1.08171	1.382607	2.171424	-0.53416			
H	1.048917	0.891196	-1.64936	-1.14412	0.367589	-1.5932			
H	1.577572	2.211874	-0.63083	-1.97337	-1.04286	-0.97724			
H	2.085563	0.531983	1.206663	-1.95217	0.055933	1.352885			
H	1.602037	-0.76197	0.111438	-1.27009	1.485514	0.591673			
H	3.816849	1.239913	-0.47041	-3.23202	1.713717	-0.86352			
H	-0.9863	1.895547	-1.82378	-0.05671	-2.16732	-1.86381			

H	-5.42163	-1.01115	-0.8352	5.628525	0.421048	-0.78541			
H	-5.28585	-0.37109	0.810804	5.340913	-0.63145	0.608868			
H	-4.96042	0.682339	-0.57904	4.813384	-1.13406	-1.00994			
H	6.201146	-0.63817	1.507772	-5.63903	-0.90647	-1.56254			
H	5.744159	0.97793	0.723858	-4.7694	0.617909	-2.15949			
H	3.145327	-2.13346	1.317587	-3.58387	-1.61237	1.063203			
H	3.747751	-2.54671	-0.2838	-4.68642	-0.49731	1.863441			
H	4.837968	-2.60653	1.116502	-5.31401	-1.68547	0.702607			
H	-3.65686	0.004571	2.523769	3.671382	-1.18249	2.213389			
H	-2.03933	0.668033	2.765142	1.941086	-1.48133	2.374313			
H	-2.24616	-1.0563	2.453625	2.571894	0.16006	2.554026			
H	-2.46455	-2.39657	0.388771	4.276712	2.166182	0.359346			
H	0.180589	1.567704	2.074667	-0.4209	-1.61927	1.660259			
H	0.349531	3.076761	1.158128	-0.95842	-2.69006	0.3592			
H	-1.25629	2.480782	1.590576	0.751455	-2.63082	0.80041			
H	4.216032	-0.30188	-2.13567	-3.17969	2.587311	1.323865			

Table S 59. Energy analysis for 2*S*, 3*R*, 6*R*, 7*R*, *10S*-8

Conf.	Steric Energy (kJ/mol)	Relative Energy (kJ/mol)	Distribution (%)
8a-1	-813.603111	0.00031	7.91%
8a-2	-813.601598	0.001823	1.59%
8a-3	-813.603192	0.000229	8.62%
8a-4	-813.602405	0.001016	3.75%
8a-5	-813.603421	0	10.99%
8a-6	-813.602626	0.000795	4.74%
8a-7	-813.602752	0.000669	5.41%
8a-8	-813.601993	0.001428	2.42%
8a-9	-813.602742	0.000679	5.35%
8a-10	-813.60271	0.000711	5.18%
8a-11	-813.601259	0.002162	1.11%
8a-12	-813.601195	0.002226	1.04%
8a-13	-813.602982	0.000439	6.90%
8a-14	-813.602791	0.00063	5.64%
8a-15	-813.602199	0.001222	3.01%
8a-16	-813.602497	0.000924	4.13%
8a-17	-813.601767	0.001654	1.91%
8a-18	-813.60236	0.001061	3.57%
8a-19	-813.601961	0.00146	2.34%
8a-20	-813.601376	0.002045	1.26%
8a-21	-813.602053	0.001368	2.58%
8a-22	-813.602614	0.000807	4.68%
8a-23	-813.602827	0.000594	5.86%

y

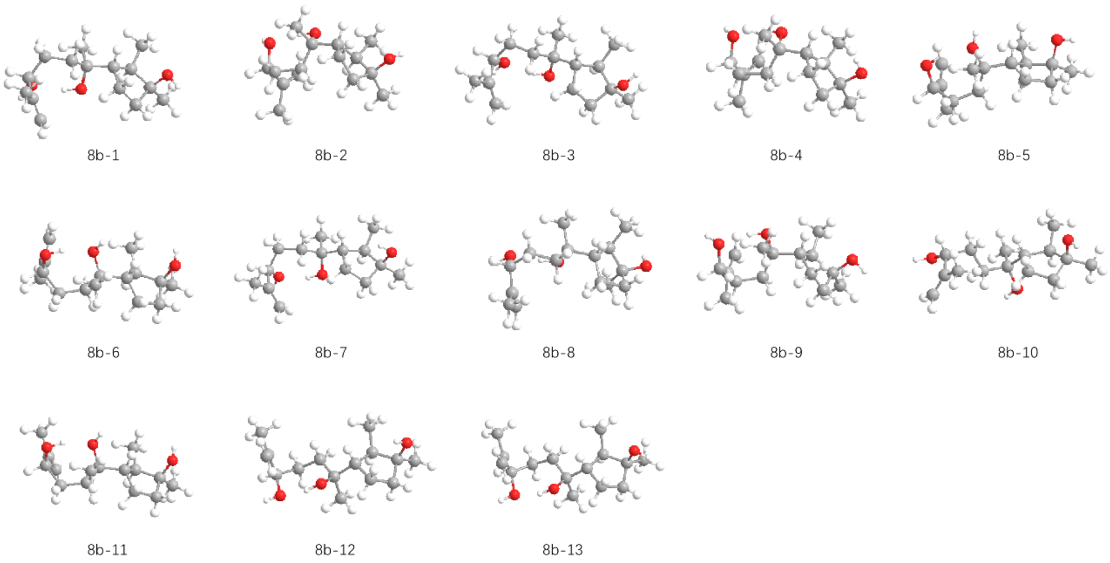

Figure S 110. Most stable conformers of 2*R*, 3*S*, 6*S*, 7*S, 10R*-8 at the CAM-B3LYP/DGDZVP level

Table S 60. Optimized Z-Matrixes of 2*R*, 3*S*, 6*S*, 7*S, 10R*-8 conformations in the methanol at CAM-B3LYP/DGDZVP level

	8b-1			8a-2			8b-3		
C	-1.18671	0.269541	-0.68402	1.380704	-1.07403	0.200815	-1.18465	0.266593	-0.67851
C	-2.46815	0.598794	0.132711	1.785908	0.353039	0.66314	-2.46664	0.589457	0.138119
C	-3.28925	-0.70549	0.166138	2.952619	0.78004	-0.26132	-3.29069	-0.72137	0.160546
C	-2.20333	-1.78009	0.1992	2.624103	0.069862	-1.57101	-2.20884	-1.79342	0.180627
C	-1.15806	-1.28024	-0.79812	2.139784	-1.3131	-1.13007	-1.1526	-1.28264	-0.80046
C	0.122085	0.849998	-0.09138	-0.14787	-1.31193	0.131359	0.122895	0.84902	-0.08533
C	1.266155	0.588821	-1.10358	-0.80984	-0.42712	-0.94981	1.266428	0.593026	-1.09947
C	2.660136	1.18108	-0.84259	-2.29315	-0.65055	-1.28793	2.659723	1.186685	-0.838
C	3.518179	0.541755	0.254694	-3.3405	-0.13296	-0.29555	3.520397	0.544319	0.255424
C	3.680299	-0.95798	0.089163	-3.16117	1.334234	0.048513	3.684682	-0.9544	0.083307
O	0.335114	0.162763	1.144481	-0.62537	-0.97995	1.439078	0.33827	0.159627	1.148739
C	-4.27082	-0.7873	1.330168	3.096543	2.291502	-0.40233	-4.26715	-0.81113	1.328375
C	3.224116	-1.83949	0.982845	-2.87231	1.759301	1.281337	3.230452	-1.84033	0.973602
C	4.402909	-1.38394	-1.16275	-3.33525	2.283817	-1.10856	4.406622	-1.37383	-1.17119
H	-1.29738	0.704509	-1.68331	1.743527	-1.78707	0.949232	-1.2964	0.704791	-1.67634
C	-3.25879	1.798517	-0.38636	2.097657	0.452336	2.154047	-3.25504	1.792174	-0.37855
O	-4.00871	-0.78531	-1.07812	4.199995	0.216212	0.181413	-4.00039	-0.91063	-1.0771
C	0.002463	2.352032	0.192828	-0.4264	-2.79971	-0.12728	-0.00113	2.350031	0.202348
O	2.971292	0.919727	1.520218	-3.33012	-0.99158	0.846988	2.974488	0.916066	1.523229
H	-2.17247	0.789036	1.170735	0.962306	1.043533	0.456515	-2.17505	0.778987	1.177494
H	-2.59778	-2.7739	-0.03899	3.486674	0.040338	-2.24232	-2.61185	-2.77644	-0.07866
H	-1.77312	-1.82269	1.204676	1.821161	0.617071	-2.07607	-1.78801	-1.85027	1.188817
H	-0.17556	-1.70044	-0.5798	1.534216	-1.80166	-1.89714	-0.17164	-1.70124	-0.57185
H	-1.42952	-1.58537	-1.8118	3.000765	-1.96105	-0.95107	-1.40933	-1.58343	-1.81915

H	0.933119	0.993182	-2.06563	-0.26074	-0.58	-1.88361	0.931737	1.00001	-2.05983
H	1.367715	-0.49104	-1.24559	-0.66771	0.623444	-0.67814	1.369318	-0.48625	-1.24502
H	2.604708	2.253219	-0.63561	-2.50198	-1.70867	-1.46791	2.602861	2.25788	-0.62657
H	3.227138	1.093782	-1.7742	-2.48913	-0.1477	-2.23942	3.225565	1.104164	-1.77073
H	4.519325	0.986944	0.172235	-4.31807	-0.23451	-0.78669	4.520648	0.99161	0.173537
H	1.220211	0.414399	1.467408	-1.59922	-1.03251	1.414021	1.223947	0.410524	1.470804
H	-5.01265	0.013816	1.269671	3.318714	2.758608	0.56321	-5.02476	-0.02163	1.275449
H	-4.80392	-1.74385	1.314977	3.912121	2.530185	-1.09052	-4.78243	-1.77549	1.313905
H	-3.74941	-0.70698	2.287798	2.175265	2.737388	-0.78673	-3.74421	-0.71113	2.283048
H	3.375834	-2.90581	0.841493	-2.76914	2.819281	1.495451	3.383445	-2.90583	0.827555
H	2.676003	-1.53562	1.869102	-2.71431	1.074501	2.108605	2.682497	-1.54099	1.861493
H	3.789832	-1.19454	-2.04983	-2.50847	2.186568	-1.81971	3.792008	-1.18209	-2.05671
H	5.334378	-0.82355	-1.29138	-4.25761	2.071741	-1.65851	5.336798	-0.81106	-1.29876
H	4.640096	-2.4488	-1.1395	-3.36562	3.32025	-0.76851	4.64594	-2.4383	-1.15259
H	-4.16	1.969355	0.210931	2.426823	1.459602	2.433241	-4.18071	1.941386	0.187945
H	-2.66204	2.712524	-0.35106	1.204155	0.216516	2.734437	-2.67659	2.714436	-0.29917
H	-3.56741	1.63761	-1.42199	2.87817	-0.25826	2.445797	-3.51471	1.667478	-1.43472
H	-4.39538	-1.66962	-1.1296	4.421967	0.617073	1.032415	-4.69392	-0.23976	-1.12977
H	-0.18749	2.921514	-0.72231	-0.16733	-3.09854	-1.14692	-0.19913	2.920527	-0.71048
H	-0.80503	2.547126	0.900859	0.151549	-3.4126	0.569926	-0.80487	2.539783	0.91611
H	0.923759	2.729095	0.644646	-1.48486	-3.02482	0.028936	0.920917	2.730523	0.649608
H	3.547979	0.58251	2.219664	-4.03425	-0.71458	1.449496	3.552176	0.576185	2.22057
	8b-4			8b-5			8b-6		
C	1.381935	-1.0665	0.204268	1.359812	-0.79749	0.252924	1.360787	0.800471	-0.24461
C	1.787466	0.362831	0.666512	1.757292	0.685435	0.119099	1.758133	-0.67938	-0.09885
C	2.959891	0.78843	-0.25282	3.26919	0.603329	-0.15744	3.279088	-0.59496	0.162186
C	2.630355	0.076391	-1.56843	3.363514	-0.55251	-1.16669	3.396806	0.588672	1.129536
C	2.140322	-1.30629	-1.12902	2.200584	-1.51067	-0.83319	2.196968	1.516559	0.84001
C	-0.1464	-1.30815	0.131553	-0.14614	-1.1442	0.238366	-0.14498	1.147366	-0.24075
C	-0.80815	-0.42271	-0.94922	-0.88249	-0.43032	-0.9047	-0.88766	0.440851	0.903065
C	-2.29003	-0.65059	-1.29071	-2.32936	-0.83955	-1.22221	-2.33654	0.851634	1.209608
C	-3.34079	-0.13874	-0.299	-3.42855	-0.34259	-0.26381	-3.43062	0.341991	0.252244
C	-3.16767	1.328549	0.047738	-3.43557	1.173012	-0.12137	-3.4355	-1.17528	0.128632
O	-0.62616	-0.97996	1.438446	-0.74072	-0.65534	1.464589	-0.732	0.649701	-1.46726
C	3.104788	2.294704	-0.39587	3.885532	1.898694	-0.67411	3.88897	-1.88036	0.710739
C	-2.88179	1.752636	1.281588	-3.35718	1.778751	1.06658	-3.35236	-1.79541	-1.05154
C	-3.34406	2.279356	-1.10799	-3.55948	1.950764	-1.40761	-3.56342	-1.93727	1.423871
H	1.741524	-1.78103	0.953809	1.733171	-1.13596	1.228755	1.741399	1.133744	-1.22007
C	2.089762	0.471927	2.158022	1.359976	1.589277	1.280692	1.350906	-1.59304	-1.25041
O	4.227902	0.332341	0.251898	3.894365	0.231031	1.082919	3.979161	-0.20399	-1.03242
C	-0.41994	-2.79637	-0.13049	-0.32674	-2.66549	0.194745	-0.32552	2.668591	-0.2068
O	-3.32841	-0.9993	0.842082	-3.41307	-1.02435	0.977322	-3.40964	1.008273	-0.99716
H	0.964718	1.050067	0.447286	1.317168	1.085152	-0.80315	1.322843	-1.07253	0.828564

H	3.492744	0.046267	-2.23968	4.343483	-1.03591	-1.11695	4.361859	1.082795	0.997751
H	1.824818	0.618531	-2.07548	3.254499	-0.15588	-2.18004	3.357715	0.218423	2.156959
H	1.53231	-1.78978	-1.89644	1.606356	-1.70155	-1.73095	1.611375	1.664298	1.751787
H	2.991366	-1.975	-0.96328	2.553842	-2.48136	-0.47972	2.511916	2.507051	0.505729
H	-0.2572	-0.57106	-1.88261	-0.29334	-0.58654	-1.81362	-0.30417	0.603917	1.814214
H	-0.67048	0.627555	-0.67445	-0.85389	0.645097	-0.70204	-0.85733	-0.63606	0.708669
H	-2.49484	-1.70896	-1.47363	-2.42054	-1.92662	-1.29779	-2.42974	1.939271	1.272682
H	-2.48572	-0.14612	-2.24134	-2.56385	-0.45485	-2.2196	-2.57485	0.47797	2.210235
H	-4.3172	-0.24347	-0.79171	-4.38497	-0.61623	-0.72639	-4.38952	0.620371	0.706751
H	-1.59998	-1.03453	1.411788	-0.29671	-1.08431	2.20965	-0.29272	1.083641	-2.21227
H	3.306423	2.759627	0.573202	3.835612	2.682689	0.085766	3.840937	-2.68916	-0.02566
H	3.932931	2.534041	-1.06884	4.93886	1.744165	-0.93188	4.940262	-1.71721	0.963492
H	2.189759	2.729682	-0.80482	3.367811	2.248832	-1.57117	3.362924	-2.21077	1.610318
H	-2.78287	2.812631	1.497582	-3.3813	2.86241	1.146729	-3.37521	-2.87999	-1.11859
H	-2.72225	1.067155	2.107996	-3.26762	1.211267	1.987279	-3.26067	-1.23879	-1.97866
H	-2.516	2.186719	-1.81827	-2.64001	1.888861	-1.99887	-2.64641	-1.86682	2.017965
H	-4.26486	2.064498	-1.65945	-4.36658	1.552466	-2.03147	-4.37347	-1.53229	2.039531
H	-3.37902	3.315066	-0.76622	-3.76292	3.005716	-1.21406	-3.7648	-2.99479	1.242601
H	2.402111	1.485916	2.427128	1.766101	2.597201	1.151976	1.782014	-2.59296	-1.13715
H	1.197466	0.220496	2.734202	0.273409	1.667411	1.351382	0.265747	-1.69831	-1.29558
H	2.889956	-0.21279	2.454757	1.730582	1.190768	2.228791	1.671523	-1.18472	-2.21475
H	4.177222	-0.62013	0.407804	4.831689	0.080496	0.902743	3.884474	-0.91714	-1.67834
H	-0.16038	-3.09292	-1.15064	-0.0713	-3.07459	-0.78562	-0.07397	3.081861	0.772862
H	0.159133	-3.40918	0.565811	0.317426	-3.1451	0.939093	0.322372	3.144299	-0.95042
H	-1.47782	-3.02422	0.025163	-1.36158	-2.93323	0.419214	-1.35917	2.935541	-0.43747
H	-4.03464	-0.72656	1.444082	-2.51236	-0.91977	1.345696	-2.50592	0.903874	-1.35806
	8b-7			8b-8			8b-9		
C	-1.18656	0.267505	-0.67854	-1.23337	0.475776	-0.72309	1.379677	-1.06022	0.213315
C	-2.47069	0.594093	0.136906	-2.49097	0.616321	0.185485	1.787803	0.37121	0.662576
C	-3.29656	-0.71622	0.164061	-3.16676	-0.79058	0.186539	2.967434	0.773044	-0.24573
C	-2.20935	-1.79072	0.194117	-2.68778	-1.42181	-1.11997	2.635671	0.063006	-1.5607
C	-1.15051	-1.2844	-0.78887	-1.22997	-0.98506	-1.22392	2.140726	-1.31379	-1.11423
C	0.121869	0.851143	-0.08743	0.092583	0.923154	-0.07201	-0.14849	-1.30013	0.139947
C	1.264856	0.591997	-1.10143	1.238731	0.664387	-1.07853	-0.8088	-0.42015	-0.94628
C	2.658537	1.184534	-0.83987	2.64039	1.216034	-0.77584	-2.28858	-0.65278	-1.29354
C	3.517033	0.543647	0.25608	3.466989	0.508289	0.303492	-3.34529	-0.14249	-0.30725
C	3.680448	-0.95558	0.087443	3.61593	-0.9826	0.061059	-3.17903	1.326121	0.03745
O	0.335254	0.162993	1.146977	0.248688	0.135563	1.110823	-0.63277	-0.96491	1.444215
C	-4.27211	-0.80026	1.326544	-2.71689	-1.59902	1.404187	3.146737	2.280107	-0.39149
C	3.225076	-1.8392	0.979454	3.140718	-1.90416	0.902996	-2.90237	1.753789	1.272179
C	4.403449	-1.37834	-1.16533	4.34838	-1.35136	-1.20328	-3.35183	2.27403	-1.12119
H	-1.29405	0.701845	-1.67931	-1.38258	1.127086	-1.5929	1.740728	-1.76546	0.969873
C	-3.26076	1.794473	-0.38013	-3.41819	1.726531	-0.3099	2.074991	0.49686	2.156026

O	-4.10334	-0.86072	-1.01916	-4.59726	-0.74927	0.150691	4.156644	0.198188	0.324704
C	-0.00154	2.352527	0.196969	0.027258	2.406443	0.313226	-0.42333	-2.78952	-0.11454
O	2.969317	0.918539	1.522198	2.895763	0.822414	1.575641	-3.33518	-1.00057	0.835658
H	-2.17378	0.782242	1.174831	-2.18502	0.854097	1.208497	0.975055	1.064244	0.423605
H	-2.60943	-2.77708	-0.05631	-3.27262	-0.99639	-1.94344	3.494753	0.021018	-2.23845
H	-1.78221	-1.83806	1.200372	-2.82444	-2.5069	-1.13522	1.838586	0.613502	-2.07267
H	-0.16996	-1.69771	-0.5519	-0.60192	-1.6051	-0.57754	1.534009	-1.80176	-1.8805
H	-1.38403	-1.60149	-1.81112	-0.84035	-1.07349	-2.24075	2.996142	-1.96716	-0.92808
H	0.931133	0.998299	-2.0624	0.928946	1.09955	-2.03522	-0.25281	-0.56961	-1.87657
H	1.367046	-0.48755	-1.24572	1.311305	-0.41543	-1.24075	-0.67507	0.631257	-0.67387
H	2.602467	2.256252	-0.6311	2.600515	2.278117	-0.51913	-2.48958	-1.71212	-1.47529
H	3.225069	1.09909	-1.77187	3.220381	1.160536	-1.70176	-2.48208	-0.15062	-2.24592
H	4.517651	0.99011	0.174762	4.474656	0.944786	0.266652	-4.31924	-0.25131	-0.80404
H	1.220824	0.413624	1.469973	1.135559	0.328555	1.467688	-1.60606	-1.02402	1.41517
H	-5.01974	-0.00434	1.26783	-3.08551	-1.13251	2.32489	3.350719	2.740304	0.579238
H	-4.79509	-1.7606	1.312522	-3.10622	-2.62005	1.351454	3.987248	2.504615	-1.05698
H	-3.74242	-0.70875	2.277575	-1.62713	-1.63149	1.471183	2.249707	2.741795	-0.81313
H	3.377671	-2.90511	0.836013	3.28334	-2.96336	0.708603	-2.80859	2.814553	1.486709
H	2.676944	-1.5376	1.86647	2.5864	-1.64065	1.798256	-2.74538	1.070404	2.100807
H	3.790213	-1.18789	-2.05209	3.744221	-1.11645	-2.08559	-2.51901	2.183027	-1.82609
H	5.334335	-0.81676	-1.2929	5.283522	-0.79007	-1.29645	-4.26838	2.054678	-1.67795
H	4.641727	-2.44299	-1.14416	4.580564	-2.41727	-1.22931	-3.39278	3.310278	-0.78168
H	-4.16914	1.954007	0.20889	-4.27725	1.86889	0.351482	2.384454	1.513829	2.418526
H	-2.67001	2.711601	-0.33309	-2.87348	2.674311	-0.35997	1.175471	0.25402	2.724827
H	-3.56153	1.646775	-1.42114	-3.80015	1.506064	-1.31161	2.871916	-0.18663	2.459792
H	-3.52615	-0.82525	-1.79371	-4.90762	-0.41194	1.001547	4.873285	0.330911	-0.31006
H	-0.19579	2.921559	-0.71748	-0.05557	3.043853	-0.57278	-0.16404	-3.09102	-1.13333
H	-0.80719	2.544955	0.907741	-0.83225	2.599284	0.959102	0.156304	-3.39902	0.584284
H	0.920096	2.731748	0.646015	0.923587	2.701809	0.865017	-1.48123	-3.01664	0.042557
H	3.546403	0.581301	2.221315	3.459283	0.450395	2.26815	-4.04563	-0.72932	1.433318
	8b-10			8b-11			8b-12		
C	-1.00764	0.182298	-0.21748	1.367677	0.744351	-0.33698	1.135352	-0.12563	0.596777
C	-2.51045	0.58484	-0.20069	1.800221	-0.70159	-0.03292	2.087354	0.743195	-0.27628
C	-3.28891	-0.72783	0.020104	3.332247	-0.56914	0.12231	3.521201	0.201461	-0.02205
C	-2.34213	-1.53327	0.910427	3.485581	0.727139	0.926107	3.276463	-1.27565	0.29856
C	-0.96194	-1.2793	0.303862	2.256875	1.600604	0.59315	2.022244	-1.2597	1.17458
C	-0.08738	1.140061	0.568068	-0.1409	1.075286	-0.27992	-0.13303	-0.66099	-0.11601
C	1.38897	0.69786	0.513233	-0.79747	0.509877	0.986611	-0.96405	0.531557	-0.63758
C	1.978312	0.503605	-0.88256	-2.23555	0.930082	1.332782	-2.23954	0.261337	-1.45283
C	3.47444	0.174985	-0.87134	-3.38153	0.40072	0.455911	-3.48405	-0.24963	-0.71909
C	3.792292	-1.06297	-0.05049	-3.48858	-1.12135	0.35651	-3.88167	0.614493	0.463014
O	-0.51683	1.071796	1.940152	-0.7953	0.415184	-1.39258	-0.83452	-1.36061	0.917871
C	-4.67538	-0.52648	0.622039	3.996528	-1.76896	0.788876	4.465682	0.426041	-1.19183

C	4.568909	-1.02359	1.035797	-3.05555	-1.95027	1.312993	-3.90642	0.158189	1.717887
C	3.163042	-2.33936	-0.54629	-4.20027	-1.6434	-0.8647	-4.25875	2.031375	0.114855
H	-0.66527	0.206028	-1.25636	1.681355	0.955401	-1.369	0.75378	0.485428	1.422684
C	-2.96989	1.364561	-1.43141	1.342468	-1.75679	-1.0351	1.960679	2.247369	-0.04018
O	-3.40683	-1.37052	-1.26228	3.953307	-0.32262	-1.15197	4.146191	0.852484	1.097443
C	-0.2226	2.587424	0.081511	-0.34459	2.586789	-0.42736	0.189632	-1.64229	-1.25049
O	4.162642	1.339882	-0.43275	-3.40053	1.01503	-0.82776	-3.26551	-1.62022	-0.37718
H	-2.69169	1.193461	0.691984	1.42792	-0.98036	0.960825	1.878037	0.548173	-1.33412
H	-2.6147	-2.59323	0.951644	4.433834	1.209178	0.678383	4.146828	-1.73223	0.777516
H	-2.38405	-1.13619	1.929507	3.509983	0.487903	1.992149	3.081349	-1.81678	-0.63334
H	-0.17026	-1.44663	1.035192	1.72501	1.861444	1.512379	1.50579	-2.22037	1.199348
H	-0.78614	-1.9651	-0.52909	2.536402	2.541431	0.114949	2.28737	-1.03735	2.214073
H	1.491153	-0.23026	1.082316	-0.16817	0.798429	1.834313	-0.31695	1.119571	-1.2943
H	1.985253	1.44685	1.048499	-0.74879	-0.57977	0.925909	-1.20375	1.173207	0.218343
H	1.849939	1.40405	-1.49095	-2.32017	2.020619	1.347989	-2.0365	-0.42959	-2.27585
H	1.459722	-0.30411	-1.40643	-2.42729	0.609915	2.361757	-2.52332	1.204753	-1.92834
H	3.764886	-0.04398	-1.90984	-4.31348	0.737095	0.925552	-4.31137	-0.2091	-1.44112
H	0.055833	1.654847	2.456622	-0.38432	0.723503	-2.21262	-1.70376	-1.61986	0.558615
H	-5.30291	0.075055	-0.04132	3.919912	-2.66466	0.164074	4.583479	1.493779	-1.39747
H	-5.17118	-1.49097	0.774091	5.057736	-1.56439	0.955452	5.451823	0.01047	-0.96716
H	-4.61146	-0.02364	1.590725	3.530183	-1.98886	1.752867	4.082127	-0.05975	-2.09207
H	4.793479	-1.9266	1.596792	-3.21119	-3.02317	1.234281	-4.21775	0.798742	2.538134
H	4.988594	-0.09319	1.406067	-2.54163	-1.60185	2.203755	-3.60472	-0.8528	1.973011
H	2.07977	-2.32605	-0.38346	-5.17289	-1.15672	-0.98578	-3.37953	2.601411	-0.20257
H	3.322922	-2.4682	-1.62158	-3.62822	-1.42896	-1.7714	-4.9738	2.057677	-0.71354
H	3.56946	-3.21018	-0.02886	-4.35358	-2.72247	-0.79771	-4.70116	2.544562	0.970225
H	-4.04195	1.580214	-1.38692	1.799827	-2.72937	-0.82672	2.687254	2.804269	-0.63929
H	-2.44573	2.319241	-1.51348	0.258978	-1.88071	-0.99871	0.963642	2.606086	-0.30609
H	-2.77856	0.795345	-2.34401	1.59593	-1.46821	-2.06083	2.132206	2.497987	1.010055
H	-3.75495	-2.25889	-1.10757	3.836074	-1.11254	-1.69699	3.591482	0.728657	1.879246
H	-0.0339	2.674098	-0.99166	-0.01935	3.122984	0.467359	0.681171	-1.14995	-2.09449
H	-1.22417	2.9713	0.28608	0.232065	2.967006	-1.27708	0.834463	-2.4491	-0.89578
H	0.495406	3.23034	0.60177	-1.39817	2.816319	-0.5999	-0.7291	-2.10274	-1.62283
H	5.110777	1.18014	-0.52936	-2.54081	0.809934	-1.2464	-4.07491	-1.97514	0.015937
	8b-13								
C	1.136377	-0.13063	0.598694						
C	2.08281	0.744478	-0.27299						
C	3.510737	0.207703	-0.02438						
C	3.272424	-1.2732	0.279514						
C	2.0291	-1.26222	1.16806						
C	-0.13271	-0.66389	-0.11444						
C	-0.96361	0.529242	-0.6352						
C	-2.2396	0.261304	-1.45059						

C	-3.48515	-0.24835	-0.71769						
C	-3.88135	0.614943	0.46547						
O	-0.83611	-1.36437	0.918238						
C	4.463901	0.451988	-1.18942						
C	-3.90705	0.157156	1.719778						
C	-4.25561	2.033008	0.11907						
H	0.758472	0.476729	1.428452						
C	1.951099	2.247691	-0.03078						
O	4.01119	0.864812	1.153244						
C	0.187731	-1.64469	-1.25012						
O	-3.26948	-1.61984	-0.37763						
H	1.875601	0.552271	-1.33205						
H	4.144275	-1.74309	0.747199						
H	3.076633	-1.80483	-0.65803						
H	1.515201	-2.22451	1.199324						
H	2.317319	-1.02035	2.19395						
H	-0.31641	1.117351	-1.29173						
H	-1.20247	1.17058	0.221199						
H	-2.03758	-0.42938	-2.27412						
H	-2.52216	1.205421	-1.92553						
H	-4.31237	-0.20519	-1.43974						
H	-1.70528	-1.62184	0.558067						
H	4.569283	1.52287	-1.38458						
H	5.456899	0.049148	-0.96412						
H	4.1014	-0.03328	-2.09973						
H	-4.21714	0.797297	2.540824						
H	-3.60692	-0.85461	1.973673						
H	-3.37515	2.601615	-0.19748						
H	-4.97048	2.061811	-0.70941						
H	-4.69708	2.546032	0.975025						
H	2.66864	2.811572	-0.63481						
H	0.950002	2.601323	-0.28868						
H	2.132457	2.486833	1.019308						
H	4.836294	0.425246	1.398581						
H	0.681643	-1.1524	-2.0929						
H	0.830476	-2.45311	-0.89537						
H	-0.73172	-2.10248	-1.62426						
H	-4.07927	-1.97304	0.01622						

Table S 61. Energy analysis for 2*R*, 3*S*, 6*S*, 7*S*, *10R*-8

Conf.	Steric Energy	Relative Energy	Distribution
	(kJ/mol)	(kJ/mol)	(%)
8b-1	-813.602253	0.001538	5.52%

8b-2	-813.602676	0.001115	8.63%
8b-3	-813.602078	0.001713	4.58%
8b-4	-813.601677	0.002114	3.00%
8b-5	-813.603344	0.000447	17.51%
8b-6	-813.603791	0.000000	28.10%
8b-7	-813.602024	0.001767	4.33%
8b-8	-813.601666	0.002125	2.96%
8b-9	-813.602604	0.001187	8.00%
8b-10	-813.602964	0.000827	11.71%
8b-11	-813.601162	0.002629	1.74%
8b-12	-813.601235	0.002556	1.88%
8b-13	-813.601317	0.002474	2.05%

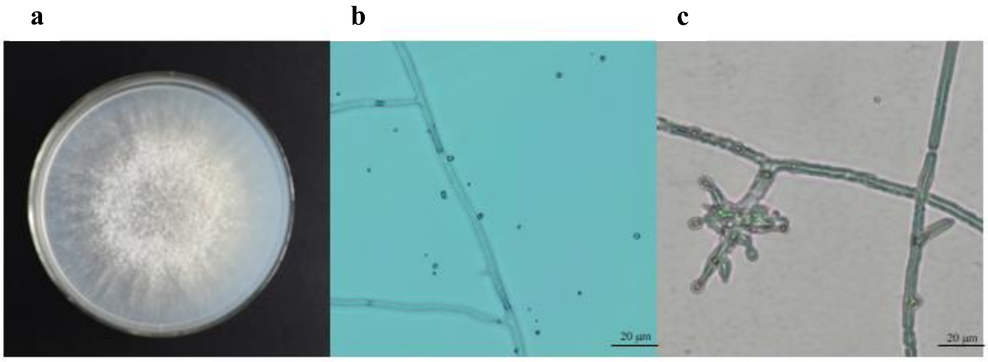

Figure S 111 Morphological characteristics of b-3. **a** Characteristics of colony of strain b-3 on PDA. **b** Conidia of strain b-3 on PDA. **c** Spores of strain b-3.
